# Cross-Scale Variation in Biodiversity-Environment Links Illustrated by Coastal Sandflat Communities

**DOI:** 10.1371/journal.pone.0142411

**Published:** 2015-11-10

**Authors:** Casper Kraan, Carsten F. Dormann, Barry L. Greenfield, Simon F. Thrush

**Affiliations:** 1 National Institute of Water and Atmospheric Research, Hamilton, New Zealand; 2 Biometry and Environmental System Analysis, University of Freiburg, Freiburg, Germany; 3 Institute for Marine Science, University of Auckland, Auckland, New Zealand; University of Sydney, AUSTRALIA

## Abstract

Spatial variation in the composition of communities is the product of many biotic and environmental interactions. A neglected factor in the analysis of community distribution patterns is the multi-scale nature of the data, which has implications for understanding ecological processes and the development of conservation and environmental management practice. Drawing on recently established multivariate spatial analyses, we investigate whether including relationships between spatial structure and abiotic variables enable us to better discern patterns of species and communities across scales. Data comprised 1200 macrozoobenthic samples collected over an array of distances (30 cm to 1 km) in three New Zealand harbours, as well as commonly used abiotic variables, such as sediment characteristics and chlorophyll a concentrations, measured at the same scales. Moran’s eigenvector mapping was used to extract spatial scales at which communities were structured. Benthic communities, representing primarily bivalves, polychaetes and crustaceans, were spatially structured at four spatial scales, i.e. >100 m, 50–100 m, 50–15 m, and < 15 m. A broad selection of abiotic variables contributed to the large-scale variation, whereas a more limited set explained part of the fine-scale community structure. Across all scales, less than 30% of the variation in spatial structure was captured by our analysis. The large number of species (48) making up the 10 highest species scores based on redundancy analyses illustrate the variability of species-scale associations. Our results emphasise that abiotic variables and biodiversity are related at all scales investigated and stress the importance of assessing the relationship between environmental variables and the abundance and distribution of biological assemblages across a range of different scales.

## Introduction

Community composition is an integrative response variable encompassing demography, functional traits, and species interactions, influenced by heterogeneity in environmental conditions [1,2]. Broad-scale biodiversity and ecological studies predominantly focus on environmental correlatives of species communities, such as land use or land cover, typically limited to a single spatial scale dictated by available data (but see, e.g. [3]). However, community composition and many factors underpinning ecology are scale-dependent (e.g. [2]). Therefore, the structuring factors underlying heterogeneity of communities should not be tied to a single scale. Moreover, the importance of specific factors likely changes with scale. Neglecting such variation affects assessments of biodiversity and may fail to maximise the extraction of information from available data. Although the role of spatial scale and cross-scale interactions is generally recognised as an important structuring factor in ecosystems [4,5], it is only recently becoming integrated in statistical approaches aimed at downscaling broad-scale data to enable predictive modelling of fine-grained diversity (e.g. [3,6]).

Defining across which scales species, communities and environmental characteristics are related is critical to advance ecological insight into the spatial organisation of communities and ecosystem dynamics [4,7,8]. Such spatial organisation occurs along a continuum from communities displaying certain regular patterns with clear patch boundaries often at a single scale (reviewed by [9]) to others showing multi-scale patterning and fuzzy patch boundaries (e.g. [10,11]). A common simplifying assumption is that biotic processes, such as competition, facilitation or predation [12,13], dominate fine-scale variation, whereas at broader spatial scales abiotic variables, such as hydrodynamics or climatic variables, drive spatial variation [13,14].

Ideally, in addition to embracing scale-dependency, studies addressing spatial variation in community data should apply spatially explicit multivariate models, which encompass multiple predictors and multivariate response variables. Moran’s eigenvector mapping [2,15] is one of the tools available, and employed here. This spatially explicit framework allows us to infer the scale-dependent association between abiotic variables and community distributions without *a priori* assigning scales, rather letting the data speak for themselves within constraints set by the sampling design. The aim is to understand whether including spatial variation and abiotic variables enables us to differentiate the scale-dependent patterns of communities across scales (from 30 cm to 1 km).

We use a large data set on macrozoobenthic communities, primarily bivalves, crustaceans, and polychaetes, from three estuarine areas of New Zealand explicitly collected for such cross-scale analysis. 1) We explore generality of relationships between abiotic variables and community composition by dissecting variation in species assemblages across a broad set of spatial scales and across multiple sites. In contrast to many species distribution analyses restricted to a single scale and a single species (as criticised by [16,17]), this approach embraces potential changing relevance of abiotic variables with scale. 2) We statistically assess scale-dependent patterns in community-environment relationships. Thereby we discern co-occurrence of species and specific habitat features.

## Materials and Methods

### Community Data

**Fig 1:**
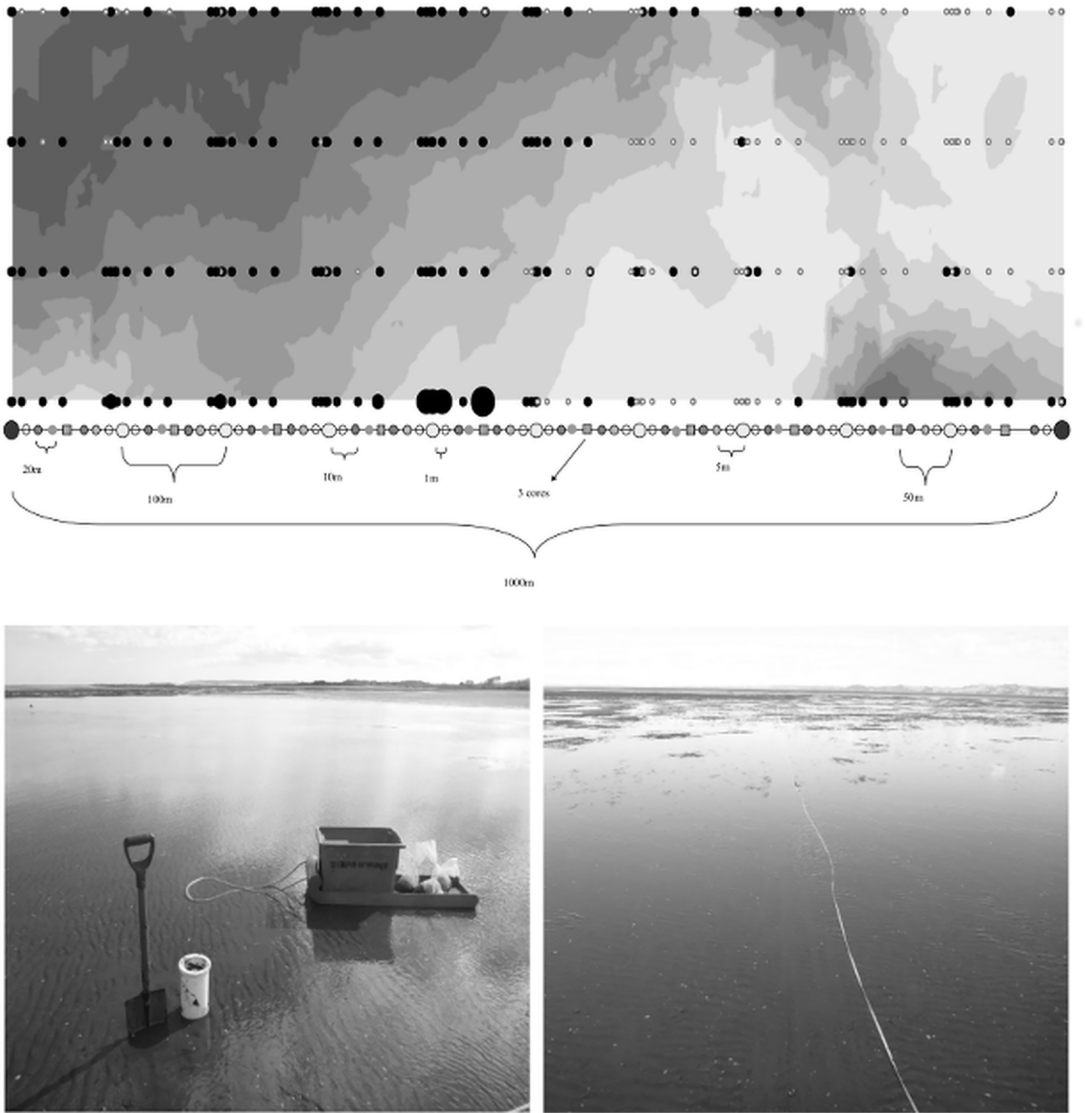
Sampling design matching a number of spatial lags ranging from 0.3 m to 1 km. Illustrated are the abundances of *Macomona liliana* (scaled dots, encompassing values between 0 and 40 ind./core) across a sandflat of 300 m by 1000 m at Kaipara Harbour. The background displays an interpolated seascape of median grain size, ranging between 170 and 250 μm (darker grey indicated a larger median grain size, i.e. coarser sands). The middle panel (not to scale) illustrates how sampling points were positioned along a single transect, where the label “3 cores” indicates a sampling distance of 30 cm. The two bottom panels show a sampling area at low tide.

The abundance and distribution of benthic infauna was sampled in Kaipara (175°56’ S, 37°27’ E), Manukau (174°41’ S, 37°7’ E), and Tauranga (174°17’ S, 36°23’ E) harbours, North Island, New Zealand in the austral summer of 2012 (see, e.g. [18,19] for area descriptions). 400 cores (13 cm diam., 20 cm deep) were sampled on a pre-determined grid (Fig 1) in each harbour during low tide. Transects had a length of 1 km, and the distance between transects was 100 m. This grid was designed to allow sampling at multiple spatial scales and encompass patterns on scales from 30 cm to 1 km (Fig 1), advancing the sampling design previously employed by [20]. Sampling points along transects were spaced at distances of 30 cm, 1 m, 5 m, 10 m, 30 m, 50 m, 100 m, 500 m and 1000 m (Fig 1). This grid covered the intertidal area from the high- to low-water mark to capture tidal variation. Cores were sieved (500 μm mesh) and the residue preserved with 70% isopropyl alcohol. In the laboratory, Rose Bengal (2%) stained species were identified to the lowest practical taxonomic resolution and their abundance assessed (see S1 Appendix). (No specific permissions were required for sampling these locations, as our sampling is a permitted activity. Field studies did not involve endangered or protected species)

### Environmental Variables

Prior to sampling the seafloor surface, at each sampling point (*n* = 1200) on the grid a photograph of 0.25-m^2^ of the sediment surface was taken. Coverage (%) of seagrass (*Zostera mulleri*), bare sand, and shell hash (i.e. broken shell fragments) was estimated based on 75 random points within that photograph using CPCe [21]. To determine sediment median grain size (μm), % sediment fractions (silt < 63 μm, very fine 63–125 μm, fine 125–250 μm, medium 250–500 μm, and coarse > 500 μm), organic content (%), and chlorophyll *a* concentration (mg/g) at each point, we pooled three surface sediment cores (2 cm diam., 2 cm deep) from each sampling point (*n* = 1200). These samples were stored in the dark on ice immediately after collecting and freeze-dried upon arrival in the laboratory. Prior to freeze-drying, seagrass and bivalves were removed from the sediment samples. Sediment grain sizes were measured using a Malvern Mastersizer, chlorophyll *a* concentrations were determined using a fluorometer, and loss on ignition was used to assess organic content (see [22,23] for methodological details). These abiotic variables are commonly associated with coastal benthic diversity (amongst others [10–14, 18–20]).

### Statistical Approach

Moran’s eigenvector mapping (MEM, [2,15,24]) was used to evaluate spatial variation in benthic community composition and abiotic variables on multiple scales for each harbour separately. This method is a modification of the Principal Coordinates of Neighbour Matrices approach (PCNM, [25]), using a distance-based (Euclidean) connectivity matrix to define how points are linked across space (see [24,26]).

Employing the MEM-framework involved several steps: 1) Community data were Hellinger-transformed to reduce the importance of the most abundant species [27]. Preliminary analyses indicated that other species transformations, such as relying only on presence-absence information, resulted in poorer model performances; 2) transformed community data were linearly detrended using geographical coordinates to remove a large-scale spatial gradient, and residuals of this model were retained for further analyses (see [24]).

**Fig 2:**
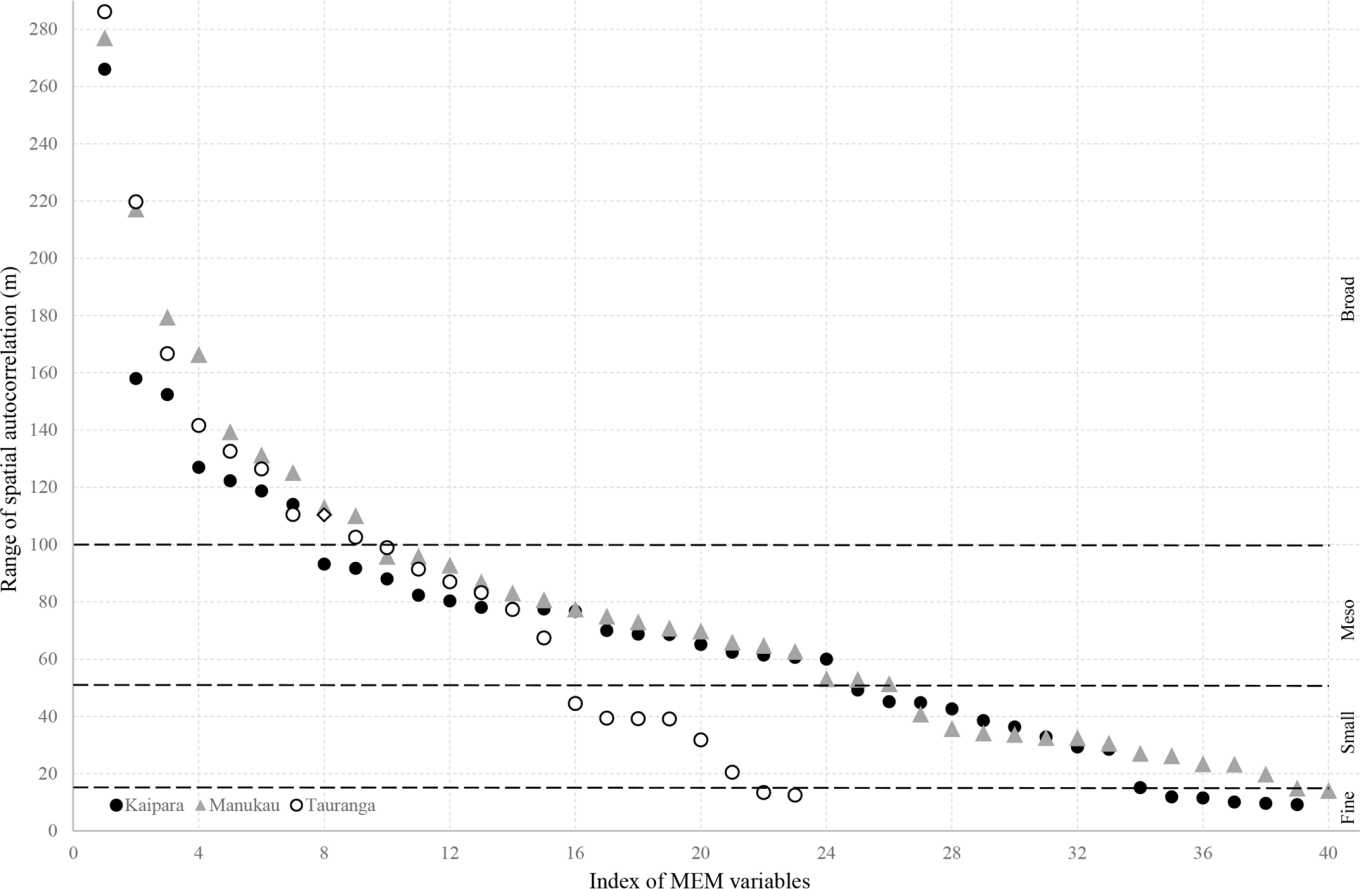
Range of spatial autocorrelation of each significant positive MEM variable. Broad: MEM variables with a range > 100 m; Meso: MEM variables with a range < 100 m and > 50 m; Small: MEM variables with a range < 50 m and > 15 m; Fine: MEM variables with a range < 15 m. Delineation into 4 distinct spatial scales is based on visual appraisal (see Materials and Methods).

Next, (3) we constructed a spatial weighting matrix (SWM) to define linkages between sampling points, used for the decomposition in orthogonal spatial variables. We trialled connectivity based on Delaunay triangulation, minimum spanning tree, relative neighbourhood, nearest neighbours, Gabriel neighbourhood, and distance thresholds (see [26]), selecting a distance-based SWM (Table 1). This particular matrix optimised performance, as determined by the AICc (Table 2), and reflects a data-driven approach [15,24,26]. Subsequently, (4) this SWM was used in eigen decomposition of community data, providing spatial eigen functions (“MEM-variables”) that can be used as spatial predictors in ordination approaches (see, e.g. [2]). Significant positive MEM-variables, representing positive spatial autocorrelation (*p* ≤ 0.05, 9999 permutations), were grouped (Fig 2) based on a visual comparison of similarities in their range of spatial autocorrelation. This represents a routine method of clustering as single MEM-variables harbour little significance [28,29]. This grouping in MEM-subsets was constrained by our sampling design, such that “spatial scales” were limited between the smallest (30 cm) and largest (1 km) inter-sample distance.

**Table 1:** Optimal connectivity network.

	Kaipara	Tauranga	Manukau
Optimal distance (m)	104	199	131
AIC_c_	-355	-540	-571
N_var_	40	26	4
α	2	3	5
R_adj_	28	19	41
Positive MEM-variables (*n*)	39	23	40

AIC_c_ (corrected Akaike Information Criterion), N_var_ (number of MEM variables), α (parameter of concave spatial weighting function dictating how similarity decays with distance; see [26]), and R_adj._ (% adjusted explained variance) summarise the optimal connectivity. See Materials and Methods for details.

**Table 2:** Mean environmental characteristics (standard deviation) and number of macrozoobenthic species encountered during macrobenthic sampling. Highest mean environmental characteristics, as identified by single variable ANOVA’s, in bold.

	Kaipara	Tauranga	Manukau
Sampling 2012	18 & 19 April	23 & 24 April	4 & 5 May
Species (*n*)	114	81	109
Individuals (*n*)	21846	25394	26573
Median grain size (μm)	**213** (14.7)	197 (23.4)	166 (35.1)
Silt (%)	1 (2.3)	5 (3.1)	**14** (10.5)
Very fine sediments (%)	6 (2.9)	17 (4.8)	**17** (5.9)
Fine sediments (%)	**61** (4.6)	44 (5.7)	48 (10.8)
Medium sediments (%)	**32** (6.5)	28 (5.4)	18 (7.2)
Coarse sediments (%)	0.4 (0.5)	**6** (3.4)	3 (4.7)
Organic content (%)	0.8 (0.2)	2 (0.6)	**2** (1.1)
Chlorophyll *a* (mg/g)	5 (3.1)	11 (4.2)	**23** (7.4)
Bare sand cover (%)	**84** (28.1)	73 (18.9)	79 (23.5)
Shell hash cover (%)	2 (3.3)	3 (4.2)	**16** (17.6)
Sea grass cover (%)	13 (27.2)	**23** (18.3)	5 (18.4)

**Fig 3:**
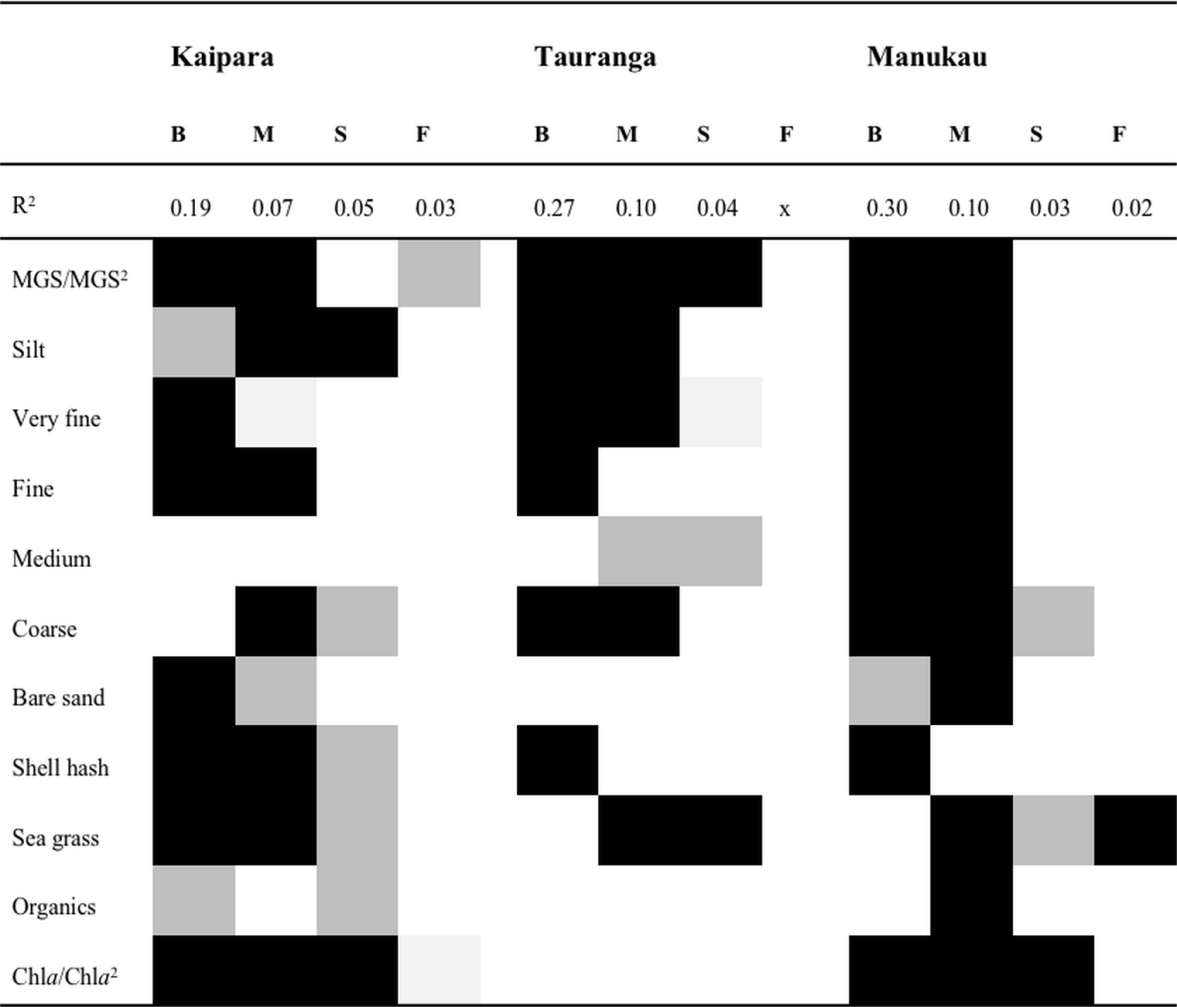
Environmental variables linked to community distributions at each distinct scale. Blocks from left to right represent b(road) scale (> 100 m), m(eso) scale (< 100 m and > 50 m), s(mall) scale (< 50 m and > 15 m) and f(ine) scale (< 15 m) spatial subsets. Fine scale spatial subset for Tauranga not shown, since none of the environmental variables linked to this scale. Light grey = p ≤ 0.05; dark grey = p ≤ 0.01; black = p ≤ 0.001.

Then, (5) the spatial variables of each MEM-subset were used in a redundancy analysis (RDA) with environmental variables in order to identify the abiotic variables linked to that scale. We included quadratic functions of abiotic variables, in case of continuous variables, to enhance fitting more complex relationships (e.g. [25,30]). Few missing abiotic variables (*n* = 2 in Tauranga and Manukau, *n* = 19 in Kaipara) values were estimated using interpolations based on inverse distance weighting (e.g. [31,32]). Forward selection with a significance level of 0.05 and 9999 random permutations of explanatory variables was then used to obtain the model with the most parsimonious set of abiotic variables ([33], see Fig 3). Finally, (6) we identified characteristic species, defined as the 10 benthic species with the highest positive or negative scores on the first two environmental ordination axes in the RDA of species abundances and environmental variables for each MEM-subset (e.g. [34]).

To determine how much of the captured community variation was related to measured abiotic variables or spatial structure we performed variance partitioning (see, e.g. [24,26]) in each harbour for each spatial submodel.

All analyses were done in R [35] using the packages *spacemakeR*, *ncf*, *packfor*, *spdep*, and *vegan*. Detailed explanations of the MEM-framework and variation partitioning, including R-scripts and formal matrix notations, can be found in [2,24], and [26].

## Results

### Benthic Communities

Across the three sites, we identified 146 taxa, comprising 73813 individuals (Table 2). Species accumulation curves indicated these were representative of the complete benthic communities present (Greenfield, Kraan, and Thrush, *unpublished data*). The spionid polychaetes *Prionospio aucklandia* (*n* = 9142, 813 sampling points) and *Aonides trifida* (*n* = 8097, 674 sampling points) were the most abundant, followed by the bivalves *Austrovenus stutchburyi* (*n* = 6520, 786 sampling points), *Macomona liliana* (*n* = 5347, 1135 sampling points) and *Nucula hartvigiana* (*n* = 4638, 524 sampling points). Concentrating on individual sites, in Kaipara *A*. *trifida* (*n* = 3915, 229 sampling points) was the most abundant species, whereas *M*. *liliana* (*n* = 1952, 379 sampling points) was the most widespread species. *A*. *stutchburyi* (*n* = 4235, 281 sampling points) was the most abundant species in Manukau. *M*. *liliana* (*n* = 1936, 384 sampling points) again was the most widespread species. In Tauranga, the most abundant and most widely distributed species was *P*. *aucklandia* (*n* = 7214, 385 sampling points).

The three locations differed significantly in this set of environmental characteristics (single variable ANOVA’s, df = 2, residuals = 1195, all *p*-values ≤ 0.05). Manukau Harbour was the muddiest site (identified by the highest % silt) and contained the highest concentration of chlorophyll *a* (Table 2). The coarsest sediment was encountered in Tauranga Harbour, which also contained the highest coverage of sea grass and shell hash. Kaipara Harbour had the highest coverage of bare sand (Table 2). All three sites had a median grain size classified as fine sands.

### Spatial Scales

The best spatial weighting matrix was based on a different optimal distance for all three harbours, i.e. Kaipara 104 m, Tauranga 199 m, and Manukau 131 m. This optimal distance indicates the spatial distance across which sampling points share similar communities. Across the three estuaries, broad-scale subsets captured 19% to 30% of the variation in benthic community composition (Fig 3). Meso-scale subsets explained 7% to 10% of the variation, whereas small-scale and fine-scale subsets captured between 2% and 5% of the variation (Fig 3). For Tauranga none of the available explanatory variables explained the fine-scale variation in community composition (Fig 3).

At spatial scales larger than 50 m most of the selected abiotic explanatory variables contributed significantly to explaining community composition, particularly at Kaipara and Manukau sites (Fig 3). In Tauranga only the smaller grain-size fractions related to community patterns. Small- and fine-scale subsets in all sites were mostly associated with coverage of seagrass and chlorophyll *a* content. There was no consistent pattern of relationships between abiotic variables and community patterns across spatial scales (Fig 3). A common feature was the decrease of explanatory power at finer scales.

**Fig 4:**
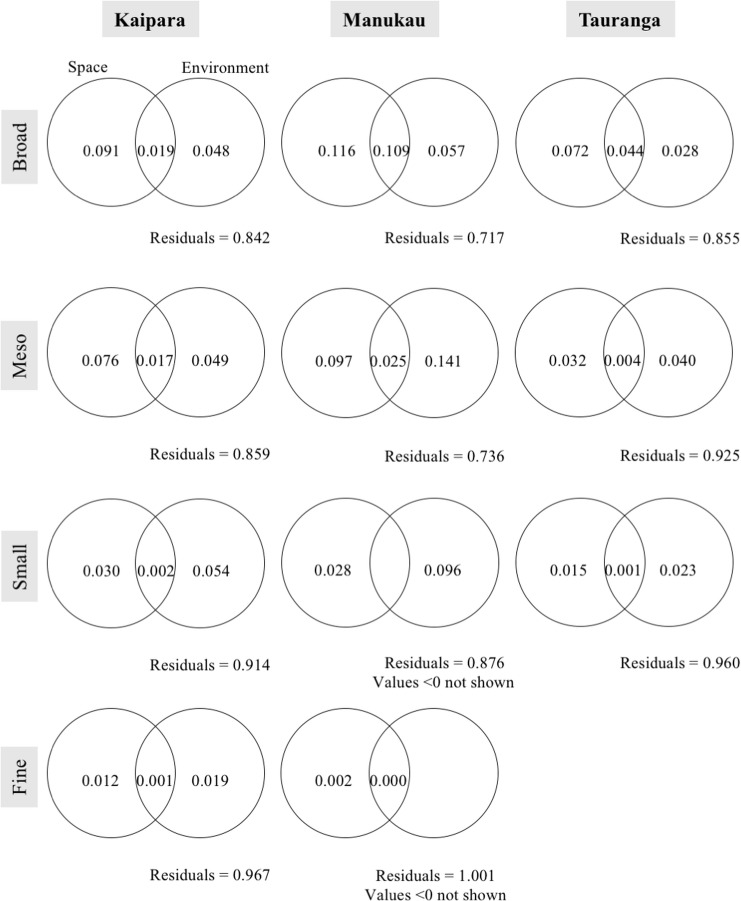
Partitioning the variation in an environmental component and a spatial component. No results are shown for the fine-scale MEM-subset for Tauranga, since none of the abiotic variables were linked to that scale. Numbers indicate values for adjusted R^2^.

Partitioning of the variance showed that for the broad-scale sub-models little variation of the spatial components could be captured by the abiotic variables (Fig 4). This lack of variation explained by abiotic variables was generally more pronounced for the other spatial sub-models. Also, part of the variation was captured by spatially structured abiotic factors (overlapping parts of the circles in Fig 4). All fractions were significant (*p* < 0.05) with the exception of the Manukau fine-scale partitioning.

### Characteristic Species

48 different species (Table 3), encompassing bivalves (*n* = 6), polychaetes (*n* = 28), crustaceans (*n* = 10) and gastropods (*n* = 4), represented the most characteristic species (see Materials and Methods for their definition). Most of these, such as the bivalve *M*. *liliana* or the crustacean *Paracalliope novizealandiae*, were associated with more than one spatial scale. A minority of these characteristic species, such as the polychaete *Travisia olens*, were captured best by a single scale model (Table 3), suggesting they are specialist in their resource requirements. Interestingly, most crustaceans and gastropods were characteristic species only at single sites. In contrast, bivalves were characteristic at more than one site. Polychaetes were either characteristic in all three sites or limited to just a single site (Table 3).

**Table 3:** Characteristic macrozoobenthic species associated with broad, meso, small and fine scale MEM models.

	Kaipara			Tauranga			Manukau		
Broad	AusMod	AusStu	Euchon	AntAur	AonTri	AusStu	AntAur	AonTri	Aricid
	HetFil	MacLil	MacSte	Cerato	HetFil	Lumbri	AusStu	BocSyr	BumCir
	NucHar	OrbPap	OwePet	Lysian	MacSte	NucHar	CosCon	Dexami	HetFil
	PapAus	ParNou	Phoron	PerVal	Phoxoc	ScoCyl	MacLil	MacSte	Nemert
	PriAuc	PseFat	SolSil	ScoLel	ZeaSub	NotSca	NucHar	OwePet	
	TraOle	TroDen	WaiBre				PriAuc		
Meso	AonTri	AusMod	AusStu	AntAur	Cerato	HetFil	AntAur	AonTri	Aricid
	Euchon	Hesion	HetFil	LasPar	Lumbri	Lysian	AusStu	CosCon	HetFil
	MacLil	MagDak	Nemert	MacLil	NucHar	PerVal	MacLil	MacSte	MagDak
	NucHar	OwePet	Phoron	Phoxoc	PriAuc	ScoBen	Nemert	NotSca	NucHar
	PriAuc	SolSil		ScoCyl	Scolel	ZeaLut	ParNou	PriAuc	SolSil
				ZeaSub			TroDen		
Small	AonTri	AusMod	BumCir	AntAur	AonTri	Cerato	AntAur	AonTri	AusStu
	Cerato	Euchon	LasPar	ColLem	DilSub	LasPar	BocSyr	CosCon	HetFil
	MacLil	MacSte	NicAes	Lumbri	Lysian	MacLil	MacLil	MagDak	NicAes
	OrbPap	PapAus	ParNou	PerVal	Phoxoc	PriAuc	NotSca	NucHar	ParLyr
	PriAuc	PseThi	ScoBen	ScoCyl	Scolel	ZeaSub	PriAuc		
	SolSil	WaiBre							
Fine	AonTri	AusMod	BocSyr				AntAur	AusStu	BocSyr
	BumCir	ColLem	Euchon				ComGla	HalWhi	HetFil
	MacLil	MacSte	MagDak				MacLil	MacSte	MagDak
	Nemert	NucHar	OwePet				Nemert	NicAes	NucHar
	PapAus	ParNou	Phoron				OwePet	ParLyr	PlaAus
	PseFat	TroDen					PriAuc	PseFat	

Fine scale subset for Tauranga not shown, since none of the environmental variables linked to this scale. **Bivalves**: AusStu = *Austrovenus stutchburyi*, LasPar = *Lasaea parangaensis*, MacLil = *Macomona liliana*, NucHar = *Nucula hartvigiana*, PapAus = *Paphies australis*, SolSil = *Soletellina siliqua*. **Crustaceans**: AusMod = *Austrominius modestus*, ColLem = *Colurostylis lemurum*, Dexami = Dexaminidea, HalWhi = *Halicarcinus whitei*, ParNou = *Paracalliope nouzealandia*, Phoxoc = Phoxocephalidea, WaiBre = *Waitangi brevirostris*. **Polychaetes**: AonTri = *Aonides trifida*, Aricid = Aricidea, BocSyr = *Boccardia syrtis*, BumCir =“Bumpy cirrisyllid”, Cerato = Ceratonereis sp., Euchon = Euchone sp., CosCon = *Cossura consimilis*, Hesion = Hesionidea, HetFil = *Heteromastus filiformis*, Lumbri = Lumbrineridea, Lysian = Lysianassidae, MacSte = *Macroclymenella stewartensis*, MagDak = *Magelona dakini*, Nemert = Nemertean, NicAes = *Nicon aestuariensis*, OrbPap = *Orbinia papillosa*, OwePet = *Owenia petersonae*, ParLyr = *Paradonereis lyra*, PerVal = *Perinereis vallata*, Phoron = Phoronis sp., PlaAus = *Platynereis australis*, PriAuc = *Prionospio aucklandia*, PseFat = Pseudopolydora “fat”, PseThi = Pseudopolydora “thin”, ScoBen = *Scolecolepides benhami*, ScoCyl = *Scoloplos cylindrifer*, Scolel = Scolelepis sp., TraOle = *Travisia olens*, TroDen = *Trochodata dendyi*. **Gastropods**: ComGla = *Cominella glandiformis*, DilSub = *Diloma subrostrata*, NotSca = *Notoacmea scapha*, ZeaLut = *Zeacumantus lutulentus*, ZeaSub = *Zeacumantus subcarinatus*. **Cnidaria**: AntAur = *Anthopleura aureoradiata*.

## Discussion

Addressing the role of scale-dependency in the spatial structuring of communities in relationship to abiotic variables to infer processes has been recognised as critical in ecology (e.g. [36]). This helps to focus studies on the scales that are most relevant to community composition, as well as identifying abiotic variables most associated with these scales. In return this provides information on the mechanisms that likely underpin community diversity and distributions. The inferences drawn from such analysis can help support conservation or environmental management measures due to improved ecological understanding. Realising such research goals requires sampling communities with purposefully cross-scale sampling designs and using models capable of capturing such information. Yet, to our knowledge, our work offers a scarce example of studies to gather response and explanatory data at corresponding scales across such a large range of spatial scales in coastal ecosystems (but see, amongst others, [32, 37, 38, 39]).

Moran’s eigenvector mapping, as applied here to intertidal communities living in sandy sediments, allowed us to define distinct spatial scale ranges by explicitly including spatial autocorrelation in the analysis of multivariate biodiversity data [15,40]. While we found strong support for the links between abiotic variables and communities on scales from 1 km to just 15 m, at spatial scales finer than 15 m other factors seem to be associated with spatial structuring of communities (see below). The variation captured by the various spatial subsets is similar to previous studies (e.g. [24,30]), demonstrating less spatial variation in community composition captured from broader to finer scales. Ecological studies incorporate many sources of variation; therefore the explained spatial variation is likely to remain limited, especially in dynamic systems [24].

The fine-scale subsets explained limited variability, particularly in the Tauranga site. Explanatory abiotic variables linked to fine-scale variation were not measured or the patterns are due to biotic variables (see [24,30]). Other studies (e.g. [29]) discussed the possibility of their sampling design being inadequate to detect fine-scale variability due to lack of replication at fine spatial scales. However, our spatial sampling grid was explicitly designed to capture such patterns, foregoing arguments of a lack of power at finer scales. Alternatively, community-environment relationships might not be apparent at such fine scales simply due to the fact that macrozoobenthic communities respond to abiotic variables at scales larger than 15 m. Partitioning of variation indicated that our set of abiotic variables had a significant, yet relatively minor (up to 14%), contribution to small-scale community structure. This suggests that biotic variables are a likely explanation for small-scale community structure. Prior to the development of multivariate spatial modelling approaches, such quantitative insight into the organisation of community structure would have been difficult to obtain.

It is becoming increasingly clear that purely environmental based species distribution models are too simple to capture complex community responses to habitat change (e.g. [16,41]). In addition, experimental field studies indicate a profound role for complex interactions across scales governing community dynamics [19,42,43]. The MEM-framework offers a quantitative approach towards understanding scale-dependent community interactions associated with abiotic variables (also see, e.g. [44]). This is essential if the role of biodiversity in affecting response to changing environmental conditions (e.g. climate change) is to be fully realised. Indeed, the merit of our study is providing an ecological case study on the impact of including spatial structure and abiotic variables to better differentiate biogeographical patterns of species communities across scales. Yet, an open, non trivial, question remains how such MEM-derived spatial templates can be incorporated in predictive species distribution models to accommodate cross-scale variation in biodiversity-environment links.

In conclusion, our study emphasises the lack of one right scale to study spatial variation in species communities. As highlighted by the MEM-framework, different species and different habitat features are linked to various spatial scales. This emphasises the importance of assessing the relationship between environmental variables and the abundance and distribution of biological assemblages across a range of different scales.

## Supporting Information

S1 Appendix (PDF)
