# Supplementary material for: Cross-Scale Variation in Biodiversity-Environment Links Illustrated by Coastal Sandflat Communities
Source: PLoS One. 2015 Nov 10;10(11):e0142411. doi: 10.1371/journal.pone.0142411 (PMC4640831; doi:10.1371/journal.pone.0142411)
Supplement: S1 Appendix — (PDF) [file pone.0142411.s001.pdf]

Harbour	tsilt	tveryfine	Fine	Medium	tcoarse	tseagrass	tshellhash	tsand	tloi	tchla
k	0	0,17653297	64,0395584	32,8501053	0,01618341	0	0,22551341	1,34528292	-0,1895767	3,19891662
k	0	0,17653297	64,0395584	32,8501053	0,01618341	0	0,22551341	1,34528292	-0,1895767	3,19891662
k	0	0,17653297	64,0395584	32,8501053	0,01618341	0	0,22551341	1,34528292	-0,1895767	3,19891662
k	0,11212442	0,21257114	60,7035484	32,4485817	0,10720735	0	0,30469265	0,33806525	-0,0477483	2,9070071
k	0	0,18410472	63,9005623	32,7036552	0,02108473	0	0,33806525	1,23273107	-0,1677338	2,82106663
k	0,20977947	0,18633707	64,6457977	31,2189713	0,01406602	0	0,28675655	1,28403977	-0,0548895	3,58288804
k	0	0,17308733	62,8357201	34,123745	0,02728213	0	0,20135792	1,36943841	-0,2002307	2,06381045
k	0	0,3334136	66,4466553	22,8427448	0	0	0,20135792	1,36943841	-0,2520273	1,58581355
k	0	0,33563002	66,5189972	22,6329346	0	0	0	1,57079633	-0,0369994	1,61221737
k	0	0,32180173	66,4471893	23,5497379	0	0	0,10016742	1,47062891	-0,2707847	1,45699468
k	0	0,35680567	66,8711929	20,9164772	0,01117073	0	0	1,57079633	-0,1634125	1,70948591
k	0	0,34281827	67,0214386	40,2018776	0,05622909	0	0,10016742	1,47062891	-0,1052736	1,78402601
k	0	0,28741067	71,716713	20,2477627	0	0	0	1,57079633	-0,3026064	1,95624037
k	0	0,31997685	68,0016098	22,1045647	0	0	0	1,57079633	-0,291242	1,94750498
k	0	0,31879187	67,2174377	22,9473763	0,01095604	0	0	1,57079633	-0,1401767	1,74752612
k	0	0,3091183	66,0773697	24,6613712	0,0079694	0	0	1,57079633	-0,2467871	1,71984204
k	0	0,2176726	51,3456459	41,9704704	0,14261358	0	0	1,57079633	-0,119049	1,37037627
k	0	0,29858949	65,7015686	25,6357594	0,00944895	0	0,10016742	1,47062891	-0,1611943	2,00117244
k	0	0,31258092	66,6272278	23,9161911	0	0	0,20135792	1,36943841	-0,1277702	1,51284338
k	0	0,26682663	70,2146225	22,8260002	0,00842922	0	0	1,57079633	-0,093481	1,69473594
k	0	0,26682663	70,2146225	22,8260002	0,00842922	0	0	1,57079633	-0,093481	1,69473594
k	0	0,26682663	70,2146225	22,8260002	0,00842922	0	0	1,57079633	-0,093481	1,69473594
k	0,10392515	0,19878159	59,5802879	34,2067223	0,11145959	0	0,10016742	1,47062891	-0,1765106	2,37584571
k	0,21232261	0,14126895	62,2955284	31,2670708	0,01191498	0	0,22551341	1,34528292	-0,017555	3,43318794
k	0,30836539	0,2133696	55,9542351	30,2352028	0,03395605	1,39671332	0	0,17408301	0,18923343	3,7052556
k	0	0,30120752	71,8814087	19,3170719	0	0	0,10016742	1,47062891	-0,1300119	1,66849183
k	0	0,34663359	68,5461121	19,9120007	0	0	0,20135792	1,36943841	-0,0987455	2,23283888
k	0	0,34663359	68,5461121	19,9120007	0	0	0,20135792	1,36943841	-0,0987455	2,23283888

k	0	0,2772908	64,3073578	28,1826859	0,01264747	0	0	1,57079633	-0,2151876	1,78984066
k	0,18824718	0,38409008	69,7228241	12,7339535	0	0	0	1,57079633	-0,07157	1,52210768
k	0,18824718	0,38409008	69,7228241	12,7339535	0	0	0	1,57079633	-0,07157	1,52210768
k	0,18824718	0,38409008	69,7228241	12,7339535	0	0	0	1,57079633	-0,07157	1,52210768
k	0,14971358	0,36678862	66,4344559	18,4800301	0	0	0	1,57079633	-0,0804361	1,99798834
k	0,1402392	0,29267187	62,4987946	26,0412483	0,10916147	0	0,17408301	1,39671332	-0,0917615	1,26149689
k	0	0,28371136	64,7027359	27,4473152	0,01199862	0	0	1,57079633	-0,2152002	1,97394428
k	0,17694003	0,28378224	62,046257	27,0020275	0,0133533	0	0	1,57079633	-0,1576078	1,71506334
k	0,10907376	0,26098284	62,4502831	28,6791992	0,10154295	0,41151685	0,33806525	1,02439576	-0,0575105	2,0537056
k	0	0,29102494	64,6020584	27,1490192	0,01256974	0,79539883	0,35374161	0,65388706	-0,0698741	2,27684002
k	0	0,33378689	62,0528069	27,1944427	0,01380334	1,21705472	0,22551341	0,26776333	-0,0273775	2,22727767
k	0	0,30835473	65,7012024	25,0791492	0,00945953	0	0,20135792	1,36943841	-0,1591468	1,69968129
k	0	0,30835473	65,7012024	25,0791492	0,00945953	0	0,20135792	1,36943841	-0,1591468	1,69968129
k	0	0,37507505	63,2504425	23,3206787	0,00906048	0,33806525	0	1,23273107	-0,0752765	2,22109442
k	0	0,14430873	62,3565941	31,8976192	0,0156317	0	0,33806525	1,23273107	-0,1039158	3,100095
k	0	0,19998754	60,5779724	35,319088	0,03956737	0	0,33806525	1,23273107	-0,1106308	1,78444673
k	0,25279363	0,34595046	56,6715393	25,5478439	0,01638914	1,14580754	0	0,42498878	0,1269732	3,03564135
k	0,10178966	0,19266775	63,7536125	30,5775261	0,09879445	0,45102681	0,28675655	1,02439576	-0,0029353	2,32619934
k	0,10907376	0,26098284	62,4502831	28,6791992	0,10154295	0,41151685	0,33806525	1,02439576	-0,0575105	2,0537056
k	0,10907376	0,26098284	62,4502831	28,6791992	0,10154295	0,41151685	0,33806525	1,02439576	-0,0575105	2,0537056
k	0	0,34663359	68,5461121	19,9120007	0	0	0,20135792	1,36943841	-0,0987455	2,23283888
k	0,1780118	0,31374182	53,5204468	32,6880836	0,10656662	0	0,10016742	1,47062891	-0,0701159	2,46051186
k	0	0,20600128	50,5128517	42,9826851	0,15292732	0	0,17408301	1,39671332	-0,170341	1,51765573
k	0	0,30911925	60,4767952	30,112772	0,03943965	0	0,10016742	1,47062891	-0,1929144	2,01084368
k	0	0,31632893	60,7587318	29,528347	0,0189384	0	0	1,57079633	-0,1617371	0,63708778
k	0	0,2534769	63,1952477	30,459795	0,02373765	0	0	1,57079633	-0,2331733	1,66132168
k	0	0,2534769	63,1952477	30,459795	0,02373765	0	0	1,57079633	-0,2331733	1,66132168
k	0	0,2534769	63,1952477	30,459795	0,02373765	0	0	1,57079633	-0,2331733	1,66132168
k	0,19239012	0,25947636	59,9532166	29,7193928	0,02973517	1,303033	0	0,26776333	-0,1015854	1,97764196

k	0	0,22375758	52,3607178	40,8178825	0,13819492	0	0,17408301	1,39671332	-0,248903	2,06001583
k	0	0,23484166	53,0571671	39,8120766	0,13138684	0	0,17408301	1,39671332	-0,3252314	2,29115517
k	0	0,16769235	51,6332779	43,996048	0,12622635	0	0,10016742	1,47062891	-0,1794482	2,39429765
k	0	0,23826671	52,9878807	43,7466621	0,12446875	0	0,26776333	1,303033	-0,2297606	1,93657921
k	0	0,16604247	51,9803085	39,5034637	0,13967197	0	0,22551341	1,34528292	-0,2760374	1,70091699
k	0	0,25483836	60,0598679	33,1492577	0,06607739	0	0	1,57079633	-0,267507	1,83666023
k	0	0,23732453	60,1449051	34,0488091	0,05283945	0,39769942	0	1,17309691	-0,1904518	1,31016078
k	0,20948583	0,33986364	62,4217529	22,1323719	0,00920611	0,20135792	0	1,36943841	0,15702564	2,25887181
k	0,19456172	0,3506616	63,4744949	20,9870548	0	0	0	1,57079633	-0,1963204	2,27820133
k	0	0,2387179	69,3339996	25,0638084	0,01048733	0	0	1,57079633	-0,1399806	2,33839558
k	0	0,26811184	62,480896	30,3757687	0,0354389	0	0,10016742	1,47062891	-0,2092555	1,3553231
k	0	0,24823706	61,8075714	31,9823761	0,04165637	0	0,26776333	1,303033	-0,2184198	2,27820133
k	0	0,27452173	63,1315346	29,412077	0,03280726	0	0,10016742	1,47062891	-0,1874465	1,8517239
k	0	0,27452173	63,1315346	29,412077	0,03280726	0	0,10016742	1,47062891	-0,1874465	1,8517239
k	0	0,27452173	63,1315346	29,412077	0,03280726	0	0,10016742	1,47062891	-0,1874465	1,8517239
k	0	0,2676273	63,0900993	29,8171539	0,03157897	0	0	1,57079633	-0,1999409	2,48219079
k	0	0,28473877	64,3290176	27,7372437	0,02070848	0	0	1,57079633	-0,1250695	2,51537326
k	0	0,24588251	69,7155685	24,3510952	0,00915163	0	0	1,57079633	-0,233245	2,99978875
k	0	0,21049849	73,261528	22,3725739	0	0	0	1,57079633	-0,1262083	2,7745879
k	0	0,28279416	64,5500946	27,6483173	0,01235305	0	0	1,57079633	-0,2092414	0,67270904
k	0	0,25716478	69,4072037	24,1135254	0,01020312	0	0	1,57079633	-0,0969991	2,56620669
k	0	0,21338715	61,5084839	33,8956528	0,03334519	0	0	1,57079633	0,01963002	3,71170518
k	0,22289184	0,2193201	62,6086655	27,7496986	0,01476197	0	0	1,57079633	0,01524976	3,41829337
k	0,39602612	0,30956266	46,8599586	28,2444096	0,08579366	1,15927948	0,17408301	0,36886298	0,17921729	0,99199494
k	0	0,25483836	60,0598679	33,1492577	0,06607739	0	0	1,57079633	-0,267507	1,83666023
k	0	0,25483836	60,0598679	33,1492577	0,06607739	0	0	1,57079633	-0,267507	1,83666023
k	0	0,24078763	53,4648666	39,2252922	0,12775042	0	0,10016742	1,47062891	-0,2197532	2,19942472
k	0	0,20618088	55,2821007	39,5019264	0,10140671	0	0,10016742	1,47062891	-0,2511166	2,29066812
k	0,27363255	0,31572129	61,455883	21,6004982	0	0,65388706	0	0,91690926	0,0206909	3,06200062

k	0,13689552	0,35758155	65,0949554	20,791996	0	0,10016742	0	1,47062891	0,0407658	2,40203091
k	0	0,29650616	65,7659378	25,6907063	0,00800321	0,67449093	0	0,8963054	-0,1580564	1,92269675
k	0,23572164	0,31379206	62,2672653	22,750845	0	0,67449093	0	0,8963054	-0,0100025	2,13016007
k	0,19497864	0,25543386	65,7921371	24,0701561	0	0,26776333	0	1,303033	0,04787125	2,11088802
k	0,39602612	0,30956266	46,8599586	28,2444096	0,08579366	1,15927948	0,17408301	0,36886298	0,17921729	0,99199494
k	0,39602612	0,30956266	46,8599586	28,2444096	0,08579366	1,15927948	0,17408301	0,36886298	0,17921729	0,99199494
k	0	0,25084565	56,5638084	36,2958374	0,09910049	0,60126422	0,10016742	0,95885661	-0,0368636	4,20412255
k	0,30575552	0,29756099	54,3363991	27,6420135	0,06042451	1,21705472	0,10016742	0,33806525	0,22573559	1,37749515
k	0	0,25692842	54,5052376	37,7290382	0,11463997	0	0,20135792	1,36943841	-0,3394319	1,90335927
k	0	0,25475989	61,7214355	31,7851353	0,03774104	1,0131975	0	0,55759883	-0,1449029	1,86190758
k	0	0,22160465	57,0957184	37,3136864	0,08726558	0	0,33806525	1,23273107	-0,2120368	1,79012903
k	0	0,20732684	55,6729393	39,1833801	0,09535336	0	0,10016742	1,47062891	-0,2470635	1,89435984
k	0	0,22093964	57,6389885	36,9354782	0,07901342	0	0	1,57079633	-0,2577247	1,64512316
k	0	0,19229683	65,4746017	30,8635769	0,00967899	0	0	1,57079633	-0,1108806	3,41211608
k	0,08561995	0,24751481	62,2734375	29,9266453	0,10344927	0,64350111	0,35374161	0,8054035	-0,0657042	3,48532516
k	0	0,20201248	60,5907249	35,2186508	0,04062299	0	0	1,57079633	-0,1363241	3,5759676
k	0,190879	0,29355443	62,3258972	25,6936836	0,01065572	0,87589139	0,17408301	0,66421524	-0,1191864	2,74172918
k	0,09240588	0,31866546	64,0578308	23,1929512	0,14482048	0	0,10016742	1,47062891	-0,0578305	1,92007393
k	0,18468913	0,28845198	62,3797836	26,1458092	0,01159681	1,20193334	0,22551341	0,28675655	0,01182588	1,62842097
k	0,08717875	0,28205258	63,4855461	27,9917355	0,01341644	0,8963054	0,28675655	0,59050002	0,05215372	1,1743897
k	0	0,28837428	65,0087967	26,8915138	0,0108127	0	0,17408301	1,39671332	-0,1090299	1,9351672
k	0	0,30835473	65,7012024	25,0791492	0,00945953	0	0,20135792	1,36943841	-0,1591468	1,69968129
k	0,19497864	0,25543386	65,7921371	24,0701561	0	0,26776333	0	1,303033	0,04787125	2,11088802
k	0,19497864	0,25543386	65,7921371	24,0701561	0	0,26776333	0	1,303033	0,04787125	2,11088802
k	0,2445072	0,32823088	61,2203331	22,5188713	0,00918816	0,17408301	0	1,39671332	0,13039498	2,89100306
k	0	0,23416775	59,8589745	34,3890305	0,06070366	0,28675655	0	1,28403977	-0,1253703	1,72770474
k	0	0,23416775	59,8589745	34,3890305	0,06070366	0,28675655	0	1,28403977	-0,1253703	1,72770474
k	0	0,23416775	59,8589745	34,3890305	0,06070366	0,28675655	0	1,28403977	-0,1253703	1,72770474
k	0	0,27579125	56,9363785	35,0120163	0,0798608	0,63305184	0,17408301	0,90658109	-0,0919031	1,71359033

k	0	0,24416499	54,7255707	38,3497925	0,10413588	0,63305184	0,10016742	0,92729522	-0,2698155	1,52369234
k	0,14627331	0,27286923	62,4338531	28,0803165	0,03141711	0,64350111	0,51197269	0,6847192	-0,1821943	1,36257238
k	0	0,1809795	63,6622543	33,0549011	0,02075938	0	0	1,57079633	-0,1258723	2,25494678
k	0	0,22141088	52,0194969	35,7862129	0,06286137	0,52359878	0,39769942	0,88607712	-0,1103187	2,44110496
k	0	0,22349052	67,9851761	27,1026325	0	0,26776333	0,20135792	1,23273107	-0,2269788	2,52549551
k	0,10060213	0,20977709	59,4826813	40,0467415	0,14550963	0	0,30469265	0,33806525	-0,1261964	2,02039642
k	0,09589968	0,27923837	58,1097488	31,8981705	0,12189046	0	0,26776333	1,17309691	-0,2142939	3,51108865
k	0,09589968	0,27923837	58,1097488	31,8981705	0,12189046	0	0,26776333	1,17309691	-0,2142939	3,51108865
k	0,09589968	0,27923837	58,1097488	31,8981705	0,12189046	0	0,26776333	1,17309691	-0,2142939	3,51108865
k	0	0,22353542	61,6141243	33,356411	0,03396739	0	0,26776333	1,303033	-0,1998184	2,14295398
k	0	0,21760105	61,589138	33,6306076	0,03457523	0,10016742	0,28675655	1,26610367	-0,2871083	1,87553563
k	0,19791479	0,28589791	60,4050674	27,6872387	0,02968095	1,21705472	0	0,35374161	0,07512634	1,78128145
k	0,24805853	0,33470244	55,8173447	27,124012	0,04901677	1,57079633	0	0	0,00553963	1,61992094
k	0,26418744	0,33195249	55,8035278	26,6362095	0,03482643	1,47062891	0	0,10016742	0,19435607	2,55209592
k	0,09461475	0,23747562	60,7759323	31,7046185	0,10484776	1,57079633	0	0	-0,1085035	2,25663426
k	0,2242854	0,34479336	57,1777802	26,4202156	0,01756422	1,21705472	0	0,35374161	-0,009262	1,80645566
k	0,20377495	0,29357058	60,3617401	27,0969677	0,02691366	1,47062891	0	0,10016742	0,00624058	2,26642758
k	0,20715331	0,30426471	61,2358627	25,4803028	0,0279493	1,39671332	0,17408301	0	0,02217911	1,74971462
k	0,20715331	0,30426471	61,2358627	25,4803028	0,0279493	1,39671332	0,17408301	0	0,02217911	1,74971462
k	0,20715331	0,30426471	61,2358627	25,4803028	0,0279493	1,39671332	0,17408301	0	0,02217911	1,74971462
k	0,2223106	0,24924964	64,6125717	24,4328995	0,00909133	0,26776333	0	1,303033	-0,1184799	2,30305252
k	0	0,22195492	60,8020477	34,1195679	0,0482218	0,17408301	0	1,39671332	-0,2201081	2,3676286
k	0,24805876	0,21938484	56,111084	32,7101593	0,06441612	0,8963054	0,17408301	0,64350111	0,15770285	2,65614256
k	0,09873432	0,24302344	60,5809555	32,0222054	0,07976586	1,15927948	0,10016742	0,39769942	-0,0800421	2,31465482
k	0,25859779	0,20619768	61,4580078	27,7934742	0,01309503	0	0,30469265	1,26610367	-0,0426058	2,3966148
k	0,20969456	0,22998498	56,6275063	33,3428955	0,07075471	0,5686755	0,30469265	0,90658109	0,08142533	1,74766937
k	0,21107047	0,24062015	58,510746	31,1931648	0,04775251	1,34528292	0,14189705	0,17408301	0,05824526	2,18088534
k	0,23957151	0,21091162	53,687851	35,4389191	0,09286676	0,81541619	0,17408301	0,72525322	0,1132973	2,5878628
k	0,23957151	0,21091162	53,687851	35,4389191	0,09286676	0,81541619	0,17408301	0,72525322	0,1132973	2,5878628

k	0,23957151	0,21091162	53,687851	35,4389191	0,09286676	0,81541619	0,17408301	0,72525322	0,1132973	2,5878628
k	0,22426379	0,24554887	57,8308868	30,9837036	0,05752285	0,36886298	0,30469265	1,08259106	-0,1398248	2,51807024
k	0,26956993	0,18636871	58,4844933	30,8845711	0,03243438	0	0,17408301	1,39671332	0,0275933	3,4317818
k	0,21030776	0,2050239	62,7416992	28,7284451	0,0163809	0	0,33806525	1,23273107	-0,1490266	3,08813476
k	0,19129374	0,20130916	63,5283813	28,8336563	0,01580002	0	0,20135792	1,36943841	-0,1204543	2,40545988
k	0	0,17288081	62,7474022	34,2161827	0,0278052	0	0,17408301	1,39671332	-0,0797925	3,46202535
k	0	0,22321054	61,9347954	33,0076447	0,0396919	0	0,26776333	1,303033	-0,2505608	2,54989697
k	0,09607803	0,22228039	60,7030182	32,1105194	0,11886206	0	0,28675655	1,28403977	-0,2147541	2,96420454
k	0	0,20885754	60,8914337	34,6317978	0,04216472	0	0,17408301	1,39671332	-0,1966207	2,39056526
k	0	0,20885754	60,8914337	34,6317978	0,04216472	0	0,17408301	1,39671332	-0,1966207	2,39056526
k	0	0,20885754	60,8914337	34,6317978	0,04216472	0	0,17408301	1,39671332	-0,1966207	2,39056526
k	0	0,17732743	63,6148605	33,2230492	0,0224545	0	0,35374161	1,21705472	-0,0921551	2,72223247
k	0,21272208	0,14676071	62,6996155	30,6893082	0,01240919	0,14189705	0,20135792	1,32332926	-0,1554575	2,92447306
k	0,20853653	0,14879575	63,0826874	30,4169827	0,01285687	0	0,30469265	1,26610367	-0,0534828	3,91154024
k	0,28084316	0,18101816	58,3948517	30,6299324	0,02280614	1,28403977	0,20135792	0,20135792	0,0245682	1,06816487
k	0,25951527	0,21786431	62,4549599	26,281683	0,00807412	0,88607712	0,10016742	0,67449093	0,07703876	2,1651614
k	0,25951527	0,21786431	62,4549599	26,281683	0,00807412	0,88607712	0,10016742	0,67449093	0,07703876	2,1651614
k	0,25951527	0,21786431	62,4549599	26,281683	0,00807412	0,88607712	0,10016742	0,67449093	0,07703876	2,1651614
k	0,1948189	0,20749241	62,9069786	29,0819092	0,0139901	0,69490494	0,33806525	0,76539283	-0,0048699	1,00392751
k	0,22622291	0,19290698	61,2775497	29,9810715	0,01871659	0,96953211	0,20135792	0,55759883	-0,0586236	2,8690178
k	0,3442586	0,25785804	56,971035	25,0874043	0,02191111	1,04719755	0,26776333	0,43814903	0,22662843	1,34277606
k	0,19701483	0,32420163	63,3726616	22,6482716	0	0,51197269	0,10016742	1,04719755	-0,1406047	2,90189979
k	0,27332641	0,21238503	61,6385307	26,6237507	0,00886352	1,57079633	0	0	0,19569171	2,50011301
k	0,26598892	0,18992981	60,1375465	29,3713894	0,0131061	1,05882364	0,30469265	0,39769942	0,18419514	3,73789397
k	0	0,19842522	60,2634048	35,6520805	0,04458629	0	0	1,57079633	-0,1735952	2,29041695
k	0	0,20079512	60,6785393	35,1986694	0,03806455	0	0,10016742	1,47062891	-0,0877582	2,22384842
k	0	0,20836232	61,0657845	34,5248146	0,0361163	0	0	1,57079633	-0,205012	1,84522279
k	0	0,21000081	60,2624512	35,1538162	0,0488197	0	0	1,57079633	-0,1548949	3,02315755
k	0	0,15927631	62,1859665	35,2320938	0,02577571	0	0,10016742	1,47062891	-0,0675881	3,69225871

k	0	0,17632792	64,1985779	32,6985207	0,01607396	0	0	1,57079633	-0,0535204	3,93485086
k	0	0,16617836	62,3120003	34,8714142	0,02835587	0	0	1,57079633	-0,1271458	3,07742642
k	0	0,16617836	62,3120003	34,8714142	0,02835587	0	0	1,57079633	-0,1271458	3,07742642
k	0	0,16617836	62,3120003	34,8714142	0,02835587	0	0	1,57079633	-0,1271458	3,07742642
k	0	0,16502873	62,1128693	35,1157112	0,02694895	0	0	1,57079633	-0,1473272	2,72578738
k	0	0,19813168	59,2876205	36,5071754	0,05753713	0	0,10016742	1,47062891	-0,2776941	2,45092568
k	0	0,16868983	61,7537956	35,3025169	0,03535592	0	0	1,57079633	-0,1093637	3,1226401
k	0	0,16180536	60,9496384	36,2760086	0,0423241	0	0	1,57079633	-0,2750081	1,30286838
k	0	0,20705179	58,6029205	36,6358833	0,07321339	0,32175055	0,22551341	1,17309691	-0,2415571	2,15638954
k	0	0,16405406	60,9492683	36,2078171	0,04191681	0	0,10016742	1,47062891	-0,2053287	2,60304104
k	0	0,18527008	63,2952309	33,2008553	0,03324902	0	0,17408301	1,39671332	-0,1636086	2,56469893
k	0	0,23447331	62,3776398	32,1080742	0,03414298	0,10016742	0,17408301	1,36943841	-0,2547642	1,2431691
k	0	0,23447331	62,3776398	32,1080742	0,03414298	0,10016742	0,14189705	1,39671332	-0,2547642	1,2431691
k	0	0,23447331	62,3776398	32,1080742	0,03414298	0,10016742	0,14189705	1,39671332	-0,2547642	1,2431691
k	0	0,17491831	61,8364372	34,9536896	0,04259315	0,10016742	0,17408301	1,36943841	-0,2392995	2,08072001
k	0	0,20462805	57,8239746	37,3460732	0,08381236	0,30469265	0,17408301	1,21705472	-0,2393703	2,38445908
k	0	0,21529885	59,8541756	35,2065277	0,06128548	0,36886298	0,22551341	1,1326473	-0,20412	1,85687312
k	0	0,19185253	55,9844666	39,3805504	0,10012636	0,383497	0,24746706	1,10714872	-0,3525475	1,90929195
k	0,10438877	0,23332539	61,1910019	31,1885796	0,10924362	0,30469265	0	1,26610367	0,09284417	2,52970114
k	0	0,19943545	57,0714989	38,2255402	0,08831715	0,39769942	0,20135792	1,11976951	-0,1261262	2,0925654
k	0	0,18759286	56,6909828	39,1523399	0,08247417	0,39769942	0,10016742	0,41151685	-0,1880296	2,49837861
k	0,24501009	0,18333254	53,1323547	37,0035934	0,08129333	0,39769942	0,14189705	1,14580754	-0,1280601	3,02680391
k	0,24501009	0,18333254	53,1323547	37,0035934	0,08129333	0,383497	0,14189705	1,15927948	-0,1280601	3,02680391
k	0,24501009	0,18333254	53,1323547	37,0035934	0,08129333	0,33806525	0,14189705	1,20193334	-0,1280601	3,02680391
k	0	0,22425309	54,3794479	39,3498917	0,11538426	0,35374161	0,10016742	1,20193334	0,00576101	3,46804362
k	0,18582422	0,16513617	43,8038216	45,4301949	0,21735022	0,28675655	0,14189705	1,24904577	-0,0998964	2,22809492
k	0,20319242	0,17381204	58,9637222	33,8152237	0,04048908	0,26776333	0,14189705	1,26610367	-0,1294515	2,56862188
k	0	0,1821247	55,7650871	40,1901741	0,08753669	0	0	1,57079633	-0,0891908	2,60365866
k	0	0,18844745	57,3074722	38,6101265	0,07576878	0,22551341	0,17408301	1,28403977	-0,1718839	1,70807488

k	0	0,16381573	53,7309151	42,5790291	0,10168465	0	0,10016742	1,47062891	-0,2140866	3,17267151
k	0	0,18626661	52,2955208	42,9062386	0,11725818	0	0,10016742	1,47062891	-0,0939905	2,21017993
k	0	0,17106261	54,3721542	41,8050461	0,09632514	0	0,10016742	1,47062891	-0,2019985	2,01464844
k	0	0,14985935	51,557991	44,7240105	0,12232843	0	0	1,57079633	-0,1824822	2,77212502
k	0	0,14985935	51,557991	44,7240105	0,12232843	0	0	1,57079633	-0,1824822	2,77212502
k	0	0,14985935	51,557991	44,7240105	0,12232843	0	0	1,57079633	-0,1824822	2,77212502
k	0	0,18674041	56,9214134	39,0349426	0,07732968	0	0	1,57079633	-0,2301004	1,94633531
k	0	0,20077938	58,5503578	37,0548668	0,06465308	0	0,10016742	1,47062891	-0,2743888	2,54178443
k	0	0,17292318	55,7697105	40,4891739	0,08846459	0,35374161	0,10016742	1,20193334	-0,1363033	2,13400638
k	0	0,16808103	55,8952675	40,5715981	0,08580918	0	0	1,57079633	-0,0531318	2,66928671
k	0	0,23569171	56,5896835	37,2418785	0,08468742	0	0	1,57079633	-0,033701	1,88071112
k	0	0,19631302	57,5717239	38,0135994	0,07818527	0	0,17408301	1,39671332	-0,1840643	2,90025417
k	0	0,16313129	53,4910965	42,6261253	0,11181772	0	0	1,57079633	-0,1077679	2,01786928
k	0	0,17380235	56,1436539	40,1741829	0,08326661	0	0,10016742	1,47062891	-0,0671038	2,99119324
k	0	0,17380235	56,1436539	40,1741829	0,08326661	0	0,10016742	1,47062891	-0,0671038	2,99119324
k	0	0,17380235	56,1436539	40,1741829	0,08326661	0	0,10016742	1,47062891	-0,0671038	2,99119324
k	0	0,1794385	56,8512154	39,3109589	0,08086061	0	0	1,57079633	-0,1668032	3,88511052
k	0	0,17487329	56,549942	39,7391205	0,08279383	0	0	1,57079633	-0,0859443	1,75871064
k	0	0,15813597	54,2134972	42,3368721	0,0986337	0	0	1,57079633	-0,0938397	2,78186894
k	0	0,23145504	60,7984161	33,6713257	0,05180115	0	0,10016742	1,47062891	-0,3740815	1,97764196
k	0	0,17906098	57,1214294	39,1105728	0,07726753	0	0,10016742	1,47062891	-0,1277838	3,5479322
k	0	0,16947916	54,8909454	41,3253479	0,09704326	0	0	1,57079633	-0,1043522	3,43907171
k	0	0,17190298	54,6578293	41,3162308	0,10506794	0	0	1,57079633	0,01793759	2,60573676
k	0	0,1663161	54,6138649	41,6415062	0,10036482	0	0	1,57079633	-0,0307182	1,78402601
k	0	0,1663161	54,6138649	41,6415062	0,10036482	0	0	1,57079633	-0,0307182	1,78402601
k	0	0,1663161	54,6138649	41,6415062	0,10036482	0	0	1,57079633	-0,0307182	1,78402601
k	0	0,20151947	58,8670692	36,6888237	0,06621279	0	0,17408301	1,39671332	-0,0798034	2,5249904
k	0	0,2142985	60,4251366	34,8297081	0,04720227	0,20135792	0,28675655	1,21705472	-0,2153165	2,80490757
k	0	0,16495859	60,9426422	36,1758118	0,04302548	0	0	1,57079633	-0,1971223	3,34484475

k	0	0,17587887	63,1789131	33,6761818	0,02887077	0	0	1,57079633	-0,1205217	2,85727202
k	0	0,20202203	59,0613098	36,5171356	0,0629292	0,17408301	0	1,39671332	-0,305434	1,13394466
k	0	0,18768625	58,4031372	37,7160034	0,06324143	0,10016742	0,20135792	1,34528292	-0,178103	2,16365934
k	0	0,16471586	62,119873	35,0795631	0,03345486	0	0,10016742	1,47062891	-0,1242661	2,35917157
k	0	0,17364684	61,9159622	34,9401169	0,03985814	0	0,30469265	1,26610367	-0,2189854	2,82625809
k	0	0,17364684	61,9159622	34,9401169	0,03985814	0	0,30469265	1,26610367	-0,2189854	2,82625809
k	0	0,17364684	61,9159622	34,9401169	0,03985814	0	0,30469265	1,26610367	-0,2189854	2,82625809
k	0	0,16394185	60,912117	36,2302895	0,04404752	0	0,10016742	1,47062891	-0,1765232	1,71506334
k	0	0,1890379	64,2656708	32,1541328	0,02214295	0,17408301	0	1,39671332	-0,2751774	2,23540094
k	0	0,16407932	61,1622391	35,9877243	0,04266362	0	0	1,57079633	-0,186851	2,20592424
k	0	0,22512235	62,1318169	32,7851028	0,03164545	0	0	1,57079633	-0,1042058	1,78402601
k	0	0,21765379	60,9194221	34,1758957	0,04918323	0	0,17408301	1,39671332	-0,2511619	1,17856459
k	0	0,18442568	63,2976036	33,2271233	0,03352866	0	0,10016742	1,47062891	-0,1312102	1,1861929
k	0	0,17279115	62,1598282	34,7329979	0,03888048	0,30469265	0,10016742	1,24904577	-0,1988488	1,87164738
k	0	0,18206783	63,1598969	33,4548225	0,03269864	0	0,20135792	1,36943841	-0,2039111	0,85865483
k	0	0,18206783	63,1598969	33,4548225	0,03269864	0	0,20135792	1,36943841	-0,2039111	0,85865483
k	0	0,18206783	63,1598969	33,4548225	0,03269864	0	0,20135792	1,36943841	-0,2039111	0,85865483
k	0	0,18962644	63,9090881	32,4673271	0,02658489	0	0,20135792	1,36943841	-0,2376798	1,64495359
k	0	0,18426688	63,7397308	32,8270645	0,02757812	0,28675655	0	1,28403977	-0,1116613	2,37238579
k	0	0,21704477	61,626091	33,6083984	0,03580947	0,22551341	0	1,34528292	-0,2046257	1,77166922
k	0,25295379	0,21214822	54,9141464	33,9280891	0,06794527	0,88607712	0	0,6847192	-0,1888986	2,11728896
k	0	0,19115655	64,4026489	31,9406891	0,0216514	0,30469265	0	1,26610367	-0,0951014	1,99460171
k	0	0,19034492	64,1976089	32,1626968	0,02452265	0,10016742	0	1,47062891	-0,1822898	1,94315503
k	0	0,20611488	66,1470261	29,6547356	0,0098617	0,47603382	0	1,09476251	-0,1056829	1,25275932
k	0	0,22999898	61,3826904	33,2392654	0,04252281	0,33806525	0	1,23273107	-0,0567671	2,14123616
k	0	0,22999898	61,3826904	33,2392654	0,04252281	0,33806525	0	1,23273107	-0,0567671	2,14123616
k	0	0,22999898	61,3826904	33,2392654	0,04252281	0,33806525	0	1,23273107	-0,0567671	2,14123616
k	0,21208569	0,21425108	56,1952667	34,4151154	0,06623581	1,39671332	0	0,17408301	0,06653868	1,88990776
k	0,25441635	0,2283981	56,5511246	31,7144032	0,05233727	0,64350111	0,10016742	0,91690926	0,08120484	2,23381889

k	0,21554586	0,22537769	57,6850395	32,5042381	0,0492276	1,26610367	0	0,30469265	-0,0015534	1,86105252
k	0	0,36378874	62,8756561	24,4513779	0,01111328	0	0	1,57079633	-0,0905365	2,36964244
k	0,19788935	0,23005065	58,467041	32,23946	0,04784178	0,96953211	0	0,60126422	-0,197446	1,74971462
k	0	0,22688647	61,3429985	33,3925972	0,04522448	0	0	1,57079633	-0,2046367	1,96562292
k	0	0,24109841	62,6544075	31,5505829	0,03065231	0	0	1,57079633	-0,1484484	2,46051186
k	0,08437677	0,24315838	60,7440453	31,394352	0,11675512	0	0	1,57079633	-0,2706175	2,01464844
k	0,08437677	0,24315838	60,7440453	31,394352	0,11675512	0	0	1,57079633	-0,2706175	2,01464844
k	0,08437677	0,24315838	60,7440453	31,394352	0,11675512	0	0	1,57079633	-0,2706175	2,01464844
k	0,18204669	0,32381785	63,4239349	23,1671104	0,00832356	0	0	1,57079633	0,01670181	1,56848174
k	0,13509478	0,3456175	64,4325027	22,2698441	0,00814564	0	0,17408301	1,39671332	0,01347033	2,36964244
k	0	0,3656949	62,8948021	24,3072205	0,01015842	1,00212082	0	0,5686755	-0,1481912	2,27013816
k	0	0,21353575	59,1028709	35,9993439	0,06383362	0,17408301	0,20135792	1,303033	-0,1434728	1,52993819
k	0,19143017	0,39531651	65,3624878	16,1872768	0	0,98029631	0	0,59050002	0,03520896	3,50956115
k	0,13935274	0,32967138	63,7913742	23,7864571	0,01119845	0	0,20135792	1,36943841	0,03423521	1,92887905
k	0	0,37264511	62,6348915	24,0984936	0,01057044	0	0	1,57079633	-0,0253059	2,31465482
k	0,13002948	0,26971889	60,9210281	30,1430588	0,03932056	0	0	1,57079633	-0,0756418	1,87264797
k	0,13002948	0,26971889	60,9210281	30,1430588	0,03932056	0	0	1,57079633	-0,0756418	1,87264797
k	0,13002948	0,26971889	60,9210281	30,1430588	0,03932056	0	0	1,57079633	-0,0756418	1,87264797
k	0	0,21694352	58,7695351	36,1909676	0,06379442	0	0	1,57079633	-0,2337737	1,70807488
k	0	0,21784073	59,4253235	35,5671196	0,05805778	0	0	1,57079633	-0,3139912	1,70671753
k	0	0,22235237	59,9200363	34,8866234	0,05749688	0	0	1,57079633	-0,2648727	2,08937162
k	0	0,29109372	65,4929428	26,2611828	0,00947168	0	0	1,57079633	-0,1505141	1,15180315
k	0	0,21287098	59,6734619	35,5829353	0,05296151	0,26776333	0	1,303033	-0,2137319	0,95435663
k	0	0,22362603	59,8899269	34,8547516	0,05810756	0	0	1,57079633	-0,3268942	2,05731022
k	0	0,23945094	61,9364319	32,2503204	0,04340971	0	0,17408301	1,39671332	-0,1897414	1,19088008
k	0	0,31293734	72,8126602	17,7098885	0	0,71516746	0	0,85562887	-0,1955342	2,1052836
k	0	0,31293734	72,8126602	17,7098885	0	0,71516746	0	0,85562887	-0,1955342	2,1052836
k	0	0,31293734	72,8126602	17,7098885	0	0,71516746	0	0,85562887	-0,1955342	2,1052836
k	0	0,30266209	65,6693573	25,4377594	0,00936069	0	0	1,57079633	-0,3549168	1,81691061

k	0	0,26641897	62,7188568	30,2076168	0,03768809	0	0,20135792	1,36943841	-0,2447527	1,49862401
k	0	0,28739611	65,7364273	26,2211876	0,00874711	0	0	1,57079633	-0,0881111	1,90335927
k	0	0,27845518	71,1901245	21,2544842	0	0	0	1,57079633	-0,3533391	1,34966409
k	0	0,31281461	67,2520065	23,2660236	0,01082241	0,45102681	0	1,11976951	-0,0253504	2,23217227
k	0	0,31137473	67,1972809	23,4052563	0,01065759	0	0	1,57079633	-0,1982939	2,22013897
k	0	0,30249727	67,236496	23,8887634	0	0,81541619	0	0,75538013	-0,1193674	2,17970259
k	0	0,27412374	63,5998764	29,0157661	0,02373364	0	0	1,57079633	-0,1604796	1,33022237
k	0	0,27412374	63,5998764	29,0157661	0,02373364	0	0	1,57079633	-0,1604796	1,33022237
k	0	0,27412374	63,5998764	29,0157661	0,02373364	0	0	1,57079633	-0,1604796	1,33022237
k	0	0,25563155	69,9904861	23,6089153	0,00834096	0	0	1,57079633	-0,2266585	2,36142031
k	0	0,25670053	69,7965622	23,7486229	0,0093671	0	0	1,57079633	-0,1379077	1,74240891
k	0,2065054	0,31281627	68,7758102	17,5496635	0	0	0,14189705	1,42889927	-0,1947918	3,40440431
k	0	0,36618164	67,7045441	19,4752693	0	0	0	1,57079633	-0,1555839	2,27858014
k	0,18554058	0,29888778	68,6837845	19,2459908	0	0	0,14189705	1,42889927	0,07438666	2,26692489
k	0,20791581	0,39189816	65,0845261	16,0664349	0	0	0,10016742	1,47062891	0,16706905	2,44577529
k	0	0,24043948	61,2111549	32,9493713	0,04111	0	0,20135792	1,36943841	-0,169265	1,55646371
k	0	0,24043948	61,2111549	32,9493713	0,04111	0	0,20135792	1,36943841	-0,169265	1,55646371
k	0	0,24043948	61,2111549	32,9493713	0,04111	0	0,20135792	1,36943841	-0,169265	1,55646371
k	0,13260358	0,26240504	59,8187599	31,0190067	0,08286563	0	0	1,57079633	-0,1306553	2,23766133
k	0	0,37447413	61,0487518	25,4548321	0,03416613	0	0	1,57079633	-0,0014604	1,73507237
k	0	0,35296369	67,4658203	20,5763474	0,00913468	0	0	1,57079633	-0,018087	1,82989534
k	0	0,33010693	67,6797638	21,8062897	0,00836849	0	0	1,57079633	-0,0817847	1,88071112
k	0	0,24894514	62,3174744	31,4986973	0,03368589	0	0	1,57079633	-0,15434	2,14652258
k	0	0,32478449	67,2978058	22,5097218	0,00985105	0	0	1,57079633	-0,0537262	2,10656845
k	0	0,33848256	62,4326134	26,5250797	0,01272119	0	0	1,57079633	-0,1843472	1,66083784
k	0	0,32167149	65,1269913	24,8630924	0,01210732	0	0	1,57079633	-0,2281151	2,30293502
k	0	0,32167149	65,1269913	24,8630924	0,01210732	0	0	1,57079633	-0,2281151	2,30293502
k	0	0,32167149	65,1269913	24,8630924	0,01210732	0	0	1,57079633	-0,2281151	2,30293502
k	0	0,24217667	69,0969315	25,1518784	0	0	0	1,57079633	-0,0974133	2,05172225

k	0	0,24711964	63,5011444	30,4943123	0,01450361	0	0,10016742	1,47062891	-0,1718973	1,87110229
k	0	0,2363383	61,8659058	32,5090294	0,03778544	0	0,10016742	1,47062891	-0,2172852	1,32508907
k	0	0,24936743	62,1138802	31,6802197	0,03396562	0	0,10016742	1,47062891	-0,16482	2,3491181
k	0	0,25671551	56,4171829	36,3082542	0,09110868	0	0,10016742	1,47062891	-0,1664724	1,44683823
k	0	0,2953116	60,7547874	30,6945229	0,02835464	0	0	1,57079633	-0,1240409	2,41657065
k	0,14689075	0,25952797	61,9837875	29,2679539	0,01429664	0,41151685	0	1,15927948	-0,100736	2,79157877
k	0	0,15591239	52,0465851	44,3534775	0,10924487	0	0,26776333	1,303033	-0,2439958	2,14652258
k	0	0,15591239	52,0465851	44,3534775	0,10924487	0	0,26776333	1,303033	-0,2439958	2,14652258
k	0	0,15591239	52,0465851	44,3534775	0,10924487	0	0,26776333	1,303033	-0,2439958	2,14652258
k	0	0,22826952	60,5384521	34,1429825	0,04448331	0,42498878	0	1,14580754	-0,0618978	2,86807462
k	0,20675158	0,22023762	55,1881218	35,1851692	0,08009235	0,87589139	0,10016742	0,6847192	-0,2042844	2,51826945
k	0	0,24311449	54,5625648	38,3166199	0,11540298	0	0,20135792	1,36943841	-0,1486226	1,98125976
k	0	0,26770488	63,1918144	29,7372932	0,02718312	0	0,26776333	1,303033	-0,1420524	1,88990776
k	0	0,18637626	55,5568428	40,1237068	0,0942603	0	0	1,57079633	-0,2535926	1,92174964
k	0	0,23614602	60,7288704	33,4816628	0,05623141	0	0	1,57079633	-0,289087	1,53741956
k	0	0,24516577	55,4715233	37,518383	0,10597775	0	0,22551341	1,34528292	-0,1830325	1,84253413
k	0,29463999	0,30431424	53,1133003	29,0289268	0,06686831	1,47062891	0	0,10016742	0,0326101	3,00013558
k	0,29463999	0,30431424	53,1133003	29,0289268	0,06686831	1,47062891	0	0,10016742	0,0326101	3,00013558
k	0,29463999	0,30431424	53,1133003	29,0289268	0,06686831	1,47062891	0	0,10016742	0,0326101	3,00013558
k	0,18036363	0,25278696	59,4889336	30,7713737	0,05165096	0,28675655	0,10016742	1,26610367	-0,0427657	2,79983117
k	0	0,19163549	56,2310104	39,4191055	0,08508515	0	0,22551341	1,34528292	-0,2108334	2,62894253
k	0	0,20847648	57,7998009	37,306633	0,07817711	0	0,20135792	1,36943841	-0,2710491	2,23300385
k	0	0,19709765	56,3523102	38,9720078	0,09183393	0	0,10016742	1,47062891	-0,2670814	1,53204146
k	0	0,16074965	61,496666	35,8106384	0,03617818	0,63305184	0,17408301	0,90658109	-0,172484	1,18695302
k	0	0,19858551	57,6383705	37,9272232	0,07371156	0	0,20135792	1,36943841	-0,2368898	2,03582401
k	0	0,22559199	54,4931908	39,2202339	0,11352001	0	0,17408301	1,39671332	-0,3243352	2,10875088
k	0	0,20994067	59,9744148	35,413044	0,05192757	0	0,10016742	1,47062891	-0,2628208	2,18227755
k	0	0,20994067	59,9744148	35,413044	0,05192757	0	0,10016742	1,47062891	-0,2628208	2,18227755
k	0	0,17907607	62,697361	34,0246239	0,03245905	0,36886298	0	1,20193334	-0,202835	2,14994576

k	0	0,1987253	58,6536713	37,0279427	0,06492403	0,33806525	0,17408301	1,18729932	-0,2034009	1,57778406
k	0	0,18202475	63,3345184	33,3067703	0,02861177	0,50017961	0,20135792	1,02439576	-0,1305945	2,37584571
k	0	0,18125662	57,1462402	39,002018	0,07767758	0,42498878	0,10016742	1,1326473	-0,2048432	2,6170452
k	0	0,14189859	58,1753159	39,5222435	0,05501848	0,39769942	0	1,17309691	-0,2851285	2,83060191
k	0	0,16131949	60,7866783	36,4602318	0,04162812	0,50017961	0	1,07061672	-0,1544396	2,5249904
k	0	0,19335854	58,6891747	37,224781	0,06278341	0,17408301	0,10016742	1,36943841	-0,1730243	1,74766937
k	0	0,15683288	60,3138313	37,0618858	0,04299359	0,71516746	0,26776333	0,78539816	-0,2996955	2,32565094
k	0	0,15683288	60,3138313	37,0618858	0,04299359	0,71516746	0,26776333	0,78539816	-0,2996955	2,32565094
k	0	0,15683288	60,3138313	37,0618858	0,04299359	0,71516746	0,26776333	0,78539816	-0,2996955	2,32565094
k	0	0,16064813	54,9875526	41,5718422	0,09405068	0,39769942	0,17408301	1,1326473	-0,1168907	2,68938593
k	0	0,15831541	54,6505508	42,0042343	0,09285381	0,51197269	0	1,05882364	-0,2869869	2,59819367
k	0	0,1345666	57,7794647	40,1144028	0,05536543	0,46364761	0	1,10714872	-0,0859641	2,5249904
k	0	0,14482608	58,9472885	38,7065315	0,0513398	0,33806525	0	1,23273107	-0,102563	3,24178832
k	0	0,11791467	54,1881714	43,8231354	0,07784363	0	0	1,57079633	-0,2343192	2,97276356
k	0	0,1342568	57,2905846	40,5588913	0,05993897	0	0	1,57079633	-0,2020655	2,42338269
k	0	0,13317786	56,8564148	40,9709435	0,06403359	0,22551341	0	1,34528292	-0,1146999	2,21517172
k	0	0,13163131	56,6734428	41,2049217	0,06320369	0	0,10016742	1,47062891	-0,1767926	2,42864261
k	0	0,13163131	56,6734428	41,2049217	0,06320369	0	0,10016742	1,47062891	-0,1767926	2,42864261
k	0	0,13163131	56,6734428	41,2049217	0,06320369	0	0,10016742	1,47062891	-0,1767926	2,42864261
k	0	0,14982619	60,0894585	37,4999466	0,04273911	0,41151685	0,10016742	1,14580754	-0,2366025	2,81551081
k	0	0,16776662	56,4847069	40,1787491	0,07411444	0,36886298	0	1,20193334	-0,1313912	2,78394573
k	0	0,14437445	58,7781754	38,9299316	0,04712774	0	0	1,57079633	-0,138552	2,63144075
k	0	0,13580401	57,1017265	40,6730537	0,06267144	0	0	1,57079633	-0,114457	2,26332575
k	0	0,1776937	56,4495544	39,8025856	0,079041	0	0	1,57079633	-0,1866653	1,94315503
k	0	0,12477155	55,4104576	42,5522079	0,06995712	0	0,20135792	1,36943841	-0,1895962	2,43179093
k	0	0,17056946	55,9474716	40,5046844	0,08173392	0	0	1,57079633	-0,1589653	2,17970259
k	0	0,13890185	57,4200401	40,2731323	0,06247684	0	0	1,57079633	-0,2275901	2,1621268
k	0	0,13890185	57,4200401	40,2731323	0,06247684	0	0	1,57079633	-0,2275901	2,1621268
k	0	0,13890185	57,4200401	40,2731323	0,06247684	0	0	1,57079633	-0,2275901	2,1621268

k	0	0,12751462	55,6513786	42,2638283	0,06843396	0	0	1,57079633	-0,1715228	2,08318934
k	0	0,13294724	56,4508095	41,3736153	0,06473423	0	0	1,57079633	-0,2031558	2,23132361
k	0	0,14921591	58,5977478	38,9175301	0,05243213	0	0	1,57079633	-0,248554	2,2922905
k	0	0,14585329	58,2963448	39,272171	0,0565286	0	0	1,57079633	-0,0816505	2,51435877
k	0	0,1765006	55,5371094	40,6339035	0,08647643	0	0	1,57079633	-0,1568014	1,96629829
k	0	0,15674156	60,524147	36,8826752	0,03956269	0	0	1,57079633	-0,1317554	2,30281513
k	0	0,13859263	57,5314026	40,2162209	0,05867238	0	0	1,57079633	-0,2220765	2,48382543
k	0	0,14745625	58,2460365	39,2893257	0,05534678	0	0	1,57079633	-0,2008926	2,3575225
k	0	0,14745625	58,2460365	39,2893257	0,05534678	0	0	1,57079633	-0,2008926	2,3575225
k	0	0,14745625	58,2460365	39,2893257	0,05534678	0	0	1,57079633	-0,2008926	2,3575225
k	0	0,12260917	53,7644997	44,057766	0,0826746	0	0	1,57079633	-0,3010726	2,22294864
k	0	0,12550908	55,1391983	42,7891731	0,07109706	0	0	1,57079633	-0,1772197	2,46051186
k	0	0,19338715	57,1834068	38,5656815	0,07473158	0	0	1,57079633	-0,3120882	2,07029027
k	0	0,14436518	57,6195908	39,9885521	0,05678992	0	0	1,57079633	-0,1417297	2,39276147
k	0	0,1697484	63,7670555	33,3679314	0,01055055	0	0	1,57079633	0,00610476	3,70851509
k	0	0,13364299	56,2101212	41,5677185	0,06688698	0	0	1,57079633	-0,1217398	2,83017989
k	0	0,17669135	55,8591423	40,4108887	0,08010671	0	0	1,57079633	-0,2208249	2,57749053
k	0	0,15783663	54,0558395	42,5997887	0,09361142	0	0,17408301	1,39671332	-0,1164254	4,2768793
k	0	0,15783663	54,0558395	42,5997887	0,09361142	0	0,17408301	1,39671332	-0,1164254	4,2768793
k	0	0,15783663	54,0558395	42,5997887	0,09361142	0	0,17408301	1,39671332	-0,1164254	4,2768793
k	0	0,16454279	54,4385605	42,0782356	0,08956892	0	0,20135792	1,36943841	-0,1974775	2,91866873
k	0	0,23673218	57,4805832	36,4768333	0,07370659	0	0,28675655	1,28403977	-0,0096009	3,56443961
k	0	0,23952378	64,0375748	30,3210297	0,01145101	0	0	1,57079633	0,03705773	3,1746976

Harbour	tseagrass	tshell	tsand	tchla	chla2	tmgs	mgs2	tsilt	tveryfine	tfine
m	0	0,22551341	1,34528292	4,09948411	16,80577	13,9377186	194,26	0	0,32035643	0,95119749
m	0	0,26776333	1,303033	3,65979098	13,39407	13,773126	189,699	0	0,33778854	0,96568453
m	0	0,39769942	1,17309691	3,35741418	11,27223	13,8349557	191,406	0	0,33520596	0,95556986
m	0	0,20135792	1,36943841	3,88977377	15,13034	13,8956468	193,089	0	0,32444158	0,9554101

m	0,10016742	0,26776333	1,28403977	4,34576115	18,88564	13,911506	193,53	0	0,32570161	0,9503101
m	0	0	1,57079633	3,58802313	12,87391	13,8367988	191,457	0	0,33157549	0,95905909
m	0	0	1,57079633	3,58802313	12,87391	13,8367988	191,457	0	0,33157549	0,95905909
m	0	0	1,57079633	3,58802313	12,87391	13,8367988	191,457	0	0,33157549	0,95905909
m	0	0	1,57079633	5,20154016	27,05602	14,042222	197,184	0	0,33548769	0,90986488
m	0	0,30469265	1,26610367	4,06854397	16,55305	13,7145543	188,089	0	0,34650856	0,96699351
m	0	0,17408301	1,39671332	4,78981315	22,94231	10,8258487	117,199	0,53874647	0,55304382	0,67316454
m	0	0	1,57079633	4,35863625	18,99771	12,1412932	147,411	0,31524645	0,54865164	0,801788
m	0	0,17408301	1,39671332	4,43633633	19,68108	11,2269764	126,045	0,501303	0,53922059	0,7029699
m	0	0,17408301	1,39671332	4,26679388	18,20553	11,3699604	129,276	0,45646593	0,55991714	0,72777409
m	0	0,17408301	1,39671332	5,58273858	31,16697	9,54929317	91,189	0,67993336	0,51583129	0,58450024
m	0	0	1,57079633	5,35922569	28,7213	8,44653775	71,344	0,74335413	0,54110573	0,50525227
m	0	0	1,57079633	5,35922569	28,7213	8,44653775	71,344	0,74335413	0,54110573	0,50525227
m	0	0	1,57079633	5,35922569	28,7213	8,44653775	71,344	0,74335413	0,54110573	0,50525227
m	0	0	1,57079633	4,87824046	23,79723	11,3718952	129,32	0,48330454	0,5370869	0,7150607
m	0	0	1,57079633	4,4444201	19,75287	11,3181271	128,1	0,48262071	0,54469518	0,71360543
m	0	0,17408301	1,39671332	4,20477942	17,68017	13,7805297	189,903	0	0,33759101	0,96431481
m	0	0,54640056	1,02439576	4,4322263	19,64463	13,9853852	195,591	0,09452209	0,30622592	0,93951142
m	0	0,26776333	1,303033	4,36586647	19,06079	13,8097791	190,71	0	0,33220266	0,96513799
m	0,41151685	0,26776333	1,07061672	4,88276971	23,84144	13,9156387	193,645	0	0,31673537	0,96103
m	0	0,30469265	1,26610367	4,51269875	20,36445	14,0310727	196,871	0	0,30182822	0,95298956
m	0	0,61193971	0,95885661	5,71534076	32,66512	13,9628435	194,961	0	0,31134725	0,95715683
m	0	0,61193971	0,95885661	5,71534076	32,66512	13,9628435	194,961	0	0,31134725	0,95715683
m	0	0,61193971	0,95885661	5,71534076	32,66512	13,9628435	194,961	0	0,31134725	0,95715683
m	0	0,59050002	0,98029631	4,03876095	16,31159	13,8898524	192,928	0	0,34673124	0,92780957
m	0	0,64350111	0,92729522	4,78236343	22,871	13,9288908	194,014	0	0,34605406	0,91991843
m	0	0,65388706	0,91690926	4,71531017	22,23415	13,7302221	188,519	0,24456601	0,31189474	0,91422563
m	0	0,98029631	0,59050002	5,13752762	26,39419	13,833257	191,359	0,22078524	0,39349464	0,78989892
m	0	0,69490494	0,87589139	6,79675437	46,19587	13,9044238	193,333	0	0,34563154	0,92549356

m	0	0,7753975	0,79539883	4,52520386	20,47747	13,6358718	185,937	0,24782508	0,32208108	0,91972062
m	0,28675655	0,73531445	0,75538013	4,29716302	18,46561	14,3522124	205,986	0,19588424	0,29782371	0,83418172
m	0	0,88607712	0,6847192	4,26352671	18,17766	14,4581119	209,037	0,22327198	0,33050238	0,76366562
m	0	0,88607712	0,6847192	4,26352671	18,17766	14,4581119	209,037	0,22327198	0,33050238	0,76366562
m	0	0,88607712	0,6847192	4,26352671	18,17766	14,4581119	209,037	0,22327198	0,33050238	0,76366562
m	0	1,15927948	0,41151685	5,107889	26,09053	13,856623	192,006	0,29727092	0,37980096	0,74364646
m	0	1,20193334	0,36886298	4,7917909	22,96126	13,3466101	178,132	0,25591672	0,40607033	0,84621088
m	0	0,8963054	0,67449093	4,96651789	24,6663	14,0333888	196,936	0,22539615	0,39022442	0,75428679
m	0	0,73531445	0,83548187	4,70544472	22,14121	12,3912066	153,542	0,39443906	0,48298842	0,73241506
m	0	0,67449093	0,8963054	4,92554058	24,26095	13,5735036	184,24	0,25789884	0,41549911	0,77486578
m	0	0,71516746	0,85562887	5,38706599	29,02048	12,5669408	157,928	0,38645119	0,46943846	0,72837473
m	0	0,8054035	0,76539283	6,05567007	36,67114	12,2033192	148,921	0,34520419	0,52939791	0,76328476
m	0	0,6847192	0,88607712	4,30525493	18,53522	12,1912674	148,627	0,31715604	0,52843819	0,8338794
m	0	0,6847192	0,88607712	4,30525493	18,53522	12,1912674	148,627	0,31715604	0,52843819	0,8338794
m	0	0,6847192	0,88607712	4,30525493	18,53522	12,1912674	148,627	0,31715604	0,52843819	0,8338794
m	0	0,60126422	0,96953211	4,5919179	21,08571	12,6860947	160,937	0,33698755	0,48032207	0,75966731
m	0	0,5686755	1,00212082	4,80742447	23,11133	12,6242624	159,372	0,34954493	0,48208888	0,75150191
m	0	0,81541619	0,75538013	3,64280798	13,27005	12,4757765	155,645	0,3775557	0,48134773	0,74986095
m	0	0,59050002	0,98029631	5,6604514	32,04071	11,3181712	128,101	0,58051893	0,44897569	0,6159712
m	0	0,59050002	0,98029631	4,78003138	22,8487	12,6220046	159,315	0,31610653	0,49116114	0,79513898
m	0	0,69490494	0,87589139	4,37857283	19,1719	12,5394178	157,237	0,3621381	0,48103539	0,76083278
m	0	0,61193971	0,95885661	4,43726042	19,68928	13,062006	170,616	0,32352187	0,45286622	0,7445373
m	0	0,91690926	0,65388706	5,54324544	30,72757	12,6931084	161,115	0,40989509	0,44476409	0,71976635
m	0	0,91690926	0,65388706	5,54324544	30,72757	12,6931084	161,115	0,40989509	0,44476409	0,71976635
m	0	0,91690926	0,65388706	5,54324544	30,72757	12,6931084	161,115	0,40989509	0,44476409	0,71976635
m	0	0,71516746	0,85562887	5,43559932	29,54574	12,5265318	156,914	0,47589263	0,42411279	0,66605181
m	0	0,72525322	0,8455431	4,93486069	24,35285	12,4112449	154,039	0,44770953	0,44945169	0,70397648
m	0	0,64350111	0,92729522	5,45143009	29,71809	11,5994827	134,548	0,54460639	0,45772378	0,64074304
m	0	0,59050002	0,98029631	4,89711752	23,98176	10,3834965	107,817	0,61720813	0,50789469	0,62419533

m	0	0,28675655	1,28403977	5,20179969	27,05872	12,6743836	160,64	0,4601014	0,41915777	0,68025476
m	0	0,67449093	0,8963054	4,67911851	21,89415	13,357657	178,427	0,30126536	0,43505596	0,74524672
m	0	0,26776333	1,303033	5,22774426	27,32931	11,299115	127,67	0,59495566	0,43382063	0,61301997
m	0	0,36886298	1,20193334	5,34461411	28,5649	11,9565463	142,959	0,51151188	0,44920313	0,66766894
m	0	0,36886298	1,20193334	5,34461411	28,5649	11,9565463	142,959	0,51151188	0,44920313	0,66766894
m	0	0,36886298	1,20193334	5,34461411	28,5649	11,9565463	142,959	0,51151188	0,44920313	0,66766894
m	0	0,52359878	1,04719755	4,52065371	20,43631	11,5094309	132,467	0,46721385	0,532944	0,72434002
m	0	0,35374161	1,21705472	4,47460166	20,02206	12,1308285	147,157	0,38365088	0,51295119	0,7723898
m	0	0,54640056	1,02439576	4,86098344	23,62916	10,9664032	120,262	0,57806825	0,4886902	0,62478004
m	0	0,59050002	0,98029631	5,86064502	34,34716	8,21821148	67,539	0,76332329	0,51551183	0,50119633
m	0	0,42498878	1,14580754	4,43726042	19,68928	10,4276555	108,736	0,62520935	0,49302528	0,62886467
m	0	0,46364761	1,10714872	5,3591016	28,71997	9,10071426	82,823	0,70719126	0,50988871	0,55518504
m	0	0,33806525	1,23273107	5,76247603	33,20613	7,90601037	62,505	0,78793507	0,508517	0,47907348
m	0	0,59050002	0,98029631	8,67313381	75,22325	7,5594312	57,145	0,8187098	0,50456657	0,45529869
m	0	0,59050002	0,98029631	8,67313381	75,22325	7,5594312	57,145	0,8187098	0,50456657	0,45529869
m	0	0,59050002	0,98029631	8,67313381	75,22325	7,5594312	57,145	0,8187098	0,50456657	0,45529869
m	0	0,61193971	0,95885661	5,6578989	32,01182	8,12508461	66,017	0,77079469	0,51464173	0,49670251
m	0	0,72525322	0,8455431	5,68431702	32,31146	8,32394137	69,288	0,76111842	0,48625542	0,52461524
m	0	0,76539283	0,8054035	5,17572604	26,78814	9,83412426	96,71	0,64787558	0,52797242	0,59686526
m	0	0	1,57079633	5,21478859	27,19402	10,0534074	101,071	0,63805506	0,51980486	0,61342559
m	0	0,95885661	0,61193971	4,69769305	22,06832	11,4905178	132,032	0,45747424	0,54369742	0,72810749
m	0	0,45102681	1,11976951	3,97145439	15,77245	11,9344878	142,432	0,34496199	0,55999603	0,78861519
m	0	0,26776333	1,303033	4,27519824	18,27732	11,8119854	139,523	0,38586893	0,55309785	0,7657399
m	0	0,22551341	1,34528292	4,78236343	22,871	10,6860657	114,192	0,56871671	0,53607497	0,65722157
m	0	0,22551341	1,34528292	4,78236343	22,871	10,6860657	114,192	0,56871671	0,53607497	0,65722157
m	0	0,22551341	1,34528292	4,78236343	22,871	10,6860657	114,192	0,56871671	0,53607497	0,65722157
m	0	0	1,57079633	4,52145773	20,44358	10,4570072	109,349	0,59959703	0,52572751	0,63980167
m	0	0,20135792	1,36943841	4,87523333	23,7679	10,9871288	120,717	0,53283751	0,53891744	0,686564
m	0	0,10016742	1,47062891	4,57745563	20,9531	11,2372595	126,276	0,4836855	0,55412204	0,71378628

m	0	0,17408301	1,39671332	5,00022599	25,00226	10,0267143	100,535	0,62162298	0,55072152	0,61242197
m	0	0,10016742	1,47062891	4,73384727	22,40931	10,7062599	114,624	0,58026989	0,5203622	0,6588682
m	0	0	1,57079633	5,18864915	26,92208	10,2343051	104,741	0,61563367	0,53311366	0,6298009
m	0	0	1,57079633	5,72847886	32,81547	10,5390702	111,072	0,59175215	0,52699838	0,64972362
m	0	0	1,57079633	6,03253098	36,39143	9,05455686	81,985	0,71135999	0,51435846	0,55946139
m	0	0	1,57079633	6,03253098	36,39143	9,05455686	81,985	0,71135999	0,51435846	0,55946139
m	0	0	1,57079633	6,03253098	36,39143	9,05455686	81,985	0,71135999	0,51435846	0,55946139
m	0	0	1,57079633	6,01695687	36,20377	10,1353342	102,725	0,62739149	0,52760928	0,62039406
m	0	0	1,57079633	4,4966532	20,21989	9,88043521	97,623	0,63893622	0,54050712	0,60033726
m	0	0	1,57079633	5,53138771	30,59625	11,8215904	139,75	0,45434999	0,50070268	0,75534968
m	0	0	1,57079633	4,48970044	20,15741	11,9445804	142,673	0,4021846	0,52154431	0,77667522
m	0	0	1,57079633	8,3257288	69,31776	9,86686374	97,355	0,67505067	0,47789322	0,60445982
m	0	0	1,57079633	6,27828241	39,41683	11,2989823	127,667	0,55285822	0,47865031	0,69785538
m	0	0	1,57079633	4,4124517	19,46973	11,9765187	143,437	0,39388264	0,52396393	0,77983015
m	0	0	1,57079633	4,4124517	19,46973	11,9765187	143,437	0,39388264	0,52396393	0,77983015
m	0	0	1,57079633	4,4124517	19,46973	11,9765187	143,437	0,39388264	0,52396393	0,77983015
m	0	0	1,57079633	6,26455266	39,24462	9,41493494	88,641	0,71536621	0,45535541	0,58773693
m	0	0	1,57079633	5,00113687	25,01137	11,6418212	135,532	0,47190534	0,51100673	0,73761547
m	0	0	1,57079633	5,51734175	30,44106	10,6107021	112,587	0,59622019	0,51224332	0,65339477
m	0	0	1,57079633	4,96438113	24,64508	12,0034995	144,084	0,36123512	0,53903186	0,7957376
m	0	0,10016742	1,47062891	4,7577274	22,63597	11,0005454	121,012	0,55335766	0,51566988	0,68811626
m	0	0	1,57079633	6,19721228	38,40544	11,7049989	137,007	0,45394337	0,51586287	0,75116282
m	0	0	1,57079633	5,13636642	26,38226	11,6612178	135,984	0,44737054	0,52809316	0,74801845
m	0	0	1,57079633	5,08434362	25,85055	11,5996552	134,552	0,4626421	0,52351044	0,74310976
m	0	0	1,57079633	5,08434362	25,85055	11,5996552	134,552	0,4626421	0,52351044	0,74310976
m	0	0	1,57079633	5,08434362	25,85055	11,5996552	134,552	0,4626421	0,52351044	0,74310976
m	0	0	1,57079633	5,98026254	35,76354	11,3577727	128,999	0,49860316	0,52452643	0,71886893
m	0	0	1,57079633	5,60575954	31,42454	9,98563969	99,713	0,65455491	0,50595707	0,61431597
m	0	0	1,57079633	4,91282505	24,13585	11,4085933	130,156	0,4906754	0,52470289	0,7263357

m	0	0	1,57079633	5,12522975	26,26798	11,4709633	131,583	0,44958788	0,55139371	0,74185043
m	0	0,35374161	1,21705472	4,51209264	20,35898	11,6978203	136,839	0,41119583	0,54984532	0,7605198
m	0	0,17408301	1,39671332	4,73384727	22,40931	10,3843151	107,834	0,61960783	0,51074871	0,64232841
m	0	0	1,57079633	4,78403073	22,88695	11,5626986	133,696	0,42496908	0,55779357	0,75494564
m	0	0	1,57079633	4,63211831	21,45652	11,567195	133,8	0,45634473	0,53290196	0,74286719
m	0	0	1,57079633	4,63211831	21,45652	11,567195	133,8	0,45634473	0,53290196	0,74286719
m	0	0	1,57079633	4,63211831	21,45652	11,567195	133,8	0,45634473	0,53290196	0,74286719
m	0	0,22551341	1,34528292	4,48230967	20,0911	10,9436283	119,763	0,52653069	0,55166483	0,6904921
m	0	0,22551341	1,34528292	5,54676212	30,76657	9,61597629	92,467	0,67703996	0,51575694	0,59295887
m	0	0,28675655	1,28403977	4,8380895	23,40711	11,0505203	122,114	0,52582749	0,53834685	0,69656994
m	0	0,22551341	1,34528292	3,58802313	12,87391	12,1328892	147,207	0,26795543	0,55830927	0,84984738
m	0	0,73531445	0,83548187	4,69604727	22,05286	12,2554886	150,197	0,40733576	0,4819786	0,77006266
m	0	0,39769942	1,17309691	4,24566367	18,02566	12,0143664	144,345	0,31065914	0,55656211	0,83275664
m	0	0	1,57079633	5,95503065	35,46239	11,7841843	138,867	0,38417926	0,55634505	0,77390945
m	0	0,17408301	1,39671332	2,70501941	7,31713	11,9579262	142,992	0,32955924	0,56503284	0,79598345
m	0	0,17408301	1,39671332	2,70501941	7,31713	11,9579262	142,992	0,32955924	0,56503284	0,79598345
m	0	0,17408301	1,39671332	2,70501941	7,31713	11,9579262	142,992	0,32955924	0,56503284	0,79598345
m	0	0,20135792	1,36943841	5,0242084	25,24267	11,3579488	129,003	0,50838895	0,51617557	0,70435912
m	0	0,26776333	1,303033	4,55790193	20,77447	11,6076699	134,738	0,49403438	0,4974836	0,72273969
m	0	0,87589139	0,69490494	4,99314831	24,93153	12,5716745	158,047	0,32450413	0,48779791	0,80681751
m	0	0,36886298	1,20193334	4,87216584	23,738	12,6900355	161,037	0,26835387	0,47757359	0,86825689
m	0	0,54640056	1,02439576	4,40019545	19,36172	13,363308	178,578	0,22822475	0,40517893	0,86935372
m	0	0,92729522	0,64350111	4,90455401	24,05465	12,7995703	163,829	0,29290385	0,45731587	0,85073444
m	0	0,65388706	0,91690926	3,78954483	14,36065	13,2846152	176,481	0,22194253	0,38585803	0,92240908
m	0	0,61193971	0,95885661	4,32745768	18,72689	12,8381463	164,818	0,24666228	0,46582431	0,87368959
m	0	0,61193971	0,95885661	4,32745768	18,72689	12,8381463	164,818	0,24666228	0,46582431	0,87368959
m	0	0,61193971	0,95885661	4,32745768	18,72689	12,8381463	164,818	0,24666228	0,46582431	0,87368959
m	0	0,35374161	1,21705472	3,37710675	11,40485	13,0926697	171,418	0,23289673	0,40925627	0,92356081
m	0	0,54640056	1,02439576	4,54288455	20,6378	12,8866986	166,067	0,23492541	0,4630018	0,87769799

m	0	0,41151685	1,15927948	4,34576115	18,88564	12,7597414	162,811	0,31758768	0,44949411	0,84758839
m	0	0,47603382	1,09476251	3,65979098	13,39407	13,1706492	173,466	0,22903768	0,42699135	0,87744673
m	0	0,41151685	1,15927948	4,98745426	24,8747	12,9297718	167,179	0,46401526	0,39318334	0,66767141
m	0	0,41151685	1,15927948	3,52937388	12,45648	13,1961737	174,139	0,23010881	0,42099813	0,88141094
m	0	0,46364761	1,10714872	3,78095094	14,29559	13,3460856	178,118	0,20056173	0,41084254	0,88471681
m	0	0,33806525	1,23273107	4,12572781	17,02163	13,2212329	174,801	0,22825497	0,41981211	0,87799688
m	0	0,33806525	1,23273107	4,12572781	17,02163	13,2212329	174,801	0,22825497	0,41981211	0,87799688
m	0	0,33806525	1,23273107	4,12572781	17,02163	13,2212329	174,801	0,22825497	0,41981211	0,87799688
m	0	0,41151685	1,15927948	4,04237059	16,34076	13,0387883	170,01	0,34216513	0,42478402	0,79293095
m	0	0,33806525	1,23273107	4,7917909	22,96126	11,6849048	136,537	0,56180403	0,42938521	0,66016106
m	0	0,17408301	1,39671332	3,65979098	13,39407	12,9208359	166,948	0,42359836	0,40598421	0,72491587
m	0	0,39769942	1,17309691	4,59800174	21,14162	12,7319284	162,102	0,46198604	0,40975362	0,68830205
m	0	0,6847192	0,88607712	4,82022925	23,23461	12,8219733	164,403	0,42189329	0,42349307	0,70671906
m	0,22551341	0,51197269	1,00212082	4,1260429	17,02423	13,5091451	182,497	0,36409922	0,38873226	0,71742942
m	0	0,50017961	1,07061672	5,11190375	26,13156	11,9496862	142,795	0,54882635	0,42034652	0,63404561
m	0	0,47603382	1,09476251	5,02990159	25,29991	12,4154742	154,144	0,49016094	0,42410359	0,66536451
m	0	0,47603382	1,09476251	5,02990159	25,29991	12,4154742	154,144	0,49016094	0,42410359	0,66536451
m	0	0,47603382	1,09476251	5,02990159	25,29991	12,4154742	154,144	0,49016094	0,42410359	0,66536451
m	0	0,39769942	1,17309691	4,92554058	24,26095	13,8295698	191,257	0,27008965	0,39373984	0,75123288
m	0	0,67449093	0,8963054	4,95970967	24,59872	12,4501807	155,007	0,50238794	0,40168741	0,67833162
m	0	0,52359878	1,04719755	4,82759567	23,30568	12,7110975	161,572	0,4502578	0,42132258	0,68524497
m	0	0,55759883	1,0131975	4,80603059	23,09793	13,1037018	171,707	0,35170142	0,4337973	0,73767152
m	1,20193334	0	0,36886298	5,40816697	29,24827	15,1643991	229,959	0,25743249	0,32362421	0,66792098
m	0	0,50017961	1,07061672	4,26564298	18,19571	13,0569522	170,484	0,38656275	0,42417664	0,71193989
m	0	0,28675655	1,28403977	4,26241598	18,16819	12,1920876	148,647	0,52104785	0,42120158	0,64244891
m	0	0,47603382	1,09476251	4,89930913	24,00323	12,5962296	158,665	0,51456319	0,39930749	0,61112378
m	0	0,47603382	1,09476251	4,89930913	24,00323	12,5962296	158,665	0,51456319	0,39930749	0,61112378
m	0	0,47603382	1,09476251	4,89930913	24,00323	12,5962296	158,665	0,51456319	0,39930749	0,61112378
m	1,34528292	0	0,22551341	5,28757884	27,95849	14,8434497	220,328	0,28837911	0,33471057	0,67134855

m	0,79539883	0,17408301	0,74535537	4,91012423	24,10932	15,2305286	231,969	0,38427522	0,3262157	0,57137521
m	0,59050002	0,22551341	0,92729522	5,20128446	27,05336	14,1869658	201,27	0,41724396	0,34834793	0,60938904
m	1,303033	0	0,26776333	5,32577788	28,36391	14,4890648	209,933	0,26337676	0,35708206	0,70658996
m	0	0,42498878	1,14580754	4,91282505	24,13585	13,7224998	188,307	0,26681896	0,30738972	0,90150441
m	1,14580754	0	0,42498878	4,48230967	20,0911	13,5387222	183,297	0,40965277	0,3792212	0,66810555
m	0,61193971	0,50017961	0,72525322	5,4502899	29,70566	14,2871271	204,122	0,40172812	0,32721222	0,64037094
m	0	0,52359878	1,04719755	3,74591511	14,03188	14,322814	205,143	0,42521862	0,30799941	0,63166393
m	0	0,52359878	1,04719755	3,74591511	14,03188	14,322814	205,143	0,42521862	0,30799941	0,63166393
m	0	0,52359878	1,04719755	3,74591511	14,03188	14,322814	205,143	0,42521862	0,30799941	0,63166393
m	0	0,36886298	1,20193334	5,57830888	31,11753	14,6196443	213,734	0,22836889	0,26836058	0,80057619
m	0	0,41151685	1,15927948	5,59042217	31,25282	14,5544495	211,832	0,24800317	0,26318401	0,8034719
m	0	0,42498878	1,14580754	4,85853785	23,60539	14,0665561	197,868	0,15362807	0,30014583	0,90594386
m	0	0,39769942	1,17309691	4,55109657	20,71248	14,059623	197,673	0	0,32849756	0,91388781
m	0	0,20135792	1,36943841	3,74841833	14,05064	13,844349	191,666	0	0,3245162	0,96549015
m	0	0,20135792	1,36943841	5,06251222	25,62903	13,7650282	189,476	0,21402628	0,31401969	0,92557597
m	0	0,45102681	1,11976951	4,88251779	23,83898	14,0091399	196,256	0	0,33485203	0,91569716
m	0	0,33806525	1,23273107	4,5243983	20,47018	13,8995683	193,198	0	0,34439395	0,92740699
m	0	0,33806525	1,23273107	4,5243983	20,47018	13,8995683	193,198	0	0,34439395	0,92740699
m	0	0,33806525	1,23273107	4,5243983	20,47018	13,8995683	193,198	0	0,34439395	0,92740699
m	0	0,22551341	1,34528292	4,77322951	22,78372	13,7754492	189,763	0	0,33282446	0,97080483
m	0	0,20135792	1,36943841	3,88819495	15,11806	13,7260701	188,405	0	0,30784497	1,0159863
m	0	0,22551341	1,34528292	4,40019545	19,36172	13,6915302	187,458	0	0,34388104	0,97525072
m	0	0,63305184	0,93774449	4,21084315	17,7312	15,1546363	229,663	0,18170138	0,23879373	0,76963735
m	0	0,36886298	1,20193334	4,10390546	16,84204	13,8191896	190,97	0,451306	0,32844467	0,64671271
m	0	0,60126422	0,96953211	5,31885984	28,29027	15,0680457	227,046	0,26146294	0,22519098	0,74996812
m	0	0,54640056	1,02439576	5,92665673	35,12526	14,991631	224,749	0,29266659	0,26956213	0,70607099
m	0	0,55759883	1,0131975	5,5790017	31,12526	14,6175579	213,673	0,23383166	0,25632018	0,8088808
m	0	0,52359878	1,04719755	6,3175644	39,91162	14,9851593	224,555	0,27272349	0,22474065	0,75458406
m	0	0,52359878	1,04719755	6,3175644	39,91162	14,9851593	224,555	0,27272349	0,22474065	0,75458406

m	0	0,52359878	1,04719755	6,3175644	39,91162	14,9851593	224,555	0,27272349	0,22474065	0,75458406
m	0	0,28675655	1,28403977	6,05803186	36,69975	13,9476163	194,536	0,39193651	0,32281455	0,71033249
m	0	0,30469265	1,26610367	5,94140135	35,30025	14,0093897	196,263	0,38792358	0,32077803	0,7075983
m	0	0,10016742	1,47062891	4,5104124	20,34382	14,0789559	198,217	0,34729125	0,33898705	0,71821943
m	0	0,64350111	0,92729522	5,15053978	26,52806	13,5133268	182,61	0,43539469	0,36611039	0,65686488
m	0	0,26776333	1,303033	4,84144813	23,43962	14,0423645	197,188	0,41868239	0,31703502	0,6693175
m	0	0,28675655	1,28403977	4,78981315	22,94231	14,3173322	204,986	0,34565209	0,3313124	0,69373244
m	0	0,22551341	1,34528292	5,01478315	25,14805	13,9756932	195,32	0,38611794	0,33248378	0,70169363
m	0	0,73531445	0,83548187	4,83155772	23,34395	14,260014	203,348	0,37646144	0,3279844	0,67721126
m	0	0,73531445	0,83548187	4,83155772	23,34395	14,260014	203,348	0,37646144	0,3279844	0,67721126
m	0	0,73531445	0,83548187	4,83155772	23,34395	14,260014	203,348	0,37646144	0,3279844	0,67721126
m	0	0,45102681	1,11976951	5,66753562	32,12096	14,113327	199,186	0,40961663	0,32914411	0,65499184
m	0	0,52359878	1,04719755	5,04467343	25,44873	13,0334569	169,871	0,49360083	0,3628529	0,64816192
m	0	0,75538013	0,81541619	4,86903481	23,7075	14,2389606	202,748	0,27388507	0,36850111	0,71864585
m	0	0,79539883	0,7753975	5,55286052	30,83426	14,7152982	216,54	0,32625671	0,32832265	0,66096633
m	0	0,51197269	1,05882364	4,75874353	22,64564	14,1797391	201,065	0,30687818	0,37224483	0,69369206
m	0	0,64350111	0,92729522	4,51209264	20,35898	14,7728129	218,236	0,26769987	0,33232719	0,69545019
m	0,55759883	0,54640056	0,73531445	3,47939363	12,10618	15,3109438	234,425	0,29709275	0,3199784	0,63367859
m	1,28403977	0	0,28675655	3,77016047	14,21411	17,4793307	305,527	0,19696156	0,2866444	0,56797116
m	1,28403977	0	0,28675655	3,77016047	14,21411	17,4793307	305,527	0,19696156	0,2866444	0,56797116
m	1,28403977	0	0,28675655	3,77016047	14,21411	17,4793307	305,527	0,19696156	0,2866444	0,56797116
m	1,57079633	0	0	5,44328026	29,6293	13,799384	190,423	0,29862096	0,40041727	0,71778842
m	1,57079633	0	0	4,93732721	24,3772	14,2488947	203,031	0,2791954	0,37389669	0,70261943
m	0,36886298	0,72525322	0,71516746	4,66167245	21,73119	14,2987762	204,455	0,34506096	0,35391359	0,66576506
m	0	0,42498878	1,14580754	4,4428178	19,73863	13,960086	194,884	0,28094072	0,38365203	0,7329623
m	0,55759883	0,52359878	0,75538013	4,59946953	21,15512	14,3832542	206,878	0,26700982	0,36197568	0,71120519
m	0	0,71516746	0,85562887	4,43900101	19,70473	14,1503357	200,232	0,30804946	0,36449047	0,70805256
m	0	0,67449093	0,8963054	5,01805241	25,18085	13,6330114	185,859	0,35032119	0,38667371	0,7166125
m	0	0,5686755	1,00212082	4,68897537	21,98649	13,6349551	185,912	0,30011028	0,38802938	0,76881312

m	0	0,5686755	1,00212082	4,68897537	21,98649	13,6349551	185,912	0,30011028	0,38802938	0,76881312
m	0	0,5686755	1,00212082	4,68897537	21,98649	13,6349551	185,912	0,30011028	0,38802938	0,76881312
m	0	0,45102681	1,11976951	4,61305647	21,28029	13,4724905	181,508	0,32294067	0,402927	0,75101146
m	0	0,50017961	1,07061672	3,49460155	12,21224	13,4655486	181,321	0,28884629	0,41955103	0,75890437
m	0,17408301	0,36886298	1,15927948	4,62660459	21,40547	13,1836641	173,809	0,32191169	0,42505547	0,77702247
m	0	0,41151685	1,15927948	4,82106316	23,24265	13,4111148	179,858	0,20967516	0,37046956	0,92589818
m	0	0,59050002	0,98029631	4,78794946	22,92446	13,0412039	170,073	0,41503195	0,40713572	0,7081729
m	0,47603382	0,51197269	0,83548187	5,17572604	26,78814	13,8094895	190,702	0,20013412	0,35909134	0,85801245
m	0	0,55759883	1,0131975	4,75169128	22,57857	13,335404	177,833	0,29339505	0,41831869	0,78738497
m	0	0,35374161	1,21705472	4,55790193	20,77447	13,2420165	175,351	0,40906764	0,38470275	0,71867006
m	0	0,35374161	1,21705472	4,55790193	20,77447	13,2420165	175,351	0,40906764	0,38470275	0,71867006
m	0	0,35374161	1,21705472	4,55790193	20,77447	13,2420165	175,351	0,40906764	0,38470275	0,71867006
m	0	0,45102681	1,11976951	3,82415219	14,62414	13,397649	179,497	0,21865257	0,39636488	0,88267059
m	0	0,51197269	1,05882364	4,66167245	21,73119	13,205832	174,394	0,24315281	0,41325678	0,88038997
m	0	0,51197269	1,05882364	4,9599748	24,60135	13,7088293	187,932	0,19771078	0,37236775	0,8605676
m	0	0,22551341	1,34528292	3,54357729	12,55694	10,114544	102,304	0,63275085	0,51230897	0,60498995
m	0	0,61193971	0,95885661	4,23620703	17,94545	13,3685078	178,717	0,19772924	0,37840775	0,93225581
m	0	0,51197269	1,05882364	4,66885318	21,79819	13,6616983	186,642	0,19217891	0,34190182	0,92505261
m	0	0,46364761	1,10714872	3,77981613	14,28701	13,2285676	174,995	0,23885871	0,41636655	0,87459684
m	0	0,55759883	1,0131975	4,75248041	22,58607	13,9709699	195,188	0,18486781	0,3452469	0,85590985
m	0	0,55759883	1,0131975	4,75248041	22,58607	13,9709699	195,188	0,18486781	0,3452469	0,85590985
m	0	0,55759883	1,0131975	4,75248041	22,58607	13,9709699	195,188	0,18486781	0,3452469	0,85590985
m	0	0,51197269	1,05882364	4,41127079	19,45931	13,1362476	172,561	0,20790061	0,43708832	0,88545054
m	0	0,28675655	1,28403977	5,16250521	26,65146	12,8782375	165,849	0,3239907	0,45251512	0,80342507
m	0	0	1,57079633	5,22827218	27,33483	11,8833497	141,214	0,47657141	0,4805119	0,72988468
m	0	0	1,57079633	4,61225867	21,27293	12,1217573	146,937	0,33868624	0,53636402	0,80447399
m	0	0,28675655	1,28403977	4,10878449	16,88211	12,5639166	157,852	0,31762301	0,49254879	0,81195835
m	0	0,50017961	1,07061672	3,78210788	14,30434	12,9810246	168,507	0,1999862	0,43168174	0,9400671
m	0	0,17408301	1,39671332	3,81937168	14,5876	13,0571436	170,489	0,15334016	0,43200085	0,94959048

m	0	0,26776333	1,303033	3,64280798	13,27005	12,241977	149,866	0,35797916	0,51120316	0,79724437
m	0	0,26776333	1,303033	3,64280798	13,27005	12,241977	149,866	0,35797916	0,51120316	0,79724437
m	0	0,26776333	1,303033	3,64280798	13,27005	12,241977	149,866	0,35797916	0,51120316	0,79724437
m	0	0	1,57079633	4,88690904	23,88188	12,3447155	152,392	0,2751265	0,52346909	0,85821024
m	0	0,22551341	1,34528292	3,96960074	15,75773	12,2725303	150,615	0,29947397	0,52352718	0,84917275
m	0	0	1,57079633	4,89711752	23,98176	12,1517488	147,665	0,35342792	0,52478223	0,79823827
m	0	0	1,57079633	3,93902272	15,5159	12,2468772	149,986	0,34627198	0,51557442	0,80640234
m	0	0	1,57079633	3,76388098	14,1668	12,0906576	146,184	0,36186364	0,52802285	0,79445456
m	0	0,10016742	1,47062891	3,96157292	15,69406	11,8490928	140,401	0,32612048	0,57220807	0,82037077
m	0	0	1,57079633	3,9923602	15,93894	12,0959497	146,312	0,31331315	0,54180717	0,84084131
m	0	0	1,57079633	5,16302431	26,65682	12,2160141	149,231	0,27663298	0,52559757	0,88697064
m	0	0	1,57079633	5,16302431	26,65682	12,2160141	149,231	0,27663298	0,52559757	0,88697064
m	0	0	1,57079633	5,16302431	26,65682	12,2160141	149,231	0,27663298	0,52559757	0,88697064
m	0	0	1,57079633	4,36765383	19,0764	12,1657306	148,005	0,35100946	0,52332715	0,80385525
m	0	0	1,57079633	3,83499804	14,70721	12,517348	156,684	0,28180134	0,49348122	0,86918748
m	0	0	1,57079633	3,96387058	15,71227	12,3605421	152,783	0,30316866	0,5068936	0,85807774
m	0	0	1,57079633	4,55946378	20,78871	12,6053163	158,894	0,30572164	0,46940376	0,86534802
m	0	0	1,57079633	4,56935335	20,87899	12,459374	155,236	0,31107189	0,48851118	0,85897411
m	0	0	1,57079633	4,92977079	24,30264	11,8925607	141,433	0,45672103	0,49165605	0,75115881
m	0	0	1,57079633	3,85208126	14,83853	12,3580338	152,721	0,38406326	0,48188741	0,78823158
m	0	0	1,57079633	4,86489363	23,66719	12,090244	146,174	0,42227621	0,49001947	0,77367689
m	0	0	1,57079633	4,86489363	23,66719	12,090244	146,174	0,42227621	0,49001947	0,77367689
m	0	0	1,57079633	4,86489363	23,66719	12,090244	146,174	0,42227621	0,49001947	0,77367689
m	0	0	1,57079633	4,28544747	18,36506	12,1112345	146,682	0,42948507	0,48176066	0,77255955
m	0	0	1,57079633	4,9531495	24,53369	12,3800646	153,266	0,35934551	0,49037363	0,80520094
m	0	0	1,57079633	5,44925683	29,6944	12,5190255	156,726	0,30467569	0,46535056	0,89438844
m	0	0	1,57079633	4,97160839	24,71689	12,4474495	154,939	0,41168607	0,45188501	0,78005126
m	0	0	1,57079633	4,03655918	16,29381	12,6371278	159,697	0,27824408	0,4591831	0,90908994
m	0	0	1,57079633	5,04196291	25,42139	11,9297108	142,318	0,47293027	0,47543279	0,74291848

m	0	0	1,57079633	4,32093971	18,67052	12,294755	151,161	0,3796043	0,4901247	0,79600016
m	0	0	1,57079633	5,62868724	31,68212	11,4723145	131,614	0,50909348	0,50126314	0,7145276
m	0	0	1,57079633	5,62868724	31,68212	11,4723145	131,614	0,50909348	0,50126314	0,7145276
m	0	0	1,57079633	5,62868724	31,68212	11,4723145	131,614	0,50909348	0,50126314	0,7145276
m	0	0	1,57079633	6,3594245	40,44228	11,7664353	138,449	0,50653067	0,46736018	0,72311602
m	0	0	1,57079633	5,25515271	27,61663	11,8838546	141,226	0,49621434	0,45963989	0,73617218
m	0	0	1,57079633	5,09979411	26,0079	12,1234071	146,977	0,41198704	0,40739046	0,67123829
m	0	0	1,57079633	4,12003155	16,97466	14,2627487	203,426	0,36506823	0,32117498	0,69273209
m	0	0	1,57079633	5,02839139	25,28472	12,2728155	150,622	0,45295073	0,41614509	0,71605781
m	0	0	1,57079633	4,99314831	24,93153	12,3720653	153,068	0,4902044	0,38532229	0,69948798
m	0	0	1,57079633	4,48230967	20,0911	12,4897958	155,995	0,4388488	0,34215675	0,68920711
m	0	0	1,57079633	4,48230967	20,0911	12,4897958	155,995	0,4388488	0,34215675	0,68920711
m	0	0	1,57079633	4,48230967	20,0911	12,4897958	155,995	0,4388488	0,34215675	0,68920711
m	0	0	1,57079633	5,83184619	34,01043	12,6715824	160,569	0,42513758	0,3293541	0,73209687
m	0	0,17408301	1,39671332	5,01309685	25,13114	12,7975388	163,777	0,37392596	0,31458243	0,66274031
m	0	0,10016742	1,47062891	5,43765023	29,56804	13,0965263	171,519	0,26884532	0,32930394	0,71089305
m	0	0	1,57079633	5,22936134	27,34622	13,0158749	169,413	0,32266361	0,38140477	0,89948289
m	0	0	1,57079633	4,13035229	17,05981	13,0218278	169,568	0,30142937	0,41391207	0,87045572
m	0	0	1,57079633	5,21600422	27,2067	12,7507647	162,582	0,38104325	0,43175949	0,8008081
m	0	0	1,57079633	5,84097338	34,11697	12,8879013	166,098	0,34014511	0,43367812	0,82138884
m	0	0	1,57079633	5,35037756	28,62654	13,4332051	180,451	0,28015069	0,33793803	0,91597848
m	0	0	1,57079633	5,35037756	28,62654	13,4332051	180,451	0,28015069	0,33793803	0,91597848
m	0	0	1,57079633	5,35037756	28,62654	13,4332051	180,451	0,28015069	0,33793803	0,91597848
m	0	0	1,57079633	4,11486573	16,93212	12,791677	163,627	0,35329178	0,39750782	0,88934163
m	0	0	1,57079633	5,26377621	27,70734	12,8830121	165,972	0,30746492	0,40589876	0,90822231
m	0	0	1,57079633	4,47460166	20,02206	12,6978345	161,235	0,37634647	0,43978195	0,80391309
m	0	0,10016742	1,47062891	5,71384634	32,64804	12,3548776	152,643	0,47398397	0,42343955	0,74580279
m	0	0,10016742	1,47062891	4,34847904	18,90927	13,0205223	169,534	0,30656762	0,38750017	0,90898309
m	0	0	1,57079633	4,77750772	22,82458	12,2451623	149,944	0,44999832	0,45171214	0,7644102

m	0	0	1,57079633	5,60669778	31,43506	12,634912	159,641	0,3908156	0,44010855	0,79604567
m	0	0	1,57079633	3,94171536	15,53712	13,1338875	172,499	0,21675645	0,37396272	0,98630261
m	0	0	1,57079633	3,94171536	15,53712	13,1338875	172,499	0,21675645	0,37396272	0,98630261
m	0	0	1,57079633	3,94171536	15,53712	13,1338875	172,499	0,21675645	0,37396272	0,98630261
m	0	0	1,57079633	3,97555028	15,805	13,2534147	175,653	0,20584923	0,35792294	0,98937251
m	0	0	1,57079633	4,59638227	21,12673	13,3243386	177,538	0,27506617	0,35729788	0,91522228
m	0	0	1,57079633	3,83333536	14,69446	13,2480565	175,511	0,22778824	0,38158159	0,9382899
m	0	0,20135792	1,36943841	3,80197054	14,45498	13,2473771	175,493	0,23024749	0,38243224	0,93550765
m	0	0,28675655	1,28403977	4,10878449	16,88211	12,8489299	165,095	0,31707114	0,43356258	0,85731205
m	0	0	1,57079633	4,8844836	23,85818	13,2309108	175,057	0,24402946	0,38084449	0,92999047
m	0	0,17408301	1,39671332	4,38700923	19,24585	13,0680144	170,773	0,28550428	0,39205802	0,91210507
m	0	0,10016742	1,47062891	4,39354299	19,30322	12,9718541	168,269	0,25446342	0,41657428	0,92374428
m	0	0,10016742	1,47062891	4,39354299	19,30322	12,9718541	168,269	0,25446342	0,41657428	0,92374428
m	0	0,10016742	1,47062891	4,39354299	19,30322	12,9718541	168,269	0,25446342	0,41657428	0,92374428
m	0	0,39769942	1,17309691	5,11313896	26,14419	13,0466088	170,214	0,24490711	0,4094373	0,92589027
m	0	0,28675655	1,28403977	5,70855761	32,58763	11,6208433	135,044	0,52368133	0,47081928	0,70448304
m	0	0	1,57079633	5,96846044	35,62252	11,8289053	139,923	0,49829903	0,47046125	0,71294861
m	0	0,26776333	1,303033	5,33653914	28,47865	11,6447842	135,601	0,53136759	0,46041889	0,70199016
m	0	0,33806525	1,23273107	4,71994597	22,27789	13,5399409	183,33	0,20311606	0,38436312	0,88165646
m	0	0,22551341	1,34528292	5,04598751	25,46199	11,7297485	137,587	0,52859228	0,45247939	0,70863132
m	0	0,33806525	1,23273107	5,02990159	25,29991	12,4072156	153,939	0,41243735	0,45833938	0,77731831
m	0	0,33806525	1,23273107	4,69190899	22,01401	12,7736447	163,166	0,34790121	0,45241886	0,79938139
m	0	0,33806525	1,23273107	4,69190899	22,01401	12,7736447	163,166	0,34790121	0,45241886	0,79938139
m	0	0,33806525	1,23273107	4,69190899	22,01401	12,7736447	163,166	0,34790121	0,45241886	0,79938139
m	0	0,42498878	1,14580754	4,23683962	17,95081	13,2534147	175,653	0,22465435	0,39000564	0,92335325
m	0	0,50017961	1,07061672	4,62930988	21,43051	13,3846554	179,149	0,22684917	0,3970166	0,87923687
m	0	0,45102681	1,11976951	4,59638227	21,12673	13,4112266	179,861	0,25978568	0,38621825	0,86365396
m	0	0,30469265	1,26610367	5,107889	26,09053	13,4523976	180,967	0,21778305	0,38981692	0,8828983
m	0	0,55759883	1,0131975	4,4966532	20,21989	12,7366401	162,222	0,43938375	0,40529692	0,75327667

m	0	0,39769942	1,17309691	4,93974392	24,40107	13,4539214	181,008	0,20647325	0,36699415	0,92485008
m	0	0,96953211	0,60126422	4,42447059	19,57594	13,4674793	181,373	0,2036963	0,36739676	0,92334535
m	0	0,61193971	0,95885661	4,64699042	21,59452	14,2173486	202,133	0,18114218	0,32036757	0,84346218
m	0	0,61193971	0,95885661	4,64699042	21,59452	14,2173486	202,133	0,18114218	0,32036757	0,84346218
m	0	0,61193971	0,95885661	4,64699042	21,59452	14,2173486	202,133	0,18114218	0,32036757	0,84346218
m	0	0,61193971	0,95885661	4,16335201	17,3335	14,5995548	213,147	0,19952825	0,32905108	0,76040676
m	0	0,55759883	1,0131975	4,07536992	16,60864	13,8923	192,996	0,22274915	0,34438073	0,84832565
m	0	0,72525322	0,8455431	4,23089589	17,90048	13,523535	182,886	0,30957654	0,36045816	0,8255478
m	0,73531445	0,59050002	0,51197269	4,28367482	18,34987	13,6569762	186,513	0,29002572	0,3523501	0,82787845
m	0	0,6847192	0,88607712	5,15003981	26,52291	14,0044279	196,124	0,24907763	0,37096797	0,76777602
m	0	0,45102681	1,11976951	4,53482414	20,56463	13,0880862	171,298	0,23581148	0,40795299	0,92497834
m	0	0,69490494	0,87589139	4,21516785	17,76764	14,0475977	197,335	0,22557151	0,33212789	0,83037068
m	0	0,55759883	1,0131975	4,52691065	20,49292	14,0386965	197,085	0,23655268	0,36395014	0,78328416
m	0	0,55759883	1,0131975	4,52691065	20,49292	14,0386965	197,085	0,23655268	0,36395014	0,78328416
m	0	0,55759883	1,0131975	4,52691065	20,49292	14,0386965	197,085	0,23655268	0,36395014	0,78328416
m	0	0,46364761	1,10714872	5,17572604	26,78814	14,1530915	200,31	0,25055837	0,36271214	0,75448778
m	0	0,76539283	0,8054035	5,57533407	31,08435	14,1608969	200,531	0,29852521	0,34754271	0,73369065
m	0	0,67449093	0,8963054	5,29527336	28,03992	14,616087	213,63	0,23005002	0,33751116	0,72941195
m	0	0,64350111	0,92729522	5,60150337	31,37684	14,544621	211,546	0,23653944	0,33943433	0,73237494
m	0	0,46364761	1,10714872	5,25055902	27,56837	13,6189574	185,476	0,44007171	0,35836477	0,64594219
m	0	0,75538013	0,81541619	5,17572604	26,78814	14,072029	198,022	0,22836159	0,36973576	0,7737067
m	0	0,7753975	0,79539883	5,22671886	27,31859	13,1701177	173,452	0,30916944	0,40217766	0,84158454
m	0	0,50017961	1,07061672	5,01643399	25,16461	13,3524155	178,287	0,42183092	0,38011089	0,67935568
m	0	0,50017961	1,07061672	5,01643399	25,16461	13,3524155	178,287	0,42183092	0,38011089	0,67935568
m	0	0,50017961	1,07061672	5,01643399	25,16461	13,3524155	178,287	0,42183092	0,38011089	0,67935568
m	0	0,46364761	1,10714872	5,09979411	26,0079	14,1492049	200,2	0,30263532	0,36186138	0,71591262
m	0	0,61193971	0,95885661	4,56139452	20,80632	15,2111144	231,378	0,27796951	0,30715546	0,66410934
m	0	0,42498878	1,14580754	5,20377843	27,07931	14,0942187	198,647	0,31487355	0,36364115	0,70790692
m	0,60126422	0,8054035	0,41151685	4,76913409	22,74464	14,7344834	217,105	0,30269222	0,33445425	0,67145593

m	1,17309691	0,17408301	0,35374161	3,83333536	14,69446	12,8253655	164,49	0,46176633	0,40947742	0,66517239
m	0	0,35374161	1,21705472	4,58369938	21,0103	15,2346316	232,094	0,25186604	0,32309149	0,66467914
m	0,93774449	0,22551341	0,57963974	1,51399505	2,292181	14,6901668	215,801	0,31149806	0,33190705	0,67328486
m	0,96953211	0,26776333	0,52359878	4,30883163	18,56603	12,8758301	165,787	0,37026714	0,45047865	0,72633671
m	0,96953211	0,26776333	0,52359878	4,30883163	18,56603	12,8758301	165,787	0,37026714	0,45047865	0,72633671
m	0,96953211	0,26776333	0,52359878	4,30883163	18,56603	12,8758301	165,787	0,37026714	0,45047865	0,72633671
m	1,10714872	0,17408301	0,43814903	4,95970967	24,59872	13,7873855	190,092	0,32649297	0,39462069	0,70516782
m	1,20193334	0	0,36886298	4,92374451	24,24326	14,4117313	207,698	0,29565525	0,35548151	0,69832888
m	1,26610367	0	0,30469265	4,32716535	18,72436	15,4419558	238,454	0,1807015	0,29956683	0,69767623
m	1,02439576	0	0,54640056	5,30708018	28,1651	13,0005769	169,015	0,44007599	0,48791407	0,7891694
m	0	0,36886298	1,20193334	6,59280441	43,46507	14,2627487	203,426	0,36506823	0,39455236	0,91876374
m	0	0,42498878	1,14580754	4,74363047	22,50203	13,4754963	181,589	0,33440007	0,49799706	0,76137072
m	0,93774449	0,28675655	0,54640056	4,96787782	24,67981	13,8254476	191,143	0,34128702	0,44997202	0,74896383
m	0,79539883	0,46364761	0,5686755	5,18852098	26,92075	13,8795533	192,642	0,39864639	0,40778519	0,73586971
m	0,79539883	0,46364761	0,5686755	5,18852098	26,92075	13,8795533	192,642	0,39864639	0,40778519	0,73586971
m	0,79539883	0,46364761	0,5686755	5,18852098	26,92075	13,8795533	192,642	0,39864639	0,40778519	0,73586971
m	0	0,26776333	1,303033	4,72476984	22,32345	14,0068198	196,191	0,35850038	0,43403598	0,76137843
m	0	0,22551341	1,17309691	5,51078307	30,36873	14,6215594	213,79	0,35879687	0,41644319	0,7765358
m	0	0,33806525	1,23273107	5,51078307	30,36873	14,252719	203,14	0,34089431	0,43019253	0,80226526

Label	tgrass	tshell	tsand	tchla	chla2	tMGS	mgs2	tsilt	tveryfine	tfine
t	0,775397497	0,100167421	0,775397497	3,767024024	14,19047	13,02681849	169,698	0,32203095	0,493078289	0,683532953
t	0,674490928	0	0,896305399	4,825671974	23,28711	15,56158732	242,163	0,22957298	0,357735013	0,62685893
t	0,611939715	0	0,958856612	3,578369741	12,80473	13,03291986	169,857	0,308037591	0,500925155	0,68170507
t	0,557598827	0	1,0131975	3,56805269	12,731	12,94445827	167,559	0,315669601	0,502906224	0,68893263
t	0,653887062	0	0,916909265	3,436371633	11,80865	13,90877421	193,454	0,285013319	0,443668974	0,669343498
t	0,735314453	0,225513406	0,775397497	3,359677068	11,28743	13,94058822	194,34	0,26991544	0,46046273	0,647656953
t	0,735314453	0,225513406	0,775397497	3,359677068	11,28743	13,94058822	194,34	0,26991544	0,46046273	0,647656953
t	0,735314453	0,225513406	0,775397497	3,359677068	11,28743	13,94058822	194,34	0,26991544	0,46046273	0,647656953

t	0,500179609	0,100167421	1,058823639	3,273415647	10,71525	15,50457997	240,392	0,270064763	0,362525199	0,60658635
t	0,267763327	0,225513406	1,217054721	3,656015591	13,36645	15,36479743	236,077	0,263025967	0,354371832	0,628738214
t	0,368862984	0,201357921	1,145807544	3,35346239	11,24571	14,38002086	206,785	0,210947787	0,36910957	0,75120171
t	0,611939715	0,511972688	0,715167456	4,255441223	18,10878	13,60209543	185,017	0,238274672	0,424567205	0,785410463
t	0,694904938	0,100167421	0,855628871	3,494167998	12,20921	13,91517158	193,632	0,22433077	0,393640332	0,786405264
t	0,611939715	0,225513406	0,896305399	3,320293662	11,02435	14,07437388	198,088	0,210530736	0,384206451	0,779887952
t	0,633051836	0,304692654	0,845543105	3,923015422	15,39005	14,0644232	197,808	0,214588027	0,396205226	0,765303154
t	0,500179609	0,304692654	0,96953211	3,481710786	12,12231	13,67051572	186,883	0,325568984	0,404777964	0,725585409
t	0,500179609	0,304692654	0,96953211	3,481710786	12,12231	13,67051572	186,883	0,325568984	0,404777964	0,725585409
t	0,500179609	0,304692654	0,96953211	3,481710786	12,12231	13,67051572	186,883	0,325568984	0,404777964	0,725585409
t	0	0,411516846	1,159279481	3,215621246	10,34022	14,47262934	209,457	0,197292891	0,369607535	0,74528565
t	0,100167421	0,338065255	1,217054721	3,232239162	10,44737	14,0338163	196,948	0,218078954	0,398277963	0,763151324
t	0,546400564	0,267763327	0,958856612	4,371579806	19,11071	14,9504515	223,516	0,246790008	0,382836151	0,650877797
t	0,79539883	0,100167421	0,765392826	3,249493807	10,55921	13,29454775	176,745	0,259376313	0,497837979	0,69119045
t	0,225513406	0,174083011	1,284039775	3,996104353	15,96885	16,34398972	267,126	0,220121351	0,331652475	0,589720078
t	0,546400564	0	1,024395763	4,535778875	20,57329	15,6505591	244,94	0,26279651	0,349519599	0,608646194
t	0,397699415	0	1,173096912	3,440982418	11,84036	15,08280478	227,491	0,273979683	0,376903189	0,628491799
t	0,424988783	0,100167421	1,119769515	3,14698157	9,903493	14,82902559	219,9	0,251518419	0,405553655	0,637375428
t	0,424988783	0,100167421	1,119769515	3,14698157	9,903493	14,82902559	219,9	0,251518419	0,405553655	0,637375428
t	0,424988783	0,100167421	1,119769515	3,14698157	9,903493	14,82902559	219,9	0,251518419	0,405553655	0,637375428
t	0,79539883	0,174083011	0,755380134	3,481710786	12,12231	12,91712042	166,852	0,280322801	0,519725524	0,697011756
t	1,047197551	0	0,523598776	3,47378468	12,06718	13,17736696	173,643	0,243680849	0,512279948	0,701175339
t	0,633051836	0,100167421	0,927295218	3,591645862	12,89992	13,45607669	181,066	0,273469492	0,480576181	0,682513254
t	1,058823639	0,201357921	0,463647609	2,85297599	8,139472	14,00871158	196,244	0,228086774	0,449570383	0,687606486
t	0,338065255	0	1,232731072	3,1879335	10,16292	13,60275707	185,035	0,274295022	0,472168197	0,67069492
t	0,424988783	0,100167421	1,132647296	3,009051346	9,05439	14,33799149	205,578	0,244473371	0,448377748	0,635458877
t	0	0	1,570796327	3,323141586	11,04327	14,01199486	196,336	0,340169272	0,426484824	0,626562106
t	0,765392826	0,100167421	0,79539883	3,230958062	10,43909	13,53380952	183,164	0,287489797	0,480899581	0,653934907
t	0,765392826	0,100167421	0,79539883	3,230958062	10,43909	13,53380952	183,164	0,287489797	0,480899581	0,653934907

t	0,765392826	0,100167421	0,79539883	3,230958062	10,43909	13,53380952	183,164	0,287489797	0,480899581	0,653934907
t	0,79539883	0	0,775397497	3,448757747	11,89393	13,61800279	185,45	0,231977834	0,476243579	0,700564329
t	0,643501109	0,286756552	0,845543105	2,920717549	8,530591	14,21165015	201,971	0,208274774	0,445702256	0,677883029
t	1,058823639	0	0,511972688	3,569855739	12,74387	13,44581719	180,79	0,230732671	0,482562449	0,720099301
t	1,058823639	0	0,511972688	2,62001145	6,86446	13,32719025	177,614	0,20318787	0,487384039	0,75400915
t	0,674490928	0	0,896305399	3,598878992	12,95193	12,81265	164,164	0,250849699	0,527567666	0,739188911
t	0,916909265	0	0,653887062	3,2414796	10,50719	13,23438703	175,149	0,228826358	0,497531647	0,734544534
t	0,368862984	0	1,201933343	1,894772546	3,590163	13,46406328	181,281	0,182546417	0,486610891	0,74277797
t	0,93774449	0	0,633051836	3,712779282	13,78473	12,58399777	158,357	0,245441004	0,54395139	0,771349515
t	0,93774449	0	0,633051836	3,712779282	13,78473	12,58399777	158,357	0,245441004	0,54395139	0,771349515
t	0,93774449	0	0,633051836	3,712779282	13,78473	12,58399777	158,357	0,245441004	0,54395139	0,771349515
t	0	0,174083011	1,396713316	3,003413724	9,020494	13,18548444	173,857	0,248798863	0,489543114	0,739808922
t	0,775397497	0	0,79539883	2,877493527	8,279969	13,11502955	172,004	0,20526777	0,508673	0,752551443
t	0,353741606	0,100167421	1,201933343	2,821066642	7,958417	13,54905901	183,577	0,211649588	0,466225511	0,744784317
t	0,338065255	0,100167421	1,217054721	3,116701622	9,713829	13,57759183	184,351	0,163939112	0,461893924	0,777101583
t	0,805403501	0	0,765392826	2,904183878	8,434284	13,26472766	175,953	0,186631216	0,496010579	0,761793697
t	0,735314453	0	0,835481874	2,537608323	6,439456	13,6337449	185,879	0,175136918	0,466686452	0,749384432
t	0	0	1,570796327	2,575019806	6,630727	13,29232861	176,686	0,207564031	0,490772238	0,750521288
t	0	0,174083011	1,396713316	2,68989944	7,235559	13,11987043	172,131	0,232499627	0,49821828	0,748793575
t	0	0,174083011	1,396713316	2,68989944	7,235559	13,11987043	172,131	0,232499627	0,49821828	0,748793575
t	0	0,174083011	1,396713316	2,68989944	7,235559	13,11987043	172,131	0,232499627	0,49821828	0,748793575
t	0,601264217	0,174083011	0,93774449	2,961722641	8,771801	13,82577303	191,152	0,189238947	0,442294582	0,750953927
t	0,523598776	0,174083011	1,0131975	3,113639992	9,694754	13,66250343	186,664	0,177514426	0,450265713	0,774119107
t	0,958856612	0	0,611939715	2,967423967	8,805605	13,15268034	172,993	0,179412158	0,498857652	0,787190767
t	0,601264217	0,201357921	0,927295218	3,072397923	9,439629	13,78071116	189,908	0,171697479	0,441304707	0,772809133
t	0,694904938	0,174083011	0,845543105	3,485324949	12,14749	13,23219559	175,091	0,189232397	0,487155262	0,78754987
t	0,835481874	0,100167421	0,725253222	3,030594661	9,184504	13,45213738	180,96	0,172022769	0,473683866	0,774909994
t	0,815416193	0,201357921	0,715167456	2,612162514	6,823393	13,98631474	195,617	0,158696703	0,437025218	0,746319089
t	0,463647609	0,100167421	1,094762509	2,038606387	4,155916	13,36899398	178,73	0,175786693	0,482558435	0,77243291

t	0,463647609	0,100167421	1,094762509	2,038606387	4,155916	13,36899398	178,73	0,175786693	0,482558435	0,77243291
t	0,463647609	0,100167421	1,094762509	2,038606387	4,155916	13,36899398	178,73	0,175786693	0,482558435	0,77243291
t	0,735314453	0,201357921	0,79539883	3,117860645	9,721055	13,25198098	175,615	0,186621728	0,492846814	0,77339211
t	0,601264217	0,225513406	0,916909265	2,863069856	8,197169	13,09713709	171,535	0,231081772	0,498419197	0,75955656
t	0,411516846	0	1,159279481	3,052873892	9,320039	14,01035331	196,29	0,172185281	0,422072604	0,760984364
t	0,601264217	0	0,96953211	3,313288397	10,97788	13,8192981	190,973	0,153183766	0,401048697	0,831490819
t	0,568675503	0	1,002120823	3,144321071	9,886755	13,45511055	181,04	0,204868682	0,460676094	0,77985035
t	0,694904938	0,100167421	0,855628871	2,416570711	5,839814	13,79623137	190,336	0,157422127	0,432217257	0,791360905
t	0,267763327	0,174083011	1,266103673	3,059790516	9,362318	13,88286714	192,734	0,174103439	0,432950963	0,765204074
t	0,694904938	0,100167421	0,855628871	2,972763529	8,837323	13,42441805	180,215	0,180107476	0,466490065	0,794607284
t	0,694904938	0,100167421	0,855628871	2,972763529	8,837323	13,42441805	180,215	0,180107476	0,466490065	0,794607284
t	0,694904938	0,100167421	0,855628871	2,972763529	8,837323	13,42441805	180,215	0,180107476	0,466490065	0,794607284
t	0,523598776	0	1,047197551	2,870298765	8,238615	13,97472719	195,293	0,166823057	0,382898194	0,823124951
t	0,476033818	0	1,094762509	2,817308112	7,937225	13,63363488	185,876	0,157834047	0,417903391	0,841975824
t	0,523598776	0	1,047197551	3,190531304	10,17949	13,65390054	186,429	0,178395687	0,409572149	0,839051069
t	0,694904938	0	0,875891389	3,856183346	14,87015	13,54197179	183,385	0,19461093	0,414665693	0,845044431
t	0,353741606	0,201357921	1,132647296	2,561120458	6,559338	13,91599799	193,655	0,177955995	0,411106031	0,789933426
t	0,523598776	0	1,047197551	3,026803925	9,161542	13,65034798	186,332	0,182055696	0,433205477	0,803762592
t	0,557598827	0,100167421	1,002120823	2,976348602	8,858651	13,81365267	190,817	0,165527087	0,394147108	0,837024748
t	0,535070807	0,141897055	1,002120823	3,157944268	9,972612	13,66623577	186,766	0,18208574	0,400394011	0,848379683
t	0,535070807	0,141897055	1,002120823	3,157944268	9,972612	13,66623577	186,766	0,18208574	0,400394011	0,848379683
t	0,535070807	0,141897055	1,002120823	3,157944268	9,972612	13,66623577	186,766	0,18208574	0,400394011	0,848379683
t	0,523598776	0,201357921	1,002120823	3,485874352	12,15132	13,91786622	193,707	0,187614365	0,406633439	0,789157799
t	0,611939715	0,201357921	0,916909265	3,181188457	10,11996	13,85756111	192,032	0,181580769	0,387413267	0,830958284
t	0,674490928	0	0,896305399	3,600758253	12,96546	13,46692244	181,358	0,212887077	0,419847306	0,840673385
t	0,755380134	0,225513406	0,765392826	3,493787343	12,20655	13,9837048	195,544	0,231965074	0,392121877	0,770939348
t	0,500179609	0,201357921	1,024395763	3,416722113	11,67399	13,72115156	188,27	0,187968822	0,393409913	0,84513699
t	0,338065255	0,100167421	1,217054721	3,488796354	12,1717	13,83997832	191,545	0,190806088	0,415618109	0,789138698
t	0,684719203	0,100167421	0,875891389	3,555840266	12,644	13,71313239	188,05	0,203190274	0,420672954	0,799170005

t	0,568675503	0,267763327	0,927295218	3,485874352	12,15132	13,8878004	192,871	0,242972829	0,396537539	0,774576419
t	0,568675503	0,267763327	0,927295218	3,485874352	12,15132	13,8878004	192,871	0,242972829	0,396537539	0,774576419
t	0,568675503	0,267763327	0,927295218	3,485874352	12,15132	13,8878004	192,871	0,242972829	0,396537539	0,774576419
t	0,694904938	0,225513406	0,815416193	3,375123701	11,39146	13,93208527	194,103	0,239796688	0,400243552	0,759935459
t	0,476033818	0,304692654	0,980296312	3,211647552	10,31468	14,07430282	198,086	0,21426253	0,389593122	0,769664367
t	0	0,286756552	1,284039775	2,860990038	8,185264	13,65902632	186,569	0,193122929	0,434690725	0,78834668
t	0,476033818	0,353741606	0,958856612	3,772650262	14,23289	14,27984594	203,914	0,267608258	0,37222299	0,726479404
t	0	0,201357921	1,369438406	3,470144089	12,0419	13,63444168	185,898	0,218583946	0,429270147	0,784408363
t	0	0,397699415	1,173096912	3,52825736	12,4486	13,47505102	181,577	0,208541094	0,444821115	0,800148603
t	0,643501109	0,304692654	0,835481874	3,183939384	10,13747	14,31813535	205,009	0,222864873	0,375350104	0,746608265
t	0,643501109	0,304692654	0,835481874	3,183939384	10,13747	14,31813535	205,009	0,222864873	0,375350104	0,746608265
t	0,643501109	0,304692654	0,835481874	3,183939384	10,13747	14,31813535	205,009	0,222864873	0,375350104	0,746608265
t	0,805403501	0,247467063	0,705052837	3,805457923	14,48151	13,92034482	193,776	0,253431915	0,41082151	0,741379122
t	0,451026812	0,476033818	0,886077124	3,216198066	10,34393	14,89355565	221,818	0,234620204	0,335547835	0,706888551
t	0,557598827	0,267763327	0,93774449	3,177247236	10,0949	14,96429083	223,93	0,199892471	0,333946454	0,717488872
t	0,601264217	0,286756552	0,886077124	3,454039664	11,93039	14,5196763	210,821	0,233664307	0,362886797	0,725874969
t	0,590500015	0	0,980296312	4,138161427	17,12438	14,07664733	198,152	0,239362902	0,383628403	0,763251222
t	0,725253222	0,225513406	0,79539883	3,098595327	9,601293	14,81978407	219,626	0,231822945	0,336583721	0,715418116
t	0,653887062	0,488205263	0,694904938	3,371151139	11,36466	14,13173733	199,706	0,243432321	0,39415571	0,737524851
t	0,674490928	0,267763327	0,825440953	3,358054496	11,27653	13,81466612	190,845	0,231822632	0,407754975	0,779735942
t	0,674490928	0,267763327	0,825440953	3,358054496	11,27653	13,81466612	190,845	0,231822632	0,407754975	0,779735942
t	0,674490928	0,267763327	0,825440953	3,358054496	11,27653	13,81466612	190,845	0,231822632	0,407754975	0,779735942
t	0,96953211	0,100167421	0,590500015	3,833234926	14,69369	14,23587019	202,66	0,225882232	0,380436432	0,751265559
t	0,463647609	0,304692654	1,002120823	3,656015591	13,36645	14,24387588	202,888	0,207668913	0,37476952	0,768530464
t	0,286756552	0	1,284039775	3,281127855	10,7658	14,49141125	210,001	0,176084962	0,358636252	0,766475347
t	0,397699415	0,174083011	1,132647296	4,210843146	17,7312	14,47007256	209,383	0,191976146	0,360998984	0,759960391
t	0,463647609	0	1,107148718	2,692905123	7,251738	14,77619708	218,336	0,151692068	0,346173609	0,751016675
t	0,411516846	0,286756552	1,058823639	3,305988506	10,92956	14,3572978	206,132	0,181596392	0,374567509	0,765987989
t	0,633051836	0,267763327	0,86574349	2,847835845	8,110169	14,45786983	209,03	0,17138695	0,372180187	0,75753234

t	0,397699415	0	1,173096912	2,952841852	8,719275	14,37696074	206,697	0,167820097	0,374068126	0,76958953	
t	0,397699415	0	1,173096912	2,952841852	8,719275	14,37696074	206,697	0,167820097	0,374068126	0,76958953	
t	0,397699415	0	1,173096912	2,952841852	8,719275	14,37696074	206,697	0,167820097	0,374068126	0,76958953	
t	0,511972688	0,174083011	1,024395763	2,795211262	7,813206	14,06801336	197,909	0,190780336	0,403227426	0,770588398	
t	0,546400564	0	1,024395763	3,190531304	10,17949	14,2407865	202,8	0,179967357	0,383355768	0,773572761	
t	0,286756552	0,286756552	1,159279481	2,789932257	7,783722	14,70826298	216,333	0,151357989	0,358734517	0,746738856	
t	0,725253222	0	0,845543105	2,614833264	6,837353	14,49168727	210,009	0,161766394	0,367082675	0,763730382	
t	0,557598827	0,174083011	0,980296312	2,641027073	6,975024	13,93330542	194,137	0,18825602	0,429860817	0,752603355	
t	0,451026812	0,174083011	1,217054721	2,863642785	8,20045	14,17906908	201,046	0,166603277	0,399784378	0,770061359	
t	0,633051836	0	0,805403501	3,496242268	12,22371	13,593675	184,788	0,207938439	0,44241116	0,781499424	
t	0,397699415	0,100167421	0,96953211	3,298122193	10,87761	14,72059781	216,696	0,170607415	0,376209387	0,719792274	
t	0,397699415	0,100167421	0,96953211	3,298122193	10,87761	14,72059781	216,696	0,170607415	0,376209387	0,719792274	
t	0,397699415	0,100167421	0,96953211	3,298122193	10,87761	14,72059781	216,696	0,170607415	0,376209387	0,719792274	
t	0,511972688	0	1,058823639	2,656142504	7,055093	14,63915981	214,305	0,158454511	0,385230697	0,725054884	
t	0,694904938	0,100167421	0,86574349	3,032976261	9,198945	14,07096301	197,992	0,173197224	0,425362563	0,745674889	
t	0,916909265	0	0,653887062	2,952841852	8,719275	13,54396545	183,439	0,184285586	0,463250741	0,76945056	
t	0,523598776	0	1,047197551	2,649930565	7,022132	13,61447759	185,354	0,173935665	0,459153778	0,770352193	
t	0,568675503	0,100167421	0,991156586	2,546120382	6,482729	14,07277513	198,043	0,202020498	0,432058704	0,720516532	
t	0	0	1,570796327	3,159852686	9,984669	14,5798834	212,573	0,171889128	0,388580558	0,722719734	
t	0,451026812	0	1,119769515	3,091874512	9,559688	13,77483212	189,746	0,165065231	0,443932138	0,771091211	
t	0,557598827	0,100167421	1,002120823	2,614136186	6,833708	14,01128117	196,316	0,165758073	0,434520397	0,743489912	
t	0,557598827	0,100167421	1,002120823	2,614136186	6,833708	14,01128117	196,316	0,165758073	0,434520397	0,743489912	
t	0,557598827	0,100167421	1,002120823	2,614136186	6,833708	14,01128117	196,316	0,165758073	0,434520397	0,743489912	
t	0,411516846	0	1,159279481	2,69932825	7,286373	14,12511947	199,519	0,166832248	0,416718573	0,752105568	
t	0,100167421	0,201357921	1,345282921	2,03835571	4,154894	15,22271329	231,731	0,183730251	0,371017984	0,665844626	
t	0,397699415	0	1,173096912	2,598193603	6,75061	14,05894733	197,654	0,178965236	0,438423313	0,725973062	
t	0,590500015	0,225513406	0,927295218	2,09029352	4,369327	14,04944839	197,387	0,195143904	0,436853732	0,721062584	
t	0,500179609	0,100167421	1,058823639	3,371376573	11,36618	13,58992274	184,686	0,18593081	0,46709174	0,753906246	
t	0,611939715	0	0,958856612	2,867420618	8,222101	14,33907249	205,609	0,15855569	0,418990028	0,724343851	

t	0,684719203	0,100167421	0,875891389	4,291484592	18,41684	14,22947645	202,478	0,181878314	0,416093291	0,731076161
t	0,500179609	0,174083011	1,03572552	2,68633505	7,216396	14,03919513	197,099	0,234943741	0,425793921	0,713782243
t	0,500179609	0,174083011	1,03572552	2,68633505	7,216396	14,03919513	197,099	0,234943741	0,425793921	0,713782243
t	0,500179609	0,174083011	1,03572552	2,68633505	7,216396	14,03919513	197,099	0,234943741	0,425793921	0,713782243
t	0,451026812	0,174083011	1,082591063	2,60365858	6,779038	14,11003189	199,093	0,19512164	0,424416527	0,730640774
t	0,353741606	0,100167421	1,201933343	2,744541127	7,532506	14,06531194	197,833	0,173167738	0,428458098	0,74349513
t	0,568675503	0,201357921	0,958856612	2,457735136	6,040462	13,59227722	184,75	0,202374509	0,46140069	0,752551242
t	0,633051836	0,201357921	0,896305399	2,244850106	5,039352	13,31307628	177,238	0,181800386	0,48828287	0,771818894
t	0,397699415	0	1,173096912	3,355882894	11,26195	12,98160237	168,522	0,190388783	0,514792021	0,786352564
t	0,368862984	0	1,201933343	3,015249741	9,091731	13,26894871	176,065	0,19655751	0,494343793	0,7587853
t	0,611939715	0	0,958856612	2,777585822	7,714983	13,23336692	175,122	0,216983946	0,488733746	0,761810716
t	0,735314453	0,100167421	0,825440953	3,070736557	9,429423	13,59536686	184,834	0,175881332	0,469800992	0,753536805
t	0,735314453	0,100167421	0,825440953	3,070736557	9,429423	13,59536686	184,834	0,175881332	0,469800992	0,753536805
t	0,735314453	0,100167421	0,825440953	3,070736557	9,429423	13,59536686	184,834	0,175881332	0,469800992	0,753536805
t	0,715167456	0,174083011	0,825440953	2,734860143	7,47946	13,22459829	174,89	0,198161468	0,496110318	0,762294543
t	0,557598827	0,174083011	0,980296312	3,290074467	10,82459	13,29074866	176,644	0,183788364	0,492060948	0,765837174
t	0,568675503	0,174083011	0,96953211	3,002963203	9,017788	13,47827882	181,664	0,1913985	0,474879688	0,756049113
t	0,557598827	0,100167421	1,002120823	3,552651123	12,62133	13,35597245	178,382	0,20633304	0,483674934	0,753165142
t	0,304692654	0	1,266103673	2,630640416	6,920269	13,97132778	195,198	0,215482124	0,455426804	0,692029773
t	0,451026812	0,100167421	1,107148718	2,902853596	8,426559	13,74456256	188,913	0,191391806	0,457245949	0,739105254
t	0,304692654	0,100167421	1,249045772	2,949576241	8,7	13,36817115	178,708	0,204267224	0,4888078	0,741557008
t	0,633051836	0,100167421	0,927295218	4,036280466	16,29156	12,98714749	168,666	0,249549049	0,514453246	0,72460257
t	0,633051836	0,100167421	0,927295218	4,036280466	16,29156	12,98714749	168,666	0,249549049	0,514453246	0,72460257
t	0,633051836	0,100167421	0,927295218	4,036280466	16,29156	12,98714749	168,666	0,249549049	0,514453246	0,72460257
t	0,601264217	0	0,96953211	3,625745441	13,14603	13,26642378	175,998	0,270828986	0,487350688	0,701787643
t	0,735314453	0,201357921	0,79539883	3,824975817	14,63044	12,90166656	166,453	0,281500602	0,519657665	0,692635129
t	0,304692654	0,225513406	1,187299323	2,758229686	7,607831	14,57230249	212,352	0,207152719	0,422295465	0,668048174
t	0,653887062	0	0,916909265	3,866235896	14,94778	12,74754878	162,5	0,338842412	0,509894931	0,671045373
t	0	0,174083011	1,396713316	3,398879227	11,55238	15,50116125	240,286	0,351285637	0,35040804	0,562221618

t	0,643501109	0	0,927295218	3,439071096	11,82721	12,99869224	168,966	0,257846122	0,51793178	0,703856393
t	0,96953211	0,225513406	0,546400564	4,271739692	18,24776	12,86468033	165,5	0,293718411	0,514941818	0,702261313
t	0,557598827	0	1,0131975	3,995439901	15,96354	13,52124255	182,824	0,326563411	0,470746057	0,630063705
t	0,557598827	0	1,0131975	3,995439901	15,96354	13,52124255	182,824	0,326563411	0,470746057	0,630063705
t	0,557598827	0	1,0131975	3,995439901	15,96354	13,52124255	182,824	0,326563411	0,470746057	0,630063705
t	0,601264217	0,286756552	0,886077124	4,093375136	16,75572	14,54912368	211,677	0,274833873	0,413812393	0,641533203
t	0	0,225513406	1,345282921	3,352686982	11,24051	16,41840431	269,564	0,305980334	0,320905418	0,547007523
t	0	0,174083011	1,396713316	3,198917004	10,23307	14,45108992	208,834	0,369752382	0,395707684	0,591447769
t	0	0	1,570796327	3,492131154	12,19498	14,32773534	205,284	0,462872533	0,372271986	0,526010598
t	0	0	1,570796327	3,523169028	12,41272	14,53282491	211,203	0,270651067	0,412846746	0,648316815
t	0	0	1,570796327	3,205133383	10,27288	16,10499922	259,371	0,22710081	0,321071706	0,609385947
t	0	0	1,570796327	3,423904204	11,72312	15,52617145	241,062	0,380984008	0,348736719	0,537551297
t	0	0	1,570796327	3,91248131	15,30751	15,12954064	228,903	0,282423733	0,371240041	0,624132698
t	0	0	1,570796327	3,91248131	15,30751	15,12954064	228,903	0,282423733	0,371240041	0,624132698
t	0	0	1,570796327	3,91248131	15,30751	15,12954064	228,903	0,282423733	0,371240041	0,624132698
t	0	0	1,570796327	3,404558709	11,59102	15,73969504	247,738	0,243615583	0,371240337	0,595978577
t	0	0,100167421	1,470628906	3,726405775	13,8861	14,90681052	222,213	0,265399336	0,395690111	0,632123204
t	0	0	1,570796327	3,563366386	12,69758	14,74920337	217,539	0,272941167	0,400298029	0,638008972
t	0,715167456	0	0,855628871	3,198917004	10,23307	13,31307628	177,238	0,181800386	0,567713447	0,676694761
t	0	0,201357921	1,369438406	4,128801279	17,047	12,98160237	168,522	0,190388783	0,392702604	0,663507018
t	0,546400564	0	1,024395763	4,280428717	18,32207	13,26894871	176,065	0,19655751	0,585111689	0,685318846
t	0,815416193	0	0,755380134	3,355882894	11,26195	13,23336692	175,122	0,216983946	0,513757407	0,632265113
t	0,451026812	0,100167421	1,107148718	4,374286227	19,13438	13,59536686	184,834	0,175881332	0,554891652	0,679478226
t	0,500179609	0,286756552	0,980296312	4,159944711	17,30514	13,22459829	174,89	0,198161468	0,445105275	0,632863868
t	0,500179609	0,286756552	0,980296312	4,159944711	17,30514	13,22459829	174,89	0,198161468	0,445105275	0,632863868
t	0,500179609	0,286756552	0,980296312	4,159944711	17,30514	13,22459829	174,89	0,198161468	0,445105275	0,632863868
t	0	0,201357921	1,369438406	1,265585635	1,601707	13,29074866	176,644	0,183788364	0,388097474	0,621983507
t	0	0,100167421	1,470628906	3,833433187	14,69521	13,47827882	181,664	0,1913985	0,340465957	0,634647786
t	0	0	1,570796327	3,451321486	11,91162	13,35597245	178,382	0,20633304	0,326987746	0,622776482

t	0,93774449	0	0,633051836	4,333354359	18,77796	13,97132778	195,198	0,215482124	0,513838944	0,682044827
t	0	0	1,570796327	3,388307837	11,48063	13,74456256	188,913	0,191391806	0,332760978	0,639830793
t	0	0,174083011	1,396713316	3,828144459	14,65469	13,36817115	178,708	0,204267224	0,407393489	0,649740334
t	0	0,201357921	1,369438406	4,028328437	16,22743	12,98714749	168,666	0,249549049	0,405922879	0,631355222
t	0,643501109	0,174083011	0,896305399	2,834861725	8,036441	13,26642378	175,998	0,270828986	0,413896045	0,623783029
t	0,643501109	0,174083011	0,896305399	2,834861725	8,036441	13,26642378	175,998	0,270828986	0,413896045	0,623783029
t	0,643501109	0,174083011	0,896305399	2,834861725	8,036441	13,26642378	175,998	0,270828986	0,413896045	0,623783029
t	0,201357921	0,201357921	1,284039775	3,427181057	11,74557	12,90166656	166,453	0,281500602	0,48681154	0,680154753
t	0,755380134	0	0,815416193	2,994118067	8,964743	14,57230249	212,352	0,207152719	0,518694655	0,693915907
t	0,735314453	0	0,835481874	3,553153529	12,6249	12,74754878	162,5	0,338842412	0,509924508	0,688978888
t	0,267763327	0,100167421	1,284039775	3,789253225	14,35844	15,50116125	240,286	0,351285637	0,517022423	0,767308317
t	0,633051836	0,100167421	0,927295218	3,319415611	11,01852	12,99869224	168,966	0,257846122	0,483220569	0,693599406
t	0,855628871	0,100167421	0,705052837	3,240182094	10,49878	12,86468033	165,5	0,293718411	0,50682279	0,702321239
t	0,755380134	0,201357921	0,775397497	3,57998324	12,81628	13,52124255	182,824	0,326563411	0,512614818	0,706906573
t	0,286756552	0	1,284039775	3,677514106	13,52411	14,54912368	211,677	0,274833873	0,508139744	0,72246533
t	0,286756552	0	1,284039775	3,677514106	13,52411	14,54912368	211,677	0,274833873	0,508139744	0,72246533
t	0,286756552	0	1,284039775	3,677514106	13,52411	14,54912368	211,677	0,274833873	0,508139744	0,72246533
t	0,611939715	0	0,958856612	3,616477568	13,07891	16,41840431	269,564	0,305980334	0,497373666	0,760219523
t	0,174083011	0,267763327	1,249045772	3,433188023	11,78678	14,45108992	208,834	0,369752382	0,469330138	0,7504036
t	0,411516846	0	1,159279481	3,434763456	11,7976	15,12954064	228,903	0,282423733	0,500409714	0,76297745
t	0,267763327	0,174083011	1,249045772	2,746709668	7,544414	14,53282491	211,203	0,270651067	0,387057216	0,686475168
t	0,225513406	0,225513406	1,249045772	3,062000653	9,375848	13,09144759	171,386	0,223634793	0,508000698	0,764726075
t	0,601264217	0	0,96953211	2,948300527	8,692476	16,10499922	259,371	0,22710081	0,48577299	0,740997933
t	0,568675503	0	1,002120823	1,304769711	1,702424	15,52617145	241,062	0,380984008	0,480487013	0,765672547
t	0,601264217	0,304692654	0,875891389	3,020575938	9,123879	15,73969504	247,738	0,243615583	0,496480133	0,758379817
t	0,601264217	0,304692654	0,875891389	3,020575938	9,123879	15,73969504	247,738	0,243615583	0,496480133	0,758379817
t	0,601264217	0,304692654	0,875891389	3,020575938	9,123879	15,73969504	247,738	0,243615583	0,496480133	0,758379817
t	0,286756552	0,286756552	1,159279481	3,231821468	10,44467	14,90681052	222,213	0,265399336	0,430885965	0,730425378
t	0,368862984	0,100167421	1,159279481	3,67786351	13,52668	14,74920337	217,539	0,272941167	0,409754991	0,726194015

t	0,463647609	0,174083011	1,070616718	3,194526569	10,205	13,69532767	187,562	0,216889201	0,454959055	0,737233408
t	0,353741606	0,286756552	1,107148718	3,93491296	15,48354	14,32885201	205,316	0,172307483	0,411438009	0,726663476
t	0,476033818	0,286756552	1,002120823	3,623374118	13,12884	14,31537635	204,93	0,18270583	0,410815774	0,727226217
t	0,286756552	0,100167421	1,266103673	3,174696836	10,0787	13,89697809	193,126	0,206243358	0,441367754	0,732771549
t	0,424988783	0	1,145807544	3,071100129	9,431656	14,00514191	196,144	0,18980765	0,442185092	0,724008742
t	0,368862984	0,353741606	1,047197551	3,273415647	10,71525	14,07089194	197,99	0,185449413	0,435231365	0,726695195
t	0,368862984	0,353741606	1,047197551	3,273415647	10,71525	14,07089194	197,99	0,185449413	0,435231365	0,726695195
t	0,368862984	0,353741606	1,047197551	3,273415647	10,71525	14,07089194	197,99	0,185449413	0,435231365	0,726695195
t	0,424988783	0	1,145807544	3,401833329	11,57247	14,03207041	196,899	0,220220495	0,436926636	0,705799467
t	0,476033818	0,201357921	1,047197551	3,414352062	11,6578	14,03050249	196,855	0,201357895	0,437039822	0,717080489
t	0,353741606	0,353741606	1,058823639	3,114049614	9,697305	14,12331406	199,468	0,211743611	0,421558322	0,721352873
t	0,500179609	0	1,070616718	2,617552483	6,851581	14,38109871	206,816	0,163080984	0,393967848	0,746596028
t	0,411516846	0,100167421	1,145807544	3,126705774	9,776289	14,19436508	201,48	0,181336658	0,423297917	0,725726717
t	0,590500015	0	0,96953211	2,748500318	7,554254	14,44600291	208,687	0,168593823	0,411559261	0,714409612
t	0,523598776	0,100167421	1,03572552	2,381760063	5,672781	13,9458596	194,487	0,174388053	0,440028118	0,743555241
t	0,546400564	0	1,024395763	3,995976727	15,96783	14,25149115	203,105	0,160452732	0,418098412	0,733997778
t	0,546400564	0	1,024395763	3,995976727	15,96783	14,25149115	203,105	0,160452732	0,418098412	0,733997778
t	0,546400564	0	1,024395763	3,995976727	15,96783	14,25149115	203,105	0,160452732	0,418098412	0,733997778
t	0,424988783	0	1,145807544	3,41862253	11,68698	14,11176814	199,142	0,170656985	0,41275953	0,759689537
t	0,694904938	0	0,875891389	2,915489839	8,500081	14,19355488	201,457	0,173477118	0,404080936	0,756499977
t	0,353741606	0	1,217054721	2,550625414	6,50569	15,03825123	226,149	0,137055579	0,357672201	0,713532073
t	0,286756552	0,174083011	1,232731072	2,270138102	5,153527	14,60239706	213,23	0,149637144	0,37458234	0,746033009
t	0,286756552	0,225513406	1,201933343	2,830179853	8,009918	15,5199549	240,869	0,140294713	0,3421009	0,676555068
t	0,424988783	0	1,145807544	3,554702519	12,63591	14,68587757	215,675	0,168538076	0,377304526	0,723222246
t	0,633051836	0	0,93774449	2,810401573	7,898357	14,53292813	211,206	0,165017873	0,384935547	0,735394152
t	0,463647609	0	1,107148718	2,885944386	8,328675	14,12575662	199,537	0,154437267	0,424876295	0,744401844
t	0,463647609	0	1,107148718	2,885944386	8,328675	14,12575662	199,537	0,154437267	0,424876295	0,744401844
t	0,463647609	0	1,107148718	2,885944386	8,328675	14,12575662	199,537	0,154437267	0,424876295	0,744401844
t	0,100167421	0,174083011	1,369438406	2,805785452	7,872432	14,80503293	219,189	0,160052378	0,349496909	0,740859386

t	0,286756552	0	1,284039775	2,593138832	6,724369	14,39683993	207,269	0,201397772	0,391692549	0,729826523
t	0,500179609	0,174083011	1,03572552	2,765198365	7,646322	14,87954972	221,401	0,146764025	0,34897004	0,737166297
t	0,511972688	0,174083011	1,024395763	3,079448002	9,483	14,46336752	209,189	0,210369569	0,363485898	0,748455962
t	0,546400564	0	1,024395763	2,848861176	8,11601	14,20274621	201,718	0,174511406	0,398981163	0,762638304
t	0,476033818	0,201357921	1,047197551	2,834861725	8,036441	14,87101207	221,147	0,16496121	0,355338815	0,725397663
t	0,397699415	0,286756552	1,070616718	2,959505026	8,75867	14,89496559	221,86	0,178015593	0,345361027	0,726201668
t	0,338065255	0,286756552	1,119769515	3,102083655	9,622923	14,48098754	209,699	0,199819647	0,378376	0,735401187
t	0,338065255	0,286756552	1,119769515	3,102083655	9,622923	14,48098754	209,699	0,199819647	0,378376	0,735401187
t	0,338065255	0,286756552	1,119769515	3,102083655	9,622923	14,48098754	209,699	0,199819647	0,378376	0,735401187
t	0	0,225513406	1,345282921	2,781868976	7,738795	15,36951528	236,222	0,162543567	0,32382068	0,697179363
t	0,590500015	0,100167421	0,96953211	3,265054364	10,66058	14,7154001	216,543	0,191244927	0,347029472	0,740380667
t	0,500179609	0,174083011	1,03572552	3,036659349	9,2213	14,62108067	213,776	0,190574708	0,36153521	0,739167118
t	0,424988783	0,523598776	0,86574349	3,288820457	10,81634	14,97220759	224,167	0,215305275	0,35073342	0,694027467
t	0,353741606	0,225513406	1,145807544	3,017940689	9,107966	14,62815778	213,983	0,209134236	0,357602213	0,732653695
t	0,500179609	0,201357921	1,024395763	2,552096001	6,513194	14,08019886	198,252	0,191708742	0,397339836	0,774214331
t	0,725253222	0,201357921	0,805403501	3,456517033	11,94751	14,92115947	222,641	0,1988919	0,329348706	0,726858415
t	0,411516846	0,225513406	1,094762509	3,219434733	10,36476	15,04423478	226,329	0,172722857	0,332937789	0,72086313
t	0,411516846	0,225513406	1,094762509	3,219434733	10,36476	15,04423478	226,329	0,172722857	0,332937789	0,72086313
t	0,411516846	0,225513406	1,094762509	3,219434733	10,36476	15,04423478	226,329	0,172722857	0,332937789	0,72086313
t	0,368862984	0,463647609	0,958856612	3,188976325	10,16957	15,09350854	227,814	0,210792981	0,334094166	0,697838521
t	0,643501109	0,338065255	0,815416193	3,139750627	9,858034	14,50068964	210,27	0,217075396	0,374537699	0,725513293
t	0,735314453	0,286756552	0,755380134	3,205916406	10,2779	14,85930685	220,799	0,198803234	0,347778881	0,717636125
t	0	0,201357921	1,369438406	3,763880976	14,1668	14,06385438	197,792	0,237265451	0,386010583	0,760637644
t	0,805403501	0,174083011	0,735314453	3,462785006	11,99088	13,96506355	195,023	0,260346272	0,406571085	0,734601026
t	0,225513406	0,338065255	1,159279481	2,628942563	6,911339	14,30898319	204,747	0,243015669	0,381468156	0,728681008
t	1,0131975	0	0,557598827	3,367408499	11,33944	14,60054793	213,176	0,224920972	0,357498354	0,725885443
t	0	0,267763327	1,303033	4,262303368	18,16723	14,78614216	218,63	0,200280327	0,335624696	0,737624205
t	0	0,267763327	1,303033	4,262303368	18,16723	14,78614216	218,63	0,200280327	0,335624696	0,737624205
t	0	0,267763327	1,303033	4,262303368	18,16723	14,78614216	218,63	0,200280327	0,335624696	0,737624205

t	0	0,100167421	1,470628906	3,834998044	14,70721	13,66982077	186,864	0,250220031	0,415259692	0,778178813
t	0	0,201357921	1,369438406	3,029344153	9,176926	13,78129167	189,924	0,211940536	0,41729794	0,782087139
t	0,141897055	0,174083011	1,345282921	3,905210622	15,25067	14,49703418	210,164	0,22929872	0,367758769	0,725466357
t	0,775397497	0,225513406	0,745355373	3,198917004	10,23307	15,07617325	227,291	0,191625323	0,337556926	0,705468363
t	0,201357921	0,174083011	1,303033	3,195280895	10,20982	14,17762321	201,005	0,242375122	0,38777263	0,737397667
t	0	0	1,570796327	2,985246388	8,911696	14,46098199	209,12	0,228870171	0,367054613	0,731211256
t	0	0,267763327	1,303033	3,901813681	15,22415	14,50568854	210,415	0,267832377	0,363489509	0,70546816
t	0	0,267763327	1,303033	3,901813681	15,22415	14,50568854	210,415	0,267832377	0,363489509	0,70546816
t	0	0,267763327	1,303033	3,901813681	15,22415	14,50568854	210,415	0,267832377	0,363489509	0,70546816
t	0,93774449	0,267763327	0,557598827	4,937327212	24,3772	14,17550705	200,945	0,270031684	0,381508384	0,725126202
t	0,805403501	0,225513406	0,715167456	3,214431521	10,33257	14,67514906	215,36	0,226535476	0,367275032	0,704654783
t	0,286756552	0,353741606	1,107148718	3,346257611	11,19744	14,85513379	220,675	0,215347386	0,358146269	0,699330148
t	0,424988783	0	1,145807544	3,707536918	13,74583	15,08545657	227,571	0,203745962	0,345946554	0,691325538
t	0	0,267763327	1,303033	3,131207115	9,804458	15,04629523	226,391	0,167016115	0,332124966	0,723425208
t	0,397699415	0,476033818	0,927295218	3,602751726	12,97982	14,86048451	220,834	0,210330857	0,350942096	0,708827319
t	0,896305399	0,286756552	0,590500015	0,961981445	0,9254083	14,88250651	221,489	0,217810751	0,34637909	0,70632112
t	0,755380134	0,286756552	0,735314453	3,499429954	12,24601	14,42899858	208,196	0,218532307	0,369485062	0,739170834
t	0,755380134	0,286756552	0,735314453	3,499429954	12,24601	14,42899858	208,196	0,218532307	0,369485062	0,739170834
t	0,755380134	0,286756552	0,735314453	3,499429954	12,24601	14,42899858	208,196	0,218532307	0,369485062	0,739170834
t	0,735314453	0,267763327	0,765392826	2,395088516	5,736449	14,21084093	201,948	0,191498738	0,394844219	0,754805379
t	0,201357921	0,286756552	1,217054721	3,248522741	10,5529	14,77538494	218,312	0,191672251	0,356510063	0,722439122
t	0,424988783	0	1,145807544	2,712926833	7,359972	14,27697447	203,832	0,214231122	0,383343227	0,747976637
t	0,201357921	0,174083011	1,303033	2,58257604	6,669699	14,47522021	209,532	0,19204477	0,375717181	0,740977553
t	0,424988783	0	1,145807544	2,785865934	7,761049	14,9594786	223,786	0,17482262	0,348019599	0,71787854
t	0,601264217	0,201357921	0,927295218	2,474470246	6,123003	14,38294128	206,869	0,202855472	0,379353049	0,743998877
t	0,174083011	0	1,396713316	2,746323178	7,542291	15,1018873	228,067	0,163065098	0,323980266	0,725622172
t	0,523598776	0,100167421	1,03572552	4,268012418	18,21593	14,55362498	211,808	0,193043145	0,36893018	0,739269856
t	0,523598776	0,100167421	1,03572552	4,268012418	18,21593	14,55362498	211,808	0,193043145	0,36893018	0,739269856
t	0,523598776	0,100167421	1,03572552	4,268012418	18,21593	14,55362498	211,808	0,193043145	0,36893018	0,739269856

t	0,476033818	0,100167421	1,082591063	3,492927139	12,20054	15,15166658		229,573	0,154449964	0,334744409	0,715575552
t	0,397699415	0,201357921	1,119769515	2,633135014	6,9334	15,00623204		225,187	0,205297811	0,348001195	0,699339892
t	0,201357921	0,304692654	1,201933343	3,846720681	14,79726	15,10946723		228,296	0,191346313	0,342356178	0,699521977
t	0,353741606	0	1,217054721	2,926037936	8,561698	15,16749815		230,053	0,180465742	0,336333589	0,702558699
t	0,397699415	0	1,173096912	2,844110757	8,088966	14,86990249		221,114	0,168618153	0,364473052	0,715753984
t	0,353741606	0,225513406	1,145807544	3,222924138	10,38724	14,82865469		219,889	0,177871429	0,36669697	0,712708786
t	0,476033818	0,225513406	1,03572552	3,257007829	10,6081	14,82190946		219,689	0,184465555	0,368670855	0,708935079
t	0,286756552	0,201357921	1,217054721	2,844295519	8,090017	15,49725782		240,165	0,173573082	0,327741181	0,678180869
t	0,286756552	0,201357921	1,217054721	2,844295519	8,090017	15,49725782		240,165	0,173573082	0,327741181	0,678180869
t	0,286756552	0,201357921	1,217054721	2,844295519	8,090017	15,49725782		240,165	0,173573082	0,327741181	0,678180869
t	0,174083011	0,174083011	1,323329264	3,173695007	10,07234	15,93882053		254,046	0,166459305	0,313723023	0,650644697
t	0,463647609	0,225513406	1,047197551	2,697829869	7,278286	15,14143322		229,263	0,158435225	0,348901256	0,703152067
t	0,100167421	0,353741606	1,201933343	2,491754201	6,208839	15,23791324		232,194	0,185084886	0,340177856	0,690385788
t	0,174083011	0,100167421	1,369438406	2,720525317	7,401258	15,14493315		229,369	0,169671234	0,348293845	0,699793053
t	0,353741606	0	1,217054721	3,233833638	10,45768	13,27584272		176,248	0,20374904	0,483461515	0,7718344
t	0,225513406	0,267763327	1,217054721	2,574488687	6,627992	15,60961242		243,66	0,155588592	0,311223198	0,685112927
t	0,353741606	0	1,217054721	3,41614988	11,67008	15,41878724		237,739	0,164052292	0,332711418	0,685420371
t	0,353741606	0	1,217054721	3,41614988	11,67008	15,41878724		237,739	0,164052292	0,332711418	0,685420371
t	0,353741606	0	1,217054721	3,41614988	11,67008	15,41878724		237,739	0,164052292	0,332711418	0,685420371
t	0,546400564	0	1,024395763	2,901573366	8,419128	13,39996269		179,559	0,209628395	0,471403802	0,763924363
t	0,267763327	0,267763327	1,187299323	2,692905123	7,251738	13,36297123		178,569	0,184428587	0,48465874	0,76256633
t	0,424988783	0	1,145807544	3,249493807	10,55921	13,29360749		176,72	0,201120213	0,483120424	0,770393011
t	0,353741606	0	1,217054721	2,90947607	8,465051	13,11571576		172,022	0,201753198	0,494919054	0,787782972
t	0,397699415	0,225513406	1,107148718	3,078778816	9,478879	13,4643975		181,29	0,238128178	0,468404212	0,733697886
t	0,338065255	0,100167421	1,217054721	2,570215166	6,606006	13,46978099		181,435	0,185463179	0,473788922	0,762408564
t	0,451026812	0	1,119769515	3,588023133	12,87391	13,0060755		169,158	0,237478048	0,498818138	0,772389996
t	0,174083011	0,267763327	1,249045772	2,830179853	8,009918	13,44325109		180,721	0,190625322	0,48610122	0,737065527
t	0,174083011	0,267763327	1,249045772	2,830179853	8,009918	13,44325109		180,721	0,190625322	0,48610122	0,737065527
t	0,174083011	0,267763327	1,249045772	2,830179853	8,009918	13,44325109		180,721	0,190625322	0,48610122	0,737065527

t	0,338065255	0	1,232731072	3,556461725	12,64842	12,93611224	167,343	0,238441889	0,508925806	0,763853797
t	0,353741606	0	1,217054721	3,648683324	13,31289	12,63273525	159,586	0,302381246	0,517412849	0,745886654
t	0,368862984	0	1,201933343	3,729367239	13,90818	13,15537913	173,064	0,248985572	0,485729029	0,75452024
t	0,451026812	0	1,119769515	4,100112194	16,81092	13,28736242	176,554	0,249000479	0,477967777	0,739560986
t	0,500179609	0	1,070616718	4,526910646	20,49292	13,64100436	186,077	0,270192185	0,453083755	0,698874072
t	0,96953211	0	0,601264217	4,164850538	17,34598	13,09492268	171,477	0,291361701	0,480913358	0,728620721
t	0,568675503	0,353741606	0,875891389	3,473855207	12,06767	12,8491634	165,101	0,365320489	0,476721413	0,686685257
t	0,225513406	0,201357921	1,266103673	3,945559529	15,56744	13,83170272	191,316	0,285803407	0,433215188	0,691201343
t	0,225513406	0,201357921	1,266103673	3,945559529	15,56744	13,83170272	191,316	0,285803407	0,433215188	0,691201343
t	0,225513406	0,201357921	1,266103673	3,945559529	15,56744	13,83170272	191,316	0,285803407	0,433215188	0,691201343
t	0,267763327	0,304692654	1,159279481	3,068364385	9,41486	13,98323997	195,531	0,291690068	0,423793008	0,67510704
t	0,353741606	0,100167421	1,201933343	3,358453513	11,27921	13,95969197	194,873	0,267136732	0,439982318	0,67649587
t	0,397699415	0,225513406	1,107148718	4,406971749	19,4214	12,99892303	168,972	0,298530458	0,499318933	0,698894579
t	0,368862984	0,174083011	1,159279481	3,216763902	10,34757	13,23276237	175,106	0,278027426	0,48475079	0,706768067
t	0,286756552	0,100167421	1,266103673	3,69549726	13,6567	13,75692553	189,253	0,309663921	0,445744802	0,657549978
t	0,653887062	0,225513406	0,86574349	3,488019495	12,16628	13,63469838	185,905	0,259641177	0,461303354	0,695786396
t	0,815416193	0,225513406	0,705052837	5,13636642	26,38226	12,55268895	157,57	0,352375105	0,507697939	0,705779211
t	0,715167456	0	0,855628871	3,701060659	13,69785	12,1290148	147,113	0,34229688	0,555998679	0,716526484
t	0,715167456	0	0,855628871	3,701060659	13,69785	12,1290148	147,113	0,34229688	0,555998679	0,716526484
t	0,715167456	0	0,855628871	3,701060659	13,69785	12,1290148	147,113	0,34229688	0,555998679	0,716526484
t	0,476033818	0	1,094762509	3,593310451	12,91188	12,63408089	159,62	0,310222548	0,520970719	0,720711768
t	0,694904938	0,201357921	0,835481874	4,386766463	19,24372	12,36203867	152,82	0,359454705	0,527777304	0,685974144
t	0,500179609	0,201357921	1,024395763	4,079835781	16,64506	13,2028406	174,315	0,308222837	0,487619148	0,670872875
t	0,500179609	0	1,070616718	6,348996771	40,30976	13,46268918	181,244	0,324993224	0,468932262	0,644164626
t	0	0	1,570796327	3,014457829	9,086956	12,55830403	157,711	0,500722985	0,441723	0,54337458
t	0,500179609	0	1,070616718	4,583699379	21,0103	14,44371836	208,621	0,30412136	0,414499969	0,623470725
t	0,225513406	0,286756552	1,201933343	3,873158917	15,00136	12,95866505	167,927	0,373215332	0,477092415	0,647415014
t	0,845543105	0	0,725253222	3,927846738	15,42798	13,94948028	194,588	0,360556325	0,427427842	0,608305486
t	0,845543105	0	0,725253222	3,927846738	15,42798	13,94948028	194,588	0,360556325	0,427427842	0,608305486

t	0,845543105	0	0,725253222	3,927846738	15,42798	13,94948028	194,588	0,360556325	0,427427842	0,608305486
t	0	0,225513406	1,345282921	4,21750163	17,78732	14,64643301	214,518	0,432098017	0,389866136	0,509834248
t	0	0	1,570796327	3,020575938	9,123879	13,81412321	190,83	0,355003127	0,45349968	0,58848534
t	0	0	1,570796327	4,778462096	22,8337	12,75390136	162,662	0,457748498	0,460876962	0,56062399
t	0	0	1,570796327	5,513096226	30,39423	14,9833908	224,502	0,31336782	0,417646132	0,566227054
t	0,201357921	0	1,369438406	2,777585822	7,714983	11,95746629	142,981	0,288034228	0,605659189	0,715251988
t	0	0	1,570796327	4,128801279	17,047	13,60477857	185,09	0,369661561	0,460759964	0,582556345
t	0	0	1,570796327	3,415259873	11,664	13,38521572	179,164	0,341813957	0,484011515	0,604399618
t	0,590500015	0,174083011	0,948262907	2,929608506	8,582606	12,41921898	154,237	0,274850354	0,568090898	0,697642618
t	0,590500015	0,174083011	0,948262907	2,929608506	8,582606	12,41921898	154,237	0,274850354	0,568090898	0,697642618
t	0,590500015	0,174083011	0,948262907	2,929608506	8,582606	12,41921898	154,237	0,274850354	0,568090898	0,697642618
t	0,875891389	0,100167421	0,684719203	3,749285265	14,05714	11,72467484	137,468	0,356499209	0,595373865	0,697282767
t	0,568675503	0	1,002120823	2,993947561	8,963722	11,9913719	143,793	0,295165911	0,598918449	0,711864577
t	0,338065255	0	1,232731072	2,259927211	5,107271	11,7657129	138,432	0,288121966	0,626300073	0,725468069

MGS	chla2	MGS2	aglmac	alpheu	amaaus	antaur	aontri	aricid	armand	artbif
217,264008	10,2330675	47203,649	0	0	0	1	26	0	0	0
217,264008	10,2330675	47203,649	0	0	0	2	16	0	0	0
217,264008	10,2330675	47203,649	0	0	0	0	9	0	0	0
215,343002	8,45069027	46372,6086	1	0	0	2	73	0	0	0
216,666	7,95841694	46944,1557	0	0	0	0	5	0	0	0
215,492996	12,8370867	46437,2314	0	0	0	0	49	0	0	0
219,419006	4,25931358	48144,7003	0	0	0	1	8	0	0	0
192,162994	2,5148046	36926,6164	0	0	0	0	0	0	0	0
191,690994	2,59924483	36745,4373	0	0	0	0	0	0	0	0
194,102005	2,12283349	37675,5883	1	0	0	0	0	1	0	0
187,751007	2,92234206	35250,4407	1	0	0	0	0	0	0	0
189,794998	3,18274879	36022,1413	1	0	0	0	0	0	0	0
192,451996	3,8268764	37037,7707	1	0	0	0	0	0	0	0
192,203995	3,79277563	36942,3756	1	0	0	0	1	0	0	0
193,475998	3,05384755	37432,9618	1	0	0	0	0	0	0	0
196,526993	2,95785666	38622,8589	1	0	0	0	2	0	0	0
235,113998	1,87793112	55278,5922	1	0	0	0	7	0	0	0
198,673004	4,00469112	39470,9626	0	0	0	0	0	0	0	0
195,25	2,2886951	38122,5625	2	0	0	0	0	0	0	0
197,242996	2,87212992	38904,7996	0	0	0	0	3	0	0	0
197,242996	2,87212992	38904,7996	1	0	0	0	1	0	0	0
197,242996	2,87212992	38904,7996	2	0	0	0	5	0	0	1
219,130997	5,64464283	48018,3937	0	0	0	0	41	0	0	2
215,714996	11,7867794	46532,9596	0	0	0	0	39	0	0	0
205,884003	13,728919	42388,2226	0	0	0	0	1	4	0	1
190,210999	2,78386497	36180,224	1	0	0	0	3	0	0	1
187,335007	4,98556948	35094,4047	2	0	0	0	0	0	0	0
187,335007	4,98556948	35094,4047	0	0	0	0	0	0	0	0

203,981995	3,2035296	41608,6541	0	0	0	0	3	0	0	0
172,664993	2,3168118	29813,1999	0	0	0	0	0	0	0	0
172,664993	2,3168118	29813,1999	3	0	0	0	4	0	0	0
172,664993	2,3168118	29813,1999	2	0	0	0	0	0	0	0
182,330002	3,99195743	33244,2296	1	0	0	0	1	0	0	0
200,115997	1,5913744	40046,4124	1	0	0	0	0	2	0	0
202,395996	3,896456	40964,1392	2	0	0	0	0	0	0	0
199,893997	2,94144225	39957,6101	3	0	0	0	1	0	0	0
206,283005	4,21770668	42552,6781	0	0	0	0	7	0	1	0
201,414001	5,18400049	40567,6	0	0	0	0	0	1	1	0
198,320999	4,96076584	39331,2187	1	0	0	0	0	0	0	0
197,130997	2,88891649	38860,6299	1	0	0	0	0	0	0	0
197,130997	2,88891649	38860,6299	0	0	0	0	0	0	0	0
189,279007	4,93326044	35826,5425	0	0	0	0	0	0	0	0
215,285995	9,61058903	46348,0599	0	0	0	1	15	0	0	0
219,761002	3,18425012	48294,8978	0	0	0	0	28	0	0	0
190,526993	9,21511841	36300,535	1	0	0	0	0	1	0	0
213,667999	5,41120338	45654,0139	0	0	0	0	19	0	0	0
206,283005	4,21770668	42552,6781	0	0	0	0	3	0	1	0
206,283005	4,21770668	42552,6781	0	0	0	0	2	0	0	0
187,335007	4,98556948	35094,4047	0	0	0	0	0	0	0	0
208,492996	6,05411863	43469,3295	0	0	0	0	4	0	0	4
238,309006	2,30327892	56791,1822	1	0	0	0	13	0	0	0
204,688004	4,04349232	41897,1788	0	0	0	0	0	0	0	0
203,184006	0,40588084	41283,7402	1	0	0	0	0	0	0	0
208,927002	2,75998974	43650,4921	0	0	0	1	1	0	0	0
208,927002	2,75998974	43650,4921	1	0	0	0	2	1	0	4
208,927002	2,75998974	43650,4921	0	0	0	0	0	0	0	0
205,656006	3,91106772	42294,3927	1	0	0	0	0	0	0	0

232,119995	4,24366522	53879,6921	0	0	0	0	5	0	0	0
229,201004	5,24939203	52533,1002	0	0	0	0	1	0	0	0
239,889008	5,73266125	57546,736	0	0	0	0	3	0	0	0
239,276993	3,75033903	57253,4793	1	0	0	0	1	0	0	0
228,798996	2,89311862	52348,9806	0	0	0	0	0	0	0	0
213,628998	3,37332082	45637,3487	0	0	0	0	0	0	0	0
216,052002	1,71652126	46678,4675	1	0	0	1	0	0	1	0
187,600998	5,10250187	35194,1344	2	0	0	0	0	0	0	0
185,572998	5,19020128	34437,3376	0	0	0	0	0	0	0	0
202,192993	5,46809387	40882,0065	1	0	0	0	0	0	0	0
207,966995	1,83690071	43250,2711	1	0	0	0	0	0	0	0
211,742996	5,19020128	44835,0964	1	0	0	0	0	0	0	0
205,968994	3,42888141	42423,2265	0	0	0	0	0	0	0	0
205,968994	3,42888141	42423,2265	1	0	0	0	0	0	0	0
205,968994	3,42888141	42423,2265	1	0	0	0	1	0	0	0
207,018997	6,1612711	42856,8652	1	0	0	0	0	0	0	0
202,593994	6,32710266	41044,3265	1	0	0	0	0	0	0	0
200,753006	8,99873257	40301,7694	1	0	0	0	0	0	0	0
200,815002	7,69833803	40326,6652	2	0	0	0	0	0	0	0
202,748001	0,45253745	41106,7519	1	0	0	0	0	0	0	0
199,740005	6,58541679	39896,0698	0	0	0	0	0	0	0	0
216,671997	13,7767553	46946,7543	0	0	0	0	5	0	0	0
204,936005	11,6847296	41998,766	0	0	0	1	0	0	0	0
190,309998	0,98405397	36217,8952	0	0	0	0	10	0	0	0
213,628998	3,37332082	45637,3487	1	0	0	0	0	0	0	0
213,628998	3,37332082	45637,3487	1	0	0	0	0	0	0	0
227,570007	4,8374691	51788,1082	3	0	0	0	0	0	0	0
228,563995	5,24716043	52241,5	0	0	0	0	4	0	0	0
186,725998	9,37584782	34866,5983	0	0	0	0	1	0	0	0

186,132996	5,7697525	34645,4921	3	0	0	0	0	0	1	0
198,972	3,6967628	39589,8568	2	0	0	0	0	0	0	0
189,979996	4,53758192	36092,3988	4	0	0	0	1	0	1	1
197,947006	4,45584822	39183,0173	1	0	0	0	0	0	0	0
190,309998	0,98405397	36217,8952	0	0	0	0	7	0	0	0
190,309998	0,98405397	36217,8952	0	0	0	0	7	0	0	1
219,936996	17,6746464	48372,2824	0	0	0	0	4	0	0	0
195,395004	1,89749289	38179,2077	0	0	0	0	14	0	0	1
223,261993	3,62277651	49845,9177	1	0	0	0	0	0	0	0
211,164001	3,46669984	44590,2355	0	0	0	0	1	0	0	0
223,304001	3,20456195	49864,6768	1	0	0	0	0	0	0	0
227,697006	3,58859921	51845,9266	1	0	0	0	1	0	0	0
222,432007	2,7064302	49475,9977	0	0	0	0	0	0	0	0
213,406006	11,6425362	45542,1233	0	0	0	0	11	1	0	0
209,218994	12,1474915	43772,5875	0	0	0	0	1	0	0	0
219,488007	12,7875443	48174,985	0	0	0	0	4	0	0	0
196,990005	7,51707888	38805,0623	0	0	0	0	0	0	0	0
195,389999	3,68668389	38177,2519	1	0	0	0	1	0	0	0
198,106003	2,65175486	39245,9883	0	0	0	0	1	0	0	0
202,953003	1,37919116	41189,9214	0	0	0	0	0	4	0	0
201,253998	3,74487209	40503,1716	1	0	0	0	18	0	0	0
197,130997	2,88891649	38860,6299	1	0	0	0	2	0	0	0
197,947006	4,45584822	39183,0173	2	0	0	0	0	0	0	0
197,947006	4,45584822	39183,0173	1	0	0	0	0	0	0	0
188,046005	8,35789871	35361,3001	0	0	0	0	0	0	7	0
216,839005	2,98496366	47019,1539	1	0	0	0	4	0	0	0
216,839005	2,98496366	47019,1539	1	0	0	0	0	0	0	0
216,839005	2,98496366	47019,1539	2	0	0	0	2	0	0	0
216,044006	2,93639183	46675,0127	2	0	0	1	1	0	2	0

224,686005	2,32163835	50483,8007	2	0	0	0	1	0	0	0
202,729004	1,8566035	41099,049	0	0	0	0	28	0	0	0
217,356003	5,08478498	47243,632	0	0	0	3	37	0	0	0
220,386002	5,95899343	48569,9897	0	0	0	1	4	0	0	0
206,080994	6,37812757	42469,3759	0	0	0	0	13	0	0	0
230,514008	4,08200169	53136,7077	1	0	0	0	15	0	0	0
210,753006	12,3277435	44416,8295	0	0	0	1	46	0	0	0
210,753006	12,3277435	44416,8295	0	0	0	0	18	0	0	0
210,753006	12,3277435	44416,8295	0	0	0	0	45	0	0	0
215,283997	4,59225178	46347,1992	0	0	0	0	9	0	0	0
216,031998	3,51763391	46669,824	0	0	0	0	13	0	0	0
200,494003	3,17296362	40197,8454	0	0	0	0	0	0	0	0
194,203995	2,62414384	37715,1916	0	0	0	0	1	0	1	0
192,882004	6,51319361	37203,4674	0	0	0	0	2	0	0	0
212,839005	5,09239817	45300,4418	0	0	0	0	0	0	0	0
193,024002	3,26328206	37258,2654	0	0	0	0	2	0	0	0
198,919006	5,13669395	39568,7711	0	0	0	0	0	0	0	1
195,302002	3,06150126	38142,872	0	0	0	0	0	0	0	0
195,302002	3,06150126	38142,872	0	0	0	0	2	0	0	11
195,302002	3,06150126	38142,872	0	0	0	0	0	0	0	1
197,968994	5,30405092	39191,7226	0	0	0	0	29	0	0	0
216,720993	5,60566521	46967,9888	1	0	0	0	21	0	0	0
211,785004	7,05509329	44852,8878	0	0	0	0	1	0	0	0
212,309998	5,35762691	45075,5351	0	0	0	0	0	1	0	0
204,895996	5,74376249	41982,3692	0	0	0	0	49	0	0	0
213,292999	3,05434823	45493,9035	0	0	0	0	2	0	0	0
208,690994	4,75626087	43551,9311	0	0	0	0	2	0	0	0
217,994003	6,69703388	47521,3855	0	0	0	0	0	0	0	2
217,994003	6,69703388	47521,3855	0	0	0	0	0	0	0	0

217,994003	6,69703388	47521,3855	0	0	0	0	1	0	0	0
207,751007	6,34067774	43160,4809	0	0	0	0	3	0	0	1
210,529007	11,7771263	44322,4628	0	0	0	0	36	5	0	0
207,485001	9,53657627	43050,0255	0	0	0	1	71	4	0	0
207,923004	5,78623724	43231,9757	0	0	0	0	83	0	0	0
219,569	11,9856195	48210,5459	0	0	0	2	34	0	0	0
214,669998	6,50197458	46083,2081	0	0	0	1	110	0	0	0
214,705002	8,78650856	46098,2378	1	0	0	2	72	0	0	0
218,197006	5,71480227	47609,9335	0	0	0	1	46	0	0	0
218,197006	5,71480227	47609,9335	0	0	0	0	44	0	0	1
218,197006	5,71480227	47609,9335	0	0	0	2	35	1	0	0
217,757996	7,41054964	47418,5447	0	0	0	1	68	0	0	0
214,528	8,55254269	46022,2627	0	0	0	1	59	1	0	1
214,072998	15,3001471	45827,2485	0	0	0	0	48	0	0	0
210,274994	1,14097619	44215,5731	0	0	0	0	2	0	0	2
202,033997	4,68792391	40817,7358	0	0	0	0	6	1	0	2
202,033997	4,68792391	40817,7358	0	0	0	0	19	0	0	0
202,033997	4,68792391	40817,7358	0	0	0	0	20	0	0	0
208,292007	1,00787044	43385,5604	0	0	0	0	1	0	0	3
209,873993	8,23126316	44047,0929	0	0	0	0	3	0	0	0
192,876007	1,80304754	37201,1541	0	0	0	0	2	0	0	1
190,203003	8,42102242	36177,1823	0	0	0	0	0	0	0	0
202,436005	6,25056505	40980,336	0	0	0	0	4	0	0	5
208,238998	13,9718513	43363,4805	0	0	0	0	14	0	0	2
220,423004	5,24600983	48586,3008	0	0	0	1	48	0	0	0
219,514999	4,9455018	48186,835	0	0	0	9	54	0	0	0
218,031006	3,40484715	47537,5195	0	0	0	0	41	0	0	0
219,121002	9,13948154	48014,0136	0	0	0	0	0	0	0	0
221,798996	13,6327744	49194,7946	0	0	0	0	40	0	0	0

217,005005	15,4830513	47091,1721	0	0	0	2	62	0	0	0
220,960007	9,4705534	48823,3246	0	0	0	0	0	0	0	0
220,960007	9,4705534	48823,3246	1	0	0	0	0	0	0	0
220,960007	9,4705534	48823,3246	0	0	0	0	1	0	0	0
221,406006	7,42991686	49020,6194	0	0	0	0	0	0	0	0
222,117996	6,00703669	49336,4042	0	0	0	0	0	0	0	0
221,520004	9,7508812	49071,1123	0	0	0	0	2	0	0	0
223,445999	1,69746602	49928,1145	0	0	0	0	1	0	0	0
222,218994	4,65001583	49381,2814	0	0	0	0	0	0	0	0
223,255997	6,77582264	49843,2401	0	0	0	0	0	0	0	0
217,319	6,57768059	47227,5479	0	0	0	1	0	0	0	0
212,584	1,5454694	45191,9569	0	0	0	4	0	0	0	0
212,584	1,5454694	45191,9569	0	0	0	0	0	0	0	0
212,584	1,5454694	45191,9569	0	0	0	1	0	0	0	0
220,651993	4,32939577	48687,3019	0	0	0	2	2	0	0	0
223,824005	5,6856451	50097,1853	0	0	0	0	0	0	0	0
219,069	3,44797778	47991,2269	0	0	0	0	3	0	0	0
228,634995	3,64539576	52273,9607	0	0	0	0	0	0	0	0
212,238007	6,39938784	45044,9714	0	0	0	1	41	0	0	0
225,794006	4,37882996	50982,9333	0	0	0	0	6	0	0	0
227,845993	6,24189568	51913,7965	0	0	0	2	31	0	0	0
221,876007	9,16154194	49228,9625	0	0	0	2	66	0	0	0
221,876007	9,16154194	49228,9625	0	0	0	1	64	0	0	0
221,876007	9,16154194	49228,9625	0	0	0	0	64	0	0	0
227,763	12,0273266	51875,9844	0	0	0	0	34	0	0	0
250,218002	4,96440697	62609,0487	0	0	0	0	21	0	0	0
217,231003	6,59781837	47189,3086	0	0	0	1	60	0	0	0
230,229004	6,77903843	53005,3942	0	0	0	0	38	0	0	0
226,651001	2,91751981	51370,6762	0	0	0	1	20	0	0	0

235,895996	10,0658445	55646,921	0	0	0	0	47	0	0	0
236,479996	4,88489532	55922,7884	0	0	0	0	38	0	0	0
234,005997	4,05880833	54758,8065	0	0	0	0	4	0	0	0
241,5	7,68467712	58322,25	0	0	0	0	3	0	0	0
241,5	7,68467712	58322,25	0	0	0	0	0	0	0	0
241,5	7,68467712	58322,25	0	0	0	0	5	0	0	0
227,567993	3,78822112	51787,1915	0	0	0	0	13	0	0	0
223,156998	6,46066809	49799,0456	0	0	0	0	23	0	0	0
231,031998	4,55398321	53375,784	0	0	0	0	14	0	0	0
231,238007	7,12509155	53471,0157	0	0	0	0	113	0	0	0
222,123993	3,53707433	49339,0682	0	0	0	0	17	0	0	0
225,326996	8,41147423	50772,2551	0	0	0	0	34	0	0	0
236,358994	4,07179642	55865,5738	0	0	0	1	17	0	0	0
230,253998	8,94723701	53016,9035	0	0	0	0	63	0	0	0
230,253998	8,94723701	53016,9035	0	0	0	0	50	0	0	0
230,253998	8,94723701	53016,9035	0	0	0	0	70	0	0	0
228,369003	15,0940838	52152,4017	0	0	0	0	65	0	0	0
229,367004	3,09306312	52609,2227	0	0	0	0	62	0	0	0
235,369003	7,7387948	55398,5677	0	0	0	2	94	0	0	0
215,617004	3,91106772	46490,6926	0	0	0	1	1	0	0	0
227,893997	12,5878229	51935,674	0	0	0	0	70	0	0	0
233,007004	11,8272142	54292,2638	0	0	0	3	46	0	0	0
233,182007	6,78986406	54373,8483	0	0	0	11	5	0	0	0
233,830994	3,18274879	54676,9336	0	0	0	0	39	0	0	0
233,830994	3,18274879	54676,9336	0	0	0	0	127	0	0	0
233,830994	3,18274879	54676,9336	0	0	0	0	67	0	0	0
222,445007	6,3755765	49481,7813	0	0	0	0	8	0	0	0
218,395996	7,8675065	47696,8111	0	0	0	0	3	0	0	0
223,186005	11,1879864	49811,9927	0	0	0	0	0	0	0	0

218,535004	8,16400337	47757,5478	0	0	0	0	0	0	0	0
222,050003	1,2858305	49306,2039	0	0	0	0	6	0	0	0
224,796005	4,68142176	50533,244	0	0	0	0	0	0	0	0
221,302002	5,56569052	48974,5761	0	0	0	1	2	0	0	0
220,738007	7,98773479	48725,2676	0	0	0	0	0	0	0	0
220,738007	7,98773479	48725,2676	0	0	0	0	0	0	0	0
220,738007	7,98773479	48725,2676	0	0	0	4	3	0	0	0
223,266998	2,94144225	49848,1525	0	0	0	0	0	0	0	0
215,479004	4,99701738	46431,2011	0	0	0	0	3	0	0	0
222,848007	4,86610174	49661,2343	0	0	0	0	0	0	0	0
214,235992	3,18274879	45897,0605	0	0	0	0	12	0	0	0
217,035004	1,38901448	47104,1928	0	0	0	0	1	0	0	0
217,386993	1,40705359	47257,1049	0	0	0	0	0	0	0	0
220,395996	3,50306392	48574,3951	0	0	0	1	2	0	0	0
217,891998	0,73728812	47476,9229	0	0	0	0	2	0	0	0
217,891998	0,73728812	47476,9229	0	0	0	0	6	0	0	0
217,891998	0,73728812	47476,9229	0	0	0	0	4	0	0	0
215,966003	2,7058723	46641,3146	0	0	0	0	1	0	0	0
216,770996	5,62821436	46989,6647	0	0	0	0	5	0	0	0
215,994995	3,13881183	46653,8379	0	0	0	0	11	0	0	0
214,401993	4,48291254	45968,2145	1	0	0	0	0	0	0	0
215,050003	3,97843599	46246,5038	1	0	0	0	12	0	0	0
215,445007	3,77585149	46416,5512	2	0	0	0	5	0	0	0
210,763	1,56940591	44421,0424	1	0	0	0	14	0	0	0
214,809998	4,58489227	46143,3351	1	0	0	0	12	0	0	0
214,809998	4,58489227	46143,3351	1	0	0	0	19	1	0	0
214,809998	4,58489227	46143,3351	0	0	0	0	4	0	0	0
216,065002	3,57175136	46684,0853	0	0	0	0	0	0	0	0
209,330002	4,98994684	43819,0497	0	0	0	0	5	0	2	0

211,772995	3,46351647	44847,8014	1	0	0	0	0	0	0	0
191,809998	5,61520529	36791,0752	3	0	0	0	1	0	0	0
211,399002	3,06150126	44689,5381	2	0	0	0	6	0	0	0
215,255005	3,86367345	46334,7171	1	0	0	0	1	1	0	0
211,378998	6,05411863	44681,0807	1	0	0	0	0	0	0	0
212,468002	4,05880833	45142,652	1	0	0	0	0	0	0	2
212,468002	4,05880833	45142,652	1	0	0	0	2	0	0	0
212,468002	4,05880833	45142,652	0	0	0	0	3	0	0	0
191,177002	2,46013498	36548,6461	3	0	0	0	0	0	0	0
189,175995	5,61520529	35787,557	3	0	0	0	0	0	0	0
191,559006	5,15352726	36694,8527	2	0	0	0	0	0	0	0
220,692001	2,34071088	48704,9595	1	0	0	0	0	0	0	0
175,748993	12,3170195	30887,7085	0	0	0	0	3	0	0	4
192,401001	3,72057438	37018,1452	1	0	0	0	8	0	0	0
190,707001	5,35762691	36369,1601	1	0	0	0	0	1	0	0
206,681	3,50681043	42717,0357	3	0	0	0	0	1	0	0
206,681	3,50681043	42717,0357	2	0	0	0	4	0	0	0
206,681	3,50681043	42717,0357	3	0	0	0	0	0	0	0
220,951996	2,91751981	48819,7845	2	0	0	0	0	0	0	0
219,695007	2,91288471	48265,8962	1	0	0	0	0	0	0	0
218,227005	4,36547375	47623,0257	0	0	0	0	0	0	0	0
200,074005	1,3266505	40029,6075	0	0	0	0	1	0	0	0
219,824005	0,91079658	48322,5932	3	0	0	0	0	0	0	0
218,130005	4,23252535	47580,699	1	0	0	0	4	0	0	0
212,643005	1,41819537	45217,0477	1	0	0	0	0	0	0	0
187,257004	4,43221903	35065,1855	3	0	0	0	1	0	0	0
187,257004	4,43221903	35065,1855	0	0	0	2	0	0	0	0
187,257004	4,43221903	35065,1855	2	0	0	0	0	1	0	0
198,076004	3,30116415	39234,1034	2	0	0	0	1	1	0	0

207,707001	2,24587393	43142,1982	1	0	0	1	0	0	0	0
200,279007	3,62277651	40111,6806	3	0	1	0	0	0	0	0
194,341995	1,82159317	37768,8111	0	0	0	0	0	0	0	0
194,335999	4,98259306	37766,4803	3	0	0	0	3	0	0	0
194,690994	4,92901707	37904,5832	1	0	0	0	0	2	0	0
195,932999	4,7511034	38389,74	3	0	0	0	0	0	0	0
205,360001	1,76949155	42172,7299	2	0	0	0	0	0	0	0
205,360001	1,76949155	42172,7299	2	0	0	0	0	0	0	0
205,360001	1,76949155	42172,7299	2	0	0	1	0	1	0	0
199,093002	5,57630587	39638,0236	1	0	0	0	0	5	0	0
199,197998	3,03598881	39679,8424	2	0	0	0	0	8	0	0
184,220993	11,5899687	33937,3743	2	0	0	0	0	0	0	0
185,074997	5,19192743	34252,7545	1	0	0	0	1	2	0	0
188,511002	5,13894844	35536,3977	1	0	0	0	0	1	1	0
175,445999	5,98181677	30781,2986	2	0	0	0	1	2	0	0
213,906998	2,42257929	45756,2037	1	0	0	0	0	4	0	0
213,906998	2,42257929	45756,2037	2	0	0	0	1	0	0	0
213,906998	2,42257929	45756,2037	1	0	0	0	0	0	0	0
209,291	5,00712824	43802,7228	1	0	0	0	0	1	0	0
192,304001	3,01047611	36980,8287	1	0	0	0	0	0	0	0
187,526993	3,34851694	35166,373	4	0	0	0	0	1	0	0
191,011002	3,53707433	36485,2027	1	0	0	0	1	0	0	0
210,897003	4,6075592	44477,5459	0	0	0	0	2	0	0	0
192,438004	4,43763065	37032,3852	2	0	0	0	3	3	0	0
196,916	2,75838232	38775,9112	0	0	0	0	0	1	0	0
195,667999	5,30350971	38285,9659	1	0	0	0	0	1	0	0
195,667999	5,30350971	38285,9659	2	0	0	0	1	1	0	0
195,667999	5,30350971	38285,9659	2	0	0	0	0	1	0	0
202,003006	4,20956421	40805,2144	1	0	0	0	0	0	0	0

209,304001	3,50102377	43808,1648	2	0	0	0	0	1	0	0
213,266998	1,75586104	45482,8126	0	0	0	0	0	1	0	0
211,216003	5,51835585	44612,2001	1	0	0	0	0	0	0	0
219,593002	2,09334087	48221,0867	3	0	0	0	0	0	0	0
206,406006	5,83981371	42603,4393	1	1	0	0	0	0	0	9
205,589996	7,79291201	42267,2466	3	0	0	0	0	0	0	0
240,067993	4,6075592	57632,6413	0	0	0	0	0	1	0	0
240,067993	4,6075592	57632,6413	0	0	0	0	0	0	0	0
240,067993	4,6075592	57632,6413	0	0	0	0	1	0	0	0
216,615005	8,22585201	46922,0606	1	0	0	0	0	0	0	0
217,490997	6,341681	47302,3339	0	0	0	0	0	0	0	0
225,028	3,92539024	50637,6007	3	0	0	0	1	0	0	0
206,895004	3,57175136	42805,5428	1	0	0	0	0	0	0	0
230,128006	3,69312167	52958,8991	0	0	0	0	2	0	0	0
215,084	2,36365891	46261,1269	0	0	0	0	0	0	0	0
222,912003	3,39493203	49689,7609	1	0	0	0	0	1	0	0
198,063995	9,00081348	39229,3463	1	0	0	0	0	0	1	0
198,063995	9,00081348	39229,3463	0	0	0	0	7	1	10	0
198,063995	9,00081348	39229,3463	0	0	0	0	1	0	0	0
207,843002	7,83905458	43198,7136	0	0	0	0	22	0	0	0
228,341995	6,91133881	52140,0668	1	0	0	0	4	0	0	0
223,550003	4,98630619	49974,6039	0	0	0	0	2	0	0	0
227,477005	2,34715104	51745,7878	1	0	0	0	0	0	0	0
222,675995	1,40885746	49584,5987	1	0	0	0	2	0	0	0
225,011002	4,14457941	50629,9508	0	0	0	0	0	0	0	0
227,376007	4,44683027	51699,8486	0	0	0	0	4	0	0	0
219,608002	4,7623353	48227,6744	1	0	0	0	0	0	0	0
219,608002	4,7623353	48227,6744	0	0	0	0	0	0	0	0
218,994995	4,62226677	47958,8079	0	0	0	1	1	0	0	0

223,149994	2,48940253	49795,9198	0	0	0	0	0	0	0	0
217,682007	5,64464283	47385,4561	1	0	0	1	0	0	0	0
227,656006	6,84892559	51827,257	0	0	0	0	0	0	0	0
229,912003	8,01230717	52859,5289	0	0	0	1	0	0	0	0
223,785995	6,3755765	50080,1718	1	0	0	3	0	0	0	0
223,677994	3,05434823	50031,8449	0	0	0	0	0	0	0	0
224,994003	5,40865231	50622,3015	0	0	0	0	0	0	0	0
224,994003	5,40865231	50622,3015	0	0	0	0	0	0	0	0
224,994003	5,40865231	50622,3015	0	0	0	0	0	0	0	0
233,636993	7,23279667	54586,2447	0	0	0	0	0	0	0	1
234,516006	6,75061035	54997,7573	0	0	0	1	0	0	0	0
231,169998	6,3755765	53439,5681	0	0	0	0	0	0	0	0
228,315002	10,5091915	52127,7403	0	0	0	1	0	0	0	0
238,785995	8,83732319	57018,7516	0	0	0	0	0	0	0	0
232,041	5,87278366	53843,0259	1	0	0	0	0	0	0	0
232,873993	4,90698576	54230,2966	0	0	0	3	0	0	0	0
233,320007	5,89830494	54438,2258	0	0	0	0	0	0	0	0
233,320007	5,89830494	54438,2258	0	0	0	0	0	0	0	4
233,320007	5,89830494	54438,2258	0	0	0	0	1	0	0	0
226,007004	7,92710114	51079,1658	0	0	0	0	0	0	0	0
230,238998	7,75035381	53009,9964	0	0	0	1	5	0	0	0
228,701996	6,92448044	52304,6029	0	0	0	0	0	0	0	0
232,257004	5,12264347	53943,3158	0	0	0	0	2	0	0	0
229,352997	3,77585149	52602,7972	0	0	0	0	5	0	0	0
236,095001	5,91360712	55740,8496	0	0	0	2	4	0	0	0
230,977005	4,7511034	53350,3768	0	0	0	0	10	0	0	0
231,427994	4,67479229	53558,9163	0	0	0	0	3	0	0	0
231,427994	4,67479229	53558,9163	0	0	0	0	0	0	0	0
231,427994	4,67479229	53558,9163	0	0	0	0	5	0	0	0

235,468002	4,33967781	55445,1801	0	0	0	0	3	0	0	0
233,639008	4,97880507	54587,1859	0	0	0	0	0	0	0	1
228,567993	5,25459576	52243,3275	0	0	0	0	3	0	0	0
229,380997	6,32200003	52615,6416	0	0	0	0	2	0	0	0
231,220993	3,86632895	53463,1476	0	0	0	0	5	0	0	0
224,686996	5,30295753	50484,2464	0	0	0	0	2	0	0	0
231,287003	6,16938877	53493,6776	0	0	0	0	11	0	0	0
229,350998	5,55791235	52601,8802	0	0	0	1	23	0	0	0
229,350998	5,55791235	52601,8802	0	0	0	0	14	0	0	0
229,350998	5,55791235	52601,8802	0	0	0	0	33	0	0	0
239,298996	4,94150066	57264,0095	0	0	0	0	60	0	0	0
236,569	6,05411863	55964,8919	0	0	0	0	3	0	0	0
226,485992	4,28610182	51295,9048	0	0	0	0	5	0	0	0
230,740997	5,72530746	53241,4078	0	0	0	0	5	0	0	0
218,451004	13,7530842	47720,8412	0	0	0	0	1	0	0	0
234,029999	8,00991821	54770,0403	0	0	0	0	3	0	0	0
230,615997	6,64345741	53183,7382	0	0	0	0	2	0	0	0
235,804993	18,2916965	55603,9946	0	0	0	0	1	0	0	0
235,804993	18,2916965	55603,9946	0	0	0	0	1	0	0	0
235,804993	18,2916965	55603,9946	0	0	0	0	2	0	0	0
234,481003	8,51862717	54981,3407	0	0	0	0	10	0	0	0
220,328995	12,7052298	48544,8659	0	0	0	0	46	0	0	0
209,610001	10,0787048	43936,3524	0	0	0	0	0	0	0	0

tmedium	tcoarse	tloi	aglmac	alpheu	amaaus	antaur	aontri	aricid	armand	artbif
0,50960509	0,00876538	1,1061433	1	0	0	0	3	2	0	0
0,48052227	0,00999867	0,99648527	0	0	0	0	4	5	0	0
0,49406935	0	1,06871792	1	0	0	1	0	0	0	0
0,50210769	0	0,94537453	0	0	0	0	0	6	0	0

0,50680076	0,00907812	0,93246619	0	0	0	0	1	3	0	0
0,49282318	0	0,98836415	0	0	0	0	1	11	0	0
0,49282318	0	0,98836415	1	0	0	0	1	9	0	0
0,49282318	0	0,98836415	0	0	0	0	6	7	0	0
0,54431704	0,01645036	0,97055628	1	0	0	0	0	3	0	0
0,472282	0,0089332	0,89354513	0	0	0	0	2	2	0	0
0,27193498	0	2,1677456	0	0	0	2	0	0	0	0
0,34672999	0	1,27977811	0	0	0	7	0	0	0	2
0,30022718	0	1,93930503	0	0	0	0	0	0	0	0
0,28882631	0	1,96069579	0	0	0	2	0	1	0	0
0,24089547	0	2,1862923	0	0	0	0	0	1	0	0
0,20739519	0	2,24000536	0	0	0	4	0	0	0	0
0,20739519	0	2,24000536	0	0	0	8	0	1	0	0
0,20739519	0	2,24000536	0	0	0	1	0	1	0	0
0,30882947	0	1,38489602	0	0	0	4	0	1	0	0
0,30059485	0	2,02152987	0	0	0	7	0	0	0	0
0,48221753	0,0106974	0,94549357	1	0	0	0	2	8	0	0
0,49986077	0,13753258	0,96093553	0	0	0	2	28	1	0	0
0,48535943	0,0100739	0,91153925	1	0	0	4	0	1	0	0
0,50130347	0	1,00306381	0	0	0	0	5	6	0	0
0,52021962	0,00908638	1,00822517	1	0	0	4	23	5	0	0
0,50933958	0	1,07746415	1	0	0	1	20	1	0	0
0,50933958	0	1,07746415	1	0	0	0	15	0	0	0
0,50933958	0	1,07746415	1	0	0	0	17	0	0	0
0,51658106	0,00881487	1,00659823	1	0	0	0	17	0	0	0
0,52574203	0,01376307	0,91594077	0	0	0	3	74	2	0	0
0,48794088	0,00788932	1,16565647	0	0	0	0	29	5	0	0
0,50255225	0,2648696	1,03560031	0	0	0	3	5	0	1	0
0,51993011	0,0110261	1,01524037	0	0	0	0	68	0	0	0

0,47227571	0	1,14214404	0	0	0	0	77	0	0	0
0,56643279	0,19980888	1,07439192	1	0	0	1	21	1	0	0
0,55176821	0,30921784	1,24217229	0	0	0	4	26	0	0	1
0,55176821	0,30921784	1,24217229	0	0	0	1	8	0	0	0
0,55176821	0,30921784	1,24217229	0	0	0	0	34	0	0	3
0,51015616	0,28677635	1,2580314	0	0	0	3	10	0	3	0
0,46844145	0,12435839	1,08573385	0	0	0	4	15	0	0	2
0,50172181	0,3302252	0,94294873	0	0	0	4	2	1	2	1
0,38268433	0,22568591	1,34165793	0	0	0	6	2	0	0	0
0,47669222	0,27168463	1,31407154	0	0	0	7	7	0	1	0
0,39137247	0,25714933	1,4279888	0	0	0	3	2	0	0	0
0,32969099	0,22028539	1,38018622	0	0	0	12	1	0	3	0
0,32197016	0	1,28044953	0	0	0	6	2	0	0	0
0,32197016	0	1,28044953	0	0	0	17	1	1	0	0
0,32197016	0	1,28044953	0	0	0	6	2	0	0	0
0,40266092	0,22396873	1,69124717	0	0	0	10	0	0	0	0
0,39520684	0,23320634	1,25491155	0	0	0	12	0	0	2	1
0,3922231	0,19917696	1,31298286	0	0	0	9	3	1	0	1
0,37631517	0,20627795	1,921936	0	0	0	11	0	0	0	0
0,42448874	0,03928325	1,21302143	0	0	0	9	4	0	0	0
0,39897069	0,18558285	1,21840428	0	0	0	3	0	0	0	0
0,43832067	0,26416114	1,41555643	0	0	0	12	4	0	0	0
0,4326769	0,21545146	1,34571691	0	0	0	12	0	0	0	0
0,4326769	0,21545146	1,34571691	0	0	0	14	3	0	0	0
0,4326769	0,21545146	1,34571691	0	0	0	6	0	1	1	0
0,42481119	0,26603834	1,78390022	0	0	0	10	2	0	0	0
0,41539025	0,20650711	1,49938487	0	0	0	8	0	0	1	1
0,38032142	0,20427641	2,00307164	0	0	0	18	1	0	1	0
0,29902702	0,01231737	1,85408037	0	0	0	5	0	0	0	0

0,4405593	0,24683899	1,67335113	0	0	0	10	0	0	0	0
0,47118776	0,26454138	1,33562757	0	0	0	9	1	0	1	0
0,38457035	0,19467722	1,91657377	0	1	0	3	0	0	1	0
0,38792744	0,21466638	1,54051842	0	0	0	7	0	0	0	0
0,38792744	0,21466638	1,54051842	0	2	0	3	0	1	0	0
0,38792744	0,21466638	1,54051842	0	2	0	0	1	0	0	3
0,32159967	0	1,52949959	0	0	0	8	0	0	0	0
0,37183822	0,00748539	1,388784	0	0	0	6	0	1	1	1
0,3182122	0,20394554	2,18358444	0	0	0	1	1	3	0	0
0,22130572	0	2,27141035	1	0	0	7	1	0	2	0
0,30007279	0,00847713	1,64001982	0	0	0	12	0	3	0	1
0,25129528	0	2,10264096	0	0	0	4	0	0	0	0
0,22045812	0,01037371	2,54299823	0	0	0	1	0	1	0	0
0,20045608	0	2,76778359	0	0	0	7	0	0	0	0
0,20045608	0	2,76778359	0	0	0	11	1	0	4	0
0,20045608	0	2,76778359	0	0	0	2	0	0	0	0
0,21432928	0	2,1914194	0	0	0	8	0	0	1	0
0,23673157	0	2,37871036	0	0	0	5	0	0	0	0
0,26000912	0	1,8474634	0	0	0	8	0	0	0	0
0,26202482	0	2,03221406	0	0	0	0	0	0	0	0
0,31243079	0	1,4884539	0	0	0	8	0	0	1	0
0,32220877	0	1,46882947	0	0	0	3	0	0	1	1
0,3251287	0	1,5472831	0	0	0	4	1	0	0	0
0,27903105	0	2,03890142	0	0	0	4	0	0	0	0
0,27903105	0	2,03890142	0	0	0	0	0	0	0	0
0,27903105	0	2,03890142	0	0	0	0	0	0	0	0
0,27407714	0	2,02030517	0	0	0	0	0	0	1	0
0,28074189	0	1,63275136	0	0	0	2	0	0	1	3
0,2834539	0	1,53694665	0	0	0	3	0	0	0	0

0,24013196	0	1,80408342	0	0	0	0	0	0	0	0
0,28193566	0	1,77939821	0	0	0	0	0	0	0	0
0,25026227	0	1,85055262	0	0	0	0	0	0	0	0
0,26758122	0	1,98516674	0	0	0	0	0	0	0	1
0,22563501	0	2,14517365	0	0	0	0	0	0	0	0
0,22563501	0	2,14517365	0	0	0	1	0	0	0	0
0,22563501	0	2,14517365	0	0	0	1	0	0	0	0
0,2556268	0	2,18771296	0	0	0	1	0	1	0	0
0,24842921	0	1,79108989	0	0	0	0	0	1	0	0
0,33320318	0	1,99387437	0	0	0	6	0	0	1	0
0,33369885	0	1,35844544	0	0	0	3	0	2	1	0
0,27731478	0	2,46979331	0	0	0	2	0	0	0	0
0,32042809	0	2,03145859	0	0	0	4	0	1	0	0
0,33480338	0	1,69071109	0	0	0	6	0	1	0	0
0,33480338	0	1,69071109	0	0	0	1	0	1	0	0
0,33480338	0	1,69071109	0	0	0	5	1	0	1	0
0,26569512	0	2,29892584	0	0	0	4	0	1	0	0
0,32488382	0	1,66366223	0	0	0	4	0	0	0	0
0,27729293	0	2,21124897	0	0	0	4	0	4	0	0
0,32385499	0	1,42140072	0	0	0	3	0	1	0	0
0,28175046	0	2,18420123	0	0	0	9	0	0	0	0
0,31938347	0	2,33105448	0	0	0	8	0	0	0	0
0,31553726	0	2,15297144	0	0	0	10	0	3	0	0
0,31009903	0	1,64960268	0	0	0	4	0	0	0	0
0,31009903	0	1,64960268	0	0	0	9	0	0	0	0
0,31009903	0	1,64960268	0	0	0	6	0	2	1	0
0,29915356	0	1,80901133	0	0	0	5	0	2	0	0
0,25213837	0	2,11826887	0	0	0	3	0	0	1	0
0,29748714	0	1,92546825	0	0	0	6	0	0	0	0

0,28707988	0	1,64398996	0	0	0	2	1	0	0	0
0,30823763	0	1,81440707	0	0	0	3	0	0	0	1
0,25767342	0	1,97628439	0	0	0	1	0	1	1	0
0,28726028	0	1,64491155	0	0	0	7	0	1	1	0
0,30502473	0	1,62318545	0	0	0	3	0	1	0	1
0,30502473	0	1,62318545	0	0	0	4	0	0	0	0
0,30502473	0	1,62318545	0	0	0	5	0	2	0	0
0,26203829	0	2,15408194	0	0	0	2	0	0	0	0
0,23004898	0	2,27710013	0	0	0	0	0	1	0	0
0,2746407	0	1,80001639	0	0	0	0	0	2	0	0
0,29583141	0	1,568125	0	0	0	1	0	0	0	0
0,38837274	0,01108851	1,40358648	0	0	0	5	0	0	1	0
0,28712266	0	1,37306154	0	0	0	5	0	0	0	0
0,30841032	0	1,67637555	0	0	0	10	0	0	0	0
0,31837198	0	1,56478177	0	0	0	5	0	0	0	0
0,31837198	0	1,56478177	0	0	0	1	0	0	1	0
0,31837198	0	1,56478177	0	0	0	7	0	0	0	0
0,32198382	0	1,80768664	0	0	0	2	0	0	0	0
0,33792365	0	1,66930584	0	0	0	4	2	0	0	0
0,40777558	0	1,40748961	0	0	0	15	0	0	0	0
0,37764353	0	1,28756825	0	0	0	4	0	0	0	0
0,4736378	0,04265258	1,40282928	0	0	0	2	0	1	0	1
0,40666454	0,00807845	1,53319601	0	0	0	6	0	0	0	0
0,43087911	0,01232103	1,04419491	0	0	0	4	1	0	0	0
0,39874617	0,00747804	1,18996471	0	0	0	4	0	0	0	0
0,39874617	0,00747804	1,18996471	0	0	0	2	0	0	0	0
0,39874617	0,00747804	1,18996471	0	0	0	3	0	0	0	0
0,40045836	0	1,247352	0	0	0	2	0	0	0	0
0,40393182	0,00893992	1,39422954	0	0	0	1	0	0	0	0

0,39991253	0,00842803	1,30809098	0	0	0	3	0	0	0	0
0,44499152	0,01134352	1,19631016	0	0	0	3	0	0	0	0
0,44994023	0,2875121	1,93498062	0	0	0	4	1	0	0	0
0,44521566	0,0089483	1,27525762	0	0	0	4	0	0	0	0
0,46518234	0,03016266	1,31712072	0	0	0	8	2	1	0	0
0,45109461	0,02336941	1,38339401	0	0	0	0	1	0	0	0
0,45109461	0,02336941	1,38339401	0	0	0	3	0	0	0	0
0,45109461	0,02336941	1,38339401	0	0	0	0	0	0	0	0
0,47183557	0,05879039	1,60335055	0	0	0	0	2	0	0	0
0,40099374	0,12059132	1,90528239	0	0	0	4	2	0	1	0
0,45879185	0,19952411	1,81443683	0	0	0	2	1	0	0	0
0,45661554	0,21303409	1,86378888	0	0	0	10	0	0	0	0
0,43680104	0,25336248	1,70595047	0	0	0	4	2	0	0	0
0,48579297	0,28544423	1,37612318	0	0	0	2	0	0	0	0
0,41593273	0,21880738	1,93684124	0	0	0	7	0	1	0	1
0,43338327	0,22909651	1,66034605	0	0	0	5	3	0	2	0
0,43338327	0,22909651	1,66034605	0	0	0	8	2	1	0	0
0,43338327	0,22909651	1,66034605	0	0	0	4	2	0	0	0
0,49879302	0,29928632	1,21516254	0	0	0	5	2	0	0	1
0,4275393	0,2246156	1,4475538	0	0	0	10	6	0	0	0
0,44163023	0,24778388	1,69727988	0	0	0	4	0	0	0	0
0,4596116	0,23788007	1,47171227	0	0	0	5	0	0	0	0
0,5594167	0,42341339	0,39616259	0	0	0	2	8	0	0	0
0,46024603	0,25654477	1,3363061	0	0	0	6	5	0	0	0
0,41279259	0,25802978	1,63675869	0	0	0	7	1	0	0	0
0,42906004	0,32890145	1,82073831	0	0	0	8	2	0	0	1
0,42906004	0,32890145	1,82073831	0	0	0	13	19	0	0	0
0,42906004	0,32890145	1,82073831	0	0	0	7	17	0	0	0
0,54961804	0,39966945	1,3898061	0	0	0	10	73	0	0	0

0,56357602	0,43675264	1,57515777	0	0	0	3	4	1	0	0
0,50040651	0,41351448	1,55077303	0	0	0	2	2	0	0	0
0,53715935	0,36391527	1,51949498	0	1	0	0	5	0	0	0
0,49242566	0,0189574	1,15168008	0	0	0	5	87	0	0	0
0,49144145	0,31815199	1,65989247	0	0	0	4	4	0	0	0
0,51553852	0,38888577	1,72872959	0	0	0	1	33	0	0	0
0,53811318	0,36374297	1,26150426	0	0	0	1	18	0	1	0
0,53811318	0,36374297	1,26150426	0	0	0	9	68	0	0	0
0,53811318	0,36374297	1,26150426	0	0	0	2	33	0	0	0
0,59274534	0,2282248	1,38968486	0	0	0	8	58	0	0	0
0,58961211	0,21330968	1,14646718	0	0	0	13	70	1	0	1
0,51236233	0,17247781	1,18934982	0	0	0	0	61	1	0	0
0,54385044	0,03312297	1,43591643	0	0	0	2	39	2	0	0
0,49074926	0	1,12541148	1	0	0	0	6	2	0	0
0,48954119	0,00864013	0,99970601	0	0	0	2	35	0	0	0
0,53692697	0,03929675	0,99100772	0	0	0	0	46	1	0	0
0,51869791	0,01154625	0,90027429	0	0	0	4	45	2	0	0
0,51869791	0,01154625	0,90027429	0	0	0	1	48	2	0	0
0,51869791	0,01154625	0,90027429	0	0	0	0	24	0	0	0
0,47844286	0,01027345	0,86856733	2	0	0	2	14	0	0	0
0,44550504	0	1,07376813	0	0	0	4	24	0	0	0
0,46491029	0	1,0335947	0	0	0	1	7	6	0	0
0,63927449	0,2701257	1,30930745	0	0	0	5	102	0	0	0
0,52133942	0,31263388	1,94701053	0	0	0	6	87	0	0	0
0,64098456	0,24984771	1,39676054	0	0	0	1	121	0	0	0
0,59012687	0,34626462	1,20465846	1	0	0	6	144	0	0	0
0,60904574	0,17780512	1,33532655	0	0	0	9	79	0	0	0
0,63938351	0,23124259	1,36902739	0	0	0	14	69	0	0	0
0,63938351	0,23124259	1,36902739	0	0	0	17	180	0	0	0

0,63938351	0,23124259	1,36902739	0	0	0	8	87	0	0	0
0,5425817	0,25085858	1,39915903	0	0	0	10	50	0	0	0
0,54335424	0,26318891	1,41292958	0	0	0	3	26	0	0	0
0,5433704	0,27405795	1,44639206	0	0	0	5	83	0	0	0
0,49850124	0,30901762	1,96365501	0	0	0	3	0	0	0	0
0,53499033	0,30926505	1,37852748	0	0	0	7	97	0	0	0
0,54562146	0,32381538	1,57774554	0	0	0	7	57	0	0	0
0,54506765	0,26010236	1,42638529	0	0	0	3	23	0	0	0
0,55690549	0,30301579	1,7018725	0	0	0	5	30	0	0	0
0,55690549	0,30301579	1,7018725	0	0	0	5	30	0	0	0
0,55690549	0,30301579	1,7018725	0	0	0	5	73	0	0	0
0,53377188	0,33321434	1,93079025	0	0	0	5	2	0	2	1
0,4751735	0,27882453	1,57217111	0	0	0	2	5	0	0	0
0,53554716	0,32708538	1,3859733	0	0	0	6	5	0	0	0
0,54215713	0,39799815	1,48876728	0	0	0	4	2	0	0	0
0,51217532	0,36510346	1,51123261	0	0	0	3	4	0	1	0
0,56820368	0,35907675	1,4094318	0	0	0	4	4	0	0	0
0,56515313	0,43548372	1,27905903	0	0	0	3	7	1	1	5
0,57851919	0,57245011	1,43372103	0	0	0	1	6	0	0	0
0,57851919	0,57245011	1,43372103	0	0	0	1	5	0	0	0
0,57851919	0,57245011	1,43372103	0	0	0	9	6	1	2	0
0,4894572	0,3346382	1,50490963	0	0	0	11	1	0	0	0
0,51050538	0,37521668	1,27975701	0	0	0	8	8	0	0	0
0,52574099	0,37198871	1,40228849	0	0	0	4	0	0	0	0
0,51211293	0,31373035	1,48518787	0	0	0	5	4	2	0	0
0,53620465	0,35050883	1,63011257	0	0	0	4	7	1	1	0
0,53148067	0,32370712	1,99042006	0	0	0	8	11	0	1	0
0,49636707	0,29000661	1,70615328	0	0	0	3	4	0	1	0
0,50850124	0,22317665	1,40791264	0	0	0	3	6	0	0	0

0,50850124	0,22317665	1,40791264	0	0	0	6	8	0	0	0
0,50850124	0,22317665	1,40791264	0	0	0	10	4	0	0	0
0,48380609	0,25479142	1,5752057	0	0	0	2	4	0	0	0
0,4721619	0,27272858	1,31784028	0	0	0	7	1	0	2	0
0,46871171	0,18589808	1,39564967	0	0	0	4	0	1	0	0
0,44701726	0,0084789	1,23767241	0	0	0	0	0	0	0	0
0,46610459	0,23795724	1,47577674	0	0	0	2	1	0	0	0
0,49981453	0,18799459	1,3697456	0	0	0	2	0	0	0	0
0,47407753	0,2037613	1,26612717	0	0	0	5	0	0	0	0
0,48473478	0,22632087	1,74467905	0	0	0	1	2	0	0	0
0,48473478	0,22632087	1,74467905	0	0	0	5	2	0	0	0
0,48473478	0,22632087	1,74467905	0	0	0	0	0	0	0	0
0,47147869	0,0306399	1,30758174	0	0	0	8	4	0	0	1
0,44621852	0,00957929	1,20031204	0	0	0	1	1	0	0	0
0,48745702	0,18760247	1,37722438	0	0	0	2	0	0	0	0
0,29836948	0,01025542	2,26802094	0	0	0	1	0	1	0	0
0,43830206	0,00709724	1,12338506	0	0	0	7	0	0	0	0
0,47966702	0,01715689	1,15470039	0	0	0	4	0	0	0	0
0,45304742	0,01171778	1,14707846	0	0	0	1	1	0	0	0
0,52172	0,18274159	1,22162105	0	0	0	0	3	0	0	0
0,52172	0,18274159	1,22162105	1	0	0	6	0	1	0	0
0,52172	0,18274159	1,22162105	0	0	0	3	0	1	0	0
0,43650239	0,00939375	1,41794499	0	0	0	5	0	0	0	0
0,44897352	0,03268915	1,52568149	0	0	0	1	0	0	0	0
0,37079449	0	1,7550245	0	0	0	0	0	0	0	0
0,33711283	0	1,48664454	0	0	0	3	0	1	0	0
0,40091833	0	1,41333648	0	0	0	2	0	0	0	0
0,37369048	0	1,16832958	1	0	0	3	4	0	0	0
0,38366009	0	1,02884936	0	0	0	4	1	1	0	0

0,36294299	0	1,47688354	0	0	0	0	0	0	0	0
0,36294299	0	1,47688354	0	0	0	3	0	0	0	0
0,36294299	0	1,47688354	1	0	0	3	0	1	0	1
0,32795522	0	1,30512605	0	0	0	3	0	0	0	0
0,32079073	0	1,09139177	0	0	0	0	0	1	0	0
0,34807169	0	1,59704602	0	0	0	7	0	0	0	0
0,35486473	0	1,63912629	0	0	0	1	0	0	0	0
0,34089066	0	1,37257022	0	0	0	2	0	1	0	0
0,26620649	0	1,42213642	0	0	0	1	0	1	0	0
0,29358571	0	1,25376792	1	0	0	6	2	0	1	0
0,27319716	0	1,35446927	0	0	0	3	6	0	0	0
0,27319716	0	1,35446927	0	0	0	3	0	0	0	0
0,27319716	0	1,35446927	0	0	0	4	0	0	0	0
0,34370237	0	1,58095762	0	0	0	4	0	1	0	0
0,34579527	0	1,29080246	0	0	0	7	0	0	0	0
0,32634152	0	1,41292958	0	0	0	1	0	3	0	0
0,36163298	0	1,5095655	0	0	0	2	1	0	0	0
0,34243807	0	1,61821785	0	0	0	3	0	1	1	0
0,34888165	0	1,71564653	0	0	0	3	0	0	0	0
0,3863492	0	1,70841125	0	0	0	9	0	1	0	0
0,35718708	0	1,46181223	0	0	0	3	0	1	0	0
0,35718708	0	1,46181223	0	0	0	0	0	0	0	0
0,35718708	0	1,46181223	1	0	0	5	0	1	0	0
0,361007	0	1,56702712	0	0	0	1	0	2	0	0
0,3758528	0	1,51460028	0	0	1	0	1	1	0	0
0,32261024	0	1,43826667	0	0	0	1	0	1	0	0
0,40391369	0,00917128	1,7957678	0	0	0	0	0	4	0	0
0,33110656	0	1,35303104	0	0	0	0	0	1	0	0
0,36203275	0	1,64170003	0	0	0	4	0	1	0	1

0,3696013	0	1,8824415	0	0	0	2	0	0	0	0
0,32535972	0	1,96906602	0	0	0	5	0	0	0	0
0,32535972	0	1,96906602	0	0	0	0	0	0	0	0
0,32535972	0	1,96906602	0	0	0	1	0	1	0	0
0,35951255	0	1,86050531	0	0	0	2	0	0	1	0
0,36238177	0	1,47979086	0	0	0	7	0	2	1	0
0,37697931	0	1,45937658	0	0	0	0	0	0	0	0
0,55224637	0,30437296	1,53297554	0	0	0	4	0	0	0	0
0,40553945	0,00868688	1,60732075	0	0	0	2	0	0	0	0
0,42547522	0,01224816	1,47669022	0	0	0	0	2	0	0	0
0,42031525	0,01088874	1,5616264	0	0	0	1	0	1	0	0
0,42031525	0,01088874	1,5616264	0	0	0	0	0	0	0	0
0,42031525	0,01088874	1,5616264	0	0	0	0	1	1	1	0
0,43159121	0,01152544	1,65705341	0	0	0	0	0	0	0	0
0,43263749	0	1,42123256	0	0	0	2	0	1	1	0
0,39755601	0	1,71458975	0	0	0	1	0	1	0	0
0,39461351	0	1,45874329	0	0	0	4	0	0	0	0
0,41890096	0	1,70811212	0	0	0	1	0	1	0	0
0,42654974	0	1,48466225	0	0	0	1	0	1	0	0
0,43312765	0	1,63445128	0	0	0	1	0	1	0	0
0,4440109	0	1,29549334	0	0	0	0	0	0	0	0
0,4440109	0	1,29549334	0	0	0	2	0	1	0	0
0,4440109	0	1,29549334	0	0	0	5	0	0	0	0
0,3647297	0	1,56243976	0	0	0	8	0	0	0	0
0,37021704	0	1,58652293	0	0	0	3	0	1	2	0
0,41850683	0	1,5551897	0	0	0	0	0	0	0	0
0,41475816	0	1,52517442	0	0	0	0	0	0	0	0
0,38894776	0	0,99631461	0	0	0	6	0	0	0	0
0,38529502	0	1,49794493	0	0	0	1	0	0	0	0

0,41522461	0	1,59704227	0	0	0	1	0	0	1	0
0,36099914	0	1,16193244	0	0	0	0	0	0	0	0
0,36099914	0	1,16193244	1	0	0	0	0	1	0	0
0,36099914	0	1,16193244	0	0	0	5	0	1	0	0
0,3793388	0	1,38645736	0	0	0	6	1	1	0	0
0,4323115	0	1,15802375	0	0	0	7	0	2	0	0
0,41125792	0	1,21549496	0	0	0	2	0	0	0	0
0,41259295	0	1,3008159	0	0	0	4	0	0	0	0
0,40414843	0	1,2609544	0	0	0	1	0	0	0	0
0,41270564	0	1,44636061	0	0	0	0	0	0	0	0
0,39675952	0	1,46125768	0	0	0	4	0	0	0	0
0,37816578	0	1,25526451	0	0	0	1	0	0	0	0
0,37816578	0	1,25526451	0	0	0	0	0	0	0	0
0,37816578	0	1,25526451	0	0	0	1	1	0	0	0
0,3893426	0	1,21339524	0	0	0	5	1	4	0	0
0,36094799	0	1,88598568	0	0	0	1	0	1	0	0
0,3809617	0,00731238	1,94404347	0	0	0	4	0	0	0	0
0,36700983	0,00918163	1,47114309	0	0	0	1	0	0	0	0
0,49035979	0,03542082	1,24058736	0	0	0	6	0	0	0	0
0,37032909	0	1,76479659	0	0	0	2	0	2	0	0
0,39998181	0	1,63038431	0	0	0	5	0	1	0	0
0,43634198	0,00901677	1,47767892	0	0	0	5	0	1	0	0
0,43634198	0,00901677	1,47767892	0	0	0	3	1	0	0	0
0,43634198	0,00901677	1,47767892	0	0	0	7	3	0	0	0
0,42435257	0,00963446	1,21957985	0	0	0	2	1	0	1	0
0,47164202	0,01345886	1,25910127	0	0	0	12	0	0	0	0
0,47968864	0,03465459	1,3926166	0	0	0	3	0	0	2	0
0,47829261	0,01322045	1,16519912	0	0	0	9	0	0	0	0
0,45784788	0,01741639	1,45437478	0	0	0	2	2	0	1	0

0,45295543	0,00768317	1,24601445	0	0	0	8	0	0	0	0
0,45583919	0,00831815	1,16859745	0	0	0	3	2	0	0	0
0,55960488	0,17004099	1,16059381	0	0	0	3	1	0	0	0
0,55960488	0,17004099	1,16059381	0	0	0	6	1	0	0	0
0,55960488	0,17004099	1,16059381	0	0	0	7	0	0	0	2
0,57064259	0,30379226	1,08144209	0	0	0	5	4	0	0	0
0,52706233	0,14697971	0,98343378	0	0	0	8	0	0	0	0
0,48611526	0,15687404	1,05677765	0	0	0	4	1	0	0	0
0,50216197	0,15884531	1,12824864	0	0	0	1	1	1	4	0
0,51380156	0,29380905	1,13377599	0	0	0	6	0	0	0	0
0,39804405	0	1,32706255	1	0	0	6	3	0	1	0
0,53718278	0,19328373	1,33999254	0	0	0	0	1	0	0	0
0,53064199	0,25648581	1,18874556	0	0	0	3	2	0	0	0
0,53064199	0,25648581	1,18874556	0	0	0	2	0	0	0	0
0,53064199	0,25648581	1,18874556	0	0	0	3	0	0	0	0
0,52243567	0,31251928	1,12227269	0	0	0	1	2	0	2	1
0,52769088	0,31427813	1,25931608	0	0	0	5	0	0	0	0
0,54122305	0,36708238	1,31937258	0	0	0	5	2	0	2	0
0,53611174	0,3632332	1,2857317	0	0	0	4	3	1	0	0
0,5099292	0,31314748	1,29406723	0	0	0	1	9	0	0	0
0,52589429	0,28279429	1,22890968	1	0	0	8	0	0	0	0
0,45932693	0,03983817	1,52525998	0	0	0	10	0	0	0	0
0,48700246	0,28850722	1,4213381	0	0	0	3	0	0	0	0
0,48700246	0,28850722	1,4213381	0	0	0	4	7	0	2	0
0,48700246	0,28850722	1,4213381	0	0	0	3	1	0	0	2
0,53722031	0,31027585	1,2626884	0	0	0	7	6	0	2	0
0,57274085	0,41110003	1,43143215	0	0	0	3	5	0	0	0
0,51686031	0,33898226	1,34248687	0	0	0	5	1	0	1	0
0,54181373	0,39827726	1,51772231	0	0	0	2	1	0	0	0

0,4663601	0,24723864	1,68868292	0	0	0	3	1	0	0	0
0,55864707	0,43252031	1,47486101	0	0	0	3	3	0	1	1
0,55784557	0,37011845	1,3953831	0	0	0	2	5	0	0	0
0,43533428	0,24865588	1,58088551	0	1	0	4	0	0	1	0
0,43533428	0,24865588	1,58088551	0	0	0	1	0	0	0	0
0,43533428	0,24865588	1,58088551	0	1	0	2	1	1	3	1
0,50996195	0,30631443	1,50706735	0	0	0	6	0	0	3	0
0,55807066	0,32204794	1,38891036	0	2	0	0	0	0	1	0
0,6202689	0,36890684	1,09678804	0	0	0	0	5	1	0	0
0,47257457	0,26284783	1,56306046	0	0	0	0	1	0	0	0
0,55224637	0,30437296	1,40211412	0	0	0	11	22	0	0	0
0,48920846	0,28148222	1,64389811	0	0	0	3	4	0	0	0
0,51136778	0,30939782	1,53527913	0	0	0	4	5	0	0	0
0,54012707	0,26362051	1,51462173	0	0	0	9	8	0	0	0
0,54012707	0,26362051	1,51462173	0	0	0	12	15	0	0	0
0,54012707	0,26362051	1,51462173	0	0	0	4	4	0	0	0
0,55710416	0,21597935	1,34794584	0	0	0	4	42	0	0	0
0,56747586	0,34357911	1,60195287	0	0	0	11	38	0	0	0
0,56932796	0,26239083	1,4214169	0	0	0	10	26	0	0	0

tmedium	tcoarse	tloi	aglmac	alpheu	ampeli	antaur	aontri	aricid	artbif	auscra
0,505708516	0,207081264	0,16526874	0	0	0	0	3	0	0	0
0,630319217	0,374931387	0,130210037	0	0	0	1	4	0	0	0
0,499301567	0,228111176	0,163429647	0	0	0	0	0	0	0	0
0,500886221	0,192480072	0,149903376	0	0	0	1	3	0	0	0
0,557602724	0,27106553	0,14217258	0	0	0	0	4	0	5	0
0,543324077	0,322037778	0,143779552	0	0	0	0	1	0	0	0
0,543324077	0,322037778	0,143779552	0	0	0	3	4	0	0	0
0,543324077	0,322037778	0,143779552	0	0	0	0	6	0	0	0

0,632438366	0,366872019	0,162964031	0	0	0	0	20	0	0	0
0,633683827	0,346751889	0,146072299	0	0	0	3	7	0	3	0
0,59497074	0,216198715	0,107988844	0	0	0	1	1	0	0	0
0,53268689	0,129530209	0,113695753	0	0	0	0	2	1	0	0
0,563919968	0,129593003	0,103325892	0	0	0	16	6	0	0	0
0,57521813	0,160023933	0,120394762	0	0	0	1	1	0	0	0
0,576785042	0,169399024	0,123364204	0	0	0	1	2	1	0	0
0,548873758	0,174313509	0,134536015	0	0	0	0	0	1	1	1
0,548873758	0,174313509	0,134536015	0	0	0	0	0	0	0	0
0,548873758	0,174313509	0,134536015	0	0	0	0	1	0	0	0
0,602100753	0,226350726	0,101113926	0	0	0	0	3	0	0	0
0,570616193	0,183383101	0,114567657	0	0	0	0	9	0	0	0
0,611556537	0,328429771	0,142578641	0	0	0	0	4	0	0	0
0,51259107	0,246010312	0,152860721	0	0	0	0	5	0	0	0
0,65095881	0,424791082	0,122846912	0	0	0	1	6	0	0	0
0,637606881	0,374751172	0,156012849	0	0	0	0	9	0	0	0
0,619184302	0,336227006	0,153017962	0	0	0	2	9	0	1	0
0,600192657	0,33691079	0,147856022	0	0	0	1	8	0	0	0
0,600192657	0,33691079	0,147856022	0	0	0	0	11	0	0	0
0,600192657	0,33691079	0,147856022	0	0	0	1	7	0	0	0
0,481677972	0,225773515	0,154076712	0	0	0	0	0	0	0	0
0,506957563	0,224637817	0,139628219	0	0	0	0	0	0	0	0
0,522334378	0,261028983	0,158133658	0	0	0	0	4	0	0	0
0,55503914	0,285794645	0,152718522	0	0	0	0	3	0	0	0
0,519393489	0,299594525	0,15385889	0	0	0	1	3	0	0	0
0,539307967	0,379829421	0,152037359	0	0	0	2	4	0	0	0
0,554605882	0,315729167	0,190778724	0	0	0	0	1	0	0	0
0,519016747	0,303880546	0,151451181	0	0	0	0	0	0	0	0
0,519016747	0,303880546	0,151451181	0	0	0	0	0	0	0	0

0,519016747	0,303880546	0,151451181	0	0	0	0	0	0	0	0
0,53138522	0,257041531	0,130581345	0	0	0	0	1	0	0	0
0,551864199	0,327093757	0,134793564	0	0	0	0	0	0	2	0
0,520446922	0,225961911	0,134543839	0	0	0	1	1	0	0	0
0,501640106	0,201607164	0,14031544	0	0	0	1	0	0	0	0
0,467895253	0,167128598	0,157395451	0	0	0	0	0	0	0	0
0,505387304	0,193928684	0,135988435	0	0	0	0	0	0	1	0
0,510741207	0,23002403	0,119409382	0	0	0	1	1	0	0	0
0,437194961	0,089055737	0,137882799	0	0	0	0	0	0	0	0
0,437194961	0,089055737	0,137882799	0	0	0	1	0	0	0	0
0,437194961	0,089055737	0,137882799	0	0	0	2	0	0	0	0
0,49617326	0,193946372	0,139586311	0	0	0	0	0	0	0	0
0,477208727	0,209204391	0,127131297	0	0	0	1	0	0	0	0
0,52660561	0,205811665	0,158248305	0	0	0	2	0	0	0	0
0,514930503	0,202706714	0,132469492	0	0	0	0	0	0	0	0
0,493400318	0,197098823	0,137051108	0	0	0	1	0	0	0	0
0,514841607	0,249000939	0,126243645	0	0	0	0	0	0	0	0
0,495951208	0,210842681	0,121201649	0	0	0	0	0	0	0	0
0,474621671	0,216824476	0,125084816	0	0	0	0	0	0	2	0
0,474621671	0,216824476	0,125084816	0	0	0	0	0	0	0	0
0,474621671	0,216824476	0,125084816	0	0	0	0	0	0	0	0
0,539559605	0,229805123	0,143326692	0	0	0	0	0	0	0	0
0,529998327	0,18854663	0,119485028	0	0	0	4	0	0	0	0
0,478063259	0,161381482	0,117461	0	0	0	4	0	0	0	0
0,546130119	0,17758193	0,134722319	0	0	0	0	3	0	0	0
0,492258812	0,141777756	0,130458479	0	0	0	0	0	0	0	0
0,505514411	0,197734432	0,119117175	0	0	0	0	0	0	0	0
0,540413854	0,268208227	0,139408308	0	0	0	1	0	0	0	0
0,494780633	0,205323138	0,127217142	0	0	0	4	0	0	0	0

0,494780633	0,205323138	0,127217142	0	0	0	0	0	0	0	0
0,494780633	0,205323138	0,127217142	0	0	0	1	2	0	0	0
0,488871475	0,183148322	0,133572435	0	0	0	0	3	0	0	0
0,480956271	0,176503028	0,116869852	0	0	0	6	0	0	0	0
0,559370793	0,215296219	0,140583927	0	0	0	0	0	0	0	0
0,536683147	0,130271463	0,114041674	0	0	0	1	2	0	1	0
0,516420141	0,151319274	0,129214889	0	0	0	0	4	0	0	0
0,54883354	0,1482991	0,127469383	0	0	0	1	9	0	0	0
0,553308005	0,196200895	0,121319949	0	0	0	0	2	0	0	0
0,514364649	0,12019323	0,114088765	0	0	0	5	2	0	0	0
0,514364649	0,12019323	0,114088765	0	0	0	1	1	0	0	0
0,514364649	0,12019323	0,114088765	0	0	0	0	0	0	0	0
0,558514121	0,119992753	0,120972555	0	0	0	0	0	0	0	0
0,518792317	0,091110395	0,119144292	0	0	0	0	0	0	1	0
0,521579385	0,09029593	0,128879791	0	0	0	0	2	0	0	0
0,50777514	0,066063497	0,112312597	0	0	0	0	3	0	0	0
0,560387132	0,148611234	0,111636947	0	0	0	0	5	0	0	0
0,535269812	0,111894487	0,125086513	0	0	0	5	1	0	0	0
0,539269274	0,100926403	0,127489604	0	0	0	1	1	0	0	0
0,520855351	0,069606464	0,142810551	0	0	0	1	1	0	0	0
0,520855351	0,069606464	0,142810551	0	0	0	0	0	0	0	0
0,520855351	0,069606464	0,142810551	0	0	0	4	0	0	0	0
0,550075805	0,17913357	0,119057666	0	0	0	3	2	0	0	0
0,545592607	0,099565023	0,123041433	0	0	0	2	2	0	0	0
0,500816419	0,059245716	0,117089883	0	0	0	4	2	0	0	0
0,568575005	0,160879448	0,136040131	0	0	0	1	3	0	0	0
0,527680997	0,070712791	0,127240025	0	0	0	7	3	0	0	0
0,548249304	0,160787656	0,130255469	0	0	0	7	2	0	0	0
0,543697985	0,105719234	0,109240533	0	0	0	1	3	0	1	0

0,563603812	0,135910948	0,10910005	0	0	0	4	1	0	1	0
0,563603812	0,135910948	0,10910005	0	0	0	5	8	0	2	0
0,563603812	0,135910948	0,10910005	0	0	0	0	9	0	1	0
0,553158026	0,204399665	0,133474589	0	0	0	0	0	0	2	0
0,569297897	0,190083849	0,134828403	0	0	0	0	4	1	0	0
0,526152913	0,175980797	0,128811555	0	0	0	0	1	0	0	0
0,587999887	0,222984764	0,117671412	0	0	0	0	4	0	0	0
0,520901917	0,182870172	0,117752096	1	0	0	0	3	1	1	0
0,517742058	0,111083987	0,135443475	0	0	0	0	0	1	0	0
0,59515094	0,204384621	0,117541354	0	0	0	1	3	5	0	0
0,59515094	0,204384621	0,117541354	0	0	0	0	3	1	0	0
0,59515094	0,204384621	0,117541354	0	0	0	0	7	1	0	0
0,560978676	0,197669995	0,1399654	0	0	0	0	0	0	0	0
0,626426305	0,271697374	0,125932357	0	0	0	0	2	0	1	0
0,630466959	0,274163351	0,123830273	0	0	0	1	5	0	1	0
0,595771477	0,257547102	0,116980457	0	0	0	0	0	0	1	0
0,579854883	0,160825398	0,11774631	0	0	0	8	2	0	0	0
0,615052587	0,27733041	0,13227053	0	0	0	0	0	0	0	1
0,576778042	0,213378862	0,131289759	0	0	0	0	0	0	0	0
0,556209791	0,130257487	0,129306068	0	0	0	1	1	0	0	0
0,556209791	0,130257487	0,129306068	0	0	0	2	0	0	0	0
0,556209791	0,130257487	0,129306068	0	0	0	3	0	0	0	0
0,589044419	0,194638045	0,125966139	0	0	0	0	9	0	0	0
0,593708827	0,166246079	0,126068395	0	0	0	8	5	0	0	0
0,613404236	0,184495917	0,105780351	0	0	0	5	7	0	0	0
0,612799484	0,183948612	0,10460922	0	0	0	2	0	0	0	0
0,63310425	0,216976883	0,102373932	0	0	0	4	1	0	0	0
0,602198119	0,18001363	0,105711801	0	0	0	9	6	0	3	0
0,602702294	0,214196244	0,121534484	0	0	0	0	3	0	0	0

0,601458531	0,186013055	0,10625794	0	0	0	6	1	0	0	0
0,601458531	0,186013055	0,10625794	0	0	0	3	2	0	0	0
0,601458531	0,186013055	0,10625794	0	0	0	0	2	0	1	0
0,57879831	0,160854433	0,128265015	0	0	0	1	0	0	0	0
0,591802798	0,17016132	0,127020528	0	0	0	4	4	0	0	0
0,61617519	0,244573289	0,101011412	0	0	0	3	5	0	0	0
0,610508343	0,196872541	0,11913726	0	0	0	0	3	0	1	0
0,553188784	0,220935579	0,123589279	0	0	0	0	1	0	0	0
0,586226127	0,174924821	0,108831376	0	0	0	1	2	0	0	0
0,521213797	0,174246988	0,127031868	0	0	0	2	4	0	0	0
0,611612803	0,271499667	0,113091121	0	0	0	5	0	0	0	0
0,611612803	0,271499667	0,113091121	0	0	0	2	3	0	0	0
0,611612803	0,271499667	0,113091121	0	0	0	3	1	0	1	0
0,600319558	0,277356441	0,104124085	0	0	0	1	0	0	0	0
0,563797546	0,234785671	0,133181276	0	0	0	1	5	0	0	0
0,515195384	0,201168384	0,133380618	0	0	0	0	1	0	0	0
0,526684431	0,190893417	0,116129521	0	0	0	0	2	0	0	0
0,562374374	0,257385043	0,130836288	0	0	0	0	1	0	0	0
0,595599441	0,277377572	0,122250169	0	0	0	1	1	0	0	0
0,538859206	0,200103634	0,119200795	0	0	0	0	2	0	0	0
0,549309413	0,257446377	0,123886515	0	0	0	0	0	0	0	0
0,549309413	0,257446377	0,123886515	0	0	0	0	0	0	0	0
0,549309413	0,257446377	0,123886515	0	0	0	2	0	0	0	0
0,564819319	0,237499171	0,115186657	0	0	0	0	0	0	0	0
0,616969543	0,351964314	0,112292842	0	0	0	0	0	0	0	0
0,558248399	0,261430256	0,123458873	0	0	0	0	2	0	0	0
0,559879032	0,258813896	0,128506117	0	0	0	3	1	0	0	0
0,524791339	0,210112759	0,132641199	0	0	0	0	0	0	0	0
0,580345349	0,267388326	0,105561303	0	0	0	0	4	0	0	0

0,574708781	0,252966192	0,113110874	0	0	0	2	1	0	0	0
0,563974643	0,249214015	0,129602874	0	0	0	3	2	0	0	0
0,563974643	0,249214015	0,129602874	0	0	0	0	2	0	0	0
0,563974643	0,249214015	0,129602874	0	0	0	3	4	0	0	0
0,568548998	0,242367332	0,139461238	0	0	0	1	3	0	1	0
0,566259082	0,229476568	0,12196824	0	0	0	1	1	0	0	0
0,530484466	0,196797381	0,129554097	0	0	0	0	0	0	0	0
0,501167381	0,17406129	0,139468611	0	0	0	0	2	0	0	0
0,463337037	0,14566878	0,132547185	0	0	0	3	0	0	0	0
0,499546677	0,185138282	0,11952693	0	0	0	0	1	0	0	0
0,492345962	0,183658616	0,130011354	0	0	0	0	0	0	0	0
0,525401008	0,21306893	0,121069515	0	0	0	1	0	0	0	0
0,525401008	0,21306893	0,121069515	0	0	0	0	0	0	0	0
0,525401008	0,21306893	0,121069515	0	0	0	0	3	0	1	0
0,493195079	0,184364175	0,146745637	0	0	0	1	0	0	2	0
0,49666217	0,190645773	0,120349297	0	0	0	2	4	0	0	0
0,513070804	0,209236556	0,132023849	0	0	0	0	3	0	0	0
0,51030985	0,189273774	0,14228828	0	0	0	0	1	0	0	0
0,551665387	0,284828073	0,117782992	0	0	0	0	0	0	0	0
0,540613187	0,226110985	0,118054501	0	0	0	0	1	0	0	0
0,508806171	0,213362989	0,133547999	0	0	0	1	1	0	0	0
0,475496427	0,220807934	0,153828017	0	0	0	0	0	0	0	0
0,475496427	0,220807934	0,153828017	0	0	0	0	0	0	0	0
0,475496427	0,220807934	0,153828017	0	0	0	0	2	0	0	0
0,504933822	0,243955447	0,130776436	0	0	0	0	1	0	1	0
0,475241956	0,245587271	0,137150784	0	0	0	0	0	0	0	0
0,582300993	0,327621893	0,139069958	0	0	0	3	5	0	1	0
0,474096611	0,239387757	0,14404626	0	0	0	1	1	0	0	0
0,623048098	0,382200397	0,180099192	0	0	0	0	1	0	0	0

0,479121268	0,244348878	0,155309003	0	0	0	2	3	0	0	0
0,484391014	0,200200145	0,164457335	0	0	0	6	3	0	0	0
0,516361916	0,322539225	0,160876128	0	0	0	1	0	0	0	0
0,516361916	0,322539225	0,160876128	0	0	0	0	1	0	0	0
0,516361916	0,322539225	0,160876128	0	0	0	1	3	0	0	0
0,590604985	0,315791856	0,163619437	0	0	0	0	8	0	0	0
0,634122614	0,448737682	0,154392225	0	0	0	1	1	0	0	0
0,581946934	0,334577757	0,162979427	0	0	0	0	2	0	0	0
0,255936032	0,359482948	0,206526421	0	0	0	1	5	0	0	0
0,594585587	0,303166015	0,119342995	0	0	0	0	7	0	0	0
0,661826545	0,389838401	0,133864118	0	0	0	0	14	0	0	0
0,610640329	0,403291564	0,181501536	0	0	0	0	0	0	0	0
0,623060865	0,336008615	0,124879819	0	0	0	0	6	0	0	0
0,623060865	0,336008615	0,124879819	0	0	0	1	11	0	0	0
0,623060865	0,336008615	0,124879819	0	0	0	0	0	0	1	0
0,622255375	0,405202666	0,132618121	0	0	0	0	3	0	0	0
0,608080717	0,333473727	0,127351837	0	0	0	2	0	0	1	0
0,603443959	0,31958243	0,136019738	0	0	0	3	9	0	0	0
0,44301386	0,217992397	0,159926355	0	0	0	0	0	0	0	0
0,614554581	0,302510494	0,109365493	0	0	0	5	0	0	0	0
0,41311334	0,165864867	0,165148687	0	0	0	0	1	0	0	0
0,507077709	0,282012864	0,182059431	0	0	0	1	12	0	0	0
0,451834605	0,198737368	0,168891906	0	0	0	4	0	0	0	0
0,557117643	0,344681762	0,148594399	0	0	0	0	17	0	0	0
0,557117643	0,344681762	0,148594399	0	0	0	8	10	0	0	0
0,557117643	0,344681762	0,148594399	0	0	0	0	1	0	0	0
0,608657173	0,359092708	0,122532864	0	0	0	7	7	0	1	0
0,662075082	0,344352723	0,125624175	0	0	0	1	4	0	0	0
0,672940306	0,375249676	0,107906967	0	0	0	7	4	0	0	0

0,477807387	0,236355564	0,157332426	0	0	0	3	2	0	1	0
0,670135081	0,350033518	0,123436082	0	0	0	3	5	0	0	0
0,60177861	0,305775217	0,12142849	0	0	0	1	13	0	0	0
0,5954038	0,333224856	0,148709541	0	0	0	1	16	0	0	0
0,588466172	0,339456625	0,163602356	0	0	0	1	12	0	0	0
0,588466172	0,339456625	0,163602356	0	0	0	1	0	0	1	0
0,588466172	0,339456625	0,163602356	0	0	0	1	9	0	0	0
0,512012375	0,274228072	0,152822974	0	0	0	2	11	0	2	0
0,461761562	0,210425776	0,138752233	0	0	0	0	5	0	4	0
0,466598844	0,230947082	0,17449231	0	0	0	1	1	0	1	0
0,45138358	0,162137808	0,130809652	0	0	0	0	0	0	0	0
0,518127386	0,244849242	0,156870183	0	0	0	1	0	0	0	0
0,471003295	0,261222174	0,151237049	0	0	0	18	0	0	2	0
0,451228665	0,209941723	0,138029811	0	0	0	0	0	0	0	0
0,477512714	0,22045784	0,150879183	0	1	0	0	2	0	0	0
0,477512714	0,22045784	0,150879183	0	0	0	0	5	0	0	0
0,477512714	0,22045784	0,150879183	0	0	0	0	1	0	0	0
0,483291901	0,18868145	0,13177856	0	0	0	0	0	0	0	0
0,523256686	0,207056065	0,130154061	0	0	0	0	0	0	0	0
0,506019575	0,188268965	0,143830704	0	0	0	1	1	0	0	0
0,607450354	0,309253789	0,136751213	0	0	0	2	5	0	0	0
0,480046377	0,176840565	0,148533977	0	0	0	2	0	0	0	0
0,475225488	0,160451591	0,141746191	0	0	0	1	1	0	0	0
0,493255026	0,188055635	0,145772006	0	0	0	2	0	0	0	0
0,487332199	0,15848355	0,129952649	0	0	0	0	1	0	0	0
0,487332199	0,15848355	0,129952649	0	0	0	0	0	0	1	0
0,487332199	0,15848355	0,129952649	0	0	0	0	0	0	0	0
0,566769295	0,234296142	0,129777935	0	0	0	5	10	0	1	0
0,587828058	0,240677541	0,130606671	0	0	0	3	3	0	0	0

0,54030482	0,211533216	0,129165186	0	0	0	3	6	0	0	0
0,574046247	0,27621532	0,110703305	0	0	0	0	0	0	0	0
0,587854494	0,243195604	0,104683293	0	0	0	5	2	0	0	0
0,556533568	0,223368499	0,137785587	0	0	0	0	7	0	0	0
0,560447212	0,24739526	0,137495319	0	0	0	0	3	0	0	0
0,565409252	0,246972414	0,114903273	0	0	0	2	2	0	0	0
0,565409252	0,246972414	0,114903273	0	0	0	1	8	0	0	0
0,565409252	0,246972414	0,114903273	0	0	0	10	5	0	0	0
0,548496122	0,287897802	0,138894994	0	0	0	0	0	0	0	0
0,542771758	0,290736558	0,128593306	0	0	0	4	10	0	0	0
0,56814117	0,253206769	0,1291388	0	0	0	1	3	0	0	0
0,586801768	0,244233682	0,123841123	0	0	0	3	4	0	0	0
0,566185748	0,268802281	0,12847472	0	0	0	2	2	0	0	0
0,585498773	0,281516845	0,128223625	0	0	0	0	2	0	0	0
0,554437889	0,232474828	0,124210265	0	0	0	0	0	0	0	0
0,567760319	0,271192954	0,119127218	0	0	0	0	6	0	0	0
0,567760319	0,271192954	0,119127218	0	0	0	1	3	0	0	0
0,567760319	0,271192954	0,119127218	0	0	0	2	6	0	0	0
0,57221169	0,20821006	0,112406659	0	0	0	0	1	0	0	0
0,577213525	0,217877333	0,124052443	0	0	0	0	3	0	0	0
0,629838299	0,293050291	0,116168442	0	0	0	0	4	0	0	0
0,617320422	0,221252182	0,123665523	0	0	0	1	0	0	0	0
0,635562499	0,359088452	0,101905287	0	0	0	0	0	0	0	0
0,605586757	0,275709319	0,115121648	0	0	0	1	1	0	0	0
0,597970772	0,257807205	0,111116795	0	0	0	5	2	0	0	0
0,552676731	0,271236017	0,115091848	0	0	0	1	0	0	0	0
0,552676731	0,271236017	0,115091848	0	0	0	0	1	0	1	0
0,552676731	0,271236017	0,115091848	0	0	0	9	3	0	0	0
0,633147961	0,229460224	0,123738454	0	0	0	4	1	0	0	0

0,59486905	0,238163512	0,117251896	0	0	0	6	2	0	0	0
0,63724777	0,238577384	0,10119475	0	0	0	1	2	0	0	0
0,610275101	0,197396656	0,120118904	0	0	1	5	2	0	1	0
0,587459072	0,1868224	0,113926718	0	0	0	0	0	0	0	0
0,629934936	0,257433339	0,11890469	0	0	0	4	4	0	0	0
0,636062195	0,247931342	0,110053988	0	0	0	1	3	0	1	0
0,606304917	0,223988909	0,111551648	0	0	0	4	6	0	0	0
0,606304917	0,223988909	0,111551648	0	0	0	3	0	0	3	0
0,606304917	0,223988909	0,111551648	0	0	0	8	2	0	0	0
0,648417472	0,314346237	0,106183099	0	0	0	7	4	0	1	0
0,624346403	0,22878376	0,130350048	0	0	0	3	1	0	1	0
0,617540631	0,225272698	0,120550951	0	0	0	0	0	0	1	0
0,612145138	0,316672029	0,111412222	0	0	0	0	0	0	0	1
0,616842815	0,230967322	0,118623263	0	0	0	3	1	0	0	0
0,576903389	0,167029139	0,133618037	0	0	0	2	2	0	1	0
0,633720816	0,256014337	0,116172481	0	0	0	12	0	0	0	0
0,635113216	0,279370918	0,123339389	0	0	0	0	4	0	0	0
0,635113216	0,279370918	0,123339389	0	0	0	1	1	0	0	0
0,635113216	0,279370918	0,123339389	0	0	0	0	5	0	1	0
0,633578512	0,296388091	0,117686593	0	0	0	0	0	0	1	0
0,593405794	0,261472627	0,117212296	0	0	0	0	1	0	1	0
0,619003492	0,278804306	0,13282663	0	0	0	0	1	0	4	0
0,570989006	0,189961168	0,120383733	0	0	0	0	1	0	0	0
0,565378525	0,204006032	0,141566322	0	0	0	0	0	0	6	0
0,586219508	0,234633273	0,125251651	0	0	0	0	0	1	0	0
0,59706422	0,269891625	0,126342521	0	0	0	0	1	0	0	0
0,628818732	0,233809525	0,115062347	0	0	0	0	6	0	0	0
0,628818732	0,233809525	0,115062347	0	0	0	0	3	1	0	0
0,628818732	0,233809525	0,115062347	0	0	0	0	6	2	0	0

0,541059522	0,134119002	0,11611766	0	0	0	0	2	0	0	0
0,541359263	0,171912358	0,123928792	0	0	0	0	1	0	0	0
0,599063192	0,24939513	0,115181226	0	0	0	0	0	0	0	0
0,632940295	0,293579396	0,110722156	0	0	0	0	1	1	0	0
0,56750432	0,244086627	0,111441949	0	0	0	0	0	0	0	0
0,591272862	0,253930111	0,115413772	0	0	0	0	0	1	0	0
0,599657348	0,257077544	0,129711876	0	0	0	1	0	2	0	0
0,599657348	0,257077544	0,129711876	0	0	0	0	0	0	0	0
0,599657348	0,257077544	0,129711876	0	0	0	1	2	0	0	0
0,568092331	0,249293494	0,140288141	0	0	0	0	0	1	0	0
0,592088086	0,303892927	0,122435143	0	0	0	0	0	1	1	0
0,605144283	0,310718869	0,117904014	0	0	0	0	0	0	0	0
0,616939451	0,326531649	0,117588099	0	0	0	0	1	1	0	0
0,636114301	0,277298927	0,117340777	0	0	0	7	3	0	0	0
0,617562873	0,285271232	0,126457867	0	0	0	0	0	2	0	0
0,616441472	0,291431765	0,135792233	0	0	0	0	0	1	0	0
0,593711199	0,238403761	0,13240058	0	0	0	0	0	0	1	0
0,593711199	0,238403761	0,13240058	0	0	0	0	0	0	0	0
0,593711199	0,238403761	0,13240058	0	0	0	0	0	0	0	0
0,576752993	0,223263042	0,134156275	0	0	0	0	0	0	0	0
0,615581175	0,270238229	0,132149695	0	0	0	7	1	0	0	0
0,590489745	0,20716201	0,129385305	0	0	0	3	2	0	1	0
0,59713186	0,241369354	0,122346839	0	0	0	2	0	0	0	0
0,638868429	0,258196153	0,123297815	0	0	0	9	8	0	0	0
0,590286912	0,234132105	0,117247033	0	0	0	5	0	0	1	0
0,647815213	0,263399917	0,103348893	0	0	0	4	2	0	0	0
0,615605674	0,215583288	0,120757711	0	0	0	5	4	0	0	0
0,615605674	0,215583288	0,120757711	0	0	0	3	0	0	2	0
0,615605674	0,215583288	0,120757711	0	0	0	5	1	0	0	0

0,653454953	0,264644388	0,108042867	0	0	0	6	0	0	0	0
0,638926713	0,271979481	0,119885465	0	0	0	8	3	0	0	0
0,6482094	0,271773644	0,108560145	0	0	0	5	4	0	0	0
0,649744382	0,278016457	0,104819848	0	0	0	8	3	0	0	0
0,626299126	0,26897933	0,111865554	0	0	0	3	8	0	2	0
0,610424994	0,294055213	0,120053027	0	0	0	9	4	0	0	0
0,612415375	0,290793157	0,114684841	0	0	0	6	0	0	1	0
0,65384864	0,326693557	0,110095934	0	0	0	1	15	0	0	0
0,65384864	0,326693557	0,110095934	0	0	0	6	4	0	0	0
0,65384864	0,326693557	0,110095934	0	0	0	5	4	0	0	0
0,66453331	0,369056667	0,11776371	0	0	0	9	6	0	0	0
0,63929068	0,294313878	0,116416301	0	0	0	7	1	0	0	0
0,650501939	0,291358594	0,115145527	0	0	0	8	7	0	0	0
0,646989151	0,280879729	0,115997723	0	0	0	14	6	0	0	0
0,495954194	0,17432876	0,14092715	0	0	0	0	0	0	2	0
0,672832488	0,310661633	0,118777529	0	0	0	0	3	0	0	0
0,646760637	0,326448035	0,10462116	0	0	0	3	1	0	0	0
0,646760637	0,326448035	0,10462116	0	0	0	2	2	0	2	0
0,646760637	0,326448035	0,10462116	0	0	0	2	1	0	0	0
0,505962727	0,19365807	0,13982258	0	0	0	11	2	0	0	0
0,494555901	0,218154239	0,143765835	0	0	0	4	3	0	0	0
0,503151747	0,164409683	0,124303328	0	0	0	0	0	0	0	0
0,485276793	0,119844823	0,139344018	0	0	0	3	0	0	0	0
0,513454671	0,227526586	0,116531529	0	0	0	0	2	0	0	0
0,511077761	0,205320207	0,130569377	0	0	0	4	0	0	1	0
0,47477086	0,141309944	0,115852801	0	0	0	1	0	0	0	0
0,498091772	0,259522081	0,121740145	0	0	0	0	12	0	1	0
0,498091772	0,259522081	0,121740145	0	0	0	0	2	0	0	0
0,498091772	0,259522081	0,121740145	0	0	0	0	2	0	0	0

0,459539656	0,178789209	0,136553447	0	0	0	0	8	0	0	0
0,453661658	0,118834373	0,164945405	0	0	0	1	3	0	0	0
0,492743047	0,169367669	0,145687788	0	0	0	1	1	0	0	0
0,497289595	0,215899415	0,12931948	0	0	0	4	14	0	0	0
0,516512712	0,285544035	0,141983707	0	0	0	0	0	0	2	0
0,485806292	0,206736804	0,161351844	0	0	0	0	2	0	0	0
0,470051527	0,236151157	0,146318515	0	0	0	0	11	0	1	0
0,543919549	0,268022868	0,164316944	0	0	0	0	9	0	0	0
0,543919549	0,268022868	0,164316944	0	0	0	1	7	0	0	0
0,543919549	0,268022868	0,164316944	0	0	0	1	8	0	0	0
0,541108561	0,308662199	0,160732974	0	0	0	1	11	0	0	0
0,539982632	0,309260153	0,140976075	0	0	0	2	0	0	0	0
0,478062279	0,244583227	0,155580427	0	0	0	0	0	0	0	0
0,495374215	0,247184268	0,162030562	0	0	0	0	0	0	0	0
0,539046046	0,294453793	0,171392841	0	0	0	0	6	0	0	0
0,519865051	0,283643758	0,157512747	0	0	0	0	0	0	0	0
0,442270798	0,202968295	0,172010287	0	0	0	1	1	0	0	0
0,403351677	0,153471405	0,18180899	0	0	0	0	0	0	1	0
0,403351677	0,153471405	0,18180899	0	0	0	0	0	0	0	0
0,403351677	0,153471405	0,18180899	0	0	0	1	0	0	0	0
0,452170182	0,181805307	0,174954723	0	0	0	1	1	0	0	0
0,433068849	0,214458774	0,179464347	0	0	0	1	1	0	1	0
0,501140326	0,270051404	0,150582533	0	0	0	1	4	0	0	0
0,523496001	0,292698515	0,151176645	0	0	0	2	4	0	0	0
0,493120979	0,313977533	0,221576315	0	0	0	0	1	0	0	0
0,556365226	0,366406464	0,167757482	0	0	0	0	1	0	0	0
0,490826003	0,268270307	0,178102015	0	0	0	0	3	0	0	0
0,549075044	0,329560709	0,176948977	0	0	0	0	8	0	0	1
0,549075044	0,329560709	0,176948977	0	0	0	0	2	0	0	0

0,549075044	0,329560709	0,176948977	0	0	0	1	15	0	0	0
0,544227417	0,430194116	0,213253103	0	0	0	0	0	0	0	0
0,531785037	0,356768654	0,162953318	0	0	0	0	0	0	0	0
0,498743627	0,31399852	0,19730535	0	0	0	1	10	0	0	0
0,564524229	0,420221019	0,147199059	0	0	0	4	0	0	0	0
0,382869915	0,160530546	0,154495055	0	0	0	0	2	0	0	0
0,523026894	0,353104286	0,174655509	0	0	0	4	1	0	0	0
0,51344614	0,333111932	0,17716221	0	0	0	0	4	0	0	0
0,438803953	0,210532938	0,171485078	0	0	0	0	0	0	0	0
0,438803953	0,210532938	0,171485078	0	0	0	0	0	0	0	0
0,438803953	0,210532938	0,171485078	0	0	0	0	0	0	1	0
0,380844492	0,11506033	0,190107886	0	0	0	0	2	0	0	0
0,384964136	0,173535896	0,15577279	0	0	0	0	1	0	0	0
0,351646063	0,13009639	0,147276961	0	0	0	0	0	0	0	0

asychi	auscra	ausmod	ausstu	barlep	bocsyr	bumcir	callia	capite	caprel	cerato
0	0	15	4	0	0	0	0	0	0	0
0	0	24	6	0	0	1	0	0	0	1
0	0	0	5	0	0	0	0	0	0	1
0	0	25	4	0	0	0	0	0	0	7
0	0	0	0	0	0	0	0	0	0	0
0	0	1	4	0	0	0	0	0	0	0
0	0	0	2	0	1	0	0	0	0	0
0	0	0	0	0	0	0	0	0	0	0
0	0	0	0	0	0	0	0	0	0	0
0	0	0	0	0	0	5	0	0	0	0
0	0	0	0	0	0	0	0	0	0	0
0	0	0	0	0	0	1	0	0	0	0
0	0	0	0	0	0	1	0	0	0	0
0	0	0	0	0	0	0	0	0	0	0
0	0	0	0	0	2	1	0	0	0	0
0	0	0	0	0	0	0	0	0	0	0
0	0	0	1	0	2	3	0	0	0	0
0	0	0	0	0	0	0	0	0	0	0
0	0	0	0	0	0	4	0	0	0	0
0	0	0	0	0	0	2	0	0	0	0
0	0	0	0	0	0	0	0	0	0	0
0	0	0	0	0	1	3	0	0	0	0
0	0	9	4	0	0	0	0	0	0	0
0	0	0	3	0	0	0	0	0	0	1
0	0	0	1	0	0	1	0	0	0	0
0	0	0	0	0	0	1	0	0	0	0
0	0	0	0	0	0	0	0	0	0	0
0	0	0	0	0	0	0	0	0	0	0

0	0	0	0	0	1	0	0	0	0	0
1	0	0	0	0	0	0	0	0	0	0
1	0	0	0	0	0	0	0	0	0	0
0	0	0	0	0	0	0	0	0	0	0
0	0	0	0	0	0	1	0	0	0	0
0	0	0	2	0	1	0	0	0	0	0
0	0	0	1	1	0	1	0	0	0	0
0	0	0	0	0	0	0	0	0	0	0
0	0	9	40	0	1	0	0	0	0	0
0	0	0	3	0	0	0	0	0	0	0
0	0	0	3	0	0	0	0	0	0	0
0	0	0	0	0	0	0	0	0	0	0
0	0	0	1	0	2	0	0	0	0	0
0	0	0	1	0	1	0	0	0	0	0
0	0	6	5	0	0	16	0	1	0	0
0	0	0	5	0	0	0	0	0	0	0
0	0	0	1	0	0	0	0	0	0	0
0	0	5	3	0	0	1	0	0	0	0
0	0	17	28	0	0	0	0	0	0	0
0	0	16	21	0	0	1	0	0	0	0
0	0	0	1	0	0	1	0	0	0	0
0	0	0	3	0	0	3	0	1	0	0
0	0	0	9	0	2	3	0	0	0	0
0	0	0	0	0	2	0	0	0	0	0
0	0	0	0	0	0	1	0	0	0	0
0	0	0	0	0	0	0	0	0	0	0
0	0	0	1	0	0	1	0	0	0	0
0	0	0	0	0	0	0	0	0	0	0
0	0	0	1	0	0	0	0	0	0	0

0	0	0	5	0	0	2	0	0	0	0
0	0	0	1	0	1	2	0	0	0	0
0	0	0	3	0	0	1	0	0	0	0
0	0	0	3	0	0	3	0	0	0	0
0	0	0	1	0	0	0	0	0	0	0
0	0	0	2	0	1	9	0	0	0	0
0	0	0	0	0	8	3	0	0	0	0
0	0	0	0	0	0	2	0	0	0	0
0	0	0	1	0	4	0	0	0	0	0
0	0	0	0	0	0	2	0	0	0	0
0	0	0	0	0	0	4	0	0	0	0
0	0	0	1	0	0	1	0	0	0	0
0	0	0	0	0	0	3	0	0	0	0
0	0	0	0	0	0	24	0	0	0	0
0	0	0	0	0	0	4	0	0	0	0
0	0	0	0	0	0	0	0	0	0	0
0	0	0	0	0	0	0	0	0	0	0
0	0	0	0	0	0	3	0	0	0	0
0	0	0	2	0	0	0	0	0	0	0
0	0	0	0	0	0	1	0	0	0	0
0	0	0	0	0	0	1	0	0	0	0
0	0	1	12	0	0	0	0	0	0	0
0	0	0	4	0	0	0	0	0	0	0
0	0	0	10	0	0	0	0	1	0	0
0	0	0	1	0	2	6	0	0	0	0
0	0	0	0	0	0	1	0	0	0	0
0	0	0	3	0	1	17	0	0	0	0
0	0	0	2	0	0	2	0	0	0	0
0	0	0	0	0	0	1	0	0	0	0

0	0	0	0	0	2	3	0	0	0	0
0	0	1	0	0	0	3	0	0	0	0
0	0	0	0	0	0	0	0	0	0	0
0	0	0	0	0	1	2	0	0	0	0
0	0	0	11	0	0	0	0	0	0	0
0	1	0	10	0	0	0	0	0	0	0
0	0	6	8	0	0	0	0	0	0	0
0	0	0	18	0	0	0	0	0	0	0
0	0	0	1	0	0	2	0	0	0	0
0	0	0	4	0	12	5	0	0	0	0
0	0	0	0	0	0	5	0	0	0	0
0	0	0	1	0	0	2	0	1	0	1
0	0	0	2	0	0	1	0	0	0	0
0	0	0	4	0	0	0	0	2	0	6
0	0	0	12	0	0	0	0	0	0	0
0	0	0	6	0	0	0	0	0	0	2
0	0	0	4	0	0	0	0	0	0	0
0	0	0	0	0	0	0	0	0	0	0
0	0	0	0	0	0	0	0	0	0	0
0	0	0	0	0	0	0	0	0	0	0
0	0	0	0	0	0	0	0	0	0	0
0	0	0	0	0	1	0	0	0	0	0
0	0	0	0	0	7	1	0	0	0	0
0	0	0	0	0	35	5	0	0	0	0
0	0	0	0	0	0	25	0	1	0	0
0	0	0	1	0	4	4	0	0	0	0
0	0	0	3	0	1	4	0	0	0	0
0	0	0	1	0	0	3	0	0	0	0
0	0	0	2	0	1	9	0	0	0	0

0	0	0	0	0	0	1	0	0	0	0
0	1	1	4	0	0	0	0	1	0	0
0	0	15	14	0	0	0	0	0	0	0
0	0	3	8	0	0	0	0	0	0	1
0	0	20	11	0	0	1	0	0	0	1
0	0	6	9	0	0	0	0	0	0	0
0	0	12	2	0	0	0	0	0	0	0
0	0	42	9	0	0	0	0	0	0	0
0	0	7	3	0	0	0	0	0	0	2
0	0	0	4	0	0	0	0	0	0	0
0	0	0	5	0	0	0	0	0	0	0
0	0	0	0	0	1	0	0	1	0	0
0	0	0	0	0	0	0	0	0	0	0
0	0	0	0	0	0	1	0	1	0	0
0	0	0	0	0	0	0	0	0	0	0
0	0	0	1	0	0	0	0	1	0	0
0	1	0	0	0	0	0	0	0	0	0
0	0	0	0	0	0	0	0	0	0	0
0	0	0	1	0	0	1	0	2	0	0
0	0	0	0	0	0	0	0	2	0	0
0	0	0	0	0	0	0	0	0	0	0
0	0	0	1	0	0	1	0	0	0	0
0	0	0	1	0	0	0	0	0	0	1
0	0	0	1	0	0	0	0	0	0	0
0	0	2	3	0	0	0	0	0	0	4
0	0	0	0	0	0	1	0	1	0	0
0	0	0	0	0	0	0	0	0	0	0
0	0	0	3	0	0	0	0	0	0	0
0	0	0	5	0	0	0	0	0	0	0

0	0	0	7	0	0	0	0	0	0	0
0	0	0	7	0	0	0	0	0	0	0
0	0	4	11	0	0	0	0	0	0	2
0	0	7	11	0	0	0	0	0	0	1
0	0	0	4	0	0	0	0	0	0	0
0	0	7	5	0	0	0	0	0	0	1
0	0	1	6	0	0	0	0	0	0	2
0	0	0	2	0	1	0	0	0	0	1
0	0	0	0	0	0	0	0	0	0	0
0	0	0	3	0	0	0	0	0	0	0
0	0	0	1	0	0	1	0	1	0	0
0	0	0	0	0	0	0	0	0	0	1
0	0	16	6	0	0	2	0	0	0	5
0	0	2	4	0	0	0	0	0	0	1
0	0	0	5	0	0	0	0	0	0	0
0	0	1	4	0	0	0	0	1	0	0
0	0	12	14	0	0	0	0	1	0	0
0	0	0	9	0	0	1	0	2	0	0
0	0	1	3	0	0	0	0	0	0	0
0	0	11	29	0	0	0	0	0	0	0
0	0	13	18	0	0	0	0	0	0	0
0	1	0	9	0	0	0	0	0	0	0
1	0	4	28	0	0	0	0	1	0	0
0	1	60	32	0	0	0	0	2	0	0
0	0	11	2	0	0	0	0	0	0	0
0	0	40	2	0	0	0	0	0	0	1
0	0	1	0	0	0	1	0	0	0	0
0	0	0	1	0	2	0	0	0	0	0
0	0	8	4	0	0	0	0	0	0	0

0	0	41	5	0	0	0	0	0	0	3
0	0	0	1	0	0	0	0	0	0	0
0	0	0	3	0	0	0	0	0	0	0
0	0	1	6	0	0	0	0	0	0	1
0	0	0	1	0	0	0	0	0	0	0
0	0	0	4	0	0	0	0	0	0	0
0	0	0	6	0	0	1	0	0	0	1
0	0	0	0	0	0	0	0	0	0	0
0	0	1	3	0	0	0	0	0	0	0
0	0	0	5	0	0	1	0	0	0	0
0	0	0	3	0	0	0	0	0	0	0
0	0	50	7	0	0	0	0	0	0	0
0	0	0	3	0	0	0	0	0	0	0
0	0	11	7	0	0	0	0	0	0	0
0	0	5	3	0	0	0	0	0	0	0
0	0	0	5	0	0	0	0	0	0	0
0	0	0	0	0	0	0	0	0	0	0
0	0	0	4	0	0	0	0	0	0	1
0	0	0	3	0	0	0	0	0	0	1
0	0	0	3	0	0	0	0	0	0	0
0	0	14	3	0	0	0	0	0	0	1
0	0	2	7	0	0	0	0	0	0	0
0	0	0	9	0	0	0	0	0	0	0
0	0	0	7	0	0	0	0	0	0	1
0	0	2	7	0	0	0	0	0	0	1
0	0	3	6	0	0	0	0	0	0	0
0	0	0	13	0	0	0	0	0	0	0
0	0	0	4	0	0	1	0	1	0	2
0	0	0	0	0	0	0	0	0	0	0

0	0	0	3	0	0	0	0	0	0	4
0	0	0	1	0	0	0	0	0	0	3
0	0	0	0	0	0	0	0	0	0	0
0	0	0	0	0	0	0	0	0	0	3
0	0	0	0	0	0	0	0	0	0	4
0	0	0	1	0	0	0	0	0	0	2
0	0	0	0	0	0	0	0	0	0	0
0	0	0	0	0	0	0	0	0	0	1
0	0	0	0	0	0	0	0	0	0	3
0	0	0	1	0	0	0	0	0	0	0
0	0	0	2	0	0	0	0	0	0	0
0	0	1	3	0	0	0	0	0	0	0
0	0	49	1	0	0	0	0	0	0	0
0	0	15	1	0	0	0	0	0	0	2
0	0	0	0	0	0	0	0	0	0	1
0	0	11	3	0	0	0	0	0	0	1
0	0	0	5	0	0	0	0	0	0	0
0	0	0	2	0	0	0	0	0	0	3
0	0	0	2	0	0	0	0	0	0	0
0	0	0	2	0	0	0	0	0	0	0
0	0	0	2	0	0	0	0	0	0	0
0	0	6	3	0	0	0	0	0	0	1
0	0	0	2	0	0	0	0	0	0	1
0	0	0	1	0	0	0	0	0	0	1
0	0	6	4	0	0	0	0	0	0	0
0	0	0	2	0	1	0	0	0	0	0
0	1	0	2	0	0	0	0	0	0	0
0	0	0	0	0	0	0	0	0	0	1
0	0	0	1	0	0	0	0	0	0	0

0	0	0	0	0	0	0	0	0	0	0
0	0	0	2	0	0	0	0	0	0	3
0	0	0	0	0	0	0	0	0	0	0
0	0	5	3	0	0	0	0	0	0	2
0	0	3	4	0	0	0	0	0	0	0
0	0	0	0	0	0	0	0	0	0	0
0	0	10	5	0	0	0	0	0	0	0
0	0	3	2	0	0	0	0	0	0	0
0	0	0	1	0	0	0	0	0	0	0
0	0	0	3	0	0	0	0	0	0	1
0	0	0	0	0	0	0	0	0	0	0
0	0	0	2	0	0	0	0	0	0	0
0	0	0	2	0	0	0	0	0	0	0
0	0	0	1	0	0	0	0	0	0	0
0	0	0	1	0	0	0	0	0	0	0
0	0	0	2	0	0	0	0	0	0	0
0	0	0	1	0	1	0	0	0	0	0
0	0	0	1	0	0	0	0	0	0	0
0	0	0	2	0	0	0	0	0	0	0
0	0	0	1	0	0	0	0	0	0	0
0	0	0	0	0	0	0	0	0	0	0
0	0	0	2	0	0	0	0	0	0	0
0	0	0	1	0	2	1	0	0	0	0
0	0	0	1	0	0	0	0	0	0	0
0	0	0	0	0	0	0	0	0	0	0
0	0	0	2	0	0	0	0	0	0	0
0	0	0	2	0	1	0	0	0	0	0
0	0	0	0	0	1	1	0	0	0	0
0	0	0	0	0	0	1	0	0	0	0

0	0	0	0	0	0	0	0	0	0	0
0	0	0	0	0	0	2	0	0	0	0
0	0	0	0	0	0	0	0	0	0	0
0	0	0	0	0	3	1	0	0	0	0
0	0	0	0	1	0	0	0	0	0	0
0	0	0	0	0	2	0	0	0	0	0
0	0	0	0	0	0	0	0	0	0	0
0	0	0	0	0	0	1	0	0	0	0
0	0	0	0	0	1	1	0	0	0	0
0	0	0	1	0	0	0	0	0	0	0
0	0	0	0	0	1	0	0	0	0	0
0	0	0	0	0	0	0	0	0	0	0
0	0	0	0	0	0	0	0	0	0	0
0	0	0	0	0	0	4	0	0	0	0
0	0	0	0	0	0	1	0	0	0	0
0	0	0	0	0	0	0	0	0	0	0
2	0	0	0	0	0	3	0	2	0	0
0	0	0	0	0	19	0	0	0	0	0
0	0	0	0	0	0	1	0	0	0	0
0	0	0	0	0	0	1	0	0	0	0
0	0	0	0	0	0	2	0	0	0	0
0	0	0	0	0	77	5	0	0	0	0
0	0	0	0	0	0	7	0	0	0	0
0	0	0	0	0	0	1	0	0	0	0
0	0	0	0	0	0	3	0	0	0	0
0	0	0	0	0	1	5	1	0	0	0
0	0	0	0	0	1	2	0	0	0	0
0	0	0	0	0	0	0	0	0	0	0
0	0	0	0	0	10	12	0	1	0	0

0	0	0	0	0	0	4	0	0	0	0
0	0	0	0	0	56	10	0	0	0	0
0	0	0	0	0	0	0	0	0	0	0
0	0	0	0	0	96	11	0	0	0	0
0	0	0	0	0	77	5	0	1	0	0
0	0	0	0	0	3	5	0	0	0	0
0	0	0	0	0	0	9	0	0	0	0
0	0	0	0	0	0	0	0	0	0	0
0	0	0	0	0	0	4	0	0	0	0
0	0	0	0	0	0	7	2	0	0	0
0	0	0	0	0	0	7	0	0	0	0
0	0	0	0	0	1	0	0	0	1	0
0	0	0	0	0	1	4	0	0	0	0
0	1	0	0	0	1	4	0	0	0	0
0	0	0	0	1	2	3	0	1	0	0
0	0	0	0	0	0	4	0	0	0	0
0	0	0	0	0	0	3	0	0	0	0
0	0	0	1	1	0	4	0	0	0	0
1	0	0	0	0	0	2	0	1	0	0
1	0	0	0	0	0	3	0	0	0	0
0	0	0	0	0	0	2	0	0	0	0
0	0	0	0	0	0	4	0	0	0	0
0	0	0	0	0	0	1	0	0	0	0
1	0	0	0	0	2	12	0	0	0	0
0	0	0	0	0	8	4	0	0	0	0
0	0	0	0	0	0	4	0	0	0	0
0	0	0	0	0	0	3	0	0	0	0
0	0	0	0	0	0	0	0	0	0	1
0	0	0	0	0	0	7	0	0	0	0

0	0	0	0	0	0	2	0	0	0	0
0	0	0	0	0	0	1	0	0	0	0
0	0	0	0	0	0	0	0	0	0	0
1	0	0	0	0	0	1	0	0	0	0
0	0	0	0	0	0	0	0	0	0	0
0	0	0	0	0	0	0	0	0	0	0
0	0	0	0	0	0	2	0	0	0	0
0	0	0	0	0	0	5	0	0	0	0
0	0	0	1	0	0	7	0	0	0	0
0	0	0	1	0	0	2	0	0	0	0
0	0	0	0	0	0	0	0	0	0	0
0	0	0	1	0	0	3	0	0	0	0
0	0	0	1	0	0	2	0	0	0	0
0	0	0	0	0	1	0	0	0	0	0
0	0	0	1	0	0	1	0	0	0	0
0	0	0	0	0	0	4	0	0	0	0
0	0	0	0	0	0	0	0	0	0	0
0	0	0	0	0	1	0	0	0	0	0
0	0	0	1	0	0	0	0	0	0	0
0	0	0	2	0	0	0	0	0	0	0
0	0	0	1	0	0	0	0	0	0	0
0	0	0	1	0	0	2	0	0	0	0
0	0	0	0	0	0	0	0	0	0	0
0	0	0	1	0	0	0	0	0	0	0
0	0	0	0	0	0	1	0	0	0	0
0	0	0	0	0	0	1	0	0	0	1
0	0	0	0	0	0	0	0	0	0	0
0	0	0	0	0	0	0	0	0	0	0
0	0	6	1	0	0	0	0	0	0	0

0	0	0	0	0	0	1	0	0	0	0
0	0	0	1	0	0	1	0	0	0	0
0	0	0	2	0	0	1	0	0	0	0
0	0	16	4	0	0	0	0	0	0	0
0	0	0	2	0	0	0	0	0	0	0
0	0	0	1	0	1	2	0	0	0	0
0	0	0	3	0	0	1	0	0	0	0
0	1	0	0	0	0	0	0	0	0	0
0	0	0	0	0	0	0	0	0	0	0
0	0	0	1	0	0	0	0	0	0	0
0	0	0	1	0	0	0	0	0	0	0
0	0	0	5	0	0	0	0	0	0	0
0	0	15	7	0	0	0	0	0	0	0
0	0	11	2	0	0	0	0	0	0	0
0	0	0	2	0	0	0	0	0	0	0
0	0	28	6	0	0	0	0	1	0	0
0	0	9	0	0	0	0	0	0	0	0
0	0	0	3	0	0	0	0	0	0	0
0	0	0	2	0	0	0	0	0	0	0
0	0	0	3	0	0	0	0	0	0	0
0	0	4	10	0	0	0	0	0	0	0
0	0	0	1	0	0	0	0	0	0	0
0	0	0	2	0	0	0	0	0	0	0
0	0	0	1	0	0	0	0	0	0	0
0	0	16	4	0	0	0	0	1	0	0
0	0	0	1	0	0	0	0	0	0	0
0	0	0	2	0	0	0	0	0	0	0
0	0	0	0	0	0	0	0	0	0	0
0	0	0	4	0	0	0	0	0	0	0

0	0	0	5	0	0	0	0	0	0	0
0	0	0	2	0	0	0	0	0	0	0
0	0	0	2	0	0	0	0	0	0	0
0	0	0	0	0	0	0	0	0	0	0
0	0	0	0	0	0	0	0	0	0	0
0	0	0	1	0	0	0	0	0	0	0
0	0	0	2	0	0	0	0	0	0	0
0	0	0	0	0	0	0	0	0	0	0
0	0	0	0	0	0	0	0	0	0	0
0	0	0	0	0	0	0	0	0	0	0
0	0	0	1	0	0	0	0	0	0	2
0	0	0	0	0	0	0	0	0	0	0
0	0	0	0	0	0	0	0	0	0	0
0	0	0	0	0	0	0	0	0	0	0
0	0	0	2	0	0	0	0	1	0	0
0	0	2	1	0	0	0	0	0	0	2
0	0	0	0	0	0	0	0	0	0	2
0	0	0	0	0	0	1	0	0	0	6
0	0	0	0	0	0	0	0	0	0	3
0	0	0	1	0	0	0	0	0	0	3
0	0	0	0	0	0	0	0	0	0	3
0	0	0	0	0	0	0	0	0	0	8
0	0	0	1	0	0	0	0	0	0	3

asychi	auscra	ausmod	ausstu	barlep	bocsyr	bumcir	callia	capite	caprel	cerato
0	0	0	0	0	0	0	0	0	0	0
0	0	0	1	0	1	0	0	0	0	0
0	0	0	0	0	0	0	0	0	0	0
0	0	0	1	0	0	0	0	0	0	0

0	0	0	0	0	0	0	0	1	0	0
0	0	0	2	0	1	0	0	0	0	0
0	0	0	0	0	0	0	0	0	0	0
0	0	0	0	0	0	0	0	0	0	0
0	0	0	1	0	0	0	0	0	0	0
0	0	0	0	0	0	0	0	0	0	0
0	0	0	0	0	0	0	0	0	0	0
0	0	0	0	0	1	0	0	0	0	0
0	0	0	0	0	1	0	0	0	0	0
0	0	0	0	0	1	2	0	0	1	0
0	0	0	0	0	0	0	0	0	0	0
1	0	0	0	0	0	0	0	0	0	0
0	0	0	0	0	1	0	0	0	0	0
0	0	0	0	0	1	0	0	0	0	0
0	0	0	0	0	0	0	0	0	0	0
1	0	0	0	0	6	0	0	0	0	0
0	0	0	0	0	0	0	0	0	0	0
0	0	0	2	0	0	0	0	0	0	0
0	0	0	2	0	0	0	0	0	0	0
0	0	0	2	0	0	1	0	0	0	0
0	0	0	1	0	1	1	0	0	0	0
0	0	1	1	0	0	0	0	0	0	0
0	0	0	0	0	1	0	0	0	0	0
0	0	0	2	0	0	0	0	0	0	0
0	0	0	1	0	0	0	0	0	0	0
0	0	0	1	0	0	0	0	0	0	0
0	1	0	3	0	0	0	0	0	0	0
0	0	0	26	0	0	0	0	0	0	0
0	0	0	2	0	0	0	0	0	0	0

0	0	0	3	0	0	0	0	0	0	0
0	0	0	3	0	0	0	0	0	0	0
0	0	0	8	0	0	0	0	0	0	0
0	0	0	5	0	0	0	0	0	0	0
0	0	0	3	0	0	0	0	0	0	0
0	0	0	10	0	0	0	0	0	0	0
0	0	0	24	0	0	0	0	0	0	0
0	0	0	12	0	1	0	0	0	0	0
0	0	0	35	0	0	0	0	0	0	0
0	0	0	19	0	1	0	0	0	0	0
0	0	0	15	0	0	0	0	0	0	0
0	0	1	33	0	1	0	0	0	0	0
0	0	1	12	0	0	0	0	0	0	0
0	0	0	33	0	0	0	0	0	0	1
0	0	0	25	0	0	0	0	0	0	0
0	0	1	16	0	1	0	0	0	0	0
0	0	0	25	0	1	0	0	1	0	0
0	0	0	40	0	0	0	0	0	0	0
0	1	0	18	0	0	0	0	0	0	0
0	0	0	37	0	0	0	0	0	0	0
0	0	0	28	0	0	0	0	0	0	0
0	0	0	45	0	0	0	0	0	0	0
0	0	0	38	0	0	0	0	0	0	0
0	0	0	37	0	0	0	0	0	0	0
0	0	0	9	0	0	0	0	0	0	0
0	0	0	29	0	1	0	0	0	0	0
0	0	0	8	0	0	0	0	0	0	0
0	0	0	10	0	0	0	0	0	0	0
0	0	0	28	0	2	0	0	0	0	0

0	0	0	18	0	0	0	0	0	0	0
0	0	0	18	0	0	0	0	0	0	0
0	0	0	1	0	0	0	0	0	0	0
0	0	0	4	0	0	0	0	0	0	0
0	0	0	3	0	0	0	0	0	0	0
0	0	0	3	0	0	0	0	0	0	0
0	0	0	21	0	0	0	0	0	0	0
0	0	0	19	0	0	0	0	0	0	0
0	0	0	9	0	0	0	0	0	0	0
0	0	0	7	0	0	2	0	0	0	0
0	0	0	14	0	1	0	0	0	0	0
0	0	0	14	0	0	0	0	0	0	0
0	0	0	5	0	0	0	0	0	0	0
0	0	0	10	0	0	0	0	0	0	0
0	1	0	11	0	0	0	0	0	0	0
0	0	0	9	0	0	0	0	0	0	0
0	0	0	9	0	0	0	0	0	0	0
0	0	0	2	0	0	0	0	0	0	0
0	0	0	6	0	0	0	0	0	0	0
0	0	0	2	0	2	0	0	0	0	0
0	1	0	6	0	0	0	0	0	0	0
0	0	0	3	0	0	0	0	0	0	0
0	0	0	9	0	0	0	0	0	0	0
0	0	0	2	0	1	0	0	0	0	0
0	0	0	1	0	5	0	0	0	0	0
0	0	0	2	0	0	0	0	0	0	0
0	0	0	0	0	1	0	0	0	0	0
0	0	0	0	0	0	0	0	0	0	0
0	0	0	0	0	1	0	0	0	0	0

0	0	0	0	0	0	0	0	0	0	0
0	0	0	0	0	6	0	0	0	0	0
0	0	0	1	0	1	0	0	0	0	0
0	0	0	0	0	1	0	0	0	0	0
0	0	0	1	0	0	0	0	0	0	0
0	0	0	0	0	0	0	0	0	0	0
0	0	0	0	0	0	0	0	0	0	0
0	0	0	0	0	1	0	0	0	0	0
1	0	0	0	0	0	0	0	0	0	0
0	0	0	0	0	2	1	0	0	0	0
0	0	0	0	0	0	0	0	0	0	0
0	0	0	0	0	4	0	0	0	0	0
2	0	0	0	0	3	1	0	0	0	0
0	0	0	0	0	0	1	0	1	0	0
1	0	0	0	0	0	0	0	0	0	0
0	0	0	1	0	1	1	0	0	0	0
0	0	0	0	0	1	1	0	0	0	0
0	0	0	0	0	0	1	0	0	0	0
0	0	0	0	0	1	0	0	0	0	0
0	0	0	1	0	3	1	0	0	0	1
0	0	0	0	0	0	0	0	0	0	0
0	0	0	0	0	2	0	0	0	0	0
0	0	0	1	0	1	4	0	0	0	1
1	0	0	0	0	0	0	0	1	0	0
0	0	0	0	0	1	0	0	0	0	0
0	0	0	0	0	3	0	0	0	0	0
0	0	0	0	0	3	0	0	0	0	0
0	0	0	0	0	1	1	0	0	0	0
0	0	0	0	0	2	0	0	0	0	0

0	0	0	1	0	4	0	0	0	0	0
0	0	0	0	0	2	0	0	1	0	0
0	0	0	0	0	2	1	0	0	0	0
0	0	0	0	0	3	0	0	0	0	0
0	0	0	1	0	3	3	0	0	0	0
0	0	0	1	0	0	0	0	0	0	0
0	0	0	0	0	1	0	0	0	0	0
0	0	0	1	0	0	0	0	0	0	0
0	0	0	1	0	0	0	0	0	0	0
0	0	0	1	0	1	1	0	0	0	0
0	0	0	0	0	2	0	0	0	0	0
0	0	0	7	0	1	0	0	0	0	0
0	0	0	1	0	3	0	0	0	0	0
0	0	0	0	0	7	0	0	0	0	0
0	0	0	0	0	0	0	0	0	0	0
0	0	0	1	0	0	1	0	0	0	0
0	0	0	2	0	1	0	0	0	0	0
0	0	0	1	0	9	0	0	0	0	0
0	0	0	1	0	0	0	0	0	0	0
0	0	0	3	0	0	0	0	0	0	0
0	0	0	5	0	2	0	0	0	0	0
0	0	0	9	0	0	0	0	0	0	0
0	0	0	8	0	3	0	0	0	0	0
0	0	0	9	0	2	1	0	0	0	0
0	0	0	5	0	2	1	0	0	0	0
0	0	0	12	0	0	0	0	0	0	0
0	0	0	8	0	0	0	0	0	0	0
0	0	0	7	0	0	0	0	0	0	0
0	0	0	31	0	0	1	0	0	0	0

0	0	0	3	0	0	0	0	0	0	0
0	0	0	13	0	0	0	0	0	0	0
0	0	0	15	0	0	0	0	0	0	0
0	0	0	22	0	1	0	0	0	0	0
0	0	0	34	0	0	0	0	0	0	0
0	0	0	7	0	0	0	0	0	0	0
0	0	0	15	0	1	0	0	0	0	0
0	0	0	1	0	0	0	0	0	0	0
0	0	0	4	0	0	0	0	0	0	0
0	0	1	23	0	0	0	0	0	0	0
0	0	0	6	0	0	0	0	0	0	0
0	0	0	16	0	0	0	0	0	0	0
0	0	0	33	0	0	0	0	0	0	0
0	0	0	6	0	0	0	0	0	0	0
0	0	0	22	0	0	0	0	0	0	0
0	0	0	12	0	0	0	0	0	0	0
0	0	1	37	0	0	0	0	0	0	0
0	0	0	20	0	0	0	0	0	0	0
0	0	0	25	0	0	0	0	0	0	0
0	0	0	29	0	0	0	0	0	0	0
0	0	0	25	0	1	0	0	0	0	0
0	0	0	22	0	1	0	0	0	0	0
0	0	0	7	0	0	0	0	1	0	0
0	0	0	42	0	3	0	0	0	0	0
0	0	0	12	0	0	0	0	0	0	0
0	0	0	7	0	1	0	0	0	0	0
0	0	0	17	0	1	0	0	0	0	0
0	0	0	25	0	0	0	0	0	0	0
0	0	0	21	0	0	0	0	0	0	0

0	0	0	0	0	0	0	0	0	0	0
0	0	0	0	0	0	0	0	0	0	0
0	0	0	0	0	1	0	0	0	0	0
0	0	0	2	0	1	0	0	0	0	0
0	0	0	4	0	0	0	0	0	0	0
0	0	0	7	0	1	0	0	0	0	0
0	0	0	12	0	0	0	0	0	0	0
0	0	0	10	0	0	0	0	0	0	0
0	0	1	7	0	0	1	0	0	0	0
0	0	0	4	0	0	0	0	0	0	0
0	0	0	4	0	0	0	0	0	0	0
0	0	0	0	0	0	0	0	0	0	0
0	1	0	2	0	0	0	0	0	0	0
0	0	0	4	0	2	0	0	0	0	0
0	0	0	3	0	0	0	0	0	0	0
0	0	0	0	0	0	0	0	0	0	0
0	0	0	0	0	1	1	0	0	0	0
0	0	0	1	0	1	0	0	0	0	0
0	0	0	0	0	0	0	0	0	0	0
0	0	0	1	0	0	0	0	0	0	0
0	0	0	2	0	0	0	0	0	0	0
0	0	0	1	0	0	0	0	0	0	0
0	0	0	0	0	0	0	0	0	0	0
0	0	0	1	0	0	0	0	0	0	0
0	0	0	2	0	0	0	0	0	0	0
0	0	0	3	0	2	0	0	0	0	1
0	0	0	1	0	0	0	0	0	0	0
0	0	0	2	0	0	0	0	0	0	0
0	0	0	3	0	0	0	0	0	0	0

0	0	0	1	0	0	0	0	0	0	0
0	0	0	7	0	0	0	0	0	0	0
0	0	0	6	0	1	0	0	0	0	0
0	0	0	8	0	0	0	0	0	0	0
0	0	1	16	0	0	0	0	0	0	0
0	0	0	7	0	0	0	0	0	0	0
0	0	0	5	0	0	0	0	0	0	0
0	0	0	3	0	0	0	0	0	0	0
0	0	0	4	0	0	0	0	1	0	0
0	0	0	4	0	0	0	0	0	0	0
0	0	0	5	0	0	0	0	0	0	0
0	0	1	16	0	0	0	0	0	0	0
0	0	0	9	0	0	0	0	0	0	0
0	0	1	22	0	1	0	0	0	0	0
0	0	0	21	0	0	0	0	0	0	0
0	0	0	13	0	0	0	0	0	0	0
0	0	0	13	0	0	0	0	0	0	0
0	0	0	10	0	0	0	0	0	0	0
0	0	0	29	0	0	0	0	0	0	0
0	0	0	7	0	0	0	0	0	0	0
0	0	0	49	0	0	0	0	1	0	0
0	0	0	66	0	0	0	0	0	0	0
0	1	0	51	0	0	0	0	0	0	0
0	0	0	10	0	0	0	0	0	0	0
0	0	0	38	0	0	0	0	0	0	0
0	0	0	16	0	0	0	0	0	0	0
0	0	0	38	0	0	0	0	0	0	0
0	0	0	19	0	2	0	0	0	0	0
0	0	0	32	0	1	0	0	0	0	0

0	0	0	38	0	0	0	0	0	0	0
0	0	0	32	0	0	1	0	0	0	0
0	0	0	39	0	0	1	0	0	0	0
0	0	0	15	0	0	0	0	1	0	0
0	0	0	40	0	0	0	0	0	0	0
0	0	0	11	0	0	0	0	0	0	0
0	1	0	22	0	0	0	0	0	0	0
0	0	0	46	0	0	0	0	0	0	0
0	0	0	43	0	0	0	0	0	0	0
0	0	0	17	0	0	0	0	0	0	0
0	0	0	16	0	0	0	0	0	0	0
0	0	0	15	0	1	1	0	0	0	0
0	0	0	26	0	0	0	0	0	0	0
0	0	0	11	0	0	1	0	0	0	0
0	0	0	25	0	0	0	0	0	0	0
0	0	0	5	0	0	0	0	0	0	0
0	0	0	39	0	0	0	0	0	0	0
0	0	0	17	0	0	0	0	0	0	0
0	0	0	15	0	1	0	0	0	0	0
0	0	0	6	0	0	0	0	0	0	0
0	0	0	16	0	0	1	0	0	0	0
0	0	0	11	0	1	0	0	0	0	0
0	0	0	8	0	4	0	0	0	0	0
0	0	0	8	0	0	1	0	0	0	0
0	0	0	7	0	0	0	0	0	0	0
0	0	0	0	0	2	0	0	0	0	0
0	0	0	1	0	8	1	0	0	0	0
0	0	0	5	0	1	0	0	0	0	0
0	0	0	4	0	2	0	0	0	0	0

0	0	0	0	0	1	0	0	0	0	0
0	0	0	3	0	1	0	0	0	0	0
2	0	0	0	0	0	1	0	0	0	0
0	0	0	0	0	1	0	0	0	0	0
0	0	0	0	0	0	1	0	0	0	0
0	0	0	2	0	0	2	0	0	0	0
0	0	0	0	0	8	1	0	0	0	0
0	0	0	1	0	1	2	0	0	0	0
0	0	0	1	0	6	0	0	0	0	0
0	0	0	1	0	7	1	0	0	0	0
0	0	0	0	0	3	2	0	0	0	0
0	0	0	1	0	4	1	0	0	0	0
0	0	0	0	0	2	0	0	0	0	0
0	0	0	0	0	2	2	0	0	0	0
0	0	0	0	0	8	0	0	0	0	0
0	0	0	0	0	11	2	1	0	0	0
0	0	0	0	0	2	0	0	0	0	0
0	0	0	0	0	7	1	0	0	0	0
0	0	0	0	0	3	0	0	0	0	0
0	0	0	0	0	6	1	0	0	0	0
0	0	0	0	0	2	3	0	0	0	0
0	0	0	0	0	1	0	0	0	0	0
0	0	0	0	0	10	1	0	0	0	0
0	0	0	0	0	1	0	0	0	0	0
0	0	0	0	1	0	0	0	0	0	0
0	0	0	0	0	3	0	0	0	0	0
0	0	0	0	0	0	0	0	0	0	0
0	0	0	0	0	1	2	0	0	0	0
0	0	0	0	0	2	0	0	0	0	0

0	0	0	0	0	0	1	0	0	0	0
0	0	0	0	0	1	0	0	1	0	0
0	0	0	0	0	0	0	0	0	0	0
0	0	0	0	0	1	0	0	1	0	0
0	0	0	0	0	0	0	0	0	0	0
0	0	0	0	0	5	1	0	0	0	0
0	0	0	0	0	1	0	0	1	0	0
0	0	0	0	0	3	0	0	0	0	0
0	0	0	92	0	0	0	0	0	0	0
0	0	0	0	0	0	0	0	0	0	0
0	0	0	0	0	0	0	0	0	0	0
0	0	0	0	0	1	0	0	0	0	0
0	0	0	0	0	0	0	0	0	0	0
0	0	0	0	0	2	0	0	0	0	0
0	0	0	0	0	1	0	0	0	0	0
0	0	0	0	0	0	0	0	0	0	0
0	0	0	0	0	0	1	0	0	0	0
0	0	0	0	0	0	3	0	0	0	0
0	0	0	0	0	1	0	0	0	0	0
0	0	0	0	0	0	0	0	0	0	0
0	0	0	0	0	0	1	0	0	0	0
0	0	0	0	0	1	0	0	0	0	0
0	0	0	0	0	1	0	0	0	0	0
0	0	0	1	0	0	0	0	0	0	0
0	0	0	0	0	2	0	0	0	0	0
0	0	0	0	0	3	3	0	0	0	0
0	0	0	1	0	4	0	0	0	0	0
0	0	0	1	0	3	0	0	0	0	0
0	0	0	0	0	2	2	0	0	0	0

0	0	0	0	0	2	0	0	1	0	0
0	0	0	0	0	0	2	0	1	0	0
0	0	0	0	0	1	0	0	0	0	0
0	0	0	0	0	2	4	0	0	0	0
1	0	0	0	0	3	2	0	0	0	0
0	0	0	4	0	7	5	0	0	0	0
0	0	0	0	0	0	2	0	0	0	0
0	0	0	0	0	4	4	0	0	0	0
0	0	0	5	0	0	0	0	0	0	0
0	0	0	1	0	1	2	0	0	0	0
0	0	0	0	0	2	0	0	0	0	0
0	0	0	1	0	1	2	0	0	0	0
0	0	0	2	0	4	2	0	0	0	0
0	0	0	1	0	0	4	0	0	0	0
0	0	0	5	0	1	1	0	0	0	0
0	0	0	9	0	0	3	0	0	0	0
0	0	0	4	0	2	0	0	0	0	0
0	0	0	13	0	0	0	0	0	0	0
0	0	0	20	0	0	1	0	0	0	0
0	0	0	9	0	3	1	0	0	0	0
0	0	0	17	0	3	0	0	0	0	0
0	0	0	58	0	2	1	0	0	0	0
0	0	0	29	0	2	1	0	0	0	0
0	0	0	37	0	1	0	0	0	0	0
0	0	0	20	0	0	0	0	1	0	0
0	0	0	17	0	0	0	0	0	0	0
0	0	0	20	0	0	0	0	0	0	0
0	0	0	28	0	0	0	0	0	0	0
0	0	0	16	0	0	0	0	0	0	0

0	0	0	10	0	0	0	0	0	0	0
0	0	0	9	0	0	1	0	0	0	0
0	0	0	8	0	0	0	0	0	0	0
0	1	0	6	0	0	0	0	0	0	0
0	0	0	18	0	0	0	0	0	0	0
0	0	0	4	0	0	0	0	0	0	0
0	0	0	12	0	0	0	0	0	0	0
0	0	0	19	0	0	0	0	0	0	0
0	0	0	10	0	0	0	0	0	0	0
0	0	0	45	0	1	0	0	0	0	0
0	0	1	15	0	0	0	0	0	0	0
0	0	0	23	0	0	0	0	0	0	0
0	0	0	29	0	0	0	0	0	0	0
0	1	0	27	0	0	1	0	0	0	0
0	0	0	50	0	0	0	0	0	0	0
0	0	0	39	0	0	0	0	4	0	0
0	0	1	67	0	0	0	0	0	0	0
0	0	0	37	0	0	0	0	0	0	0
0	0	0	48	0	1	0	0	0	0	0
0	1	0	64	0	0	0	0	0	0	0
0	0	1	50	0	1	0	0	0	0	0
0	0	1	60	0	0	0	0	0	0	0
0	0	1	24	0	0	0	0	0	0	0
0	0	0	23	0	0	0	0	0	0	0
0	1	0	43	0	0	0	0	0	0	0
0	0	0	42	0	0	0	0	0	0	0
0	0	0	34	0	0	0	0	0	0	0
0	0	0	20	0	0	0	0	0	0	0
0	0	1	20	0	0	0	0	0	0	0

0	0	0	70	0	0	0	0	0	0	0
0	0	0	29	0	0	0	0	0	0	0
0	0	0	20	0	0	1	0	0	0	0
0	0	0	22	0	0	0	0	0	0	0
0	0	0	26	0	0	0	0	0	0	0
0	0	0	19	0	0	0	0	0	0	0
0	0	0	17	0	0	0	0	0	0	0
0	0	0	0	0	1	0	0	0	0	0
0	0	0	21	0	0	0	0	0	0	0
0	0	0	0	0	3	0	0	0	0	0
0	0	0	6	0	0	0	0	0	0	0
0	1	0	22	0	0	0	0	0	0	0
0	0	0	2	0	0	0	0	0	0	0
0	0	0	12	0	0	0	0	1	0	0
0	1	0	15	0	0	0	0	0	0	0
0	0	0	7	0	0	0	0	0	0	0
0	0	2	6	0	0	0	0	0	0	0
0	0	0	15	0	0	0	0	0	0	0
0	0	0	5	0	0	0	0	0	0	0

ausmod	ausstu	bocsyr	bts	capite	cerato	chaeto	chigla	collem	comgla	comisp
0	0	0	0	0	3	0	0	0	1	0
0	1	0	0	0	2	0	0	1	0	0
0	0	0	0	2	8	0	0	2	0	0
0	7	0	0	0	11	0	0	1	0	0
0	0	0	0	0	2	0	0	0	0	0
0	0	0	0	0	4	0	0	0	1	0
0	1	0	0	0	2	0	0	0	0	0
0	3	0	0	0	0	0	0	8	0	0

0	0	0	0	0	8	0	0	0	0	0
0	1	0	0	0	1	0	0	1	0	0
0	5	0	0	0	4	0	0	0	1	0
0	17	0	0	0	6	0	0	0	0	0
0	19	0	0	0	5	0	0	0	0	0
0	5	0	0	0	4	0	0	0	0	0
0	18	0	0	0	0	0	0	1	0	0
0	10	0	0	0	3	0	0	0	0	0
0	14	0	0	0	0	0	0	0	0	0
0	10	0	0	0	3	0	0	0	0	0
0	18	0	0	0	0	0	0	2	0	0
1	36	0	0	0	6	0	1	0	1	0
0	0	0	0	1	2	0	0	0	1	0
0	0	0	0	0	9	0	0	0	0	0
0	0	0	0	0	0	0	0	1	2	0
0	3	0	0	0	1	0	0	2	0	0
0	2	0	0	0	11	0	0	0	0	0
0	1	0	0	0	0	0	0	0	1	0
0	0	0	0	0	11	0	0	1	0	0
0	1	0	0	1	6	0	0	0	0	0
0	1	0	0	0	0	0	0	3	0	0
0	0	0	0	0	3	0	0	2	0	0
0	0	0	0	0	24	0	0	2	0	0
0	0	0	0	0	9	0	0	3	0	0
0	0	0	0	2	7	0	0	0	1	0
0	3	0	0	0	2	0	0	0	0	0
0	2	0	0	0	3	0	0	2	1	0
0	0	0	0	0	10	0	0	0	0	0
0	0	0	0	0	8	0	0	1	0	0

0	1	0	0	1	12	0	0	0	0	0
0	0	0	0	1	4	0	0	2	0	0
0	0	0	0	0	0	0	0	1	0	0
0	0	0	0	0	4	0	0	0	1	0
0	5	0	0	0	6	0	0	0	1	0
0	0	0	0	0	2	0	0	0	0	0
0	1	0	0	0	5	0	0	0	0	0
0	1	0	0	0	3	0	0	0	0	0
0	2	0	0	1	5	0	0	0	0	0
0	0	0	0	0	4	0	0	0	0	0
0	1	0	0	0	2	0	0	0	0	0
0	1	0	0	3	9	0	0	0	0	0
0	2	0	0	0	14	0	0	0	1	0
3	4	0	0	0	5	0	0	0	0	0
0	0	0	0	0	5	0	0	0	0	0
0	0	0	0	0	3	0	0	0	0	0
0	0	0	0	0	6	0	0	1	0	0
0	0	0	0	0	1	0	0	0	0	0
0	0	0	0	0	3	0	0	0	0	0
0	0	0	0	0	3	0	0	0	0	0
0	1	0	0	0	5	0	0	0	0	0
0	2	0	0	1	2	0	0	0	0	0
0	2	0	0	1	5	0	0	0	2	0
0	2	0	0	0	2	0	0	0	0	0
0	0	0	0	0	8	0	0	0	4	0
0	0	0	0	0	2	0	0	0	0	0
0	0	0	0	0	3	0	0	0	0	0
1	4	0	0	0	4	0	0	0	0	0
0	3	0	0	0	1	0	0	1	1	0

0	0	0	0	0	4	0	0	1	0	0
0	1	0	0	0	7	0	0	1	0	0
0	1	0	0	0	9	0	0	0	0	0
0	3	1	0	0	8	0	0	0	0	0
0	0	0	0	0	7	0	0	0	0	0
0	2	0	0	0	5	0	0	1	0	0
0	0	0	0	0	6	0	0	1	0	0
0	0	0	0	0	12	0	0	0	1	0
0	0	0	0	0	10	0	0	0	0	0
0	3	0	0	1	1	0	0	1	0	0
0	2	0	0	0	11	0	0	1	0	0
0	0	0	0	0	5	0	0	0	0	0
0	0	0	0	0	7	0	0	0	1	0
0	1	0	0	0	2	0	0	0	0	0
0	1	0	0	0	9	0	0	0	0	0
0	2	0	0	0	2	0	0	0	0	0
0	1	0	0	0	12	0	0	1	1	0
0	3	0	0	0	10	0	0	2	0	0
0	3	0	0	0	11	0	0	2	1	0
0	5	0	0	0	5	0	0	0	0	0
0	0	0	0	0	5	0	0	2	1	0
0	2	0	0	0	4	0	0	0	0	0
0	6	0	0	0	7	0	0	2	2	0
0	3	0	0	0	7	0	0	2	1	0
0	5	0	0	0	0	0	0	0	0	0
0	10	0	0	0	2	0	0	0	0	0
0	7	0	0	1	15	0	0	1	0	0
0	3	0	0	0	9	0	0	0	2	0
0	4	0	0	0	8	0	0	0	0	0

0	11	0	0	0	0	0	0	1	1	0
0	5	0	0	0	7	0	0	0	0	0
0	1	0	0	0	10	0	0	0	0	0
0	12	0	0	0	5	0	1	0	0	0
0	8	0	0	2	3	0	0	0	0	0
0	6	0	0	0	0	0	0	0	0	0
0	4	0	0	0	1	0	0	0	0	0
0	14	0	0	0	3	0	0	0	0	0
0	24	0	0	0	6	0	0	0	1	0
1	12	0	0	0	13	0	0	1	2	0
0	22	0	0	0	7	0	1	1	0	0
0	20	0	0	1	26	0	0	0	0	0
0	6	0	0	1	3	0	0	0	0	0
0	9	0	0	0	0	0	0	0	0	0
0	3	0	0	1	3	0	2	0	0	0
0	6	0	0	0	0	0	1	0	0	1
0	4	0	0	0	5	0	0	1	0	0
0	3	0	0	0	5	0	1	1	1	0
0	9	0	0	0	1	0	0	0	0	0
0	2	0	0	0	4	0	0	0	0	0
0	5	0	0	0	9	0	0	0	0	0
0	9	0	0	0	1	0	0	0	0	0
0	2	0	0	1	12	0	0	0	0	0
0	9	0	0	0	7	0	1	0	0	0
0	6	1	0	1	8	0	0	1	2	0
0	2	0	0	0	0	0	0	0	4	0
0	2	0	0	0	1	0	0	1	0	0
0	9	0	0	0	6	0	0	0	0	0
0	3	0	0	0	9	0	0	0	1	0

0	2	0	0	0	0	0	0	1	1	0
0	4	0	0	0	10	0	0	3	0	0
0	1	0	0	0	10	0	0	1	0	0
0	1	0	0	0	2	0	0	0	1	0
0	4	0	0	0	9	0	0	3	0	0
0	2	0	0	0	12	0	0	0	0	0
0	2	0	0	0	9	0	0	0	2	0
0	1	0	0	0	7	0	0	0	2	0
0	0	0	0	0	13	0	0	2	0	0
0	3	0	0	0	13	0	0	0	0	0
0	1	0	0	0	5	0	0	1	0	0
0	5	0	0	0	3	0	0	0	1	0
0	0	0	0	0	4	0	0	0	0	0
0	2	0	0	0	5	0	0	0	0	0
0	3	0	0	0	16	0	0	0	2	0
0	4	0	0	0	2	0	0	0	0	0
0	0	0	0	0	7	0	0	0	0	0
0	2	0	0	0	0	0	0	0	0	0
0	5	0	0	0	5	0	0	0	0	0
0	0	0	0	0	10	0	0	0	1	0
0	0	0	0	0	3	0	0	0	1	0
0	2	0	0	0	11	0	0	0	0	0
0	2	0	0	0	8	0	0	0	0	0
0	0	0	0	0	6	0	0	0	0	0
0	0	0	0	0	0	0	0	0	0	0
0	0	0	0	0	5	0	0	0	0	0
0	1	0	0	0	4	0	0	0	0	0
0	1	0	0	0	4	0	0	0	1	0
0	2	0	0	0	0	0	0	1	0	0

0	1	0	0	0	7	0	0	0	1	0
0	0	0	0	0	11	0	0	1	0	0
0	0	0	0	0	7	0	0	3	1	0
1	1	0	0	0	3	0	0	0	0	0
0	1	0	0	0	11	0	0	0	0	0
0	1	0	0	0	3	0	0	0	2	0
0	1	0	0	0	0	0	0	0	0	0
0	2	0	0	0	3	0	0	0	0	0
0	2	0	0	0	3	0	0	0	1	0
0	0	0	0	0	7	0	0	2	0	0
0	0	0	0	0	4	0	0	0	0	0
0	2	0	0	0	0	0	0	0	0	0
0	0	0	0	0	4	0	0	0	1	0
0	0	0	0	0	6	0	0	0	0	0
1	0	0	0	0	5	0	0	0	0	0
0	4	0	0	0	4	0	0	0	0	0
0	0	0	0	0	7	0	0	2	1	0
0	0	0	0	0	6	0	0	0	1	0
0	0	0	0	0	1	0	0	0	0	0
0	2	0	0	0	2	0	0	0	0	0
1	1	0	0	0	5	0	0	0	0	0
0	0	0	0	0	15	0	0	1	0	0
0	1	0	0	0	2	1	0	0	0	0
0	0	0	0	0	3	0	0	0	0	0
0	1	0	0	0	0	0	0	0	1	0
0	1	0	0	0	4	0	0	0	0	0
0	2	0	0	0	2	0	0	0	0	0
0	2	0	0	0	1	0	0	0	0	0
0	1	0	0	0	2	0	0	0	0	0

0	3	0	0	0	7	0	0	0	0	0
0	2	0	0	0	6	0	0	0	0	0
0	0	0	0	0	4	0	0	1	0	0
0	0	0	0	1	4	0	0	0	0	0
0	0	0	0	0	8	0	0	3	0	0
0	1	0	0	0	4	0	0	0	0	0
0	1	1	0	0	2	0	0	0	0	0
0	2	0	0	1	1	1	0	1	1	0
0	1	0	0	0	14	0	0	0	1	0
0	0	0	0	1	0	0	0	0	0	0
0	0	0	0	0	18	0	0	2	0	0
0	0	0	0	0	1	0	0	1	2	0
0	0	0	0	0	3	0	0	0	0	0
0	2	0	0	0	12	0	0	0	1	0
0	0	0	0	1	6	0	0	0	0	0
0	0	0	0	0	2	0	0	0	1	0
0	1	0	0	0	0	0	0	1	0	0
0	2	0	0	0	7	0	0	0	0	0
0	0	0	0	0	0	0	0	0	0	0
0	2	0	0	0	8	0	0	0	0	0
0	0	0	0	0	10	0	0	0	0	0
0	1	0	0	0	5	0	0	0	0	0
0	1	0	0	0	3	0	0	0	0	0
0	0	2	0	1	6	0	0	0	0	0
0	2	1	0	0	2	0	0	0	0	0
0	1	0	0	1	2	0	0	0	0	0
0	3	0	0	0	20	0	0	1	0	0
0	0	0	0	0	6	0	0	1	0	0
0	3	0	0	0	3	0	0	1	1	0

0	1	0	0	0	2	0	0	0	4	0
0	1	0	0	4	9	0	0	2	0	0
0	0	0	0	0	15	0	0	0	1	0
0	1	0	0	0	11	0	0	6	1	0
0	0	0	0	1	3	0	0	0	0	0
0	2	0	0	0	2	0	0	1	0	0
0	1	0	0	0	1	0	0	1	0	0
0	2	0	0	0	0	0	0	0	0	0
0	1	0	0	0	6	0	0	0	0	0
0	2	0	0	0	0	0	0	0	0	0
0	0	0	0	0	5	0	0	0	0	0
0	2	0	0	0	2	0	0	0	0	0
0	4	0	0	0	0	0	0	0	0	0
0	1	1	0	0	0	0	0	0	0	0
0	0	0	0	0	3	0	0	0	2	0
0	0	0	0	0	2	0	0	0	1	0
0	1	0	0	0	0	0	0	0	0	0
0	0	0	0	0	2	0	0	0	0	0
0	0	0	0	0	5	0	0	1	0	0
0	0	0	0	0	5	0	0	0	2	0
0	1	0	0	0	16	0	0	7	0	0
0	2	0	0	0	6	0	0	0	2	0
0	0	0	0	0	2	0	0	5	0	0
0	2	0	0	0	2	0	0	0	1	0
0	0	0	0	0	5	0	0	0	0	0
0	0	0	0	0	10	0	0	2	0	0
0	0	0	0	0	5	0	0	0	1	0
0	6	0	0	0	5	0	0	0	0	0
0	1	0	0	0	11	0	0	0	0	0

1	1	0	0	0	6	0	0	3	0	0
0	4	0	0	0	3	0	0	0	0	0
0	0	0	0	0	2	0	0	7	0	0
0	0	0	0	1	14	0	0	2	1	0
0	0	0	0	0	19	0	0	2	0	0
0	2	0	0	0	8	0	0	1	0	0
0	0	0	0	2	6	0	0	4	0	0
0	3	0	0	0	17	0	0	1	1	0
0	0	0	0	0	0	0	0	1	0	0
0	2	0	0	0	6	0	0	0	0	0
0	0	0	0	0	0	0	0	0	0	0
0	1	0	0	0	6	0	0	0	0	0
0	0	0	0	0	3	0	0	1	0	0
0	1	0	0	0	9	0	0	0	0	0
0	2	0	0	0	1	0	0	0	0	0
0	3	0	0	0	0	0	0	0	1	0
0	4	0	0	0	3	0	0	0	0	0
0	2	0	0	0	7	0	0	0	0	0
0	4	0	0	0	15	0	0	0	0	0
0	4	0	0	0	4	0	0	1	0	0
0	1	0	0	0	16	0	0	1	0	0
0	2	0	0	0	1	0	0	0	0	0
0	0	0	0	0	7	0	0	0	0	0
0	1	0	0	0	14	0	0	0	0	0
0	3	0	0	0	8	0	0	0	1	0
0	7	0	0	0	2	0	0	0	0	0
0	3	0	0	0	5	0	0	0	0	0
0	5	0	0	0	7	0	0	0	0	0
0	3	0	0	0	8	0	0	2	0	0

0	4	0	0	0	10	0	0	0	0	0
0	1	0	0	0	3	0	0	1	1	0
0	5	0	0	0	0	0	0	0	0	0
0	0	0	0	0	1	0	0	0	1	0
2	0	0	0	0	7	0	0	0	3	0
0	4	0	0	0	3	0	0	4	1	0
0	1	0	0	0	6	0	0	0	1	0
0	1	0	0	0	4	0	0	0	1	1
0	1	0	0	0	5	0	0	0	0	0
0	11	0	0	1	5	0	0	0	0	0
1	4	0	0	0	1	0	0	1	0	0
0	3	0	0	2	0	0	0	0	0	0
0	3	0	0	0	0	0	0	0	0	0
0	4	0	0	1	2	0	0	0	1	0
0	4	0	0	0	0	0	0	1	0	0
0	11	0	0	0	0	0	0	0	0	0
0	3	0	0	0	9	0	0	1	0	0
0	7	1	0	0	4	0	0	1	2	0
0	6	0	0	0	4	0	0	0	0	0
0	6	0	0	0	0	0	0	0	0	0
0	3	0	0	0	0	0	2	0	0	0
0	9	0	0	0	1	0	1	0	0	0
2	18	0	0	1	6	0	0	0	1	0
0	5	0	0	1	2	0	0	0	0	0
0	1	0	0	0	0	0	0	0	0	0
0	14	0	0	0	1	0	0	0	0	0
0	19	1	0	0	3	0	2	0	0	0
0	7	0	0	0	7	0	0	0	1	0
3	5	2	0	0	10	0	0	1	0	0

0	8	0	0	0	3	0	0	0	0	0
0	16	0	0	0	0	0	0	0	1	0
0	11	0	0	0	0	0	0	0	1	0
0	13	0	0	0	0	0	0	1	0	0
1	16	0	0	0	5	0	0	0	2	0
0	16	0	0	0	0	0	0	0	0	0
1	8	0	0	0	8	0	0	0	0	0
1	35	0	0	0	10	0	0	1	3	0
1	26	0	1	0	4	0	0	0	1	0
0	42	0	0	0	6	0	1	0	1	0
0	11	0	0	0	1	0	0	0	0	0
0	39	0	0	0	0	0	0	0	0	0
0	9	0	0	0	3	0	0	2	0	0
0	11	0	0	0	3	0	0	0	0	0
0	6	0	0	0	3	0	0	0	0	0
0	13	0	0	0	2	0	0	0	0	0
0	5	0	0	0	6	0	0	0	2	0
0	6	0	0	0	0	0	0	0	0	0
0	15	0	0	0	1	0	0	0	1	0
0	3	0	0	0	2	0	2	1	0	0
0	10	0	0	1	3	0	0	0	0	0
0	2	0	0	2	0	0	0	0	1	0
0	5	0	0	0	0	0	0	0	0	0
0	3	0	0	0	15	0	0	0	0	0
0	8	0	0	1	0	0	0	0	1	0
0	8	0	0	0	4	0	0	0	0	0
0	8	0	0	0	6	0	0	0	0	0
0	7	0	0	0	9	0	0	0	0	0
0	2	0	0	0	3	0	0	0	0	0

0	6	0	0	0	4	0	0	1	2	0
0	7	0	0	0	5	0	0	0	1	0
0	1	0	0	0	11	0	0	2	0	0
0	8	0	0	0	1	0	0	0	0	0
0	0	0	0	0	5	0	0	0	0	0
0	3	0	0	0	2	0	0	0	0	0
0	4	0	0	0	5	0	0	0	1	0
0	3	0	0	0	6	0	0	5	0	0
0	1	0	0	0	8	0	0	2	1	0
0	2	0	0	0	13	0	0	4	1	0
0	7	0	0	0	0	0	0	0	3	0
0	3	0	0	0	0	0	0	0	0	0
0	1	0	0	0	0	0	0	0	0	0
0	3	0	0	0	2	0	0	0	0	0
0	1	0	0	0	9	0	0	1	0	0
0	1	0	0	0	0	0	0	0	0	0
0	0	0	0	0	0	0	0	0	0	0
0	3	0	0	0	9	0	0	0	0	0
0	0	0	0	0	7	0	0	2	1	0
0	4	0	0	0	0	0	0	0	0	0
0	5	0	0	0	6	0	0	0	1	0
0	0	0	0	0	3	0	0	1	0	0
0	3	0	0	0	5	0	0	2	1	0
1	4	0	0	0	4	0	0	3	0	0
0	2	0	0	0	0	0	0	0	1	0
0	1	0	0	0	3	0	0	1	0	0
2	1	0	0	0	5	0	0	0	0	0
0	1	0	0	0	0	0	0	0	0	0
0	1	0	0	0	0	0	0	0	0	0

0	2	0	0	0	2	0	0	7	0	0
0	3	0	0	0	0	0	0	1	0	0
0	2	0	0	0	0	0	0	0	0	0
0	1	0	0	0	12	0	0	3	0	0
0	1	0	0	0	4	0	0	0	0	0
0	1	0	0	0	2	0	0	0	0	0
0	0	0	0	0	3	0	0	0	0	0
0	0	0	0	0	4	0	0	2	0	0
0	4	0	0	0	13	0	0	0	0	0
0	4	0	0	0	4	0	0	4	1	0
0	2	0	0	0	2	0	0	0	0	0
0	2	0	0	0	4	0	0	0	3	0
0	4	0	0	0	7	0	0	0	0	0
0	2	1	0	0	4	0	0	0	1	0
0	0	0	0	0	1	0	0	0	0	0
0	4	0	0	0	5	0	0	0	1	0
0	6	0	0	0	0	0	0	0	0	0
0	1	0	0	1	4	0	0	0	0	0
0	1	1	0	0	8	0	0	1	0	0
0	1	0	0	0	4	0	0	0	2	0
0	3	0	0	0	2	0	0	0	0	0
0	0	0	0	0	0	0	0	0	0	0
0	0	0	0	0	4	0	0	3	1	0
0	2	0	0	0	7	0	0	3	1	0
0	0	0	0	0	1	0	0	2	0	0
0	1	1	0	0	3	0	0	0	0	0
0	3	1	0	1	8	0	0	0	0	0
0	2	0	0	0	0	0	0	0	0	0
0	1	0	0	0	2	0	0	0	2	0

0	2	4	0	0	6	0	0	3	0	0
0	0	1	0	0	0	0	0	0	0	0
0	0	6	0	0	1	0	0	0	0	0
0	1	3	0	0	16	0	0	4	1	0
0	0	1	0	0	0	0	0	0	2	0
0	0	0	0	0	6	0	0	0	1	0
0	2	0	0	0	12	0	0	2	1	0
0	0	1	0	0	7	0	0	6	0	0
0	0	0	0	0	1	0	0	0	0	0
0	0	0	0	1	3	0	0	1	0	0
0	0	0	0	0	5	0	0	0	0	0
0	1	0	0	1	9	0	0	0	0	0
0	0	0	0	0	9	0	0	0	0	0
0	0	0	0	0	2	0	0	0	1	0

chaeto	chigla	cirrat	collem	comads	comgla	coroph	coscon	cragig	cycova	cyctho
0	0	0	1	0	0	0	0	0	0	0
0	0	0	1	0	5	0	0	0	0	0
0	0	0	1	0	3	0	0	0	0	0
0	0	0	1	0	1	0	0	0	0	0
0	0	0	0	0	0	0	0	0	0	0
0	0	0	0	0	1	0	0	0	0	0
0	0	0	1	0	0	0	0	0	0	0
0	0	0	0	0	0	0	0	0	0	0
0	0	1	0	0	1	0	0	0	0	0
0	0	0	1	0	0	0	0	0	0	0
0	0	0	0	0	1	0	0	0	2	0
0	0	0	0	0	0	0	0	0	0	0
0	0	0	0	0	0	0	0	0	0	0
0	0	0	0	0	0	0	0	0	0	0
0	0	0	3	0	0	0	0	0	0	0
0	0	0	0	0	0	0	0	0	0	0
0	0	0	0	0	0	0	0	0	0	0
0	0	0	0	0	0	0	0	0	0	0
0	0	0	0	0	0	0	0	0	0	0
0	0	0	0	0	0	0	0	0	0	0
0	0	0	0	0	0	0	0	0	0	0
0	0	0	1	0	0	0	0	0	0	0
0	0	0	0	0	0	0	0	0	0	0
0	0	0	0	0	0	0	0	0	0	0
0	0	0	0	0	0	0	0	0	0	0
0	0	0	0	0	0	0	0	0	0	0
0	0	0	0	0	0	0	0	0	0	0
0	0	0	1	0	0	0	0	0	0	0

0	0	0	0	0	0	0	0	0	0	0
0	0	0	0	0	0	0	0	0	0	0
0	0	1	0	0	0	0	0	0	0	0
0	0	0	7	0	0	0	0	0	0	0
0	0	0	1	0	0	0	0	0	0	0
0	0	0	4	0	0	0	0	0	0	0
0	0	0	1	0	0	0	0	0	0	0
0	0	0	0	0	0	0	0	0	0	0
0	0	1	0	0	1	0	0	0	0	0
0	0	0	0	0	0	0	0	0	0	0
0	0	0	0	0	1	0	0	0	0	0
0	0	0	0	0	0	0	0	0	0	0
0	0	0	0	0	0	0	0	0	0	0
0	0	0	0	0	0	0	0	0	0	0
0	0	0	1	0	0	0	0	0	0	0
0	0	0	1	0	0	0	0	0	0	0
0	0	0	0	0	1	0	0	0	0	0
0	0	0	0	0	0	0	0	0	0	0
0	0	0	0	0	0	0	0	0	0	0
0	0	0	0	0	0	0	0	0	0	0
0	0	0	4	0	0	0	0	0	0	0
0	0	0	0	0	0	0	0	0	0	0
0	0	0	1	0	0	0	0	0	1	0
0	0	0	2	0	0	0	0	0	0	0
0	0	0	0	0	0	0	0	0	0	0
0	0	0	1	0	0	0	0	0	0	0
0	0	0	1	0	0	0	0	0	0	0
0	0	0	0	0	0	0	0	0	0	0
0	0	0	0	0	0	0	0	0	0	0

0	0	0	1	0	0	0	0	0	0	0
0	0	0	1	0	1	0	0	0	0	0
0	0	0	1	0	0	0	0	0	0	0
0	0	0	0	0	0	0	0	0	0	0
0	0	0	0	0	0	0	0	0	0	1
0	0	0	1	0	0	0	0	0	0	0
0	0	0	5	0	0	0	0	0	0	0
0	0	0	0	0	1	0	0	0	0	0
0	0	0	0	0	0	0	0	0	0	0
0	0	0	0	0	0	0	0	0	0	0
0	0	0	2	0	0	0	0	0	0	0
0	0	0	0	0	0	0	0	0	1	0
0	0	0	0	0	0	0	0	0	0	0
0	0	0	1	0	0	0	0	0	0	0
0	0	0	0	0	0	0	0	0	0	0
0	0	0	0	0	0	0	0	0	0	0
0	0	2	0	0	0	0	0	0	0	0
0	0	0	0	0	0	0	0	0	0	0
0	0	0	0	0	0	0	0	0	0	0
0	0	0	1	0	0	0	0	0	0	0
0	0	0	0	0	0	0	0	0	0	0
0	0	0	1	0	0	0	0	0	0	0
0	0	0	0	0	0	0	0	0	0	0
0	0	0	0	0	0	0	0	0	0	0
0	0	0	0	0	0	0	0	0	0	0
0	0	0	1	0	0	0	0	0	0	0
0	0	0	2	0	0	0	0	0	1	0
0	0	0	0	0	0	0	0	0	0	0
0	0	0	0	0	0	0	0	0	0	0

0	0	0	0	0	0	0	0	0	1	0
0	0	0	0	0	0	0	0	0	0	0
0	0	0	0	0	1	0	0	0	0	0
0	0	0	0	0	0	0	0	0	0	0
0	0	0	0	0	0	0	0	0	0	0
0	0	0	0	0	0	0	0	0	0	0
0	0	0	0	0	0	0	0	0	0	0
0	0	0	0	0	1	0	0	0	0	0
0	0	0	0	0	1	0	0	0	0	0
0	0	0	1	0	0	0	0	0	0	0
0	0	0	0	0	0	0	0	0	0	0
0	0	0	0	0	0	0	0	0	0	0
0	0	0	0	0	0	0	0	0	0	0
0	0	0	1	0	0	0	0	0	0	0
0	0	0	0	0	0	0	0	0	0	0
0	0	0	3	0	0	0	0	0	0	0
0	0	0	0	0	0	0	0	0	0	0
0	0	0	0	0	0	0	0	0	0	0
0	0	0	0	0	1	0	0	0	0	0
0	0	0	0	0	0	0	0	0	0	0
0	0	0	0	0	0	0	0	0	0	0
0	0	0	1	0	0	0	0	0	1	0
0	0	0	0	0	1	0	0	0	2	0
0	0	0	0	0	0	0	0	0	0	0
0	0	0	0	0	0	0	0	0	2	0
0	0	0	0	0	1	3	0	0	0	0
0	0	0	1	0	1	0	0	0	0	0
0	0	0	0	0	0	0	0	0	2	0
0	0	0	0	0	1	0	0	0	0	1

0	0	0	1	0	0	0	0	0	1	0
0	0	0	1	0	3	0	0	0	0	0
0	0	0	0	0	1	0	0	0	0	0
0	0	0	1	0	0	0	0	0	0	0
0	0	0	3	0	0	0	0	0	0	0
0	0	0	0	0	0	0	0	0	0	0
0	0	0	1	0	0	0	0	0	0	0
0	0	0	0	0	1	0	0	0	0	0
0	0	0	2	0	0	0	0	0	0	0
0	0	0	1	0	0	0	0	0	0	0
0	0	0	0	0	0	0	0	0	0	0
0	0	0	0	0	0	0	0	0	0	0
0	0	0	0	0	0	0	0	0	0	0
0	0	0	0	0	0	0	0	0	0	0
0	0	0	0	0	10	0	0	0	0	0
0	0	0	0	0	0	0	0	0	0	0
0	0	0	0	0	0	0	0	0	0	0
0	0	0	0	0	0	0	0	0	0	0
0	0	0	0	0	0	0	0	0	0	0
0	0	0	1	0	1	0	0	0	0	0
0	0	0	1	0	0	0	0	0	0	0
0	0	0	1	0	0	0	0	0	0	0
0	0	0	0	0	1	0	0	0	0	0
0	0	0	0	0	1	0	0	0	0	0
0	0	0	3	0	1	0	0	0	0	0
0	0	0	0	0	0	0	0	0	0	0
0	0	0	0	0	1	0	0	0	0	0
0	0	0	0	0	0	0	0	0	0	0
0	0	0	0	0	3	0	0	0	0	1

0	0	0	0	0	0	0	0	0	0	0
0	0	0	0	0	2	0	0	0	0	0
0	0	0	0	0	0	0	0	0	0	0
0	0	0	1	0	1	0	0	0	0	0
0	0	0	0	0	0	0	0	0	0	0
0	0	0	2	0	0	0	0	0	0	0
0	0	0	5	0	0	0	0	0	0	0
0	0	0	0	0	1	0	0	0	0	0
0	0	0	0	0	0	0	0	0	0	0
0	0	0	0	0	0	0	0	0	0	0
0	0	0	0	0	0	0	0	0	0	0
0	0	0	2	0	0	0	0	0	0	0
0	0	0	2	0	0	0	0	0	0	0
0	0	0	0	0	0	0	0	0	0	0
0	0	0	0	0	2	0	0	0	0	0
0	0	0	0	0	0	0	0	0	0	0
0	0	0	0	0	0	0	0	0	0	0
0	0	0	0	0	0	0	0	0	0	0
0	1	0	0	0	0	0	0	0	0	0
0	2	0	0	0	0	0	0	0	0	0
0	1	0	0	0	0	0	1	0	0	0
0	0	0	0	0	0	0	0	0	1	0
0	1	0	0	0	0	0	0	3	0	0
0	0	0	0	0	1	0	0	0	0	0
0	0	0	2	0	0	0	0	0	0	0
0	0	0	1	0	0	0	0	0	0	0
0	1	0	4	0	0	0	0	0	0	0
0	0	0	2	0	0	0	0	0	0	0
0	0	0	3	0	0	0	0	0	0	0

0	0	0	0	0	0	0	0	0	0	0
0	0	0	1	0	0	0	0	0	0	0
0	0	0	2	0	0	0	0	0	0	0
0	0	0	1	0	0	0	0	0	0	0
0	0	0	0	0	0	0	0	0	0	0
0	0	0	1	0	0	0	0	0	0	0
0	0	0	1	0	0	0	0	0	0	0
0	0	0	1	0	0	0	0	0	0	0
0	0	0	1	0	0	0	0	0	0	0
0	0	0	0	0	0	0	0	0	0	0
0	0	0	0	0	0	0	0	0	0	0
0	0	0	0	0	0	0	0	0	0	0
0	0	0	0	0	0	0	0	0	0	0
0	0	0	0	0	0	0	0	0	0	0
0	0	0	1	0	0	0	0	0	0	0
0	0	0	1	0	0	0	0	0	0	0
0	0	0	0	0	1	0	0	0	0	0
0	0	0	0	0	1	0	0	0	0	0
0	0	0	0	0	0	0	0	0	0	0
0	0	0	0	0	0	0	0	0	0	0
0	0	0	2	0	0	0	0	0	0	0
0	0	0	0	0	0	0	0	0	0	0
0	0	0	1	0	0	0	0	0	0	0
0	0	0	2	0	0	0	0	0	0	0
0	0	0	3	0	0	0	0	0	0	0
0	0	0	0	0	1	0	0	0	0	0
0	0	0	0	0	0	0	0	0	0	0
0	0	0	2	0	0	0	0	0	0	0
0	0	0	3	0	0	0	0	0	0	0

0	0	0	1	0	0	0	0	0	0	0
0	0	0	1	0	0	6	0	0	0	0
0	0	0	2	0	0	0	0	0	0	0
0	0	0	6	0	0	0	0	0	0	0
0	0	0	1	0	0	0	0	0	0	0
0	0	0	2	0	1	0	0	0	0	0
0	0	0	7	0	0	0	0	0	0	0
0	0	0	2	0	0	0	0	0	0	0
0	0	0	3	0	1	0	0	0	0	0
0	0	0	4	0	0	0	0	0	0	0
0	0	0	5	0	1	0	0	0	0	0
0	0	0	2	0	0	0	0	0	0	0
0	0	0	0	0	1	0	0	0	0	0
0	0	0	6	0	0	0	0	0	0	0
0	0	0	3	0	0	0	0	0	0	0
0	0	0	3	0	0	0	0	0	0	0
0	0	0	7	0	0	0	0	0	0	0
0	0	0	8	0	1	0	0	0	0	0
0	0	0	1	0	1	0	0	0	0	0
0	0	0	0	0	0	0	0	0	0	0
0	0	0	2	0	0	0	0	0	0	0
0	0	0	1	0	0	0	0	0	0	0
0	0	0	10	0	0	0	0	0	0	0
0	0	0	7	0	0	0	0	0	0	0
0	0	0	6	0	1	0	0	0	0	0
0	0	0	5	0	0	0	0	0	0	0
0	0	0	0	0	0	0	0	0	0	0
0	0	0	2	0	0	0	0	0	0	0
0	0	0	1	0	0	0	0	0	0	0

0	0	0	0	0	0	0	0	0	0	0
0	0	0	7	0	0	0	0	0	0	0
0	0	0	0	0	0	0	0	0	0	0
0	0	0	1	0	0	0	0	0	0	0
0	0	0	0	0	1	0	0	0	0	0
0	0	0	0	0	0	0	0	0	0	0
0	0	0	0	0	0	0	0	0	0	0
0	0	0	4	0	0	0	0	0	0	0
0	0	0	1	0	1	0	0	0	0	0
0	0	0	1	0	0	0	0	0	0	0
0	0	0	0	0	0	0	0	0	2	0
0	0	0	2	0	0	0	0	0	0	0
0	0	0	3	0	0	0	0	0	0	0
0	0	0	1	0	0	0	0	0	0	0
0	0	0	0	0	0	0	0	0	0	0
0	0	0	1	0	0	0	0	0	0	0
0	0	0	0	0	1	0	0	0	0	0
0	0	0	0	0	1	0	0	0	0	0
0	0	0	0	0	1	0	0	0	0	0
0	0	0	4	0	1	0	0	0	0	0
0	0	0	0	0	1	0	0	0	0	0
0	0	0	1	0	0	0	0	0	1	0
0	0	0	3	0	0	0	0	0	0	0
0	0	0	2	0	0	0	0	0	1	0
0	0	0	0	0	0	0	0	0	0	0
0	0	0	0	0	0	0	0	0	1	0
0	0	0	3	0	0	0	0	0	0	0
0	0	0	2	0	0	0	0	0	0	0
0	0	0	0	0	0	0	0	0	1	0

0	0	0	0	0	0	0	0	0	0	0
0	0	0	1	0	0	0	0	0	0	0
0	0	0	1	0	3	0	0	0	0	0
0	0	0	5	0	0	0	0	0	1	0
0	0	0	0	0	0	0	0	0	0	0
0	0	0	1	0	0	0	0	0	0	0
0	0	0	0	0	0	0	0	0	0	0
0	0	0	2	1	1	0	0	0	0	1
0	0	0	3	0	1	0	0	0	1	1
0	0	0	6	0	0	0	0	0	0	1
0	0	0	0	0	0	0	0	0	1	0
0	0	0	2	0	0	0	0	0	0	0
0	0	0	0	0	0	0	0	0	0	0
0	0	0	3	0	0	0	0	0	0	0
0	0	0	4	0	0	0	0	0	0	0
0	0	0	0	0	0	0	0	0	0	1
0	0	0	1	0	0	0	0	0	0	0
0	0	0	1	0	1	0	0	0	0	0
0	0	0	0	0	0	0	0	0	0	0
0	0	0	0	0	0	0	0	0	0	0
0	0	0	1	0	0	0	0	0	0	0
0	0	0	1	0	2	0	0	0	0	0
0	0	0	0	0	0	0	0	0	0	0
0	0	0	2	0	0	0	0	0	0	0
0	0	0	3	0	0	0	0	0	0	0
0	0	0	1	0	1	0	0	0	0	0
0	0	0	2	0	0	0	0	0	0	0
0	0	0	1	0	0	0	0	0	0	0
0	0	0	4	0	0	0	0	0	0	0

0	0	0	3	0	0	0	0	0	0	0
0	0	0	7	0	0	0	0	0	0	0
0	0	0	1	0	0	0	0	0	1	0
0	0	0	2	0	0	0	0	0	0	1
0	0	0	4	0	0	2	0	0	0	1
0	0	0	3	0	0	0	0	0	0	0
0	0	0	4	0	0	0	0	0	0	0
0	0	0	6	0	0	0	0	0	0	0
0	0	0	0	0	0	0	0	0	0	0
0	0	0	0	0	0	0	0	0	0	0
0	0	0	3	0	0	0	0	0	0	0
0	0	0	6	0	1	0	0	0	3	3
0	0	0	7	0	0	0	0	0	0	0
0	0	0	3	0	0	1	0	0	0	2
0	0	0	2	0	0	0	0	0	2	13
0	0	0	5	0	0	0	0	0	0	1
0	0	0	1	0	0	0	0	0	0	0
0	0	0	4	1	0	0	0	0	0	0
0	0	0	2	0	0	0	0	0	0	1
0	0	0	2	0	0	0	0	0	0	0
0	0	0	1	0	0	0	0	0	0	0
0	0	0	4	0	0	0	0	0	0	0
0	0	0	1	0	0	0	0	0	0	0
0	0	0	3	0	0	0	0	0	1	1
0	0	0	1	0	0	0	0	0	0	0
0	0	0	0	0	0	0	0	0	0	0
0	0	0	2	0	0	0	0	0	0	0
0	0	1	3	0	0	0	0	0	1	0
0	0	0	4	0	0	0	0	0	0	0

0	0	0	3	0	0	0	0	0	0	0
0	0	0	3	0	0	0	0	0	0	0
0	0	0	1	0	0	0	0	0	1	0
0	0	0	3	0	0	0	0	0	0	0
0	0	0	0	0	0	0	4	0	0	0
0	0	0	0	1	0	0	0	0	0	0
0	0	0	5	0	0	0	0	0	0	0
0	0	0	4	0	0	0	0	0	0	0
0	0	0	7	0	0	0	0	0	0	0
0	0	0	0	0	0	0	0	0	0	0
0	0	0	0	0	0	0	0	0	0	0
0	0	1	3	0	0	0	0	0	0	0
0	0	0	0	0	0	0	0	0	0	0
0	0	0	1	0	0	0	0	0	0	0
0	0	0	1	0	0	0	0	0	0	0
0	0	0	4	0	0	0	0	0	1	0
0	0	0	0	0	1	0	0	0	0	0
0	0	0	0	0	1	0	0	0	0	0
0	0	0	0	0	0	0	0	0	0	0
0	0	0	5	0	0	0	0	0	0	0
0	0	0	2	0	1	0	0	0	0	0
0	0	0	0	0	0	0	0	0	0	0
0	0	0	1	0	0	0	0	0	0	0
0	0	0	3	0	2	0	0	0	0	0
0	0	0	0	0	0	0	0	0	0	0
0	0	0	1	0	0	0	0	0	0	0
0	0	0	2	0	0	0	0	0	0	0
0	0	0	1	0	0	0	0	0	0	0
0	0	0	5	0	0	0	0	0	0	0

0	0	0	1	0	0	0	0	0	0	0
0	0	0	5	0	0	0	0	0	0	0
0	0	0	2	0	1	0	0	0	0	0
0	0	0	2	0	1	0	0	0	0	0
0	0	0	1	0	0	0	0	0	0	0
0	0	0	0	0	1	0	0	0	0	0
0	0	0	4	0	0	0	0	0	0	0
0	0	0	0	0	1	0	0	0	0	0
0	0	0	1	0	0	0	0	0	0	0
0	0	0	1	0	0	0	0	0	0	0
0	0	0	1	0	1	0	0	0	0	0
0	0	0	1	0	0	0	0	0	0	0
0	0	0	1	0	0	0	0	0	0	0
0	0	0	1	0	0	0	0	0	0	0
0	0	0	1	0	0	0	0	0	0	0
0	0	0	0	0	0	0	0	0	0	0
0	0	0	0	0	0	0	0	0	0	0
0	0	0	0	0	0	0	0	0	0	0
0	0	0	2	0	0	0	0	0	0	0
0	0	0	7	0	0	0	0	0	0	0
0	0	0	0	0	1	0	0	0	0	0
0	0	0	3	0	0	0	0	0	0	0
0	0	0	4	0	0	0	0	0	0	0
0	0	0	2	0	0	0	0	0	0	0
0	0	0	2	0	0	0	0	0	0	0
0	0	0	2	0	0	0	0	0	0	0
0	0	0	2	0	1	0	0	0	0	0
0	0	0	2	0	0	0	0	0	0	0
0	0	0	3	0	0	0	0	0	0	0

0	0	0	1	0	0	0	0	0	0	0
1	0	0	8	0	0	0	0	0	0	0
0	0	0	0	0	0	0	0	0	0	0
0	0	0	1	0	0	0	0	0	0	0
0	0	0	5	0	0	0	0	0	0	0
0	0	0	2	0	0	0	0	0	0	0
0	0	0	7	0	0	0	0	0	0	0
0	0	0	4	0	0	0	0	0	0	0
0	0	0	1	0	0	0	0	0	0	0
0	0	0	2	0	0	0	0	0	0	0
0	0	0	1	0	0	0	0	0	0	0
0	0	0	3	0	0	0	0	0	0	0
0	0	0	1	0	0	0	0	0	0	0
0	0	0	3	0	0	0	0	0	0	0
0	0	0	0	0	0	0	0	0	0	0
0	0	0	2	0	0	0	0	0	0	0
0	0	0	5	0	0	0	0	0	0	0
0	0	0	6	0	0	0	0	0	0	0
0	0	0	0	0	0	8	0	0	0	0
0	0	0	2	0	0	0	0	0	0	0
0	0	0	9	0	0	0	0	0	0	0
0	0	0	0	0	0	0	0	0	0	0
0	0	0	2	0	0	0	0	0	0	0
chigla	cirola	cirrat	collem	comads	comgla	coscon	cycova	cyctho	dexami	dilsub
0	0	0	0	0	0	0	0	0	0	0
0	0	0	1	0	0	0	0	0	0	0
0	0	0	0	0	0	0	0	0	0	0
0	0	0	0	0	0	0	0	0	0	0

0	0	0	0	0	0	0	0	0	0	0
0	0	0	0	0	0	0	0	0	0	0
0	0	0	1	0	0	0	0	0	0	0
0	0	0	1	0	0	0	0	0	0	0
0	0	0	1	0	0	0	0	0	0	0
0	0	0	2	0	0	0	0	0	0	0
0	0	0	0	0	0	9	0	1	1	0
0	0	0	0	0	0	8	0	1	2	0
0	0	0	0	0	0	8	0	0	1	0
0	0	0	0	0	0	7	0	0	0	0
0	0	0	0	0	0	4	0	0	1	0
0	0	0	0	0	0	8	0	0	0	0
0	0	0	0	0	1	19	0	0	0	0
0	0	0	0	0	0	10	0	0	1	0
0	0	0	0	0	0	5	0	0	7	0
0	0	0	0	0	0	7	0	0	1	0
0	0	0	1	0	0	0	0	0	0	0
0	0	0	1	0	0	0	0	0	0	0
0	0	0	0	0	1	0	0	0	0	0
0	0	0	0	0	0	0	0	0	0	0
0	0	0	0	0	0	0	0	0	0	0
0	0	0	4	0	0	0	0	0	0	0
0	0	0	1	0	0	0	0	0	0	0
0	0	0	0	0	0	0	0	0	0	0
0	0	0	0	0	0	0	0	0	0	0
0	0	0	1	0	0	0	0	0	0	0
0	0	0	1	0	0	0	0	0	0	0
0	0	0	1	0	0	0	0	0	0	0
0	0	0	0	0	0	0	0	0	0	0

0	0	0	0	0	0	0	0	0	0	0
0	0	0	0	0	0	0	0	0	0	0
0	0	0	1	0	1	0	0	0	0	0
0	0	0	0	0	0	0	0	0	0	0
0	0	0	1	0	0	0	0	0	0	0
0	0	0	0	0	0	0	0	0	0	2
1	0	0	1	0	2	0	0	0	0	0
0	0	0	1	0	0	0	0	0	0	1
0	0	0	0	0	0	0	1	0	0	0
0	0	0	1	0	1	0	0	1	0	1
0	0	0	0	0	0	0	0	0	0	0
0	0	0	1	0	0	0	0	0	0	1
0	0	0	0	0	0	0	0	0	0	0
0	0	0	0	0	0	0	0	0	0	1
0	0	0	0	0	0	0	0	0	0	1
0	0	0	0	0	0	0	0	0	0	1
0	0	0	0	0	2	0	0	0	0	0
0	0	0	0	0	0	0	0	0	0	0
0	0	1	0	0	0	0	0	0	0	1
0	0	0	0	0	0	0	0	0	0	0
0	0	0	0	0	0	0	0	0	0	0
0	0	0	0	0	0	0	0	0	0	2
0	0	0	0	0	0	0	0	0	0	2
0	0	0	0	0	0	0	0	0	0	2
0	0	0	0	0	0	0	0	0	0	1
0	0	0	0	0	0	0	0	0	0	0
0	0	0	0	0	0	0	0	1	0	1
0	0	0	0	0	0	0	0	0	0	0
0	0	0	1	0	0	0	0	3	0	1

0	0	0	0	0	0	0	0	0	0	0
0	0	0	0	0	2	0	0	0	0	0
0	0	0	0	0	0	0	0	0	0	0
1	0	0	0	0	0	0	0	0	0	0
0	0	0	0	0	0	2	0	0	0	0
0	0	0	0	0	0	0	0	0	0	0
0	0	0	0	0	0	0	0	0	0	0
0	0	0	0	0	0	1	0	0	0	0
0	0	0	0	0	0	1	0	0	0	0
0	0	0	0	0	0	2	0	0	0	0
0	0	0	0	0	0	0	0	0	0	0
0	0	0	0	0	1	0	0	0	0	2
0	0	0	0	0	0	0	0	0	0	0
0	0	0	0	0	0	0	1	0	0	0
0	0	0	0	0	0	0	1	0	0	0
0	0	0	0	0	0	0	0	0	0	0
0	0	0	0	0	0	0	0	0	0	0
0	0	0	0	0	0	3	1	0	0	1
0	0	0	0	0	0	0	0	1	0	1
0	0	0	0	0	0	4	0	0	0	0
0	0	0	0	0	0	0	0	1	0	1
0	0	0	0	0	0	2	1	0	0	1
0	0	0	0	0	0	3	1	0	0	2
0	0	0	1	0	0	5	0	0	0	0
0	0	0	0	0	0	4	0	0	0	0
0	0	0	0	0	0	6	0	0	0	0
0	0	0	0	0	0	3	0	0	0	0
0	0	0	0	0	0	11	0	2	0	0
0	0	0	0	0	0	5	0	0	0	0

0	0	0	0	0	0	2	0	0	0	0
0	0	0	0	0	0	6	0	0	0	0
0	0	0	0	0	0	4	0	0	0	0
0	0	0	0	0	2	1	0	0	0	0
0	0	0	0	0	0	12	0	0	0	0
0	0	0	0	0	0	11	0	0	0	0
0	0	0	0	0	0	8	0	0	0	0
0	0	0	1	0	0	7	0	0	0	0
0	0	0	0	0	0	6	0	0	5	0
0	0	0	0	0	0	4	0	0	1	0
0	0	0	0	0	0	4	0	0	0	0
0	0	0	0	0	0	9	0	0	8	0
0	0	0	0	0	0	10	0	1	1	0
0	0	0	0	0	0	9	0	0	0	0
0	0	0	0	0	0	5	0	0	2	0
0	0	0	0	0	0	6	0	1	2	0
0	0	0	0	0	0	12	0	0	2	0
0	0	0	0	0	0	8	0	0	5	0
0	0	0	0	0	0	2	0	0	3	0
0	0	0	0	0	0	7	0	0	14	0
0	0	0	0	0	0	6	0	0	0	0
0	0	0	0	0	0	11	0	0	1	0
0	0	0	0	0	0	8	0	0	1	0
0	0	0	0	0	1	8	0	0	0	0
0	0	0	0	0	0	13	0	0	3	0
0	0	0	0	0	0	9	0	2	0	0
0	0	0	0	0	0	3	0	0	0	0
0	0	0	0	0	0	4	0	0	0	0
0	0	0	0	0	0	5	0	0	1	0

0	0	0	0	0	1	9	0	0	0	0
0	0	0	0	0	0	5	0	4	1	0
0	0	0	0	0	0	5	0	0	0	0
0	0	0	0	0	0	7	0	0	1	0
0	0	0	0	0	0	6	0	2	0	0
0	0	0	0	1	0	8	0	0	0	0
0	0	0	0	0	1	13	0	0	0	0
0	0	0	0	0	1	4	0	0	0	0
0	0	0	0	0	0	5	0	0	0	0
0	0	0	0	0	0	3	0	0	0	0
0	0	0	0	0	0	8	0	4	0	0
0	0	0	0	0	0	2	0	0	0	1
0	0	0	0	0	1	6	0	0	0	0
0	0	0	0	0	0	7	0	1	0	0
0	0	0	0	0	0	2	0	2	0	0
0	0	0	0	0	0	2	1	0	0	0
0	0	0	0	0	0	4	0	0	0	0
0	0	0	0	0	0	5	0	1	0	0
0	0	0	0	0	0	4	0	0	0	0
0	0	0	0	0	0	2	0	2	0	1
0	0	0	0	0	0	3	0	1	0	0
0	0	0	0	0	0	1	0	2	0	0
0	0	0	0	0	0	5	0	1	0	0
0	0	0	0	1	0	0	0	3	0	0
0	0	0	0	1	1	4	0	5	0	1
0	0	0	0	0	0	0	0	2	0	0
0	0	0	0	0	0	1	0	0	0	0
0	0	0	0	0	0	0	0	0	0	0
0	0	0	0	0	2	2	0	2	0	2

0	0	0	1	0	0	0	0	0	0	0
0	0	0	0	0	0	0	0	0	0	1
0	0	0	0	0	0	0	0	0	0	0
0	0	0	0	0	0	0	0	1	0	0
0	0	0	0	0	0	1	0	1	0	1
0	0	0	0	0	2	0	0	0	0	0
0	0	0	0	0	0	2	0	3	0	0
0	0	0	0	0	2	1	0	0	0	0
0	0	0	0	0	0	0	0	2	0	1
0	0	0	0	0	0	0	0	0	0	0
0	0	0	0	0	0	0	0	0	0	0
0	0	0	0	0	0	0	0	1	0	0
0	0	0	0	0	2	0	0	0	0	2
0	0	0	0	0	0	0	0	0	0	0
0	0	0	0	0	0	0	0	1	0	1
0	0	0	0	0	0	0	1	0	0	0
0	0	0	0	0	0	0	0	1	0	0
0	0	0	0	0	0	0	0	1	0	3
0	0	0	0	0	0	0	0	0	0	1
0	0	0	0	0	1	0	0	1	0	2
0	0	0	0	0	0	0	0	0	0	0
0	0	0	0	0	0	0	0	0	0	0
0	0	0	0	0	0	0	0	0	0	0
0	0	0	0	0	0	0	0	0	0	0
0	0	2	0	0	0	0	0	0	0	0
0	0	0	0	0	0	0	0	0	0	0
0	0	0	0	0	0	0	0	0	0	1
1	0	0	0	0	0	0	0	0	0	2
0	0	0	0	0	0	0	0	0	0	0

0	0	0	0	0	0	0	0	0	0	0
0	0	1	0	0	0	0	0	0	0	0
0	0	0	0	0	0	0	0	0	0	0
0	0	0	0	0	0	0	0	0	0	0
1	0	0	0	0	0	0	0	0	0	0
0	0	0	0	0	0	0	0	0	0	0
0	0	0	0	0	0	0	0	0	0	0
1	0	0	0	0	0	0	0	0	0	3
0	0	0	0	0	0	0	0	0	0	0
0	0	0	0	0	1	0	0	0	0	0
2	0	0	0	0	1	0	0	0	0	0
0	0	0	0	0	2	0	0	0	0	0
0	0	0	0	0	0	0	0	1	0	0
0	0	0	0	0	0	0	0	0	0	0
0	0	0	0	0	0	0	0	0	0	0
0	0	0	0	0	0	0	0	0	0	0
0	0	0	0	0	0	0	0	1	0	2
0	0	0	0	0	0	0	0	0	0	0
0	0	0	0	0	0	0	0	0	0	0
0	0	0	0	0	0	0	0	0	0	0
0	0	0	0	0	0	0	0	0	0	0
0	0	0	0	0	0	0	0	0	0	0
0	0	0	0	0	0	0	0	0	0	0
0	0	0	0	0	1	0	0	0	0	0
0	0	0	2	0	0	0	0	0	0	0
0	0	0	0	0	0	0	0	0	0	1
0	0	0	0	0	0	0	0	0	0	0
0	0	0	0	0	0	0	0	0	0	0
0	0	0	0	0	0	0	0	1	0	0

0	0	0	0	0	0	0	0	0	0	0
0	0	0	0	0	0	0	0	0	0	0
0	0	0	0	0	0	0	0	1	0	2
0	0	0	0	0	0	0	0	0	0	0
0	0	0	0	0	0	0	0	0	0	0
0	0	0	0	0	0	0	0	0	0	0
0	0	0	0	0	0	0	0	0	0	0
0	0	0	0	0	0	0	0	0	0	0
0	0	0	0	0	0	0	0	0	0	1
0	0	0	0	0	0	0	0	0	0	0
0	0	0	0	0	0	0	0	0	0	0
0	0	0	0	0	0	0	0	1	0	0
0	0	0	0	0	0	0	0	0	0	0
0	0	0	0	0	2	0	0	1	0	0
0	0	0	0	0	0	0	0	0	0	1
0	0	0	0	0	0	0	2	2	0	0
0	0	0	0	0	0	0	0	0	0	1
0	0	0	0	0	0	0	0	0	0	1
0	0	0	0	0	1	0	0	0	0	1
0	0	0	0	0	1	0	0	0	0	0
0	0	0	0	0	0	0	0	0	0	0
0	0	0	0	0	0	0	0	0	0	0
0	0	0	0	0	0	0	0	0	0	0
0	0	0	0	0	0	0	0	2	0	0
0	0	0	0	0	0	0	0	1	0	0
0	0	0	0	0	2	0	0	1	0	1
0	0	0	0	0	0	0	0	2	0	2
0	0	0	0	0	0	0	0	0	0	0
0	0	0	0	0	0	0	0	2	0	1

0	0	0	0	0	0	0	0	0	0	2
0	0	0	0	0	0	0	0	0	0	0
0	0	0	0	0	0	0	0	0	0	1
0	0	0	0	0	0	0	0	0	0	0
0	0	0	0	0	0	0	0	0	0	0
0	0	0	0	0	0	0	0	0	0	2
0	0	0	0	0	0	0	0	0	0	0
0	0	0	0	0	0	0	0	0	0	0
0	0	0	0	0	0	0	0	1	0	0
0	0	0	0	0	0	0	0	2	0	0
0	0	0	0	0	2	0	0	1	0	0
0	0	0	0	0	0	0	0	0	0	0
0	0	0	0	0	0	0	0	2	0	1
0	0	0	0	0	0	0	0	0	0	0
0	0	0	0	0	2	0	0	4	0	0
0	0	0	0	0	1	3	0	0	0	0
0	0	0	0	0	0	0	0	1	0	1
0	0	0	0	0	0	0	0	0	0	1
0	0	0	0	0	0	0	0	0	0	1
0	0	0	0	0	0	0	0	0	0	1
0	0	0	0	0	0	0	0	1	0	1
0	0	0	0	0	0	0	0	2	0	1
0	0	0	0	0	0	1	0	0	0	0
0	0	0	0	0	0	0	0	2	0	0
0	0	0	0	0	2	1	0	0	0	0
0	0	0	0	0	0	11	0	0	0	0
0	0	0	0	0	0	3	0	0	0	0
0	0	0	0	0	0	5	0	1	0	0
0	0	0	0	0	0	1	0	2	0	0

0	0	0	0	0	0	4	0	0	0	0
0	0	0	0	0	1	1	0	1	0	0
0	0	0	0	0	0	2	0	0	0	0
0	0	0	0	0	0	1	0	8	0	0
0	0	0	0	0	0	5	0	0	0	0
0	0	0	0	0	0	12	0	1	0	0
0	0	0	0	0	0	10	0	2	0	0
0	0	0	0	0	0	2	0	0	0	0
0	0	0	0	0	0	4	0	0	0	0
0	0	0	0	0	0	15	0	0	0	0
0	0	0	0	0	0	6	0	0	0	0
0	0	0	0	0	0	6	0	1	0	0
0	0	0	0	0	0	5	0	1	0	0
0	0	0	0	0	0	5	0	0	0	0
0	0	0	0	0	0	4	0	0	0	0
0	0	0	0	0	0	5	0	0	0	0
0	0	0	0	0	0	1	1	0	0	0
0	0	0	0	0	0	1	0	0	4	0
0	0	0	0	0	0	2	0	0	0	0
0	0	0	0	0	0	4	0	1	1	0
0	0	0	0	0	0	3	0	0	0	0
0	0	0	0	0	0	1	0	0	0	0
0	0	0	0	0	0	8	0	0	0	0
0	0	0	0	0	0	6	0	0	0	0
0	0	0	0	0	0	5	0	0	0	0
0	0	0	0	0	0	7	0	0	0	0
0	1	0	0	0	0	4	0	0	0	0
0	0	0	0	0	0	8	0	5	0	0
0	0	0	0	0	0	3	0	0	1	0

0	0	0	0	0	0	5	0	1	1	0
0	0	0	0	0	0	8	0	0	2	0
0	0	0	0	0	0	5	0	0	0	0
0	0	0	0	0	0	3	0	0	0	0
0	0	0	0	0	0	6	0	0	0	0
0	0	0	0	0	0	3	0	1	2	0
0	0	0	0	0	0	1	0	0	0	0
0	0	0	0	0	1	3	0	0	0	0
0	0	0	0	0	2	0	0	0	0	0
0	0	0	0	0	0	4	0	0	0	0
0	0	0	0	0	0	2	0	0	0	0
0	0	0	0	0	0	3	0	0	0	0
0	0	0	0	0	0	3	0	0	0	0
0	0	0	0	0	0	1	0	0	0	0
0	0	0	0	0	0	0	0	1	0	0
0	0	0	0	0	0	5	0	2	0	0
0	0	0	0	0	0	1	0	0	1	0
0	0	0	0	0	0	2	0	0	0	0
0	0	0	0	0	0	3	0	0	0	0
0	0	0	0	0	0	2	0	0	0	0
0	0	0	0	0	0	1	0	1	0	0
0	0	0	0	0	0	0	0	0	0	0
0	0	0	0	0	0	0	0	1	0	0
0	0	0	0	0	0	4	0	0	0	0
0	0	0	0	0	0	2	0	0	0	0
0	0	0	0	0	0	1	0	0	0	0
0	0	0	0	0	0	1	0	1	0	0
0	0	0	0	0	0	0	0	0	0	0
0	0	0	0	0	0	2	0	0	1	0

0	0	0	0	0	0	0	0	0	0	0
0	0	0	0	0	0	1	0	2	0	0
0	0	0	0	0	0	0	0	0	0	0
0	0	0	0	0	0	2	0	0	0	0
0	0	0	0	0	0	0	0	0	0	0
0	0	0	0	0	0	0	1	1	0	0
0	0	0	0	0	0	0	0	0	0	0
0	0	0	0	0	0	1	0	0	0	0
0	0	0	0	0	0	1	0	0	0	0
0	0	0	0	0	0	0	0	1	0	1
0	0	0	0	0	1	1	0	0	0	0
0	0	0	0	0	0	1	0	1	0	0
0	0	0	0	0	0	0	0	1	0	0
0	0	0	0	0	0	0	0	0	0	0
0	0	0	0	0	1	2	0	1	0	0
0	0	0	0	0	0	0	1	0	0	2
0	0	0	0	0	0	0	0	0	0	0
0	0	0	0	0	0	1	0	0	0	0
0	0	0	0	0	1	0	0	0	0	0
0	0	0	0	0	0	0	0	0	0	0
0	0	0	0	1	1	1	0	0	0	0
0	0	0	0	0	0	0	1	0	0	1
0	0	0	0	0	2	0	0	0	0	1
0	0	0	0	0	0	0	0	1	0	1
0	0	0	0	0	0	0	0	5	0	0
0	0	0	0	0	0	0	0	0	0	0
0	0	0	0	0	0	0	0	3	0	0
0	0	0	0	0	0	0	0	0	0	1
0	0	0	0	0	1	0	0	3	0	1

0	0	0	0	0	0	0	0	3	0	0
0	0	0	0	0	0	0	0	0	0	3
0	0	0	0	0	0	0	0	0	0	1
0	0	0	0	0	1	0	0	2	0	0
0	0	0	1	0	1	0	0	0	0	1
0	0	0	0	0	0	0	0	2	0	2
0	0	0	0	0	0	0	0	0	0	0
0	0	0	0	0	0	0	0	1	0	1
0	0	0	0	0	0	0	0	3	0	0
0	0	0	0	0	0	0	0	0	0	0
0	0	0	0	0	0	0	0	1	0	0
0	0	0	1	0	0	0	0	1	0	0
0	0	0	0	0	2	0	0	1	0	0
0	0	0	0	0	1	0	0	1	0	0
0	0	0	0	0	0	0	0	1	0	2
0	0	0	0	0	0	0	0	0	0	0
0	0	0	0	0	0	0	0	1	0	3
0	0	0	0	0	0	0	0	0	0	1
0	0	0	0	0	7	0	0	0	0	0
0	0	0	0	0	0	0	0	1	0	0
0	0	0	0	0	0	0	0	0	0	1
0	0	0	0	0	0	0	0	0	0	1
0	0	0	0	0	0	0	0	0	0	0
0	0	0	0	0	0	0	0	2	0	0
0	0	0	0	0	0	0	0	0	0	0
0	0	0	0	0	0	0	0	1	0	0
0	0	0	2	0	0	0	0	0	0	0
0	0	0	0	0	1	0	0	0	0	0
0	0	0	0	0	0	0	0	0	0	0

0	0	0	0	0	0	0	0	0	0	1
0	0	0	0	0	0	0	0	0	0	0
0	0	0	0	0	0	0	0	0	0	0
0	0	0	0	0	0	0	0	0	0	0
0	0	0	0	0	0	0	0	0	0	0
0	0	0	0	0	1	0	0	0	0	0
0	0	0	0	0	0	0	0	0	0	0
0	0	0	0	0	0	0	0	0	0	0
0	0	0	0	0	0	0	0	0	0	0
0	0	0	0	0	0	5	0	0	0	0
0	0	0	0	0	0	0	0	0	0	0
0	0	0	0	0	0	0	0	0	0	0
0	0	0	0	0	0	0	0	1	0	0
0	0	0	0	0	0	0	0	0	0	0
0	0	0	0	0	0	0	0	0	0	0
0	0	0	0	0	1	0	1	0	0	1
0	0	0	0	0	0	0	0	0	0	0
0	0	0	0	0	0	0	0	0	0	0
0	0	0	0	0	1	0	0	0	0	0

coroph	dilsub	dorvil	eatoni	exowai	flatwo	halcoo	halwhi	hamzel	hemcre	hemedw
0	0	0	0	0	0	0	0	0	0	0
0	0	0	0	0	0	0	1	0	0	0
0	0	0	0	0	0	0	0	0	0	0
0	0	0	0	0	0	0	0	0	0	0
0	0	0	0	0	0	0	2	0	0	0
1	0	0	0	0	0	0	2	0	0	0
0	0	0	0	0	0	0	1	0	0	0
0	0	0	0	0	0	0	0	0	0	0

0	0	0	0	0	0	0	1	0	0	0
0	0	0	0	0	0	0	0	0	0	0
0	0	0	0	0	0	0	1	0	0	0
0	0	0	0	0	0	0	1	0	0	0
0	0	0	0	0	0	0	0	0	0	0
0	0	0	0	0	0	0	0	0	0	0
0	2	0	0	0	0	0	0	0	0	0
0	0	0	0	0	0	4	1	0	0	0
0	0	0	0	0	0	1	0	0	0	0
0	0	0	0	0	0	0	1	0	0	0
0	0	0	0	0	0	0	0	0	0	0
0	0	0	0	0	0	0	2	0	0	0
0	0	0	0	0	0	0	0	0	0	0
0	0	0	0	0	0	0	0	0	0	0
0	0	0	0	0	0	0	0	0	0	0
0	0	0	0	0	0	0	1	0	0	0
0	0	0	0	0	0	0	0	0	0	0
0	0	0	0	0	0	0	1	0	0	0
0	0	0	0	0	0	0	1	0	0	0
0	0	0	0	0	0	0	1	0	0	0
0	0	0	0	0	0	0	1	0	0	0
0	0	0	0	0	0	0	1	0	0	0
0	0	0	0	0	0	0	1	0	0	0
0	0	0	0	0	0	0	0	1	0	0
0	0	0	0	0	0	0	2	1	0	0
0	0	0	0	0	0	0	0	0	0	0
0	0	0	0	0	0	0	1	0	0	0
0	0	0	0	0	0	0	0	0	0	0
0	0	0	0	0	0	0	1	0	0	0

0	0	0	0	0	0	0	0	0	0	0
0	0	0	0	0	0	0	1	0	0	0
0	0	0	0	0	0	0	2	0	0	0
0	1	0	0	0	0	0	1	0	0	0
0	0	0	0	0	0	0	0	0	0	0
0	0	0	0	0	0	0	0	0	0	0
0	0	0	0	0	0	0	1	0	0	0
0	0	0	0	0	0	0	0	0	0	0
0	0	0	0	0	0	0	0	0	0	0
0	0	0	0	0	0	0	1	0	0	0
0	1	0	0	0	0	0	1	0	0	0
0	0	0	0	0	0	0	0	0	0	0
0	0	0	0	0	0	0	0	0	0	0
0	0	0	0	0	0	0	0	0	0	0
0	0	0	0	0	0	0	0	0	0	0
0	0	0	0	0	0	0	1	0	0	0
0	0	0	0	0	0	0	0	0	0	1
0	0	0	0	0	0	0	0	0	0	0
0	0	0	0	0	0	0	0	0	0	0
0	0	0	0	0	0	0	0	0	0	0
0	0	0	0	0	0	0	0	0	0	0
0	0	0	0	0	0	0	0	0	0	0
0	1	0	0	0	0	0	1	0	0	0
0	1	0	0	0	0	0	1	0	0	0
0	0	0	0	0	0	0	2	0	0	0
0	0	0	0	0	0	0	1	0	0	0
0	0	0	0	0	0	0	1	0	0	0
0	2	0	0	1	0	0	0	0	0	0
0	2	0	0	0	0	0	1	0	0	0

0	1	0	0	0	0	0	0	0	0	0
0	0	0	0	0	0	0	0	0	0	0
0	0	0	0	0	0	0	0	0	0	0
0	0	0	0	0	0	0	0	0	0	0
0	0	0	0	0	0	0	0	0	0	0
0	0	0	0	0	0	0	3	0	0	0
0	0	0	0	0	0	1	0	0	0	0
0	0	0	0	0	0	0	0	0	0	0
0	0	0	0	0	0	0	0	0	0	0
0	0	0	0	0	0	0	0	0	0	0
0	1	0	0	0	0	1	2	0	0	0
0	0	0	0	0	0	0	1	0	0	0
0	0	0	0	0	0	0	1	0	0	0
0	0	0	0	0	0	0	1	0	0	0
0	0	0	0	0	0	0	0	0	0	0
0	0	0	0	0	0	0	1	0	0	0
0	0	0	0	0	0	0	0	0	0	0
0	0	0	0	0	0	0	0	0	0	0
0	1	0	0	0	0	0	0	0	0	0
0	0	0	0	0	0	0	0	0	0	0
0	0	0	0	0	0	0	1	0	0	0
0	0	0	0	0	0	0	0	0	0	0
0	0	0	0	0	0	0	1	1	0	0
0	0	0	0	0	0	0	0	0	0	0
0	0	0	0	0	0	0	0	0	0	0
0	0	0	0	0	0	0	0	0	0	0
0	1	0	0	0	0	0	0	0	0	0
0	1	0	0	0	0	0	2	1	0	0
0	0	0	0	0	0	0	0	0	0	0

0	1	0	0	0	1	1	1	0	0	0
0	0	0	0	0	0	1	1	1	0	0
0	0	0	0	0	0	0	0	1	0	0
0	0	0	0	0	0	0	0	0	0	0
0	1	0	0	0	0	0	1	0	0	0
0	0	0	0	0	0	0	0	0	0	0
0	0	0	0	0	0	0	0	0	0	0
0	0	0	0	0	0	0	0	0	0	0
0	0	0	0	0	0	0	3	0	0	0
0	0	0	0	0	0	0	0	0	0	0
0	0	0	0	0	0	0	0	0	0	0
0	0	0	0	0	0	0	0	0	0	0
0	4	1	0	0	0	0	2	0	0	0
0	1	0	0	0	0	0	2	0	0	0
0	2	0	0	0	0	0	0	0	0	0
0	0	0	0	0	0	0	1	0	0	0
0	0	0	0	0	0	0	0	0	0	0
0	0	0	0	0	0	0	0	0	0	0
0	1	0	0	0	0	2	0	0	0	0
0	3	0	0	0	0	0	0	0	0	0
0	0	0	0	0	0	0	0	1	0	0
0	1	0	0	0	0	0	1	0	0	0
0	0	0	0	0	0	0	1	0	0	0
0	2	0	0	0	0	0	1	5	0	0
0	0	0	0	0	0	0	2	0	0	0
0	1	0	0	0	0	0	1	0	0	0
0	0	0	0	0	0	0	1	0	0	0
0	0	0	0	0	0	0	0	0	0	0
0	0	0	0	0	0	0	0	0	0	0

0	0	0	0	0	0	0	0	0	0	0
0	0	0	0	0	0	0	0	0	0	0
0	0	0	0	0	0	0	0	0	0	0
0	0	0	0	0	0	0	1	0	0	0
0	0	0	0	0	0	0	0	0	0	0
0	0	0	0	0	0	0	1	0	0	0
0	0	0	0	0	0	0	0	1	0	0
0	0	0	0	0	0	0	1	0	0	0
0	0	0	0	0	0	0	0	0	0	0
0	0	0	0	0	0	0	1	0	0	0
0	0	0	0	0	0	0	0	0	0	0
0	1	0	0	0	0	0	0	0	0	0
0	0	0	0	0	0	0	1	0	0	0
0	0	0	0	0	0	0	1	0	0	0
0	0	0	0	0	0	1	1	0	0	0
0	2	0	0	0	0	0	0	0	0	0
0	0	0	0	0	0	0	0	0	0	0
0	0	0	0	0	0	0	0	0	0	0
0	0	0	0	0	0	0	0	0	0	0
0	0	0	0	0	0	0	0	0	0	0
0	0	0	0	0	0	0	0	0	0	0
0	0	0	0	0	0	0	1	0	0	0
0	1	0	0	0	0	0	3	0	0	0
0	0	0	0	0	0	0	0	0	0	0
0	0	0	0	0	0	0	1	0	0	0
0	0	0	0	0	0	0	0	0	0	0
0	0	0	0	0	0	0	0	0	0	0
0	0	0	0	0	0	0	0	0	0	0
0	0	0	0	0	0	0	0	0	0	0

0	0	0	0	0	0	0	0	0	0	0
0	0	0	0	0	0	0	1	0	0	0
0	0	0	0	0	0	0	1	0	0	0
0	1	0	0	0	0	0	0	0	0	0
0	0	0	0	0	0	0	1	0	0	0
0	0	0	0	0	0	0	1	0	0	0
0	0	0	0	0	0	0	0	0	0	0
0	0	0	0	0	0	0	2	0	0	0
0	0	0	1	0	0	0	0	0	0	0
0	0	0	0	0	0	0	0	0	0	0
0	0	0	0	0	0	0	0	0	0	0
0	0	0	0	0	0	0	0	0	0	0
0	0	0	0	0	0	0	0	0	0	0
0	0	0	0	0	0	0	1	0	0	0
0	0	0	0	0	0	0	0	0	0	0
0	0	0	0	0	0	0	0	0	0	0
0	0	0	0	0	0	0	0	0	0	0
0	0	0	0	0	0	0	1	1	0	0
0	0	0	0	0	0	0	0	0	0	0
0	1	0	0	0	0	0	0	0	0	0
0	1	0	0	0	0	0	1	0	0	0
0	0	0	0	0	0	0	0	0	0	0
0	0	0	0	0	0	0	1	0	0	0
0	0	0	0	0	0	0	0	0	0	0
0	1	0	0	0	0	0	2	0	0	0
0	0	0	0	0	0	0	1	0	0	0
0	0	0	0	0	0	0	0	0	0	0
0	0	0	0	0	0	0	1	0	0	0
0	0	0	0	0	0	0	0	0	0	0

0	0	0	0	0	0	0	0	0	0	0
0	0	0	0	0	0	0	1	0	0	0
0	0	0	0	0	0	0	1	0	0	0
0	0	0	0	0	0	0	0	0	0	0
0	0	0	0	0	0	0	2	0	0	0
0	0	0	0	0	0	0	2	0	0	0
0	0	0	0	0	0	0	0	0	0	0
0	0	0	0	0	0	0	0	0	0	0
0	0	0	0	0	0	0	1	0	0	0
0	0	0	0	0	0	0	0	0	0	0
0	0	0	0	0	0	0	0	0	0	0
0	0	0	0	0	0	0	0	0	0	0
0	0	0	0	0	0	0	0	0	0	0
0	0	0	0	0	0	0	0	0	0	0
0	0	0	1	0	0	0	3	0	0	0
0	0	0	0	0	0	0	0	1	0	0
0	0	0	0	0	0	0	2	0	0	0
0	0	0	0	0	0	0	0	0	0	0
0	0	0	0	0	0	0	1	0	0	0
0	0	0	0	0	0	0	0	0	0	0
0	0	0	0	0	0	0	0	0	0	0
0	0	0	0	0	0	0	2	0	0	0
0	0	0	0	0	0	0	0	0	0	0
0	0	0	0	0	0	0	0	0	0	0
0	0	0	0	0	0	0	0	0	0	0
0	0	0	0	0	0	0	0	0	0	0
0	0	0	0	0	0	0	0	0	0	0
0	0	0	0	0	0	0	0	0	0	0
0	0	0	0	0	0	0	0	0	0	0

0	0	0	0	0	0	0	1	0	0	0
0	0	0	0	0	0	0	0	0	0	0
0	0	0	0	0	0	0	0	0	0	0
0	0	0	0	0	0	0	1	0	0	0
0	0	0	0	0	0	0	2	0	0	0
0	0	0	0	0	0	0	0	1	0	0
0	0	0	0	0	0	0	1	0	0	0
0	0	0	0	0	0	0	0	1	0	0
0	1	0	0	0	0	0	0	0	0	0
0	0	0	0	0	0	0	0	0	0	0
0	0	0	0	0	0	0	0	0	0	0
0	1	0	0	0	0	0	2	0	0	0
0	0	0	0	0	0	0	2	3	0	0
0	1	0	0	0	0	0	1	0	0	0
0	0	0	0	0	0	0	0	0	0	0
0	0	0	0	0	0	0	0	0	0	0
0	0	0	0	0	0	0	0	0	0	0
0	0	0	0	0	0	0	0	0	0	0
0	1	0	0	0	0	0	0	0	0	0
0	0	0	0	0	0	0	0	1	0	0
0	0	0	0	0	0	0	1	1	0	0
0	0	0	0	0	0	0	0	0	0	0
0	0	0	0	0	0	0	2	1	0	0
0	1	0	0	0	0	0	0	0	0	0
0	0	0	0	0	0	0	1	0	0	0
0	0	0	0	0	0	0	0	1	0	0
0	0	0	0	0	0	0	0	0	0	0
0	0	0	0	0	0	0	1	0	0	0
0	0	0	0	0	0	0	0	0	0	0

0	1	0	0	0	0	0	1	1	0	0
0	0	0	0	0	0	0	0	0	0	0
0	1	0	0	0	0	0	0	0	0	0
0	1	0	0	0	0	0	2	0	0	0
0	0	0	0	0	0	0	3	0	0	0
0	1	0	0	0	0	0	3	0	0	0
0	0	0	0	0	0	0	1	0	0	0
0	0	0	0	0	0	0	0	0	0	0
0	0	0	0	0	0	0	0	0	0	0
0	0	0	0	0	0	0	1	0	0	0
0	0	0	0	0	0	0	1	0	0	0
0	0	0	0	0	0	0	0	0	0	0
0	0	0	0	0	0	0	1	0	0	0
0	0	0	0	0	0	0	0	0	0	0
0	0	0	0	0	0	0	0	0	0	0
0	0	0	0	0	0	0	0	0	0	0
0	0	0	0	0	0	0	0	0	0	0
0	0	0	0	0	0	0	0	0	0	0
0	0	0	0	0	0	0	0	0	0	0
0	0	0	0	0	0	0	1	0	0	0
0	0	0	0	0	0	0	0	0	0	0
0	0	0	0	0	0	0	0	0	0	0
0	0	0	0	0	0	0	0	0	0	0
0	0	0	0	0	0	0	0	0	0	0
0	0	0	0	0	0	0	1	0	0	0
0	0	0	0	0	0	0	1	0	0	0
0	0	0	0	0	0	0	0	0	0	0
0	0	0	0	0	0	0	0	0	0	0
0	0	0	0	0	0	0	1	0	0	0

0	0	0	0	0	0	0	2	0	0	0
0	0	0	0	0	0	0	0	0	0	0
0	0	0	0	0	0	0	1	0	0	0
0	0	0	0	0	0	0	0	0	0	0
0	0	0	0	0	0	0	0	0	0	0
0	0	0	0	0	0	0	0	0	0	0
0	0	0	0	0	0	0	1	0	0	0
0	0	0	0	0	0	0	0	0	0	0
0	0	0	0	0	0	0	0	1	0	0
0	0	0	0	0	0	0	2	0	0	0
0	0	0	0	0	0	0	0	0	0	0
0	1	0	0	0	0	0	2	0	0	0
0	1	0	0	0	0	0	0	0	0	0
0	0	0	0	0	0	0	0	0	0	0
0	0	0	0	0	0	0	0	0	0	0
0	1	0	0	0	0	1	0	1	0	0
0	0	0	0	0	0	0	1	0	0	0
0	1	0	0	0	0	0	1	0	0	0
0	2	0	0	0	0	0	0	0	0	0
0	0	0	0	0	0	0	0	0	0	0
0	0	0	0	0	0	0	2	0	0	0
0	0	0	0	0	0	0	1	0	0	1
0	0	0	0	0	0	0	1	0	0	0
0	0	0	0	0	0	0	0	0	0	0
0	0	0	0	0	0	0	0	0	0	0
0	0	0	0	0	0	0	0	0	0	0
0	0	0	0	0	0	0	0	0	0	0
0	0	0	0	0	0	2	0	0	0	0
0	0	0	0	0	0	0	0	0	0	0

0	1	0	0	0	0	0	0	0	0	1
0	1	0	0	0	0	0	0	0	0	0
0	0	0	0	0	0	0	0	0	0	0
0	0	0	0	0	0	0	0	1	0	0
0	0	0	0	0	0	0	0	0	0	0
0	1	0	0	0	0	0	0	0	0	0
0	0	0	0	0	0	0	0	0	0	0
0	0	0	0	0	0	0	1	0	0	0
0	0	0	0	0	0	0	1	2	0	0
0	2	0	0	0	0	0	0	0	0	0
0	0	0	0	0	0	0	0	0	0	0
0	0	0	0	0	0	0	0	0	0	1
0	0	0	0	0	0	0	0	0	0	0
0	1	0	0	0	0	0	1	0	0	0
0	0	0	0	0	0	0	0	0	0	0
0	0	0	0	0	0	0	0	0	0	0
0	0	0	0	0	0	0	1	0	0	0
0	0	0	0	0	0	1	1	0	0	0
0	1	0	0	0	0	0	0	0	0	0
0	0	0	0	0	0	0	0	0	0	0
0	0	0	0	0	0	0	2	0	0	0
0	0	0	0	0	0	0	0	0	0	0
0	0	0	0	0	0	0	0	0	0	0
0	0	0	0	0	0	0	1	0	0	0
0	0	0	0	0	0	0	1	0	0	0
0	0	0	0	0	0	0	1	0	0	0
0	0	0	0	0	0	0	0	0	0	0
0	0	0	0	0	0	0	2	0	0	0
0	0	0	0	0	0	0	1	0	0	0

0	1	0	0	0	0	0	0	0	0	0
0	1	0	0	0	0	0	2	0	0	0
0	2	0	0	0	0	0	0	0	0	0
0	0	0	0	0	0	0	0	0	0	0
0	0	0	0	0	0	0	1	0	0	0
0	0	0	0	0	0	0	1	0	0	0
0	1	0	0	0	0	0	3	0	0	0
0	0	0	0	0	0	0	1	0	0	0
0	1	0	0	0	0	0	0	0	0	0
0	0	0	0	0	0	0	1	0	0	0
0	4	0	0	0	0	0	2	0	0	0
0	3	0	0	0	0	0	0	0	0	0
0	0	0	0	0	0	0	0	0	0	0
0	2	0	0	0	0	0	1	0	0	0
0	0	0	0	0	0	0	1	0	0	0
0	2	0	0	0	0	0	0	0	0	1
0	0	0	0	0	0	0	1	0	0	0
0	0	0	0	0	0	0	1	0	0	0
0	0	0	0	0	0	0	0	0	0	0
0	0	0	0	0	0	0	1	0	0	1
0	0	0	0	0	0	0	1	0	0	0
0	0	0	0	0	0	0	0	0	0	0
0	1	0	0	0	0	0	0	0	0	0
0	0	0	0	0	0	0	0	0	0	0
0	0	0	0	0	0	0	1	1	0	0
0	0	0	0	0	0	0	2	0	0	0
0	0	0	0	0	0	0	0	0	0	0
0	0	0	0	0	0	0	2	0	0	0
0	0	0	0	0	0	0	1	0	0	0

0	0	0	0	0	0	0	0	0	0	0
0	0	0	0	0	0	0	0	0	0	0
0	0	0	0	0	0	0	1	0	0	0
0	0	0	0	0	0	0	2	0	0	0
0	1	0	0	0	0	0	0	0	0	0
0	1	0	0	0	0	1	2	0	0	0
0	0	0	0	0	0	0	2	0	0	0
0	0	0	0	0	0	0	2	0	0	0
0	0	0	0	0	0	0	0	2	0	0
0	0	0	0	0	0	0	0	0	0	0
0	2	0	0	0	0	0	4	1	0	0
0	1	0	0	0	0	0	2	0	0	0
0	0	0	0	0	0	0	0	0	0	0
0	0	0	0	0	0	0	0	0	0	0
0	0	0	0	0	0	0	0	0	0	0
0	3	0	0	0	0	0	0	2	0	0
0	0	0	0	0	0	0	0	0	0	0
0	0	0	0	0	0	0	1	0	0	0
0	0	0	0	0	0	0	1	0	0	0
0	1	0	0	0	0	0	1	2	0	0
0	0	0	0	0	0	0	2	1	0	0
0	0	0	0	0	0	1	0	1	1	0
0	0	0	0	0	0	0	1	0	0	0
0	1	0	0	0	0	0	2	0	0	0
0	0	0	0	0	0	0	0	1	0	0
0	0	0	0	0	0	0	0	0	0	0
0	0	0	0	0	0	0	1	0	0	0
0	1	0	0	0	0	0	4	0	0	0
0	0	0	0	0	0	0	1	0	0	0

0	0	0	0	0	0	0	0	0	0	0
0	0	0	0	0	0	0	1	1	0	0
0	0	0	0	0	0	0	0	0	0	0
0	0	0	0	0	0	0	0	0	0	0
0	0	0	0	0	0	0	1	0	0	0
0	0	0	0	0	0	0	1	0	0	0
0	0	0	0	0	0	0	0	0	0	0
0	0	0	0	0	0	0	1	0	0	0
0	0	0	0	0	0	0	0	0	0	0
0	1	0	0	0	0	0	0	1	1	0
0	0	0	0	0	0	0	1	0	0	0
0	0	0	0	0	0	0	1	1	0	0
0	0	0	0	0	0	0	0	0	0	0
0	0	0	0	0	0	0	0	0	0	0

dexami	dilsub	divari	dorvil	eatoni	edward	euchon	euttri	exofal	exogon	exowai
0	0	0	0	0	0	0	0	0	0	0
0	0	0	0	0	0	0	0	0	0	0
0	0	0	0	0	0	0	0	0	0	0
0	0	0	0	0	0	0	0	0	0	0
0	1	0	0	0	0	0	0	0	0	0
0	0	0	0	0	0	0	0	0	0	0
0	0	0	0	0	0	0	0	0	0	0
0	0	0	0	0	0	1	0	0	0	0
0	0	0	0	0	0	0	0	0	0	0
0	0	0	0	0	0	1	0	0	0	0
0	0	0	0	0	0	0	0	1	0	1
0	0	0	0	0	0	0	0	0	0	0
0	0	0	0	0	0	2	0	2	0	0
0	0	0	0	0	0	0	0	0	0	0
0	0	0	0	0	0	2	0	0	0	0
0	0	0	0	0	0	0	0	0	0	0
0	0	0	0	0	0	0	1	0	0	0
0	0	0	0	0	0	0	0	0	0	0
0	0	0	0	0	0	0	0	0	0	0
0	0	0	0	0	0	1	0	0	0	0
0	0	0	0	0	0	0	0	0	0	0
0	0	0	0	0	0	2	1	1	0	0
0	0	0	0	0	0	0	0	0	0	0
0	0	0	0	0	0	0	0	1	0	0
0	0	0	0	0	0	0	0	0	0	0
0	0	0	0	0	0	10	0	0	0	0
0	0	0	0	0	0	0	0	0	0	0
0	1	0	0	0	0	0	0	0	0	0

0	0	0	0	0	0	1	1	0	0	0
0	0	0	0	0	0	5	0	0	0	0
0	0	0	0	0	0	13	0	0	0	0
0	0	0	0	0	0	10	0	0	0	0
0	0	0	0	0	0	1	0	0	0	0
0	0	0	0	0	0	1	0	0	0	0
0	0	0	0	0	0	0	0	0	0	0
0	0	0	0	0	0	1	0	0	0	0
0	0	0	0	0	0	0	0	0	0	0
0	0	0	0	0	0	0	0	0	0	0
0	0	0	0	0	0	0	0	0	0	0
0	0	0	0	0	0	0	0	0	0	0
0	0	0	0	0	0	0	0	0	0	0
0	0	0	0	0	0	0	0	0	0	0
0	0	0	0	0	0	0	0	0	0	0
0	0	0	0	0	0	0	0	0	0	0
0	0	0	0	1	0	0	0	0	0	0
0	0	0	0	0	0	0	0	0	0	0
0	0	0	0	0	0	0	0	0	0	0
0	0	0	0	0	0	0	0	0	0	0
0	0	0	0	0	0	0	0	0	0	0
0	0	0	0	0	0	2	0	0	0	0
0	0	0	0	0	0	1	0	2	0	0
0	0	0	0	0	0	0	0	0	0	0
0	0	0	0	0	0	3	0	0	0	0
0	0	0	0	0	0	1	0	0	0	0
0	0	0	0	0	0	0	0	0	0	0
0	0	0	0	0	0	0	0	2	0	0
0	0	0	0	0	0	3	0	0	0	0

0	0	0	0	0	0	1	1	1	0	0
0	0	0	0	0	0	0	0	0	0	0
0	0	0	0	0	0	0	0	1	0	0
0	0	0	0	0	0	0	0	0	0	0
0	0	0	0	0	0	0	0	1	0	0
0	0	0	0	0	0	0	0	1	0	0
0	0	0	0	0	0	3	0	0	0	2
0	0	0	0	0	0	3	0	0	0	0
0	0	0	0	0	0	1	0	0	0	0
0	0	0	0	0	0	0	1	1	0	0
0	0	0	0	0	0	1	0	0	0	0
0	0	0	0	0	0	1	1	2	0	0
0	0	0	0	0	0	0	0	0	0	0
0	0	0	0	0	0	1	0	0	0	0
0	0	0	0	0	0	0	0	2	0	0
0	0	0	0	0	0	0	0	1	0	0
0	0	0	0	0	0	2	0	0	0	0
0	0	0	0	0	0	0	0	4	0	0
0	0	0	0	0	0	0	1	0	0	0
0	0	0	0	0	0	5	0	0	0	0
0	0	0	0	0	0	0	0	1	0	0
0	0	0	0	0	0	2	0	0	0	0
0	0	0	0	0	0	0	0	0	0	0
0	0	0	0	0	0	0	0	0	0	0
0	0	0	0	0	0	0	0	1	0	0
0	0	0	0	0	0	0	0	1	0	0
0	0	0	0	0	0	2	1	2	0	0
0	0	0	0	0	0	0	0	0	0	0
0	0	0	0	0	0	4	0	0	0	0

0	0	0	0	0	0	17	0	0	0	0
0	0	0	0	0	0	0	0	0	0	0
0	0	0	0	0	0	59	0	0	0	0
0	0	0	0	0	0	39	0	0	0	0
0	0	0	0	0	0	0	0	0	0	0
0	1	0	0	0	0	0	0	0	0	0
0	0	0	0	0	0	0	0	0	0	0
0	0	0	0	1	0	0	0	0	0	0
0	0	0	0	0	0	0	0	0	0	0
0	0	0	0	0	0	22	0	1	0	0
0	0	0	0	0	0	0	0	0	0	0
0	0	0	0	0	0	0	0	0	0	0
0	0	0	0	0	0	1	0	2	0	0
0	0	0	0	0	0	0	0	2	0	0
0	1	0	0	0	0	0	0	0	0	0
0	0	0	0	0	0	0	0	1	0	0
0	0	0	0	0	0	0	0	0	0	0
0	0	0	0	0	0	1	0	0	0	0
0	0	0	0	0	0	0	0	0	0	0
0	0	0	0	0	0	0	0	0	0	0
0	0	0	0	0	0	0	0	1	0	0
0	0	0	0	0	0	1	0	0	0	0
0	0	0	0	0	0	21	0	0	0	0
0	0	0	0	0	0	22	0	0	0	0
0	0	0	1	1	0	86	0	0	0	0
0	0	0	0	0	0	5	0	3	0	0
0	0	0	0	0	0	3	0	4	0	0
0	0	0	0	2	0	4	0	4	0	0
0	0	0	0	0	0	2	0	0	0	0

0	0	0	0	0	0	2	0	0	0	0
0	0	0	0	0	0	0	0	0	0	0
0	0	0	0	0	0	0	0	0	0	0
0	0	0	0	0	0	0	0	0	0	0
0	0	0	0	0	0	0	0	5	0	0
0	0	0	0	0	0	0	0	0	0	0
0	1	0	0	0	0	0	0	0	0	0
0	0	0	0	0	0	0	0	0	0	0
0	0	0	0	0	0	1	0	1	0	0
0	0	0	0	0	0	0	0	0	0	0
0	0	0	0	0	0	0	0	0	0	0
0	0	0	0	0	0	0	0	0	0	0
0	0	0	0	0	0	0	0	0	0	0
0	0	0	0	0	0	0	0	0	0	0
0	0	0	0	0	0	0	0	0	0	0
0	0	0	0	0	0	0	0	0	0	0
0	0	0	0	1	0	0	0	0	0	0
0	0	0	0	0	0	0	0	0	0	0
0	0	0	0	0	0	0	0	0	0	0
0	1	0	0	0	0	0	0	0	0	0
0	0	0	0	0	0	0	0	0	0	0
0	0	0	0	0	0	0	0	0	0	0
0	0	0	0	0	0	0	0	0	0	0
0	0	0	0	0	0	0	0	0	0	0
0	0	0	0	0	0	0	0	1	1	0
0	0	0	0	0	0	0	0	0	0	0
0	0	0	0	0	0	0	0	0	0	0
0	0	0	0	0	0	0	0	0	0	0
0	0	0	0	0	0	0	0	0	0	0

0	0	0	0	0	0	0	0	0	0	0
0	0	0	0	0	0	0	0	0	0	0
0	0	0	0	0	0	0	0	0	0	0
0	0	0	0	0	0	0	0	0	0	0
0	0	0	0	0	0	0	0	0	0	0
0	0	0	0	0	0	0	0	0	0	0
0	1	0	0	0	0	0	0	0	0	0
0	0	0	0	0	0	0	0	0	0	0
0	0	0	0	0	0	0	0	0	0	0
0	0	0	0	0	0	0	0	1	0	0
0	0	0	0	0	0	0	0	2	0	0
0	0	0	0	0	0	0	0	1	0	0
0	0	0	0	0	0	0	0	0	0	0
0	0	0	0	0	0	0	0	0	0	0
0	1	0	0	0	0	0	0	0	0	0
0	0	0	0	0	0	0	0	0	0	0
0	0	0	0	0	0	0	0	0	0	0
0	0	0	0	0	0	0	0	0	0	0
0	0	0	0	0	0	0	0	0	0	0
0	1	0	0	0	0	0	0	0	0	0
0	0	0	0	0	0	0	0	0	0	0
0	3	0	0	0	0	0	0	0	0	0
0	0	0	0	0	0	0	0	0	0	0
0	1	0	0	0	0	0	0	0	0	0
0	0	0	0	0	0	0	0	0	0	0
0	0	0	0	0	0	0	0	0	0	0
0	0	0	0	0	0	0	0	0	0	0
0	0	0	0	0	0	0	0	0	0	0
0	0	0	0	0	0	0	0	1	0	0

0	0	0	0	0	0	0	0	0	0	0
0	0	0	0	0	0	0	0	0	0	0
0	0	0	0	0	0	0	0	0	0	0
0	0	0	0	0	0	0	0	0	0	0
0	0	0	0	0	0	0	0	1	0	0
0	0	0	0	0	0	0	0	0	0	0
0	0	0	0	0	0	0	0	0	0	0
0	0	0	0	0	0	0	0	0	0	0
0	0	0	0	0	0	0	0	1	0	0
0	1	0	0	0	1	0	0	0	0	0
0	0	0	0	0	0	0	0	0	0	0
0	0	0	0	1	0	0	0	0	0	0
0	0	0	0	0	0	0	0	0	0	0
0	0	0	0	0	0	0	0	0	0	0
0	0	0	0	0	0	0	0	0	0	0
0	0	0	0	0	0	0	0	2	0	0
0	0	0	0	0	0	0	0	1	0	0
0	0	0	0	1	0	0	0	0	0	0
0	0	0	0	0	0	2	0	0	0	0
0	0	0	0	0	0	0	0	0	0	0
0	0	0	0	0	0	0	0	2	0	0
0	0	0	0	0	0	0	0	0	0	0
0	0	0	0	0	0	0	0	0	0	0
0	0	0	0	0	0	0	0	2	0	0
0	0	0	0	0	0	1	0	0	0	0
0	0	0	0	0	0	0	0	0	0	0
0	0	0	0	0	0	0	0	0	0	0
0	0	0	0	0	0	1	0	2	0	0
0	0	0	0	0	0	0	0	1	0	0

0	1	0	0	0	0	0	0	0	0	0
0	0	0	0	0	0	0	0	6	0	0
0	0	0	0	0	0	0	0	0	0	0
0	0	0	0	0	0	0	0	2	0	0
0	0	0	0	0	0	0	0	3	0	0
0	0	0	0	0	0	0	0	1	0	0
0	0	0	0	0	0	0	0	0	0	0
0	0	0	0	0	0	0	0	1	0	0
0	0	0	0	0	0	0	0	0	0	0
0	0	0	0	0	0	0	0	1	0	0
0	0	0	0	0	0	0	0	0	0	0
0	0	0	0	0	0	0	0	0	0	0
0	0	0	0	0	0	0	0	0	0	0
0	0	0	0	0	0	0	0	0	0	0
0	0	0	0	0	0	0	0	0	0	0
0	0	0	0	0	0	0	0	0	0	0
0	0	0	0	0	0	0	0	1	0	0
0	0	0	0	0	0	0	0	0	0	0
0	0	0	0	0	0	0	0	0	0	0
0	0	0	0	0	0	0	0	0	0	0
0	0	0	0	0	0	0	0	0	0	0
0	0	0	0	0	0	0	0	0	0	0
0	1	0	0	0	0	0	0	0	0	0
0	0	0	0	0	0	0	0	2	0	0
0	0	0	0	0	0	0	0	1	0	0
0	0	0	0	0	0	0	0	2	0	0
0	0	0	0	0	0	0	0	0	0	0
0	0	0	0	0	0	0	0	1	0	0
0	0	0	0	0	0	0	0	0	0	0

0	0	0	0	0	0	0	0	0	0	0
0	0	0	0	0	0	0	0	0	0	0
0	0	0	0	0	0	0	0	0	0	0
0	0	0	0	0	0	0	0	0	0	0
0	0	0	0	0	0	0	0	0	0	0
0	0	0	0	0	0	0	0	0	0	0
0	0	0	0	0	0	0	0	0	0	0
0	0	0	0	0	0	0	0	0	0	0
0	1	0	0	0	0	0	0	0	0	0
0	0	0	0	0	0	0	0	2	0	0
0	0	0	0	0	0	0	0	0	0	0
0	0	0	0	0	0	0	0	1	0	0
0	0	0	0	0	0	0	0	1	0	0
0	0	0	0	0	0	0	0	0	0	0
0	0	0	0	0	0	0	0	0	0	0
0	0	0	0	0	0	0	0	0	0	0
0	0	0	0	0	0	0	0	0	0	0
0	0	0	0	0	0	0	0	0	0	0
0	0	0	0	0	0	0	0	0	0	0
0	0	0	0	0	0	0	0	0	0	0
0	0	0	0	0	0	0	0	0	0	0
0	0	0	0	0	0	0	0	0	0	0
0	0	0	0	0	0	0	0	1	0	0
0	0	0	0	0	0	0	0	0	0	0
0	0	0	0	0	0	0	0	0	0	0
0	0	0	0	0	0	0	0	0	0	0
0	0	0	0	1	0	0	0	1	0	0
0	1	0	0	0	0	0	0	0	0	0
0	1	0	0	0	0	0	0	0	0	0

0	0	0	0	0	0	0	0	0	0	0
0	0	0	0	0	0	2	0	0	0	0
0	0	0	0	0	0	0	0	0	0	0
0	0	0	0	0	0	0	0	0	0	0
0	0	0	0	0	0	0	0	0	0	0
0	0	0	0	0	0	0	0	0	0	0
0	0	0	0	0	0	2	0	0	0	0
0	0	0	0	0	0	1	0	0	0	0
0	0	0	0	0	0	1	0	0	0	0
0	0	0	0	0	0	1	0	0	0	0
0	0	0	0	0	0	0	0	0	0	0
0	0	0	0	0	0	0	0	0	0	1
0	0	0	0	0	0	0	0	0	0	1
0	0	0	0	0	0	1	0	1	0	0
0	0	0	0	0	0	1	0	0	0	0
0	0	0	0	0	0	9	0	0	0	0
0	0	0	0	0	0	5	0	0	0	0
0	0	0	0	0	0	7	0	0	0	0
0	0	0	0	0	0	2	0	0	0	0
0	0	0	0	0	0	0	0	3	0	0
0	0	0	0	0	0	0	0	0	0	0
0	0	0	0	0	0	9	0	0	0	0
0	0	0	0	0	0	1	0	1	0	0
0	0	0	0	0	0	0	0	1	0	0
0	0	0	0	0	0	0	0	1	0	0
0	0	1	0	0	0	1	0	0	0	0
2	0	0	0	0	0	4	0	0	0	0
0	0	0	0	0	0	0	0	0	0	0
0	0	0	0	0	0	4	0	0	0	0

0	0	0	0	0	0	0	0	0	0	0
0	0	0	0	0	0	24	1	0	0	0
0	0	0	0	0	0	6	0	0	0	0
1	0	0	0	0	0	33	0	0	0	0
0	0	0	0	0	0	30	0	0	0	0
0	0	0	0	0	0	8	1	0	0	0
0	0	0	0	0	0	2	0	0	0	0
0	0	0	0	0	0	2	0	0	0	0
0	0	0	0	0	0	6	0	0	0	0
0	0	0	0	0	0	17	0	0	0	0
0	0	0	0	0	0	11	0	0	0	0
0	0	0	0	0	0	10	1	0	0	0
0	0	0	0	0	0	18	0	0	0	0
0	0	0	0	0	0	6	0	0	0	0
2	0	0	0	0	0	9	0	0	0	0
0	0	0	0	0	0	9	0	0	0	0
0	0	0	0	0	0	8	0	0	0	0
0	0	0	0	0	0	12	0	0	0	0
0	0	0	0	0	0	14	0	0	0	0
0	0	0	0	0	0	3	0	0	0	0
0	0	0	0	0	0	3	0	0	0	0
0	0	0	0	0	0	14	0	0	0	0
0	0	0	0	0	0	1	0	0	0	0
0	0	0	0	0	0	31	0	0	0	0
0	0	0	0	0	0	23	0	0	0	0
0	0	0	0	0	0	6	0	0	0	0
0	0	0	0	0	0	11	0	0	0	0
0	0	0	0	0	0	6	0	0	0	0
0	0	0	0	0	0	1	0	0	0	0

0	0	0	0	0	0	1	0	0	0	0
0	0	0	0	0	0	0	0	0	0	0
0	0	0	0	0	0	1	0	0	0	0
0	0	0	0	0	0	0	0	0	0	0
0	0	0	0	0	0	1	0	0	0	0
0	0	0	0	0	0	1	0	0	0	0
0	0	0	0	0	0	0	0	0	0	0
0	0	0	0	0	0	0	0	0	0	0
0	0	0	0	0	0	0	0	0	0	0
0	0	0	0	0	0	1	2	0	0	0
0	0	0	0	0	0	7	0	0	0	0
0	0	0	0	0	0	3	0	0	0	0
0	0	0	0	0	0	1	0	0	0	0
0	0	0	0	0	0	0	0	2	0	0
0	0	0	0	0	0	1	0	0	0	0
0	0	0	0	0	0	0	0	0	0	0
0	0	0	0	0	0	0	0	0	0	0
0	0	0	3	0	0	1	0	0	0	0
0	0	0	0	0	0	1	0	0	0	0
0	0	0	0	0	0	0	0	0	0	0
0	0	0	0	0	0	0	0	2	0	0
0	0	0	0	0	0	0	0	0	0	0
0	0	0	0	0	0	0	0	0	0	0
0	0	0	0	0	0	0	0	1	0	0
0	0	0	0	0	0	0	0	1	0	0
0	0	0	0	0	0	0	0	0	0	0
0	0	0	0	0	0	0	0	1	0	0
0	0	0	0	0	0	0	0	0	0	0
0	0	0	0	0	0	0	0	0	0	0

0	0	0	0	0	0	0	0	0	0	0
0	0	0	0	1	0	0	0	4	0	0
0	0	0	0	0	0	0	0	1	0	0
0	0	0	0	0	0	0	0	0	0	0
0	0	0	0	0	0	0	0	2	0	0
0	0	0	0	0	0	0	0	0	0	0
0	0	0	0	1	1	0	0	0	0	0
0	0	0	0	0	0	0	0	1	0	0
0	0	0	0	0	0	0	0	1	0	0
0	0	0	0	0	0	0	0	1	0	0
0	0	0	0	0	1	0	0	0	0	0
0	0	0	0	0	0	0	0	0	0	0
0	0	0	0	2	0	0	0	1	0	0
0	0	0	0	0	0	0	0	1	0	0
0	0	0	0	0	0	0	0	0	0	0
0	0	0	0	0	0	0	0	1	0	0
0	0	0	0	0	0	0	0	0	0	0
0	0	0	0	0	0	0	0	0	0	0
0	0	0	0	0	0	0	0	0	0	0
0	0	0	0	0	0	0	0	0	0	0
0	0	0	0	0	0	0	0	1	0	0
0	0	0	0	0	0	0	0	0	0	0
0	0	0	0	0	0	0	0	0	0	0
0	0	0	0	0	0	0	0	0	0	0
0	0	0	0	0	0	0	0	0	0	0
0	0	0	0	0	0	0	0	1	0	0
0	0	0	0	0	0	0	0	0	0	0
0	0	0	0	0	0	0	0	0	0	0
0	0	0	0	0	0	0	0	0	0	0

0	0	0	0	0	0	0	0	0	0	0
0	0	0	0	0	0	0	0	0	0	0
0	0	0	0	0	0	0	0	0	0	0
0	0	0	0	0	0	0	0	0	0	0
0	0	0	0	0	0	0	0	0	0	0
0	0	0	0	0	0	0	0	0	0	0
0	0	0	0	0	0	0	0	0	0	0
0	0	0	0	0	0	0	0	0	0	0
0	0	0	0	0	0	0	0	0	0	0
0	0	0	0	0	0	0	0	0	0	0
0	0	0	0	0	0	0	0	0	0	0
0	0	0	0	0	0	0	0	0	0	0
0	0	0	0	0	0	0	0	0	0	0
0	0	0	0	0	0	0	0	0	0	0
0	0	0	0	0	0	0	0	3	0	0
0	0	0	0	0	0	0	0	0	0	0
0	0	0	0	0	0	0	0	0	0	0
0	0	0	0	0	0	0	0	1	0	0
0	0	0	0	0	0	0	0	1	0	0
0	0	0	0	0	0	0	0	0	0	0
0	0	0	0	0	0	0	0	1	0	0
0	0	0	0	0	0	0	0	0	0	0
0	0	0	0	0	0	0	0	1	0	0
diopat	dorvil	eteone	eunice	exochi	exogon	exoobt	exopla	exowai	flatwo	glycer
0	0	0	0	0	0	0	0	0	0	0
0	0	0	0	0	0	0	0	0	0	0
0	0	0	0	0	0	0	0	0	0	0
0	0	0	0	0	0	0	0	1	0	0

0	0	0	0	0	0	0	0	0	0	0
0	0	0	0	0	0	0	0	0	0	0
0	0	0	0	0	0	0	0	0	0	0
0	0	0	0	0	0	0	0	0	0	0
0	0	0	0	0	0	0	0	0	0	0
0	0	0	0	0	0	0	0	0	0	0
0	0	0	0	0	0	0	0	0	0	1
0	0	0	0	0	0	0	0	0	0	1
0	0	0	0	0	0	0	0	0	0	0
0	0	0	0	0	0	0	0	0	0	0
0	0	0	0	0	0	0	0	0	0	0
0	0	0	0	0	0	0	0	0	0	0
0	0	0	0	0	0	0	0	0	0	2
2	0	0	0	0	0	0	0	0	0	0
0	0	0	0	0	0	0	0	0	0	0
0	0	0	0	0	0	0	0	0	0	0
0	0	0	0	0	0	0	0	1	0	0
0	0	0	0	0	0	0	0	1	0	0
0	0	0	0	0	0	0	0	0	0	0
0	0	0	0	0	0	0	0	1	0	0
0	0	0	0	0	0	0	0	0	0	0
0	0	0	0	0	0	0	0	1	0	0
0	0	0	0	0	0	0	0	0	0	0
0	0	0	0	0	0	0	0	2	0	0
0	0	0	0	0	0	0	0	0	0	0
0	0	0	0	0	0	0	0	0	0	0
0	0	0	0	0	0	0	0	1	0	0
0	0	0	0	0	0	0	0	0	0	0
0	0	0	0	0	0	0	0	1	0	0

0	0	0	0	0	0	0	0	0	0	0
0	0	0	0	0	0	0	0	0	0	0
0	0	1	0	0	0	0	0	0	0	0
0	0	0	0	0	0	0	0	0	0	0
0	0	0	0	0	0	0	0	0	0	0
0	0	0	0	0	0	0	0	0	0	0
0	0	0	0	0	0	0	0	0	0	0
0	0	0	0	0	0	0	0	0	0	0
0	0	0	0	0	0	0	0	0	0	0
0	0	0	0	0	0	0	0	0	0	0
0	0	0	0	0	1	0	0	0	0	0
0	0	0	0	0	0	0	0	0	0	0
0	0	0	0	0	0	0	0	0	0	0
0	0	0	0	0	0	0	0	0	0	0
0	0	0	0	0	0	0	0	0	0	0
0	0	0	0	1	0	0	0	0	0	1
0	0	0	0	0	0	0	0	0	0	1
0	0	0	0	0	0	0	0	0	0	1
0	0	0	0	0	0	0	0	0	0	0
1	0	0	0	0	0	0	0	0	0	0
0	0	0	0	0	0	0	0	0	0	0
0	0	0	0	2	0	0	0	0	0	0
0	0	0	0	0	0	0	0	0	0	1
0	0	0	0	0	0	0	0	0	0	1
0	0	0	0	0	0	0	0	0	0	0
0	0	0	0	0	0	0	0	0	0	1
1	0	0	0	0	0	0	0	0	0	0
0	0	0	0	0	0	0	0	0	0	0
0	0	0	0	0	0	0	0	0	0	2

0	0	0	0	0	0	0	0	0	0	0
0	0	0	0	0	0	0	0	0	0	0
0	0	0	0	0	0	0	0	0	0	0
0	0	0	0	0	0	0	0	0	0	2
0	0	0	0	0	0	0	0	0	0	1
0	0	0	0	0	0	0	0	0	0	1
0	0	0	0	0	0	0	0	0	0	0
0	0	0	0	0	0	0	0	0	0	1
0	0	0	0	0	0	0	0	0	0	1
0	0	0	0	1	0	0	0	0	0	0
0	0	0	0	0	0	0	0	0	0	1
0	0	0	0	0	0	0	0	0	0	0
0	0	0	0	0	0	0	0	0	0	0
0	0	0	0	0	0	0	0	0	0	0
0	0	0	0	0	0	0	0	0	0	0
0	0	0	0	0	0	0	0	0	0	0
0	0	0	0	0	0	0	2	0	0	0
0	0	0	0	0	0	0	0	0	0	0
0	0	0	0	0	0	0	0	0	0	0
0	0	0	0	0	0	0	0	0	0	0
0	0	0	0	0	0	0	0	0	0	1
0	0	0	0	0	0	0	0	0	0	1
0	0	0	0	0	0	0	0	0	0	0
0	0	0	0	0	0	0	1	0	0	0
0	0	0	0	0	0	0	1	0	0	0
0	0	0	0	0	0	0	1	0	0	0
0	0	0	0	0	0	0	0	0	0	0
0	0	0	0	0	0	0	0	0	0	0
0	0	0	0	0	0	0	0	0	0	0

0	0	0	0	0	0	0	0	0	0	0
0	0	0	0	0	0	0	0	0	0	0
0	0	0	0	0	0	0	0	0	0	0
0	0	0	0	0	0	0	0	0	0	0
0	0	0	0	0	0	0	0	0	0	1
0	0	0	0	0	0	0	0	0	0	0
0	0	0	0	0	0	0	0	0	0	0
0	0	0	0	0	0	0	0	0	0	1
0	0	0	0	0	0	0	0	0	0	1
0	0	0	0	0	0	0	0	0	0	0
0	0	0	0	0	0	0	0	0	0	0
0	0	0	0	0	0	0	0	0	0	0
0	0	0	0	0	0	0	0	0	0	0
0	0	0	0	0	0	0	0	0	0	0
0	0	0	0	0	0	0	0	0	0	0
0	0	0	0	0	0	0	0	0	0	0
0	0	0	0	0	0	0	0	0	0	0
0	0	0	0	0	0	0	1	0	0	0
0	0	0	0	0	0	0	0	0	0	0
0	0	0	0	0	0	0	0	0	0	0
0	0	0	0	0	0	0	0	0	0	1
0	0	0	0	0	0	0	0	0	0	0
0	0	0	0	0	0	0	0	0	0	1
0	0	0	0	0	0	0	0	0	0	1
0	0	0	0	0	0	0	0	0	0	0
0	0	0	0	0	1	0	0	0	0	1
0	0	0	0	0	0	0	0	0	0	0
0	0	0	0	0	0	0	1	0	0	0
0	0	0	0	0	0	0	0	0	0	0

0	0	0	0	0	0	0	0	0	0	1
0	0	0	0	0	0	0	0	0	0	0
0	0	0	0	0	0	0	0	0	0	0
0	0	0	0	0	0	0	0	0	0	0
0	0	0	0	0	0	0	0	0	0	1
0	0	0	0	0	0	0	0	0	0	0
0	0	0	0	0	0	0	0	0	0	0
0	0	0	0	0	0	0	0	0	0	0
0	0	0	0	0	0	0	0	0	0	0
0	0	0	0	0	0	0	0	0	0	0
0	0	0	0	0	0	0	0	0	0	0
0	0	0	0	0	0	0	0	1	0	0
0	0	0	0	0	0	0	0	0	0	0
0	0	0	0	0	0	0	0	0	0	0
0	0	0	0	0	0	0	0	0	0	1
0	0	0	0	0	0	0	1	0	0	0
0	0	0	0	0	0	0	0	0	0	0
1	0	0	0	0	0	0	0	0	0	0
0	0	0	0	0	0	0	0	0	0	0
0	0	0	0	0	0	0	2	0	0	1
0	0	0	0	0	0	0	1	0	0	0
0	0	0	0	0	0	0	0	0	0	0
0	0	0	0	0	0	0	0	0	0	0
0	0	0	0	0	0	0	1	0	0	1
0	0	0	0	0	0	0	0	0	0	0
0	0	0	0	0	0	0	0	0	0	0
0	0	0	0	0	0	0	0	0	0	0
0	0	0	0	0	0	0	0	0	0	0
0	0	0	0	0	0	0	0	0	0	0

0	0	0	0	0	0	0	0	0	0	0
0	0	0	0	0	0	0	0	0	0	0
1	0	0	0	0	3	0	0	0	0	1
0	0	0	0	0	0	0	0	0	0	0
1	0	0	0	0	0	0	0	0	0	0
0	0	0	0	0	0	0	0	0	0	1
0	0	0	0	0	0	1	0	0	0	1
0	0	0	0	0	0	0	0	0	0	0
0	0	0	0	0	0	0	0	0	0	0
0	0	0	0	0	1	0	0	0	0	0
0	0	0	0	0	0	0	0	0	0	0
0	0	0	0	0	0	0	0	0	0	0
0	0	0	0	0	0	0	0	0	0	0
0	0	0	0	0	0	0	0	0	0	0
0	0	0	0	0	1	0	0	0	0	0
0	0	0	0	0	1	0	0	0	0	1
0	0	0	0	0	0	0	1	0	0	0
0	1	0	0	0	0	0	0	0	0	1
0	0	0	0	0	1	0	0	0	0	1
0	0	0	0	0	0	0	0	0	0	0
0	0	0	0	0	0	0	0	0	0	1
0	0	0	0	0	0	0	0	0	0	1
0	0	0	0	0	0	0	0	0	0	0
0	0	0	0	0	0	0	0	0	0	0
0	0	0	0	0	0	0	0	0	0	0
0	0	0	0	0	0	0	0	0	0	0
0	0	0	0	0	0	0	0	0	0	1
0	1	0	0	0	1	0	0	0	0	1
0	0	0	0	0	0	0	0	0	0	1

0	0	0	0	0	0	0	0	0	0	1
0	0	0	0	0	0	0	0	0	0	0
0	0	0	0	0	0	0	0	0	0	0
0	0	0	0	0	0	0	0	0	0	0
0	0	0	0	0	0	0	0	0	0	0
0	0	0	0	0	0	0	0	0	0	0
0	0	0	0	0	0	0	0	0	0	0
0	0	0	0	0	0	0	0	0	0	1
0	0	0	0	0	0	0	0	0	0	1
0	0	0	0	0	0	0	0	0	0	0
0	0	0	0	0	0	0	0	0	0	0
0	0	0	0	1	0	0	0	0	0	0
0	0	0	0	0	0	0	0	0	0	0
0	0	0	0	0	0	0	0	0	0	0
0	0	0	0	0	0	0	0	0	0	0
0	0	0	0	0	0	0	0	0	0	0
0	0	0	0	0	0	0	0	0	0	0
0	0	0	0	0	0	0	0	0	0	0
0	0	0	0	0	0	0	0	0	0	0
0	0	0	0	0	0	0	0	0	0	0
0	0	0	0	0	0	0	0	1	0	0
0	0	0	0	0	0	0	0	0	0	0
0	0	0	0	0	0	0	0	0	0	0
0	0	0	0	0	0	0	0	0	0	0
0	0	0	0	0	0	0	0	0	0	0
0	0	0	0	0	0	0	0	0	0	0
0	0	0	0	0	0	0	0	0	0	0
0	0	0	0	0	0	0	0	0	0	0
0	0	0	0	1	0	0	0	0	0	0

0	0	0	0	0	0	0	0	0	0	0
0	0	0	0	0	0	0	0	0	0	0
0	0	0	0	0	0	0	0	0	0	1
0	0	0	0	0	0	0	0	0	0	1
0	0	0	0	0	0	0	0	0	0	0
0	0	0	0	0	0	0	0	0	0	1
0	0	0	0	0	0	0	0	0	0	0
0	0	0	0	0	0	0	0	0	0	0
0	0	0	0	0	0	0	0	0	0	0
0	0	0	0	0	0	0	0	0	0	0
0	0	0	0	0	0	0	0	0	0	0
0	0	0	0	0	0	0	0	0	0	0
0	0	0	0	0	0	0	0	0	0	1
0	0	0	0	0	0	0	0	0	0	1
0	0	0	0	0	0	0	0	0	0	0
0	0	0	0	0	0	0	0	0	0	1
0	0	0	0	0	0	0	0	0	0	0
0	0	0	0	0	0	0	0	0	0	0
0	0	0	0	0	0	0	0	0	0	0
0	0	0	0	0	0	0	0	0	0	0
0	0	0	0	0	0	0	0	0	0	1
0	0	0	0	0	0	0	0	0	0	0
0	0	0	0	0	0	0	0	0	0	0
0	0	0	0	0	0	0	0	0	0	0
0	0	0	0	0	0	0	0	0	0	1
1	0	0	0	0	0	0	0	0	0	0
0	0	0	0	0	0	0	0	0	0	0
0	0	0	0	0	0	0	0	0	0	0
0	0	0	0	0	0	0	0	0	0	1

0	0	0	0	0	0	0	0	0	0	0
0	0	0	0	0	0	0	0	0	0	0
0	0	0	0	0	0	0	0	0	0	2
0	0	0	0	0	0	0	0	0	0	0
0	0	0	0	0	0	0	0	0	0	0
0	0	0	0	1	0	0	0	0	0	0
0	0	0	0	0	0	0	0	0	2	0
0	0	0	0	0	0	0	0	0	0	0
0	0	0	0	0	0	0	0	0	0	1
0	0	0	0	0	0	0	0	0	0	0
0	0	0	0	0	0	0	0	0	0	0
0	0	0	0	0	0	0	0	0	0	0
0	0	0	0	0	0	0	0	0	0	0
0	0	0	0	0	0	0	0	0	0	0
0	0	0	0	0	0	0	0	0	0	0
0	0	0	0	0	0	0	0	0	0	0
0	0	0	0	0	0	0	0	0	0	0
0	0	0	0	0	0	0	0	0	0	1
0	0	0	0	0	0	0	0	0	0	1
0	0	0	0	0	0	0	0	0	0	0
0	0	0	0	0	0	0	0	0	0	0
0	0	0	0	0	0	0	1	0	0	0
0	0	0	0	0	0	0	0	0	0	0
0	0	0	0	0	0	0	0	0	0	0
0	0	0	0	0	0	0	0	0	0	0
0	0	0	0	0	0	0	0	0	0	0
0	0	0	0	0	0	0	1	0	0	0
0	0	0	0	0	0	0	0	0	0	0
0	0	0	0	0	0	0	0	0	0	0

0	0	0	0	0	0	0	0	0	0	0
0	0	0	0	0	0	0	0	0	0	0
0	0	0	0	0	0	0	2	0	0	0
0	0	0	0	0	0	0	0	0	0	0
0	0	0	0	0	0	0	0	0	0	0
0	0	0	0	0	0	0	0	0	0	0
0	0	0	0	0	0	0	0	0	0	0
0	0	0	0	0	0	0	0	0	0	0
0	0	0	0	0	0	0	0	0	0	0
0	0	0	0	0	0	0	0	0	0	0
0	0	0	0	0	0	0	0	0	0	0
0	0	0	0	0	0	0	0	0	0	0
0	0	0	0	0	0	0	0	0	0	0
0	0	0	0	0	0	0	0	0	0	0
0	0	0	0	0	0	0	0	0	0	0
0	0	0	0	0	0	0	0	0	0	0
0	0	0	0	0	0	0	0	0	0	0
0	0	0	0	0	0	0	0	0	0	0
0	0	0	0	0	0	0	0	0	0	0
0	0	0	0	0	0	0	0	0	0	0
0	0	0	0	0	0	0	0	0	0	0
0	0	0	0	0	0	0	0	0	0	0
0	0	0	0	0	0	0	0	0	0	1
0	0	0	0	0	0	0	0	0	0	0
0	0	0	0	0	0	0	0	0	0	0
0	0	0	0	0	0	0	0	0	0	0
0	0	0	0	0	0	0	0	0	0	0
0	0	0	0	0	0	0	0	0	0	1
2	0	0	0	0	0	0	0	0	0	1

1	0	0	0	0	0	0	0	0	0	0
0	0	0	0	0	0	0	0	0	0	0
0	0	0	0	0	0	0	0	0	0	0
0	0	0	0	0	0	0	0	0	0	0
0	0	0	0	0	0	0	0	0	0	0
0	0	0	0	0	0	0	0	0	0	0
0	0	0	0	0	0	0	0	0	0	0
0	0	0	0	0	0	0	0	0	0	0
0	0	0	0	0	0	0	0	0	0	0
0	0	0	0	0	0	0	0	0	0	0
0	0	0	0	0	0	0	0	0	0	1
0	0	0	0	0	0	0	0	0	0	1
0	0	0	0	0	0	0	0	0	0	0
0	0	0	0	0	0	0	0	0	0	0
0	0	0	0	0	0	0	0	0	0	0
0	0	0	0	0	0	0	0	0	0	0
0	0	0	0	0	0	0	0	0	0	1
0	0	0	0	0	0	0	0	0	0	0
0	0	0	0	0	0	0	0	0	0	0
0	0	0	0	0	0	0	0	0	0	0
0	0	0	0	0	0	0	0	0	0	1
0	0	0	0	0	0	0	0	0	0	0
0	0	0	0	0	0	0	0	0	0	0
0	0	0	0	0	0	0	0	0	0	0
0	0	0	0	0	0	0	0	0	0	0
0	0	0	0	0	0	0	0	0	0	0
0	0	0	0	0	0	0	0	0	0	0
0	0	0	0	0	0	0	0	0	0	1
0	0	0	0	0	0	0	0	0	0	0

0	0	0	0	0	0	0	0	0	0	0
0	0	0	0	0	0	0	0	0	0	0
0	0	0	0	0	0	0	0	0	0	0
0	0	0	0	0	0	0	0	0	0	0
0	0	0	0	0	0	0	0	0	0	0
0	0	0	0	0	0	0	0	0	0	0
0	0	0	0	0	0	0	0	0	0	0
0	0	0	0	0	0	0	0	0	0	0
0	0	0	0	0	0	0	0	0	0	0
0	0	0	0	0	0	0	0	0	0	0
0	0	0	0	0	0	0	0	0	0	1
0	0	0	0	0	0	0	0	0	0	1
0	0	0	0	0	0	0	0	0	0	0
0	0	0	0	0	0	0	0	0	0	0
0	0	0	0	0	0	0	0	0	0	0
0	0	0	0	0	0	0	0	0	0	1
0	0	0	0	0	0	0	0	0	0	0
0	0	0	0	0	0	0	0	0	0	0
0	0	0	0	0	0	0	0	0	0	0
0	0	0	0	0	0	0	0	0	0	0
0	0	0	0	0	0	0	0	0	0	0
0	0	0	0	0	0	0	0	0	0	0
0	0	0	0	0	0	0	0	0	0	0
0	0	0	0	0	0	0	0	0	0	0
0	0	0	0	1	0	0	0	0	0	0
0	0	0	0	0	0	0	0	0	0	0
0	0	0	0	0	0	0	0	0	0	1
0	0	0	0	0	0	0	0	0	0	0
0	0	0	0	0	0	0	0	0	0	1

0	0	0	0	0	0	0	0	0	0	0
0	0	0	0	0	0	0	0	0	0	0
0	0	0	0	0	0	0	0	0	0	0
0	0	0	0	0	0	0	0	0	0	1
0	0	0	0	0	0	0	0	0	0	0
0	0	0	0	0	0	0	0	0	0	1
0	0	0	0	1	0	0	0	0	0	0
0	0	0	0	0	0	0	0	0	0	0
0	0	0	0	0	0	0	0	0	0	0
0	0	0	0	0	0	0	0	0	0	0
1	0	0	0	0	0	0	0	0	0	0
0	0	0	0	0	0	0	0	0	0	1
0	0	0	0	0	0	0	0	0	0	0
0	0	0	0	0	0	0	0	0	0	1
0	0	0	0	0	0	0	0	0	0	0
0	0	0	0	1	0	0	0	0	0	1
0	0	0	0	0	0	0	0	0	0	0
0	0	0	0	0	0	0	1	0	0	1
0	0	0	0	0	0	0	0	0	0	1
0	0	0	0	0	0	0	0	0	0	0
0	0	0	0	0	1	0	2	0	0	0
0	0	0	0	0	0	0	0	0	0	0
0	0	0	0	0	0	0	0	0	0	1
0	0	0	0	0	1	0	0	0	0	0
0	0	0	0	0	0	0	0	0	0	0
0	0	0	0	0	0	0	0	0	0	0
0	0	0	0	0	0	0	0	0	0	1
0	0	0	0	0	0	0	0	0	0	0
0	0	0	0	0	0	0	0	0	0	0

0	0	0	0	0	0	0	0	0	0	1
0	0	0	0	0	0	0	1	0	0	1
0	0	0	0	0	0	0	0	0	0	1
0	0	0	1	0	0	0	0	0	0	0
0	0	0	0	0	0	0	0	0	0	0
0	0	0	0	0	0	0	0	0	0	0
0	0	0	0	0	0	0	0	0	0	0
0	0	0	0	0	0	0	0	0	0	0
0	0	0	0	0	0	0	0	0	1	0
0	0	0	0	0	0	0	0	0	0	0
0	0	0	0	1	0	0	0	0	0	1
0	0	0	0	0	0	0	0	0	0	0
0	0	0	0	0	0	0	0	0	0	0
0	0	0	0	0	0	0	0	0	0	0
0	0	0	0	0	0	0	0	0	0	0
0	0	0	0	0	0	0	0	0	0	0
0	0	0	0	0	0	0	0	0	0	0
0	0	0	0	0	0	0	0	0	0	0
0	0	0	0	0	0	0	0	0	0	0

hemhir	hetfil	laspar	levgra	lumbri	lysian	maclil	macste	magdak	melawa	methal
0	3	1	0	0	0	0	0	0	0	0
0	0	4	0	3	22	5	0	0	0	0
0	0	0	0	0	0	2	0	0	0	0
0	2	7	0	0	5	0	0	0	0	0
0	0	6	0	5	7	3	0	0	0	0
0	0	1	0	1	12	4	0	0	0	0
0	1	2	0	1	2	4	0	0	0	0
0	2	4	0	0	9	10	0	0	0	0

0	2	0	0	0	6	4	0	0	0	0
0	0	0	0	2	25	4	0	0	0	0
0	0	0	0	0	2	3	0	0	0	0
0	4	1	0	0	16	4	1	0	0	0
0	3	1	0	5	16	3	0	0	0	0
0	1	1	0	0	6	7	1	0	0	0
0	3	0	0	0	4	3	0	0	0	0
0	7	1	0	0	1	1	0	0	0	1
0	0	0	0	0	0	2	0	0	0	0
0	3	1	0	0	0	9	0	0	0	0
1	6	0	0	0	0	6	1	0	0	1
0	7	2	0	0	31	9	0	0	0	0
0	0	0	0	4	4	2	0	0	0	0
0	0	2	1	0	2	1	0	0	0	0
0	0	6	0	10	30	4	0	0	0	0
0	0	1	0	8	18	0	0	0	0	0
0	0	0	0	3	10	4	0	0	0	0
0	0	0	0	0	2	2	0	0	0	0
1	0	0	0	1	7	3	0	0	0	0
0	0	2	0	1	7	1	0	0	0	0
0	0	0	0	0	0	4	0	0	0	0
0	0	1	0	0	1	1	0	0	0	0
0	1	4	0	0	6	4	0	0	0	0
0	0	3	0	0	0	2	0	0	0	0
0	0	3	0	5	2	1	0	0	0	0
0	0	1	0	10	3	4	0	0	0	0
0	0	0	0	0	0	1	0	0	0	0
0	0	2	0	0	6	6	0	0	0	0
0	0	0	0	0	2	1	0	0	0	0

0	0	0	0	0	0	1	0	0	0	0
0	0	0	0	0	1	3	0	0	0	0
0	0	1	0	0	1	3	0	0	0	0
0	0	0	0	0	1	2	0	0	0	0
0	0	1	0	0	2	2	0	0	0	0
1	3	1	0	0	3	3	0	0	0	0
0	1	3	0	0	0	3	0	0	0	0
0	0	0	0	0	0	2	0	0	0	0
0	0	1	0	0	1	2	0	0	0	0
1	0	0	0	0	2	1	0	0	0	0
1	0	1	0	0	7	1	0	0	0	0
1	0	0	0	0	0	0	0	0	0	0
0	0	0	0	0	3	1	0	0	0	0
0	0	0	0	0	0	1	0	0	0	0
0	0	0	0	0	0	3	0	0	0	0
0	0	0	0	0	3	0	0	0	0	0
0	0	0	0	0	1	0	0	0	0	0
0	1	0	0	0	0	0	0	0	0	0
0	0	0	0	0	0	0	0	0	0	0
0	0	0	0	0	0	0	0	0	0	0
0	0	1	0	0	1	0	0	0	0	0
0	0	0	0	0	0	0	0	0	0	0
0	0	2	0	1	5	2	0	0	0	0
0	0	3	0	0	4	2	0	0	0	0
0	1	0	0	0	3	2	0	0	0	0
2	0	1	0	0	4	0	0	0	0	0
0	0	1	0	0	3	2	0	0	0	0
0	0	0	0	0	4	2	0	0	0	0
0	0	1	0	7	0	4	0	0	0	0

0	2	1	0	4	1	2	0	0	0	0
0	0	0	0	4	0	3	0	0	0	0
0	0	0	0	6	4	3	0	0	0	0
1	0	0	0	2	2	7	0	0	0	0
0	2	0	0	0	3	3	0	0	0	0
0	0	0	0	1	5	6	0	0	0	0
0	2	1	0	0	6	4	0	0	0	0
0	1	5	0	0	4	2	0	0	0	0
0	2	0	0	7	8	4	0	0	0	0
0	0	1	0	0	5	6	0	0	1	0
1	1	0	0	0	8	3	0	0	0	0
0	1	0	0	0	2	4	0	0	0	0
0	1	0	0	2	2	5	0	0	0	0
0	1	0	0	3	5	3	0	0	0	0
0	1	0	0	2	2	4	0	0	0	0
0	0	0	0	0	12	5	0	0	0	0
0	0	0	0	1	1	6	0	0	0	0
0	1	1	0	1	2	6	0	0	0	0
0	1	1	0	0	3	6	0	0	0	0
0	0	1	0	0	1	4	0	0	0	0
0	0	0	0	0	3	4	0	0	0	0
0	1	1	0	0	2	6	0	0	0	0
0	1	1	0	0	7	5	0	0	0	0
0	3	1	0	1	0	10	0	1	0	0
0	0	0	0	0	0	3	0	0	0	0
0	3	0	0	0	2	7	1	0	0	0
0	2	1	0	0	5	6	0	0	0	0
0	4	0	0	2	7	5	0	0	0	0
0	0	1	0	0	4	6	0	0	0	0

0	0	0	0	1	3	4	2	0	0	0
0	1	0	0	1	9	7	0	0	0	0
0	2	0	0	0	7	6	0	0	0	0
0	1	3	0	0	8	4	1	0	0	0
0	2	1	0	0	2	1	0	0	0	0
0	0	0	0	0	0	4	4	1	0	0
0	0	1	0	0	1	6	0	0	0	0
0	3	0	0	0	1	7	2	3	0	0
0	2	0	0	0	3	6	1	0	0	0
0	3	0	0	0	3	4	0	0	0	0
0	8	1	0	0	4	9	0	0	0	0
0	8	4	0	0	2	5	0	0	0	0
0	5	0	0	0	0	2	0	0	0	0
0	2	1	0	0	2	2	0	0	0	0
0	1	2	0	0	0	3	2	0	0	0
0	1	1	0	0	1	3	1	0	0	0
0	1	0	0	0	3	6	0	0	0	0
0	2	1	0	0	0	0	1	0	0	0
0	3	2	0	0	0	1	0	0	0	0
0	3	0	0	0	0	3	0	0	0	0
0	2	0	0	0	6	2	0	0	0	0
0	1	1	0	0	5	8	2	0	0	0
0	1	2	0	0	8	10	0	0	0	0
0	1	0	0	0	2	4	0	0	0	0
0	0	0	0	0	6	7	1	0	0	0
0	2	0	0	0	6	5	0	0	0	0
0	0	1	0	0	14	4	0	0	0	0
0	1	0	0	0	25	6	0	0	0	0
0	0	0	0	0	4	4	0	0	0	0

0	0	0	0	0	2	5	0	0	0	0
0	0	0	0	0	9	4	0	0	0	0
0	2	0	0	0	0	3	0	0	0	0
0	1	1	0	0	13	0	0	0	0	0
0	0	0	0	2	2	4	0	0	0	0
0	0	0	0	2	2	6	0	0	0	0
0	1	0	0	0	2	5	0	0	0	0
0	0	0	0	5	2	3	0	0	0	0
0	5	2	0	0	2	3	0	0	0	0
0	1	0	0	1	4	6	0	0	0	0
0	2	1	0	3	2	2	0	0	0	0
0	0	1	0	2	1	4	0	0	0	0
0	0	2	0	1	8	6	0	0	0	0
0	1	0	0	1	1	4	0	0	0	0
0	1	5	0	9	3	2	0	0	0	0
0	1	2	0	4	2	2	0	0	0	0
0	0	0	0	3	0	6	0	0	0	0
0	0	2	0	2	0	1	0	0	0	0
0	0	1	0	5	3	4	0	0	0	0
0	0	2	0	4	0	4	0	0	0	0
0	0	1	0	3	0	3	0	0	0	0
0	0	1	0	2	2	4	0	0	0	0
0	2	1	0	2	8	3	0	0	0	0
0	0	4	0	6	2	3	0	0	0	0
0	0	0	0	5	0	0	0	0	0	0
0	0	0	0	4	0	4	0	0	0	0
0	1	0	0	0	1	2	0	0	0	0
0	0	0	0	4	1	3	0	0	0	0
0	0	3	0	5	3	1	0	0	0	0

0	1	11	0	1	2	3	0	0	0	0
0	1	2	0	1	3	2	0	0	0	0
0	0	2	0	4	1	1	0	0	0	0
0	0	3	0	1	3	2	0	0	0	0
0	0	3	0	0	0	3	0	0	0	0
0	3	2	0	3	0	3	0	0	0	0
0	0	0	0	2	1	3	0	0	0	0
0	0	0	0	8	7	4	0	0	0	0
0	0	3	0	0	2	4	0	0	0	0
0	0	1	0	4	1	4	0	0	0	0
0	0	0	0	6	0	2	0	0	0	0
0	0	0	0	8	0	3	0	0	0	0
1	0	1	0	2	1	2	0	0	0	0
0	0	0	0	5	0	3	0	0	0	0
0	0	0	0	0	3	4	0	0	0	0
0	0	0	0	0	5	4	0	0	0	0
0	4	1	0	0	1	3	0	0	1	0
0	1	0	0	1	4	5	0	0	0	0
0	0	3	0	2	1	2	0	0	0	0
0	2	0	0	2	2	4	0	0	0	0
0	1	0	0	2	1	4	0	0	0	0
0	1	2	0	0	5	4	0	0	0	0
0	1	0	0	0	0	5	0	0	0	0
0	0	1	0	0	0	5	0	0	0	0
0	0	1	0	3	6	3	0	0	0	0
0	0	0	0	2	14	5	0	0	0	0
0	0	2	0	0	1	5	0	0	0	0
0	0	1	0	1	2	1	0	0	0	0
0	0	1	0	2	6	2	0	0	0	0

0	1	3	0	5	4	3	0	0	0	0
0	0	5	0	2	18	4	0	0	0	0
0	0	9	0	0	5	2	0	0	0	0
0	0	7	0	0	0	1	0	0	0	0
0	0	7	0	0	7	1	0	0	0	0
0	1	2	0	1	6	5	0	0	0	0
0	0	0	0	7	6	0	0	0	0	0
0	0	7	0	5	6	0	0	0	0	0
0	0	4	0	1	17	1	0	0	0	0
0	0	1	0	12	3	4	0	0	0	0
0	0	0	0	0	12	0	0	0	0	0
0	0	0	0	2	4	0	0	0	0	0
0	0	5	0	2	8	0	0	0	0	0
1	0	1	0	6	9	2	0	0	0	0
1	0	6	0	6	16	2	0	0	0	0
0	0	0	0	1	4	2	0	0	0	0
0	0	0	0	9	1	7	0	0	0	0
0	2	1	0	11	6	4	0	0	0	0
0	1	0	0	0	1	4	0	0	0	0
0	0	0	0	0	4	4	0	0	0	0
0	3	3	0	0	8	7	0	0	0	0
0	2	2	0	0	5	5	0	0	0	0
0	2	1	0	0	2	4	0	0	0	0
1	1	0	0	7	0	3	0	0	0	0
0	1	0	0	4	5	4	0	0	0	0
0	2	2	0	3	1	6	0	0	0	0
0	0	0	0	1	4	2	0	0	0	0
1	0	0	0	0	1	1	0	0	0	0
0	0	0	0	0	2	3	0	0	0	0

1	2	2	0	1	9	6	0	0	0	0
0	0	2	0	1	6	3	0	0	0	0
0	0	0	0	0	1	6	0	0	0	0
0	1	0	0	0	6	2	0	0	0	0
0	0	0	0	0	0	5	0	0	0	0
0	0	0	0	0	8	1	0	0	0	0
0	0	1	0	0	3	2	0	0	0	0
0	0	8	0	0	4	5	0	0	0	0
0	0	12	0	1	4	4	0	0	0	0
0	2	0	0	1	4	7	0	0	0	0
0	1	1	0	0	3	4	0	0	0	0
0	0	2	0	0	4	6	0	0	0	0
0	4	1	0	2	4	2	0	0	0	0
0	1	0	0	0	4	5	0	0	0	0
0	0	0	0	0	0	4	0	0	0	0
1	3	0	0	0	0	7	0	0	0	0
0	1	0	0	0	0	3	0	0	0	0
0	0	1	0	1	1	8	0	0	0	0
0	0	1	0	0	0	4	0	0	0	0
0	1	0	0	0	2	4	0	0	0	0
0	0	5	0	0	0	3	0	0	0	0
0	0	0	0	0	0	7	0	0	0	0
0	3	0	0	0	8	5	0	0	0	0
0	0	1	0	3	4	6	0	0	0	0
0	0	3	0	0	5	7	0	0	0	0
0	0	3	0	0	7	3	0	0	0	0
0	1	1	0	0	7	2	0	0	0	0
0	0	2	0	2	6	0	0	0	0	0
0	0	2	0	3	0	2	0	0	0	0

0	1	2	0	5	2	3	0	0	0	0
0	0	4	0	0	5	7	0	0	0	0
0	0	0	0	0	0	5	0	0	0	0
0	0	4	0	10	14	6	0	0	0	0
0	0	5	0	1	0	3	0	0	0	0
0	0	5	0	1	6	1	0	0	0	0
0	0	4	0	2	1	2	0	0	0	0
0	0	2	0	1	3	6	0	0	0	0
0	0	2	0	1	5	3	0	0	0	0
0	0	5	0	4	5	2	0	0	0	0
0	0	1	0	5	6	3	0	0	0	0
0	0	4	0	5	4	2	0	0	0	0
0	0	3	0	3	2	1	0	0	0	0
0	0	1	0	6	2	1	0	0	0	0
0	0	4	0	6	0	4	0	0	0	0
0	0	2	0	6	2	1	0	0	0	0
0	1	3	0	9	0	3	0	0	0	0
0	2	2	0	4	3	2	0	0	0	0
0	0	0	0	3	1	1	0	0	0	0
0	0	6	0	7	1	3	0	0	0	0
0	1	2	0	5	0	4	0	0	0	0
0	0	3	0	4	1	4	0	0	0	0
0	0	1	0	0	0	3	0	0	0	0
0	0	3	0	3	4	3	0	0	0	0
0	0	1	0	2	13	4	0	0	0	0
0	0	1	0	2	5	2	0	0	0	0
0	0	0	0	3	3	6	0	0	0	0
0	0	0	0	4	1	5	0	0	0	0
0	1	0	0	11	2	4	0	0	0	0

0	1	3	0	9	4	5	0	0	0	0
0	1	2	0	4	2	7	0	0	0	0
0	0	1	0	1	7	6	0	0	0	0
0	0	2	0	0	10	7	0	0	0	0
1	1	1	0	5	1	7	0	0	0	0
0	2	2	0	4	0	6	0	0	0	0
0	0	0	0	1	4	5	0	0	0	0
0	0	1	0	1	10	6	0	0	0	0
0	2	0	0	2	12	4	0	0	0	0
0	2	2	0	4	22	7	0	0	0	0
0	0	0	0	0	10	4	0	0	0	0
0	3	3	0	0	8	3	0	0	0	0
0	1	1	0	0	2	4	0	0	0	0
0	3	2	0	2	2	6	0	0	0	0
0	3	0	0	0	0	1	0	0	0	0
0	0	1	0	0	8	7	0	0	0	0
0	0	1	0	0	3	4	2	0	0	0
0	3	0	0	0	1	5	0	0	0	0
0	3	0	0	0	2	4	0	0	0	0
0	1	2	0	0	0	5	0	0	0	0
0	0	0	0	0	0	4	1	0	0	0
0	3	0	0	0	1	1	1	0	0	0
0	3	0	0	0	0	10	0	1	1	0
0	1	0	0	0	0	5	2	0	0	0
0	3	0	0	0	0	4	1	0	0	0
0	0	0	0	0	0	7	2	0	0	0
0	6	0	0	0	0	8	0	0	0	0
1	3	0	0	0	3	6	2	0	0	0
0	6	1	0	0	0	4	1	0	0	0

0	1	0	0	0	0	7	6	0	0	0
0	1	0	0	0	0	8	3	2	0	0
0	3	0	0	0	0	2	1	0	0	0
0	5	2	0	0	5	1	1	0	0	0
0	1	0	0	0	4	7	1	0	0	0
0	5	0	0	0	0	6	0	0	0	1
0	1	0	0	0	5	2	2	0	0	0
0	4	0	0	0	15	8	0	0	0	0
0	6	0	0	0	6	7	1	0	0	0
0	8	1	0	0	12	0	0	0	0	0
0	1	1	0	0	4	3	1	0	0	0
0	2	0	0	0	2	3	1	0	0	0
0	0	0	0	0	5	5	1	0	0	0
0	2	3	0	6	33	2	2	1	0	0
0	2	0	0	0	6	4	2	0	0	0
0	6	0	0	0	8	3	1	0	0	0
0	0	1	0	0	6	2	0	0	0	0
0	0	3	0	0	18	1	0	0	0	0
0	0	1	0	0	8	5	0	0	0	0
0	0	3	0	0	0	4	0	0	0	0
0	5	1	0	0	10	3	1	0	0	0
0	2	3	0	4	7	4	0	1	0	0
0	0	1	0	2	28	2	1	0	0	0
0	0	1	0	6	11	6	0	0	0	0
0	2	2	0	8	14	6	0	0	0	0
0	5	1	0	6	22	4	0	0	0	0
0	0	2	0	2	8	6	0	0	0	0
0	4	0	0	1	13	7	0	0	0	0
0	4	0	0	3	9	4	0	0	0	0

0	2	0	0	5	5	4	0	0	0	0
0	2	0	0	0	8	3	0	0	0	0
0	1	2	0	0	9	3	0	0	0	0
0	0	1	0	6	12	8	0	0	0	0
0	1	2	0	7	5	1	0	0	0	0
0	0	2	0	8	10	3	0	0	0	0
1	2	1	0	4	13	1	0	0	0	0
0	1	3	0	4	9	8	0	0	0	0
0	1	0	0	4	12	5	1	0	0	0
0	0	2	0	2	6	3	0	0	0	0
0	0	1	0	2	12	5	0	0	0	0
0	0	1	0	9	2	4	0	0	0	0
0	0	2	0	4	18	4	0	0	0	0
0	0	0	0	7	3	3	0	0	0	0
0	1	0	0	4	4	3	0	0	0	0
0	0	0	0	4	1	1	0	0	0	0
0	0	3	0	8	9	4	0	0	0	0
0	0	1	0	3	1	2	1	0	0	0
0	0	0	0	4	14	7	0	0	0	0
0	1	0	0	1	0	6	0	0	0	0
0	2	0	0	7	7	4	0	0	0	0
0	1	3	0	5	0	4	0	0	0	0
0	1	0	0	1	1	5	0	0	0	0
0	1	0	0	1	6	4	0	0	0	0
0	0	0	0	2	10	4	0	0	0	0
0	0	0	0	1	0	6	0	0	0	0
0	1	0	0	0	0	4	0	0	0	0
0	0	4	0	0	18	4	0	0	0	0
0	0	2	0	0	8	5	0	0	0	0

0	0	7	0	1	13	8	0	0	0	0
0	0	2	0	0	6	7	0	0	0	0
0	0	0	0	0	8	6	0	0	0	0
0	0	1	0	1	0	2	0	0	0	0
1	2	0	0	0	0	3	1	0	0	0
0	1	6	0	4	5	4	0	0	0	0
0	1	0	0	2	14	8	0	0	0	0
0	1	0	0	0	1	3	0	0	0	0
0	1	0	0	0	1	3	0	0	0	0
0	6	0	0	0	1	4	0	0	0	0
0	0	2	0	0	6	3	0	0	0	0
0	5	1	0	0	0	3	0	0	0	0
0	2	0	0	0	2	4	0	0	0	0
0	2	0	0	0	5	2	1	0	0	0
0	2	2	0	0	0	2	0	0	0	0
0	4	0	0	0	0	2	0	0	0	0
0	2	1	0	0	0	3	0	0	0	0
0	8	1	0	0	2	3	0	1	0	1
0	3	0	0	0	9	5	0	0	0	0
0	3	4	0	0	2	5	0	0	0	0
0	3	1	0	0	0	4	0	0	0	0
0	2	2	0	0	9	6	1	1	0	0
0	5	1	0	0	1	4	1	0	0	0
0	5	0	0	0	2	1	0	0	0	0
0	0	0	0	0	1	0	0	0	0	0
0	5	0	0	0	1	4	0	0	0	0
0	10	0	0	0	2	2	0	0	0	0
0	4	0	0	1	0	3	0	0	0	0
0	4	0	0	1	0	5	0	0	0	0

1	7	6	0	0	0	4	0	0	0	0
0	0	2	0	0	0	0	0	0	0	0
0	1	0	0	0	0	4	0	0	0	0
0	0	0	0	0	3	0	0	0	0	0
0	0	1	0	0	5	0	0	0	0	0
0	0	2	0	0	0	3	0	0	0	0
0	0	15	0	0	7	4	0	0	0	0
0	0	3	0	0	2	2	0	0	0	0
0	3	0	0	0	2	0	0	0	0	0
0	0	0	0	0	3	3	0	0	0	0
0	0	0	0	0	1	5	0	0	0	0
0	1	5	0	0	1	2	0	0	0	0
1	0	1	0	0	0	3	0	0	0	0
0	0	0	0	0	0	1	0	0	0	0

felzel	gammar	glycer	glygra	glytri	halcoo	halwhi	hamzel	hemedw	hemhir	hesion
0	0	0	0	0	0	0	0	0	0	0
0	0	0	0	0	0	0	0	0	0	0
0	0	0	0	0	0	0	0	0	0	0
0	0	0	0	0	0	0	0	0	0	0
0	0	0	0	0	0	0	0	0	0	0
0	0	0	0	0	0	0	0	0	0	1
0	0	0	0	0	0	0	0	0	0	0
0	0	0	0	0	0	0	0	0	0	0
0	0	0	0	0	0	0	0	0	0	0
0	0	0	0	0	0	0	0	0	0	0
0	0	0	0	0	0	0	0	0	0	1
0	0	0	0	0	0	0	0	0	0	1
0	0	0	0	0	0	0	0	0	0	0
0	0	0	0	0	0	0	0	0	1	0
0	0	0	0	0	0	0	0	0	0	0
0	0	0	0	0	0	0	0	0	0	2
0	0	0	0	0	0	0	0	0	0	3
0	0	0	0	0	0	0	0	0	0	0
0	0	0	0	0	0	0	0	0	0	1
0	0	0	0	0	0	0	0	0	0	3
0	0	0	0	0	0	0	0	0	0	1
0	0	0	0	0	0	0	0	0	0	1
0	0	0	0	0	0	0	0	0	0	0
0	0	0	0	0	0	2	0	0	0	0
0	0	0	0	0	0	0	0	0	0	0
0	0	0	0	0	0	0	0	0	0	1
0	0	0	0	0	0	0	0	0	0	0
0	0	0	0	0	0	0	0	0	0	1

0	0	0	0	0	0	0	0	0	0	0
0	0	1	0	0	0	0	0	0	0	0
0	0	0	0	0	0	0	0	0	0	1
0	0	0	0	0	0	0	0	0	0	0
0	0	0	0	0	0	0	0	0	0	0
0	0	0	0	0	0	0	0	0	0	0
0	0	0	0	0	0	0	0	0	0	0
0	0	0	0	0	0	0	0	0	0	0
0	0	0	0	0	0	2	0	0	0	1
0	0	0	0	0	1	0	0	0	0	0
0	0	0	0	0	0	0	0	0	0	0
0	0	0	0	0	0	0	0	0	0	0
0	0	0	0	0	0	0	0	0	0	0
0	0	0	0	0	0	0	0	0	0	0
0	0	0	0	0	0	0	0	0	0	0
0	0	0	0	0	0	1	0	0	0	0
0	0	0	0	0	2	0	0	0	3	0
0	0	0	0	0	0	0	0	0	0	0
0	0	0	0	0	0	2	1	0	1	0
0	0	0	0	0	1	0	0	0	1	0
0	0	0	0	0	0	0	0	0	0	1
0	0	0	0	0	0	1	0	0	0	0
0	0	0	0	0	0	1	0	0	0	0
0	0	0	0	0	0	0	0	0	0	0
0	0	0	0	0	0	0	0	0	0	0
0	0	0	0	0	0	0	0	0	0	0
0	0	0	0	0	0	0	0	0	0	1
0	0	0	0	0	0	1	0	0	0	0
0	0	0	0	0	0	1	0	0	0	0

0	0	0	0	0	0	0	0	0	0	5
1	0	0	0	0	0	0	0	0	0	2
0	0	0	0	0	0	0	0	0	0	0
0	0	0	0	0	0	0	0	0	0	1
0	0	0	0	0	0	0	0	0	0	0
0	0	0	0	0	0	0	0	0	0	0
0	0	0	0	0	0	0	0	0	0	0
0	0	0	0	0	0	1	0	0	0	0
0	0	0	0	0	0	0	0	0	0	0
0	0	0	0	0	0	0	0	0	0	0
0	0	0	0	0	0	0	0	0	0	1
0	0	0	0	0	0	0	0	0	0	0
0	0	0	0	0	0	0	0	0	0	1
0	0	0	0	0	0	0	0	0	0	0
0	0	0	0	0	0	0	0	0	0	0
0	0	0	0	0	0	0	0	0	0	0
0	0	0	0	0	0	0	0	0	0	0
0	0	0	0	0	0	0	0	0	0	0
0	0	0	0	0	0	0	0	0	0	0
0	0	1	0	0	0	1	0	0	1	5
0	0	0	0	0	0	0	0	0	0	1
0	0	0	0	0	0	1	0	0	0	0
0	0	0	0	0	0	0	0	0	0	0
0	0	0	0	0	0	0	0	0	0	0
0	0	0	0	0	0	0	0	0	0	0
0	0	0	0	0	0	0	0	0	0	0
0	0	0	0	0	0	0	0	0	0	1
0	0	0	0	0	0	0	0	0	0	0
0	0	0	0	0	1	0	0	0	0	0

0	0	0	0	0	3	0	0	0	2	0
0	0	0	0	0	0	1	0	0	0	0
0	0	0	0	0	3	5	0	0	1	0
0	0	0	0	0	2	0	0	0	1	0
0	0	0	0	0	0	0	0	0	0	0
0	0	0	0	0	0	0	0	0	0	0
0	0	0	0	0	0	0	0	0	0	0
0	0	0	0	0	0	0	0	0	1	0
0	0	0	0	0	0	0	0	0	0	1
0	0	0	0	0	0	0	0	0	0	2
0	0	0	0	0	0	1	0	0	0	0
0	0	0	0	0	0	0	0	0	0	2
0	0	0	0	0	0	0	0	0	0	0
0	0	0	0	0	0	0	0	0	0	0
0	0	0	0	0	0	0	0	0	1	0
0	0	0	0	0	0	0	0	0	0	0
0	0	0	0	0	0	0	0	0	0	0
0	0	0	0	0	0	0	0	0	0	0
0	0	0	0	0	0	0	0	0	0	0
0	0	0	0	0	1	1	0	0	0	0
0	0	0	0	0	0	0	0	0	0	1
0	0	0	0	0	0	0	0	0	0	0
0	0	0	0	0	2	0	0	0	0	0
0	0	0	0	0	0	0	0	0	1	0
0	0	0	0	0	0	5	0	0	2	3
0	0	0	0	0	0	1	0	0	0	0
0	0	0	0	0	0	0	0	0	0	1
0	0	0	0	0	1	0	0	0	0	0
0	0	0	0	0	3	1	0	0	0	0

0	0	0	0	0	0	0	0	0	0	0
0	0	0	0	0	0	0	0	0	0	0
0	0	0	0	0	0	0	0	0	0	1
0	0	0	0	0	0	1	0	0	0	0
0	0	0	0	0	0	0	0	0	0	0
0	0	0	0	0	0	0	0	0	2	0
0	0	0	0	0	0	0	0	0	0	0
0	0	0	0	0	0	1	0	0	0	0
0	0	0	0	0	0	0	0	0	0	1
0	0	0	0	0	0	0	0	0	0	0
0	0	0	0	0	0	0	0	0	0	0
0	0	0	0	0	0	1	0	0	0	0
0	0	1	0	0	1	0	0	0	1	0
0	0	0	0	0	2	0	0	0	0	0
0	0	0	0	0	1	0	0	0	0	0
0	0	0	0	0	0	0	0	0	2	0
0	1	0	0	0	2	0	0	0	2	0
0	0	1	0	0	0	1	0	1	0	0
0	0	0	0	0	0	0	0	0	0	0
0	0	0	0	0	0	0	0	0	1	0
0	0	1	0	0	0	1	0	0	0	0
0	0	0	0	0	0	0	0	0	0	0
0	0	0	0	0	0	0	0	0	0	0
0	0	0	0	0	0	0	0	0	0	0
0	0	0	0	0	0	0	0	0	1	1
0	0	0	0	0	0	0	0	0	0	0
0	0	0	0	0	0	0	0	0	0	0
0	0	0	0	0	0	2	0	0	0	0
0	0	0	0	0	0	0	0	0	0	0

0	0	1	0	0	0	0	0	0	0	0
0	0	0	0	0	0	1	0	0	1	0
0	0	0	0	0	0	0	0	0	0	0
0	0	1	0	0	0	0	0	0	0	0
0	0	0	0	0	0	0	0	0	0	2
0	0	0	0	0	0	0	0	0	0	0
0	0	0	0	0	0	0	0	0	0	5
0	0	0	0	0	0	0	0	0	0	1
0	0	1	0	0	0	0	0	0	0	0
0	0	0	0	0	0	0	0	0	0	1
0	0	0	0	0	0	1	0	0	0	2
0	0	0	0	0	0	0	0	0	0	0
0	0	0	0	0	0	1	1	0	0	0
0	0	0	0	0	0	0	0	0	0	0
0	0	0	0	0	0	0	0	0	0	0
0	0	0	0	0	0	0	0	0	0	0
0	0	0	0	0	0	0	0	0	0	0
0	0	0	0	0	0	0	0	0	0	0
0	0	0	0	0	0	0	0	0	0	0
0	0	0	0	0	0	0	0	0	0	0
0	0	0	0	0	0	0	0	0	2	0
0	0	0	0	0	0	0	0	0	0	0
0	0	0	0	0	0	0	0	0	0	0
0	0	1	0	0	1	0	0	0	1	0
0	0	0	0	0	0	0	0	0	0	0
0	0	0	0	0	0	0	1	0	0	0
0	0	0	0	0	0	0	1	0	0	0
0	0	0	0	0	0	0	0	0	0	0
0	0	0	0	0	0	0	0	0	0	0

0	0	0	0	0	0	0	0	0	0	0
0	0	0	0	0	0	0	0	0	0	0
0	0	0	0	0	0	0	0	0	0	0
0	0	0	0	0	0	0	0	0	0	0
0	0	0	0	0	0	0	0	0	0	0
0	0	0	0	0	0	0	0	0	0	0
0	0	0	0	0	0	0	0	0	0	0
0	0	0	0	0	0	0	0	0	0	0
0	0	0	0	0	0	0	0	0	0	0
0	0	0	0	0	0	0	0	0	0	0
0	0	0	0	0	0	0	0	0	0	0
0	0	0	0	0	0	0	0	0	0	0
0	0	0	0	0	0	0	0	0	0	0
0	0	0	0	0	0	0	0	0	0	0
0	0	0	0	0	0	0	0	0	0	0
0	0	0	0	0	0	0	0	0	0	0
0	0	0	0	0	0	0	0	0	0	0
0	0	0	0	0	0	1	0	0	0	0
0	0	0	0	0	0	0	0	0	0	0
0	0	0	0	0	0	0	0	0	0	1
0	0	0	0	0	0	0	0	0	0	0
0	0	0	0	0	0	1	0	0	1	0
0	0	0	0	0	0	0	0	0	0	0
0	0	0	0	0	0	0	0	0	0	0
0	0	0	0	0	0	0	0	0	0	0
0	0	0	0	0	0	0	0	0	0	0
0	0	0	0	0	0	0	0	0	0	0
0	0	0	0	0	0	1	0	0	0	0
0	0	0	0	0	0	1	0	0	0	0

0	0	0	0	0	0	0	0	0	0	0
0	0	0	0	0	0	0	0	0	0	0
0	0	0	0	0	0	0	0	0	0	0
0	0	0	0	0	0	0	0	0	0	0
0	0	0	0	0	0	0	0	0	0	0
0	0	0	0	0	0	0	0	0	0	0
0	0	0	0	0	0	0	0	0	0	0
0	0	0	0	0	0	0	0	0	0	0
0	0	0	0	0	0	0	0	0	0	0
0	0	0	0	0	0	0	0	0	0	0
0	0	0	0	0	0	0	0	0	0	0
0	0	0	0	0	0	0	0	0	0	0
0	0	0	0	0	0	0	0	0	0	0
0	0	0	0	0	0	0	0	0	0	0
0	0	0	0	0	0	0	0	0	0	0
0	0	0	0	0	0	1	0	0	0	0
0	0	0	0	0	0	0	0	0	0	0
0	0	0	0	0	0	0	0	0	0	2
0	0	0	0	0	0	0	0	0	0	0
0	0	0	0	0	0	0	0	0	0	1
0	0	0	0	0	0	0	0	0	0	1
0	0	0	0	0	0	0	0	0	0	0
0	0	0	0	0	0	0	0	0	0	0
0	0	1	0	0	0	0	0	0	0	0
0	0	0	0	0	0	0	0	0	0	2
0	0	0	0	0	0	0	0	0	0	0
0	0	0	0	0	0	0	0	0	0	0
0	0	0	0	0	0	1	0	0	0	0
0	0	0	0	0	0	0	0	0	0	2

0	0	0	0	0	0	0	0	0	0	2
0	0	0	0	0	0	1	0	0	0	0
0	0	0	0	0	0	0	0	0	0	0
0	0	0	0	0	0	0	0	0	0	1
0	0	0	0	0	0	0	0	0	0	0
0	0	0	0	0	0	0	0	0	0	0
0	0	0	0	0	0	1	0	0	0	0
0	0	0	0	0	0	0	0	0	0	1
0	0	0	0	0	0	1	0	0	0	2
0	0	0	0	0	0	0	0	0	0	1
0	0	0	0	0	0	0	0	0	0	7
0	0	0	0	0	0	0	0	0	0	0
0	0	0	0	0	0	0	0	0	0	1
0	0	0	0	0	0	0	0	0	0	0
0	0	0	0	0	0	0	0	0	0	0
0	0	1	0	0	0	0	0	0	1	0
0	0	0	0	0	0	1	0	0	0	2
0	0	0	0	0	0	1	0	0	0	0
0	0	0	0	0	0	0	0	0	0	1
0	0	0	0	0	0	0	0	0	0	0
0	0	0	0	0	0	1	0	0	0	0
0	0	0	0	0	0	0	0	0	0	1
0	0	0	0	0	0	0	0	0	0	0
0	0	0	0	0	0	0	0	0	0	1
0	0	1	0	0	0	1	0	0	0	0
0	0	0	0	0	0	0	0	0	0	0
0	0	0	0	0	0	0	0	0	0	2
0	0	0	0	0	0	0	0	0	1	0
0	0	0	0	0	2	0	0	0	1	0

0	0	0	0	0	0	3	0	0	0	1
0	0	0	0	0	0	0	0	0	0	0
0	0	0	0	0	2	0	0	0	0	3
0	0	0	0	0	0	0	0	0	0	3
0	0	0	0	0	0	0	0	0	0	0
0	0	0	0	0	0	0	0	0	0	0
0	0	0	0	0	0	0	0	0	1	0
0	0	0	0	0	0	0	0	0	0	0
0	0	1	0	0	0	1	0	0	0	0
0	0	0	0	0	0	0	0	0	0	0
0	0	0	0	0	0	0	0	0	0	0
0	0	0	0	0	0	0	0	0	0	0
0	0	1	0	0	0	0	0	0	0	0
0	0	0	0	0	0	0	0	0	0	0
0	0	0	0	0	0	0	0	0	0	2
0	0	0	0	0	0	3	0	0	0	0
0	0	0	0	0	0	0	0	0	0	1
0	0	0	0	0	0	0	0	0	1	0
0	0	0	0	0	0	0	0	0	0	0
0	0	0	0	0	0	0	0	0	0	1
0	0	0	0	0	0	0	0	0	0	1
0	0	1	0	0	0	0	0	0	0	0
0	0	0	0	0	0	0	0	0	0	0
0	0	0	0	0	0	0	0	0	0	2
0	0	0	0	0	0	0	0	0	0	0
0	0	0	0	0	0	2	1	0	0	2
0	0	0	0	0	0	0	0	0	0	0
0	0	0	0	0	0	0	0	0	0	3
0	0	0	0	0	0	0	0	0	0	2

0	0	0	0	0	0	0	0	0	0	4
0	0	0	0	0	0	3	0	0	0	3
0	0	0	0	0	0	0	0	0	0	0
1	0	0	0	0	0	2	0	0	0	2
0	0	0	0	0	0	0	0	0	0	3
0	0	0	0	0	0	0	0	0	0	5
0	0	0	0	0	0	0	0	0	1	0
0	0	0	0	0	0	0	0	0	0	0
0	0	0	0	0	0	0	0	0	0	0
0	0	0	0	0	0	0	0	0	0	2
0	0	0	0	0	0	0	0	0	0	1
0	0	0	0	1	0	0	0	0	0	1
0	0	0	0	0	0	0	0	0	0	1
0	0	0	0	0	0	3	0	0	1	0
0	0	0	0	0	0	1	0	0	0	1
0	0	0	0	0	0	0	0	0	0	1
0	0	0	0	0	0	0	0	0	0	0
0	0	0	0	0	0	0	0	0	0	0
0	0	0	0	0	0	0	0	0	0	0
0	0	0	0	0	0	0	0	0	0	0
0	0	0	0	0	0	0	0	0	0	0
0	0	0	0	0	0	0	0	0	0	0
0	0	0	0	0	0	0	0	0	0	6
0	0	0	0	0	0	0	0	0	0	1
0	0	0	0	0	0	0	0	0	0	2
0	0	0	0	0	0	0	0	0	0	1
0	0	0	1	0	0	0	0	0	0	2
0	0	0	0	0	0	0	0	0	0	0
0	0	0	0	0	0	0	0	0	0	1

0	0	1	0	0	0	0	0	0	0	6
0	0	0	0	0	0	0	0	0	0	6
0	0	0	0	0	0	0	0	0	0	5
0	0	0	0	0	0	1	0	0	0	0
0	0	0	0	0	0	0	0	0	0	1
0	0	0	0	0	0	0	0	0	0	0
0	0	0	0	0	0	0	0	0	0	0
0	0	0	0	0	0	0	0	0	0	4
0	0	0	0	0	0	0	0	0	0	3
0	0	0	0	0	0	0	0	0	0	1
0	0	0	0	0	0	0	0	0	0	0
0	0	0	0	0	0	0	0	0	0	7
0	0	0	0	0	0	0	0	0	0	0
0	0	0	0	0	0	0	0	0	0	1
0	0	0	0	0	0	0	0	0	0	3
0	0	0	0	0	0	0	0	0	0	4
0	0	0	0	0	1	0	0	0	0	0
0	0	1	0	0	0	4	0	0	1	0
0	0	0	0	0	2	0	0	0	1	0
0	0	0	0	0	0	1	1	0	0	0
0	0	0	0	0	0	0	0	0	0	1
0	0	0	0	0	0	0	0	0	0	2
0	0	0	0	0	0	0	0	0	0	0
0	0	0	0	0	0	0	0	0	0	0
0	0	0	0	0	0	0	0	0	0	2
0	0	0	0	0	0	0	0	0	0	0
0	0	1	0	0	0	0	0	0	0	0
0	0	0	0	0	0	0	0	0	0	0
0	0	0	0	0	0	0	0	0	0	0

0	0	0	0	0	0	1	0	0	0	0
0	0	0	0	0	0	0	0	0	0	0
0	0	0	0	0	0	0	0	0	0	0
0	0	0	0	0	0	0	0	0	1	0
0	0	0	0	0	0	0	0	0	0	0
0	0	0	0	0	0	0	0	0	0	0
0	0	0	0	0	0	1	0	0	0	0
0	0	0	0	0	0	1	0	0	0	0
0	0	0	0	0	0	2	0	0	0	1
0	0	0	0	0	0	0	0	0	0	0
0	0	0	0	0	0	0	0	0	0	0
0	0	0	0	0	0	0	0	0	0	0
0	0	0	0	0	0	0	0	0	0	0
0	0	0	0	0	0	0	0	0	0	1
0	0	0	0	0	0	2	0	0	0	1
0	0	0	0	0	0	0	0	0	0	0
0	0	0	0	0	0	0	0	0	0	0
0	0	0	0	0	0	0	0	0	0	0
0	0	0	0	0	0	0	0	0	0	0
0	0	0	0	0	0	1	0	0	0	1
0	0	0	0	0	0	0	0	0	0	0
0	0	0	0	0	0	0	0	0	0	1
0	0	0	0	0	0	0	0	0	0	1
0	0	0	0	0	0	0	0	0	0	2
0	0	0	0	0	0	0	0	0	0	4
0	0	0	0	0	0	0	0	0	0	1
0	0	0	0	0	0	0	0	0	0	1
0	0	0	0	0	0	1	0	0	0	0
0	0	0	0	0	0	0	0	0	0	3

0	0	0	0	0	0	0	0	0	0	1
0	0	0	0	0	0	0	0	0	0	0
0	0	0	0	0	0	0	0	0	0	0
0	0	0	0	0	0	0	0	0	0	0
0	0	0	0	0	0	0	0	0	0	0
0	0	0	0	0	0	0	0	0	0	2
0	0	0	0	0	0	0	0	0	0	0
0	0	0	0	0	0	0	0	0	0	0
0	0	0	0	0	0	0	0	0	0	0
0	0	0	0	0	0	0	0	0	0	0
0	0	0	0	0	0	0	0	0	0	0
0	0	0	0	0	0	0	0	0	0	1
0	0	0	0	0	0	0	0	0	0	1
0	0	0	0	0	0	0	0	0	0	0
0	0	0	0	0	0	0	0	0	0	0
0	0	0	0	0	0	0	0	0	0	2
0	0	0	0	0	0	0	0	0	0	0
0	0	0	0	0	0	0	0	0	0	0
0	0	0	0	0	0	0	0	0	0	1
0	0	0	0	0	0	1	0	0	1	1
0	0	0	0	0	0	0	0	0	0	0
0	0	0	0	0	0	0	0	0	0	0
0	0	0	0	0	0	0	0	0	0	0

glytri	halcoo	halwhi	hamzel	hemcre	hemhir	hetfil	isocla	laspar	lepido	levgra
0	0	0	0	0	0	0	0	0	0	0
0	0	0	0	0	0	2	0	0	0	0
0	0	0	0	0	0	0	0	0	0	0
0	0	0	0	0	0	1	0	0	0	0

0	0	0	0	0	0	0	0	0	0	0
0	0	0	0	0	0	0	0	0	0	0
0	0	0	0	0	0	2	0	0	0	0
0	0	0	0	0	0	0	0	0	0	0
0	0	1	0	0	0	0	0	0	0	0
0	0	0	0	0	0	0	0	0	0	0
0	0	0	0	0	0	14	0	0	0	0
0	0	2	0	0	1	8	0	0	0	0
0	0	1	0	0	0	26	0	0	0	0
0	0	0	0	0	0	28	0	0	0	0
0	0	3	0	0	0	28	0	0	0	0
0	0	1	0	0	0	20	0	0	0	0
0	0	0	0	0	1	30	0	0	0	0
0	0	0	0	0	0	28	0	0	0	0
0	0	1	0	0	0	9	0	0	0	0
0	0	2	0	0	0	13	0	0	0	0
0	0	0	0	0	0	0	0	0	0	0
0	0	0	0	0	0	1	0	0	0	0
0	0	0	0	0	0	0	0	0	0	0
0	0	0	0	0	0	1	0	0	0	0
0	0	0	0	0	0	0	0	0	0	0
0	0	0	0	0	0	0	0	0	0	0
0	0	1	0	0	0	0	0	0	0	0
0	0	1	0	0	0	0	0	0	0	0
0	0	0	0	0	0	0	0	0	0	0
0	0	1	0	0	0	1	0	0	0	0
0	0	0	0	0	0	0	0	0	0	0
0	0	0	0	0	0	1	0	0	0	0
0	0	0	0	0	0	0	0	0	0	0

0	0	0	0	0	0	1	0	0	0	0
0	0	1	0	0	0	0	0	0	0	0
0	0	1	0	0	0	0	0	0	0	0
0	0	1	0	0	0	0	0	0	0	0
0	0	1	0	0	0	0	0	0	0	0
0	0	0	0	0	0	0	0	0	0	0
0	0	0	0	0	0	1	0	0	0	0
0	0	0	0	0	0	3	0	1	0	0
0	0	0	0	0	1	5	0	0	0	0
0	0	0	0	0	0	2	0	0	0	0
0	0	0	1	0	0	3	0	0	0	0
0	0	1	0	0	0	3	0	0	0	0
0	0	0	0	0	0	3	0	0	0	0
0	0	0	0	0	0	4	0	0	0	0
0	0	0	0	0	0	5	0	0	0	0
0	0	0	0	0	1	1	0	0	0	0
0	0	0	0	0	0	2	0	0	0	0
0	0	0	0	0	0	2	0	0	0	0
0	0	0	0	0	1	6	0	0	0	0
0	0	0	0	0	0	2	0	0	0	0
0	0	0	0	0	0	2	0	0	0	0
0	0	0	0	0	0	6	0	0	0	0
0	0	0	0	0	0	5	0	0	0	0
0	0	0	0	0	0	6	0	0	0	0
0	0	1	0	0	1	4	0	0	0	0
0	0	1	0	0	0	5	0	0	0	0
0	0	0	0	0	1	0	0	0	0	0
0	0	2	0	0	0	3	0	0	0	0
0	0	0	0	0	0	23	0	0	0	0

0	0	2	1	0	0	4	0	0	0	0
0	0	0	0	0	0	2	0	0	0	0
0	0	0	0	0	0	2	0	0	0	2
0	0	0	0	0	1	4	0	0	0	0
0	0	1	0	0	0	5	0	0	0	0
0	0	1	0	0	0	5	0	0	0	0
0	0	0	0	0	0	12	0	0	0	0
0	0	1	0	0	1	15	0	0	0	0
0	0	0	0	0	2	11	0	0	0	0
0	0	0	0	0	0	27	0	0	0	0
0	0	1	2	0	0	28	0	0	0	0
0	0	0	1	0	0	27	0	0	0	0
0	0	0	0	0	1	24	0	0	0	0
0	0	0	1	0	1	29	0	0	0	0
0	0	0	1	0	1	30	0	0	0	0
0	0	0	0	0	0	25	0	0	0	0
0	0	2	0	0	0	12	0	0	0	0
0	0	0	0	0	0	15	0	0	0	0
0	0	1	0	0	0	24	0	0	0	0
0	0	2	0	0	2	15	0	0	0	0
0	0	0	0	0	0	15	0	0	0	0
0	0	0	0	0	1	19	0	0	0	0
0	0	0	0	0	1	16	0	0	0	0
0	0	0	1	0	0	2	0	0	0	0
0	0	0	0	0	0	8	0	0	0	0
1	0	1	0	0	0	14	0	0	1	0
0	0	0	0	0	0	5	0	0	0	0
0	0	0	0	0	0	20	0	0	0	0
0	0	1	0	0	0	17	0	0	0	0

0	0	0	0	0	0	13	0	0	0	0
0	0	0	0	0	0	8	0	0	0	0
0	0	1	0	0	0	17	0	0	0	0
0	0	0	0	0	0	5	0	0	0	0
0	0	1	0	0	1	29	0	0	0	0
0	0	0	0	0	1	11	0	0	0	0
0	0	0	0	0	0	14	0	0	0	0
0	0	0	0	0	0	16	0	0	0	0
0	0	0	0	0	0	23	0	0	0	0
0	0	0	0	0	0	14	0	0	0	0
0	0	0	0	0	0	8	0	0	0	0
0	0	0	0	0	0	9	0	0	0	0
0	0	0	0	0	0	13	0	0	0	0
0	0	0	0	0	0	4	0	0	0	0
0	0	2	0	0	0	3	0	0	0	0
0	0	2	0	0	1	11	0	0	0	0
0	0	1	0	0	0	10	0	0	0	0
0	0	3	0	0	0	6	0	0	0	0
0	0	4	0	0	0	6	0	0	0	0
0	0	1	0	0	0	19	0	0	0	0
0	0	0	0	0	1	12	0	0	0	0
0	0	4	0	0	0	8	0	0	0	0
0	0	1	0	0	1	15	0	0	0	0
0	0	1	0	0	0	11	0	0	1	0
0	0	0	0	0	0	17	0	0	0	0
0	0	2	0	0	0	10	0	0	0	0
0	0	3	0	0	0	13	0	0	0	0
0	0	0	0	0	1	18	0	0	0	0
0	0	1	0	0	0	11	0	0	0	0

0	0	3	0	0	0	15	0	0	0	0
0	0	4	0	0	0	8	0	0	0	0
0	0	0	0	0	0	4	0	0	0	0
0	0	1	0	0	1	10	0	0	0	0
0	0	1	0	0	1	10	0	0	0	0
0	0	1	0	0	2	14	0	0	0	0
0	0	0	0	0	1	21	0	0	0	0
0	0	1	0	0	0	7	0	0	0	0
0	0	2	0	0	0	14	0	0	0	0
0	0	0	0	0	0	4	0	0	0	0
0	0	0	0	0	0	16	0	0	0	0
0	0	3	0	0	0	6	0	0	0	0
0	0	1	0	0	0	5	0	0	0	0
0	0	2	0	0	2	7	0	0	0	0
0	0	1	0	0	0	2	0	0	0	0
0	0	0	0	0	0	14	0	0	0	0
0	0	1	0	0	0	9	0	0	0	0
0	0	0	0	0	0	13	0	0	0	0
0	0	1	1	0	0	12	0	0	0	0
0	0	6	0	0	0	7	0	0	0	0
0	0	1	0	0	0	4	0	0	0	0
0	0	0	0	0	0	1	0	0	0	0
0	0	0	0	0	0	2	0	0	1	0
0	0	2	0	0	0	10	0	0	0	0
1	0	6	0	0	0	5	0	0	0	0
0	0	1	1	0	0	1	0	0	0	0
0	0	0	0	0	0	1	0	0	0	0
0	0	0	0	0	0	11	0	0	0	0
0	0	0	1	0	0	8	0	0	0	0

0	0	0	0	0	0	1	0	0	0	0
0	0	1	0	0	0	5	0	0	0	0
0	0	2	0	0	0	3	0	0	0	0
0	0	0	0	2	0	5	0	0	0	0
0	0	1	2	0	0	8	0	0	0	0
0	0	0	0	0	0	6	0	0	0	0
0	0	0	1	0	0	9	0	0	0	0
0	0	1	0	0	0	9	0	0	0	0
0	0	0	1	0	0	8	0	0	0	0
0	0	1	0	0	0	7	0	0	0	0
0	0	0	0	0	0	5	0	0	0	0
0	0	4	1	0	0	5	0	0	0	0
0	0	0	0	0	0	8	0	0	0	0
0	0	0	0	0	1	2	0	0	0	0
0	0	1	0	1	1	5	0	0	0	0
0	0	1	0	0	0	9	0	0	0	0
0	0	0	0	0	0	4	0	0	0	0
0	0	0	0	0	0	6	0	0	0	0
0	0	0	0	0	0	6	0	0	0	0
0	0	0	0	0	0	3	0	0	0	0
0	0	0	0	0	0	4	0	0	0	0
0	0	0	0	0	1	8	0	0	0	0
0	0	0	0	0	0	7	0	0	0	0
0	0	0	0	0	0	6	0	0	0	0
0	0	0	0	0	1	9	0	0	0	0
0	0	0	0	0	0	0	0	0	0	0
0	0	0	0	0	0	0	0	0	0	0
0	0	0	0	0	0	6	0	0	0	0
0	0	0	0	0	0	2	0	0	0	0

0	0	0	0	0	0	3	0	0	0	0
0	0	0	0	0	0	4	0	0	0	0
0	0	0	0	0	0	3	0	0	0	0
0	0	0	0	0	0	0	0	0	0	0
0	0	5	0	0	0	3	0	0	0	0
0	0	0	0	0	0	3	0	0	0	0
0	0	0	1	0	0	2	0	0	0	0
0	0	1	0	0	0	1	0	0	0	0
0	0	0	0	0	0	0	0	0	0	0
0	0	0	0	0	0	0	0	0	0	0
0	0	0	0	0	0	1	0	0	0	0
0	0	1	0	0	0	0	0	0	0	0
0	0	0	0	0	0	1	0	0	0	0
0	0	0	0	0	0	0	0	0	0	0
0	0	0	0	0	0	1	0	0	0	0
0	0	0	0	0	0	0	0	0	0	0
0	0	0	0	0	0	0	0	0	0	0
0	0	0	0	0	0	0	0	0	0	0
0	0	0	0	0	0	0	0	0	0	0
0	0	0	0	0	0	0	0	0	0	0
0	0	0	0	0	0	0	0	0	0	0
0	0	1	0	0	0	0	0	0	0	0
0	0	0	0	0	0	1	0	0	0	0
0	0	0	0	0	0	0	0	0	0	0
0	0	0	0	0	0	0	0	0	0	0
0	0	0	0	0	0	0	0	0	0	0
0	0	0	0	0	0	0	0	0	0	0
0	0	1	0	0	0	0	0	0	0	0
0	0	1	0	0	0	0	0	0	0	0

0	0	0	0	0	0	1	0	0	0	0
0	0	1	0	0	0	0	0	0	0	0
0	0	0	0	0	0	1	0	0	0	0
0	0	1	0	0	0	0	0	0	0	0
0	0	0	0	0	0	6	0	0	0	0
0	0	2	0	0	0	6	0	0	0	0
0	0	0	0	0	0	1	0	0	0	0
0	0	0	0	0	1	3	0	0	0	0
0	0	0	0	0	0	3	0	0	0	0
0	0	0	0	0	1	1	0	0	0	0
0	0	0	0	0	0	3	0	0	0	0
0	0	0	1	1	0	3	0	0	0	0
0	0	0	0	0	0	6	0	0	0	0
0	0	0	0	0	0	11	0	0	0	0
0	0	1	0	0	0	4	0	0	0	0
0	0	0	1	1	0	2	0	0	0	0
0	0	2	0	0	0	0	0	0	0	0
0	0	0	1	0	0	1	0	0	0	0
0	0	0	0	0	0	3	0	0	0	0
0	0	0	0	0	0	1	0	0	0	0
0	0	1	0	0	0	2	0	0	0	0
0	0	0	0	0	0	3	0	0	0	0
0	0	0	0	0	0	2	0	0	0	0
0	0	1	0	0	0	2	0	0	0	0
0	0	0	0	0	0	2	0	0	0	0
0	0	0	1	0	0	4	0	0	0	0
0	0	2	0	0	0	8	0	0	0	0
0	0	4	0	0	0	0	0	0	0	0
0	0	1	1	1	0	7	0	0	0	0

0	0	0	0	0	0	9	0	0	0	0
0	0	0	0	0	0	4	0	0	0	0
0	0	0	0	0	1	8	0	0	0	0
0	0	1	0	0	0	4	0	0	0	0
0	0	0	0	0	0	6	0	0	0	0
0	0	0	0	0	0	3	0	0	0	0
0	0	3	0	3	0	4	0	0	0	0
0	0	0	0	0	0	9	0	0	0	0
0	0	1	0	0	0	4	0	0	0	0
0	0	1	0	0	0	6	0	0	0	0
0	0	0	0	0	0	5	0	0	0	0
0	0	0	0	0	0	6	0	0	0	0
0	0	2	0	0	1	5	0	0	0	0
0	0	0	0	0	0	3	0	0	0	0
0	0	0	0	0	0	5	0	0	0	0
0	0	0	0	0	0	1	0	0	0	0
0	0	1	0	1	0	4	0	0	0	0
0	0	0	0	2	0	4	0	0	0	0
0	0	0	0	0	0	3	0	0	0	0
0	0	0	0	0	0	1	0	0	0	0
0	0	0	0	0	0	1	0	0	0	0
0	0	0	0	0	0	1	0	0	0	0
0	0	4	0	0	0	3	0	0	0	0
0	0	1	0	0	0	3	0	0	0	0
0	0	2	0	0	0	5	0	0	0	0
1	0	0	0	0	0	2	0	0	0	0
0	0	4	0	0	1	10	0	0	0	0
0	0	0	0	0	0	4	0	0	0	0
0	0	4	0	0	0	3	0	0	0	0

0	0	0	0	0	0	0	0	0	0	0
0	0	1	0	0	0	7	0	0	0	0
0	0	0	0	0	0	2	0	0	0	0
0	0	1	0	0	1	17	0	0	0	0
0	0	0	0	0	0	8	0	0	0	0
0	0	2	0	0	0	14	0	0	0	0
0	0	2	0	0	0	13	0	0	0	0
0	0	1	0	0	0	6	0	0	0	0
0	0	1	0	0	0	5	0	0	0	0
0	0	0	0	0	0	17	0	0	0	0
0	0	0	0	0	0	2	0	0	0	0
0	0	2	0	0	0	7	0	0	0	0
0	0	2	0	0	0	3	0	0	0	0
0	0	3	0	0	0	15	0	0	0	0
0	0	1	0	0	0	5	0	0	0	0
0	0	1	0	0	0	11	0	0	0	0
0	0	0	0	0	0	3	0	0	0	0
0	0	3	0	0	0	11	0	0	0	0
0	0	0	0	0	0	13	0	0	0	0
0	0	2	0	0	0	8	0	0	0	0
0	0	1	0	0	0	7	0	0	0	0
0	0	0	0	0	0	0	0	0	0	0
0	0	1	0	0	1	28	0	0	0	0
0	0	0	0	0	0	9	0	0	0	0
0	0	1	0	0	0	15	0	0	0	0
1	0	1	0	0	0	10	0	0	0	0
0	0	0	0	0	0	7	0	0	0	0
0	0	0	0	0	0	11	0	0	0	0
0	0	2	0	0	0	7	0	0	0	0

0	0	1	0	0	1	9	0	0	0	0
0	0	0	0	0	0	14	0	0	0	0
0	0	0	0	0	0	10	0	0	0	0
0	0	1	0	0	1	17	0	0	0	0
0	0	0	0	0	0	12	0	0	0	0
0	0	1	0	0	0	20	0	0	0	0
0	0	0	0	0	0	7	0	0	0	0
0	0	0	0	0	0	6	0	0	0	0
0	0	0	0	0	0	2	0	0	0	0
0	0	0	0	0	1	9	0	0	0	0
0	0	0	0	0	0	11	0	0	0	0
0	0	0	0	0	0	27	0	0	0	0
0	0	0	0	0	0	18	0	0	0	0
0	0	0	0	0	0	15	0	0	0	0
0	0	0	0	0	0	12	0	0	0	0
0	0	0	0	0	0	8	0	0	0	0
0	0	0	0	0	0	11	0	0	0	0
0	0	3	0	0	0	11	0	0	0	0
0	0	0	0	0	0	17	0	0	0	0
0	0	0	0	0	0	12	0	0	0	0
0	0	0	0	0	0	15	0	0	0	0
0	0	0	0	0	0	14	0	0	0	0
0	0	1	0	0	0	15	0	0	0	0
0	0	3	0	0	0	14	0	0	0	0
0	0	0	0	0	0	14	0	0	0	0
0	0	0	0	0	0	20	0	0	0	0
0	0	2	0	0	0	12	0	0	0	0
1	0	4	0	0	0	12	0	0	0	0
0	0	1	0	0	0	7	0	0	1	0

0	0	0	0	0	0	6	0	0	0	0
0	0	0	0	0	0	11	0	0	0	0
1	0	1	0	0	0	12	0	0	0	0
0	0	3	0	0	0	11	0	0	0	0
0	0	0	0	0	0	3	0	0	0	0
0	0	1	0	0	0	6	0	0	0	0
0	0	2	0	0	0	6	0	0	0	0
0	0	0	0	0	0	4	0	0	0	0
0	0	0	0	0	0	11	0	0	0	0
0	0	4	0	0	0	5	0	0	0	0
0	0	3	0	0	0	9	0	0	0	0
0	0	2	0	0	0	5	0	0	0	0
0	0	2	0	0	0	18	0	0	0	0
0	0	2	0	0	0	4	0	0	0	0
0	0	0	0	0	0	10	0	0	0	0
0	0	0	0	0	0	4	0	0	0	0
1	0	6	1	0	0	18	0	0	0	0
0	0	0	0	0	0	7	0	0	0	0
0	0	1	0	0	0	9	0	0	0	0
1	0	2	1	0	0	17	0	0	0	0
0	0	0	0	0	0	22	0	0	0	0
0	0	1	0	0	0	12	0	0	0	0
0	0	0	1	0	0	7	0	0	0	0
0	0	3	2	0	0	11	0	0	0	0
0	0	0	0	0	0	18	0	0	0	0
0	0	0	0	0	0	5	0	0	0	0
0	0	0	1	0	0	3	0	0	0	0
0	0	0	0	0	0	29	0	0	0	0
0	0	2	0	0	0	4	0	0	0	0

0	0	2	0	0	0	9	0	0	0	0
1	0	0	0	0	0	6	0	0	0	0
0	0	1	0	0	0	5	0	0	0	0
0	0	0	0	0	0	9	0	0	0	0
0	0	2	0	0	0	11	0	0	0	0
0	0	0	0	0	0	7	0	0	0	0
0	0	0	0	0	0	8	0	0	0	0
0	0	0	1	0	0	2	0	0	0	0
0	0	1	1	1	0	7	0	0	0	0
0	0	0	0	0	0	7	0	0	0	0
0	0	0	0	0	0	7	1	0	0	0
0	0	0	0	0	0	3	0	0	0	0
0	0	0	1	0	0	11	0	0	0	0
0	0	0	0	0	0	8	0	0	0	0
0	0	0	0	0	0	3	0	0	0	0
0	0	0	0	0	0	9	0	0	0	0
0	0	0	0	0	0	5	0	0	0	0
0	0	0	0	0	0	10	0	0	0	0
0	0	3	0	0	0	12	0	0	0	0
0	0	1	1	0	0	10	0	0	0	0
0	0	3	0	0	0	5	0	0	0	0
0	0	1	0	0	0	7	0	0	0	0
0	0	1	0	0	0	1	0	0	0	0
0	0	0	0	0	0	4	0	0	0	0
0	0	1	2	0	0	5	0	0	0	0
0	0	1	1	0	0	10	0	0	0	0
0	0	0	0	0	0	10	0	0	0	0
0	0	2	1	0	0	3	0	0	0	0
0	0	1	0	0	0	1	0	0	0	0

0	0	0	0	0	1	5	0	0	0	0
0	0	2	0	0	0	5	0	0	0	0
0	0	0	0	0	0	4	0	0	0	0
0	0	1	0	0	0	5	0	0	0	0
0	0	1	0	0	0	1	0	0	0	0
0	1	0	0	0	0	3	0	0	0	0
0	0	0	0	1	1	1	0	0	0	0
0	0	0	0	0	0	5	0	0	0	0
0	0	0	0	0	0	2	0	0	0	0
0	1	0	0	0	1	14	0	0	0	0
0	0	0	0	0	0	1	0	0	0	0
0	0	3	0	0	2	5	0	0	0	0
0	0	0	0	0	0	7	0	0	0	0
0	0	0	2	0	0	2	0	0	0	0
0	0	0	2	0	0	3	0	0	0	0
0	0	1	0	0	0	3	0	0	0	0
2	0	0	0	0	0	0	0	0	0	0
0	0	1	0	0	0	3	0	0	0	0
0	0	1	0	0	0	1	0	0	0	0

micten	mysiad	nemato	nemert	neosin	nicaes	notoma	notsca	nuchar	olifat	olithi
0	0	0	0	0	1	1	2	0	0	0
0	0	0	0	0	0	0	0	0	0	2
0	0	0	3	0	1	0	0	0	0	1
0	0	0	1	0	0	3	1	0	0	0
0	0	0	0	0	1	0	1	0	0	0
0	0	0	1	0	6	0	2	0	0	0
0	0	0	1	0	0	0	0	0	0	0
0	0	1	4	0	0	0	2	0	0	0

0	0	0	0	0	0	0	0	0	0	1
0	0	0	1	0	0	0	0	0	0	1
0	0	0	0	0	1	0	0	0	0	0
0	0	0	0	0	0	0	0	8	0	0
1	0	0	2	0	0	0	1	2	0	0
0	0	0	5	0	0	0	0	4	0	0
0	0	0	1	0	0	0	0	6	0	2
0	0	0	2	0	0	0	0	9	0	1
1	0	0	1	0	0	0	0	10	0	1
0	0	0	2	0	0	0	0	10	0	1
0	0	0	3	0	0	0	0	8	0	0
0	0	0	1	0	0	0	0	2	0	0
0	0	0	0	0	2	0	0	0	0	2
0	0	0	1	0	0	0	0	0	0	0
0	0	0	2	0	0	0	0	0	0	0
0	0	0	1	0	0	0	1	0	0	0
0	1	0	2	0	0	0	0	0	0	0
0	1	0	1	0	0	0	0	0	0	2
0	0	0	1	0	0	0	0	0	0	0
0	0	0	2	0	5	0	1	0	0	0
0	0	0	2	0	0	0	0	0	0	0
0	0	0	2	0	1	0	2	0	0	0
0	0	0	0	0	5	0	0	0	0	0
0	0	0	5	0	2	0	0	0	0	1
0	0	0	1	0	4	0	0	0	0	0
0	0	0	0	0	2	0	2	0	0	0
0	0	0	0	0	1	0	0	0	0	0
0	0	0	0	0	0	1	2	0	0	0
0	0	0	1	0	0	0	2	0	0	0

0	0	0	1	0	0	0	0	0	0	0
0	0	0	0	0	0	0	0	0	0	0
0	0	0	1	0	0	0	0	0	0	0
0	0	0	2	0	0	0	1	0	0	0
0	0	0	1	0	0	0	0	0	0	0
0	0	0	2	0	0	0	1	0	0	1
0	0	0	0	0	0	0	0	0	0	0
0	0	0	0	0	0	0	0	0	0	0
0	0	0	2	0	0	0	0	0	0	0
0	0	0	1	0	0	0	0	0	0	0
0	0	0	0	0	0	0	0	0	0	0
0	0	0	2	0	0	0	0	0	0	0
0	0	0	1	0	0	0	0	0	0	0
0	0	0	1	0	0	0	0	0	0	0
0	0	0	1	0	0	0	0	0	0	0
0	0	0	1	0	0	0	1	0	0	0
0	0	0	1	0	0	0	0	0	0	0
0	0	0	3	0	0	0	0	0	0	0
0	0	0	2	0	1	0	0	0	0	0
0	0	0	2	0	0	0	0	0	0	0
0	0	0	1	0	1	0	0	0	0	0
0	0	0	2	0	0	0	0	0	0	0
0	0	0	0	0	0	0	1	0	0	0
0	0	0	0	0	0	0	0	0	0	0
0	0	0	0	0	0	0	1	0	0	0
0	0	0	1	0	0	0	1	0	0	0
0	0	0	0	0	0	0	0	0	0	0
0	0	0	0	0	0	0	0	0	0	0
0	0	0	1	0	0	0	0	0	0	0

0	0	0	0	0	0	0	0	0	0	0
0	1	0	0	0	0	0	0	0	0	0
0	1	0	0	0	0	0	1	0	0	0
0	0	0	0	0	0	0	0	0	0	0
0	0	0	1	0	0	0	0	0	0	0
0	0	0	0	0	0	0	0	0	0	0
0	1	0	2	0	0	0	0	0	0	0
0	0	0	2	0	0	0	0	0	0	0
0	0	0	6	0	0	0	1	0	0	0
0	0	0	0	0	0	0	0	0	0	0
0	0	0	0	0	0	0	0	0	0	0
0	0	0	0	0	0	0	0	0	0	0
0	0	0	1	0	0	0	0	0	0	0
0	0	0	2	0	0	0	0	0	0	0
0	0	0	1	0	0	0	0	1	0	0
0	0	0	0	0	0	0	0	0	0	0
0	0	0	0	0	0	0	0	0	0	0
0	0	0	3	0	0	0	1	0	0	1
0	0	0	2	0	0	0	0	0	0	0
0	0	0	2	0	0	0	0	0	0	0
0	0	0	0	0	0	0	0	0	0	0
0	0	0	2	0	0	0	0	1	0	0
0	0	0	2	0	0	0	0	0	0	0
0	0	0	1	0	0	0	1	0	0	1
0	0	0	0	0	0	0	0	0	0	0
0	0	0	0	0	0	0	0	2	0	0
0	0	0	2	0	0	0	0	1	0	0
0	0	0	1	0	0	0	0	0	0	0
0	0	0	3	0	0	0	0	2	0	1

0	0	0	2	0	0	0	1	4	0	0
0	0	0	0	0	0	0	0	1	0	1
0	0	0	2	0	0	0	0	0	0	0
0	0	0	1	0	0	0	0	2	0	1
0	0	0	0	1	0	0	0	4	0	0
0	0	0	0	0	0	0	0	3	0	0
0	0	0	1	0	0	0	0	2	0	0
0	0	0	0	0	0	0	0	16	0	0
0	0	0	4	0	0	0	0	15	0	0
0	0	0	1	0	0	0	0	10	0	0
0	0	0	3	0	0	0	0	5	0	0
0	0	0	3	0	0	0	0	11	0	0
0	0	0	0	0	0	0	1	13	0	0
1	0	0	0	0	0	0	0	20	0	0
1	0	0	2	0	0	0	0	5	0	0
0	0	0	1	0	1	0	0	14	0	0
0	0	0	3	0	1	0	0	0	0	0
3	0	0	0	0	0	0	0	12	0	0
0	0	0	0	0	0	0	0	4	0	0
0	0	0	2	0	0	0	1	0	0	0
0	0	0	0	1	0	0	0	0	0	0
1	0	0	1	0	0	0	0	2	0	0
0	0	0	2	0	1	0	0	0	0	0
0	0	0	4	0	0	0	0	0	0	0
0	0	0	2	0	0	0	0	0	0	0
0	0	0	1	0	0	0	0	0	0	0
0	0	0	0	0	0	0	0	0	0	0
0	0	0	0	0	0	0	1	1	0	0
0	0	0	0	0	1	0	1	0	0	0

0	1	0	0	0	0	0	0	0	0	0
0	0	0	2	0	0	0	1	0	0	0
0	0	0	1	0	1	0	0	0	0	0
0	0	0	0	0	0	0	0	0	0	0
0	0	0	0	0	0	0	0	0	0	0
0	0	0	1	0	0	0	0	1	0	0
0	0	0	1	0	0	0	0	0	0	1
0	0	0	3	0	0	0	0	0	0	0
0	0	0	3	0	0	0	0	0	0	0
0	0	0	0	0	0	0	0	0	0	0
0	0	0	0	0	0	0	0	0	0	0
0	0	0	0	0	0	0	0	0	0	0
0	0	0	1	0	0	0	0	0	0	1
0	0	0	5	0	0	0	2	0	0	0
0	0	0	0	0	0	0	0	0	0	0
0	0	0	1	0	0	0	1	0	0	0
0	0	0	2	0	0	0	0	0	0	0
0	0	0	0	0	1	0	0	0	0	0
0	0	0	0	0	0	0	0	0	0	0
0	0	0	1	0	0	0	0	0	0	0
0	0	0	1	0	0	0	0	0	0	0
0	0	0	0	0	0	0	0	0	0	0
0	0	0	1	0	0	0	2	0	0	0
0	0	0	2	0	0	0	0	0	0	0
0	0	0	0	0	0	0	0	0	0	0
0	0	0	0	0	0	0	0	0	0	0
0	0	0	3	0	0	0	0	0	0	0
0	0	0	0	0	0	0	1	0	0	0
0	0	0	0	0	0	0	0	0	0	0

0	0	0	0	0	0	0	1	0	0	0
0	0	0	0	0	0	0	0	0	0	0
0	0	0	1	0	0	0	2	0	0	0
0	0	0	1	0	0	0	0	0	0	0
0	0	0	1	0	0	0	0	0	0	0
0	0	0	0	0	0	0	1	0	0	0
0	0	0	0	0	0	0	0	0	0	0
0	0	0	1	0	0	0	0	0	0	0
0	0	0	1	0	0	0	0	0	0	0
0	0	0	1	0	0	0	0	0	0	0
0	0	0	2	0	0	0	0	0	0	0
0	0	0	1	0	0	0	1	0	0	0
0	0	0	0	0	1	1	0	0	0	0
0	0	0	1	0	0	0	0	0	0	0
0	0	0	2	0	0	0	0	0	0	0
0	0	0	2	0	0	0	1	0	0	0
0	0	0	0	0	1	0	0	0	0	0
0	0	0	5	0	0	0	0	0	0	1
0	0	0	1	0	0	0	0	0	0	0
0	0	0	0	0	0	0	0	0	0	1
0	0	0	2	0	0	0	0	0	0	0
0	0	0	1	0	0	0	1	0	0	0
0	0	0	0	0	0	0	0	0	0	1
0	0	0	1	0	0	0	0	0	0	0
0	0	0	0	0	0	0	1	0	0	0
0	0	0	0	0	1	0	3	0	0	0
0	0	0	2	0	0	0	1	0	0	1
0	0	0	0	0	0	0	1	0	0	0
0	0	0	1	0	0	0	0	0	0	1

0	0	0	4	0	0	0	4	0	0	1
0	0	0	0	0	0	0	4	0	0	0
0	0	0	1	0	1	0	0	0	0	0
0	0	0	3	0	0	0	0	0	0	1
0	0	0	0	0	0	0	1	0	0	0
0	0	0	3	0	0	0	1	0	0	0
0	0	0	0	0	0	0	0	0	0	0
0	0	0	0	0	0	0	0	0	0	0
0	0	0	1	0	0	0	0	0	0	2
0	0	1	2	0	0	0	0	0	0	1
0	0	0	0	0	0	0	0	0	0	0
0	0	0	1	0	0	0	0	0	0	0
0	0	0	2	0	4	0	0	0	0	1
0	0	0	3	0	1	0	0	0	0	1
0	0	0	2	0	1	0	0	0	0	0
0	0	0	0	0	0	0	1	0	0	0
0	0	0	0	0	2	0	0	0	0	0
0	0	0	1	0	2	0	0	0	0	2
0	0	0	0	0	0	0	0	0	0	1
0	0	0	0	0	0	0	0	0	0	0
0	0	0	4	0	0	0	0	0	0	0
0	0	0	1	0	3	0	3	1	0	0
0	0	0	2	0	0	0	0	0	0	0
0	0	0	1	0	1	0	0	0	0	0
0	0	0	2	0	0	0	0	0	0	0
0	0	0	0	0	0	0	0	0	0	0
0	0	0	2	0	0	0	0	0	0	1
0	0	0	0	0	0	0	0	0	0	0
0	0	0	1	0	0	0	0	0	2	4

0	0	0	2	0	0	0	3	0	0	0
0	0	0	2	0	3	0	0	0	0	7
0	0	0	1	0	0	0	1	0	0	0
0	0	0	2	0	0	0	0	0	0	0
0	0	0	1	0	0	0	0	0	0	0
0	0	0	0	0	0	0	0	1	0	0
0	0	0	3	0	1	0	0	0	0	0
0	0	0	1	0	0	0	2	0	0	0
0	0	0	0	0	0	0	1	0	0	1
0	0	0	1	0	0	0	2	0	0	0
0	0	0	1	0	0	0	0	0	0	0
0	0	0	0	0	0	0	0	0	0	1
0	0	0	1	0	0	0	2	0	0	0
0	0	0	0	0	0	0	0	0	0	0
0	0	0	0	0	0	0	0	0	0	0
0	0	0	2	0	1	0	1	0	0	0
0	0	0	1	0	0	0	1	0	0	0
0	0	0	0	0	0	0	0	0	0	0
0	0	0	1	0	0	0	0	0	0	0
0	0	0	1	0	0	0	0	0	0	0
0	0	0	0	0	0	0	0	0	0	0
0	0	0	0	0	1	0	0	0	0	0
0	0	0	2	0	0	0	0	0	0	2
0	0	0	1	0	0	0	0	0	0	0
0	0	0	0	0	0	0	0	0	0	0
0	0	0	0	0	0	0	0	0	0	0
0	0	0	1	0	0	0	0	0	0	1
0	0	0	3	0	1	0	0	0	0	0
0	0	0	1	0	0	0	0	0	0	0

0	0	0	0	0	0	0	0	0	0	0
0	0	0	0	0	0	0	0	0	0	0
0	0	0	1	0	0	0	0	0	0	0
0	0	0	1	0	0	0	0	0	0	0
0	0	0	2	0	0	0	1	0	0	0
0	0	0	0	0	0	0	0	0	0	0
0	0	0	4	0	0	0	1	0	0	0
0	0	0	3	0	0	0	0	0	0	0
0	0	0	0	0	0	0	0	0	0	0
0	0	0	0	0	0	0	0	0	0	0
0	0	0	0	0	0	0	0	0	0	0
0	0	0	1	0	0	0	0	0	0	0
0	0	0	1	0	0	0	1	0	0	0
0	0	0	0	0	0	0	0	0	0	0
0	0	0	0	0	0	0	0	0	0	0
0	0	0	0	0	0	0	0	0	0	0
0	0	0	0	0	0	0	0	0	0	0
0	0	0	0	0	0	0	1	0	0	0
0	0	0	4	0	0	0	1	0	0	0
0	0	0	0	0	0	0	0	0	0	1
0	0	0	0	0	1	0	0	0	0	1
0	0	0	0	0	0	0	0	0	0	0
0	0	0	0	0	0	0	0	0	0	0
0	0	0	0	0	0	0	0	0	0	0
0	0	0	0	0	0	0	0	0	0	0
0	0	0	1	0	0	0	0	0	0	0
0	0	0	0	0	0	0	0	0	0	0
0	0	0	0	0	0	0	2	0	0	0
0	0	0	1	0	0	0	0	0	0	0

0	0	0	1	0	0	0	0	0	0	0
0	0	0	2	0	0	0	0	0	0	0
0	0	0	0	0	0	0	0	0	0	0
0	0	0	1	0	0	0	0	0	0	0
0	0	0	0	0	0	0	0	0	0	0
0	0	0	0	0	0	0	0	0	0	0
0	0	0	2	0	0	0	0	0	0	0
0	0	0	1	0	0	0	1	0	0	0
0	0	0	1	0	0	0	0	0	0	0
0	0	0	1	0	0	1	0	0	0	0
0	0	0	3	0	0	0	2	2	0	0
0	0	0	0	0	0	0	0	1	0	0
0	0	0	0	0	0	0	0	10	0	0
0	0	0	1	0	0	0	0	0	0	0
0	1	0	0	0	0	0	0	0	0	0
0	0	0	0	0	0	0	0	1	0	0
0	0	0	0	0	0	0	1	1	0	1
0	0	0	2	0	0	0	1	1	0	0
0	0	0	2	0	0	0	0	1	0	0
0	0	0	0	0	0	0	0	11	0	0
4	0	0	0	0	0	1	1	14	0	1
2	0	0	3	0	0	0	0	17	0	1
0	0	0	1	0	0	0	1	7	0	0
0	0	0	0	0	0	0	0	8	0	0
5	0	0	0	0	0	0	0	12	0	0
0	0	0	0	0	0	0	0	13	0	0
0	0	0	2	0	0	0	0	1	0	1
0	0	0	1	0	0	0	0	2	0	0
0	0	0	1	0	0	0	0	4	0	0

0	0	0	0	0	0	0	1	9	0	1
0	0	0	1	0	0	0	0	4	0	0
0	0	0	0	0	0	0	0	2	0	1
0	0	0	1	0	0	0	0	5	0	0
0	0	0	0	0	0	0	0	4	0	0
0	0	0	0	0	0	0	0	3	0	0
0	0	0	1	0	0	0	0	3	0	0
0	0	0	0	0	0	0	0	11	0	0
0	0	0	1	0	0	0	1	9	0	2
0	0	0	0	0	0	0	0	2	0	0
0	0	0	1	0	0	0	0	9	0	0
0	0	0	0	0	0	0	0	3	0	0
0	0	0	2	0	0	0	0	3	0	1
0	0	0	0	0	0	0	0	1	0	0
1	0	0	0	0	0	0	0	2	0	0
1	0	0	0	0	0	0	0	8	0	1
1	0	0	0	0	0	0	0	2	0	1
0	0	0	1	0	0	0	0	1	0	0
0	0	0	0	0	0	0	0	1	0	0
0	0	0	1	0	0	0	0	1	0	0
0	0	0	1	0	0	0	0	2	0	0
0	0	0	0	0	0	0	1	0	0	0
0	0	0	0	0	1	0	0	1	0	0
0	0	0	1	0	1	0	0	0	0	0
0	0	0	0	0	0	0	1	1	0	0
0	0	0	1	0	1	0	1	2	0	0
0	0	0	1	0	1	0	2	0	0	0
0	0	0	0	0	0	0	1	0	0	0
0	0	0	0	0	0	0	1	0	0	0

0	0	0	1	0	0	0	1	0	0	0
0	0	0	0	0	0	0	0	0	0	1
0	0	0	1	0	0	0	1	0	0	1
0	0	0	0	0	0	0	0	0	0	0
0	0	0	1	0	0	0	3	0	0	0
0	0	0	0	0	0	1	1	0	0	0
0	0	0	2	0	0	0	0	0	0	0
0	0	0	1	0	0	0	0	0	0	0
0	0	0	0	0	0	0	2	0	0	0
0	0	0	3	0	0	0	1	0	0	0
0	0	0	1	0	0	0	1	0	0	0
0	0	0	0	0	2	0	1	0	0	0
0	0	0	1	0	0	0	1	0	0	0
0	0	0	0	0	0	0	3	0	0	0
0	0	0	2	0	0	0	0	0	0	1
0	0	0	1	0	0	0	0	0	0	0
0	0	0	0	0	0	0	2	0	0	0
0	0	0	1	0	0	0	0	0	0	0
0	0	0	3	0	0	0	0	0	0	0
0	0	0	1	0	0	0	0	0	0	0
0	0	0	0	0	0	0	0	0	0	0
0	0	0	1	0	0	0	1	0	0	0
0	0	0	0	0	0	0	0	0	0	0
0	0	0	0	0	0	0	0	0	0	0
0	0	0	1	0	0	0	0	0	0	0
0	0	0	0	0	0	0	0	0	0	0
0	0	0	1	0	0	0	0	0	0	0
0	0	0	1	1	0	0	0	0	0	0
0	0	0	0	0	0	0	0	0	0	0

0	0	0	2	0	0	0	1	0	0	1
0	0	0	0	0	0	0	0	0	0	1
0	0	0	0	0	0	0	0	0	0	0
0	0	0	1	0	0	0	0	0	0	1
0	0	0	1	0	0	0	0	0	0	0
0	0	0	0	0	0	0	0	1	0	0
0	0	0	1	0	0	0	0	0	0	0
0	0	0	0	0	0	0	0	0	0	0
0	0	0	1	0	0	0	1	0	0	0
0	0	0	0	0	0	0	0	0	0	0
0	0	0	1	0	0	0	0	0	0	0
0	0	0	1	0	0	0	0	1	0	0
0	0	0	1	0	0	0	1	1	0	0
0	0	0	0	0	0	0	0	0	0	0
0	0	0	0	0	1	0	0	0	0	0
0	0	0	3	0	0	0	0	1	0	0
0	0	0	0	0	0	0	1	0	0	0
0	0	0	1	0	0	0	4	0	0	0
0	0	0	3	0	0	0	0	1	0	0
0	0	0	1	0	0	0	1	0	0	1
0	0	0	0	0	0	0	1	0	0	0
0	1	0	2	0	0	0	2	0	0	0
0	0	0	2	0	0	0	0	0	0	0
0	1	0	0	0	6	0	3	1	0	1
0	0	0	0	0	0	0	0	0	0	0
0	0	0	2	0	1	0	1	0	0	0
0	0	0	0	0	4	0	0	0	0	0
0	0	0	4	0	3	0	2	0	0	0
0	0	0	2	0	1	0	3	0	0	0

0	0	0	3	0	2	0	0	0	0	1
0	0	0	0	0	0	0	0	0	0	0
0	0	0	3	0	1	0	0	0	0	1
0	0	0	1	0	2	0	0	0	0	1
0	0	0	1	0	0	0	0	0	0	0
0	0	0	0	0	1	0	0	0	0	1
0	0	0	3	0	9	0	0	0	0	1
0	0	0	1	0	3	0	0	0	0	0
0	0	0	2	0	0	0	1	0	0	0
0	0	0	2	0	0	0	1	0	0	0
0	0	0	1	0	0	0	3	0	0	0
0	0	0	1	0	0	0	3	0	0	0
0	0	0	1	0	0	0	0	0	0	1
0	0	0	0	0	0	0	0	0	0	0

hetfil	ischyr	laspar	lepido	levgra	limpul	lumbri	lysian	maclil	macste	magdak
1	0	0	0	0	0	0	0	10	0	2
0	0	0	0	0	1	0	0	8	0	5
3	0	0	0	0	0	0	0	6	0	3
3	0	0	0	0	0	0	0	11	0	2
1	0	0	0	0	0	0	0	0	0	6
1	0	0	0	0	0	0	0	2	0	2
0	0	0	0	0	0	0	0	5	0	5
1	0	0	0	0	0	0	0	4	8	0
4	0	0	0	0	0	0	0	1	1	1
4	0	0	0	0	0	0	0	2	3	2
2	0	0	0	0	0	0	0	2	7	1
2	0	0	0	0	0	0	0	5	3	2
3	0	0	0	0	0	0	0	1	2	4
0	0	0	0	0	0	0	0	2	1	1
1	0	0	0	0	0	0	0	1	2	1
2	0	0	0	0	0	0	0	1	1	0
1	0	0	0	0	0	0	0	6	0	8
1	0	0	0	0	0	0	0	3	0	1
0	0	0	0	0	0	0	0	2	2	4
3	0	0	0	0	0	0	0	2	3	4
1	0	0	0	0	0	0	0	2	1	2
0	0	0	0	0	0	0	0	5	6	3
3	0	1	0	0	0	0	0	17	0	2
3	0	0	0	0	0	0	0	9	0	5
2	1	0	0	0	0	0	0	3	4	0
4	0	0	0	0	0	0	0	4	2	2
0	0	0	0	0	0	0	0	3	0	3
0	0	0	0	0	0	0	0	3	2	1

0	0	0	0	0	0	0	0	3	0	0
0	0	0	0	0	0	0	0	6	0	1
2	0	0	0	0	0	0	0	1	1	0
2	0	0	0	0	0	0	0	4	1	1
3	0	0	0	0	0	0	0	2	6	2
1	0	0	0	0	0	0	0	3	4	0
1	0	0	0	0	0	0	0	3	0	0
0	0	0	0	0	0	0	0	1	1	1
0	0	0	0	0	0	0	0	7	2	0
0	0	0	0	0	0	0	0	1	0	0
1	0	0	0	0	0	0	0	5	1	0
1	0	0	1	0	0	0	0	5	2	3
4	0	0	0	0	0	0	0	8	4	4
1	0	0	0	0	0	0	0	7	3	1
0	0	0	0	0	0	0	0	10	0	0
0	0	0	0	0	0	0	0	9	0	0
2	0	0	0	0	0	0	3	6	1	0
3	0	0	0	0	0	0	0	3	0	4
3	0	0	0	0	0	0	0	8	1	0
2	0	0	0	0	0	0	0	6	2	0
0	0	0	0	0	0	0	0	6	0	0
1	0	0	0	0	0	0	0	9	4	0
5	0	0	0	0	0	0	0	6	0	12
1	0	0	0	0	0	0	0	1	1	0
0	0	0	0	0	0	0	0	1	0	0
0	0	0	0	0	0	0	0	0	0	0
0	0	0	0	0	0	0	0	1	2	0
1	0	0	0	0	0	0	0	0	2	1
0	0	0	0	0	0	0	1	7	3	0

11	0	0	0	0	0	0	0	8	0	6
0	0	0	0	0	0	0	0	1	3	1
6	0	0	0	0	0	0	0	9	0	2
0	0	0	0	0	0	0	0	2	0	2
0	0	0	0	0	0	0	0	0	0	0
0	0	0	0	0	0	0	0	1	0	8
2	0	0	0	0	0	0	0	4	3	0
1	0	0	0	0	1	0	0	3	2	1
1	0	0	0	0	0	0	0	2	1	0
4	0	0	0	1	0	0	0	2	0	10
1	0	0	0	0	0	0	0	0	0	1
0	0	0	0	0	0	0	0	1	0	0
0	0	0	0	0	0	0	0	3	2	0
2	0	0	0	0	0	0	0	4	4	0
0	0	0	0	0	0	0	0	1	1	2
1	0	0	0	0	0	0	0	2	2	0
4	0	0	0	0	0	0	0	3	0	3
0	0	0	0	0	0	0	0	2	0	6
1	0	0	0	0	0	0	0	1	0	5
0	0	0	0	0	0	0	0	0	1	0
0	0	0	0	0	0	0	0	1	0	3
0	0	16	0	0	0	0	0	4	0	0
2	0	0	0	0	0	0	0	1	0	0
2	0	0	0	0	0	0	0	2	0	0
4	0	0	0	0	0	0	0	2	1	8
1	0	0	0	0	0	0	0	0	0	3
0	0	0	0	0	0	0	0	2	1	5
2	0	0	0	0	0	0	0	2	0	2
2	0	0	0	0	0	1	0	5	2	0

4	0	0	0	0	0	0	0	2	3	2
1	0	0	0	0	0	0	0	5	0	0
9	0	0	0	0	0	0	1	5	0	1
3	0	0	0	0	0	0	0	3	3	0
0	0	0	0	0	0	0	0	3	0	0
0	0	0	0	0	0	0	0	3	0	0
0	0	0	0	0	0	0	3	4	0	0
3	0	1	0	0	0	0	2	8	0	0
0	0	0	0	0	0	0	0	2	1	2
4	0	0	1	0	0	0	0	2	3	0
0	0	0	0	0	0	0	0	1	2	2
0	0	0	0	0	0	0	0	2	1	1
0	0	0	0	0	0	0	0	0	4	0
0	0	22	0	0	0	0	0	2	0	0
1	0	0	0	0	0	0	0	3	0	0
0	0	10	0	0	0	0	0	0	0	0
0	0	0	0	0	0	0	0	2	0	0
0	0	0	0	0	0	0	0	3	0	1
0	0	0	0	0	0	0	0	0	0	0
0	0	0	0	0	0	0	0	2	0	0
4	0	0	0	0	0	0	0	7	0	0
5	0	0	0	0	0	0	0	6	7	3
0	0	0	0	0	0	0	0	2	2	0
5	0	0	0	0	0	0	0	4	2	1
6	0	0	0	0	0	0	0	3	3	1
3	0	0	0	0	0	0	0	1	3	0
1	0	0	0	0	0	0	0	3	1	1
0	0	0	0	0	0	0	0	3	1	1
1	0	0	0	0	0	0	0	5	4	0

0	0	0	0	0	0	0	0	2	1	2
1	0	4	0	0	0	0	0	4	0	0
2	0	1	0	0	0	0	0	17	0	0
0	0	1	0	0	0	0	0	7	0	0
1	0	1	0	0	0	0	1	18	0	0
1	0	2	0	0	0	0	0	6	0	1
1	0	1	0	0	0	0	0	6	0	0
0	0	4	0	0	0	0	1	7	0	3
2	0	8	0	0	0	0	0	20	0	2
3	0	0	0	0	0	0	0	25	0	0
0	0	0	0	0	0	0	0	13	0	0
0	0	0	0	0	0	0	0	3	0	0
1	0	0	0	0	0	0	0	3	1	0
0	0	0	0	0	0	0	0	4	1	1
0	0	0	0	0	0	0	0	3	0	0
3	0	0	0	0	0	0	0	1	1	0
2	0	0	0	0	0	0	0	7	2	0
0	0	0	0	0	0	0	0	3	0	0
3	0	0	0	0	0	0	0	4	1	0
1	0	0	0	0	0	0	0	4	2	0
2	0	0	0	0	0	0	1	8	7	2
1	0	0	0	0	0	0	1	10	7	1
0	0	0	0	0	0	0	2	3	0	0
0	0	0	0	0	0	0	0	1	0	0
2	0	1	0	0	0	0	0	11	0	8
0	0	0	0	0	0	0	0	5	4	0
0	0	0	0	0	0	0	1	1	3	0
1	0	0	0	0	0	0	0	5	0	0
0	0	0	0	0	0	0	0	2	0	1

0	0	0	0	0	0	0	0	1	2	0
0	0	0	0	0	0	0	0	4	0	0
2	0	0	0	0	0	0	0	9	0	0
2	0	0	0	0	0	0	0	5	0	4
2	0	0	0	0	0	0	0	13	0	6
1	0	0	0	0	0	0	0	17	0	1
5	0	0	0	0	0	0	0	19	0	12
1	0	0	0	0	0	0	0	14	0	1
2	0	0	0	0	0	0	0	14	0	8
3	0	0	0	0	0	0	0	15	0	3
0	0	0	0	0	0	0	0	3	1	1
3	0	0	0	0	0	0	0	11	0	4
2	0	0	0	0	0	0	0	6	1	5
3	0	0	0	0	0	0	0	11	0	3
1	0	0	0	0	0	0	0	3	0	0
1	0	0	0	0	0	0	0	3	0	0
1	0	0	0	0	0	0	0	7	0	0
3	0	1	0	0	0	0	0	8	0	0
1	0	0	0	0	0	0	0	1	0	0
0	0	0	0	0	0	0	0	3	0	0
1	0	0	0	1	0	0	0	0	0	0
1	0	0	0	0	0	0	0	5	0	0
0	0	0	0	0	0	0	0	2	0	0
0	0	0	0	0	0	0	0	2	0	0
0	0	1	0	0	0	0	1	11	0	2
1	0	0	0	0	11	0	0	9	0	2
0	0	0	0	0	0	0	0	19	0	4
0	0	0	0	0	0	0	0	4	0	0
1	0	0	0	0	0	0	0	12	0	4

2	0	1	0	0	0	0	0	11	0	2
1	0	0	0	0	0	0	0	7	0	0
0	0	0	0	0	0	0	0	9	0	0
1	0	0	0	0	0	0	0	8	0	1
0	0	1	0	0	0	0	0	8	0	0
0	0	0	0	0	0	0	0	8	0	0
0	0	0	0	0	0	0	0	7	0	0
0	0	0	0	0	0	0	0	5	0	1
0	0	1	0	0	0	0	0	8	0	0
0	0	0	0	0	0	0	0	5	0	1
0	0	0	0	0	0	0	0	8	0	0
0	0	0	0	0	0	0	0	2	0	0
0	0	1	0	0	0	0	0	5	0	0
1	0	0	0	0	0	0	0	8	0	0
0	0	0	0	0	0	0	0	12	0	0
0	0	2	0	0	0	0	0	10	0	1
0	0	1	0	0	0	0	0	4	0	0
0	0	0	0	0	0	0	0	13	0	0
0	0	3	0	0	0	0	1	4	0	0
0	0	3	0	0	0	0	0	9	0	0
0	0	0	0	0	0	0	0	15	0	0
0	0	10	0	0	0	0	0	6	0	0
3	0	7	0	0	0	0	0	6	0	0
3	0	9	0	0	0	0	0	6	0	0
0	0	1	0	0	0	0	0	6	0	0
2	0	4	0	0	0	0	0	8	0	0
0	0	5	0	0	0	0	0	2	0	0
0	0	2	0	0	0	0	0	3	0	0
0	0	0	0	0	0	0	0	5	0	0

0	0	3	0	0	0	0	0	1	0	0
0	0	0	0	0	0	0	0	2	0	0
0	0	0	0	0	0	0	0	2	0	0
0	0	0	0	0	0	0	0	3	0	0
0	0	0	0	0	0	0	0	1	0	0
1	0	0	0	0	0	0	0	1	0	0
0	0	0	0	0	0	0	0	1	0	0
0	0	0	0	0	0	0	0	1	0	0
0	0	0	0	0	0	0	0	19	0	0
0	0	0	0	0	0	0	0	4	0	0
0	0	0	0	0	0	0	0	4	0	0
0	0	0	0	0	0	0	0	2	0	0
0	0	1	0	0	5	0	1	2	0	0
1	0	0	0	0	0	0	0	9	0	0
0	0	1	0	0	0	0	0	2	0	1
0	0	1	0	0	0	0	0	5	0	0
0	0	1	0	0	0	0	0	6	0	0
0	0	1	0	0	0	0	0	7	0	0
0	0	2	0	0	0	0	1	4	0	0
0	0	0	0	0	0	0	0	4	0	1
0	0	2	0	0	0	0	0	5	0	0
2	0	0	0	0	0	0	0	5	0	0
0	0	0	0	0	0	0	0	2	0	1
0	0	0	0	0	0	0	0	4	0	0
3	0	0	0	0	0	0	0	4	0	1
3	0	0	0	0	0	0	0	2	0	1
0	0	0	0	0	0	0	0	2	0	0
0	0	0	0	0	0	0	0	5	0	0
1	0	0	0	0	0	0	0	4	0	0

0	0	0	0	0	0	0	0	3	0	0
0	0	0	0	0	0	0	0	4	0	2
0	0	0	0	0	0	0	0	0	0	0
1	0	0	0	0	0	0	0	5	1	0
1	0	0	0	0	0	0	0	1	0	0
1	0	0	0	0	0	0	0	4	0	2
0	0	0	0	0	0	0	0	3	0	0
0	0	0	0	0	0	0	0	5	0	1
3	0	0	0	0	0	0	0	4	0	1
1	0	0	0	0	0	0	0	6	0	1
3	0	0	0	0	0	0	0	7	0	6
4	0	0	0	0	0	0	0	8	0	0
3	0	0	0	0	0	0	0	8	0	4
1	0	0	0	0	0	0	0	5	0	1
1	0	0	0	0	0	0	0	2	0	2
2	0	0	0	0	0	0	0	5	0	0
1	0	0	0	0	0	0	0	4	0	0
2	0	0	0	0	0	0	0	8	0	7
1	0	0	0	0	0	0	0	6	1	1
7	0	0	0	0	0	0	0	7	0	2
2	0	0	0	0	0	0	0	3	7	0
1	0	0	0	0	0	0	0	11	0	1
3	0	0	0	0	0	0	0	3	0	5
7	0	0	0	0	0	0	0	6	0	13
5	0	0	0	0	0	0	0	12	0	2
4	0	0	0	0	0	0	0	11	1	1
5	0	0	0	0	0	0	0	15	0	1
0	0	0	0	0	0	0	0	5	2	0
1	0	0	0	0	0	0	0	8	6	0

2	0	0	0	0	0	0	0	6	2	0
4	0	0	0	0	0	0	0	8	3	3
3	4	0	0	0	0	0	0	4	1	2
1	0	0	0	0	0	0	0	6	3	6
0	0	0	0	0	0	0	0	4	2	1
1	0	0	0	0	0	0	0	6	4	0
4	0	0	0	0	0	0	0	4	11	0
3	0	0	0	0	0	0	0	8	5	1
4	0	0	0	0	0	0	0	9	9	3
1	0	0	0	0	0	0	0	9	4	2
1	0	0	0	0	0	0	0	6	3	3
0	0	0	0	0	0	0	0	2	0	2
0	0	0	0	0	0	0	0	10	5	0
7	0	0	0	0	0	0	0	5	16	12
8	0	0	0	0	0	0	0	4	2	8
1	0	0	0	0	0	0	0	5	1	0
1	0	0	0	0	0	0	0	5	1	1
2	0	0	0	0	0	0	0	4	1	0
1	0	0	0	0	0	0	0	1	1	4
1	0	0	0	0	0	0	0	1	0	2
1	0	0	0	0	0	0	0	4	2	4
2	0	0	0	0	0	0	0	3	4	1
0	0	0	0	0	0	0	0	4	4	0
0	0	0	0	0	0	0	0	1	2	2
0	0	0	0	0	0	0	0	1	2	0
3	0	0	0	0	0	0	0	1	0	0
1	0	0	0	0	0	0	0	6	0	1
0	0	0	0	0	0	0	0	6	1	0
6	0	0	0	0	0	0	0	1	0	0

1	0	0	0	0	0	0	0	1	1	1
5	0	0	0	0	0	0	0	3	1	1
4	0	0	0	0	0	0	0	3	0	0
7	0	0	0	0	0	0	0	4	1	0
8	0	0	1	0	0	0	0	5	2	0
1	0	0	0	0	0	0	0	2	1	1
0	0	0	0	0	0	0	0	0	0	0
0	0	0	0	0	0	0	0	1	1	0
0	0	0	0	0	0	0	0	1	0	1
1	0	0	0	0	0	0	0	2	0	4
9	0	0	0	0	0	0	0	3	0	2
3	0	0	0	0	0	0	0	3	0	4
10	0	0	0	0	0	0	0	2	3	6
12	1	0	0	0	0	0	0	3	4	1
12	0	0	0	0	0	0	0	8	6	0
4	0	0	0	0	0	0	0	3	0	0
0	0	0	0	0	0	0	0	0	0	3
0	0	0	0	0	0	0	0	4	1	0
5	0	0	0	0	0	0	0	1	0	2
3	0	0	0	0	0	0	0	2	4	2
5	0	0	0	0	0	0	0	2	3	0
5	0	0	0	0	0	0	0	3	6	2
2	0	0	0	0	0	0	0	1	0	2
4	0	0	0	0	0	1	0	4	6	3
10	0	0	0	0	0	0	0	5	1	4
4	0	0	0	0	0	0	0	5	1	4
5	0	0	0	0	0	0	0	2	2	2
3	0	0	0	0	0	0	0	5	0	2
4	0	0	0	0	0	0	0	1	0	1

3	0	0	0	0	0	0	0	2	0	3
4	0	0	0	0	0	0	0	2	1	2
4	0	0	0	0	0	0	0	2	0	7
0	0	0	0	0	0	0	0	2	0	2
1	0	0	0	2	0	0	0	0	0	1
2	0	0	0	0	0	0	0	4	1	0
1	0	0	0	0	0	0	0	1	1	0
1	0	0	0	0	0	0	0	0	1	2
0	0	0	0	0	0	0	0	1	0	0
2	0	0	0	0	0	0	0	4	4	5
5	0	0	0	0	0	0	0	4	1	1
2	0	0	0	0	0	0	0	4	4	7
0	0	0	0	0	0	0	0	2	2	1
1	0	0	0	0	0	0	0	0	3	7
0	0	0	0	0	0	0	0	3	2	0
0	0	0	0	0	0	0	0	5	1	3
1	0	0	0	0	0	0	0	3	0	0
4	0	0	0	0	0	0	1	4	0	0
0	0	0	0	0	0	0	0	1	0	0
3	0	0	0	0	0	0	0	8	6	0
1	0	0	0	0	0	0	0	3	1	4
1	0	0	0	0	0	0	0	1	3	8
1	0	0	0	0	0	0	0	1	0	2
0	0	0	0	0	0	0	0	5	0	1
3	0	0	0	0	0	0	0	3	4	7
3	0	0	0	0	0	0	0	3	0	3
8	0	1	0	0	0	0	0	5	0	1
2	0	0	0	0	0	0	0	9	0	7
2	0	0	0	0	0	0	0	2	1	1

0	0	0	0	0	0	0	0	3	0	2
1	0	0	0	0	0	0	1	11	0	3
1	0	0	0	0	0	0	1	10	0	2
0	0	0	0	0	0	0	0	9	0	0
1	0	0	0	0	0	0	0	4	0	2
0	0	0	0	0	0	0	0	10	0	3
0	0	0	0	0	0	0	0	9	0	0
1	0	0	0	0	0	0	0	7	0	1
0	0	0	0	0	0	0	0	8	0	1
1	0	0	0	0	0	0	0	9	0	2
2	0	0	0	0	0	0	2	2	0	3
0	0	0	0	0	0	0	0	2	0	0
1	0	0	0	0	0	0	4	19	0	0
0	0	0	0	0	0	0	1	5	0	3
0	0	0	0	0	0	0	0	9	0	2
0	0	0	0	0	0	0	0	11	0	1
0	0	0	0	0	0	0	0	7	0	2
0	0	0	0	0	0	0	0	6	0	0
0	0	0	0	0	0	0	0	8	0	0
0	0	0	0	0	0	0	3	9	0	0
0	0	0	0	0	0	0	1	13	0	2
0	0	0	0	0	0	0	1	13	0	1
0	0	0	0	0	0	0	0	1	0	0
0	0	0	0	0	0	0	0	3	0	0
0	0	0	0	0	0	0	1	7	0	0
0	0	0	0	0	0	0	0	6	0	0
0	0	1	0	0	0	0	0	9	0	1
0	0	0	0	0	0	0	0	8	0	0
1	0	0	0	0	0	0	1	5	0	0

0	0	0	0	0	0	0	4	7	0	2
0	0	3	0	0	0	0	2	7	0	0
0	0	2	0	0	0	0	1	8	0	1
0	0	2	0	0	0	0	0	8	0	1
0	0	0	0	0	0	0	0	7	0	0
0	0	0	0	0	0	0	2	7	0	1
0	0	2	0	0	0	0	0	13	0	1
0	0	1	0	0	0	0	0	3	0	1
0	0	0	0	0	0	0	0	6	0	0
0	0	0	0	0	0	0	0	5	0	0
0	0	0	0	0	0	0	0	6	0	0
0	0	0	0	0	0	0	0	5	0	0
0	0	0	0	0	0	0	0	4	0	0
0	0	1	0	0	0	0	0	3	0	0
0	0	2	0	0	0	0	0	0	0	0
0	0	44	0	0	0	0	0	5	0	0
0	0	3	0	0	0	0	0	3	0	0
0	0	0	0	0	0	0	0	3	0	0
0	0	1	0	0	0	0	1	1	0	0
0	0	48	0	0	0	0	0	0	0	0
0	0	3	0	0	0	0	0	2	0	0
1	0	13	0	0	0	0	0	2	0	0
0	0	8	0	0	0	0	0	0	0	0

lumbri	lunsma	lysian	maclil	macste	magdak	methal	micten	mussen	mysiad	nebali
0	0	0	17	0	2	0	0	0	0	0
0	0	0	10	0	2	0	0	0	0	0
0	0	0	5	0	0	0	0	0	0	0
0	0	0	9	0	1	0	0	0	0	0

0	0	0	16	0	1	1	0	0	0	0
0	0	0	9	0	6	0	0	0	0	0
0	0	0	15	0	1	0	0	0	0	0
0	0	0	18	0	0	0	0	0	0	0
0	0	0	15	0	1	2	0	0	0	0
0	0	0	7	0	0	0	0	0	0	0
0	0	0	3	1	5	0	0	0	0	0
0	0	0	6	1	6	0	0	0	0	0
0	0	0	7	4	9	0	0	0	0	0
0	0	0	5	5	6	0	0	0	0	0
0	0	0	4	2	13	0	0	0	0	0
0	0	0	6	2	4	0	0	0	0	0
0	0	0	12	1	7	0	0	0	0	0
0	0	0	6	8	7	0	0	0	0	0
0	0	0	0	4	4	0	0	0	0	0
0	0	0	5	4	8	0	0	0	0	0
0	0	0	13	0	0	1	0	0	0	0
0	0	0	7	1	0	0	0	0	0	0
0	0	0	6	1	2	0	0	0	0	0
0	0	0	8	0	1	0	0	0	0	0
0	0	0	6	1	2	0	0	0	0	0
0	0	0	11	0	0	0	0	0	0	0
0	0	0	11	0	1	0	0	0	0	0
0	0	0	7	0	1	0	0	0	0	0
0	0	0	8	0	2	0	0	0	0	0
0	0	0	8	1	6	0	0	0	1	0
0	0	0	15	0	2	0	0	0	0	0
0	0	0	6	3	0	0	0	0	0	0
0	0	0	6	1	4	0	0	0	0	0

0	0	0	8	0	2	0	0	0	0	0
0	0	0	6	1	0	0	0	0	0	0
0	0	0	14	0	2	0	0	0	0	0
0	0	0	9	0	0	0	0	0	0	0
0	0	0	12	0	1	0	0	0	0	0
0	0	0	4	2	3	0	0	1	0	0
0	0	0	7	0	0	0	0	0	0	0
0	1	0	10	1	2	0	0	0	0	0
0	0	0	4	1	0	0	0	0	0	0
0	0	0	4	1	1	0	0	0	0	0
0	0	0	2	0	0	0	0	0	0	0
0	0	0	6	0	2	0	0	0	0	0
0	0	0	4	0	1	0	0	0	0	0
0	0	0	10	0	3	0	0	0	0	0
0	0	0	3	0	2	0	0	0	0	0
0	0	0	4	0	2	0	0	0	0	0
0	0	0	9	1	0	0	0	0	0	0
0	0	0	5	2	1	0	0	0	0	0
0	0	0	8	0	0	0	0	0	0	1
0	0	0	5	0	2	0	0	0	0	0
0	0	0	7	0	1	0	0	0	0	0
0	0	0	6	0	2	0	0	0	0	0
0	0	0	8	0	0	0	0	0	0	0
0	0	0	6	0	1	0	0	0	0	0
0	0	0	6	0	1	0	0	0	0	0
0	0	0	5	0	0	0	0	0	0	0
0	0	0	3	0	0	0	0	0	0	0
0	0	0	1	0	1	0	0	0	0	0
0	0	0	12	0	3	0	0	0	1	0

0	0	0	5	0	0	0	0	0	0	0
0	0	0	1	0	0	0	0	0	0	0
0	0	0	0	0	0	0	0	0	0	0
0	0	0	2	0	1	0	0	0	0	0
0	0	0	0	0	0	0	0	0	0	0
0	0	0	1	0	0	0	0	0	1	0
0	0	0	7	0	0	0	0	0	1	0
0	0	0	8	0	0	0	0	0	0	0
0	0	0	0	0	0	0	0	0	0	0
0	0	0	9	4	4	0	0	0	0	0
0	0	0	3	0	0	0	0	0	0	0
0	0	0	1	1	2	0	0	0	0	0
0	0	0	8	0	1	0	0	0	0	0
0	0	0	6	0	0	0	0	0	0	0
0	0	0	3	0	0	0	0	0	0	0
0	0	0	6	1	5	0	0	0	0	0
0	0	0	7	1	4	0	0	0	0	0
0	0	0	3	1	2	0	0	0	0	0
0	0	0	10	0	0	0	0	0	0	0
0	0	0	3	5	5	0	0	0	0	0
0	0	0	4	0	1	0	0	0	0	0
0	0	0	4	0	10	0	0	0	0	0
0	0	0	2	0	6	0	0	0	0	0
0	0	0	4	5	3	0	0	0	0	0
0	0	0	6	3	4	0	0	0	0	0
0	0	0	5	1	5	0	0	0	0	0
0	0	0	2	2	6	0	0	0	0	0
0	0	0	4	4	2	0	0	0	0	0
0	0	0	2	5	5	0	0	0	0	0

1	0	0	2	2	13	0	0	0	0	0
0	0	0	5	2	9	0	0	0	0	0
0	0	0	3	5	3	0	0	0	0	0
0	0	0	5	5	4	0	0	0	0	0
0	0	0	6	1	7	0	0	0	0	0
0	0	0	1	1	6	0	0	0	0	0
0	0	0	7	0	8	0	0	0	0	0
0	0	0	2	0	3	0	0	0	0	0
0	0	0	3	1	5	0	0	0	0	0
0	0	0	1	5	3	0	0	0	0	0
0	0	0	2	5	8	0	0	0	0	0
0	0	0	3	3	1	0	0	0	0	0
0	0	0	2	2	6	0	0	0	0	0
0	0	0	4	5	7	0	0	0	0	0
1	0	0	4	1	4	0	0	0	0	0
0	0	0	6	3	5	0	0	0	1	0
0	0	0	3	4	7	0	0	0	0	0
0	0	0	7	4	4	0	0	0	0	0
0	0	0	9	6	7	0	0	0	0	0
0	0	0	3	6	0	0	0	0	0	0
0	0	0	6	9	0	0	0	0	0	0
0	0	0	5	2	0	0	0	0	0	0
0	0	0	5	3	0	0	0	0	0	0
0	0	0	9	8	0	0	0	0	0	1
0	0	0	6	5	0	0	0	0	0	0
0	0	0	6	5	0	0	0	0	0	0
0	0	0	6	4	0	0	0	0	0	0
0	0	0	2	2	0	0	0	0	0	0
0	0	0	4	3	0	0	0	0	0	0

0	0	0	4	4	9	0	0	0	0	0
0	0	0	4	6	5	0	0	0	0	0
0	0	0	4	7	4	0	0	0	0	0
0	0	0	5	12	8	0	0	0	1	0
0	0	0	2	3	9	0	0	0	0	0
0	0	0	2	6	8	0	0	0	0	0
0	0	0	7	7	4	0	0	0	0	0
0	0	0	6	2	7	0	0	0	0	0
0	0	0	6	3	2	0	0	0	0	0
0	0	0	6	4	3	0	0	0	0	0
0	0	0	5	4	5	0	0	0	0	0
0	0	0	3	4	3	0	0	0	0	0
0	0	0	6	4	3	0	0	0	0	0
0	0	0	4	6	2	0	0	0	0	0
0	0	0	4	12	3	0	0	0	0	0
0	0	0	4	1	4	0	0	0	0	0
0	0	0	3	0	4	0	0	0	0	0
0	0	0	3	3	2	0	0	0	0	0
0	0	0	5	0	6	0	0	0	0	0
0	0	0	5	0	2	0	0	0	0	0
0	0	0	5	1	2	0	0	0	0	0
0	0	0	5	0	1	0	0	0	0	0
0	0	0	4	4	6	0	0	0	0	0
0	0	0	6	3	5	0	0	0	0	0
0	0	0	6	3	5	0	0	0	0	0
0	0	0	1	2	5	0	0	0	0	0
0	0	0	0	0	1	0	0	0	0	0
0	0	0	3	2	5	0	0	0	0	0
0	0	0	3	1	2	0	0	0	0	0

0	0	0	3	0	5	0	0	0	0	0
0	0	0	2	0	6	0	0	0	0	0
0	0	0	7	2	1	0	0	0	0	0
0	0	0	5	0	0	0	0	0	0	0
0	0	0	2	1	5	0	0	0	0	0
0	0	0	3	2	3	0	0	0	0	0
0	0	0	7	0	7	0	0	0	0	0
0	0	0	8	0	5	0	0	0	0	0
0	0	0	8	0	2	0	0	0	0	0
0	0	0	7	1	2	0	0	0	0	0
0	0	0	7	0	6	0	0	0	0	0
0	0	0	9	0	5	0	0	0	0	0
0	0	0	3	2	1	0	0	0	0	0
0	0	0	4	0	2	0	0	0	0	0
0	0	0	5	0	1	0	1	0	0	0
0	0	0	11	0	1	0	1	0	0	0
0	0	0	3	2	0	0	1	0	0	0
0	0	0	1	0	0	0	0	0	0	0
0	0	0	4	0	4	0	0	0	0	0
0	0	0	3	0	0	0	0	0	0	0
0	0	0	7	0	1	0	0	0	0	0
0	0	0	6	1	0	0	0	0	0	0
0	0	0	0	2	0	0	0	0	0	0
0	0	0	6	0	0	0	0	0	0	0
0	0	0	4	1	0	0	0	0	0	0
0	0	0	4	0	1	0	0	0	0	0
0	0	0	3	0	0	0	1	0	0	0
0	0	2	5	0	0	0	0	0	0	0
0	0	0	7	0	0	0	0	0	0	0

0	0	0	0	1	0	0	0	0	0	0
0	0	0	0	0	0	0	0	0	0	0
0	0	0	0	0	0	0	0	0	0	0
0	0	0	11	0	0	0	0	0	0	0
0	0	0	0	0	0	0	0	0	0	0
0	0	0	1	0	0	0	0	0	0	0
0	0	0	2	0	0	0	0	0	0	0
0	0	0	7	0	0	0	0	0	0	0
0	0	0	3	0	1	0	0	0	0	0
0	0	0	1	0	0	0	0	0	0	0
0	0	0	4	0	1	0	0	0	0	0
0	0	0	10	0	0	0	0	0	0	0
0	0	0	11	0	1	0	0	0	0	0
0	0	0	18	0	1	0	0	0	0	0
0	0	0	7	0	0	0	0	0	0	0
0	0	0	10	0	0	0	0	0	0	0
0	0	0	10	0	1	0	0	0	0	0
0	0	0	5	0	1	0	0	0	0	0
0	0	0	6	0	0	0	0	0	0	0
0	0	0	5	0	1	0	0	0	0	0
0	0	0	7	0	0	0	0	0	0	0
0	0	0	10	0	2	0	0	0	0	0
0	0	0	3	0	0	0	0	0	0	0
0	0	0	10	0	0	0	0	0	0	0
0	0	0	10	1	0	0	0	0	0	0
0	0	0	6	0	0	0	0	0	0	0
0	0	0	10	0	1	0	0	0	0	0
0	0	0	3	0	0	0	0	0	0	0
0	0	0	6	0	0	0	0	0	0	0

0	0	0	5	0	1	0	0	0	0	0
0	0	0	6	0	1	0	0	0	0	0
0	0	0	9	0	2	0	0	0	0	0
0	0	0	7	1	0	0	0	0	0	0
0	0	0	7	0	0	0	0	0	0	0
0	0	0	13	0	0	0	0	0	0	0
0	0	0	10	0	0	0	0	0	0	0
0	0	0	7	0	1	0	0	0	0	0
0	0	0	13	0	0	0	0	0	0	0
0	0	0	4	0	0	0	0	0	0	0
0	0	0	9	0	1	0	0	0	0	2
0	0	0	11	0	1	0	1	0	0	0
0	0	0	9	0	0	0	1	0	0	0
0	0	0	14	0	0	0	0	0	0	0
0	0	0	1	3	0	0	0	0	0	0
0	0	0	8	0	1	0	0	0	0	0
0	0	0	6	0	0	0	0	0	0	0
0	0	0	7	0	1	0	0	0	0	0
0	0	0	5	0	2	0	0	0	0	0
0	0	0	5	0	0	0	0	0	0	0
0	0	0	2	1	0	0	1	0	0	0
0	0	0	4	0	0	0	0	0	0	0
0	0	0	6	0	0	0	1	0	0	0
0	0	0	1	0	0	0	0	1	0	0
0	0	0	1	0	3	0	0	0	0	0
0	0	0	2	1	0	0	0	0	0	0
0	0	0	4	1	1	0	0	0	0	0
0	0	0	3	0	3	0	0	0	0	0
0	0	0	3	4	2	0	0	0	0	0

0	0	0	3	2	1	0	1	0	0	0
0	0	0	4	0	0	0	0	0	0	0
0	0	0	4	2	2	0	0	0	0	0
0	0	0	1	1	2	0	0	0	0	0
0	0	0	5	0	1	0	0	0	0	0
0	0	0	1	1	0	0	0	0	0	0
0	0	0	4	1	4	0	0	0	0	0
0	0	0	5	1	2	0	0	0	0	0
0	0	0	4	0	1	0	0	0	0	0
0	0	0	1	0	3	0	0	0	0	0
0	0	0	6	1	2	0	0	0	0	0
0	0	0	3	2	0	0	0	0	0	0
0	0	0	3	1	2	0	0	0	0	0
0	0	0	1	0	1	0	0	0	0	0
0	0	0	3	2	6	0	0	0	0	0
0	0	0	2	0	2	0	0	0	0	0
0	0	0	5	3	3	0	0	0	0	0
0	0	0	5	1	1	0	0	0	0	0
0	0	0	3	0	3	0	0	0	0	0
0	0	0	1	0	3	0	0	0	0	0
0	0	0	2	2	2	0	0	0	0	0
0	0	0	1	4	2	0	0	0	0	0
0	0	0	4	1	1	0	0	0	0	0
0	0	0	7	0	7	0	0	0	0	0
0	0	0	2	0	5	0	0	0	0	0
0	0	0	4	2	3	0	0	0	0	0
0	0	0	2	0	4	0	0	0	0	0
0	0	0	4	0	2	0	0	0	0	0
0	0	0	3	0	5	0	0	0	0	0

0	0	0	0	0	2	0	0	0	0	0
0	0	0	6	4	6	0	0	0	0	0
0	0	0	6	0	7	0	0	0	0	0
0	0	0	3	3	6	0	0	0	0	0
0	0	0	4	0	4	0	0	0	0	0
0	0	0	4	5	5	0	0	0	0	0
0	0	0	3	13	10	0	0	0	0	0
0	0	0	5	1	8	0	0	0	0	0
0	0	0	3	4	4	0	0	0	0	0
0	0	0	9	9	5	0	0	0	0	0
0	0	0	7	1	3	0	0	0	0	0
0	0	0	5	6	2	0	0	0	0	0
0	0	0	2	14	8	0	0	0	0	0
0	0	0	5	8	1	0	0	0	0	0
0	0	0	5	9	6	0	0	0	0	0
0	0	0	4	7	7	0	0	0	0	0
0	0	0	2	7	5	0	0	0	0	0
0	0	0	2	10	4	0	0	0	0	0
0	0	0	3	6	1	0	0	0	0	0
0	0	0	5	7	8	0	0	0	0	0
0	0	0	5	14	6	0	0	0	0	0
0	0	0	1	0	1	0	0	0	0	0
0	0	0	2	11	15	0	0	0	0	0
0	0	0	2	5	8	0	0	0	0	0
0	0	0	3	5	7	0	0	0	0	0
0	0	0	2	6	0	0	0	0	0	0
0	0	0	2	5	7	0	0	0	0	0
0	0	0	6	9	5	0	0	0	0	0
0	0	0	2	10	5	0	0	0	0	0

0	0	0	4	5	4	0	0	0	0	0
0	0	0	2	9	5	0	0	0	0	0
0	0	0	4	7	9	0	0	0	0	0
0	0	0	1	7	12	0	0	0	0	0
1	0	0	12	1	4	0	0	0	0	0
0	0	0	2	10	17	0	0	0	0	0
0	0	0	0	1	0	0	0	0	0	0
0	0	0	4	4	4	0	0	0	0	0
0	0	0	1	0	0	0	0	0	0	0
0	0	0	1	5	2	0	0	0	0	0
0	0	0	3	7	3	0	4	0	0	0
0	0	0	8	5	8	0	0	0	0	0
0	0	0	1	6	4	0	0	0	0	0
0	0	0	2	6	3	0	0	0	0	0
0	0	0	2	1	6	0	0	0	0	0
0	0	0	5	9	2	0	0	0	0	0
0	0	0	4	4	5	0	0	0	0	0
0	0	0	4	7	5	0	0	0	0	1
0	0	0	3	9	4	0	0	0	0	0
0	0	0	8	5	16	0	0	0	0	0
0	0	0	2	6	3	0	0	0	0	0
0	0	0	0	10	6	0	0	0	0	0
0	0	0	4	9	2	0	0	0	0	0
0	0	0	5	10	4	0	0	0	0	0
0	0	0	3	8	5	0	0	0	0	0
0	0	0	4	15	2	0	0	0	0	0
0	0	0	2	5	4	0	0	0	0	0
0	0	0	2	9	4	0	0	0	0	0
0	0	0	2	7	3	0	0	0	1	0

0	0	0	3	7	1	0	0	0	0	0
1	0	0	2	0	3	0	0	0	0	0
0	0	0	1	2	1	0	0	0	0	0
0	0	0	2	2	2	0	0	0	2	0
0	0	0	5	2	4	0	0	0	0	0
0	0	0	1	5	1	0	0	0	0	0
0	0	0	1	3	0	0	0	0	0	0
0	0	0	1	3	3	0	0	0	0	0
0	0	0	6	1	1	0	0	0	0	0
0	0	0	5	0	0	0	0	0	0	0
0	0	0	2	8	2	0	0	0	0	0
0	0	0	2	4	1	0	0	0	0	0
0	0	0	3	1	1	0	0	0	0	0
0	0	0	1	0	2	0	0	0	0	0
0	0	0	2	2	1	0	0	0	0	0
0	0	0	2	2	1	0	0	0	0	0
0	0	0	5	1	1	0	0	0	0	0
0	0	0	6	3	2	0	0	0	0	0
0	0	0	6	2	0	0	0	0	0	0
0	0	0	5	2	3	0	0	0	0	0
0	0	0	5	4	4	0	0	0	0	0
0	0	0	5	0	0	0	0	0	0	0
0	0	0	6	0	0	0	0	0	0	0
0	0	0	6	0	0	0	0	0	0	0
0	0	0	9	0	0	0	0	0	0	0
0	0	0	4	0	0	0	0	0	0	0
0	0	0	3	0	2	0	0	0	0	0
0	0	0	4	3	0	0	0	0	0	0
0	0	0	2	0	2	0	0	0	0	3

0	0	0	4	1	1	0	0	0	0	0
0	0	0	5	0	1	0	0	0	0	0
0	0	0	3	3	0	0	0	0	0	0
0	0	0	5	1	0	0	0	0	0	0
0	0	0	8	1	0	0	0	0	0	0
0	0	0	3	0	0	0	0	0	0	0
0	0	0	7	0	1	0	0	0	0	2
0	0	0	2	0	0	0	0	0	0	0
0	0	0	6	0	2	0	0	0	0	0
0	0	0	7	0	0	0	0	0	0	0
0	0	0	8	0	3	0	0	0	0	0
0	0	0	4	1	2	0	0	0	0	0
0	0	0	3	0	0	0	0	0	0	0
0	0	0	2	1	2	1	0	0	0	0
0	0	0	4	1	0	0	0	0	0	0
0	0	0	1	0	1	0	0	0	0	0
0	0	0	2	0	1	0	0	0	0	0
0	0	0	2	1	0	0	0	0	0	0
0	0	0	3	1	1	0	0	0	0	0
0	0	0	5	2	1	0	0	0	0	0
0	0	0	4	1	1	0	0	0	0	0
0	0	0	5	0	2	0	0	0	0	0
0	0	0	3	0	0	0	0	0	0	0
0	0	0	6	1	0	0	0	0	0	0
0	0	0	5	1	0	0	0	0	0	0
0	0	0	2	1	0	0	0	0	0	3
0	0	0	3	0	2	0	0	0	0	0
0	0	0	7	1	0	0	0	0	0	0
0	0	0	2	0	0	0	0	0	0	0

0	0	1	1	0	0	0	4	0	0	0
0	0	0	4	1	1	0	0	0	0	0
0	0	0	2	0	0	0	0	0	0	0
0	0	0	2	0	0	0	1	0	0	0
0	0	0	1	0	0	0	4	0	0	0
0	0	0	3	1	0	0	1	0	0	0
0	0	0	7	0	1	0	0	0	0	0
0	0	0	1	0	0	0	0	0	0	0
0	0	0	0	0	0	0	0	0	0	0
0	0	0	5	6	2	0	0	0	0	0
0	0	0	4	0	0	0	0	0	0	0
0	0	0	0	0	1	0	0	0	0	0
0	0	0	0	0	0	0	0	0	0	0
0	0	0	12	0	0	0	0	0	0	0
0	0	0	7	0	1	0	3	0	0	0
0	0	0	7	0	0	0	0	0	0	0
0	0	0	6	0	0	0	0	0	0	0
0	0	0	4	0	0	0	0	0	0	0
0	0	0	7	0	0	0	0	0	0	0

orbpap	owepet	palaem	paramo	parexc	parlyr	parnou	patiri	perval	phiaus	phoxoc
0	0	0	0	0	0	0	0	0	0	1
0	0	0	0	0	0	0	0	9	0	5
0	0	0	0	0	0	0	0	1	0	0
0	0	0	0	0	0	0	0	12	0	0
0	0	1	0	1	0	0	0	4	0	6
0	0	1	0	0	0	1	0	7	0	2
0	0	0	0	0	0	0	0	11	0	1
0	0	0	0	0	0	0	0	9	0	1

0	0	0	0	0	0	1	0	9	0	2
0	0	0	0	0	0	0	0	13	0	1
0	0	0	0	0	1	0	0	1	0	0
0	0	0	0	0	0	0	0	4	0	8
0	0	0	0	0	0	0	1	0	0	2
0	0	0	0	0	0	0	0	4	0	5
0	0	0	0	0	3	0	0	10	0	2
0	0	0	0	0	0	0	0	2	0	20
1	0	0	0	0	0	1	0	3	0	13
0	0	0	0	0	0	1	0	2	0	0
1	0	0	0	0	1	0	0	4	0	1
0	0	0	0	3	1	0	0	0	0	5
0	0	1	0	0	0	0	0	5	0	2
0	0	0	0	1	0	0	0	7	0	8
0	0	0	0	0	0	1	0	6	0	0
0	0	0	0	1	0	0	0	10	0	4
0	0	1	0	0	0	0	0	3	0	6
0	0	1	0	2	0	0	0	3	0	4
0	0	0	0	0	0	2	0	9	0	9
0	0	0	0	0	0	0	0	13	0	4
0	0	0	0	0	0	0	0	10	0	1
0	0	0	0	0	0	1	0	9	0	7
0	0	0	0	0	1	0	0	0	0	1
0	0	1	0	1	0	0	0	3	0	0
0	0	0	0	0	0	1	0	1	0	0
0	0	0	0	0	1	0	0	14	0	2
0	0	0	0	0	0	1	0	0	0	1
0	0	0	0	0	0	0	0	1	0	7
0	0	0	0	0	0	0	0	7	0	6

0	0	0	0	0	0	0	0	6	0	2
0	0	0	0	1	0	0	0	1	0	1
0	0	0	0	0	0	0	0	6	0	11
0	0	0	0	0	0	0	0	3	0	1
0	0	0	0	0	0	0	0	0	0	4
0	0	0	0	0	0	0	0	1	0	2
0	0	0	0	0	0	4	0	2	0	5
0	0	0	0	0	0	0	0	3	0	1
0	0	0	0	0	0	0	0	1	0	0
0	0	0	0	0	0	4	0	0	0	8
0	0	0	0	0	0	0	0	0	0	8
0	0	0	0	1	0	0	0	1	0	0
0	0	0	0	0	0	3	0	1	0	6
0	0	0	0	0	0	1	0	2	0	1
0	0	0	0	0	0	0	0	0	0	0
0	0	0	0	0	0	0	0	0	0	1
0	0	0	0	0	0	0	0	1	0	0
0	0	0	0	0	0	0	0	0	0	0
1	0	0	0	0	0	0	0	2	0	0
0	0	0	0	0	0	0	0	0	0	1
0	0	0	0	0	0	0	0	0	0	0
0	0	0	0	0	0	0	0	0	0	1
0	0	0	0	0	0	0	0	4	0	2
0	0	0	0	0	0	0	0	1	0	2
0	0	0	0	0	0	1	0	2	0	4
0	0	0	0	0	0	0	0	0	0	5
0	0	3	0	0	0	0	0	1	0	7
0	0	0	0	0	0	9	0	1	0	17
0	0	0	0	0	0	5	0	1	0	1

0	0	0	0	0	0	2	0	1	0	2
0	0	1	0	0	0	1	0	1	0	2
0	0	1	0	0	0	1	0	11	0	7
0	0	0	0	0	0	0	0	4	0	7
0	0	0	0	1	0	3	0	0	0	5
0	0	0	0	0	0	2	0	3	0	10
0	0	0	0	0	0	1	0	1	0	9
0	0	0	0	0	0	0	0	0	0	0
0	0	0	0	1	0	0	0	1	0	4
0	0	0	0	0	0	2	0	0	0	2
0	0	0	0	0	0	7	0	0	0	22
0	0	2	0	0	0	2	0	1	0	6
0	0	0	0	1	0	0	0	1	0	0
0	0	0	0	1	0	1	0	6	0	6
1	0	0	0	0	0	1	0	1	0	1
0	0	0	0	0	0	1	0	2	0	10
0	0	0	0	0	0	0	0	1	0	0
0	0	0	0	1	0	1	0	3	0	13
0	0	0	0	0	0	2	0	1	0	4
0	0	0	0	0	0	1	0	0	0	1
0	0	0	0	0	0	0	0	1	0	2
0	0	0	0	0	0	0	0	1	0	3
0	0	0	0	0	0	0	0	3	0	5
0	0	0	0	0	0	0	0	0	0	4
0	0	0	0	0	0	0	0	1	0	0
1	0	0	0	0	1	0	0	0	0	3
0	0	0	0	0	0	1	0	0	0	5
0	0	0	0	1	0	0	0	2	0	8
0	0	0	0	0	0	0	0	8	0	1

0	0	0	0	0	1	0	1	2	0	3
0	0	0	0	0	1	0	0	0	0	1
0	0	0	0	1	1	2	0	0	0	12
0	0	0	0	2	0	0	0	4	0	7
0	0	0	0	0	0	0	0	1	0	2
0	0	0	0	0	0	0	0	3	0	0
0	0	0	0	1	0	0	0	1	0	0
0	3	0	0	0	0	0	0	2	0	1
0	1	0	0	0	1	0	0	1	0	2
1	0	0	0	0	3	0	0	4	0	2
0	0	0	0	0	0	0	0	5	0	5
0	0	0	0	0	2	0	0	1	0	3
0	0	0	0	0	0	0	1	3	0	8
0	1	0	0	0	0	0	2	6	0	6
0	0	0	0	2	0	0	0	1	0	1
0	0	0	0	0	0	0	0	3	0	0
0	0	0	0	0	0	0	0	5	0	3
0	0	0	0	1	1	0	0	0	0	2
0	0	0	0	2	0	0	2	8	0	10
0	1	0	0	1	2	0	0	0	0	0
0	0	0	0	0	0	0	0	3	0	0
0	0	0	0	0	0	0	0	9	0	2
0	0	0	0	0	0	0	0	5	0	4
0	0	0	1	0	0	0	0	1	0	6
0	0	0	0	0	0	1	0	0	0	6
1	0	0	0	1	0	0	0	7	0	7
0	0	0	0	0	0	3	0	6	0	9
0	0	0	0	0	0	0	0	6	0	4
0	0	0	0	1	0	0	0	3	0	1

0	0	0	0	0	0	0	0	3	0	1
0	0	0	0	0	0	0	0	1	0	2
0	0	0	0	0	0	0	0	3	0	0
0	0	0	0	2	0	0	0	3	0	5
0	0	0	0	0	0	0	0	4	0	3
0	0	0	0	0	0	0	0	2	0	5
0	0	1	0	0	0	1	0	3	0	5
0	0	0	0	0	0	0	0	2	0	1
0	0	0	0	0	0	0	0	4	0	3
0	0	0	0	0	0	0	0	2	0	1
0	0	0	0	0	0	0	0	2	0	5
0	0	0	0	0	0	0	0	2	0	1
0	0	0	0	0	0	0	0	2	0	8
0	0	0	0	0	0	1	0	7	0	11
0	0	0	0	0	0	0	0	6	0	1
0	0	0	0	0	0	0	0	0	0	0
0	0	0	0	0	0	0	0	0	0	2
0	0	0	0	0	0	1	0	1	0	2
0	0	0	0	0	0	0	0	0	0	0
0	0	0	0	0	0	0	0	0	0	1
0	0	0	0	0	0	0	0	6	0	9
0	0	0	0	0	0	0	0	8	0	7
0	0	0	0	0	0	0	0	0	0	5
0	0	0	0	0	0	0	0	5	0	2
0	0	0	0	0	0	0	0	0	0	0
0	0	0	0	0	0	0	0	2	0	0
0	0	0	0	1	0	0	0	1	0	0
0	0	0	0	0	0	2	0	4	0	2
0	0	0	0	0	0	1	0	3	0	0

0	0	0	0	0	0	0	0	1	0	0
0	0	0	0	0	0	0	0	3	0	12
0	0	0	0	0	0	0	0	3	0	4
0	0	0	0	0	0	0	0	2	0	7
0	0	0	0	0	0	0	0	0	0	4
0	0	0	0	0	0	0	0	2	0	0
0	0	0	0	1	0	0	0	4	0	3
0	0	0	0	0	0	0	0	0	0	9
0	0	0	0	0	0	0	0	3	0	2
0	0	0	0	0	0	0	0	2	0	1
0	0	0	0	0	0	0	0	0	0	1
0	0	0	0	0	0	0	0	3	0	5
0	0	0	0	0	0	0	0	0	0	1
0	0	0	0	0	0	0	0	0	0	0
0	0	0	0	0	0	0	0	3	0	6
0	0	0	0	0	0	0	0	5	0	2
0	0	0	0	5	0	1	0	1	0	0
0	0	0	0	0	0	2	0	2	0	9
0	0	0	0	1	0	0	0	9	0	3
0	0	0	0	0	0	1	0	1	0	6
0	0	0	0	0	0	1	0	6	0	7
0	0	0	0	1	0	3	0	1	0	6
0	0	0	0	0	0	0	0	2	0	4
0	0	0	0	4	0	1	0	0	0	7
0	0	0	0	0	0	1	0	11	0	9
0	0	0	0	0	0	0	0	2	0	7
0	0	0	0	0	0	1	0	6	0	1
0	0	0	0	0	0	0	0	7	0	6
0	0	0	0	0	0	0	0	5	0	0

0	0	0	0	0	0	0	0	15	0	8
0	0	0	0	1	0	0	0	12	0	11
0	0	0	0	0	0	1	0	4	0	3
0	0	0	0	0	0	0	0	12	0	2
0	0	0	0	0	0	0	0	9	0	3
0	0	0	0	0	0	0	0	2	0	7
0	0	0	0	0	0	0	0	0	0	3
0	0	0	0	1	0	1	0	2	0	0
0	0	0	0	1	0	0	0	1	0	7
0	0	0	0	0	0	0	0	18	0	8
0	0	0	0	2	0	0	0	0	0	1
0	0	0	0	0	0	0	0	0	0	2
0	0	0	0	0	0	0	0	0	0	0
0	0	0	0	2	0	0	0	6	0	6
0	0	0	0	1	0	0	0	6	0	2
0	0	0	0	0	0	0	0	10	0	1
0	0	0	0	1	0	0	0	10	0	1
0	0	0	0	0	0	0	0	8	0	1
0	0	0	0	0	0	0	0	2	0	0
0	0	0	0	0	0	0	0	1	0	0
0	0	0	0	0	0	0	0	3	0	2
0	0	0	0	0	0	1	0	1	0	4
0	0	0	0	0	0	0	0	1	0	0
0	0	0	0	0	0	0	0	4	0	3
0	0	0	0	0	0	0	0	12	0	3
0	0	0	0	0	0	0	0	1	0	0
0	0	0	0	0	0	0	0	5	0	1
0	0	0	0	0	0	0	0	7	0	0
0	0	0	0	0	0	0	0	14	0	2

0	0	0	0	0	0	0	0	9	0	6
0	0	0	0	0	0	0	0	5	0	2
0	0	0	0	0	0	0	0	0	0	1
0	0	0	0	1	0	0	0	11	0	6
0	0	0	0	0	0	0	0	2	0	1
0	0	0	0	0	0	0	0	4	0	4
0	0	0	0	0	0	0	0	0	0	1
0	0	0	0	0	0	0	0	13	0	5
0	0	0	0	0	0	0	0	7	0	13
0	0	0	0	0	0	0	0	3	0	4
0	0	0	0	0	0	1	0	1	0	8
0	0	0	0	0	0	0	0	3	0	7
0	0	0	0	1	0	1	0	5	0	6
0	0	0	0	0	0	0	0	11	0	10
0	0	0	0	0	0	0	0	2	0	5
0	0	0	0	0	0	0	0	4	0	4
0	0	0	0	0	0	0	0	2	0	0
0	0	0	0	0	0	0	0	6	0	3
0	0	0	0	0	0	0	0	3	0	2
0	0	0	0	0	0	0	0	1	0	1
0	0	0	0	0	1	0	0	4	0	2
0	0	0	0	0	0	0	0	2	0	2
0	0	0	0	0	0	4	0	8	0	15
0	0	0	0	0	0	1	0	2	0	3
1	0	0	0	0	0	0	0	0	0	6
0	0	0	0	0	0	3	0	2	0	10
0	0	0	0	0	0	1	0	2	0	6
0	0	0	0	0	0	0	0	6	0	0
0	0	0	0	0	0	0	0	2	0	6

0	0	0	0	0	0	1	0	4	0	10
0	0	0	0	0	0	0	0	4	0	2
0	0	0	0	0	0	1	0	2	0	3
0	0	0	0	0	0	1	0	5	0	5
0	0	0	0	0	0	0	0	4	0	5
0	0	0	0	0	0	0	0	3	0	10
0	0	0	0	0	0	1	0	9	0	3
0	0	0	0	0	0	0	0	6	0	6
0	0	0	0	0	0	0	0	4	0	4
0	0	0	0	0	0	0	0	2	0	8
0	0	0	0	0	0	0	0	11	0	7
0	0	0	0	0	0	0	0	5	0	1
0	0	0	0	0	0	0	0	6	0	11
0	0	0	0	0	0	0	0	11	0	1
0	0	0	0	0	0	0	0	3	0	0
0	0	0	0	0	0	0	0	8	0	1
0	0	0	0	0	0	0	0	5	0	3
0	0	0	0	0	0	0	0	4	0	3
0	0	0	0	0	0	0	0	5	0	3
0	0	0	0	0	0	0	0	1	0	0
0	0	0	0	0	0	0	0	5	0	3
0	0	0	0	0	0	0	0	2	0	1
0	0	0	0	0	1	1	0	4	0	1
0	0	0	0	0	0	0	0	7	0	6
0	0	0	0	0	0	0	0	5	0	4
0	0	0	0	0	0	0	0	3	0	0
0	0	0	0	0	0	0	0	7	0	1
0	0	0	0	0	0	0	0	1	0	1
0	0	0	0	0	0	0	0	6	0	3

0	0	0	0	0	0	0	0	15	0	6
0	0	0	0	0	0	0	0	4	0	7
0	0	0	0	0	0	0	0	5	0	2
0	0	0	0	0	0	0	0	7	0	7
0	0	0	0	0	0	0	0	7	0	0
0	0	0	0	0	0	1	0	9	0	2
0	0	0	0	0	0	0	0	10	0	1
0	0	0	0	0	0	0	0	4	0	7
0	0	0	0	0	0	0	0	5	0	6
0	0	0	0	0	0	0	0	9	0	4
0	0	0	0	0	0	0	0	3	0	3
0	0	0	0	0	0	0	0	3	0	2
0	0	0	0	0	0	0	0	5	0	3
0	0	0	0	0	0	0	0	7	0	4
1	0	0	0	0	0	0	0	2	0	0
0	0	0	0	0	0	0	0	4	0	25
0	1	0	0	0	0	0	0	4	0	4
0	0	0	0	0	0	0	0	5	0	8
0	0	0	0	0	1	0	0	7	0	2
0	0	0	0	0	0	0	0	1	0	2
0	0	0	0	0	0	0	0	2	0	3
0	0	0	0	0	0	0	0	6	0	7
1	2	0	0	0	1	0	0	0	2	1
0	0	0	0	0	1	0	0	3	0	4
0	0	1	0	0	0	2	0	5	0	2
0	0	0	0	0	0	0	0	1	0	0
0	0	0	0	0	0	0	0	1	0	2
1	1	0	0	0	0	0	0	0	0	11
1	0	0	0	0	0	0	0	1	0	0

0	0	0	0	0	0	0	1	1	0	0
0	1	0	0	0	0	0	0	8	0	0
1	1	0	0	0	0	0	0	1	1	3
0	0	0	0	0	1	0	0	0	0	5
0	0	0	0	0	0	1	0	0	0	6
0	1	0	0	0	1	0	0	6	0	0
1	0	0	0	0	0	0	0	1	0	1
0	0	0	0	0	0	0	0	6	0	11
0	1	0	0	0	0	0	2	4	0	11
0	0	0	0	0	0	0	0	0	0	2
0	0	0	0	0	0	0	1	1	0	7
0	0	0	0	0	0	0	0	0	0	14
0	0	0	0	0	0	0	0	5	0	9
0	0	0	0	0	0	0	0	0	0	5
0	0	0	0	0	0	0	0	2	0	3
0	0	0	0	0	1	0	0	2	0	2
0	0	0	0	0	1	0	0	3	0	19
0	0	0	0	0	0	0	0	1	0	11
0	0	0	0	0	0	1	0	0	0	7
0	0	0	0	0	0	0	0	2	0	4
0	0	0	0	0	0	0	0	2	0	2
0	0	0	0	0	0	0	0	6	0	2
0	0	0	0	0	0	0	0	4	0	2
0	0	0	0	0	0	1	0	4	0	12
0	0	0	0	0	0	0	0	6	0	8
0	0	0	0	0	1	0	0	7	0	7
0	0	0	0	0	0	0	0	2	0	3
0	0	0	0	0	0	0	0	1	0	4
0	0	0	0	0	0	0	0	2	0	2

0	0	0	0	0	1	0	0	6	0	4
0	0	0	0	0	0	0	0	2	0	6
0	0	0	0	0	0	0	0	5	0	4
0	0	0	0	0	0	0	0	2	0	5
0	0	0	0	0	0	0	0	3	0	10
0	0	0	0	0	0	0	0	5	0	3
0	0	0	0	0	0	0	0	2	0	10
0	0	0	0	0	0	4	0	10	0	5
0	0	0	0	0	0	1	0	2	0	4
0	0	0	0	0	0	0	0	7	0	3
0	0	0	0	0	0	0	0	10	0	11
0	0	0	0	0	0	0	0	4	0	3
0	0	0	0	0	0	0	0	5	0	3
0	0	0	0	0	0	0	0	3	0	4
0	0	0	0	0	0	0	0	10	0	2
0	0	0	0	0	0	0	0	4	0	1
0	0	0	0	0	0	0	0	4	0	7
0	0	0	0	0	0	0	0	3	0	1
0	0	0	0	0	0	0	0	6	0	11
0	0	0	0	0	0	1	0	17	0	2
0	0	0	0	0	0	0	0	10	0	5
0	0	0	0	0	0	8	0	3	0	1
0	0	0	0	0	0	0	0	8	0	5
0	0	0	0	0	0	0	0	4	0	6
0	0	0	0	0	0	2	0	9	0	9
0	0	0	0	0	0	0	0	5	0	2
0	0	0	0	0	0	0	0	2	0	2
0	0	0	0	0	0	0	0	7	0	12
0	0	0	0	0	0	0	0	1	0	5

0	0	0	0	0	0	0	0	18	0	12
0	0	0	0	0	0	0	0	6	0	5
0	0	0	0	0	0	0	0	0	0	4
0	0	0	0	0	1	2	0	4	0	4
0	0	0	0	0	0	0	0	0	0	3
0	0	0	0	0	0	0	0	5	0	9
0	0	0	0	0	0	0	0	8	0	0
0	0	0	0	0	0	1	0	3	0	1
0	0	0	0	0	0	0	0	2	0	3
0	0	0	0	0	0	0	0	10	0	5
0	0	0	0	0	0	0	0	8	0	6
0	0	0	0	0	0	0	0	10	0	6
0	0	0	0	0	0	0	0	11	0	10
0	0	0	0	0	0	0	0	0	0	6
0	0	0	0	0	0	0	0	9	0	10
0	0	0	0	0	0	0	0	0	0	9
0	0	0	0	0	0	0	0	2	0	22
0	0	0	0	0	0	1	0	3	0	12
0	0	0	0	0	0	1	0	4	0	15
0	0	0	0	0	0	1	0	11	0	5
0	0	0	0	0	2	0	0	8	0	4
0	0	0	0	0	2	0	0	9	0	5
1	0	0	0	0	6	6	0	9	0	6
0	0	0	0	0	2	1	0	2	0	4
0	0	0	0	0	0	0	0	0	0	4
0	0	0	0	0	1	0	0	1	0	3
0	0	0	0	0	1	1	0	3	0	3
0	0	0	0	0	0	0	0	5	0	5
0	0	0	0	0	0	2	0	1	1	7

0	0	0	0	0	0	0	0	0	0	0	0	0	0	0	1	0	8
0	0	0	0	0	0	0	0	0	0	0	0	0	0	0	0	0	3
0	0	0	0	0	0	0	0	0	0	0	0	0	0	0	1	0	0
0	0	0	0	0	0	0	0	0	0	0	0	0	0	0	1	0	7
0	0	0	0	0	0	0	0	0	0	0	0	0	0	0	0	0	4
0	0	0	0	0	0	0	0	0	0	0	0	0	0	0	3	0	0
0	0	0	0	0	0	0	0	0	0	0	0	0	0	0	5	0	8
0	0	0	0	0	0	0	0	0	0	0	0	0	0	0	1	0	4
0	0	0	0	0	0	0	0	0	0	0	0	0	3	0	4	0	1
0	0	0	0	0	0	0	0	0	0	0	0	0	0	0	2	0	2
0	0	0	0	0	0	0	0	0	0	0	0	0	0	0	0	0	10
0	0	0	0	0	0	0	0	0	0	0	0	0	1	0	1	0	9
0	0	0	0	0	0	0	0	0	0	0	0	0	0	0	5	0	2
0	0	0	0	0	0	0	0	0	0	0	0	0	0	0	2	0	0

methal	micten	mussen	mysiad	nemert	neosin	nicaes	notoma	notsca	nuchar	olifat
0	0	0	1	1	0	2	0	2	0	0
0	0	0	0	2	0	0	0	0	0	0
0	0	0	0	0	0	0	0	0	0	0
0	0	0	1	3	0	0	0	0	0	0
0	0	0	0	3	0	0	0	1	0	0
0	0	0	0	0	0	1	0	1	0	0
0	0	0	0	0	0	0	0	1	0	0
0	0	0	1	3	0	0	0	1	5	0
0	0	0	0	6	0	0	0	0	9	0
0	0	1	0	1	0	0	0	0	2	0
0	0	0	0	0	0	0	0	1	13	0
0	0	1	0	3	0	0	0	0	5	0
0	0	0	0	8	0	0	0	0	4	0
0	0	0	0	0	0	0	0	0	1	0
0	0	0	1	4	0	0	0	0	5	0
0	0	0	0	1	0	0	0	1	0	0
0	0	0	0	1	0	0	0	0	5	0
0	0	0	0	2	0	0	0	0	0	0
0	0	0	0	1	0	0	0	0	0	0
0	0	0	2	1	0	0	0	0	2	0
0	0	0	0	0	0	0	0	0	3	0
0	0	0	0	0	0	0	0	0	4	0
0	0	0	0	1	0	0	0	0	0	0
0	0	0	0	2	0	0	0	0	0	0
0	0	1	0	0	0	1	0	0	2	0
1	0	0	0	1	0	0	0	0	18	0
0	0	0	0	0	0	0	0	0	4	0
0	0	0	0	2	0	0	1	0	2	0

0	0	0	0	0	0	0	0	0	3	0
0	0	0	0	1	0	0	0	0	1	0
0	0	0	0	2	0	0	0	0	5	0
0	0	0	0	1	0	0	0	0	3	0
0	0	0	0	11	0	0	0	0	6	0
0	0	0	0	1	0	1	0	0	2	0
0	0	0	0	0	0	0	0	0	10	0
0	0	0	0	4	0	0	0	0	5	0
0	0	0	0	12	1	0	0	3	0	0
0	0	0	0	1	0	0	0	0	2	0
0	2	0	0	1	0	0	0	0	1	0
0	0	0	0	5	0	0	0	0	2	0
0	0	0	0	3	0	0	0	0	1	0
0	0	2	0	3	0	0	0	0	5	0
0	0	0	0	1	0	0	0	2	0	0
0	0	0	0	1	0	0	0	2	0	0
0	0	0	0	2	0	0	0	3	3	0
0	0	0	0	2	0	0	0	0	2	0
0	0	0	0	3	0	0	0	0	4	0
0	1	0	0	9	0	0	0	3	2	0
1	0	0	0	1	0	0	0	0	18	0
0	0	0	0	0	0	1	0	0	14	0
0	0	0	0	0	0	0	0	0	5	0
0	0	0	0	2	0	1	0	0	0	0
0	0	0	0	0	0	0	0	0	3	0
0	0	0	0	2	0	0	1	0	1	0
0	0	0	0	1	0	0	0	2	1	0
0	0	0	2	4	0	0	0	0	3	0
0	0	0	0	0	0	0	0	1	3	0

0	0	0	0	0	0	0	0	0	1	0
0	0	0	0	0	0	0	0	0	1	0
0	0	0	0	1	0	0	0	0	0	0
0	0	0	0	0	0	0	0	0	0	0
0	0	0	0	0	0	0	0	0	0	0
0	0	1	1	0	0	0	0	0	0	0
0	0	0	0	2	0	0	0	0	3	0
0	0	2	0	2	0	0	0	1	4	0
0	0	0	0	0	0	0	0	0	0	0
0	0	0	1	0	0	0	0	0	0	0
0	0	0	3	0	0	0	0	0	2	0
0	0	0	1	0	0	0	0	0	1	0
0	0	0	0	1	0	0	0	0	0	0
0	0	0	0	1	0	0	0	0	0	0
0	0	0	0	2	0	0	0	0	0	0
0	0	0	0	4	0	0	0	0	1	0
0	0	0	0	0	0	0	0	0	0	0
0	0	0	0	1	0	0	0	0	1	0
0	0	0	0	0	0	0	0	0	0	0
0	0	0	0	1	0	0	0	0	17	0
0	0	0	0	0	0	0	0	0	0	0
0	0	0	0	1	0	2	0	0	0	0
0	0	0	0	3	0	0	0	0	0	0
0	0	0	0	0	0	0	0	1	0	0
0	0	0	1	0	0	0	0	0	0	0
0	0	0	0	0	0	0	0	0	0	0
0	0	0	1	0	0	0	0	0	0	0
0	0	0	0	0	0	0	0	0	0	0
0	0	4	0	0	0	0	0	0	12	0

0	0	0	0	4	0	0	0	0	3	0
0	0	0	0	2	0	0	0	0	0	0
0	0	3	0	3	0	0	0	0	11	0
0	0	2	0	1	0	0	1	0	4	0
0	0	0	0	1	0	0	0	0	0	0
0	0	0	0	0	0	0	0	0	0	0
0	1	0	0	0	0	0	0	0	0	0
0	2	0	0	0	0	0	0	1	0	0
0	0	0	0	0	0	0	1	0	1	0
0	0	3	0	1	0	0	0	1	2	0
0	0	0	0	0	0	0	0	0	0	0
0	0	0	1	1	0	0	0	0	0	0
0	0	0	0	1	0	0	0	0	5	0
0	0	0	0	1	0	0	0	1	0	0
0	0	0	0	1	0	0	0	1	3	0
0	0	0	0	1	0	0	0	0	0	0
0	0	0	0	1	0	0	0	0	1	0
0	0	0	0	5	0	0	0	0	4	0
0	1	0	0	0	0	0	0	0	1	0
0	1	0	0	0	0	0	0	0	0	0
0	0	0	0	0	0	0	0	0	4	0
0	0	0	2	2	0	0	0	0	0	0
0	0	3	0	2	0	0	0	0	2	0
0	0	2	0	2	0	0	0	0	5	0
0	0	5	0	3	0	1	0	0	9	0
0	0	0	0	1	0	0	0	0	2	0
0	0	0	1	1	0	0	0	0	0	0
0	0	0	0	0	0	0	1	0	0	0
0	0	0	0	1	0	0	0	1	1	0

0	0	0	0	0	0	0	0	0	0	0
0	0	0	0	0	0	0	0	0	0	0
0	0	0	0	0	0	0	0	0	1	0
0	0	0	0	0	0	0	0	0	0	0
0	0	0	0	1	0	0	0	1	0	0
0	0	0	0	1	0	1	0	1	1	0
0	0	0	0	0	0	0	0	1	0	0
0	0	0	0	0	0	0	0	0	0	0
0	0	0	0	2	0	0	0	3	0	0
0	0	0	0	5	0	1	0	0	0	0
0	0	0	0	1	0	0	0	0	0	0
0	0	0	0	0	0	0	0	8	5	0
0	0	0	0	0	1	0	0	1	0	0
0	0	0	0	0	0	0	0	2	5	0
0	4	0	0	1	0	0	0	0	8	0
0	0	0	0	0	0	0	2	0	2	0
0	1	0	0	0	0	0	0	5	1	0
0	0	0	0	0	0	0	0	1	3	0
0	0	0	0	2	0	0	0	1	1	0
0	0	1	0	0	0	0	0	1	1	0
0	0	0	0	0	0	0	0	0	0	0
0	0	0	0	2	0	0	0	0	2	1
0	0	0	0	0	0	0	0	0	1	0
0	2	0	0	3	0	0	0	1	4	0
0	0	0	1	2	0	3	0	0	0	0
0	0	0	0	3	0	0	0	0	2	0
0	0	0	0	1	0	0	0	2	1	0
0	3	0	0	1	0	0	0	0	1	0
0	1	0	0	0	0	0	0	0	3	0

0	1	0	0	0	0	0	0	1	0	0
0	1	0	0	5	0	0	0	1	4	0
0	0	0	0	3	0	0	0	1	0	0
0	0	0	0	0	1	0	0	0	0	0
0	0	0	0	1	0	3	0	0	0	0
0	0	0	0	3	0	0	0	0	0	0
0	0	0	0	3	0	0	0	3	0	0
0	0	0	0	3	0	3	0	0	1	0
0	0	0	0	1	0	0	0	0	0	0
1	0	0	1	0	0	1	0	0	0	0
0	0	0	0	1	0	0	0	0	0	0
0	0	0	1	1	0	0	0	1	0	0
0	0	0	0	0	0	0	0	0	1	0
0	0	0	0	0	0	2	0	0	0	0
0	0	0	0	0	0	0	0	1	1	0
0	0	0	0	2	0	0	0	0	1	0
0	0	0	0	0	0	0	0	0	0	0
0	1	0	0	0	0	0	0	4	1	0
0	0	1	0	0	0	0	0	0	1	0
0	0	0	0	0	0	0	0	0	6	0
0	2	0	0	0	0	1	0	0	1	0
0	0	0	0	1	0	0	0	1	7	0
0	1	1	0	0	0	0	0	0	7	0
0	1	0	0	0	0	0	0	0	4	0
1	0	0	0	4	0	1	0	0	0	0
1	0	0	0	0	0	1	0	0	0	0
0	0	0	0	3	0	3	0	0	0	0
0	0	0	1	4	0	0	0	0	0	0
0	0	0	0	4	0	0	0	0	0	0

0	0	0	0	2	0	0	0	0	1	0
0	0	0	0	0	0	0	0	0	0	0
0	0	0	0	2	0	1	0	0	0	0
0	0	0	0	1	0	0	0	0	0	0
0	0	0	0	0	0	0	0	1	0	0
0	0	0	0	0	0	0	0	0	0	0
0	0	0	0	0	0	0	0	0	0	0
0	0	0	0	1	0	0	0	0	0	0
0	0	0	1	0	0	0	0	0	0	0
0	0	0	0	3	0	0	0	0	0	0
0	0	0	0	1	0	0	0	1	0	0
0	0	0	0	1	0	0	0	0	0	0
0	0	0	0	1	0	0	0	0	0	0
0	0	0	0	3	0	0	0	0	0	0
0	0	0	0	4	0	0	0	1	0	0
0	0	0	0	0	0	0	0	0	0	0
0	0	0	0	0	0	0	0	0	0	0
0	0	0	0	2	0	0	0	2	0	0
0	0	0	0	0	0	2	0	0	2	0
0	0	0	0	1	0	1	0	0	0	0
1	0	0	0	0	0	1	0	0	0	0
0	0	0	0	3	0	0	0	2	0	0
0	0	0	0	3	0	0	0	0	0	0
0	0	0	0	1	0	0	0	0	0	0
0	0	0	0	0	0	5	0	1	0	0
0	0	0	0	0	0	0	0	1	1	0
0	0	0	0	1	0	6	0	1	0	0
0	0	0	0	2	0	1	0	0	0	0
0	0	0	0	3	0	0	0	0	0	0

0	0	0	0	0	0	0	0	0	0	0
0	0	0	0	1	0	1	0	0	0	0
0	0	0	0	3	0	1	0	0	0	0
0	0	0	0	4	0	0	0	0	0	0
0	0	0	0	0	0	0	0	0	0	0
0	0	0	2	0	0	0	0	0	0	0
0	0	0	0	3	0	0	0	0	0	0
0	0	0	0	2	0	0	0	0	0	0
0	0	0	0	4	0	0	0	0	0	0
0	0	0	0	5	0	0	0	0	0	0
0	0	0	0	2	0	0	0	0	0	0
0	0	0	0	2	0	3	0	0	0	0
0	0	0	0	8	0	0	0	4	0	0
0	0	0	0	0	0	0	0	1	0	0
0	0	0	0	2	0	0	0	0	0	0
0	0	0	0	5	0	0	0	0	0	0
0	0	0	0	3	0	0	0	0	0	0
0	0	0	0	0	0	0	0	0	0	0
0	0	0	0	3	0	0	0	0	0	0
0	0	0	0	1	0	0	0	0	0	0
0	0	0	1	1	0	1	0	0	1	0
0	0	0	0	3	0	0	0	0	0	0
0	0	0	1	1	0	0	0	0	0	0
0	0	0	0	2	0	2	0	0	0	0
1	0	0	0	1	0	2	0	0	0	0
0	0	0	0	6	0	3	0	0	0	0
1	0	0	0	0	0	0	0	0	0	0
0	0	0	1	0	0	0	0	0	0	0
0	0	0	1	0	0	0	0	0	0	0

0	0	0	0	2	0	0	0	0	0	0
0	0	0	0	0	0	0	0	0	0	0
0	0	0	0	0	0	0	0	0	0	0
0	0	0	0	3	0	0	0	0	0	0
0	0	0	0	1	0	0	0	1	0	0
0	0	0	0	0	0	0	0	0	0	0
0	0	0	0	4	0	0	0	0	0	0
0	0	0	0	3	0	0	0	0	0	0
0	0	0	0	0	0	0	0	0	0	0
0	0	0	0	0	0	0	0	0	0	0
0	0	0	0	2	0	0	0	0	0	0
1	0	0	0	1	0	0	0	0	0	0
0	0	0	1	1	0	0	0	0	0	0
0	0	0	1	0	0	4	0	0	0	0
1	0	0	0	0	0	0	0	0	0	0
0	0	0	0	1	0	0	0	0	0	0
0	0	0	0	2	0	0	0	1	0	0
0	0	0	0	4	0	0	0	0	0	0
0	0	0	0	0	0	1	0	0	0	0
0	0	0	0	0	0	0	0	1	0	0
0	0	0	0	0	0	0	0	0	1	0
0	0	0	0	4	0	0	0	0	0	0
0	0	0	0	2	0	0	0	0	0	0
2	0	0	0	3	0	0	0	0	0	0
0	0	0	0	6	0	0	0	1	1	0
0	0	0	0	1	0	2	0	0	0	0
1	0	0	0	9	1	0	0	1	3	0
0	0	0	1	4	0	0	0	0	0	0
0	0	0	0	0	0	0	0	0	1	0

0	0	0	0	3	0	0	0	0	1	0
0	0	0	0	4	0	0	2	0	1	0
0	0	1	1	1	0	0	0	1	0	0
1	0	0	3	2	0	0	0	0	1	0
0	0	0	0	5	0	0	0	0	4	0
0	0	0	0	0	0	0	0	0	6	0
0	0	0	0	1	0	0	0	0	9	0
0	0	0	0	0	0	0	3	1	12	0
0	0	0	0	4	0	0	2	0	1	0
0	0	0	0	3	0	0	1	0	1	0
0	0	0	0	4	0	0	0	0	2	0
1	0	0	2	1	0	0	0	0	0	0
0	0	0	0	0	0	0	0	3	3	0
0	0	0	0	6	0	0	1	0	0	0
0	0	0	1	2	0	0	2	0	0	0
0	0	0	0	7	0	0	0	0	10	0
0	0	0	0	22	0	0	0	0	9	0
0	0	0	0	1	0	0	0	0	13	0
0	0	0	3	0	0	0	0	0	0	0
0	0	0	3	3	0	0	0	0	0	0
0	0	0	0	1	0	0	0	0	0	0
0	0	0	0	12	0	0	0	0	9	0
0	0	0	0	0	0	0	0	0	0	0
0	0	1	2	4	0	0	0	0	0	0
0	0	0	2	1	0	0	0	0	0	0
0	0	1	0	2	0	0	0	0	2	0
0	0	1	0	2	0	0	0	0	0	0
0	0	0	0	2	0	0	0	0	2	0
0	0	0	0	4	0	0	0	0	5	0

1	0	0	1	6	0	0	0	1	3	0
0	0	0	0	16	0	0	0	0	10	0
0	0	0	0	0	0	0	0	0	4	0
0	0	4	0	9	0	0	0	0	17	0
0	0	2	0	21	0	0	0	0	15	0
0	0	1	0	7	0	0	0	0	2	0
0	0	0	0	14	0	0	0	0	14	0
0	0	0	3	4	0	0	0	0	8	0
0	0	0	0	6	0	0	0	0	8	0
0	0	0	1	8	0	0	0	0	5	0
0	0	0	0	6	0	0	0	0	0	0
0	0	0	0	1	0	0	0	0	0	0
2	0	0	0	0	0	0	0	0	5	0
0	0	0	0	2	0	0	0	0	3	0
1	0	0	0	5	0	2	0	0	6	0
0	0	0	0	7	0	0	0	0	2	0
0	0	0	0	6	0	0	0	0	12	0
0	0	0	0	2	0	0	0	0	8	0
0	0	0	0	3	0	0	0	0	2	0
0	0	0	1	7	0	0	0	0	0	0
0	0	0	0	5	0	0	0	0	5	0
2	0	0	0	2	0	0	0	0	4	0
0	0	0	1	0	0	0	0	0	0	0
0	0	0	0	4	0	0	0	0	5	0
1	0	0	1	5	0	0	0	0	9	0
1	0	0	0	5	0	0	0	0	5	0
0	0	0	0	3	0	0	0	0	6	0
0	0	0	3	5	0	0	0	0	4	0
0	0	0	2	3	0	0	0	0	5	0

1	0	0	1	2	0	0	0	0	0	0
0	0	0	0	1	0	0	0	0	0	0
0	0	0	0	1	0	0	0	0	0	0
1	0	0	0	2	0	0	0	0	2	0
0	0	0	0	3	0	1	0	0	0	0
0	0	0	0	1	0	0	0	1	3	0
0	0	0	0	4	0	0	0	0	0	0
0	0	0	0	5	0	0	0	0	1	0
0	0	0	0	1	0	0	0	0	0	0
0	0	0	0	3	0	0	0	0	0	0
0	0	0	0	5	0	0	0	0	4	0
0	0	0	0	4	0	0	0	1	2	0
0	0	0	0	1	0	0	1	0	3	0
2	0	0	0	2	0	0	0	0	0	0
0	0	0	2	2	0	0	0	2	3	0
0	0	0	2	6	0	0	1	0	1	0
0	0	0	0	0	0	0	0	0	2	0
0	0	0	0	3	0	0	1	0	5	0
0	0	0	0	1	0	0	0	0	3	0
0	0	0	0	8	0	0	0	0	6	0
0	0	0	0	3	0	1	0	0	1	0
0	0	0	0	5	0	0	0	0	0	0
0	0	0	0	3	0	0	0	0	0	0
0	0	0	0	0	0	0	0	1	0	0
0	0	0	0	3	0	0	0	0	0	0
0	0	0	0	0	0	0	0	0	0	0
0	0	0	0	0	0	0	0	0	0	0
0	0	0	0	2	0	0	0	0	0	0
0	0	0	0	2	0	0	0	0	0	0

0	0	0	0	0	0	0	0	0	0	0
0	0	0	0	0	0	0	0	0	0	0
1	0	0	0	2	0	0	0	0	0	0
0	0	0	0	4	0	0	0	1	0	0
0	0	0	0	0	0	0	0	0	0	0
0	0	0	0	1	0	0	0	0	0	0
0	0	0	0	4	0	0	0	0	0	0
0	0	0	0	1	0	0	0	1	0	0
0	0	0	0	1	0	0	0	2	0	0
0	0	0	0	1	0	0	0	0	0	0
0	0	0	0	3	0	1	0	0	2	0
0	0	0	1	2	0	0	0	0	0	0
0	0	0	0	1	0	0	0	2	0	0
0	0	0	0	2	0	0	0	0	0	0
0	0	0	2	2	0	0	0	0	0	0
0	0	0	1	1	0	0	0	0	0	0
0	0	0	0	0	0	0	0	0	0	0
0	0	0	0	4	0	0	0	0	6	0
1	0	0	0	2	0	0	0	0	0	0
0	0	0	0	2	0	0	0	0	0	0
0	0	0	0	1	0	0	0	0	0	0
0	0	0	0	2	0	1	0	0	0	0
0	0	0	0	0	0	0	0	0	0	0
0	0	0	1	1	0	0	0	0	0	0
0	0	0	0	2	0	0	0	0	0	0
0	0	0	0	5	0	1	0	0	0	0
0	0	0	0	2	0	0	0	0	0	0
0	0	0	0	1	0	0	0	0	0	0
0	0	0	0	0	0	0	0	0	0	0

0	0	0	0	1	0	1	0	0	0	0
0	0	0	0	0	0	0	0	0	0	0
0	0	0	0	1	0	0	0	0	0	0
0	0	0	0	1	0	0	0	0	0	0
0	0	0	1	0	0	0	0	0	0	0
0	0	0	0	2	0	0	0	0	0	0
0	0	0	0	2	0	0	0	0	0	0
0	0	0	0	2	0	0	0	0	0	0
0	0	0	0	1	0	0	0	0	0	0
0	0	0	0	1	0	0	0	0	0	0
0	0	0	0	1	0	1	0	0	0	0
0	0	0	1	2	0	0	0	0	0	0
0	0	0	0	5	0	0	0	0	0	0
0	0	0	0	2	0	1	0	0	0	0
0	0	0	0	2	0	1	0	0	0	0
0	0	0	0	3	0	0	0	0	0	0
1	0	0	0	4	0	0	0	0	0	0
0	0	0	0	1	0	0	0	0	0	0
0	0	0	0	0	0	0	0	0	0	0
0	0	0	1	4	0	1	1	1	0	0
0	0	0	0	0	0	0	0	0	0	0
0	0	0	0	2	0	1	0	0	0	0
0	0	0	0	5	0	0	0	0	0	0

nemert	nicaes	notomi	notsca	nuchar	olithi	ophang	orbpap	owepet	paguri	papaus
2	0	0	0	0	0	0	1	0	0	0
4	0	0	0	0	0	0	0	0	0	0
1	0	0	0	0	0	0	0	0	0	0
1	0	0	0	0	1	0	1	0	0	0

1	0	0	0	0	0	1	0	0	0	0
4	1	0	0	0	1	0	0	0	0	0
4	0	0	0	0	0	0	0	0	0	0
1	0	0	0	0	0	0	0	0	0	0
4	0	0	2	0	2	0	0	0	0	0
5	0	0	0	0	0	0	1	0	0	0
0	1	0	0	1	0	0	0	1	0	0
0	1	0	0	26	0	0	0	2	0	0
2	3	0	0	0	0	0	0	0	0	0
0	14	0	0	0	0	0	0	0	0	0
0	3	0	0	0	0	0	0	0	0	0
1	8	0	0	2	0	0	0	1	0	0
1	3	0	0	2	1	0	0	0	0	0
2	0	0	0	2	0	0	0	0	0	0
1	0	0	0	10	0	0	0	1	0	0
0	0	0	0	20	0	0	0	2	0	0
1	0	0	1	0	0	0	1	0	0	0
3	0	0	0	0	0	0	0	0	0	0
4	0	0	0	0	0	0	0	0	0	0
0	0	0	0	0	0	0	1	0	0	0
1	2	0	0	0	0	0	0	0	0	0
0	0	0	1	0	0	0	1	0	0	0
1	1	0	1	0	0	0	0	0	0	0
2	0	0	0	1	0	0	2	0	0	0
1	0	0	5	0	0	0	0	0	0	0
1	0	0	0	0	0	0	0	0	0	0
0	0	0	8	0	0	0	0	0	0	0
2	2	0	7	5	0	0	0	0	0	0
0	2	0	0	0	0	0	0	0	0	0

0	0	0	4	0	0	0	0	1	0	0
0	0	0	1	0	0	0	0	0	0	0
2	1	0	1	0	0	0	0	0	0	0
0	1	0	4	0	1	0	0	0	0	0
1	0	0	1	0	1	0	0	0	0	0
1	0	0	5	2	0	0	0	0	0	0
1	1	0	6	12	0	0	0	0	0	0
3	1	0	11	3	0	0	0	1	0	0
0	0	0	6	1	0	0	0	0	0	0
4	0	0	9	7	0	0	0	0	0	0
0	0	0	1	1	0	0	0	0	0	0
0	1	0	2	21	0	0	0	0	0	0
0	2	0	4	9	0	0	0	0	0	0
3	1	0	2	13	0	0	0	0	0	0
0	0	0	10	13	0	0	0	2	0	0
0	0	0	3	0	0	0	0	0	0	0
0	0	0	4	10	0	0	0	0	0	0
0	0	0	1	13	0	0	0	0	0	0
1	2	0	1	3	0	0	0	0	0	0
1	0	0	3	4	0	0	0	1	0	0
0	2	0	1	5	0	0	0	1	0	0
1	0	0	11	5	0	0	0	0	0	0
0	0	0	1	3	0	0	0	0	0	0
0	0	0	2	3	0	0	0	0	0	0
2	1	0	15	4	0	0	0	1	0	0
0	1	0	0	1	0	0	0	1	0	0
0	0	0	0	1	0	0	0	0	0	0
1	0	0	2	0	0	0	0	0	0	0
0	1	0	0	6	0	0	0	1	0	0

0	2	0	0	4	0	0	0	0	0	0
0	0	0	4	8	0	0	0	0	0	0
2	2	0	0	0	1	0	0	0	0	0
1	1	0	0	0	0	0	0	0	0	1
1	1	0	0	0	0	0	0	0	0	0
1	2	0	0	0	0	0	0	0	0	0
0	2	0	0	2	0	0	0	2	0	0
1	1	0	0	1	0	0	0	1	0	0
2	1	0	0	1	0	0	0	0	0	0
1	0	0	8	21	0	0	0	2	0	0
1	2	0	0	2	0	0	0	0	0	0
1	2	0	2	1	0	0	0	0	0	0
4	2	0	0	0	0	0	0	0	0	0
1	0	0	1	2	0	0	0	1	0	0
1	0	0	0	2	0	0	0	0	0	0
2	2	0	0	1	0	0	0	0	0	0
0	0	0	1	14	0	0	0	1	0	0
1	0	0	0	8	0	0	0	1	0	0
2	1	0	5	25	0	0	0	1	0	0
0	0	0	0	12	0	0	0	0	0	0
0	0	0	6	28	0	0	0	0	0	0
2	1	0	2	6	0	0	0	0	0	0
1	0	0	7	46	0	0	0	1	0	0
2	0	0	5	47	0	0	0	2	0	0
1	0	0	4	35	0	0	0	1	0	0
0	0	0	4	24	0	0	0	3	0	0
0	3	0	0	11	0	0	0	0	0	0
2	0	0	2	33	0	0	0	1	0	0
3	0	0	1	26	0	0	0	1	0	0

0	3	0	0	0	0	0	0	0	0	0
1	0	0	1	19	0	0	0	0	0	0
0	1	0	1	10	0	0	0	0	0	0
0	2	0	0	14	0	0	0	0	0	0
0	1	0	0	2	0	0	0	2	0	0
0	4	0	0	2	0	0	0	1	0	0
3	2	0	0	3	0	0	0	0	0	0
0	3	0	1	1	0	0	0	0	0	0
0	5	0	0	1	0	0	0	0	0	0
0	0	0	0	2	0	0	0	1	0	0
0	0	0	0	1	0	0	0	1	0	0
0	2	0	0	5	0	0	0	0	0	0
3	0	0	0	1	0	0	0	1	0	0
0	1	0	0	8	0	0	0	1	1	0
0	1	0	0	3	0	0	0	2	0	0
0	3	0	0	6	0	0	0	2	0	0
0	0	0	0	1	0	0	0	1	0	0
3	1	0	0	5	0	0	0	1	0	0
3	3	0	0	4	0	0	0	2	0	0
1	0	0	0	3	0	0	0	3	0	0
1	3	0	0	8	0	0	0	2	0	0
2	1	0	0	5	0	0	0	1	0	0
1	4	0	0	0	2	0	0	1	0	0
0	1	0	1	6	0	0	0	0	0	0
1	4	0	0	6	0	0	0	0	0	0
2	1	0	0	7	0	0	0	1	0	0
2	1	0	0	4	0	0	0	1	0	0
1	2	0	0	0	0	0	0	0	0	0
3	0	0	0	14	0	0	0	2	0	0

1	2	0	0	3	0	0	0	3	0	0
2	0	0	2	30	0	0	0	3	0	0
1	2	0	0	3	0	0	0	2	0	0
2	4	0	0	4	0	0	0	1	0	0
3	1	0	0	3	0	0	0	1	0	0
3	6	0	0	1	0	0	0	0	0	0
2	3	0	0	3	0	0	0	1	0	0
0	3	0	0	4	0	0	0	0	0	0
1	1	0	0	2	0	0	0	0	0	0
1	0	0	0	22	0	0	0	0	0	0
2	1	0	3	19	0	0	0	0	0	0
1	0	0	1	40	0	0	0	1	0	0
1	0	0	1	35	0	0	0	1	0	0
4	1	0	1	26	0	0	0	3	0	0
3	0	0	1	44	0	0	0	0	0	0
1	0	0	1	8	1	0	0	1	0	0
1	1	0	2	21	0	0	0	1	0	0
0	3	1	1	22	0	0	0	0	0	0
4	0	0	0	38	1	0	0	0	0	0
1	0	0	0	34	0	0	0	1	0	0
0	0	0	7	21	0	0	0	0	0	0
1	0	0	3	1	0	0	0	3	0	0
1	0	0	8	21	1	0	0	0	0	0
3	0	0	8	38	0	0	0	0	0	0
2	0	0	8	84	0	0	0	2	0	0
0	0	0	6	65	0	0	0	1	0	0
2	0	0	6	71	0	0	0	1	1	0
6	0	0	4	28	0	0	0	0	0	0
0	0	0	6	42	0	0	0	1	0	0

0	0	0	1	0	0	0	0	0	0	0
0	0	0	4	4	0	0	0	2	0	0
0	1	0	3	0	0	0	0	1	0	0
0	0	0	5	25	0	0	0	0	0	0
0	0	0	7	37	0	0	0	2	0	0
0	0	0	2	8	0	0	0	0	0	0
2	0	0	3	0	1	0	0	0	0	0
4	2	0	2	1	1	0	0	1	0	0
1	1	0	0	2	0	0	0	1	0	0
3	1	0	0	1	0	0	0	0	0	0
0	0	0	3	0	0	0	0	0	0	0
3	1	0	0	0	1	0	0	0	0	0
1	2	0	2	2	0	0	0	0	0	0
1	2	0	0	0	0	0	0	0	0	0
0	0	0	8	1	2	0	0	0	0	0
1	1	0	0	0	0	0	0	0	0	0
1	0	0	8	50	1	0	0	0	0	0
0	1	0	6	5	0	0	0	1	0	0
0	0	0	12	40	0	0	0	1	0	0
1	0	0	11	9	1	0	0	0	0	0
2	0	0	3	0	0	0	0	1	0	0
0	1	0	1	0	0	0	0	0	0	0
1	0	0	0	0	1	0	0	1	0	0
2	0	0	10	0	0	0	0	1	0	0
2	0	0	2	0	0	0	0	0	0	0
2	1	0	0	0	0	0	0	0	0	0
0	0	0	5	2	0	0	0	1	0	0
1	0	0	4	6	0	0	0	0	0	0
3	0	0	2	3	0	0	0	0	0	0

0	0	0	1	0	2	0	0	0	0	0
3	0	0	0	0	0	0	0	0	0	0
0	1	0	0	0	0	0	0	0	0	0
1	2	0	2	0	0	0	0	0	0	0
1	0	0	0	0	0	0	0	0	0	0
0	0	0	0	0	0	0	0	0	0	0
1	0	0	0	1	0	0	0	0	0	0
1	2	0	5	0	0	0	0	0	0	0
2	0	0	1	0	0	0	0	0	0	0
0	0	0	4	0	0	0	0	0	0	0
0	0	0	5	0	0	0	0	0	0	0
0	0	0	4	0	0	0	0	0	0	0
1	1	0	7	0	1	0	0	0	0	0
0	2	0	1	0	0	0	0	0	0	0
0	0	0	3	0	0	0	0	0	0	0
0	0	0	1	0	0	0	0	0	0	0
0	0	0	3	0	0	0	1	0	0	0
2	2	0	0	0	0	0	0	0	0	0
0	1	0	2	0	0	0	0	0	0	0
1	0	0	1	0	0	0	0	0	0	0
0	0	0	0	0	0	0	0	0	0	0
0	0	0	1	0	0	0	0	0	0	0
0	0	0	3	0	0	0	0	0	0	0
1	0	0	1	0	0	0	0	0	0	0
4	0	0	3	0	1	0	0	0	0	0
2	0	0	1	0	0	0	0	0	0	0
2	0	0	4	0	0	0	0	0	0	0
1	0	0	0	0	0	0	0	0	0	0
0	1	0	6	0	0	0	0	0	0	0

0	0	0	1	0	0	0	0	0	0	0
1	0	0	1	0	0	0	0	0	0	0
0	2	0	1	0	0	0	0	0	0	0
1	0	0	4	0	1	0	0	0	0	0
0	0	0	0	1	0	0	0	0	0	0
2	0	0	2	0	1	0	0	0	0	0
2	0	0	0	0	1	0	0	0	0	0
3	1	0	4	0	0	0	0	0	0	0
2	0	0	9	0	1	0	0	0	0	0
1	1	0	2	0	0	0	0	0	0	0
4	0	0	0	0	0	0	0	0	0	0
0	0	0	12	4	0	0	0	0	0	0
3	0	0	9	2	0	0	0	0	0	0
1	0	0	1	5	0	0	0	0	0	0
2	0	0	12	19	0	0	0	0	0	0
1	1	0	2	4	0	0	0	1	0	0
0	1	0	2	8	0	0	0	0	0	0
0	0	0	2	0	0	0	0	0	0	0
0	0	0	2	0	0	0	0	0	0	0
2	0	0	1	0	0	0	0	0	0	0
0	0	0	4	18	0	0	0	0	0	0
1	0	0	1	20	0	0	0	0	0	0
2	1	0	5	14	0	0	0	0	0	0
1	0	0	3	3	0	0	0	0	0	0
0	0	0	3	3	0	0	0	0	0	0
0	0	0	4	3	0	0	0	1	0	0
0	0	0	4	2	2	0	0	0	0	0
0	1	0	3	7	0	0	0	0	0	0
1	0	0	9	17	0	0	0	0	0	0

3	0	0	8	31	1	0	0	0	0	0
2	0	0	6	14	0	0	0	1	0	0
0	0	0	5	6	0	0	0	0	0	0
1	0	0	11	1	0	0	0	3	0	0
0	1	0	2	9	0	0	0	0	0	0
1	0	0	7	56	0	0	0	1	0	0
0	0	0	15	22	0	0	0	2	0	0
4	0	0	4	13	0	0	0	1	0	0
0	0	0	0	22	0	0	0	0	0	0
2	0	0	2	2	1	0	0	2	0	0
0	1	0	3	0	0	0	0	0	0	0
2	0	0	9	0	0	0	0	0	0	0
3	0	0	9	11	0	0	0	0	0	0
0	1	0	6	20	0	0	0	0	0	0
4	0	0	2	24	0	0	0	3	0	0
0	1	0	0	17	0	0	0	3	0	0
4	0	0	10	56	0	0	0	2	0	0
0	0	0	6	19	0	0	0	0	0	0
2	1	0	0	6	1	0	0	0	0	0
0	0	0	5	6	0	0	0	2	0	0
1	0	0	10	15	0	0	0	0	0	0
6	1	0	11	16	0	0	0	0	0	0
2	0	0	0	37	0	0	0	0	0	0
9	0	0	0	20	0	0	0	2	0	0
2	0	0	0	31	0	0	0	1	0	0
0	3	0	0	16	0	0	0	0	0	0
4	0	0	0	14	0	0	0	1	0	0
1	0	0	0	60	0	0	0	3	0	0
3	0	0	3	27	0	0	0	3	0	0

1	0	0	0	0	0	0	0	0	0	0
6	0	0	0	63	2	0	0	3	0	0
1	0	0	0	27	0	0	0	4	0	0
0	4	0	0	7	0	0	0	1	0	0
0	3	0	0	10	0	0	0	0	0	0
0	2	0	0	18	0	0	0	1	0	0
0	2	0	0	16	0	0	0	10	0	0
0	1	0	0	5	0	0	0	1	0	0
3	2	0	0	13	0	0	0	1	0	0
2	3	0	0	9	0	0	0	3	0	0
0	1	0	0	9	0	0	0	2	0	0
0	0	0	0	20	0	0	0	3	0	0
2	0	1	0	53	0	0	0	7	0	0
2	0	0	0	26	0	0	0	3	0	0
2	1	0	0	19	0	0	0	3	0	0
5	0	0	0	12	1	0	0	5	0	0
2	1	0	0	2	0	0	0	2	0	0
1	1	0	0	19	0	0	0	4	0	0
1	0	0	0	21	1	0	0	6	0	0
3	0	0	0	6	1	0	0	9	0	0
0	1	0	0	3	0	0	0	6	0	0
0	0	0	0	3	0	0	0	0	0	0
4	2	0	0	2	0	0	0	3	0	0
3	1	0	0	3	0	0	0	2	0	0
1	1	0	0	4	0	0	0	3	0	0
2	0	0	0	3	0	0	0	2	0	0
5	2	0	0	0	0	0	0	1	1	0
1	0	0	0	17	0	0	0	1	0	0
0	3	0	0	2	0	0	0	0	0	0

1	1	0	0	0	0	0	0	0	0	0
2	2	0	0	3	0	0	0	0	0	0
0	3	0	0	0	0	0	0	0	0	0
1	1	0	0	1	0	0	0	0	0	0
0	1	0	0	0	0	0	0	0	0	0
2	2	0	0	0	0	0	0	0	0	0
0	2	0	0	1	0	0	0	0	0	0
0	2	0	0	2	0	0	0	0	0	0
1	0	0	0	6	0	0	0	0	0	0
1	6	0	0	0	0	0	0	0	0	0
0	1	0	0	3	0	0	0	0	0	0
2	3	0	0	5	0	0	0	0	0	0
2	2	0	0	2	0	0	0	0	0	0
0	1	0	0	3	0	0	1	0	0	0
1	0	0	0	14	0	0	0	0	0	0
1	3	0	0	7	0	0	0	0	0	0
2	1	0	0	2	0	0	0	0	0	0
0	0	0	0	2	1	0	0	0	0	0
0	2	0	0	4	0	0	0	0	0	0
2	1	0	0	1	1	0	1	0	0	0
1	0	0	0	2	0	0	0	0	0	0
2	0	0	0	7	0	0	0	0	0	0
2	1	0	0	4	0	0	0	1	0	0
2	0	0	0	6	2	0	0	2	0	0
1	0	0	0	0	2	0	0	1	0	0
3	0	0	0	7	1	0	2	0	0	0
2	1	0	0	1	0	0	0	3	0	0
2	2	0	1	68	0	0	0	3	0	0
0	1	0	0	1	0	0	0	1	0	0

1	0	0	0	0	0	0	0	2	0	0
0	1	0	0	7	0	0	0	1	0	0
0	1	0	0	8	0	0	1	3	0	0
3	0	0	0	29	0	0	0	4	0	0
1	1	0	0	7	0	0	0	5	0	0
4	1	0	0	31	0	0	0	4	0	0
1	0	0	0	30	0	0	0	3	0	0
0	0	0	0	13	0	0	0	1	0	0
4	0	0	0	14	0	0	0	3	0	0
2	2	0	0	17	0	0	0	0	0	0
1	0	0	0	14	0	0	0	0	0	0
2	0	0	0	18	0	0	0	3	0	0
8	0	0	0	29	0	0	0	0	0	1
1	0	0	0	25	0	0	0	1	0	0
2	0	0	1	40	0	0	0	1	0	0
1	0	0	0	14	0	0	0	2	0	0
0	2	0	0	6	0	0	0	2	0	0
0	2	0	0	0	0	0	0	0	0	0
4	0	0	1	2	0	0	0	1	0	0
4	0	0	0	1	0	0	0	1	0	0
5	0	0	0	6	1	0	0	3	0	0
0	0	0	2	34	0	0	0	0	0	0
2	0	0	1	20	0	0	0	1	0	0
6	2	0	1	9	1	0	0	1	0	0
2	1	0	0	11	0	0	0	0	0	0
1	0	0	2	14	0	0	0	1	0	0
2	0	0	6	11	0	0	0	1	0	0
4	0	0	1	14	1	0	0	0	0	0
2	0	0	4	0	1	0	0	0	0	0

4	0	0	4	9	0	0	0	3	0	0
0	0	0	2	5	0	0	0	1	0	0
2	0	0	5	1	0	0	0	1	0	0
5	0	0	4	4	0	0	0	0	0	0
2	0	0	8	5	0	0	0	1	0	0
1	0	0	7	2	1	0	0	1	0	0
2	0	0	4	1	0	0	0	1	0	0
0	0	0	0	0	0	0	0	0	0	0
2	0	0	6	0	0	0	0	1	0	0
0	0	0	5	28	0	0	0	1	0	0
0	0	0	6	2	0	0	0	1	0	0
0	0	0	4	7	0	0	0	0	0	0
0	0	0	6	3	0	0	0	1	0	0
2	0	0	6	0	0	0	0	0	0	0
1	0	0	3	5	0	0	0	1	0	0
0	0	0	4	6	0	0	0	0	0	0
4	0	0	10	28	0	0	0	0	0	0
0	0	0	5	16	0	0	0	0	0	0
0	0	0	14	28	0	0	0	0	0	0
3	0	0	4	0	1	0	0	0	0	0
1	0	0	13	30	0	0	0	1	0	0
1	0	0	4	13	0	0	0	0	0	0
1	0	0	9	5	0	0	0	1	0	0
3	0	0	8	0	0	0	0	1	0	0
0	1	0	5	2	0	0	0	0	0	0
2	2	0	4	2	0	0	0	1	0	0
0	1	0	3	0	0	0	0	1	0	0
0	0	0	0	1	0	0	0	1	0	0
0	0	0	0	0	0	0	0	0	0	0

0	0	0	0	3	0	0	0	0	0	0
1	0	0	5	0	0	0	0	2	0	0
1	1	0	1	0	0	0	0	1	0	0
1	0	0	0	1	0	0	0	1	0	0
0	0	0	1	7	0	0	0	0	0	0
0	0	0	2	1	0	0	0	0	0	0
0	0	0	1	1	0	0	0	0	0	0
1	0	0	0	1	0	0	0	0	0	0
1	0	0	0	5	0	0	0	0	0	0
1	5	0	0	4	3	0	0	0	0	0
2	0	0	1	0	0	0	0	0	0	0
0	0	0	2	2	0	0	0	0	0	0
1	1	0	0	0	0	0	0	0	0	0
0	0	0	2	0	0	0	0	0	0	0
0	0	0	9	6	0	0	0	0	0	0
0	2	0	1	0	0	0	0	0	0	0
0	0	0	1	1	0	0	0	0	0	0
0	0	0	5	0	0	0	0	0	0	0
2	1	0	0	0	0	0	0	0	0	0

plaaus	polydo	priauc	psethi	scalib	scoben	scocyl	scolel	solpar	solsil	sphaer
0	0	16	0	0	0	8	0	0	0	0
0	0	24	0	0	1	17	0	0	0	0
0	0	43	0	0	1	18	0	0	0	0
0	0	34	0	0	0	15	0	0	0	0
0	0	26	0	0	0	8	0	0	0	0
0	0	50	0	0	1	0	0	0	0	0
0	0	34	0	0	2	9	0	0	0	0
0	0	41	0	0	2	3	0	0	0	0

0	0	19	0	0	5	11	0	0	0	0
0	0	23	0	0	0	9	0	0	0	0
0	0	16	0	0	0	1	0	0	0	0
0	0	9	0	0	1	2	0	0	0	0
0	0	26	0	0	1	0	0	0	0	0
0	0	11	0	0	1	1	0	0	0	0
1	0	26	0	0	1	0	0	0	0	0
0	0	33	0	0	0	0	0	0	0	0
0	0	13	0	0	1	0	0	0	0	0
0	0	9	0	0	0	1	0	0	0	0
0	0	24	0	0	0	1	0	0	0	0
0	0	10	0	0	1	0	0	0	0	1
0	0	16	0	0	1	11	0	0	0	0
0	0	22	0	0	0	5	0	0	0	0
0	0	18	0	0	0	9	0	0	0	0
0	0	20	0	0	2	11	0	0	0	0
0	0	20	0	0	1	12	0	0	0	0
0	0	6	0	0	1	1	0	0	0	0
0	0	20	0	0	0	15	0	0	0	0
0	0	28	0	0	1	10	0	0	0	0
0	0	17	0	0	1	8	0	0	0	0
0	0	20	0	0	1	7	0	0	0	0
0	0	16	0	0	0	8	0	0	0	0
0	0	27	0	0	2	5	6	0	0	0
0	0	14	0	0	2	6	0	0	0	0
0	0	12	0	0	2	4	0	0	0	0
0	0	5	1	0	0	5	1	0	0	0
0	0	36	0	0	0	18	0	0	0	0
0	0	33	0	0	1	12	0	0	0	0

0	0	31	0	0	2	6	0	0	0	0
0	0	21	0	0	2	9	0	0	0	0
0	0	9	0	0	3	4	2	0	0	0
0	0	26	0	0	0	4	0	0	0	0
0	0	7	0	0	1	9	1	0	0	0
0	0	11	0	0	2	7	0	0	0	0
0	0	15	0	0	1	6	5	0	0	0
0	0	7	0	0	1	10	0	0	0	0
0	0	13	0	0	0	7	2	0	0	0
0	0	22	0	0	1	8	0	0	0	0
0	0	2	0	0	0	2	0	0	0	0
0	0	0	0	0	0	3	14	0	0	0
0	0	8	0	0	1	11	7	0	0	0
0	0	4	0	0	2	8	1	0	0	0
0	0	5	0	0	1	7	1	0	0	0
0	0	7	0	0	0	1	0	0	0	0
0	0	0	0	0	1	8	3	0	0	0
0	0	0	0	0	1	7	0	0	0	0
0	0	0	0	0	2	3	1	0	0	0
0	0	1	0	0	1	8	0	0	0	0
0	0	1	0	0	1	6	1	0	0	0
0	0	4	0	0	2	7	5	0	0	0
0	0	2	0	0	1	6	4	0	0	0
0	0	0	0	0	3	11	0	0	0	0
0	0	4	0	0	1	10	0	0	0	0
0	0	5	0	0	0	4	0	0	0	0
0	0	6	0	0	2	6	0	0	0	0
0	0	17	0	0	1	3	0	0	0	0
0	0	1	0	0	1	3	0	0	0	0

0	0	2	0	0	1	2	0	0	0	0
0	0	2	0	0	0	2	1	0	0	0
0	0	7	0	1	1	2	1	0	0	0
1	0	7	0	0	1	5	0	0	0	0
0	0	23	0	0	0	5	0	0	0	0
0	0	22	0	0	1	9	0	0	0	0
0	0	13	0	0	1	2	0	0	0	0
0	0	20	0	0	1	2	0	0	0	0
0	0	19	0	0	1	5	0	0	0	0
0	0	15	0	0	0	4	0	0	0	0
0	0	16	0	0	1	10	0	0	0	0
0	0	18	0	0	2	6	0	0	0	0
0	0	3	0	0	3	9	0	0	0	0
0	0	10	0	0	2	4	0	0	0	0
0	0	15	0	0	1	10	1	0	0	0
0	0	3	0	0	0	12	0	0	0	0
0	0	20	0	0	1	7	0	0	0	0
0	0	18	0	0	0	8	1	0	0	0
1	0	18	0	0	0	5	0	0	0	0
0	0	9	0	0	1	2	0	0	0	0
0	0	11	0	0	3	10	0	0	0	0
0	0	3	0	0	1	5	0	0	0	0
0	0	26	0	0	0	0	0	0	0	0
0	0	10	0	0	1	11	0	0	0	0
0	0	4	0	0	1	3	0	0	0	0
0	0	11	0	0	0	1	0	0	0	0
0	0	37	0	0	0	6	0	0	1	0
0	0	34	0	0	0	6	0	0	0	0
0	0	46	0	0	1	6	0	0	0	0

0	0	7	0	0	1	3	0	0	0	0
0	0	30	0	0	1	7	0	0	0	0
0	0	37	0	0	0	3	0	0	0	0
0	0	8	0	0	1	0	0	0	0	0
0	0	21	0	0	1	0	0	0	0	0
0	0	10	0	0	0	0	0	1	0	0
0	0	0	0	0	1	0	0	0	0	0
0	0	16	0	0	0	0	0	0	0	0
0	0	12	0	0	0	1	0	0	0	0
2	0	17	0	0	1	4	0	0	0	0
0	0	14	0	0	1	8	0	0	0	0
0	0	21	0	0	0	6	0	0	0	0
0	0	14	0	0	0	4	0	0	0	0
0	0	10	0	0	1	1	0	0	0	0
0	0	19	0	0	0	3	0	0	0	0
0	0	13	0	0	0	0	0	0	0	0
0	0	8	0	0	0	10	0	0	0	0
0	0	20	0	0	0	3	0	0	0	0
0	0	10	0	0	0	0	0	0	0	0
0	0	29	0	0	0	5	0	0	0	0
0	0	9	0	0	1	0	0	0	0	0
0	0	11	0	0	1	2	0	0	0	0
0	0	35	0	0	1	1	0	0	0	0
0	0	51	0	0	2	3	0	0	0	0
0	0	34	0	0	1	2	0	0	0	0
0	0	2	0	0	1	3	0	0	0	0
0	0	20	0	0	0	3	0	0	0	0
0	0	25	0	0	1	18	0	0	0	0
0	0	1	0	0	0	3	0	0	0	0

0	0	6	0	0	0	5	0	0	0	0
1	0	16	0	0	5	2	0	0	0	0
0	0	19	0	0	4	13	0	0	0	0
0	0	4	0	0	0	2	0	0	0	0
0	0	8	0	0	2	5	0	0	0	0
1	0	26	0	0	2	0	0	0	0	0
0	0	10	0	0	0	12	0	0	0	0
0	0	32	0	0	0	5	0	0	0	0
0	0	27	0	0	0	3	0	0	0	0
0	0	29	0	0	0	9	0	0	0	0
0	0	22	0	0	2	11	0	0	0	0
0	0	18	0	0	0	3	0	0	0	0
0	0	7	0	0	0	7	0	0	0	0
0	0	6	0	0	0	3	0	0	0	0
0	0	28	0	0	1	3	1	0	0	0
0	0	10	0	0	0	6	0	0	0	0
0	0	5	0	0	0	4	0	0	0	0
0	0	1	0	0	0	2	0	0	0	0
0	0	1	0	0	2	5	0	0	0	0
0	0	2	0	0	2	5	1	0	0	0
0	0	17	0	0	1	5	0	0	0	0
0	0	18	0	0	1	2	0	0	0	0
0	0	23	0	0	0	3	0	0	0	0
0	0	16	0	0	0	10	0	0	0	0
0	0	12	0	0	0	6	0	0	0	0
0	0	17	0	0	0	3	0	0	0	0
0	0	8	0	0	0	8	0	0	0	0
0	0	6	0	0	1	6	0	0	0	0
0	0	0	0	0	0	3	1	0	0	0

0	0	4	0	0	0	5	0	0	0	0
0	0	12	0	0	0	4	0	0	0	0
0	0	13	0	0	1	7	0	0	0	0
0	0	4	0	0	0	3	0	0	0	0
0	0	6	0	0	0	3	0	0	0	0
0	0	13	0	0	0	0	0	0	0	0
0	0	27	0	0	1	4	0	0	0	0
0	0	42	0	0	1	4	0	0	0	0
0	0	4	0	0	1	3	0	0	0	0
0	0	17	0	0	2	7	0	0	0	0
0	0	17	0	0	2	5	0	0	0	0
0	0	3	0	0	6	5	1	0	0	0
0	0	6	0	0	0	6	0	0	0	0
0	0	14	0	0	4	1	0	0	0	0
0	0	12	0	0	0	7	0	0	0	0
0	0	26	0	0	0	7	1	0	0	0
0	0	9	0	0	0	9	0	0	0	0
0	0	16	0	0	0	10	0	0	0	0
0	0	12	0	0	1	6	0	0	0	0
0	0	9	0	0	2	4	0	0	0	0
0	0	8	0	0	0	8	0	0	0	0
0	0	22	0	0	1	11	0	0	0	0
0	0	15	0	0	1	7	0	0	0	0
0	0	19	0	0	0	12	0	0	0	0
0	0	22	0	0	0	4	0	0	0	0
0	0	13	0	0	1	10	0	0	0	0
0	0	22	0	0	0	4	0	0	0	0
0	0	13	0	0	2	4	0	0	0	0
0	0	25	0	0	2	4	0	0	0	0

0	0	32	0	0	2	9	0	0	0	0
0	0	18	0	0	2	10	0	0	0	0
0	0	11	0	0	1	19	0	0	0	0
0	0	10	0	0	1	10	0	0	0	0
0	0	32	0	0	0	16	0	0	0	0
0	0	22	0	0	2	12	0	0	0	0
0	0	0	0	0	0	4	0	0	0	0
0	0	8	0	0	0	1	0	0	0	0
0	0	20	0	0	0	10	0	0	0	0
0	0	13	0	0	0	11	0	0	0	0
0	0	18	0	0	0	20	0	0	0	0
0	0	15	0	0	0	3	0	0	0	0
0	0	10	0	0	3	9	0	0	0	0
0	0	17	0	0	1	5	0	0	0	0
0	0	26	0	0	2	10	0	0	0	0
0	0	15	0	0	0	0	1	0	0	0
0	0	8	0	0	0	10	0	0	0	0
0	0	15	0	0	3	13	1	0	0	0
0	0	3	0	0	0	3	0	0	0	0
0	0	1	0	0	0	5	0	0	0	0
0	0	19	0	0	0	9	0	0	0	0
0	0	41	0	0	1	3	0	0	0	0
0	0	6	0	0	0	6	0	0	0	0
0	0	22	0	0	1	7	0	0	0	0
0	0	39	0	0	2	2	0	0	0	0
0	0	26	0	0	1	0	0	0	0	0
0	0	1	0	0	1	0	6	0	0	0
0	0	0	0	0	0	5	3	0	0	0
0	0	4	0	0	0	2	16	0	0	0

0	0	10	0	0	1	11	0	0	0	0	
0	0	8	0	0	0	4	5	0	0	0	
0	0	9	0	0	1	3	0	0	0	0	
0	0	25	0	0	1	0	0	0	0	0	
0	0	28	0	0	0	0	0	0	0	0	
0	0	23	0	0	1	2	0	0	0	0	
0	0	23	0	0	0	0	0	0	0	0	
0	0	16	0	0	1	1	0	0	0	0	
0	0	38	0	0	1	1	0	0	0	0	
0	0	9	0	0	2	5	0	0	0	0	
0	0	21	0	0	1	0	0	0	0	0	
0	0	4	0	0	1	3	0	0	0	0	
0	0	8	0	0	1	2	0	0	0	0	
0	0	19	0	0	0	1	0	0	0	0	
0	0	12	0	0	0	7	0	0	0	0	
0	0	19	0	0	0	0	0	0	0	0	
0	0	17	0	0	0	0	0	0	0	0	
0	0	0	0	0	0	4	0	0	0	0	
0	0	8	0	0	2	7	0	0	0	0	
0	0	8	0	0	0	4	0	0	0	0	
0	0	21	0	0	0	0	0	0	0	0	
0	0	27	0	0	1	15	0	0	0	0	
0	0	29	0	0	0	9	0	0	0	0	
0	0	14	0	0	0	6	0	0	0	0	
0	0	14	0	0	0	8	0	0	0	0	
0	0	13	0	0	0	10	0	0	0	0	
0	0	23	0	0	1	11	0	0	0	0	
0	0	31	0	0	0	8	0	0	0	0	
0	0	5	0	0	0	5	0	0	0	0	

0	0	31	0	0	0	8	0	0	0	0
0	0	5	0	0	0	5	0	0	0	0
0	0	6	0	0	0	3	0	0	0	0
0	0	50	0	0	0	7	0	0	0	0
0	0	31	0	0	0	5	0	0	0	0
0	0	15	0	0	0	2	0	0	0	0
0	0	26	0	0	0	6	0	0	0	0
0	0	24	0	0	0	8	0	0	0	0
0	0	14	0	0	0	1	0	0	0	0
0	0	25	0	0	0	4	0	0	0	0
0	0	23	0	0	0	0	0	0	0	0
0	0	6	0	0	0	4	0	0	0	0
0	0	20	0	0	0	2	0	0	0	0
0	0	14	0	0	0	10	0	0	0	0
0	0	2	0	0	0	2	0	0	0	0
0	0	11	0	0	1	5	0	0	0	0
0	0	16	0	0	2	6	0	0	0	0
0	0	15	0	0	1	5	0	0	0	0
0	0	27	0	0	4	3	0	0	0	0
0	0	4	0	0	0	6	1	0	0	0
0	0	34	0	0	0	11	0	0	0	0
0	0	13	0	0	0	7	0	0	0	0
0	0	2	0	0	1	1	0	0	0	0
0	0	10	0	0	0	5	0	0	0	0
0	0	12	0	0	1	3	0	0	0	0
0	0	6	0	0	1	0	0	0	0	0
0	0	20	0	0	0	2	0	0	0	0
0	0	16	0	0	0	1	0	0	0	0
0	0	37	0	0	0	5	0	0	0	0

0	0	19	0	0	0	9	0	0	0	0
0	0	25	0	0	1	8	0	0	0	0
0	0	17	0	0	0	3	0	0	0	0
0	0	17	0	0	1	5	0	0	0	0
0	0	17	0	0	0	4	0	0	0	0
0	0	26	0	0	0	7	0	0	0	0
0	0	25	0	0	1	7	0	0	0	0
0	0	9	0	0	1	0	0	0	0	0
0	0	20	0	0	0	2	0	0	0	0
0	0	40	0	0	3	4	0	0	0	0
0	0	8	0	0	0	3	0	0	0	0
0	0	17	0	0	2	2	0	0	0	0
0	0	8	0	0	0	0	0	0	0	0
0	0	0	1	0	1	4	0	0	0	0
0	0	5	0	0	1	5	0	0	0	0
0	0	16	0	0	0	0	0	0	0	0
0	0	10	0	0	1	2	0	0	0	0
0	0	33	0	0	0	0	0	0	0	0
0	0	26	0	0	1	4	0	0	0	0
0	0	4	0	0	0	1	0	0	0	0
1	0	21	0	0	0	2	0	0	0	0
0	0	9	0	0	1	4	0	0	0	0
0	0	74	0	0	0	0	0	0	0	2
0	0	13	0	0	0	0	0	0	0	0
2	0	30	0	0	0	1	0	0	0	0
0	0	5	0	0	0	0	0	0	0	0
1	0	29	0	0	2	0	0	0	0	0
0	0	11	0	0	0	1	0	0	0	0
0	0	38	0	0	0	1	0	0	0	0

0	0	62	0	0	0	1	0	0	0	0
0	0	41	0	0	0	1	0	0	0	0
1	0	38	0	0	0	0	0	0	0	0
0	0	22	0	0	1	0	0	0	0	0
0	0	38	0	0	0	0	0	0	0	0
0	0	24	0	0	0	1	0	0	0	0
0	0	28	0	0	0	0	0	0	0	1
0	0	93	0	0	0	2	0	0	0	0
0	0	55	0	0	0	0	0	0	0	0
0	0	8	0	0	2	10	0	0	0	0
0	0	7	0	0	0	1	0	0	0	0
0	0	3	0	0	1	1	0	0	0	0
0	0	19	0	0	1	0	0	0	0	0
0	1	22	0	0	2	0	0	0	0	0
0	0	13	0	0	1	0	0	0	0	0
0	0	23	0	0	0	0	0	0	0	0
0	0	23	0	0	0	0	0	0	0	0
0	0	19	0	0	0	0	0	0	0	0
0	0	12	0	0	0	0	0	0	0	0
0	0	17	0	0	0	0	0	0	0	0
0	0	10	0	0	0	3	0	0	0	0
0	0	22	0	0	1	3	0	0	0	0
0	0	10	0	0	1	1	0	0	0	0
0	0	27	0	0	1	4	0	0	0	0
0	0	27	0	0	2	2	0	0	0	0
0	0	18	0	0	0	11	0	0	0	0
0	0	14	0	0	0	0	0	0	0	0
0	0	21	0	0	0	1	0	0	0	0
0	0	14	0	0	0	2	0	0	0	0

0	0	14	0	0	1	0	0	0	0	0
0	0	18	0	0	1	7	0	0	0	0
0	0	22	0	0	0	2	0	0	0	0
0	0	21	0	0	0	4	0	0	0	0
0	0	19	0	0	0	4	0	0	0	0
0	0	13	0	0	0	1	0	0	0	0
0	0	19	0	0	0	5	0	0	0	0
0	0	24	0	0	1	0	0	0	0	0
0	0	25	0	0	0	1	0	0	0	0
0	0	17	0	0	0	0	0	0	0	0
0	0	17	0	0	0	6	0	0	0	0
0	0	13	0	0	0	2	0	0	0	0
0	0	22	0	0	1	2	0	0	0	0
0	0	23	0	0	0	4	0	0	0	0
0	0	18	0	0	2	8	0	0	0	0
0	0	10	0	0	0	2	0	0	0	0
0	0	9	0	0	0	4	0	0	0	0
0	0	21	0	0	4	4	0	0	0	0
0	0	13	0	0	1	2	0	0	0	0
0	0	27	0	0	0	5	0	0	0	0
0	0	36	0	0	0	3	0	0	0	0
0	0	24	0	0	1	2	0	0	0	0
0	0	26	0	0	0	7	0	0	0	0
0	0	31	0	0	0	7	0	0	0	0
0	0	24	0	0	2	9	0	0	0	0
0	0	15	0	0	0	12	0	0	0	0
0	0	30	0	0	0	6	0	0	0	0
0	0	11	0	0	0	7	0	0	0	0
0	0	9	0	0	0	4	0	0	0	0

0	0	30	0	0	0	4	0	0	0	0
0	0	15	0	0	0	6	0	0	0	0
0	0	22	0	0	0	6	0	0	0	0
0	0	57	0	0	0	7	0	0	0	0
0	0	14	0	0	0	0	0	0	0	0
0	0	9	0	0	0	14	0	0	0	0
0	0	20	0	0	0	6	0	0	0	0
0	0	24	0	0	0	4	0	0	0	0
0	0	70	1	0	0	0	0	0	0	0
0	0	58	0	0	0	4	0	0	0	0
0	0	34	0	0	0	4	0	0	0	0
0	0	25	0	0	1	2	0	0	0	0
0	0	13	0	0	0	2	0	0	0	0
0	0	13	0	0	0	0	0	0	0	0
0	0	28	0	0	0	1	0	0	0	0
0	0	13	0	0	0	0	0	0	0	0
0	0	9	0	0	0	1	0	0	0	0
0	0	19	0	0	0	0	0	0	0	0
0	0	16	0	0	1	1	0	0	0	0
0	0	39	0	0	0	1	0	0	0	0
0	0	32	0	0	0	2	0	0	0	0
0	0	21	0	0	0	1	0	0	0	0
0	0	47	0	0	1	1	0	0	0	0
1	0	36	0	0	1	2	0	0	0	0
0	0	0	0	0	0	0	0	0	0	0
3	0	47	0	0	1	2	0	0	0	0
0	0	9	0	0	4	5	0	0	0	0
0	0	41	0	0	1	3	0	0	0	0
0	0	39	0	0	1	1	0	0	0	0

0	0	64	0	0	3	5	0	0	0	0
0	0	0	0	0	0	0	0	0	0	0
0	0	19	0	0	0	0	0	0	0	0
0	0	23	0	0	0	2	0	0	0	0
0	0	1	0	0	0	0	0	0	0	0
0	0	18	0	0	1	4	1	0	0	0
0	0	32	0	0	0	5	0	0	0	0
0	0	24	0	0	0	9	0	0	0	0
0	0	15	0	0	0	3	0	0	0	0
0	0	15	0	0	2	1	0	0	0	0
0	0	16	0	0	1	2	0	0	0	0
0	0	28	0	0	2	5	0	0	0	0
0	0	26	0	0	0	11	1	0	0	0
0	0	0	0	0	2	5	0	0	0	0

olithi	ophang	ophiur	orbpap	owepet	palaem	papaus	parexc	parlyr	parnou	patiri
0	0	0	4	0	0	0	0	0	5	0
0	0	0	3	0	0	0	0	0	4	0
0	0	0	4	0	0	1	0	0	2	0
1	0	0	1	0	0	0	0	0	2	0
0	0	0	2	0	0	0	0	0	0	0
0	0	0	0	0	0	0	0	0	0	0
0	0	0	2	0	0	1	0	0	4	0
0	0	0	0	0	0	0	0	0	0	0
0	0	0	0	0	0	0	0	0	0	0
1	0	0	0	0	0	0	0	0	0	0
0	0	0	0	2	0	0	0	0	0	0
0	0	0	0	1	0	0	0	0	1	0
0	0	0	0	3	0	0	0	0	0	0
0	0	0	0	3	0	0	0	0	0	0
0	0	0	0	5	0	0	0	0	0	0
0	0	0	0	1	0	0	0	0	0	0
0	0	0	0	4	0	0	0	0	0	0
0	0	0	0	1	0	0	0	0	0	0
0	0	0	0	0	0	0	0	0	0	0
0	0	0	0	1	0	0	0	0	2	0
0	0	0	0	0	0	0	0	0	1	0
0	0	0	0	5	0	0	0	0	0	0
0	0	0	1	0	0	1	0	0	0	0
0	0	0	2	1	0	0	0	0	0	0
0	0	1	0	0	0	0	0	0	5	0
0	0	0	0	7	0	0	0	0	0	0
0	0	0	0	0	0	0	0	0	0	0
1	0	0	0	0	0	0	0	0	0	0

0	0	0	0	2	0	0	0	0	0	0
0	0	0	0	0	0	0	0	0	0	0
0	0	0	0	0	0	1	0	1	0	0
3	0	0	0	0	0	0	0	1	0	0
1	0	0	0	3	0	0	0	0	0	0
2	0	0	1	1	0	0	0	0	0	0
0	0	0	0	1	0	0	0	0	0	0
0	0	0	0	1	0	0	0	0	0	0
1	0	1	0	11	0	0	0	0	1	0
0	0	0	0	0	0	0	0	0	0	0
0	0	0	0	0	0	0	0	0	3	0
0	0	0	0	1	0	0	0	0	0	0
1	0	0	0	1	0	0	0	0	1	0
0	0	0	0	2	0	0	0	0	0	0
0	0	0	1	0	0	3	0	0	0	0
1	0	0	0	0	0	2	0	0	0	0
0	0	0	0	5	0	0	0	0	0	0
1	0	0	0	0	0	0	0	0	0	0
0	0	0	0	3	0	1	0	0	0	0
0	0	0	0	0	0	0	0	0	3	0
1	0	0	0	1	0	0	0	0	0	0
2	0	0	0	35	0	0	0	1	0	0
1	0	0	0	14	0	0	0	0	0	0
1	0	0	0	0	0	0	0	0	0	0
0	0	0	0	3	0	0	0	0	0	0
0	0	0	0	3	0	0	0	0	0	0
0	0	0	0	0	0	0	0	0	0	0
0	0	0	0	1	0	0	0	0	0	0
0	0	0	0	2	0	0	0	0	0	0

0	0	0	0	0	0	0	0	0	0	0
0	0	0	1	0	0	0	0	0	0	0
0	0	0	0	2	0	1	0	0	0	0
1	0	0	0	0	0	5	0	0	0	0
0	0	0	0	1	0	0	0	0	2	0
0	0	0	0	1	0	0	0	0	2	0
1	0	0	1	2	0	0	0	0	0	0
0	0	0	0	16	0	0	0	0	0	0
0	0	0	0	2	0	0	0	0	0	0
0	0	0	0	0	0	0	0	0	0	0
0	0	0	0	0	0	0	0	0	0	0
0	0	0	0	1	0	0	0	0	0	0
0	0	0	1	0	0	0	0	0	0	0
0	0	0	0	0	0	0	0	0	0	0
0	0	0	1	0	0	0	0	0	0	0
0	0	0	0	0	0	0	0	0	0	0
0	0	0	0	0	0	1	0	0	0	0
0	0	0	0	0	0	0	0	0	0	0
0	0	0	0	0	0	0	0	0	0	0
0	0	0	0	3	0	0	0	0	0	0
0	0	0	0	1	0	0	0	0	0	0
0	0	0	0	0	0	16	0	0	0	0
1	0	0	0	0	0	1	0	0	6	0
0	0	0	0	0	0	3	0	0	0	0
0	0	0	0	0	0	0	0	0	1	0
0	0	0	0	0	0	0	0	0	0	0
0	0	0	0	0	0	0	0	0	0	0
0	0	0	0	0	0	0	0	0	2	0
0	0	0	0	3	0	0	0	0	1	0

0	0	0	0	4	0	0	0	0	1	0
0	0	0	0	1	0	0	0	0	8	0
1	0	0	0	4	0	0	0	0	0	0
0	0	0	1	10	0	0	0	0	0	0
0	0	0	0	0	0	2	0	0	0	0
0	0	0	0	0	0	1	0	0	0	0
0	0	0	0	0	0	0	0	0	0	0
0	0	0	0	0	0	1	0	0	0	0
0	0	0	0	0	0	0	0	0	0	0
0	0	0	0	9	0	0	0	0	0	0
0	0	0	0	0	0	0	0	0	0	0
0	0	0	0	0	0	0	0	0	0	0
0	0	0	0	3	0	0	0	0	0	0
1	0	0	0	0	0	37	0	0	0	0
0	0	0	0	0	0	1	0	0	3	0
0	0	0	1	0	0	21	0	0	0	0
0	0	0	0	0	0	1	0	0	1	0
0	0	0	0	1	0	0	0	0	0	0
0	0	0	0	0	0	5	0	0	0	0
0	0	0	0	1	0	1	0	0	0	0
0	0	0	0	1	0	0	0	1	0	0
0	0	0	0	2	0	1	0	0	0	0
0	0	0	0	3	0	0	0	0	0	0
1	0	0	0	5	0	0	0	0	0	0
1	0	0	2	4	0	0	0	0	1	0
0	0	0	0	0	0	1	0	0	0	0
0	0	0	0	0	0	0	0	0	2	0
0	0	0	0	3	0	0	0	0	0	0
0	0	0	0	0	0	1	0	0	27	0

0	0	0	0	0	0	0	0	0	0	0
0	0	0	1	0	0	1	0	0	3	0
0	0	0	0	0	0	5	0	0	3	0
0	0	0	0	0	0	5	0	0	2	0
0	0	0	0	0	0	0	0	0	0	0
0	0	0	0	0	0	2	0	0	3	0
0	0	0	0	0	0	1	0	0	2	0
0	0	0	3	0	0	1	0	0	3	0
0	0	0	0	0	0	4	0	0	1	0
0	0	0	0	0	0	0	0	0	4	0
0	0	0	0	0	0	0	0	0	2	0
0	0	0	0	0	0	0	0	0	0	0
0	0	0	0	6	0	0	0	0	0	0
1	0	0	0	3	0	0	0	0	0	0
0	0	0	0	0	0	2	0	0	0	0
0	0	0	0	13	0	0	0	0	2	0
0	0	0	0	1	0	0	0	0	20	0
0	0	0	0	0	0	0	0	0	0	0
0	0	0	0	0	0	1	0	0	45	0
0	0	0	0	0	0	1	0	0	3	0
0	0	0	0	3	0	1	0	0	0	0
0	0	0	0	0	0	0	0	0	0	0
0	0	0	0	0	0	0	0	0	0	0
0	0	0	0	0	0	3	0	0	4	0
1	0	0	0	0	0	1	0	0	4	0
0	0	0	0	3	0	0	0	0	0	0
0	0	0	0	0	0	1	0	0	0	0
0	0	0	0	0	0	0	0	0	2	0
0	0	0	0	0	0	0	0	0	4	0

0	0	0	0	0	0	0	0	0	3	0
1	0	0	0	0	0	1	0	0	6	0
0	0	0	0	0	0	1	0	0	0	0
1	0	0	1	0	0	0	0	0	0	0
2	0	0	0	1	0	1	0	0	0	0
0	0	0	3	0	0	0	0	0	3	0
2	0	0	0	0	0	0	0	0	4	0
1	0	0	1	0	0	1	0	0	1	0
0	0	0	2	0	0	1	0	0	3	0
0	0	0	2	1	0	0	0	0	7	0
0	0	0	5	1	0	0	0	0	2	0
1	0	0	5	0	0	0	0	0	5	0
0	0	0	0	0	0	0	0	0	0	0
2	0	0	1	0	0	0	0	0	0	0
0	0	0	0	0	0	4	0	0	3	0
0	0	0	0	0	0	1	0	0	1	0
0	0	0	0	0	0	0	0	0	0	0
0	0	0	0	0	0	0	0	0	0	0
0	0	0	0	0	0	1	0	0	8	0
0	0	0	0	0	0	0	0	0	0	0
0	0	0	0	0	0	0	0	1	0	0
0	0	0	0	0	0	0	0	0	3	0
0	0	0	0	0	0	0	0	0	0	0
0	0	0	0	0	0	0	0	0	0	0
1	0	0	0	0	0	2	0	0	5	0
0	0	0	2	0	0	5	0	0	3	0
0	0	0	0	0	0	1	0	0	3	0
0	0	0	1	0	0	7	0	0	3	0
1	0	0	2	0	0	0	0	0	0	0

0	0	0	4	0	0	3	0	0	5	0
0	0	0	6	0	0	3	0	0	0	0
0	0	0	2	0	0	1	0	0	0	0
0	0	0	2	0	0	3	0	0	0	0
0	0	0	3	0	0	0	0	0	0	0
0	0	0	4	0	0	10	0	0	4	0
0	0	0	5	0	1	11	0	0	2	0
0	0	0	1	0	0	13	0	0	0	0
0	0	0	2	0	0	6	0	0	2	0
0	0	0	2	0	0	18	0	0	0	0
0	0	0	0	0	0	6	0	0	5	0
0	0	0	1	0	0	6	0	0	0	0
0	0	0	2	0	0	6	0	0	0	0
0	0	0	0	0	0	9	0	0	0	0
0	0	0	2	0	0	7	0	0	1	0
0	0	0	4	0	0	5	0	0	0	0
0	0	0	3	0	0	7	0	0	2	0
0	0	0	2	0	0	10	0	0	0	0
1	0	0	0	0	0	10	0	0	0	0
0	0	0	0	0	0	9	0	0	2	0
2	0	0	1	0	0	2	0	0	0	0
1	0	0	1	0	0	7	0	0	0	0
1	0	0	1	0	0	14	0	0	1	0
0	0	0	0	0	0	13	0	0	0	0
1	0	0	1	0	0	6	0	0	0	0
0	0	0	0	0	0	11	0	0	0	0
1	0	0	0	0	0	10	0	0	0	0
0	0	0	0	0	0	10	0	0	0	0
0	0	0	0	0	0	31	0	0	2	0

1	0	0	0	0	0	4	0	0	2	0
0	0	0	0	0	0	7	0	0	2	0
0	0	0	0	0	0	3	0	0	8	0
0	0	0	0	0	0	9	0	0	2	0
1	0	0	0	0	0	10	0	0	2	0
0	0	0	0	0	0	17	0	0	5	0
0	0	0	0	0	0	9	0	0	0	0
0	0	0	0	0	0	14	0	0	0	0
0	0	0	1	0	0	22	0	0	1	0
0	0	0	4	0	0	7	0	0	0	0
0	0	0	4	0	0	33	0	0	1	0
0	0	0	0	0	0	13	0	0	0	0
0	0	0	2	0	0	34	0	0	0	0
0	0	0	2	0	0	36	0	0	1	0
0	0	0	1	0	0	47	0	0	0	0
0	0	0	4	0	0	47	0	0	0	0
0	0	0	1	0	0	30	0	0	0	0
0	0	0	8	0	0	19	0	0	1	0
0	0	0	0	0	0	12	0	0	0	0
0	0	0	3	0	0	5	0	0	1	0
1	0	0	7	0	0	8	0	0	1	0
0	0	0	0	0	0	9	0	0	0	0
0	0	0	8	0	0	16	0	0	1	0
0	0	0	4	0	0	15	0	1	1	0
0	0	0	5	1	0	10	0	0	2	0
1	0	0	3	0	0	18	0	0	6	0
0	0	0	2	0	0	4	0	0	0	0
0	0	0	7	0	0	3	0	0	1	0
0	0	0	6	0	0	2	0	0	0	0

0	0	0	2	0	0	0	0	0	0	0
0	0	0	5	0	0	5	0	0	1	0
0	0	0	0	0	0	0	0	0	0	0
0	0	0	5	0	0	4	0	0	3	0
0	0	0	2	0	0	3	0	0	0	0
0	0	0	5	0	0	2	0	0	0	0
0	0	0	2	0	0	13	0	0	1	0
0	0	0	1	0	0	2	0	0	0	0
0	0	0	1	0	0	3	0	0	0	0
0	0	0	1	0	0	2	0	0	3	0
0	0	0	0	1	0	1	0	0	2	0
0	0	0	0	0	0	2	0	0	0	0
0	0	0	3	0	0	2	0	0	4	0
0	0	0	1	0	0	1	0	0	0	0
0	0	0	0	0	0	0	0	0	3	0
0	0	0	1	0	0	0	0	0	0	0
0	0	0	2	0	0	1	0	0	2	0
0	0	0	0	0	0	1	0	0	1	0
0	0	0	0	1	0	1	0	0	0	0
0	0	0	0	0	0	0	0	0	1	0
1	0	0	0	1	0	1	0	1	20	0
0	0	0	0	0	0	1	0	0	0	0
0	0	0	0	1	0	0	0	0	0	0
0	0	0	0	1	0	1	0	0	0	0
0	0	0	0	0	0	0	0	0	1	0
1	0	0	0	0	0	0	0	0	0	0
0	0	0	0	0	0	0	0	0	1	0
0	0	0	0	0	0	0	0	0	0	0
0	0	0	0	1	0	0	0	0	16	0

0	0	0	0	2	0	0	0	0	12	0
1	0	0	0	4	0	0	0	0	0	0
0	0	0	0	1	0	0	0	0	16	0
0	0	0	0	12	0	0	0	0	1	0
0	0	0	0	1	0	1	0	0	0	0
0	0	0	0	3	0	0	0	0	0	0
0	0	0	1	4	0	0	0	0	1	0
0	0	0	0	2	0	1	0	1	0	0
0	0	0	0	7	0	0	0	2	1	0
0	0	0	0	0	0	0	0	1	0	0
0	0	0	0	4	0	0	0	0	0	0
0	0	0	0	0	0	0	0	0	1	0
0	0	0	0	1	0	0	0	0	0	0
0	0	0	0	2	0	0	0	0	0	0
0	0	0	0	0	0	0	0	0	0	0
0	0	0	0	26	0	0	0	0	0	0
0	0	0	0	33	0	0	0	1	0	0
0	0	0	0	11	0	0	0	0	0	0
0	0	0	0	0	0	0	0	0	0	0
0	0	0	0	0	0	0	0	0	0	0
0	0	0	0	0	0	0	0	0	1	0
0	0	0	0	22	0	0	0	0	0	0
0	0	0	0	0	0	0	0	0	0	0
0	0	0	0	0	0	0	0	0	0	0
0	0	0	0	0	0	1	0	0	0	0
0	0	0	0	0	0	0	0	0	35	0
0	0	0	0	0	0	0	0	0	0	0
0	0	0	0	0	0	0	0	0	29	1
0	0	0	0	6	0	0	0	0	0	0

0	0	0	1	3	0	0	0	0	1	0
0	0	0	0	3	0	0	0	0	1	0
0	0	0	0	5	0	0	0	0	0	0
0	0	0	0	6	0	0	0	0	0	0
1	0	0	0	7	0	0	0	0	0	0
0	0	0	0	1	0	0	0	0	0	0
0	0	0	0	1	0	0	0	0	3	0
0	0	0	0	3	0	0	0	0	0	0
0	0	0	0	3	0	0	0	0	0	0
0	0	0	0	2	0	0	0	0	0	0
1	0	0	0	1	0	0	0	0	2	0
0	0	0	0	3	0	0	0	0	0	0
0	0	0	2	0	0	0	0	0	4	0
3	0	0	0	1	0	1	1	0	1	0
3	0	0	0	3	0	0	0	0	6	0
1	0	0	0	0	0	0	0	0	1	0
0	0	0	0	0	0	0	0	0	0	0
0	0	0	1	1	0	0	0	0	0	0
1	0	0	0	0	0	0	0	1	0	0
0	0	0	1	0	0	0	0	0	0	0
0	0	0	0	0	0	0	0	1	0	0
1	0	0	0	0	0	0	0	1	1	0
0	0	0	0	0	0	0	0	0	0	0
0	0	0	0	2	0	0	0	3	0	0
0	0	0	0	6	0	0	0	0	0	0
0	0	0	0	1	0	0	0	0	0	0
3	0	0	0	0	0	0	0	0	0	0
0	0	0	0	1	0	0	0	0	0	0
2	0	0	0	0	0	0	0	0	0	0

0	0	0	0	0	0	0	0	0	0	0
0	0	0	0	0	0	0	0	0	0	0
0	0	0	0	0	0	0	0	0	0	0
0	0	0	0	1	0	0	0	0	0	0
0	0	0	0	0	0	0	0	0	1	0
0	0	0	0	12	0	0	0	0	0	0
0	0	0	0	0	0	0	0	0	0	0
0	0	0	0	0	0	0	0	0	0	0
0	0	0	0	0	0	0	0	0	0	0
8	0	0	1	0	0	0	0	0	0	0
0	0	0	0	0	0	0	0	0	2	0
0	0	0	1	1	0	1	0	0	1	0
0	0	0	0	3	0	0	0	0	0	0
0	0	0	1	2	0	0	0	0	0	0
0	0	0	0	0	0	0	0	0	0	0
0	0	0	0	1	0	0	0	0	0	0
0	0	0	0	0	0	0	0	0	0	0
0	0	0	0	11	0	0	0	0	0	0
0	0	0	0	3	0	0	0	0	0	0
2	0	0	0	11	0	0	0	0	0	0
0	0	0	0	1	0	0	0	0	1	0
0	0	0	0	2	0	1	0	0	2	0
0	0	0	0	0	0	0	0	0	1	0
0	0	0	0	0	0	0	0	0	1	0
0	0	0	0	0	0	0	0	0	0	0
0	1	0	0	1	0	0	0	0	0	0
0	0	0	0	0	0	1	0	0	1	0
0	0	0	0	0	0	0	0	0	4	0
0	0	0	0	0	0	1	0	0	0	0

0	0	0	0	0	0	0	0	0	3	0
0	0	0	0	1	0	0	0	0	2	0
0	0	0	0	0	0	0	0	0	6	0
0	0	0	0	0	0	4	0	0	16	0
0	0	0	1	0	0	0	0	0	0	0
0	0	0	0	0	0	0	0	0	0	0
0	0	0	0	0	0	0	0	0	18	0
0	0	0	0	0	0	0	0	0	17	0
0	0	0	0	0	0	0	0	0	17	0
0	0	0	1	0	0	0	0	0	5	0
0	0	0	1	0	0	0	0	0	3	0
0	0	0	1	0	0	0	0	0	1	0
0	0	0	0	0	0	3	0	0	1	0
1	0	0	3	0	0	5	0	0	7	0
0	0	0	0	0	0	0	0	0	0	0
0	0	0	3	0	0	1	0	0	7	0
0	0	0	5	0	0	1	0	0	8	0
0	0	0	2	0	0	3	0	0	0	0
0	0	0	4	0	0	1	0	0	0	0
0	0	0	6	0	0	5	0	0	0	0
0	0	0	5	0	0	6	0	0	0	0
0	0	0	2	0	0	9	0	0	0	0
0	0	0	6	0	0	7	0	0	5	0
0	0	0	1	0	0	15	0	0	1	0
0	0	0	12	0	0	7	0	0	7	0
0	0	0	2	0	0	9	0	0	3	0
0	0	0	7	0	0	22	0	0	0	0
0	0	0	3	0	0	23	0	0	1	0
0	0	0	3	0	0	28	0	0	0	0

1	0	0	2	0	0	25	0	0	0	0
0	0	0	2	0	0	47	0	0	0	0
0	0	0	0	0	0	35	0	0	4	0
0	0	0	2	0	0	22	0	0	0	0
0	0	0	0	0	0	24	0	0	3	0
0	0	0	1	0	0	31	0	0	0	0
0	0	0	3	0	0	22	0	0	0	0
0	0	0	1	0	0	16	0	0	1	0
0	0	0	0	0	0	17	0	0	0	0
0	0	0	0	0	0	18	0	0	1	0
0	0	0	0	0	0	27	0	0	0	0
0	0	0	0	0	0	19	0	0	0	0
0	0	0	0	0	0	20	0	0	11	0
0	0	0	1	0	0	14	0	0	1	0
0	0	0	0	0	0	11	1	0	0	0
0	0	0	0	0	0	0	0	0	0	0
0	0	0	1	0	0	21	0	0	0	0
0	0	0	0	0	0	21	8	0	2	0
0	0	0	0	0	0	31	0	0	2	0
0	0	0	0	0	0	0	8	0	11	0
0	0	0	0	0	0	35	0	0	7	0
1	0	0	0	0	0	0	0	0	2	0
0	0	0	0	0	0	8	0	0	0	0

paramp	parlyr	parnou	pecaus	perval	phiaus	pholoe	phoron	phoxoc	pinnov	plaaus
0	0	0	0	0	0	0	0	0	0	0
0	0	3	0	0	0	0	0	0	0	0
0	0	0	0	0	0	0	0	0	0	0
0	0	1	0	0	0	0	0	0	0	0

0	0	1	0	0	0	0	0	0	0	0
0	0	4	0	0	0	0	0	0	0	0
0	0	1	0	0	0	0	0	0	0	0
0	0	4	0	0	0	0	0	0	0	0
0	0	0	0	0	0	0	0	0	0	0
0	0	0	0	0	0	0	0	0	0	0
0	0	0	0	0	0	0	0	0	0	0
0	2	0	0	0	0	0	0	0	0	0
0	1	0	0	0	0	0	0	0	0	0
0	2	0	0	0	0	0	0	0	0	0
0	3	0	0	0	0	1	0	0	0	0
0	0	0	0	0	0	0	0	0	0	0
0	2	0	0	0	0	0	0	0	0	0
0	0	0	0	0	0	0	0	0	0	2
0	0	0	0	0	0	0	0	0	0	0
0	0	0	0	0	0	0	1	0	0	0
0	0	4	0	0	0	0	0	0	0	0
0	0	2	0	1	0	0	0	0	0	0
0	0	3	0	0	0	0	0	0	0	0
0	0	5	0	0	0	0	0	0	0	0
0	0	1	0	0	0	0	0	0	0	0
0	0	0	0	0	0	0	0	0	1	0
0	0	1	0	0	0	0	0	0	0	0
0	0	0	0	0	0	0	0	0	0	0
0	0	1	0	0	0	0	0	0	0	0
0	0	4	0	0	0	0	0	0	0	0
0	0	2	0	0	0	0	0	0	0	0
0	2	5	0	0	0	0	0	0	0	0
0	0	7	0	0	0	0	0	0	0	0

0	0	3	0	0	0	0	0	0	0	0
0	0	2	0	0	1	0	0	0	0	0
0	0	4	0	0	0	0	0	0	0	0
0	0	0	0	0	0	0	0	0	0	0
0	0	2	0	0	0	0	0	0	0	0
0	0	4	0	0	0	0	0	0	0	0
0	0	2	0	0	0	0	0	0	0	0
0	0	3	0	0	0	0	0	0	0	1
0	2	0	0	0	0	0	0	0	1	1
0	0	8	0	0	0	0	0	0	0	0
0	0	0	0	0	0	0	0	0	0	0
0	0	0	0	0	0	0	0	0	0	0
0	0	0	0	0	0	0	0	0	0	0
0	3	1	0	0	0	0	0	0	0	0
0	2	3	0	0	0	0	0	0	0	0
0	0	0	0	0	0	0	0	0	0	0
0	2	0	0	0	0	0	0	0	0	0
0	0	1	0	0	0	0	0	0	0	0
0	2	0	0	0	0	0	0	0	0	0
0	0	0	0	0	2	0	0	0	0	0
0	0	1	0	0	0	0	0	0	0	0
0	4	0	0	0	0	0	0	0	0	0
1	2	0	0	0	0	0	0	0	0	0
0	0	1	0	0	0	0	0	0	0	0
0	0	0	0	0	0	0	0	0	0	0
0	0	0	0	1	0	0	0	0	0	0
0	1	0	0	0	0	0	0	0	0	0
0	0	0	0	2	0	1	0	0	0	0
0	0	0	0	0	0	0	0	0	0	0

0	2	0	0	1	0	0	0	0	0	0
0	0	0	0	0	0	0	0	0	0	0
0	7	0	0	0	0	1	0	0	0	0
0	2	0	0	0	0	0	0	0	0	0
0	0	0	0	0	0	0	0	0	0	0
0	3	0	0	0	0	0	0	0	0	0
0	0	0	0	0	0	0	0	0	0	0
0	0	0	0	0	0	0	0	0	0	0
0	0	0	0	0	0	0	0	0	0	0
0	3	0	0	0	0	0	0	0	0	1
0	0	2	0	0	1	0	0	0	0	0
0	0	4	0	0	0	0	0	0	0	0
0	4	0	0	0	0	0	0	0	0	0
0	0	0	0	0	0	1	0	0	0	0
0	0	1	0	0	0	0	1	0	0	0
0	5	5	0	0	1	0	0	0	0	2
0	0	0	0	0	0	0	0	0	0	1
0	0	0	0	0	0	0	0	0	0	0
0	0	0	0	0	0	0	0	0	0	1
0	0	0	0	0	0	0	0	0	0	0
0	0	1	0	0	0	0	0	0	0	3
0	2	0	0	0	0	0	1	0	0	0
0	0	0	0	0	0	0	0	0	0	0
0	0	1	0	0	0	0	0	0	0	0
0	2	0	0	0	0	0	0	0	0	0
0	0	1	0	0	0	0	0	0	0	0
0	0	0	0	0	1	0	0	0	0	0
0	2	0	0	0	0	0	0	0	0	0
0	0	0	0	0	0	0	0	0	0	3

0	1	2	0	0	0	0	0	0	0	0
0	0	0	0	0	0	0	0	0	0	1
0	0	2	0	0	0	0	0	0	0	0
0	1	3	0	0	0	0	0	0	0	0
0	0	0	0	0	0	0	0	0	0	0
0	0	0	0	0	0	0	0	0	0	0
0	3	0	0	0	0	0	0	0	0	1
0	0	0	0	0	0	0	0	0	0	0
0	0	0	0	0	0	0	0	0	0	2
0	4	0	0	0	0	0	0	0	0	4
0	1	0	0	0	0	0	0	0	0	0
0	3	0	0	0	0	0	0	0	0	1
0	1	0	0	0	0	0	0	0	0	0
0	0	0	0	0	0	0	0	0	0	0
0	1	0	0	0	0	1	0	0	0	1
0	0	0	0	0	0	0	0	0	0	0
0	0	0	0	0	0	0	0	0	0	0
0	1	0	0	0	0	0	0	0	0	0
0	2	0	0	0	0	0	0	0	0	3
0	2	0	0	1	0	0	0	0	0	1
0	0	0	0	0	0	0	0	0	0	0
0	1	0	0	0	0	0	0	0	0	0
0	2	0	0	0	0	0	0	0	0	0
1	1	0	0	0	0	0	0	0	0	2
0	0	0	0	1	0	0	0	0	0	2
0	0	0	0	0	0	0	0	0	0	0
0	0	0	0	0	0	0	0	0	0	2
0	1	0	0	0	0	0	0	0	0	1
0	2	0	0	0	0	0	0	0	0	2

0	1	0	0	0	0	0	0	0	0	4
0	2	1	0	0	0	0	0	0	0	1
0	1	0	0	0	0	0	0	0	0	2
0	0	0	0	0	0	0	0	0	0	2
0	1	0	0	0	0	0	0	0	0	1
0	0	0	0	0	0	0	0	0	0	1
0	1	0	0	0	0	0	0	0	0	1
0	0	0	0	0	0	0	0	0	0	0
0	2	0	0	0	0	0	0	0	0	1
0	0	0	0	0	0	0	0	0	0	0
0	0	0	0	0	0	0	0	0	0	0
0	1	0	0	0	1	0	0	0	0	0
0	0	0	0	0	0	0	0	0	0	0
0	2	0	0	0	0	0	0	0	0	0
0	0	0	0	0	0	0	0	0	0	2
0	7	0	0	0	0	0	0	0	0	0
0	0	0	0	0	0	0	0	0	0	0
0	3	0	0	0	0	0	0	0	0	0
0	0	0	0	0	0	0	0	0	0	1
0	1	0	0	0	0	0	0	0	0	1
0	1	1	0	0	0	0	0	0	0	0
0	2	0	0	1	0	0	0	0	0	0
0	2	0	0	0	0	0	0	0	0	0
0	1	0	0	0	0	0	0	0	0	0
0	1	1	0	0	0	0	0	0	0	0
0	0	0	0	0	0	0	0	0	0	0
0	0	0	0	0	1	0	0	0	0	0
0	0	0	0	0	0	0	0	0	0	0
0	0	0	0	0	0	0	1	0	0	0

0	1	0	0	0	0	0	0	0	0	0
0	1	0	0	0	0	0	0	0	0	0
0	0	0	0	1	0	0	0	0	0	0
0	0	0	0	0	0	0	0	0	0	0
0	0	0	0	0	0	0	0	1	0	2
0	0	0	0	0	1	0	0	0	0	0
0	3	0	0	0	0	0	0	0	0	0
0	3	0	0	0	0	0	0	0	0	0
0	2	0	0	0	0	0	0	0	0	0
0	1	0	0	0	0	0	0	0	0	0
0	4	0	0	0	0	0	0	0	0	2
0	2	0	0	0	0	0	0	0	0	0
0	0	0	0	0	0	0	0	0	0	0
0	6	0	0	0	0	0	0	0	0	2
0	2	0	0	0	0	0	0	0	0	2
0	3	0	0	0	0	0	0	0	0	0
0	0	0	0	0	0	0	0	0	0	0
0	0	0	0	0	0	0	0	0	0	0
0	1	0	0	0	0	0	0	0	0	0
0	1	0	0	0	0	0	0	0	0	0
0	1	0	0	0	0	0	0	0	0	2
0	0	0	0	0	0	0	0	0	0	0
0	6	0	0	0	0	0	0	0	0	1
0	1	0	0	0	1	0	0	0	0	0
0	1	0	0	1	0	0	0	0	0	0
0	5	0	0	0	0	0	0	0	0	1
0	0	0	0	0	0	0	0	0	0	1
0	1	0	0	0	0	0	0	0	0	0
0	0	0	0	0	0	0	0	0	0	0

0	3	0	0	0	0	0	0	0	0	0
0	0	0	0	2	0	0	0	0	0	2
0	3	0	0	0	0	0	0	0	0	0
0	0	0	0	0	0	0	0	0	0	0
0	1	1	0	0	0	0	0	0	0	1
0	1	0	1	0	0	0	0	0	0	0
0	4	0	0	0	0	0	0	0	0	0
0	0	1	0	0	0	0	0	0	0	0
0	0	0	0	0	0	0	0	0	0	0
0	0	0	0	0	0	0	0	0	0	0
0	0	0	0	0	0	0	0	0	0	0
0	0	0	0	0	0	0	0	0	0	0
0	1	0	0	0	0	0	0	0	0	0
0	0	0	0	0	0	0	0	0	0	0
0	0	0	0	0	0	0	0	0	0	0
0	0	0	0	1	0	0	0	0	0	0
0	0	0	0	1	0	0	0	0	0	0
0	4	0	0	0	0	0	0	0	0	0
0	0	0	0	0	0	0	0	0	0	0
0	0	0	0	0	0	0	0	0	0	0
0	0	0	0	0	0	0	0	0	0	0
0	0	0	0	0	0	0	0	0	0	0
0	0	0	0	0	0	0	0	0	0	0
1	0	0	0	1	0	0	0	0	0	0
0	1	1	0	3	0	0	0	0	0	0
0	0	1	0	0	0	0	0	0	0	0
0	1	0	0	0	0	0	0	0	0	0
0	0	0	0	2	0	0	0	0	0	0
0	0	0	0	0	0	0	0	0	0	0

0	0	0	0	0	0	0	0	0	0	0
0	0	0	0	0	0	0	0	0	0	0
0	0	0	0	0	0	0	0	0	0	0
0	0	0	0	0	0	0	0	0	0	0
0	0	0	0	0	0	0	0	0	0	0
0	0	0	0	0	0	0	0	0	0	0
0	1	0	0	2	0	0	0	0	0	0
0	2	0	0	0	0	0	0	0	0	0
0	1	0	0	0	0	0	0	0	0	0
0	0	0	0	0	0	0	0	0	0	0
0	0	0	0	2	0	0	0	0	0	0
0	2	0	0	1	0	0	0	0	0	0
0	2	0	0	3	0	0	0	0	0	0
0	1	0	0	0	0	0	0	0	0	0
0	0	0	0	0	0	0	0	0	0	0
0	0	0	0	0	0	0	0	0	0	0
0	0	0	0	0	0	0	0	0	0	0
0	1	0	0	0	1	0	0	0	0	0
0	0	0	0	0	0	0	0	0	0	0
0	1	0	0	0	0	0	0	0	0	0
0	0	0	0	0	0	0	0	0	0	0
0	0	0	0	0	0	0	0	0	0	0
0	0	0	0	0	0	0	0	0	0	0
0	0	0	0	0	0	0	0	0	0	0
0	0	0	0	0	0	0	0	0	0	0
0	0	0	0	0	0	0	0	0	0	0
0	1	0	0	0	0	0	0	0	0	1
0	0	0	0	0	0	0	0	0	0	0
0	1	0	0	0	0	0	0	0	0	3

0	0	0	0	0	0	0	0	0	0	1
0	0	0	0	0	0	0	0	0	0	0
0	0	0	0	0	0	0	0	0	0	0
0	0	0	0	0	0	0	0	0	0	2
0	0	0	0	0	0	0	0	0	0	0
0	0	0	0	0	0	0	0	0	0	0
0	0	0	0	0	1	0	0	0	0	1
0	0	0	0	1	0	0	0	0	0	0
0	0	1	0	0	0	0	0	0	0	0
0	4	0	0	0	0	0	0	0	0	0
0	0	0	0	0	0	0	0	0	0	0
0	0	0	0	0	0	0	0	0	0	0
0	0	0	0	0	0	0	0	0	0	0
0	0	0	0	0	0	0	0	0	0	0
0	0	0	0	0	0	0	0	0	0	0
0	0	0	0	0	0	0	0	0	0	0
0	0	0	0	0	0	0	0	0	0	2
0	0	0	0	0	0	0	0	0	0	0
0	0	0	0	0	0	0	0	0	0	0
0	1	0	0	0	0	0	2	0	0	0
0	0	0	0	0	0	0	1	0	0	0
0	0	0	0	0	0	0	1	0	0	1
0	0	0	0	0	0	0	0	0	0	0
0	0	0	0	0	0	0	0	0	0	0
0	4	0	0	0	0	0	0	0	0	0
0	0	0	0	0	0	0	0	0	0	0
0	3	0	0	0	0	0	1	0	0	0
0	1	0	0	0	0	0	0	0	0	0
0	2	0	0	0	0	0	0	0	0	2

0	0	0	0	0	0	0	0	0	0	0
0	0	0	0	0	0	0	0	0	0	0
0	1	0	0	0	0	0	0	0	0	0
0	0	0	0	0	0	0	0	0	0	0
0	0	0	0	0	0	0	0	0	0	0
0	1	0	0	0	0	0	0	0	0	0
0	3	0	0	0	0	0	0	0	0	1
0	1	0	0	0	0	0	0	0	0	0
0	0	0	0	0	0	0	0	0	0	0
0	1	0	0	0	0	0	0	0	0	3
0	0	0	1	0	0	0	1	0	0	0
0	1	0	0	0	2	0	1	0	0	3
0	0	0	0	0	0	0	0	0	0	1
0	0	0	0	0	0	0	0	0	0	2
0	0	0	0	0	0	0	0	0	0	0
0	0	0	0	0	0	0	1	0	0	1
0	1	0	0	0	0	0	0	0	0	1
0	0	0	0	0	1	0	0	0	0	5
0	0	0	0	0	1	0	0	0	0	1
0	1	0	0	0	0	0	1	0	0	1
0	0	0	0	0	0	0	0	0	0	1
0	0	0	0	0	0	0	0	0	0	0
0	2	0	1	0	0	0	0	0	0	2
0	1	0	0	0	1	0	0	0	0	0
0	1	0	0	0	0	0	0	0	0	0
0	1	0	1	0	0	0	1	0	0	0
0	4	0	0	0	0	0	0	0	0	0
0	0	0	0	0	0	0	0	0	0	0
0	2	1	0	0	0	0	0	0	0	6

0	1	0	0	0	0	0	1	0	0	1
0	1	0	0	0	0	0	0	0	0	3
0	1	0	0	0	0	0	0	0	0	1
0	1	0	0	0	0	0	0	0	0	1
0	3	0	0	0	0	0	0	0	0	0
0	3	0	0	0	0	0	0	0	0	1
0	0	0	0	0	0	0	0	0	0	0
0	1	0	0	0	0	0	0	0	0	0
0	0	0	0	1	0	0	0	0	0	0
0	0	0	0	0	0	0	0	0	0	4
0	0	0	0	0	0	0	0	0	0	0
0	1	0	0	0	0	0	0	0	0	0
0	0	0	0	0	0	0	0	0	0	1
0	0	0	0	0	0	0	0	0	0	0
0	0	0	0	0	0	0	0	0	0	0
0	0	0	0	0	0	0	0	0	0	0
0	0	0	0	0	0	0	0	0	0	1
0	2	0	0	0	0	0	0	0	0	0
0	0	0	0	0	0	0	0	0	0	0
0	0	0	0	0	0	0	0	0	0	0
0	2	0	0	0	0	0	0	0	0	0
0	0	0	0	0	0	0	0	0	0	0
0	0	0	0	0	0	0	0	0	0	0
0	0	0	0	0	0	0	1	0	0	0
0	0	0	0	0	0	0	0	0	0	1
0	2	0	0	0	0	0	1	0	0	0
0	0	0	0	0	0	0	0	0	0	0
0	0	0	0	0	0	0	0	0	0	1
0	0	0	0	0	0	0	0	0	0	0

0	0	0	0	0	0	0	0	0	0	1
0	3	0	0	0	0	0	0	0	0	2
0	1	0	0	0	0	0	0	0	0	5
0	1	0	0	0	0	0	0	0	0	1
0	2	0	0	0	0	0	0	0	0	0
0	0	0	0	0	0	0	1	0	0	1
0	0	0	0	0	0	0	0	0	0	2
0	0	0	0	0	1	0	1	0	0	1
0	0	0	0	0	0	0	1	0	0	0
0	0	0	0	0	0	0	1	0	0	2
0	0	0	0	0	1	0	0	0	0	0
0	0	0	0	0	0	0	0	0	0	0
0	0	0	0	0	0	0	0	0	0	2
0	1	0	0	0	0	0	0	0	0	0
0	0	0	0	0	1	0	0	0	0	1
0	1	0	0	0	0	0	1	0	1	2
0	1	0	0	0	0	0	0	0	0	7
0	0	0	0	0	0	0	1	0	0	0
0	0	1	0	0	0	0	1	0	0	0
0	4	0	0	0	0	0	0	0	0	0
0	1	0	0	0	1	0	0	0	0	2
0	0	0	0	0	1	0	0	0	0	1
0	0	0	0	0	0	0	0	0	0	0
0	0	0	0	0	0	0	0	0	0	1
0	0	0	0	0	0	0	0	0	0	0
0	0	0	0	0	0	0	0	0	0	0
0	0	0	0	0	0	0	1	0	0	5
0	0	0	0	0	1	0	0	0	0	0
0	1	0	0	0	0	0	0	0	0	0

0	1	0	0	0	0	0	0	0	0	0
0	0	0	0	0	2	0	1	0	0	1
0	0	0	0	0	0	0	0	0	0	0
0	0	0	0	0	0	0	0	0	0	0
0	0	0	0	0	1	0	0	0	0	0
0	0	0	0	0	0	0	0	0	0	0
0	0	0	0	0	2	0	0	0	0	0
0	0	1	0	0	0	0	0	0	0	0
0	1	1	0	0	0	0	0	0	0	2
0	1	0	0	0	0	0	0	0	0	0
0	0	0	0	0	0	0	0	0	0	0
0	0	0	0	0	0	0	0	0	0	0
0	0	0	0	0	1	0	1	0	0	1
0	0	0	0	0	0	0	0	0	0	0
0	0	0	0	0	0	0	0	0	0	0
0	0	0	0	0	0	0	0	0	0	0
0	0	0	0	0	0	0	0	0	0	0
0	0	0	0	0	0	0	0	0	0	1
0	0	0	0	0	0	0	0	0	0	0
0	1	0	0	0	0	0	0	0	0	0
0	0	0	0	0	0	0	0	0	0	0
0	0	0	0	0	0	0	0	0	0	0
0	0	0	0	0	0	0	0	0	0	1
0	1	1	0	0	0	0	0	0	0	0
0	0	0	0	0	0	0	1	0	0	0
0	0	0	0	0	0	0	0	0	0	2
0	1	0	0	0	0	0	0	0	0	0
0	0	0	0	0	0	0	0	0	0	0
0	0	0	0	0	0	0	0	0	0	0

0	0	0	0	0	0	0	0	0	0	0
0	0	0	0	0	0	0	0	0	0	3
0	0	0	0	1	0	0	0	0	0	0
0	0	0	0	0	0	0	0	0	0	0
0	0	0	0	0	0	0	0	0	0	0
0	0	0	0	1	0	0	0	0	0	0
0	0	0	0	0	0	0	0	0	0	0
0	0	0	0	0	1	0	0	0	0	0
0	0	0	0	0	0	0	0	0	0	0
0	1	0	0	0	0	0	0	0	0	0
0	0	0	0	0	0	0	0	0	0	0
0	0	0	0	1	0	0	0	0	0	0
0	0	0	0	0	0	0	0	0	0	0
0	0	0	0	0	0	0	0	0	0	0
0	0	0	0	1	0	0	0	0	0	0
0	0	0	0	0	0	0	0	0	0	2
0	0	0	0	1	0	0	0	0	0	0
0	0	0	0	0	0	0	0	0	0	0
0	0	0	0	1	0	0	0	0	0	0

spion	syllid	terebe	torhur	zealut	zeasub	zoeasp
0	0	0	0	0	0	0
0	0	0	0	0	0	0
0	0	0	0	0	0	0
0	0	0	0	0	1	0
0	0	0	0	1	0	0
0	0	0	0	0	7	0
0	0	0	0	0	0	0
0	0	0	0	0	0	0

0	0	0	0	1	10	0
0	0	0	0	0	1	0
0	0	0	0	0	0	0
0	0	0	0	0	1	0
0	0	0	0	0	0	0
0	0	0	0	0	1	0
0	0	0	0	0	2	0
0	0	0	0	0	4	0
0	0	0	0	0	2	0
0	0	1	0	0	5	1
0	0	0	0	0	0	0
0	1	0	0	0	0	0
0	0	0	0	0	0	0
0	0	0	0	0	0	0
0	0	0	0	0	3	0
0	0	0	0	0	0	0
0	0	0	0	1	0	0
0	0	0	0	0	0	0
0	0	0	0	0	0	0
0	0	0	0	0	0	0
0	0	0	0	2	0	0
0	0	0	0	0	0	0
0	0	0	0	0	0	0
0	0	0	0	2	0	0
0	0	0	0	0	0	0
0	0	0	0	0	0	0
0	0	0	0	0	0	0
0	0	0	0	0	0	0
0	0	0	0	0	0	0

0	0	0	0	0	0	0
0	0	0	0	0	0	0
0	0	0	0	0	0	0
0	0	0	0	0	0	0
0	0	0	0	0	0	0
0	0	0	0	2	0	0
0	0	0	0	0	0	0
0	0	0	0	0	0	0
0	0	0	0	0	0	0
0	0	0	0	0	0	0
0	0	0	0	2	0	0
0	0	0	0	0	0	0
0	0	0	0	5	0	0
0	0	0	0	1	1	0
0	0	0	0	0	0	0
0	0	0	0	2	0	0
0	0	0	0	0	0	0
0	0	0	0	1	0	0
0	0	0	0	0	0	0
0	0	0	0	0	0	0
0	0	0	0	0	0	0
0	0	0	0	0	0	0
0	0	0	0	3	0	1
0	0	0	0	0	0	0
0	0	0	0	2	2	0
0	0	0	0	1	0	0
0	0	0	0	0	0	0
0	0	0	0	0	0	0
0	0	0	0	0	0	0

0	0	0	0	0	0	0
0	0	0	0	0	0	0
0	0	0	0	1	1	0
0	0	0	0	0	0	0
0	0	0	0	0	0	0
0	0	0	0	0	0	0
0	0	0	0	1	1	0
0	0	0	0	1	0	0
0	0	0	0	0	0	0
0	0	0	0	0	1	0
0	0	0	0	0	0	0
0	0	0	0	0	0	0
0	0	0	0	0	0	0
0	0	0	0	0	0	0
0	0	0	0	0	0	1
0	0	0	0	0	0	0
0	0	0	0	0	0	0
0	0	0	0	0	0	0
0	0	0	0	0	1	0
0	0	0	0	0	0	0
0	0	0	0	0	0	0
0	0	0	0	0	0	0
0	0	0	0	0	1	0
0	0	0	0	0	0	0
0	0	0	0	0	0	0
0	0	0	0	0	1	0
0	0	0	0	0	0	0
0	0	0	0	0	0	0
0	0	0	0	0	0	0

0	0	2	0	0	0	0
0	0	0	0	0	0	0
0	0	0	0	0	0	1
0	0	0	0	0	0	0
0	0	0	0	0	0	0
0	1	0	0	0	0	0
0	0	0	0	2	1	1
0	0	1	0	0	1	0
0	1	1	0	0	0	0
0	0	0	1	0	0	0
0	0	0	0	0	6	0
0	0	0	0	0	1	0
0	0	2	0	0	0	0
0	0	1	0	0	3	0
0	0	0	0	0	0	0
0	0	1	0	1	3	0
0	0	0	0	0	0	0
0	0	0	0	2	3	0
0	0	0	0	0	6	0
0	0	0	0	0	0	0
0	0	0	0	0	0	0
0	0	0	0	0	0	0
0	0	0	0	1	0	0
0	0	0	0	1	3	0
0	0	0	0	0	3	0
0	0	0	0	1	0	0
0	0	0	0	0	2	0
0	0	0	0	0	0	0
0	0	0	0	0	0	0

0	0	0	0	0	0	0
0	0	0	0	0	0	0
0	0	0	0	0	1	0
0	0	0	0	0	0	0
0	0	0	0	0	0	0
0	0	0	0	1	0	0
0	0	0	0	0	0	0
0	0	0	0	0	0	0
0	0	0	0	0	0	0
0	0	0	0	0	0	0
0	0	0	0	0	0	0
0	0	0	0	0	0	0
0	0	0	0	0	0	0
0	0	0	0	1	0	0
0	0	0	0	0	0	0
0	0	0	0	0	0	0
0	0	0	0	0	0	0
0	0	0	0	0	0	0
0	0	0	0	0	0	0
0	0	0	0	0	0	0
0	0	0	0	0	0	0
0	0	0	0	0	0	0
0	0	0	0	3	0	0
0	0	0	0	0	0	0
0	0	0	0	0	0	0
0	0	0	0	0	0	0
0	0	0	0	0	0	0
0	0	0	0	0	0	0
0	0	0	0	0	0	0

0	0	0	0	0	0	0
0	0	0	0	0	0	0
0	0	0	0	1	0	0
0	0	0	0	0	0	0
0	0	0	0	0	0	0
0	0	0	0	0	0	0
0	0	0	0	0	0	0
0	0	0	0	0	0	0
0	0	0	0	0	0	0
0	0	0	0	0	0	0
0	0	0	0	0	0	0
0	0	0	0	0	0	0
0	0	0	0	0	0	0
0	0	0	0	0	0	1
0	0	0	0	2	0	0
0	0	0	0	0	0	0
0	0	0	0	0	0	0
0	0	0	0	0	0	0
0	0	0	0	0	0	0
0	0	0	0	0	0	0
0	0	0	0	0	0	0
0	0	0	0	1	0	0
0	0	0	0	0	0	0
0	0	0	0	0	0	0
0	0	0	0	0	0	0
0	0	0	0	0	0	0
0	0	0	0	0	0	0
0	0	0	0	0	0	0
0	0	0	0	0	0	0

0	0	0	0	2	0	0
0	0	0	0	0	0	0
0	0	0	0	0	0	0
0	0	0	0	0	0	0
0	0	0	0	2	0	0
0	0	0	0	0	0	0
0	0	2	0	0	4	0
0	0	0	0	1	3	0
0	0	0	0	0	1	0
0	0	0	0	3	0	0
0	0	0	0	1	3	0
0	0	0	0	0	4	0
0	0	0	0	0	3	0
0	0	0	0	0	1	0
0	0	0	0	1	1	0
0	0	0	0	2	1	0
0	0	0	0	1	0	0
0	0	0	0	0	0	0
0	0	0	0	0	0	0
0	0	0	0	0	0	0
0	0	0	0	0	0	0
0	0	0	0	1	0	0
0	0	0	0	0	0	1
0	0	0	0	0	0	0
0	0	0	0	0	1	0
0	0	0	0	0	1	0
0	0	0	0	0	0	0
0	0	0	0	0	1	0
0	0	0	0	0	0	0

0	0	0	0	0	0	0
0	0	0	0	0	0	0
0	0	0	0	0	0	0
0	0	0	0	1	1	1
0	0	0	0	0	0	0
0	0	0	0	0	0	0
1	0	0	0	0	0	0
0	0	0	0	0	0	0
0	0	0	0	1	0	0
0	0	0	0	0	0	0
0	0	1	0	0	0	0
0	0	0	0	0	0	0
0	0	0	0	0	0	0
0	0	0	0	0	0	0
0	0	0	0	2	0	0
0	0	0	1	0	0	0
0	0	0	0	1	0	0
0	0	0	0	0	0	0
0	0	1	0	0	0	0
0	0	0	0	0	0	0
0	0	0	0	0	0	0
0	0	0	0	0	0	0
0	0	0	0	0	0	0
0	0	0	0	0	0	0
0	0	0	0	0	0	0
0	0	0	0	1	0	0
0	0	0	0	0	0	0
0	0	0	4	0	0	0
0	0	0	0	0	0	0

0	0	0	0	1	0	0
0	0	0	0	0	0	0
0	0	0	0	0	0	0
0	0	0	0	0	0	0
0	0	0	0	0	0	0
0	0	0	0	0	0	0
0	0	0	0	0	0	0
0	0	0	0	0	0	0
0	0	0	0	0	0	0
0	0	0	0	0	0	0
0	0	0	0	0	0	0
0	0	0	0	0	0	0
0	0	0	0	0	0	0
0	0	0	0	0	0	0
0	0	0	0	0	0	0
0	0	0	0	0	0	0
0	0	0	0	0	0	0
0	0	0	0	0	0	0
0	0	0	0	0	0	0
0	0	0	0	0	0	0
0	0	0	0	0	0	0
0	0	0	0	0	0	0
0	0	0	0	0	0	0
0	0	0	0	0	1	0
0	0	0	0	0	0	0
0	0	0	0	0	0	0
0	0	0	0	0	0	0
0	0	0	0	0	1	0
0	0	0	0	0	0	0

0	0	0	0	0	0	0
0	0	0	0	0	0	0
0	0	0	0	0	0	0
0	0	0	0	0	0	0
0	0	0	0	1	0	0
0	0	0	0	0	0	0
0	0	0	0	0	0	0
0	0	0	0	0	0	0
0	0	0	0	0	0	0
0	0	0	0	0	0	0
0	0	0	0	0	0	0
0	0	0	0	0	0	0
0	0	1	0	0	0	0
0	0	0	0	0	1	0
0	0	0	0	0	3	0
0	0	0	0	0	0	0
0	0	0	0	0	1	0
0	0	1	0	2	1	0
0	0	3	0	0	1	0
0	0	0	1	0	0	0
0	0	2	0	0	0	0
0	0	0	0	0	0	0
0	0	0	0	0	1	0
0	0	1	0	0	0	0
0	0	0	0	1	4	0
0	0	2	0	0	4	0
0	0	0	0	0	0	0
0	0	0	0	0	0	0
0	0	0	0	0	0	0

0	0	0	1	0	2	0
0	0	0	1	0	1	0
0	0	0	0	0	0	0
0	0	0	0	0	3	0
0	0	0	0	0	2	0
0	0	0	0	0	1	0
0	0	0	1	0	3	1
0	0	0	0	1	1	0
0	0	0	0	0	4	0
0	0	0	0	0	1	0
0	0	0	0	0	0	0
0	0	0	0	0	0	0
0	0	0	0	0	1	0
0	0	0	0	0	0	0
0	0	0	0	0	0	0
0	0	0	0	0	0	0
0	0	0	0	0	0	0
0	0	0	0	0	0	0
0	0	0	0	0	1	0
0	0	0	0	0	0	0
0	0	0	0	0	0	0
0	0	0	0	0	0	0
0	0	0	0	0	0	0
0	0	0	0	0	0	0
0	0	0	0	1	0	0
0	0	0	0	0	0	0
0	0	0	0	0	0	0
0	0	0	0	0	0	0
0	0	0	0	1	0	0

0	0	0	0	0	0	0
0	0	0	0	0	0	0
0	0	0	0	0	0	0
0	0	0	0	0	0	0
0	0	0	0	0	0	0
0	0	0	0	0	0	0
0	0	0	0	0	0	0
0	0	0	0	0	0	0
0	0	0	0	0	0	0
0	0	0	0	0	0	0
0	0	0	0	0	0	0
0	0	0	0	0	0	0
0	0	0	0	0	0	0
0	0	0	0	0	0	0
0	0	0	0	0	0	0
0	0	0	0	0	0	0
0	0	0	0	0	0	0
0	0	0	0	0	0	0
0	0	0	0	0	0	0
0	0	0	0	0	0	0
0	0	0	0	0	0	0
0	0	0	0	0	0	0
0	0	0	0	1	0	0
0	0	0	0	1	0	0
0	0	0	0	0	0	0
0	0	0	0	0	0	0
0	0	0	0	1	0	0
0	0	0	0	0	0	0
0	0	0	0	0	0	0

0	0	0	0	0	0	0
0	0	0	0	3	0	0
0	0	0	0	1	0	0
0	0	0	0	0	0	0
0	0	0	0	1	0	0
0	0	0	0	0	0	0
0	0	0	0	0	0	0
0	0	0	0	0	0	0
0	0	0	0	0	0	0
0	0	0	0	2	0	0
0	0	0	0	3	0	0
0	0	3	0	0	0	0
0	0	2	0	0	0	0
0	0	2	0	0	0	0
0	0	1	0	0	0	0
0	0	3	0	0	0	0
0	0	2	0	0	0	0
0	0	0	0	1	1	0
0	0	0	0	0	0	0
0	0	2	1	0	0	0
0	0	0	0	0	0	0
0	0	1	0	0	0	0
0	0	1	0	0	0	0
0	0	2	0	0	0	0
0	0	1	0	0	0	0
0	0	2	0	1	0	0
0	0	0	0	0	0	0
0	0	0	0	0	0	0
0	0	0	0	0	0	0

0	0	0	1	0	0	0
0	0	0	0	2	0	0
0	0	0	0	0	0	0
0	0	0	0	0	0	0
0	0	0	0	4	0	0
0	0	0	0	0	0	0
0	0	0	0	2	0	0
0	0	0	0	0	0	0
0	0	0	0	0	0	0
0	0	0	0	0	0	0
0	0	0	0	0	0	0
0	0	0	0	0	0	0
0	0	0	0	1	0	0
0	0	0	0	0	0	0

pecaus	perval	phiaus	pholoe	phoron	phoxoc	phyllo	pinnov	plaaus	potest	priauc
0	0	0	0	0	0	0	0	0	0	1
0	0	0	0	0	0	0	0	0	0	16
0	0	0	0	0	0	0	0	0	0	5
0	0	0	0	0	0	0	0	0	0	9
0	0	0	0	0	0	0	0	0	0	3
0	0	0	0	0	0	0	0	0	0	1
0	0	0	0	0	0	0	0	0	0	2
0	0	0	0	0	0	0	0	0	0	2
0	0	0	0	0	0	0	0	0	0	0
0	0	0	0	0	0	0	0	0	0	0
0	0	0	0	1	0	0	0	0	0	0
0	0	0	0	2	0	0	0	0	0	0
0	0	0	0	2	0	0	0	0	0	0
0	0	0	0	0	0	0	0	0	0	0
0	0	0	0	0	0	0	0	0	0	0
0	0	0	0	1	0	0	0	0	0	0
0	0	0	0	1	0	0	0	0	0	0
0	0	0	0	0	0	0	0	0	0	0
0	0	0	0	3	0	0	0	0	0	0
0	0	0	0	2	0	0	0	1	0	0
0	0	0	0	1	0	0	0	0	0	0
0	0	0	0	3	0	0	0	0	0	0
0	0	0	0	0	0	0	0	0	0	5
0	0	0	0	0	0	0	0	0	0	11
0	0	0	0	0	0	0	0	2	0	12
0	0	0	0	0	0	0	0	0	0	0
0	0	0	0	1	0	0	0	0	0	0
0	0	1	0	6	0	0	0	0	0	0

0	0	0	0	0	0	0	0	0	0	0
0	0	0	0	0	0	0	0	0	0	0
0	0	0	0	1	0	0	0	0	0	0
0	0	0	0	0	0	0	0	0	0	1
0	0	0	0	0	0	0	0	1	0	1
0	0	0	0	1	0	0	0	0	0	0
0	0	0	0	0	0	0	0	0	0	0
0	0	0	0	2	0	0	0	1	0	0
0	0	0	0	0	0	0	0	0	0	4
0	0	0	0	0	4	0	0	0	0	0
0	0	0	0	0	0	0	0	1	0	1
0	0	1	0	0	0	0	0	0	0	0
0	0	0	0	0	0	0	0	0	0	0
0	0	0	0	1	0	0	0	0	0	0
0	2	0	0	0	0	0	0	0	0	1
0	0	0	0	0	0	0	0	0	0	1
0	0	0	0	0	0	0	0	1	0	4
0	0	0	0	0	0	0	0	1	0	7
0	0	1	0	0	2	0	0	1	0	2
0	0	0	0	0	0	0	0	1	0	3
0	0	1	0	0	0	0	0	0	0	1
0	0	0	0	3	0	0	0	0	0	0
0	0	0	0	2	0	0	0	0	0	0
0	0	0	0	2	0	0	0	0	0	0
0	0	0	0	2	0	0	0	0	0	0
0	0	0	0	2	0	0	0	0	0	0
0	0	0	0	3	0	0	0	0	0	1
0	0	1	0	2	0	0	0	0	0	0
0	0	0	0	2	0	0	0	0	0	1

0	0	0	0	0	0	0	0	0	0	0
0	0	0	0	8	0	0	0	0	0	0
0	0	0	0	0	0	0	0	0	0	0
0	0	0	0	0	0	0	0	0	0	0
0	0	0	0	0	0	0	0	0	0	0
0	0	0	0	3	0	0	0	0	0	0
0	0	0	0	4	0	0	0	0	0	1
0	0	0	0	10	2	0	0	1	0	0
0	0	0	0	3	0	0	0	1	0	0
0	0	0	0	0	0	0	0	0	0	0
0	0	0	0	2	0	0	0	1	0	0
0	0	1	0	3	0	0	0	0	0	0
0	0	0	0	4	0	0	0	0	0	0
0	0	0	0	10	0	0	0	0	0	0
0	0	2	0	5	0	0	0	0	0	0
0	0	0	0	5	0	0	0	0	0	1
0	0	0	0	5	0	0	0	0	0	0
0	0	0	0	3	0	0	0	0	0	0
0	0	0	0	1	0	0	0	0	0	0
0	0	0	0	1	0	0	0	0	0	0
0	0	0	0	2	0	0	0	0	0	1
0	0	0	0	0	0	0	0	0	0	0
0	0	0	0	0	0	0	0	0	0	3
0	0	0	0	0	0	0	0	0	0	2
0	0	0	0	15	0	0	0	0	0	0
0	0	0	0	3	0	0	0	0	0	0
0	0	0	0	15	0	0	0	0	0	0
0	0	0	0	4	0	0	0	0	0	0
0	0	0	0	0	0	0	0	4	0	1

0	0	0	0	3	0	0	0	2	0	0
0	0	0	0	2	0	0	0	1	0	0
0	0	0	0	3	0	0	0	2	0	0
1	0	0	0	3	0	0	0	2	0	0
0	0	0	0	0	0	0	0	0	0	1
0	0	0	0	0	0	0	0	0	0	1
0	0	0	0	0	0	0	0	0	0	1
0	0	0	0	0	1	0	0	0	0	0
0	0	0	0	9	0	0	0	0	0	0
0	0	0	0	3	0	0	0	0	0	0
0	0	1	0	6	0	0	0	0	0	0
0	0	0	0	5	0	0	0	0	0	0
0	0	0	0	12	0	0	0	0	0	0
0	0	0	0	0	0	0	0	0	0	0
0	0	0	0	0	0	0	0	0	0	8
0	0	0	0	0	0	0	0	0	0	0
0	1	0	0	0	0	0	0	0	0	0
0	0	0	0	0	0	0	0	0	0	1
0	0	0	0	0	0	0	0	0	0	0
0	0	0	0	0	0	0	0	1	0	1
0	0	0	0	0	0	0	0	0	0	0
0	0	0	0	3	0	0	0	0	0	0
0	0	0	0	2	0	0	0	1	0	0
0	0	0	0	2	0	0	0	2	0	0
0	0	0	0	2	0	0	0	9	0	0
0	0	0	0	2	0	0	0	1	0	0
0	0	0	0	5	0	0	0	0	0	0
0	0	0	0	2	0	0	0	0	0	0
0	0	0	0	12	0	0	0	2	0	0

0	0	0	0	5	0	0	0	1	0	0
0	0	0	0	0	0	0	0	0	0	6
0	0	0	0	0	0	0	0	0	0	12
0	0	0	0	0	0	0	0	0	0	7
0	0	0	0	0	0	0	0	0	0	12
0	0	0	0	0	0	0	0	0	0	8
0	0	0	0	0	0	0	0	0	0	2
0	0	0	0	0	0	0	0	0	0	7
0	0	0	0	0	0	0	0	0	0	9
0	0	0	0	0	0	0	0	0	0	22
0	0	0	0	0	0	0	0	0	0	9
0	0	0	0	0	0	0	0	2	0	4
0	0	0	0	0	0	0	0	3	0	2
0	0	0	0	0	0	0	0	0	0	4
0	0	0	0	0	0	0	0	0	0	0
0	2	0	0	0	0	0	0	0	0	4
0	0	0	0	0	0	0	0	1	0	3
0	0	0	0	0	0	0	0	0	0	0
0	1	0	0	0	0	0	0	0	0	5
0	0	0	0	0	0	0	0	0	0	0
0	0	0	0	1	0	0	0	1	0	3
0	0	0	0	0	0	0	0	0	0	2
0	0	0	0	0	2	0	0	0	0	0
0	0	0	0	0	0	0	0	2	0	0
0	0	0	0	0	0	0	0	0	0	22
0	0	0	0	0	0	0	0	0	0	4
0	0	0	0	0	0	0	0	1	0	0
0	0	0	0	0	1	0	0	1	0	1
0	0	0	0	0	0	0	0	1	0	11

0	0	0	0	0	3	0	0	0	0	4
0	0	0	0	0	1	0	0	0	0	0
0	0	0	0	0	0	0	0	0	0	8
0	0	0	0	0	0	0	0	0	0	4
0	0	0	0	1	0	0	0	0	0	5
0	0	0	0	0	0	0	0	0	0	4
0	0	0	0	0	0	0	0	0	0	12
0	0	0	0	0	0	0	0	0	0	6
0	0	0	0	0	0	0	0	0	0	12
0	0	0	0	0	0	0	0	0	0	8
0	0	0	0	0	0	0	0	0	0	8
0	0	0	0	0	0	0	0	0	0	9
0	0	0	0	0	0	0	0	0	0	7
0	0	0	0	0	0	0	0	0	0	2
0	0	0	0	0	0	0	0	0	0	4
0	0	0	0	0	0	0	0	0	0	5
0	0	0	0	0	0	0	0	0	0	28
0	0	0	0	0	2	0	0	0	0	12
0	0	0	0	0	0	0	0	0	0	4
0	0	0	0	0	0	0	0	0	0	4
0	0	0	0	0	0	0	0	0	0	1
0	0	0	0	0	1	0	0	1	0	5
0	0	0	0	0	1	0	0	0	0	4
0	1	0	0	0	1	0	0	0	0	1
0	0	0	0	0	0	0	0	0	0	4
0	0	0	0	0	0	0	0	0	0	5
0	0	0	0	0	0	0	0	0	0	3
0	0	0	0	0	0	0	0	0	0	0
0	0	0	0	0	0	0	0	0	0	0

0	0	0	0	0	0	0	0	0	0	4
0	0	0	0	0	0	0	0	0	0	3
0	0	0	0	0	0	0	0	0	0	0
0	0	0	0	0	0	0	0	0	0	0
0	0	0	0	0	0	0	0	0	0	0
0	0	0	0	0	0	0	0	0	0	1
0	0	0	0	0	0	0	0	0	0	1
0	0	0	0	0	0	0	0	0	0	0
0	0	0	0	0	0	0	0	0	0	6
0	0	0	0	0	0	0	0	0	0	0
0	0	0	0	0	0	0	0	0	0	1
0	0	0	0	0	0	0	0	0	0	0
0	0	0	0	0	0	0	0	0	0	1
0	0	0	0	0	0	0	0	0	0	1
0	0	0	0	0	0	0	0	0	0	1
0	0	0	0	0	0	0	0	0	0	1
0	0	0	0	0	0	0	0	0	0	1
0	0	0	0	0	0	0	0	0	0	9
0	1	0	0	0	0	0	0	0	0	9
0	0	0	0	0	0	0	0	0	0	10
0	0	0	0	0	0	0	0	0	0	4
0	0	0	0	0	0	0	0	0	0	2
0	0	0	0	0	0	0	0	0	0	0
0	0	0	0	0	0	0	0	0	0	1
0	0	0	0	0	0	0	0	0	0	16
0	0	0	0	0	0	0	0	0	0	11
0	0	0	0	0	0	0	0	0	0	1
0	0	0	0	0	0	0	0	0	0	0
0	1	0	0	0	0	0	0	0	0	0

0	0	0	0	0	0	0	0	0	0	0
0	0	0	0	0	0	0	0	0	0	0
0	0	0	0	0	0	0	0	0	0	0
0	1	0	0	0	0	0	0	0	0	0
0	0	0	0	0	0	0	0	0	0	0
0	0	0	0	0	0	0	0	0	0	0
0	0	0	0	0	0	0	0	0	0	0
0	0	0	0	0	0	0	0	0	0	0
0	0	0	0	0	0	0	0	0	0	0
0	0	0	0	0	0	0	0	0	0	0
0	0	0	0	0	0	0	0	0	0	0
0	0	0	0	0	0	0	0	0	0	0
0	0	0	0	0	0	0	0	0	0	0
0	0	0	0	0	0	0	0	0	0	0
0	0	0	0	0	0	0	0	0	0	0
0	0	0	0	0	0	0	0	0	0	0
0	0	0	0	0	0	0	0	0	0	0
0	0	0	0	0	0	0	0	0	0	0
0	0	0	0	0	0	0	0	0	0	0
0	0	0	0	0	0	0	0	0	0	0
0	0	0	0	0	1	0	0	0	0	0
0	0	0	0	0	0	0	0	0	0	0
0	0	0	0	0	0	0	0	0	0	0
0	0	0	0	0	0	0	0	0	0	0
0	0	0	0	0	0	0	0	0	0	0
0	0	0	0	0	0	0	0	0	0	0
0	0	0	0	0	0	0	0	0	0	0
0	0	0	0	0	0	0	0	0	0	0
0	0	0	0	0	0	0	0	0	0	0

0	0	0	0	0	0	0	0	0	0	0
0	0	0	0	0	0	0	0	0	0	0
0	0	0	0	0	0	0	0	0	0	0
0	0	0	0	0	0	0	0	0	0	0
0	0	0	0	0	0	0	0	0	0	0
0	0	0	0	0	0	0	0	0	0	0
0	2	0	0	0	0	0	0	0	0	0
0	1	0	0	0	0	0	0	0	0	0
0	0	0	0	0	0	0	0	0	0	0
0	0	0	0	0	0	0	0	0	0	0
0	0	0	0	0	0	0	0	0	0	1
0	0	0	0	0	0	0	0	0	0	0
0	1	0	0	0	0	0	0	0	0	0
0	0	0	0	0	0	0	0	0	0	0
0	0	0	0	0	0	0	0	0	0	0
0	0	0	0	0	0	0	0	0	0	0
0	0	0	0	0	0	0	0	0	0	0
0	0	0	0	0	0	0	0	0	0	1
0	0	0	0	0	0	0	0	0	0	0
0	0	0	0	0	0	0	0	0	0	0
0	0	1	0	0	0	0	0	0	0	0
0	0	0	0	0	0	0	0	0	0	1
0	0	0	0	0	0	0	0	0	0	0
0	0	0	0	0	0	0	0	0	0	0
0	0	0	0	1	0	0	0	0	0	5
0	0	0	0	0	0	1	0	0	0	0
0	0	0	0	0	0	0	0	0	0	3
0	0	0	0	0	0	0	0	0	0	0
0	0	2	0	0	0	0	0	0	0	0

0	0	3	0	1	0	0	0	1	0	1
0	0	0	0	1	0	0	0	0	0	2
0	0	0	0	0	0	0	0	1	0	0
0	0	0	0	3	0	0	0	0	0	1
0	0	0	0	1	0	0	0	0	0	0
0	0	0	0	0	0	0	0	0	0	0
0	0	0	0	0	0	0	0	1	0	0
0	0	0	0	0	0	0	0	0	0	2
0	0	0	0	3	0	0	0	1	1	0
0	0	0	0	0	0	0	0	0	0	0
0	0	0	0	1	0	0	0	0	0	0
0	0	0	0	5	0	0	0	0	0	0
0	0	0	0	0	0	0	0	0	0	0
1	0	0	0	0	0	0	0	0	0	0
1	0	0	0	0	0	0	0	0	0	0
0	0	0	0	7	0	0	0	0	0	0
0	0	0	0	7	0	0	0	0	0	0
0	1	0	0	4	0	0	0	0	0	1
0	0	0	0	4	0	0	0	0	0	0
0	0	0	0	10	0	0	0	0	0	0
0	0	0	0	17	0	0	0	0	0	0
0	0	0	0	8	0	0	0	0	0	0
0	0	0	0	6	0	0	0	0	0	0
0	0	0	0	2	0	0	0	0	0	0
0	0	0	0	2	0	0	0	0	0	0
0	0	0	0	4	0	0	0	1	0	0
0	0	0	0	1	0	0	0	1	0	0
0	0	2	0	1	0	0	0	1	0	0
0	0	0	0	3	0	0	0	0	0	1

0	0	0	0	3	0	0	0	0	0	0
0	0	0	0	4	0	0	0	0	0	0
0	0	0	0	0	0	0	0	0	0	0
0	0	0	0	1	0	0	0	0	0	0
0	0	0	0	12	0	0	0	0	0	1
0	0	0	0	2	0	0	0	0	0	0
0	0	0	0	3	0	0	0	0	0	0
0	0	0	0	2	0	0	0	0	0	0
0	0	0	0	2	0	0	0	0	0	0
0	0	0	0	3	0	0	0	0	0	0
0	0	0	0	3	0	0	0	0	0	0
0	0	0	0	1	0	0	0	0	0	0
0	0	0	0	1	0	0	0	0	0	1
0	0	0	0	0	0	0	1	0	0	0
0	0	0	0	2	0	0	0	0	0	0
0	0	0	0	4	0	0	0	0	0	0
0	0	0	0	3	0	0	0	0	0	0
0	0	0	0	2	0	0	0	0	0	0
0	0	0	0	1	0	0	0	0	0	0
0	0	0	0	1	0	0	0	0	0	0
0	0	0	0	0	0	0	0	0	0	0
1	0	0	0	1	0	0	0	0	0	0
0	0	0	0	1	0	0	0	0	0	0
1	0	0	0	2	0	0	0	0	0	0
0	0	1	0	1	0	0	0	0	0	0
0	0	0	0	3	0	0	0	0	0	0
0	0	1	0	1	0	0	0	0	0	0
0	0	0	0	0	0	0	0	0	0	0
0	0	0	0	3	0	0	0	0	0	0

0	0	0	0	1	0	0	0	0	0	0
0	0	0	0	0	0	0	0	0	0	0
0	0	0	0	1	0	0	0	0	0	0
0	0	0	0	2	0	0	0	0	0	0
0	0	0	0	0	0	0	0	0	0	0
0	0	0	0	0	0	0	0	1	0	0
0	0	0	0	1	0	0	0	0	0	0
0	0	0	0	0	0	0	0	0	0	0
0	0	0	0	0	0	0	0	0	0	0
0	0	0	0	1	0	0	0	0	0	0
0	0	0	0	0	0	0	0	0	0	0
0	0	0	0	4	0	0	0	0	0	0
0	0	0	0	10	0	0	0	0	0	0
0	0	0	0	0	0	0	0	1	0	0
0	0	0	0	1	0	0	0	0	0	0
0	0	0	0	0	0	0	0	0	0	0
0	0	0	0	0	1	0	0	0	0	0
0	0	0	0	0	8	0	0	3	0	9
0	0	0	0	0	2	0	0	1	0	0
0	0	0	0	2	0	0	0	0	0	1
0	0	0	0	0	0	0	0	0	0	0
0	0	0	0	2	0	0	0	0	0	0
0	0	0	0	0	0	0	0	0	0	0
0	0	0	0	0	0	0	0	0	0	0
0	0	0	0	1	0	0	0	0	0	0
0	0	0	0	0	0	0	0	0	0	0
0	0	0	0	0	0	0	0	1	0	0
0	0	0	0	0	0	0	0	0	0	0
0	0	0	0	0	0	0	0	0	0	0

0	0	0	0	0	0	0	0	0	0	0
0	0	0	0	0	0	0	0	0	0	0
0	0	1	0	0	0	0	0	1	0	0
0	0	0	0	0	0	0	0	1	0	0
0	0	0	0	0	0	0	0	0	0	1
0	0	0	0	0	0	0	0	0	0	0
0	0	0	0	0	0	0	0	0	0	0
0	0	0	0	0	0	0	0	1	0	0
0	0	0	0	0	0	0	0	1	0	0
0	0	0	0	0	0	0	0	0	0	0
0	0	1	0	0	0	0	0	1	0	0
0	0	0	0	0	0	0	0	0	0	0
0	0	0	0	0	0	0	0	0	0	0
0	0	0	0	0	0	0	0	0	0	0
0	0	0	0	0	0	0	0	0	0	0
0	0	0	0	0	0	0	0	0	0	0
0	0	0	0	0	0	0	0	0	0	0
0	1	0	0	0	0	0	0	0	0	1
0	0	0	0	0	0	0	0	0	0	0
0	0	0	0	0	0	0	0	0	0	0
0	0	0	0	0	0	0	0	0	0	0
0	0	0	0	0	0	0	0	0	0	0
0	0	0	0	0	0	0	0	0	0	0
0	0	0	0	0	0	0	0	0	0	0
0	0	0	1	0	0	0	0	0	0	0
0	0	0	0	0	0	0	0	0	0	0
0	0	0	0	0	0	0	0	0	0	0
0	0	0	0	0	0	0	0	0	0	2
0	1	0	0	0	0	0	0	0	0	0

0	0	0	0	0	0	0	0	1	0	0
0	1	0	0	0	0	0	0	0	0	0
0	0	0	0	0	0	0	0	0	0	0
0	0	0	0	0	0	0	0	0	0	0
0	0	0	0	0	0	0	0	0	0	0
0	1	0	0	0	0	0	0	0	0	0
0	0	0	0	0	0	0	0	0	0	0
0	0	0	0	0	0	0	0	0	0	0
0	1	0	0	0	0	0	0	0	0	0
0	0	0	0	0	0	0	0	0	0	0
0	0	0	0	0	0	0	0	0	0	0
0	0	0	0	0	0	0	0	0	0	0
0	0	0	0	0	0	0	0	0	0	0
0	0	0	0	0	0	0	0	0	0	0
0	0	0	0	0	0	0	0	0	0	0
0	0	0	0	0	1	0	0	0	0	0
0	0	0	0	0	0	0	0	0	0	0
0	0	0	0	0	0	0	0	0	0	0
0	0	0	0	0	0	0	0	0	0	0
0	0	0	0	0	0	0	0	0	0	0
0	0	0	0	0	0	0	0	0	0	0
0	0	0	0	0	0	0	0	0	0	1
0	0	0	0	0	0	0	0	0	0	1

polydo	priauc	protoh	psefat	psethi	sabell	scoben	scocyl	scolel	signov	solpar
0	0	0	0	0	0	0	0	0	0	0
0	2	0	0	0	0	0	0	5	0	0
0	0	0	0	0	0	0	0	0	0	0
0	0	0	0	0	0	1	0	0	0	0

0	0	0	0	0	0	0	0	0	0	0
0	0	0	0	0	0	0	0	0	0	0
0	1	0	0	0	0	0	0	1	0	0
0	0	0	0	0	0	0	0	0	0	0
0	0	0	0	0	0	0	0	2	0	0
0	0	0	0	0	0	0	0	3	0	0
0	2	0	0	0	0	1	0	0	0	0
0	4	0	0	0	0	0	0	0	0	0
0	8	0	2	0	0	0	0	0	0	0
0	14	0	1	0	0	0	0	0	0	0
0	5	0	0	0	0	0	0	0	0	0
0	7	0	0	0	0	0	0	0	0	0
0	10	0	0	0	0	0	0	0	0	0
0	5	0	1	0	0	0	0	0	0	0
0	6	0	0	0	0	0	0	0	0	0
0	7	0	2	0	0	0	0	0	0	0
0	0	0	0	0	0	0	0	0	0	0
0	0	0	0	0	0	0	0	0	0	0
0	0	0	0	0	0	1	0	0	0	0
0	0	0	0	0	0	0	0	0	0	0
0	0	0	0	0	0	0	0	0	0	0
0	0	0	0	0	0	0	0	0	0	0
0	0	0	0	0	0	0	0	1	0	0
0	1	0	0	0	0	0	0	0	0	0
0	0	0	0	0	0	0	0	0	0	0
0	0	0	0	0	0	1	0	0	0	0
0	0	0	0	0	0	0	0	0	0	0
0	13	0	0	0	0	0	0	0	0	0
0	2	0	0	0	0	0	0	0	0	0

0	0	0	0	0	0	0	0	0	0	0
0	0	0	0	0	0	0	0	0	0	0
0	1	0	0	0	0	0	0	0	0	0
0	0	0	0	0	0	0	0	0	0	0
0	2	0	0	0	0	0	0	0	0	0
0	2	0	0	0	0	0	0	0	0	0
0	11	0	0	0	0	0	0	0	0	0
0	3	0	0	0	0	0	0	0	0	0
0	5	0	0	0	0	0	0	0	0	0
0	2	0	0	0	0	0	0	0	0	0
0	3	0	0	0	0	0	0	0	0	0
0	3	0	0	0	0	0	0	0	0	0
0	3	0	0	0	0	0	0	0	0	0
0	4	0	0	0	0	0	0	0	0	0
0	5	0	0	0	0	0	0	0	0	0
0	1	0	1	0	0	0	0	0	0	0
0	2	0	0	0	0	0	0	0	0	0
0	5	0	0	0	0	0	0	0	0	0
0	7	0	0	0	0	0	0	0	0	0
0	8	0	0	0	0	0	0	0	0	0
0	15	0	0	0	0	0	0	0	0	0
0	21	0	0	0	0	0	0	0	0	0
1	4	0	2	0	0	0	0	0	0	0
0	15	0	0	0	0	0	0	0	0	0
0	8	0	1	0	0	1	0	0	0	0
0	5	0	0	0	0	0	0	0	0	0
0	0	0	0	0	0	0	0	0	0	0
3	21	0	0	0	0	1	0	0	0	0
0	14	0	0	1	0	1	0	0	0	0

0	1	1	0	0	0	0	0	0	0	0
1	10	0	0	0	0	0	0	0	0	0
1	13	0	0	0	0	0	0	0	0	0
2	26	0	0	1	0	0	0	0	0	0
1	8	0	0	0	0	0	0	0	0	0
1	9	0	0	0	0	0	0	0	0	0
0	21	0	0	0	0	0	0	0	0	0
0	7	0	0	0	0	0	0	0	0	0
0	5	0	0	0	0	1	0	0	0	0
0	10	0	1	0	0	0	0	0	0	0
0	12	0	0	0	0	0	0	0	0	0
0	4	0	0	0	0	0	0	0	0	0
0	10	0	0	0	0	0	0	0	0	0
0	2	0	0	0	0	0	0	0	0	0
0	19	0	0	0	0	0	0	0	0	0
0	3	0	0	0	0	0	0	0	0	0
0	13	0	0	0	0	0	0	0	0	0
0	0	0	0	0	0	0	0	0	0	0
0	5	0	0	0	0	0	0	0	1	0
0	7	0	0	0	0	0	0	0	0	0
0	8	0	0	0	0	1	0	0	0	0
0	4	0	0	0	0	0	0	0	0	0
0	5	0	2	0	0	0	0	0	0	0
0	3	0	1	0	0	0	0	0	0	0
0	1	0	1	0	0	0	0	0	0	0
0	0	0	0	0	0	0	0	0	0	0
0	1	0	0	0	0	0	0	0	0	0
0	2	0	0	0	0	0	0	0	0	0
0	0	0	0	0	0	0	0	0	0	0

0	5	0	0	0	0	0	0	0	0	0
0	4	0	1	0	0	0	0	0	0	0
0	4	0	0	0	0	0	0	0	0	0
0	2	0	0	0	0	0	0	0	0	0
0	4	0	0	0	0	0	0	0	0	0
0	6	0	0	0	0	0	0	0	0	0
0	6	0	0	0	0	0	0	0	0	0
0	3	0	0	0	0	0	0	0	0	0
0	11	0	1	0	0	0	0	0	0	0
0	2	0	0	0	0	0	0	0	0	0
0	1	0	0	0	0	0	0	0	0	0
0	5	0	0	0	0	0	0	0	0	0
0	6	0	0	0	0	0	0	0	0	0
0	2	0	0	0	0	0	0	0	0	0
0	3	0	0	0	0	0	0	0	0	0
0	1	0	0	0	0	0	0	0	0	0
0	3	0	0	0	0	0	0	0	0	0
0	2	0	3	0	0	0	0	0	0	0
0	4	0	0	0	0	0	0	0	0	0
0	6	0	3	0	0	0	0	0	0	0
0	1	0	0	0	0	0	0	0	0	0
0	5	0	0	0	0	0	0	0	0	0
0	1	0	0	0	0	0	0	0	0	0
0	14	0	1	0	0	0	0	0	0	0
0	2	0	0	0	0	0	0	0	0	0
0	8	0	0	0	0	0	0	0	0	0
0	1	0	0	0	0	0	1	0	0	0
0	3	0	0	0	0	0	0	0	0	0
0	3	0	0	0	0	0	0	0	0	0

0	0	0	0	0	0	0	0	0	0	0
0	1	0	0	0	0	0	0	0	0	0
0	1	0	0	0	0	0	0	0	0	0
0	1	0	0	0	0	0	0	0	0	0
0	2	0	1	0	0	0	0	0	0	0
0	5	0	0	0	0	0	0	0	0	0
0	6	0	0	0	0	0	0	0	0	0
0	2	0	0	0	0	0	0	0	0	0
0	2	0	1	0	0	0	0	0	0	0
0	4	0	0	0	0	0	0	0	0	0
0	2	0	0	0	0	0	0	0	0	0
0	1	0	0	0	0	0	0	0	0	0
0	6	0	1	0	0	0	0	0	0	0
0	8	0	1	0	0	0	0	0	0	0
0	1	0	0	0	0	0	0	0	0	0
0	9	0	0	0	0	0	0	0	0	0
0	4	0	0	0	0	0	0	0	0	0
0	7	0	0	0	0	0	0	0	0	0
0	4	0	0	0	0	0	0	0	0	0
0	17	0	0	0	0	0	0	0	0	0
0	2	0	0	0	0	0	0	0	0	0
0	0	0	0	0	0	0	0	0	0	0
0	1	0	0	0	0	0	0	0	0	0
0	2	0	1	0	0	0	0	0	0	0
0	17	0	0	0	0	0	0	0	0	0
0	0	0	0	0	0	0	0	0	0	0
0	0	0	0	0	0	0	0	0	0	0
1	3	0	1	0	0	0	0	0	0	0
0	1	0	2	0	0	0	0	0	0	0

0	2	0	0	0	0	0	0	0	0	0
0	1	0	2	0	0	0	0	0	0	0
0	10	0	0	0	0	0	0	0	0	0
0	2	0	0	0	0	0	0	0	0	0
0	3	0	1	0	0	0	0	0	0	0
0	9	0	0	0	0	0	0	0	0	0
0	1	0	0	0	0	0	0	0	0	0
0	3	0	0	0	0	0	0	0	0	0
0	0	0	0	0	0	0	0	0	0	0
0	5	0	0	0	0	1	0	0	0	0
0	7	0	0	0	0	0	0	0	0	0
1	7	0	0	0	0	0	0	0	0	0
0	3	0	0	0	0	0	0	0	0	0
0	6	0	0	0	0	0	0	0	0	0
0	13	0	0	0	0	1	0	0	0	0
0	5	0	0	0	0	0	0	0	0	0
0	7	0	0	0	0	0	0	0	0	0
0	10	0	0	0	0	0	0	0	0	0
0	11	0	0	0	0	0	0	0	0	0
0	5	0	0	0	0	0	0	0	0	0
0	14	0	0	0	0	0	0	0	0	0
0	5	0	0	0	0	0	0	0	0	0
0	4	0	0	0	0	0	0	0	0	0
0	5	0	0	0	0	0	0	0	0	0
0	11	0	0	0	0	0	0	0	0	0
0	15	0	0	0	0	0	0	0	0	0
0	6	0	0	0	0	0	0	0	0	0
0	7	0	0	0	0	0	0	0	0	0
0	9	0	0	0	0	0	0	0	0	0

0	10	0	0	0	0	0	0	0	0	0
0	17	0	0	0	0	0	0	0	0	0
0	4	0	0	0	0	0	0	0	0	0
0	0	0	0	0	0	0	0	0	0	0
0	3	0	0	0	0	0	0	0	0	0
0	7	0	0	0	0	1	0	0	0	0
0	5	0	0	0	0	0	0	0	0	0
0	0	0	0	0	0	0	0	0	0	0
0	0	0	0	0	0	0	0	0	0	0
0	1	0	0	0	0	1	0	0	0	0
0	0	0	0	0	0	0	0	0	0	1
0	0	0	0	0	0	0	0	0	0	0
0	0	0	0	0	0	0	0	0	0	0
0	0	0	0	0	0	0	0	0	0	0
0	0	0	0	0	0	1	0	0	0	0
0	0	0	0	0	0	0	0	0	0	0
0	1	0	0	0	1	0	0	0	0	0
0	0	0	0	0	0	0	0	0	0	0
0	0	0	0	0	0	0	0	0	0	0
0	2	0	0	0	0	0	0	0	0	0
0	0	0	0	0	0	0	0	0	0	0
0	0	0	0	0	0	0	0	0	0	0
0	0	0	0	0	0	0	0	0	0	0
0	3	0	0	0	0	0	0	0	0	0
0	0	0	0	0	0	0	0	0	0	0
0	0	0	0	0	0	0	0	0	0	0
0	1	0	0	0	0	0	0	0	0	0
0	0	0	0	0	0	0	0	0	0	0
0	0	0	0	0	0	0	0	0	0	0

0	0	0	0	0	0	0	0	0	0	0
0	2	0	0	0	0	1	0	0	0	0
0	1	0	0	0	0	0	0	0	0	0
0	1	0	0	0	0	0	0	0	0	0
0	6	0	0	0	0	0	0	0	0	0
0	0	0	0	0	0	0	0	0	0	0
0	1	0	0	0	0	1	0	0	0	0
0	1	0	0	0	0	0	0	0	0	0
0	0	0	0	0	0	0	0	0	0	0
0	1	0	0	0	0	0	0	0	0	0
0	1	0	0	0	0	1	0	0	0	0
0	6	0	0	0	0	0	0	0	0	0
0	6	0	0	0	0	0	0	0	0	0
0	17	0	0	0	0	0	0	0	0	0
0	6	0	0	0	0	0	0	0	0	0
0	5	0	0	0	0	0	0	0	0	0
0	6	0	0	0	0	0	0	0	0	0
0	1	0	0	0	0	0	0	0	0	0
0	4	0	0	0	0	0	0	0	0	0
0	3	0	0	0	0	0	0	0	0	0
0	5	0	0	0	0	0	0	0	0	0
0	5	0	0	0	0	0	0	0	0	0
0	2	0	0	0	0	0	0	0	0	1
0	5	0	0	0	0	0	0	0	0	0
0	3	0	0	0	0	0	0	0	0	0
0	9	0	0	0	0	0	0	0	0	0
0	6	0	0	0	0	0	0	0	0	0
0	4	0	0	0	0	0	0	0	0	0
0	1	0	0	0	0	0	0	0	0	0

0	6	0	0	0	0	0	0	0	0	0
0	3	0	0	0	0	0	0	0	0	0
0	6	0	0	0	0	0	0	0	0	0
0	1	0	0	0	0	0	0	0	0	0
0	1	0	0	0	0	0	0	0	0	0
0	1	0	0	0	0	0	0	0	0	0
0	2	0	1	0	0	0	0	0	0	0
0	1	0	0	0	0	0	0	0	0	0
0	1	0	1	0	0	0	0	0	0	0
0	0	0	0	0	0	0	0	0	0	0
0	1	0	1	0	0	0	0	0	0	0
0	1	0	0	0	0	0	0	0	0	0
0	2	0	1	0	0	0	0	0	0	0
0	0	0	0	0	0	0	0	0	0	0
0	0	0	0	0	0	0	0	0	0	0
0	1	0	0	0	0	0	0	0	0	0
0	2	0	0	0	0	0	0	0	0	0
0	1	0	0	0	0	0	0	0	0	0
0	0	0	0	0	0	0	0	0	0	0
0	0	0	0	0	0	0	0	0	0	0
0	0	0	0	0	0	0	0	0	1	0
0	1	0	0	0	0	0	0	0	0	0
0	2	0	0	0	0	0	0	0	0	0
0	0	0	0	0	0	0	0	0	0	0
0	1	0	0	0	0	0	0	0	0	0
0	3	0	0	0	0	0	0	0	0	0
0	0	0	6	0	0	0	0	0	0	0
0	1	0	0	0	0	0	0	0	0	0
0	5	0	0	0	0	0	0	0	0	0

0	0	0	1	0	0	0	0	0	0	0
0	0	0	0	0	0	0	0	0	0	0
0	4	0	0	0	0	0	0	0	0	0
0	3	0	1	0	0	0	0	0	0	0
0	2	0	1	0	0	0	0	0	0	0
0	2	0	0	0	0	0	0	0	0	0
0	3	0	1	0	0	0	0	0	0	0
0	4	0	0	0	0	0	0	0	0	0
0	2	0	0	0	0	0	0	0	0	0
0	1	0	0	0	0	0	0	0	0	0
0	2	0	0	0	0	0	0	0	0	0
0	0	0	1	0	0	0	0	0	0	0
0	0	0	2	0	0	0	0	0	0	0
0	2	0	0	0	0	0	0	0	0	0
0	0	0	0	0	0	0	0	0	0	0
0	4	0	3	0	0	0	0	0	0	0
0	0	0	0	0	0	0	0	0	0	0
0	0	0	2	0	0	0	0	0	0	0
0	6	0	0	0	0	0	0	0	0	0
0	3	0	2	0	0	0	0	0	0	0
0	2	0	4	0	0	0	0	0	0	0
0	0	0	0	0	0	0	0	0	0	0
0	3	0	0	0	0	0	0	0	0	0
0	1	0	1	0	0	0	0	0	0	0
0	3	0	0	0	0	0	0	0	0	0
0	2	0	0	0	0	0	0	0	0	0
0	2	0	0	0	0	0	0	0	0	0
0	1	0	0	0	0	0	0	0	0	0
0	0	0	0	0	0	0	0	0	0	0

0	2	0	0	0	0	0	0	0	0	0
0	0	0	0	0	0	0	0	0	0	0
0	3	0	0	0	0	0	0	0	0	0
0	2	0	0	0	0	0	0	0	0	0
0	3	0	0	0	0	0	0	0	0	0
0	0	0	0	0	0	0	0	0	0	0
0	0	0	0	0	0	0	0	0	0	0
0	1	0	0	0	0	0	0	0	0	0
0	1	0	0	0	0	0	0	0	0	0
0	2	0	0	0	0	0	0	0	0	0
0	11	0	0	0	0	0	0	0	0	0
0	12	0	1	0	0	0	0	0	0	0
0	5	0	0	0	0	0	0	0	0	0
0	2	0	0	0	0	0	0	0	0	0
0	4	0	0	0	0	0	0	0	0	0
0	0	0	0	0	0	0	0	0	0	0
0	1	0	1	0	0	0	0	0	0	0
0	2	0	1	0	0	1	0	0	0	0
0	0	0	0	0	0	0	0	0	0	0
0	4	0	0	0	0	0	0	0	0	0
0	1	0	0	0	0	0	0	0	0	0
0	1	0	0	0	0	0	0	0	0	0
0	6	0	2	0	0	0	0	0	0	1
0	1	0	0	0	0	0	0	0	0	0
0	2	0	2	0	0	0	0	0	0	1
0	1	0	0	0	0	0	0	0	0	0
0	2	0	1	0	0	0	0	0	0	0
0	3	0	0	0	0	0	0	0	0	0
0	0	0	0	0	0	0	0	0	0	0

0	2	0	0	0	0	0	0	0	0	0
0	4	0	0	0	0	0	0	0	0	0
0	1	0	2	0	0	0	0	0	0	0
0	3	0	0	0	0	0	0	0	0	0
0	1	0	0	0	0	0	0	0	0	0
0	2	0	0	0	0	0	0	0	0	0
0	1	0	0	0	0	0	0	0	0	0
0	1	0	0	0	0	0	0	0	0	0
0	0	0	0	0	0	0	0	0	0	0
0	5	0	1	0	0	0	0	0	0	0
0	0	0	1	0	0	0	0	0	0	0
0	3	0	0	0	0	0	0	0	0	0
0	3	0	0	0	0	0	0	0	0	0
0	1	0	0	0	0	0	0	0	0	0
0	3	0	1	0	0	0	0	0	0	0
0	1	0	0	0	0	0	0	0	0	0
0	4	0	0	0	0	0	0	0	0	0
0	0	0	1	0	0	0	0	0	0	0
0	1	0	0	0	0	0	0	0	0	0
0	3	0	0	0	0	0	0	0	0	0
0	0	0	5	0	0	0	0	0	0	0
0	0	0	2	0	0	0	0	0	0	0
0	0	0	0	0	0	1	0	0	0	0
0	0	0	0	0	0	0	0	0	0	0
0	0	0	0	0	0	0	0	0	0	0
0	3	0	0	0	0	0	0	0	0	0
0	0	0	1	0	0	0	0	0	0	0
0	3	0	0	0	0	0	0	0	0	0
0	0	0	0	0	0	0	0	0	0	0

0	1	0	3	0	0	0	0	0	0	0
0	0	0	2	0	0	0	0	0	0	0
0	2	0	1	0	0	0	0	0	0	0
0	1	0	0	0	0	0	0	0	0	0
0	5	0	0	0	0	0	0	0	0	0
0	0	0	0	0	0	0	0	0	0	0
0	2	0	0	0	0	0	0	0	0	0
0	1	0	0	0	0	0	0	0	0	0
0	2	0	3	0	0	0	0	0	0	0
0	2	0	0	0	0	0	0	0	0	0
0	0	0	1	0	0	0	0	0	0	0
0	3	0	0	0	0	0	0	0	0	0
0	1	0	3	0	0	0	0	0	0	0
0	0	0	1	0	0	0	0	0	0	0
0	2	0	0	0	0	0	0	0	0	0
0	1	0	0	0	0	0	0	0	0	0
0	5	0	0	0	0	0	0	0	0	0
0	5	0	0	0	0	0	0	0	0	0
0	8	0	0	0	0	0	0	0	0	0
0	2	0	0	0	0	1	0	0	0	0
0	3	0	0	0	0	0	0	0	0	0
0	3	0	0	0	0	0	0	0	0	0
0	0	0	0	0	0	0	0	0	0	0
0	3	0	1	0	0	0	1	0	0	0
0	2	0	0	0	0	1	0	0	0	0
0	0	0	1	0	0	0	0	0	0	0
0	2	0	0	0	0	0	0	0	0	0
0	0	0	0	0	0	0	0	0	0	0
0	1	0	0	0	0	0	0	0	0	0

0	4	0	0	0	0	0	0	0	0	0
0	0	0	0	0	0	1	0	0	0	0
0	1	0	0	0	0	0	0	0	0	0
0	2	0	0	0	0	0	0	0	0	0
0	2	0	0	0	0	0	0	0	0	0
0	1	0	0	0	0	0	0	0	0	0
0	11	0	0	0	0	0	0	0	0	0
0	4	0	1	0	0	0	0	0	0	0
0	6	0	0	0	0	0	0	0	0	0
0	3	0	0	0	0	0	0	0	0	0
0	0	0	0	0	0	0	0	0	0	0
0	8	0	0	0	0	1	0	0	0	0
0	4	0	0	0	0	0	0	0	0	0
0	0	0	0	0	0	0	0	0	0	0
0	1	0	0	0	0	0	0	0	0	0
0	1	0	0	0	0	0	0	0	0	0
0	0	0	0	0	0	1	0	0	0	0
0	4	0	0	0	0	0	0	0	0	0
0	0	0	0	0	0	0	0	0	0	0

protoh	psefat	psethi	scalib	scoben	scocyl	scolel	solpar	solsil	squarm	thelub
0	0	0	0	0	0	0	0	7	0	0
0	0	0	0	0	0	0	0	1	0	0
0	0	0	0	0	0	0	0	4	0	0
0	0	0	0	0	0	0	0	5	0	0
0	0	0	0	1	0	0	0	0	0	0
0	0	0	0	0	0	0	0	0	0	0
0	0	0	0	0	0	0	0	7	0	0
0	0	0	0	0	0	0	0	0	0	0
0	0	0	0	0	0	0	0	0	0	0
0	0	0	0	0	0	0	0	1	0	0
0	1	0	0	0	0	0	0	1	0	0
0	1	0	0	0	0	0	0	0	0	0
0	0	0	0	0	0	0	0	1	0	0
0	0	0	0	0	0	0	0	0	0	0
0	0	0	0	0	0	0	0	1	0	0
0	0	0	0	0	0	0	0	0	0	0
0	0	0	0	0	0	0	0	8	0	0
0	0	0	0	0	0	0	0	0	0	0
0	0	0	0	0	0	0	0	0	0	0
0	0	0	0	0	0	0	0	0	0	0
0	0	0	0	0	0	0	0	0	0	0
0	0	0	0	0	0	0	0	1	0	0
0	0	0	0	0	0	0	0	10	0	0
0	0	0	0	0	0	0	0	8	0	0
0	0	0	1	0	0	0	0	0	0	1
0	0	0	0	0	0	0	0	0	0	0
0	0	0	0	0	0	0	0	0	0	0
0	0	0	0	0	0	0	0	1	0	0

0	0	0	0	0	0	0	0	0	0	0
0	0	0	0	0	0	0	0	0	0	0
0	1	0	0	0	0	0	0	0	0	0
0	0	0	0	0	0	0	0	0	0	0
0	0	0	0	0	0	0	0	0	0	0
0	0	0	0	0	0	0	0	0	0	0
0	0	0	0	0	0	0	0	2	0	0
0	0	0	0	0	0	0	0	0	0	0
0	0	0	0	0	0	0	0	0	0	0
0	1	0	0	0	0	0	0	0	0	0
0	1	0	0	1	0	0	0	0	0	0
0	3	0	0	0	0	0	0	0	0	0
0	5	0	0	0	0	0	0	0	0	0
0	1	0	0	0	0	0	0	2	0	0
0	0	0	1	0	0	0	0	7	0	0
0	0	0	0	1	0	0	0	22	0	0
0	1	1	3	0	0	0	0	0	0	0
0	0	0	0	0	0	0	0	3	0	0
0	0	0	0	0	0	0	0	0	0	0
0	0	0	0	0	0	0	0	0	0	0
0	0	0	0	0	0	0	0	2	0	0
0	0	0	0	0	0	0	0	0	0	0
0	0	0	0	1	0	0	0	4	0	0
0	0	0	0	0	0	0	0	0	0	0
0	0	0	0	0	0	0	0	0	0	0
0	0	0	0	0	1	0	1	0	0	0
0	1	0	0	0	0	0	0	1	0	0
0	0	0	0	0	0	0	0	1	0	0
0	2	2	0	0	0	0	0	0	0	0

0	0	0	0	0	0	0	0	60	0	0
0	0	0	0	0	0	0	0	0	0	0
0	0	0	0	0	0	0	0	0	0	0
0	0	0	0	0	0	0	0	0	0	0
0	0	0	0	0	0	0	0	0	0	0
0	0	0	0	0	0	0	0	0	0	0
0	1	76	0	0	0	0	0	0	0	0
0	0	15	0	0	0	0	0	0	0	0
0	1	0	0	0	0	0	0	0	0	0
0	0	0	0	0	0	0	0	7	0	0
0	0	0	0	0	0	0	0	0	0	0
0	0	0	0	0	0	0	0	1	0	0
0	0	0	0	0	0	0	0	0	0	0
0	0	0	0	0	0	0	0	0	0	0
0	0	0	0	0	0	0	0	1	0	0
0	0	0	0	0	0	0	0	3	0	0
0	0	0	0	0	0	0	0	0	0	0
0	0	0	0	0	0	0	0	19	0	0
0	0	0	0	0	0	0	0	15	0	0
0	0	0	0	0	0	0	0	2	0	0
0	0	0	0	0	0	0	0	23	0	0
0	0	0	0	1	0	0	0	11	0	0
0	0	0	1	1	0	0	0	0	0	0
0	0	0	3	1	0	0	0	0	0	0
0	0	0	0	0	0	0	0	0	0	0
0	0	0	0	0	0	0	0	0	0	0
0	0	0	0	0	0	0	0	0	0	0
0	0	0	0	0	0	0	0	0	0	0
0	3	0	0	0	0	0	0	0	0	0

0	2	0	0	0	0	0	0	0	0	0
0	3	0	1	0	0	0	0	0	0	0
0	1	62	2	0	0	0	0	1	0	0
0	0	12	0	0	0	0	0	1	0	0
0	0	0	0	0	0	0	0	0	0	0
0	0	0	0	1	0	0	0	0	0	0
0	0	0	1	0	0	0	0	0	0	0
0	0	0	1	0	0	0	0	0	0	0
0	0	0	0	0	0	0	0	2	0	0
0	0	0	0	0	0	0	0	2	0	0
0	1	0	1	0	0	0	0	2	0	0
0	0	0	0	0	0	0	0	2	0	1
0	0	0	0	0	0	0	0	0	0	0
0	0	0	0	1	0	1	0	1	0	0
0	0	0	0	0	0	0	0	0	0	0
0	0	0	0	4	0	1	0	0	0	0
0	0	0	0	0	0	0	0	0	0	0
0	3	0	0	1	0	0	0	0	0	0
0	1	0	0	1	0	0	0	0	0	0
0	1	0	0	0	0	0	0	0	0	0
0	0	0	0	0	0	0	0	0	0	0
0	0	0	0	0	0	0	0	0	0	0
0	0	1	0	0	0	0	0	0	0	0
0	0	0	0	0	0	0	0	0	0	0
0	4	0	0	0	0	0	0	0	0	0
0	0	0	0	1	0	0	0	0	0	0
0	0	0	0	0	0	0	0	2	0	0
0	0	1	0	0	0	0	0	3	0	0
0	10	1	0	0	0	0	0	2	0	0

0	1	1	0	0	0	0	0	2	0	0
0	0	0	0	1	0	0	0	0	0	0
0	0	0	0	0	0	0	0	14	0	0
0	0	0	0	0	0	0	0	0	0	0
0	0	0	0	0	0	0	0	4	0	0
0	0	0	0	1	0	0	0	0	0	0
0	0	0	0	0	0	0	0	3	0	0
0	0	0	0	0	0	0	0	9	0	0
0	0	0	0	0	0	0	0	12	0	0
0	0	0	0	2	0	0	0	9	0	0
0	0	0	0	0	0	0	0	5	0	0
0	1	0	0	0	0	0	0	0	0	0
0	1	0	0	0	0	0	0	0	0	0
0	0	9	1	0	0	0	0	0	0	0
0	0	0	0	0	0	0	0	0	0	0
0	0	0	0	0	0	0	0	0	0	0
0	3	0	0	0	0	0	0	0	0	0
0	0	0	0	0	0	0	0	1	0	0
0	0	0	0	0	0	0	0	0	0	0
0	1	0	0	0	0	0	0	0	0	0
0	7	0	0	0	0	0	0	0	0	0
0	0	0	0	0	0	0	0	0	0	0
0	0	0	0	0	0	0	0	1	0	0
0	0	0	0	0	0	0	0	0	0	0
0	1	0	0	0	0	0	0	0	0	0
0	3	0	0	0	0	0	0	0	0	0
0	0	0	0	0	0	0	0	0	0	0
0	0	0	0	0	0	0	0	0	0	0
0	0	0	0	0	0	0	0	0	0	0

0	1	0	0	0	0	0	0	0	0	0
0	0	0	0	0	0	0	0	0	0	0
0	0	0	0	1	0	0	0	0	0	0
0	4	0	0	0	0	0	0	0	0	0
0	0	0	0	0	0	0	0	2	0	0
0	0	0	0	0	0	0	0	4	0	0
0	0	0	0	2	0	0	0	1	0	0
0	0	0	0	0	0	0	0	2	0	0
0	1	0	0	0	0	0	0	1	0	0
0	0	0	0	0	0	0	0	7	0	0
0	0	0	0	0	0	0	0	2	0	0
0	1	0	0	1	0	0	0	5	0	0
0	0	0	0	1	0	0	0	3	0	0
0	0	0	0	0	0	0	0	2	0	0
0	0	0	0	0	0	0	0	0	0	0
0	0	0	1	0	0	0	0	0	0	0
0	0	0	0	0	0	0	0	0	0	0
0	1	0	0	0	0	0	0	2	0	0
0	0	0	1	1	0	0	0	0	0	0
0	0	0	0	0	0	0	0	0	0	0
0	0	0	0	0	0	0	0	0	0	0
0	1	0	0	0	0	0	0	0	0	0
0	0	0	0	0	0	0	0	0	0	0
0	0	0	0	0	0	0	0	0	0	0
0	0	0	0	0	0	0	0	3	1	0
0	0	0	0	0	0	0	0	2	0	0
0	0	0	0	0	0	0	0	9	0	0
0	0	0	0	1	0	0	0	16	0	0
0	0	0	0	0	0	0	0	8	0	0

0	0	0	0	0	0	0	0	21	0	0
0	0	0	0	0	0	0	0	5	0	0
0	0	0	0	0	0	0	0	5	0	0
0	0	0	0	0	0	0	0	10	0	0
0	0	0	0	0	0	0	0	7	0	0
0	0	0	0	0	0	0	0	22	0	0
0	0	0	0	0	0	0	0	12	0	0
0	0	0	0	0	0	0	0	4	0	0
0	0	0	0	2	0	0	0	6	0	0
0	0	0	0	0	0	0	0	8	0	0
0	0	0	0	0	0	0	0	16	0	0
0	0	0	0	0	0	0	0	4	0	0
0	0	0	0	0	0	0	0	8	0	0
0	0	0	0	0	0	0	0	9	0	0
0	0	0	0	0	0	0	0	14	0	0
0	0	0	0	0	0	0	0	14	0	0
0	0	0	0	2	0	0	0	7	0	0
0	0	0	0	0	0	0	0	22	0	0
0	0	0	0	1	0	0	0	0	0	0
0	0	0	0	0	0	0	0	4	0	0
0	0	0	0	0	0	1	0	9	0	0
0	0	0	0	0	0	0	0	2	0	0
0	0	0	0	2	0	1	0	3	0	0
0	0	0	0	0	0	0	0	2	0	0
0	0	0	0	1	0	2	0	1	0	0
0	0	0	0	0	0	0	0	0	0	0
0	0	0	0	1	0	0	0	1	0	0
0	0	0	0	3	1	0	0	0	0	0
0	0	0	0	0	0	0	0	3	0	0

0	0	0	0	1	0	0	0	0	0	0
0	0	0	0	2	0	3	0	0	0	0
0	0	0	0	1	0	0	0	1	0	0
0	0	0	0	2	0	3	0	0	0	0
0	0	0	0	0	0	0	0	0	0	0
0	0	0	0	2	0	1	0	0	0	0
0	0	0	0	0	0	0	0	0	0	0
0	0	0	0	0	0	0	0	1	0	0
0	0	0	0	1	0	0	0	9	0	0
0	0	0	0	1	0	0	0	7	0	0
0	0	0	0	0	0	0	0	6	0	0
0	0	0	0	0	0	0	0	5	0	0
0	0	0	0	0	0	0	0	5	0	0
0	0	0	0	2	0	0	0	11	0	0
0	0	0	0	0	0	0	0	5	0	0
0	0	0	0	0	0	0	0	15	0	0
0	0	0	0	0	0	0	0	9	0	0
0	0	0	0	2	0	0	0	8	0	0
0	0	0	0	0	0	0	0	13	0	0
0	0	0	0	0	0	0	0	0	0	0
0	0	0	0	1	0	0	0	15	0	0
0	0	0	0	1	0	0	0	2	0	0
0	0	0	0	0	0	0	0	4	0	0
0	0	0	0	0	0	0	0	0	0	0
0	0	0	0	0	0	0	0	4	0	0
0	0	0	0	0	0	0	0	0	0	0
0	0	0	0	1	0	0	0	0	0	0
0	0	0	0	1	0	1	0	0	0	0
0	0	0	0	0	0	0	0	1	0	0

0	0	0	0	0	0	0	0	1	0	0
0	0	0	0	0	0	0	0	0	0	0
0	0	0	0	0	0	0	0	1	0	0
0	0	0	0	0	0	0	0	13	0	0
0	0	0	0	0	0	0	0	2	0	0
0	0	0	0	0	0	0	0	1	0	0
0	0	0	0	0	0	0	0	1	0	0
0	0	0	0	0	0	0	0	0	0	0
0	0	0	0	1	0	0	0	2	0	0
0	0	0	0	0	0	0	0	2	0	0
0	0	1	0	0	0	1	0	0	0	0
0	0	0	0	0	0	0	0	1	0	0
0	0	0	0	1	0	0	0	1	0	0
0	0	0	0	0	0	0	0	1	0	0
0	0	0	0	1	0	0	0	0	0	0
0	1	0	0	1	0	0	0	1	1	0
0	0	0	0	0	0	0	0	1	0	0
0	6	1	0	0	0	0	0	1	0	0
0	7	0	0	0	0	0	0	1	0	0
0	0	0	0	1	0	0	0	1	0	0
0	2	0	0	0	0	0	0	0	0	0
0	1	0	0	0	0	0	0	0	0	0
0	9	0	0	0	0	0	0	5	0	0
0	1	0	0	0	0	0	0	1	0	0
0	0	1	0	1	0	0	0	0	0	0
0	2	0	0	0	0	0	0	1	0	0
0	2	0	0	0	0	0	0	1	0	0
0	1	0	0	0	0	0	0	1	0	0
0	15	5	0	0	0	0	0	1	0	0

0	2	0	0	0	0	0	0	2	0	0
0	0	0	0	0	0	0	0	0	0	0
0	2	0	0	0	0	0	0	0	0	0
0	1	0	0	0	0	0	0	2	0	0
0	0	0	0	0	0	0	0	0	0	0
0	0	0	0	0	0	0	0	0	0	0
0	1	0	0	0	0	0	0	0	0	0
0	0	1	0	0	0	0	0	0	0	0
0	0	0	0	0	0	0	0	0	0	0
0	0	0	0	0	0	0	0	0	0	0
0	6	0	0	0	0	0	0	0	0	0
0	0	0	0	0	0	0	0	0	0	0
0	2	0	0	0	0	0	0	0	0	0
0	0	1	0	0	0	0	0	0	0	0
0	0	0	0	0	0	0	0	0	0	0
0	2	50	0	0	0	0	0	0	0	0
0	3	144	0	0	0	0	0	0	0	0
0	0	0	0	0	0	0	0	1	0	0
0	0	0	0	0	0	0	0	0	0	0
0	0	0	0	0	0	0	0	6	0	0
0	0	0	0	0	0	0	0	0	0	0
0	0	0	0	0	0	0	0	0	0	0
0	0	0	0	0	0	0	0	0	0	0
0	0	0	0	0	0	0	0	0	0	0
0	0	0	0	0	0	0	0	1	0	0
0	0	0	0	0	0	0	0	1	0	0
0	0	0	0	0	0	0	0	0	0	0
0	0	0	0	0	0	0	0	0	0	0
0	0	0	0	0	0	0	0	0	0	0

0	0	0	0	0	0	0	0	0	0	0
0	0	0	0	0	0	0	0	1	0	0
0	0	0	0	0	0	0	0	0	0	0
1	0	0	0	0	0	0	0	5	0	0
0	0	0	0	1	0	0	0	1	0	0
0	0	0	0	0	0	0	0	0	0	0
0	0	0	0	0	0	0	0	0	0	0
0	0	0	0	0	0	0	0	0	0	0
0	0	0	0	0	0	0	0	0	0	0
0	0	3	0	0	0	0	0	1	0	0
0	0	0	0	0	0	0	0	0	0	0
0	0	0	0	0	0	0	0	0	0	0
0	0	0	0	0	0	0	0	2	0	0
0	0	0	0	0	0	0	0	0	0	0
0	0	0	0	0	0	0	0	1	0	0
0	0	0	0	0	0	0	0	0	0	0
0	0	1	0	0	0	0	0	0	0	0
0	0	0	0	0	0	0	0	0	0	0
0	0	0	0	0	0	0	0	1	0	0
0	0	0	0	0	0	0	1	1	0	0
0	0	0	0	0	0	0	0	1	0	0
0	0	3	0	0	0	0	0	0	0	0
0	0	0	0	0	0	0	0	0	0	0
0	0	13	0	0	0	0	0	0	0	0
0	1	0	0	0	0	0	0	0	0	0
0	0	0	0	0	0	0	0	0	0	0
0	0	0	0	0	0	0	0	0	0	0
0	0	0	0	0	0	0	0	0	0	0
0	0	0	0	0	0	0	0	0	0	0

0	0	0	0	0	0	0	0	0	0	0
0	0	0	0	0	0	0	0	0	0	0
0	0	0	0	0	0	0	0	0	0	0
0	1	0	0	0	0	0	0	0	0	0
0	0	0	0	0	0	0	0	0	0	2
0	0	0	0	0	0	0	0	0	0	0
0	0	0	0	0	0	0	0	0	0	0
0	0	0	0	0	0	0	0	0	0	0
0	0	0	0	0	0	0	0	0	0	0
0	0	0	0	0	0	0	0	0	0	0
0	0	0	0	0	0	0	0	0	0	0
0	0	0	0	0	0	0	0	0	0	0
0	0	0	0	0	0	0	0	1	0	0
0	0	0	0	0	0	0	0	0	0	0
0	0	0	0	0	0	0	0	0	0	0
0	0	0	0	0	0	0	0	0	0	0
0	0	0	0	0	0	0	0	1	0	0
0	0	2	0	0	0	0	0	0	0	0
0	0	1	0	0	0	0	0	0	0	0
0	4	4	0	0	0	0	0	0	0	0
0	0	1	0	0	0	0	0	4	0	0
0	2	1	0	0	0	0	0	0	0	0
0	0	0	0	0	0	0	0	1	0	0
0	1	0	0	0	0	0	0	0	0	0
0	4	1	0	0	0	0	0	0	0	0
0	1	0	0	0	0	0	0	0	0	0
0	0	1	0	0	0	0	0	0	0	0
0	1	0	0	0	0	0	0	1	0	0
0	1	0	0	0	0	0	0	0	0	0

0	0	0	0	0	0	0	0	3	0	0
0	1	0	0	0	0	0	0	1	0	0
0	1	0	0	0	0	0	0	6	0	0
0	0	0	0	1	0	0	0	4	0	0
0	0	0	0	0	0	0	0	0	0	0
0	1	0	0	0	0	0	0	2	0	0
0	1	0	0	0	0	0	0	4	0	0
0	0	0	0	0	0	0	0	0	0	0
0	2	0	0	0	0	0	0	2	0	0
0	1	0	0	0	0	0	0	2	0	0
0	1	0	0	0	0	0	0	9	0	0
0	1	0	0	0	0	0	0	0	0	0
0	0	0	0	0	0	0	0	12	0	0
0	0	0	0	0	0	0	0	14	0	0
0	1	0	0	0	0	0	0	8	0	0
0	1	0	0	0	0	0	0	6	0	0
0	0	0	0	1	0	0	0	7	0	0
0	0	0	0	0	0	0	0	2	0	0
0	0	0	0	0	0	0	0	5	0	0
0	0	0	0	1	0	0	0	23	0	0
0	0	0	0	1	0	0	0	30	0	0
0	0	0	0	1	0	0	0	25	0	0
0	0	0	0	1	0	0	0	15	0	0
0	0	0	0	0	0	0	0	10	0	0
0	0	0	0	0	0	0	0	19	0	0
0	0	0	0	0	0	0	0	19	0	0
0	0	0	0	2	0	0	0	8	0	0
0	0	0	0	1	0	0	0	5	0	0
0	0	0	0	0	0	0	0	30	0	0

0	0	0	0	0	0	0	0	19	0	0
0	0	0	0	0	0	0	0	15	0	0
0	0	0	0	1	0	0	0	7	0	0
0	0	0	0	0	0	0	0	8	0	0
0	0	0	0	0	0	0	0	5	0	0
0	0	0	0	0	0	0	0	4	0	0
0	0	0	0	0	0	0	0	2	0	0
0	0	0	0	0	0	0	0	2	0	0
0	0	0	0	0	0	0	0	1	0	0
0	0	0	0	0	0	0	0	7	0	0
0	0	0	0	0	0	0	0	1	0	0
0	0	0	0	0	0	0	0	2	0	0
0	0	0	0	0	0	0	0	4	0	0
0	0	0	0	1	0	0	0	1	0	0
0	0	0	0	1	0	0	0	0	0	0
0	0	0	0	1	0	1	0	2	0	0
0	0	0	0	0	0	0	0	1	0	0
0	0	0	0	0	0	1	0	0	0	0
0	0	0	0	0	0	0	0	1	0	0
0	0	0	0	1	0	0	0	0	0	0
0	0	0	0	5	0	0	0	0	0	0
0	0	0	0	4	0	0	0	0	0	0
0	0	0	0	3	0	0	0	0	0	0

solsil	sphaer	sphaes	squarm	syllid	thelub	torhur	traole	troden	turaot	xymple
0	0	0	0	0	0	0	0	1	0	0
2	0	0	0	0	0	0	0	3	0	0
2	0	0	0	0	0	0	0	1	0	0
1	0	0	0	0	0	0	0	1	0	0

0	0	0	0	0	0	0	0	5	0	0
1	0	0	0	0	0	0	0	3	0	0
1	0	0	0	0	0	0	0	8	0	0
0	0	0	0	0	0	0	0	2	0	0
2	0	0	0	0	0	0	0	0	0	0
2	0	0	0	0	0	0	0	2	0	0
0	0	0	0	0	0	2	0	1	0	0
0	0	0	0	0	0	2	0	0	0	0
0	0	0	0	0	0	0	0	0	0	0
0	0	0	0	0	0	1	0	0	0	0
0	0	0	0	0	0	1	0	0	0	0
0	0	0	0	0	0	0	0	0	0	0
0	0	0	0	0	0	2	0	0	0	0
0	0	0	0	0	0	0	0	0	0	0
0	0	0	0	0	0	0	0	0	0	0
0	0	0	0	0	0	2	0	0	0	0
0	0	0	0	0	0	0	1	2	0	0
0	0	0	0	0	0	0	0	1	0	0
1	0	0	0	0	0	0	0	1	0	0
2	0	0	0	0	0	0	0	4	0	0
1	0	0	0	0	0	0	0	0	0	0
4	0	0	0	0	0	0	0	4	0	0
1	0	0	0	0	0	0	0	1	0	0
3	0	0	0	0	0	0	0	2	0	0
1	0	0	0	0	0	1	0	1	0	0
2	0	0	0	0	0	0	0	1	0	0
1	0	0	0	0	0	0	0	1	0	0
0	2	0	0	0	0	0	0	2	0	0
0	0	0	0	0	0	0	0	0	0	0

2	0	0	0	0	0	0	0	0	0	0
0	0	0	0	0	0	0	1	0	0	0
0	0	0	0	0	0	0	0	1	0	0
0	0	0	1	0	0	0	0	1	0	0
0	0	0	0	0	0	0	0	0	0	0
0	0	0	0	0	0	0	0	3	0	0
0	0	0	0	0	0	0	0	0	0	0
0	1	0	0	0	0	0	0	1	0	0
0	1	0	0	0	0	0	0	0	0	0
0	0	0	0	0	0	0	0	1	0	0
0	0	0	0	0	0	1	0	0	0	0
0	0	0	0	0	0	0	0	0	0	0
0	0	0	0	0	0	0	0	0	0	0
0	1	0	0	0	0	0	0	0	0	0
0	0	0	0	0	0	0	0	0	0	0
0	0	0	0	0	0	0	0	0	0	0
0	0	0	0	0	0	0	0	0	0	0
0	0	0	0	0	0	1	0	2	0	0
0	0	0	0	0	0	1	0	0	0	0
0	0	0	0	0	0	0	0	0	0	0
0	0	0	0	0	0	0	0	0	0	0
0	0	0	0	0	0	0	0	0	0	0
0	0	0	0	0	0	0	0	0	0	0
0	0	0	0	0	0	1	0	0	0	0
0	0	0	0	0	0	1	0	0	0	0
0	0	0	0	0	0	0	0	0	0	0
0	0	0	0	0	0	0	0	0	0	0
0	0	0	0	0	0	0	0	0	0	0
0	0	0	0	0	0	2	0	0	0	0

0	0	0	0	0	0	0	0	0	0	0
0	2	0	0	0	0	0	0	0	0	0
0	0	0	0	0	0	0	0	0	0	0
0	0	0	0	0	1	0	0	2	0	0
0	0	0	0	0	0	0	0	0	0	0
0	0	0	0	0	0	2	0	0	0	0
0	0	0	0	0	0	1	0	0	0	0
0	0	0	0	0	0	2	0	0	0	0
0	0	0	0	0	0	0	0	0	0	0
0	0	0	0	0	0	0	0	0	0	0
0	0	0	0	0	0	0	0	0	0	0
0	0	0	0	0	0	3	0	0	0	0
0	0	0	0	0	0	1	0	0	0	0
0	0	0	0	0	0	1	0	0	0	0
0	0	0	0	0	0	4	0	0	0	0
0	0	0	0	0	0	3	0	0	0	0
0	0	0	0	0	0	0	0	0	0	0
0	0	0	0	0	0	0	0	0	0	0
0	0	0	0	0	0	1	0	0	0	0
0	0	0	0	0	0	0	0	0	0	0
0	0	0	0	0	0	0	0	0	0	0
0	0	0	0	0	0	3	0	0	0	0
0	1	0	0	0	0	1	0	0	0	0
0	0	0	0	0	0	2	0	0	0	0
0	0	0	0	0	0	0	0	0	0	0
0	0	0	0	0	0	2	0	0	0	0
0	0	0	0	0	0	0	0	0	0	0
0	0	0	0	0	0	6	0	0	0	0
0	0	0	0	0	0	0	0	0	0	0

0	0	0	0	0	0	1	0	0	0	0
0	0	0	0	0	0	2	0	0	0	0
0	0	0	0	0	0	4	0	0	0	0
0	0	0	0	0	0	1	0	0	0	0
0	0	0	0	0	0	3	0	0	0	0
0	0	0	0	0	0	2	0	0	0	0
0	0	0	0	0	1	3	0	0	0	0
0	0	0	0	0	0	0	0	0	0	0
0	0	0	0	1	0	3	0	0	0	0
0	0	0	0	0	0	6	0	0	0	0
0	0	0	0	0	0	3	0	0	0	0
0	1	0	0	0	0	2	0	0	0	0
0	0	0	0	0	0	2	0	0	0	0
0	0	0	0	0	0	2	0	0	0	0
0	0	0	0	0	0	0	0	0	0	0
0	0	0	0	0	0	5	0	0	0	0
0	0	0	0	0	0	0	0	0	0	0
0	0	0	0	0	0	1	0	0	0	0
0	0	0	0	0	0	2	0	0	0	0
0	3	0	0	0	0	2	0	0	0	0
0	0	0	0	0	0	1	0	1	0	0
0	0	0	0	0	0	1	0	0	0	0
0	0	0	0	0	0	3	0	0	0	0
0	0	0	0	0	0	2	0	1	0	0
0	0	0	0	0	0	0	0	0	0	0
0	0	0	0	0	0	2	0	0	0	0
0	0	0	0	0	0	1	0	0	0	0
0	0	0	0	0	0	0	0	0	0	0
0	0	0	0	0	0	2	0	0	0	0

0	0	0	0	0	0	2	0	0	0	0
0	0	0	0	0	0	1	0	0	0	0
0	0	0	0	0	0	0	0	0	0	0
0	0	0	0	0	0	1	0	0	0	0
0	0	0	0	0	0	0	0	0	0	0
0	0	0	0	0	0	0	0	0	0	0
0	0	0	0	0	0	0	0	0	0	0
0	0	0	0	0	0	0	0	0	0	0
0	0	0	0	0	0	0	0	0	0	0
0	0	0	0	0	0	2	0	0	0	0
1	0	0	0	0	0	3	0	0	0	0
0	0	0	0	0	0	0	0	0	0	0
0	0	0	0	0	0	0	0	0	0	0
0	0	0	0	0	0	2	0	0	0	0
0	0	0	0	0	0	0	0	0	0	0
0	0	0	0	0	0	0	0	0	0	0
0	0	0	0	0	0	3	0	0	0	0
0	0	0	0	0	0	1	0	0	0	0
0	0	0	0	0	0	1	0	1	0	0
0	0	0	0	0	0	0	0	0	0	0
0	0	0	0	0	0	0	0	0	0	0
0	0	0	0	0	0	0	0	0	0	0
0	0	0	0	0	0	1	0	0	0	0
0	0	0	0	0	0	1	0	0	0	0
0	0	0	0	0	0	1	0	1	0	0
0	0	0	0	0	0	0	0	1	0	0
0	0	0	0	0	0	0	0	0	0	0
0	0	0	0	1	0	0	0	1	0	0
0	0	0	0	0	0	0	0	0	0	1

0	0	0	0	0	0	0	0	0	0	0
0	0	0	0	0	0	0	0	0	0	0
0	0	0	0	0	0	0	0	0	0	0
0	0	0	0	0	0	0	0	0	0	0
0	0	0	0	0	0	1	0	0	0	0
0	0	0	0	0	0	0	0	1	0	0
0	0	0	0	0	0	0	0	0	0	0
0	0	0	0	0	0	0	0	0	0	0
0	0	0	0	0	0	0	0	0	0	0
0	0	0	0	0	0	0	0	0	0	0
0	0	0	0	0	0	0	0	0	0	0
0	0	0	0	0	0	0	0	0	0	0
0	0	0	0	0	0	0	0	0	0	0
0	0	0	0	0	0	0	0	0	0	0
0	0	0	0	0	0	0	0	0	0	0
0	0	0	0	0	0	0	0	0	0	0
0	0	0	0	0	0	0	0	0	0	0
0	0	0	0	0	0	0	0	0	0	0
0	0	0	0	0	0	0	0	0	0	0
0	0	0	0	0	0	0	0	0	0	0
0	0	0	0	0	0	0	0	1	0	0
0	0	0	0	0	0	0	0	0	0	0
0	0	0	0	0	0	0	0	0	0	0
0	0	0	0	0	0	0	0	0	0	0
0	0	0	0	1	0	0	0	0	0	0
0	0	0	0	0	0	1	0	0	0	0
0	0	0	0	0	0	0	0	0	0	0
0	0	0	0	0	0	0	0	0	0	0
0	0	0	0	0	0	0	0	0	0	0

0	0	0	0	0	0	0	0	0	0	0
0	0	0	0	0	0	0	0	0	0	0
0	0	0	0	0	0	0	0	0	0	1
0	0	0	0	0	0	0	0	0	0	0
0	0	0	0	0	0	0	0	0	0	0
0	0	0	0	0	0	1	0	1	0	0
0	0	0	0	0	0	0	0	0	0	0
0	0	0	0	0	0	0	0	1	0	0
0	0	0	0	0	0	0	0	0	0	0
0	0	0	0	0	0	0	0	0	0	0
0	0	0	0	0	0	0	0	1	0	0
0	0	0	0	0	0	0	0	0	0	0
0	0	0	0	0	0	0	0	2	0	0
0	0	0	0	0	0	0	1	0	0	0
0	0	0	0	0	0	0	0	1	0	0
0	0	0	0	0	0	0	0	0	0	0
0	0	0	0	0	0	0	0	0	0	0
0	0	0	0	0	0	0	0	1	0	0
0	0	0	0	0	0	0	0	0	0	0
0	0	0	0	0	0	0	0	0	0	0
1	0	0	0	0	0	0	0	0	0	0
0	0	0	0	0	0	0	0	1	0	0
0	0	0	0	0	0	0	0	0	0	0
0	0	0	0	0	0	0	0	0	0	0
0	0	0	0	0	0	0	0	1	0	0
0	0	0	0	0	0	0	0	1	0	0
0	0	0	0	0	0	0	0	0	0	0
0	0	0	0	0	0	0	0	0	0	0
0	0	0	0	0	0	0	0	0	0	0

0	0	0	0	0	0	0	0	1	0	0
0	0	0	0	0	0	0	0	0	0	0
0	0	0	0	0	0	0	0	0	0	0
0	0	0	0	0	0	0	0	0	0	0
0	0	0	0	0	0	0	0	0	0	0
0	0	0	0	0	0	1	0	0	0	0
0	0	0	0	0	0	0	0	0	0	0
0	1	0	0	0	0	0	0	0	0	0
0	0	0	0	0	0	0	0	1	0	0
0	0	0	0	0	0	0	0	0	0	0
0	0	0	0	0	0	0	0	0	0	0
0	0	0	0	0	0	0	0	0	0	0
0	0	0	0	0	0	0	0	1	0	0
1	1	0	0	0	0	0	0	0	0	0
0	0	0	0	0	0	0	0	0	0	0
1	0	0	0	0	0	0	0	1	0	0
0	0	0	0	0	0	0	0	0	0	0
0	0	0	0	0	0	0	0	2	0	0
0	0	0	0	0	0	0	0	0	0	0
0	0	0	0	0	0	0	0	0	0	0
0	1	0	0	0	0	0	0	2	0	0
0	0	0	0	0	0	0	0	0	0	0
0	1	0	0	0	0	0	0	0	0	0
0	0	0	0	0	0	0	0	0	0	0
0	1	0	0	0	0	0	0	0	0	0
0	1	0	0	0	0	0	0	0	0	0
0	0	0	0	0	0	0	0	0	0	0
0	0	0	0	0	0	0	0	1	0	0
0	1	0	0	0	0	0	0	0	0	0

0	0	0	0	0	0	0	0	1	0	0
0	0	0	0	0	0	0	0	0	0	0
0	0	0	0	0	0	0	0	1	0	0
0	0	0	0	0	0	0	0	0	0	1
0	0	0	0	0	0	0	0	0	0	0
0	0	0	0	0	0	0	0	1	0	0
0	0	0	0	0	0	0	0	1	0	0
0	0	0	0	0	0	0	0	2	0	0
0	0	0	0	0	0	0	0	0	0	0
0	0	0	0	0	0	0	0	0	0	0
0	0	0	0	0	0	0	0	0	0	0
0	0	0	0	0	0	0	0	1	0	0
0	0	0	0	0	0	0	0	0	0	0
0	0	0	0	0	0	0	0	1	0	0
0	0	0	0	0	0	0	0	0	0	0
0	0	0	0	0	0	0	0	0	0	0
0	0	0	0	0	0	0	0	0	0	0
0	0	0	0	0	0	0	0	1	0	0
0	0	0	0	0	0	0	0	3	0	0
0	0	0	0	0	0	0	0	0	0	0
0	0	0	0	0	0	0	0	2	0	0
0	0	0	0	0	0	0	0	0	0	0
0	0	0	0	0	0	0	0	0	0	0
0	0	0	0	0	0	1	0	0	0	0
0	0	0	0	0	0	2	0	0	0	0
0	0	0	0	0	0	0	0	0	0	0
0	0	1	0	0	0	0	0	0	0	0
0	0	0	0	1	0	0	0	0	0	0
0	0	0	0	0	0	1	0	0	0	0

0	0	0	0	0	0	0	0	0	0	0
0	0	0	0	0	0	1	0	0	0	0
0	0	0	0	0	0	1	0	0	0	0
0	0	0	0	0	0	4	0	0	0	0
0	0	0	0	0	0	0	0	0	0	0
0	0	0	0	0	0	0	0	0	0	0
0	0	0	0	0	0	0	0	0	0	0
0	0	0	0	0	0	0	0	0	0	0
0	0	0	0	0	0	0	0	0	1	0
0	0	0	0	1	0	0	0	0	0	0
0	0	0	0	0	0	0	0	0	0	0
0	0	0	0	0	0	0	0	0	0	0
0	0	0	0	0	0	1	0	0	0	0
0	1	0	0	0	0	1	0	0	0	0
0	0	0	0	0	0	1	0	0	0	0
0	0	0	0	0	0	1	0	0	0	0
0	0	0	0	0	0	1	0	0	0	0
0	0	0	0	0	0	8	0	1	0	0
0	0	0	0	0	0	1	0	0	0	0
0	0	0	0	0	0	0	0	0	0	0
0	0	0	0	0	0	1	0	0	0	0
0	0	0	0	0	0	0	0	0	0	0
0	0	0	0	0	0	0	0	0	0	0
0	0	0	0	0	0	1	0	0	0	0
0	0	0	0	0	0	0	0	0	0	0
0	0	0	0	0	0	0	0	0	0	0
0	0	0	0	0	0	0	0	0	0	0
0	0	0	0	0	0	1	0	0	0	0
0	0	0	0	0	0	1	0	0	0	0

0	0	0	0	0	0	0	0	0	0	0
0	0	0	0	0	0	2	0	0	0	0
0	0	0	0	0	0	1	0	1	0	0
0	0	0	0	0	0	2	0	0	0	0
0	0	0	0	0	0	0	0	0	0	0
0	0	0	0	0	0	0	0	0	0	0
0	0	0	0	0	0	0	0	0	0	0
0	0	0	0	0	0	0	0	0	0	0
0	0	0	0	0	0	0	0	0	0	0
0	0	0	0	0	0	0	0	0	0	0
0	0	0	0	0	0	0	0	0	0	0
0	0	0	0	0	0	2	0	0	0	0
0	0	0	0	0	0	2	0	0	0	0
0	0	0	0	0	0	3	0	0	0	0
0	0	0	0	0	0	0	0	0	0	0
0	0	0	0	0	0	0	0	0	0	0
0	0	0	0	0	0	2	0	0	0	0
0	0	0	0	0	0	1	0	0	0	0
0	0	0	0	0	0	0	0	0	0	0
0	0	0	0	0	0	2	0	0	0	0
0	1	0	0	0	0	0	0	0	0	0
0	0	0	0	0	0	0	0	1	0	0
0	0	0	0	0	0	0	0	0	0	0
0	0	0	0	0	0	1	0	0	0	0
0	0	0	0	0	0	1	0	0	0	0
0	0	0	0	0	0	0	0	0	0	0
0	1	0	0	0	0	0	0	0	0	0
0	0	0	0	0	0	0	0	1	0	0
0	1	0	0	0	0	1	0	0	0	0

0	0	1	0	0	0	2	0	0	0	0
0	0	0	0	0	0	2	0	0	0	0
0	0	0	0	0	0	0	0	0	0	0
0	0	0	0	0	0	1	0	0	0	0
0	0	0	0	0	0	0	0	0	0	0
0	0	0	0	0	0	1	0	0	0	0
0	0	0	0	0	0	0	0	0	0	0
0	0	0	0	0	0	2	0	0	0	0
0	0	0	0	0	0	0	0	0	0	0
0	0	0	0	0	0	1	0	0	0	0
0	0	0	0	0	0	0	0	0	0	0
0	0	0	0	0	0	0	0	0	0	0
0	0	0	0	0	0	1	0	0	0	0
0	0	0	0	0	0	2	0	0	0	0
0	0	0	0	0	0	0	0	2	0	0
0	0	0	0	0	0	1	0	0	0	0
0	0	0	0	0	0	1	0	0	0	0
0	0	0	0	0	0	0	0	0	0	0
0	0	0	0	0	0	0	0	0	0	0
0	0	0	0	0	0	0	0	0	0	0
0	0	0	0	0	0	1	0	0	0	0
0	0	0	0	0	0	0	0	0	0	0
0	0	0	0	0	0	2	0	0	0	0
0	0	0	0	0	0	0	0	0	0	0
0	0	0	0	0	0	4	0	0	0	0
0	0	0	0	0	0	0	0	0	0	0
0	1	0	0	0	0	0	0	1	0	0
0	0	0	0	0	0	0	0	0	0	0
0	0	0	0	0	0	0	0	1	0	0

0	1	0	0	0	0	0	0	0	0	0
0	0	0	0	0	0	0	0	1	0	0
0	0	0	0	0	0	0	0	0	0	0
0	0	0	0	0	0	0	0	0	0	0
0	0	0	0	0	0	0	0	0	0	0
0	0	0	0	0	0	0	0	2	0	0
0	0	0	0	0	0	0	0	0	0	0
0	0	0	0	0	0	0	0	0	0	0
0	1	0	0	0	0	1	0	0	0	0
0	0	0	0	0	0	0	0	2	0	0
0	0	0	0	0	0	0	0	0	0	0
0	1	0	0	0	0	0	0	0	0	0
0	0	0	0	0	0	0	0	1	0	0
0	0	0	0	0	0	0	0	0	0	0
0	0	0	0	0	0	0	0	0	0	0
0	0	0	0	0	0	0	0	0	0	0
0	0	0	0	0	0	0	0	3	0	0
0	5	0	0	0	0	0	0	1	0	0
0	0	0	0	0	0	0	0	1	0	0
0	0	0	0	0	0	0	0	1	0	0
0	0	0	0	0	0	0	0	2	0	0
0	0	0	0	0	0	0	0	1	0	0
0	0	0	0	0	0	0	0	0	0	0
0	0	0	0	1	0	0	0	0	0	0
0	0	0	0	0	0	0	0	0	0	0
0	1	0	0	0	0	0	0	0	0	0
0	0	0	0	0	0	0	0	0	0	0
0	0	0	0	0	0	0	0	1	0	0
0	0	0	0	0	0	0	0	1	0	0

0	0	0	0	0	0	0	0	0	0	0
0	0	0	0	0	0	0	0	0	0	0
0	0	0	0	0	0	0	0	0	0	0
0	0	0	0	0	0	0	0	0	0	0
0	0	0	0	0	0	0	0	0	0	0
0	0	0	0	0	0	0	0	0	0	0
0	0	0	0	1	0	0	0	3	0	0
0	0	0	0	0	0	0	0	0	0	0
0	0	0	0	0	0	0	0	0	0	0
0	0	0	0	0	0	0	0	0	0	0
0	0	0	0	0	0	0	0	0	0	0
0	0	0	0	0	0	0	0	0	0	0
0	0	0	0	0	0	0	0	0	0	0
0	0	0	0	0	0	0	0	0	0	0
0	1	0	0	0	0	1	0	0	0	0
0	1	0	0	0	1	0	0	1	0	0
0	0	0	0	0	0	0	0	1	0	0
0	0	0	0	0	0	0	0	2	0	0
0	0	0	0	0	0	0	0	0	0	0

torhur	traole	troden	turaot	waibre	xymple	zealut	zoeasp
0	1	0	0	1	0	0	0
0	0	2	0	0	0	0	0
0	1	2	0	0	0	3	0
1	0	1	0	0	0	0	0
0	0	3	0	1	0	1	0
0	0	1	0	0	0	0	0
0	0	0	0	0	0	0	0
0	0	5	0	0	0	0	0
0	0	1	0	0	0	0	0
0	0	1	0	0	0	1	0
0	0	2	0	0	0	0	0
0	0	0	0	1	0	0	0
0	1	0	0	1	0	1	0
0	0	0	0	1	0	0	0
0	0	1	0	0	0	0	1
0	0	0	0	0	0	0	0
0	0	0	0	0	0	0	0
0	0	0	0	0	0	0	0
0	0	1	0	0	0	0	0
0	0	2	0	0	0	1	0
0	0	0	0	2	0	0	0
0	0	1	0	0	0	0	1
1	1	2	0	0	0	3	0
1	0	1	0	0	0	1	0
0	0	0	0	0	0	0	0
0	0	0	0	0	0	0	0
0	0	0	0	0	0	0	0
0	0	1	0	0	0	1	0

0	1	0	0	0	0	1	0
0	0	0	0	0	0	0	0
0	0	0	0	0	0	0	0
0	0	1	0	0	0	0	0
0	0	0	0	0	0	0	0
0	0	1	0	0	0	0	0
0	0	0	0	0	0	0	0
0	0	2	0	0	0	0	0
0	0	0	0	0	0	0	0
0	0	0	0	0	0	0	0
0	0	0	0	0	0	0	0
0	0	2	0	0	0	0	0
0	0	0	0	0	0	0	0
0	0	1	0	0	0	0	0
2	1	0	0	0	0	2	0
0	1	0	0	0	0	0	0
0	0	4	0	0	0	0	0
0	0	4	0	0	0	1	0
0	1	1	0	0	0	0	0
0	0	0	0	0	0	1	1
0	0	1	0	0	0	1	1
0	0	0	0	0	0	0	0
0	0	2	0	0	0	0	0
0	0	0	0	0	0	0	0
0	0	0	0	0	0	0	0
0	0	0	0	0	0	0	0
0	0	0	0	0	0	0	0
0	0	0	0	0	0	0	0
0	0	1	0	0	0	0	0

0	0	0	0	0	0	0	0
0	0	0	0	0	0	0	0
0	0	1	0	0	0	0	0
0	0	0	0	0	0	0	0
0	1	0	0	1	0	0	0
0	0	0	1	0	0	0	0
0	0	0	0	0	0	0	0
0	1	0	0	0	0	0	0
0	0	0	0	1	0	0	0
0	0	0	0	0	0	0	0
0	0	0	0	0	0	0	0
0	0	0	0	1	0	0	0
0	0	0	0	0	0	0	0
0	0	0	0	1	0	0	0
0	0	0	0	0	0	0	0
0	0	0	0	0	0	0	0
0	0	1	0	1	0	0	0
0	0	0	0	1	0	0	0
0	0	0	0	1	0	0	0
0	0	3	0	0	0	0	0
0	0	0	0	4	0	0	0
5	0	0	0	0	0	0	0
0	0	0	0	0	0	0	0
1	0	0	0	0	0	0	0
0	0	0	0	0	0	0	0
0	0	0	0	1	0	0	0
0	0	0	0	0	0	0	0
0	0	0	0	1	0	0	0
0	0	2	0	0	0	0	0

0	0	0	0	0	0	0	0
0	0	0	0	0	0	0	0
0	0	0	0	0	0	0	0
0	0	0	0	0	1	1	0
0	0	0	0	0	0	0	0
0	0	0	0	0	0	0	0
0	0	0	0	0	0	0	0
0	0	0	0	0	0	0	0
0	0	0	0	1	0	0	0
0	1	1	0	0	0	0	0
0	0	0	0	1	0	0	0
0	0	0	0	1	0	0	0
0	0	0	0	1	0	0	0
0	1	0	0	0	0	8	0
0	0	0	0	0	0	1	0
1	0	0	0	0	0	2	0
0	0	0	0	0	0	0	0
0	1	0	0	0	0	0	0
0	0	0	0	0	0	1	0
0	0	1	0	0	0	0	0
0	0	0	0	0	0	2	0
0	0	1	0	0	0	0	0
0	0	0	0	0	0	0	0
0	0	1	0	0	0	0	0
1	0	0	0	0	0	0	0
0	0	0	0	1	0	0	0
0	0	0	0	1	0	0	0
0	0	0	0	1	0	0	0
0	0	0	0	0	0	0	0

0	0	1	0	0	0	0	1
0	0	0	0	0	0	1	0
1	2	1	0	0	0	1	0
0	0	0	0	0	0	0	0
0	1	0	0	0	0	0	0
0	0	0	0	0	0	1	0
1	0	2	0	0	0	0	0
0	1	0	0	0	0	0	0
1	1	3	0	0	0	0	0
0	0	0	0	0	0	1	0
0	3	0	0	0	0	0	0
0	0	0	0	0	1	0	0
0	0	2	0	0	0	0	0
0	0	1	0	0	0	0	0
0	0	0	0	0	0	0	0
0	0	1	0	0	0	2	0
0	0	1	0	0	0	1	1
0	0	1	0	0	0	0	0
0	0	1	1	0	0	0	0
0	0	0	0	0	0	2	0
0	0	3	0	0	0	2	0
0	0	0	0	0	0	0	0
0	0	1	0	0	0	0	0
0	0	1	0	0	0	0	0
0	1	1	0	0	0	0	0
0	0	0	0	0	0	0	1
0	0	0	0	0	0	0	0
0	0	0	0	0	0	1	0
0	0	0	0	0	0	0	0

0	0	0	0	0	0	0	0
0	0	4	0	0	0	1	0
0	0	1	0	0	0	1	1
1	1	1	0	0	0	0	0
0	1	0	0	0	0	4	0
0	0	1	0	0	0	1	0
0	1	1	0	0	0	2	0
0	2	4	0	0	0	5	1
0	0	5	0	0	0	2	0
0	2	2	0	0	0	1	0
0	0	0	0	0	0	3	0
0	0	1	0	0	0	0	0
1	0	2	0	0	0	0	0
0	0	4	0	0	0	0	0
0	0	0	0	0	0	2	0
1	0	0	0	0	0	3	0
0	0	0	0	0	0	5	0
0	0	1	0	0	0	0	0
0	0	0	0	0	0	0	0
1	0	0	0	0	0	0	0
0	0	0	0	0	0	0	0
0	0	0	0	0	0	0	0
0	0	0	0	0	0	0	0
0	0	0	0	0	0	0	0
0	0	2	0	0	0	1	0
0	1	2	0	0	0	4	0
1	1	0	0	0	0	0	0
0	2	0	0	0	0	1	0
3	1	1	0	0	0	0	0

0	0	2	0	0	0	3	0
0	2	1	0	0	0	1	0
0	0	0	0	0	0	0	1
0	1	0	0	0	0	0	0
0	1	1	0	0	0	1	0
0	0	0	0	0	0	2	0
0	1	3	0	0	0	0	1
0	0	0	0	0	0	0	0
0	2	0	0	1	0	0	0
0	2	1	0	0	0	0	0
1	1	4	0	1	0	2	0
0	1	0	0	0	0	1	0
0	0	2	0	0	0	0	1
0	2	0	0	0	0	0	0
0	3	0	0	1	0	0	0
0	1	1	0	0	0	0	0
0	4	3	0	0	0	1	0
0	1	0	0	0	0	4	0
0	0	0	0	0	0	1	0
0	0	0	0	1	0	0	0
1	0	0	0	0	0	0	0
1	0	0	0	0	0	0	0
0	0	0	0	0	0	1	0
0	0	0	0	0	0	0	0
0	0	1	0	0	0	7	0
0	0	1	0	0	0	2	0
1	0	0	0	0	0	1	0
1	0	0	0	0	0	1	0
0	0	0	0	1	0	0	0

0	0	0	0	0	0	0	0
0	0	0	0	0	0	0	0
2	0	0	0	0	0	0	0
0	0	0	0	0	0	0	0
1	0	0	0	0	0	0	0
1	0	0	0	1	0	0	0
0	0	0	0	0	0	1	0
1	0	0	0	0	0	0	0
0	0	0	0	0	0	0	0
0	0	0	0	2	0	0	0
0	0	0	0	0	0	0	0
0	0	0	0	0	0	0	0
1	0	0	0	0	0	1	0
1	0	0	0	1	0	0	1
0	1	0	0	0	0	0	0
0	0	0	0	1	0	0	0
1	0	3	0	0	0	3	0
0	0	1	0	0	0	0	0
0	0	0	0	0	0	0	0
0	0	0	0	0	0	1	0
0	0	0	0	0	0	0	0
1	0	0	0	0	0	0	0
0	0	0	0	0	0	0	1
0	0	0	0	0	0	0	0
0	0	0	0	1	0	1	0
1	0	0	0	0	0	0	0
0	0	0	0	0	0	0	0
0	0	0	0	0	0	1	0
0	0	0	0	0	0	0	1

0	0	0	0	0	0	0	0
0	0	1	0	0	0	0	0
0	0	0	0	0	0	0	0
0	1	1	0	0	0	2	0
0	0	0	0	0	0	2	0
0	1	0	0	2	0	0	0
0	0	0	0	0	0	0	0
0	1	0	0	2	0	1	0
0	0	0	0	1	0	0	0
0	1	1	0	2	0	1	0
0	1	0	0	0	0	1	0
0	0	0	0	0	0	0	0
0	0	0	0	0	0	0	0
0	1	2	0	0	0	0	0
0	0	3	0	0	0	0	0
0	0	2	0	0	0	1	0
0	0	1	0	1	0	1	0
0	1	1	0	0	0	0	0
0	1	0	0	1	0	0	0
0	1	1	0	0	0	1	0
0	0	1	0	0	0	1	0
0	0	0	0	0	0	0	0
0	0	0	0	0	0	1	0
0	0	2	0	1	0	0	0
0	0	0	0	0	0	0	0
0	0	4	0	0	0	0	0
0	0	1	0	0	0	0	0
0	0	0	0	0	0	1	0
0	0	0	0	0	0	0	0

0	0	0	0	0	0	0	0
0	0	0	0	0	0	1	0
0	0	0	0	0	1	2	0
0	0	1	0	0	0	0	0
0	0	0	0	0	0	0	0
0	0	3	0	0	0	1	0
0	0	2	0	0	0	0	0
0	1	1	0	0	0	0	0
0	0	1	0	0	0	0	0
0	0	0	0	0	0	0	0
0	0	0	0	0	0	2	0
0	0	0	0	0	0	0	0
0	0	0	0	0	0	0	0
0	0	0	0	0	0	0	0
0	0	1	0	0	0	0	0
0	0	0	0	0	0	0	0
0	0	0	0	0	0	0	0
0	0	0	0	0	0	0	0
0	0	0	0	0	0	0	0
0	0	0	0	1	0	0	0
0	0	0	0	0	0	0	0
0	0	0	0	0	0	0	0
0	0	0	0	0	0	0	0
0	0	0	0	0	0	0	0
0	0	0	0	0	0	0	0
0	0	0	0	0	0	0	0
0	0	0	0	0	0	0	0
0	0	0	0	0	0	0	0
0	0	0	0	0	0	0	0

0	0	0	0	0	0	0	0
0	0	1	0	0	0	0	0
0	0	0	0	0	0	0	0
0	0	0	0	0	0	0	0
0	0	0	0	0	0	0	0
0	0	0	0	0	0	0	0
0	0	0	0	0	0	0	0
0	0	0	0	0	0	0	0
0	0	0	0	0	0	0	0
0	0	0	0	0	0	0	0
0	0	0	0	0	0	0	0
0	0	0	0	0	0	0	0
0	0	0	0	0	0	0	0
0	0	0	0	0	0	0	0
0	0	0	0	0	0	0	0
0	0	0	0	0	0	0	0
0	0	0	0	0	0	0	0
0	0	0	0	0	0	0	0
0	0	0	0	0	0	0	0
0	0	0	0	0	0	0	0
0	0	1	0	0	0	0	0
0	0	0	1	0	0	0	0
0	0	0	0	0	0	0	0
0	1	0	0	0	0	0	0
0	0	0	0	0	0	0	0
0	0	3	0	0	0	0	0
0	0	0	0	0	0	0	0
0	0	1	0	0	0	0	0
0	0	2	0	0	0	0	0

0	0	0	0	1	0	0	0
0	0	0	0	0	0	0	0
0	0	0	0	0	0	0	0
0	0	0	0	0	0	1	0
0	0	0	0	0	0	0	0
0	0	0	0	0	0	0	0
0	0	0	0	0	0	0	0
0	0	0	0	0	0	0	0
0	0	0	0	0	0	0	0
0	0	0	0	0	0	0	0
0	0	1	0	0	0	0	0
0	0	0	0	0	0	0	0
0	0	0	0	0	0	0	0
0	0	0	0	0	0	0	0
0	0	0	0	0	0	0	0
0	0	0	0	0	0	0	1
0	0	0	0	0	1	0	0
1	0	1	0	0	0	0	0
0	0	0	0	0	1	0	0
0	0	1	0	0	0	1	0
0	0	0	0	0	0	0	0
0	0	0	0	0	0	0	0
0	0	0	0	2	0	0	0
0	0	1	0	1	0	0	0
0	1	1	0	0	0	0	0
0	1	1	0	1	0	0	0
0	0	0	0	1	0	0	1
0	1	0	0	0	0	1	0
0	2	4	0	1	0	0	0

0	2	1	0	1	0	1	0
0	1	1	0	0	0	0	0
0	0	0	0	1	0	1	0
1	1	1	0	4	0	0	0
0	0	0	0	0	0	0	0
0	0	0	0	0	0	1	0
0	2	1	0	2	0	0	0
0	0	1	0	0	0	0	0
0	0	3	0	0	0	0	0
0	5	1	0	0	0	0	0
0	1	2	0	0	0	0	0
0	0	0	0	1	0	0	0
0	1	1	0	0	0	0	0
0	2	2	0	0	0	0	0
0	0	1	0	1	0	0	0
0	0	2	0	1	0	1	0
0	1	4	0	3	0	0	0
0	0	8	0	2	0	1	0
0	0	2	0	1	0	0	0
0	0	1	0	1	0	0	0
0	2	0	0	0	0	2	0
0	1	1	0	2	0	1	0
1	1	0	0	0	0	0	0
0	0	1	0	0	0	0	1
0	0	0	0	0	0	0	0
2	0	0	0	0	0	0	0
0	1	5	0	1	1	0	0
0	0	0	0	0	0	0	0
0	0	0	0	0	0	0	0

0	0	0	0	0	0	1	0
0	0	0	0	0	0	0	0
0	0	1	0	0	0	1	0
0	0	0	0	0	0	1	0
2	0	0	0	0	0	0	0
0	0	0	0	1	0	0	0
2	0	0	0	0	0	0	0
0	0	0	0	0	0	0	0
1	0	1	0	1	0	0	0
0	0	0	0	1	0	0	0
1	0	0	0	0	0	0	0
0	0	0	0	1	0	0	0
1	0	0	0	1	0	0	0
1	0	0	0	0	0	0	0
1	0	0	0	0	0	0	0
0	0	0	0	1	0	0	0
0	0	0	0	0	0	1	0
0	0	0	0	0	0	0	0
1	0	0	0	0	0	1	0
2	0	0	0	0	0	0	0
0	0	0	0	0	0	0	0
0	0	0	0	0	0	0	0
1	0	0	0	0	0	0	0

zealut	zoeasp
0	1
0	0
0	0
0	0

0	0
0	0
0	0
0	0
1	0
0	0
0	0
0	0
0	0
0	0
0	0
0	0
0	0
0	0
0	0
0	0
1	0
0	0
0	0
0	0
0	0
0	0
0	0
0	0
0	0
2	0
0	0
0	0
0	0

0	0
0	0
0	0
2	0
0	0
0	0
0	0
0	0
1	0
0	0
0	0
0	0
0	0
0	0
2	0
1	0
0	1
0	0
0	0
0	0
0	0
0	0
0	0
0	0
0	0
0	0
0	0
0	0
0	0

0	0
0	0
0	1
0	1
0	0
0	0
0	0
0	0
0	0
0	0
0	0
0	0
0	0
0	0
1	0
0	0
0	0
0	0
1	0
0	0
0	0
3	1
2	0
0	0
0	0
0	1
0	0
0	0
0	0

0	1
0	0
0	0
0	0
0	0
0	0
0	0
0	0
0	0
0	0
0	0
0	0
0	0
0	0
0	0
0	0
0	0
0	0
0	0
0	0
0	0
0	0
0	0
0	0
0	0
0	0
0	0
0	0
0	0

0	0
0	0
0	0
0	0
0	0
0	0
0	0
0	0
0	0
0	0
0	0
0	0
0	0
0	0
0	0
0	0
0	0
0	0
0	0
0	0
0	0
0	0
0	0
0	0
0	0
0	0
0	0
0	1
0	0

0	0
0	0
0	0
0	0
1	0
0	0
0	1
1	0
0	0
1	0
0	1
2	0
0	0
0	0
0	0
0	0
1	0
0	0
0	0
0	0
0	0
0	0
0	0
0	0
1	0
0	0
0	0
0	0
0	0

0	0
0	0
0	0
0	0
0	0
1	0
0	0
0	0
0	0
0	0
0	0
0	0
1	0
0	0
1	0
0	0
0	0
0	0
1	0
0	0
0	0
1	1
0	0
0	0
1	0
0	0
0	0
0	0
0	0

0	0
2	0
0	0
0	0
1	0
0	0
0	0
0	0
1	0
0	0
0	0
1	0
0	0
0	0
0	0
0	0
0	0
0	0
0	0
0	0
0	0
0	0
0	0
0	0
0	0
2	0
0	0
0	0
0	0

0	0
0	0
0	0
0	0
0	0
1	0
0	0
0	0
1	0
1	0
0	0
0	0
0	0
0	0
0	0
0	0
0	1
2	0
2	0
2	0
0	0
1	0
0	0
0	0
0	0
0	0
0	0
0	0
0	0

0	0
0	1
0	0
0	1
0	0
0	0
0	0
0	0
0	0
0	0
0	0
0	0
0	0
0	0
0	0
0	0
0	0
0	0
0	0
0	0
0	0
0	0
0	0
0	0
0	0
0	0
0	0
0	0
0	0

0	0
0	0
0	0
0	0
0	0
0	0
0	0
0	0
0	0
0	0
0	0
0	1
0	0
0	0
0	0
0	0
0	0
0	0
0	0
0	0
0	0
0	0
0	0
0	0
0	0
0	1
0	0
0	0
0	0

0	0
0	0
0	0
0	0
0	0
0	0
0	0
0	0
0	0
0	0
0	0
0	0
0	0
0	1
0	0
0	0
0	0
0	0
0	0
0	0
0	0
0	0
0	0
0	0
0	0
0	0
1	0
1	0
0	0

1	0
1	0
0	0
0	0
0	0
0	0
0	0
0	0
0	0
1	0
0	0
1	0
0	0
0	0
0	0
0	0
0	0
1	0
0	0
0	0
1	0
0	0
0	0
0	0
1	0
0	0
0	0
0	0
0	0

0	0
0	0
1	0
0	0
0	0
0	0
0	0
0	0
0	0
0	0
1	0
0	0
0	0
1	0
0	0
0	0
0	0
0	0
2	0
